# Supplementary material for: Inhibition of O-GlcNAc transferase activates type I interferon-dependent antitumor immunity by bridging cGAS-STING pathway
Source: bioRxiv. 2024 Jun 11:2023.12.14.571787. Originally published 2023 Dec 15. Preprint. [Version 3] doi: 10.1101/2023.12.14.571787 (PMC10760207; doi:10.1101/2023.12.14.571787)
Supplement: Supplement 1 [file media-1.pdf]

										KO+GFP_#1	KO+GFP_#2	KO+GFP_#3	KO+WT_#4	KO+WT_#5	KO+WT_#6	KO+GFP_#1	KO+GFP_#2	KO+GFP_#3	KO+WT_#4	KO+WT_#5	KO+WT_#6
Protein FDR	Accession	Description	# PSMs	# Peptides	# Unique Peptide	MW [kDa]	Coverage	# AAs	calc. pI	# PSMs: A2	# PSMs: B2	# PSMs: C2	# PSMs: D2	# PSMs: E2	# PSMs: F2	# Peptides: A2	# Peptides: B2	# Peptides: C2	# Peptides: D2	# Peptides: E2	# Peptides: F2
High	P35579	Myosin-9 OS=Homo sapiens OX=9606 GN=MYH9 PE=1 SV=4	6611	165	135	226.4	67	1960	5.6	872	926	1036	224	345	233	145	143	140	93	113	96
High	Q9U6Y5	SWISS-PROT:Q9U6Y5 Green fluorescent protein (GFP-Cter-HisTag)	4079	24	24	28.1	69	249	6.52	951	1033	927	200	186	189	23	23	20	10	9	10
High	Q7Z406	Myosin-14 OS=Homo sapiens OX=9606 GN=MYH14 PE=1 SV=2	3222	140	126	227.7	65	1995	5.6	482	489	515	118	168	128	124	123	122	75	84	77
High	O15294	UDP-N-acetylglucosamine--peptide N-acetylglucosaminyltransferase 110 kDa subunit OS=Homo sapiens OX=9606 GN=OGT PE=1 S	2542	54	54	116.9	61	1046	6.7			2	293	483	327			2	37	39	38
High	P49792	E3 SUMO-protein ligase RanBP2 OS=Homo sapiens OX=9606 GN=RANBP2 PE=1 SV=2	2241	182	127	358	67	3224	6.2	8	11	13	40	44	37	8	11	13	39	42	35
High	P60709	Actin, cytoplasmic 1 OS=Homo sapiens OX=9606 GN=ACTB PE=1 SV=1	2101	25	10	41.7	74	375	5.48	246	268	268	120	147	118	21	21	21	16	15	16
High	P05787	SWISS-PROT:P05787 Tax_Id=9606 Gene_Symbol=KRT8 Keratin, type II cytoskeletal 8	1908	49	19	53.7	83	483	5.59	383	423	460	85	106	84	48	46	46	30	31	31
High	Q15149	Plectin OS=Homo sapiens OX=9606 GN=PLEC PE=1 SV=3	1463	228	220	531.5	53	4684	5.96	212	220	226	99	113	101	178	182	182	94	104	97
High	P68133	Actin, alpha skeletal muscle OS=Homo sapiens OX=9606 GN=ACTA1 PE=1 SV=1	1270	19	6	42	54	377	5.39	122	124	161	98	91	96	13	15	14	12	10	14
High	P51610	Host cell factor 1 OS=Homo sapiens OX=9606 GN=HCFC1 PE=1 SV=2	1227	61	61	208.6	44	2035	7.46		1		161	193	173		1		49	54	47
High	P35580	Myosin-10 OS=Homo sapiens OX=9606 GN=MYH10 PE=1 SV=3	1221	109	82	228.9	56	1976	5.54	162	178	176	48	73	53	90	89	90	36	44	37
High	Q13813	Spectrin alpha chain, non-erythrocytic 1 OS=Homo sapiens OX=9606 GN=SPTAN1 PE=1 SV=3	1055	141	141	284.4	68	2472	5.35	165	176	183	37	46	38	113	119	121	37	45	38
High	P78527	DNA-dependent protein kinase catalytic subunit OS=Homo sapiens OX=9606 GN=PRKDC PE=1 SV=3	1009	159	159	468.8	42	4128	7.12	143	144	127	67	73	65	127	126	111	63	71	63
High	Q01082	Spectrin beta chain, non-erythrocytic 1 OS=Homo sapiens OX=9606 GN=SPTBN1 PE=1 SV=2	907	127	116	274.4	63	2364	5.57	147	151	139	27	33	27	101	107	100	26	33	27
High	Q09666	Neuroblast differentiation-associated protein AHNAK OS=Homo sapiens OX=9606 GN=AHNAK PE=1 SV=2	903	172	172	628.7	59	5890	6.15	60	61	90	75	93	76	55	53	77	68	77	68
High	P08727	Keratin, type I cytoskeletal 19 OS=Homo sapiens OX=9606 GN=KRT19 PE=1 SV=4	859	40	25	44.1	79	400	5.14	204	195	217	35	46	37	38	38	38	22	22	22
High	P05783	Keratin, type I cytoskeletal 18 OS=Homo sapiens OX=9606 GN=KRT18 PE=1 SV=2	803	30	20	48	61	430	5.45	161	160	164	44	52	42	29	26	27	19	19	18
High	P60660	Myosin light polypeptide 6 OS=Homo sapiens OX=9606 GN=MYL6 PE=1 SV=2	782	13	9	16.9	85	151	4.65	106	121	122	44	49	47	11	12	13	8	8	9
High	H-INV:HIT	Tax_Id=9606 Gene_Symbol=- Similar to Keratin, type II cytoskeletal 8	771	24	1	49.4	44	443	5.2	163	167	176	32	44	34	24	24	22	14	15	15
High	A6NHR9	Structural maintenance of chromosomes flexible hinge domain-containing protein 1 OS=Homo sapiens OX=9606 GN=SMCHD1 PE-	769	93	93	226.2	49	2005	7.3	5	4	5	91	113	92	5	4	5	62	69	59
High	Q8WWI1	LIM domain only protein 7 OS=Homo sapiens OX=9606 GN=LMO7 PE=1 SV=3	745	73	73	192.6	42	1683	8.09	114	107	108	32	37	33	63	63	56	29	30	30
High	P35749	Myosin-11 OS=Homo sapiens OX=9606 GN=MYH11 PE=1 SV=3	716	26	1	227.2	12	1972	5.5	95	94	105	27	52	32	24	23	20	15	20	17
High	P07437	Tubulin beta chain OS=Homo sapiens OX=9606 GN=TUBB PE=1 SV=2	676	25	4	49.6	73	444	4.89	98	97	88	44	54	46	23	25	24	14	14	13
High	P02545	Prelamin-A/C OS=Homo sapiens OX=9606 GN=LMNA PE=1 SV=1	675	46	45	74.1	69	664	7.02	132	130	144	37	43	40	43	44	45	28	31	34
High	P68371	Tubulin beta-4B chain OS=Homo sapiens OX=9606 GN=TUBB4B PE=1 SV=1	674	25	1	49.8	73	445	4.89	101	98	88	43	52	43	23	25	24	14	14	13
High	Q562R1	Beta-actin-like protein 2 OS=Homo sapiens OX=9606 GN=ACTBL2 PE=1 SV=2	656	12	5	42	36	376	5.59	50	52	83	64	50	62	8	6	8	4	5	5
High	P52732	Kinesin-like protein KIF11 OS=Homo sapiens OX=9606 GN=KIF11 PE=1 SV=2	638	60	60	119.1	53	1056	5.64	69	61	64	70	91	76	49	43	44	44	46	45
High	Q00610	Clathrin heavy chain 1 OS=Homo sapiens OX=9606 GN=CLTC PE=1 SV=5	631	72	72	191.5	52	1675	5.69	81	85	72	36	38	33	66	67	52	31	34	29
High	P04350	Tubulin beta-4A chain OS=Homo sapiens OX=9606 GN=TUBB4A PE=1 SV=2	607	21	1	49.6	62	444	4.88	91	88	80	39	44	38	19	20	20	11	10	11
High	P49327	Fatty acid synthase OS=Homo sapiens OX=9606 GN=FASN PE=1 SV=3	603	85	84	273.3	45	2511	6.44	71	78	71	39	48	48	61	65	61	37	44	44
High	Q9BTC0	Death-inducer obliterator 1 OS=Homo sapiens OX=9606 GN=DIDO1 PE=1 SV=5	600	75	74	243.7	48	2240	7.88		1		77	99	85		1		48	57	56
High	O75369	Filamin-B OS=Homo sapiens OX=9606 GN=FLNB PE=1 SV=2	591	100	94	278	53	2602	5.73	62	73	74	47	49	49	58	66	69	43	46	48
High	Q14974	Importin subunit beta-1 OS=Homo sapiens OX=9606 GN=KPNB1 PE=1 SV=2	584	31	31	97.1	44	876	4.78	13	14	14	8	11	9	12	13	12	8	11	9
High	P11142	Heat shock cognate 71 kDa protein OS=Homo sapiens OX=9606 GN=HSPA8 PE=1 SV=1	571	35	23	70.9	69	646	5.52	50	47	55	63	77	58	29	26	30	22	27	22
High	P15924	Desmoplakin OS=Homo sapiens OX=9606 GN=DSP PE=1 SV=3	543	103	103	331.6	40	2871	6.81	76	75	71	45	46	43	70	69	64	44	45	42
High	Q92614	Unconventional myosin-XVIIIa OS=Homo sapiens OX=9606 GN=MYO18A PE=1 SV=3	537	87	87	233	49	2054	6.3	72	79	73	22	23	22	63	69	62	20	22	20
High	O14950	Myosin regulatory light chain 12B OS=Homo sapiens OX=9606 GN=MYL12B PE=1 SV=2	532	12	12	19.8	63	172	4.84	76	74	74	15	25	16	12	11	11	7	8	7
High	Q13885	Tubulin beta-2A chain OS=Homo sapiens OX=9606 GN=TUBB2A PE=1 SV=1	524	22	4	49.9	67	445	4.89	76	74	71	36	46	38	18	18	18	10	11	9
High	O14715	RANBP2-like and GRIP domain-containing protein 8 OS=Homo sapiens OX=9606 GN=RGPD8 PE=1 SV=2	494	66	2	198.9	41	1765	6.49	2	2	4	9	12	8	2	2	4	9	11	8
High	P38646	Stress-70 protein, mitochondrial OS=Homo sapiens OX=9606 GN=HSPA9 PE=1 SV=2	490	35	35	73.6	58	679	6.16	48	53	56	39	54	41	27	28	31	24	26	25
High	Q99666	RANBP2-like and GRIP domain-containing protein 5/6 OS=Homo sapiens OX=9606 GN=RGPD5 PE=1 SV=3	489	64	2	198.8	38	1765	6.42	2	2	4	9	12	8	2	2	4	9	11	8
High	P21333	Filamin-A OS=Homo sapiens OX=9606 GN=FLNA PE=1 SV=4	474	75	69	280.6	45	2647	6.06	58	46	44	48	49	40	39	35	41	37	39	31
High	P0DJD0	RANBP2-like and GRIP domain-containing protein 1 OS=Homo sapiens OX=9606 GN=RGPD1 PE=2 SV=1	470	45	2	196.5	30	1748	6.14			3	8	11	9			3	8	10	9
High	P07355	Annexin A2 OS=Homo sapiens OX=9606 GN=ANXA2 PE=1 SV=2	465	27	27	38.6	67	339	7.75	44	42	49	40	45	40	24	22	22	18	18	20
High	Q06830	Peroxiredoxin-1 OS=Homo sapiens OX=9606 GN=PRDX1 PE=1 SV=1	450	17	14	22.1	74	199	8.13	42	47	46	46	53	49	16	16	15	13	14	14
High	P98088	Mucin-5AC OS=Homo sapiens OX=9606 GN=MUC5AC PE=1 SV=4	443	91	88	585.2	34	5654	7.02	16	19	11	92	104	96	15	17	11	69	79	69
High	Q14204	Cytoplasmic dynein 1 heavy chain 1 OS=Homo sapiens OX=9606 GN=DYNC1H1 PE=1 SV=5	436	104	104	532.1	27	4646	6.4	49	51	53	18	27	24	49	49	50	18	27	24
High	Q9UM54	Unconventional myosin-VI OS=Homo sapiens OX=9606 GN=MYO6 PE=1 SV=4	431	66	66	149.6	53	1294	8.53	56	61	53	19	21	18	45	48	43	18	21	17
High	Q71U36	Tubulin alpha-1A chain OS=Homo sapiens OX=9606 GN=TUBA1A PE=1 SV=1	430	24	2	50.1	61	451	5.06	55	57	52	22	28	22	22	22	22	13	15	13
High	P04264	Keratin, type II cytoskeletal 1 OS=Homo sapiens OX=9606 GN=KRT1 PE=1 SV=6	418	34	27	66	57	644	8.12	52	42	62	45	45	40	29	26	31	27	24	24
High	Q6N021	Methylcytosine dioxygenase TET2 OS=Homo sapiens OX=9606 GN=TET2 PE=1 SV=3	406	71	68	223.7	46	2002	7.99				67	80	63				49	56	48
High	O15047	Histone-lysine N-methyltransferase SETD1A OS=Homo sapiens OX=9606 GN=SETD1A PE=1 SV=3	405	43	43	185.9	30	1707	5.14				54	70	56				33	37	36
High	P68104	Elongation factor 1-alpha 1 OS=Homo sapiens OX=9606 GN=EEF1A1 PE=1 SV=1	403	18	9	50.1	63	462	9.01	90	84	49	16	29	14	16	17	14	10	12	8
High	Q13509	Tubulin beta-3 chain OS=Homo sapiens OX=9606 GN=TUBB3 PE=1 SV=2	394	17	2	50.4	43	450	4.93	56	50	50	30	32	32	14	15	12	11	10	11
High	P58107	Epiplakin OS=Homo sapiens OX=9606 GN=EPPK1 PE=1 SV=3	386	90	83	555.3	48	5088	5.62	81	75	70	17	23	21	70	62	60	17	18	20
High	Q9BQE3	Tubulin alpha-1C chain OS=Homo sapiens OX=9606 GN=TUBA1C PE=1 SV=1	385	24	3	49.9	63	449	5.1	51	54	48	22	29	22	22	23	22	13	16	13
High	P00761 SV	Trypsin - Sus scrofa (Pig).	381	7	7	24.4	38	231	7.18	30	35	38	37	51	44	5	4	4	3	4	4
High	P19338	Nucleolin OS=Homo sapiens OX=9606 GN=NCL PE=1 SV=3	366	26	26	76.6	38	710	4.7	27	27	27	32	50	28	19	16	17	17	20	16
High	P68366	Tubulin alpha-4A chain OS=Homo sapiens OX=9606 GN=TUBA4A PE=1 SV=1	351	23	6	49.9	59	448	5.06	55	56	44	18	20	17	20	21	20	12	14	11
High	Q9ULV0	Unconventional myosin-Vb OS=Homo sapiens OX=9606 GN=MYO5B PE=1 SV=3	338	68	64	213.5	44	1848	7.2	60	58	53	8	13	11	50	52	47	8	13	11
High	P14618	Pyruvate kinase PKM OS=Homo sapiens OX=9606 GN=PKM PE=1 SV=4	335	27	27	57.9	63	531	7.84	40	42	43	20	28	21	24	23	22	14	18	14
High	P20700	Lamin-B1 OS=Homo sapiens OX=9606 GN=LMNB1 PE=1 SV=2	334	36	33	66.4	63	586	5.16	52	56	63	17	25	20	32	34	33	14	18	18
High	P35900	SWISS-PROT:P35900 Tax_Id=9606 Gene_Symbol=KRT20 Keratin, type I cytoskeletal 20	332	30	27	48.5	69	424	5.69	69	77	63	17	20	16	29	29	28	13	13	14
High	P0DMV9	Heat shock 70 kDa protein 1B OS=Homo sapiens OX=9606 GN=HSPA1B PE=1 SV=1	321	27	23	70	54	641	5.66	33	32	34	24	30	25	20	20	22	16	17	17
High	P35908	Keratin, type II cytoskeletal 2 epidermal OS=Homo sapiens OX=9606 GN=KRT2 PE=1 SV=2	319	41	1	65.4	65	639	8	43	39	91	20	26	24	29	23	40	16	18	17
High	P35908	SWISS-PROT:P35908 Tax_Id=9606 Gene_Symbol=KRT2 Keratin, type II cytoskeletal 2 epidermal	319	41	1	65.8	66	645	8	43	39	91	20	26	24	29	23	40	16	18	17
High	P13010	X-ray repair cross-complementing protein 5 OS=Homo sapiens OX=9606 GN=XRCC5 PE=1 SV=3	302	37	37	82.7	63	732	5.81	51	47	52	14	19	17	34	29	28	11	15	12
High	Q86V48	Leucine zipper protein 1 OS=Homo sapiens OX=9606 GN=LUZP1 PE=1 SV=2	302	47	47	120.2	50	1076	8.5	42	43	48	13	14	15	34	38	38	13	14	15
High	O43707	Alpha-actinin-4 OS=Homo sapiens OX=9606 GN=ACTN4 PE=1 SV=2	287	42	29	104.8	59	911	5.44	45	47	49	11	14	13	32	31	33	10	12	11
High	P13639	Elongation factor 2 OS=Homo sapiens OX=9606 GN=EEF2 PE=1 SV=4	285	40	39	95.3	56	858	6.83	41	35	32	18	33	18	29	24	27	16	20	16
High	Q7Z6Z7	E3 ubiquitin-protein ligase HUWE1 OS=Homo sapiens OX=9606 GN=HUWE1 PE=1 SV=3	280	73	73	481.6	23	4374	5.22	13	12	11	55	63	56	12	11	10	49	54	51
High	P11021	Endoplasmic reticulum chaperone BiP OS=Homo sapiens OX=9606 GN=HSPA5 PE=1 SV=2	278	28	26	72.3	49	654	5.16	25	25	27	26	37	27	19	19	21	20	23	22
High	Q8TDZ2	[F-actin]-monooxygenase MICAL1 OS=Homo sapiens OX=9606 GN=MICAL1 PE=1 SV=2	273	44	44	117.8	52	1067	6.4	68	68	80	6	8	6	37	40	38	5	7	5
High	A2NJV5	Immunoglobulin kappa variable 2-29 OS=Homo sapiens OX=9606 GN=IGKV2-29 PE=3 SV=2	272	3	2	13.1	17	120	7.28	20	23	18	34	51	48	2	2	2	2	2	2
High	P49411	Elongation factor Tu, mitochondrial OS=Homo sapiens OX=9606 GN=TUFM PE=1 SV=2	267	26	26	49.5	59	452	7.61	38	37	41	15	19	20	26	26	23	14	16	17
High	P35527	SWISS-PROT:P35527 Tax_Id=9606 Gene_Symbol=KRT9 Keratin, type I cytoskeletal 9	264	26	25	62.1	57	623	5.3	41	33	38	22	22	24	22	18	22	15	14	16
High	P13645	SWISS-PROT:P13645 Tax_Id=9606 Gene_Symbol=KRT10 Keratin, type I cytoskeletal 10	263	27	23	59.5	50	593	5.21	38	38	64	18	28	22	24	18	25	14	18	17
High	P19474	E3 ubiquitin-protein ligase TRIM21 OS=Homo sapiens OX=9606 GN=TRIM21 PE=1 SV=1	262	26	26	54.1	52	475	6.38	32	30	30	28	32	24	23	22	21	15	17	16
High	P46060	Ran GTPase-activating protein 1 OS=Homo sapiens OX=9606 GN=RANGAP1 PE=1 SV=1	260	29	29	63.5	53	587	4.68	1	1	1	5	6	5	1	1	1	5	6	5
High	P12956	X-ray repair cross-complementing protein 6 OS=Homo sapiens OX=9606 GN=XRCC6 PE=1 SV=2	260	31	31	69.8	52	609	6.64	47	42	37	15	21	19	28	28	28	12	15	15
High	O94832	Unconventional myosin-Id OS=Homo sapiens OX=9606 GN=MYO1D PE=1 SV=2	259	43	43	116.1	45	1006	9.39	29	33	29	13	17	11	28	30	26	13	16	11
High	P27708	CAD protein OS=Homo sapiens OX=9606 GN=CAD PE=1 SV=3	258	54	54	242.8	33	2225	6.46	38	36	40	13	23	20	36	35	34	12	20	20
High	Q9P2E9	Ribosome-binding protein 1 OS=Homo sapiens OX=9606 GN=RRBP1 PE=1 SV=5	258	43	43	152.4	41	1410	8.6	17	19	23	27	30	27	17	18	23	26	28	27
High	Q9NYL9	Tropomodulin-3 OS=Homo sapiens OX=9606 GN=TMOD3 PE=1 SV=1	257	22	22	39.6	62	352	5.19	38	34	34	10	16	12	22	21	21	10	14	11
High	P15822	Zinc finger protein 40 OS=Homo sapiens OX=9606 GN=HIVEP1 PE=1 SV=4	253	62	60	296.7	31	2718	7.84				48	61	48				43	50	44
High	Q05639	Elongation factor 1-alpha 2 OS=Homo sapiens OX=9606 GN=EEF1A2 PE=1 SV=1	251	14	5	50.4	50	463	9.03	71	70	29	12	19	12	12	13	10	6	7	6
High	P10809	60 kDa heat shock protein, mitochondrial OS=Homo sapiens OX=9606 GN=HSPD1 PE=1 SV=2	251	28	28	61	63	573	5.87	31	30	24	17	19	17	18	19	19	14	17	15
High	P05784	SWISS-PROT:P05784 Tax_Id=10090 Gene_Symbol=Krt18 Keratin, type I cytoskeletal 18	248	12	1	47.5	24	423	5.33	49	55	52	14	17	16	11	11	9	7	7	7
High	Q14980	Nuclear mitotic apparatus protein 1 OS=Homo sapiens OX=9606 GN=NUMA1 PE=1 SV=2	248	53	53	238.1	32	2115	5.78	28	30	36	20	21	20	25	28	31	19	21	19
High	Q9UHB6	LIM domain and actin-binding protein 1 OS=Homo sapiens OX=9606 GN=LIMA1 PE=1 SV=1	247	36	36	85.2	58	759	6.84	28	33	33	10	11	10	24	26	24	10	11	10
High	P14649	Myosin light chain 6B OS=Homo sapiens OX=9606 GN=MYL6B PE=1 SV=1	243	12	10	22.8	54	208	5.73	24	29	26	25	22	22	9	10	8	7	8	7
High	P02538	SWISS-PROT:P02538 Tax_Id=9606 Gene_Symbol=KRT6A Keratin, type II cytoskeletal 6A	242	20	5	60	29	564	8	38	40	57	15	15	17	12	10	14	9	8	9
High	REFSEQ:XI	Tax_Id=9606 Gene_Symbol=LOC150739 similar to Keratin, type II cytoskeletal 8	241	4	1	23.1	22	213	7.59	57	57	73	11	8	8	3	3	4	2	2	2
High	P09493	Tropomyosin alpha-1 chain OS=Homo sapiens OX=9606 GN=TPM1 PE=1 SV=2	240	24	10	32.7	52	284	4.74	28	25	32	23	30	23	15	13	17	15	21	14
High	O43151	Methylcytosine dioxygenase TET3 OS=Homo sapiens OX=9606 GN=TET3 PE=1 SV=4	234	54	51	193.6	40	1795	7.81				44	53	47				35	41	38
High	Q14160	Protein scribble homolog OS=Homo sapiens OX=9606 GN=SCRIB PE=1 SV=5	232	49	47	174.8	40	1630	5.07	31	32	35	8	8	9	31	32	34	8	8	9
High	Q9UPQ0	LIM and calponin homology domains-containing protein 1 OS=Homo sapiens OX=9606 GN=LIMCH1 PE=1 SV=4	232	40	40	121.8	46	1083	6.47	38	36	40	9	10	9	30	28	32	9	10	9
High	P08238	Heat shock protein HSP 90-beta OS=Homo sapiens OX=9606 GN=HSP90AB1 PE=1 SV=4	230	30	17	83.2	51	724	5.03	20	27	33	24	21	20	18	18	24	17	18	15
High	P08730-1	SWISS-PROT:P08730-1 Tax_Id=10090 Gene_Symbol=Krt13 Isoform 1 of Keratin, type I cytoskeletal 13	230	9	1	47.7	19	437	4.86	56	56	53	9	14	10	9	9	9	5	6	5
High	P00352	Aldehyde dehydrogenase 1A1 OS=Homo sapiens OX=9606 GN=ALDH1A1 PE=1 SV=2	229	25	22	54.8	56	501	6.73	23	23	29	9	16	9	17	18	18	9	14	9
High	Q3SX28	TREMBL:Q3SX28;Q5KR48 (Bos taurus) Tropomyosin 2	229	21	5	33	46	284	4.69	29	26	30	18	25	17	16	15	16	13	17	12
High	P61978	Heterogeneous nuclear ribonucleoprotein K OS=Homo sapiens OX=9606 GN=HNRNPK PE=1 SV=1	229	19	19	50.9	53	463	5.54	21	24	23	20	25	19	12	14	15	13	14	13
High	Q08211	ATP-dependent RNA helicase A OS=Homo sapiens OX=9606 GN=DHX9 PE=1 SV=4	228	37	37	140.9	36	1270	6.84	24	26	26	20	19	20	22	24	24	18	18	18
High	P22102	Trifunctional purine biosynthetic protein adenosine-3 OS=Homo sapiens OX=9606 GN=GART PE=1 SV=1	226	36	36	107.7	46	1010	6.7	23	25	29	17	18	16	20	21	22	15	14	16
High	P07384	Calpain-1 catalytic subunit OS=Homo sapiens OX=9606 GN=CAPN1 PE=1 SV=1	226	32	32	81.8	45	714	5.67	31	36	37	12	14	11	25	28	24	12	14	11
High	Q92598	Heat shock protein 105 kDa OS=Homo sapiens OX=9606 GN=HSPH1 PE=1 SV=1	225	36	33	96.8	50	858	5.39	20	21	23	16	23	14	18	20	23	15	19	13
High	P63010	AP-2 complex subunit beta OS=Homo sapiens OX=9606 GN=AP2B1 PE=1 SV=1	224	38	23	104.5	47	937	5.38	24	32	31	10	9	14	23	30	26	10	7	13
High	Q13045	Protein flightless-1 homolog OS=Homo sapiens OX=9606 GN=FLII PE=1 SV=2	223	38	38	144.7	38	1269	6.05	34	34	34	7	12	5	26	27	26	6	11	5
High	P23396	40S ribosomal protein S3 OS=Homo sapiens OX=9606 GN=RPS3 PE=1 SV=2	223	19	19	26.7	79	243	9.66	28	29	30	13	19	18	17	16	16	12	11	15
High	P23528	Cofilin-1 OS=Homo sapiens OX=9606 GN=CFL1 PE=1 SV=3	222	14	14	18.5	67	166	8.09	34	32	27	11	17	13	12	12	13	7	9	7
High	P07814	Bifunctional glutamate/proline--tRNA ligase OS=Homo sapiens OX=9606 GN=EPRS1 PE=1 SV=5	219	43	43	170.5	38	1512	7.33	14	19	24	13	19	16	14	18	22	13	18	16
High	P02533	Keratin, type I cytoskeletal 14 OS=Homo sapiens OX=9606 GN=KRT14 PE=1 SV=4	218	19	2	51.5	36	472	5.16	37	40	48	11	17	15	13	12	15	7	9	10
High	O00159	Unconventional myosin-Ic OS=Homo sapiens OX=9606 GN=MYO1C PE=1 SV=4	216	40	40	121.6	43	1063	9.41	32	29	31	15	10	12	30	26	28	15	10	12
High	Q2KHR3	Glutamine and serine-rich protein 1 OS=Homo sapiens OX=9606 GN=QSER1 PE=1 SV=3	214	41	41	189.9	34	1735	7.08				29	46	32				26	33	25
High	O75688	Protein phosphatase 1B OS=Homo sapiens OX=9606 GN=PPM1B PE=1 SV=1	214	19	16	52.6	48	479	5.05	24	29	18	14	30	14	16	18	13	9	14	10
High	Q13085	Acetyl-CoA carboxylase 1 OS=Homo sapiens OX=9606 GN=ACACA PE=1 SV=2	213	57	50	265.4	32	2346	6.37	28	28	32	14	10	14	27	28	31	14	10	14
High	O95782	AP-2 complex subunit alpha-1 OS=Homo sapiens OX=9606 GN=AP2A1 PE=1 SV=3	211	37	27	107.5	43	977	7.03	25	28	20	16	15	11	24	28	19	14	15	11
High	P16615	Sarcoplasmic/endoplasmic reticulum calcium ATPase 2 OS=Homo sapiens OX=9606 GN=ATP2A2 PE=1 SV=1	211	34	29	114.7	36	1042	5.34	28	29	34	13	15	16	25	25	27	12	13	14
High	P00390	Glutathione reductase, mitochondrial OS=Homo sapiens OX=9606 GN=GSR PE=1 SV=2	209	19	19	56.2	61	522	8.5	22	26	22	14	17	11	16	16	14	10	13	9
High	Q9P0U4	CXXC-type zinc finger protein 1 OS=Homo sapiens OX=9606 GN=CXXC1 PE=1 SV=2	206	29	29	75.7	46	656	8.24				31	36	23				22	24	19
High	P62937	Peptidyl-prolyl cis-trans isomerase A OS=Homo sapiens OX=9606 GN=PPIA PE=1 SV=2	205	9	9	18	53	165	7.81	23	18	24	17	29	19	9	8	8	4	5	5
High	Q9BUF5	Tubulin beta-6 chain OS=Homo sapiens OX=9606 GN=TUBB6 PE=1 SV=1	204	10	2	49.8	29	446	4.88	31	25	26	17	18	18	8	8	7	6	5	5
High	P13647	SWISS-PROT:P13647 Tax_Id=9606 Gene_Symbol=KRT5 Keratin, type II cytoskeletal 5	204	26	13	62.3	38	590	7.74	25	25	54	14	14	17	14	8	20	10	9	11
High	Q9NR30	Nucleolar RNA helicase 2 OS=Homo sapiens OX=9606 GN=DDX21 PE=1 SV=5	202	30	28	87.3	39	783	9.28	11	12	13	23	24	20	11	12	12	21	18	17
High	P13646-1	SWISS-PROT:P13646-1 Tax_Id=9606 Gene_Symbol=KRT13 Isoform 1 of Keratin, type I cytoskeletal 13	198	16	8	49.6	34	458	4.96	49	48	47	6	12	5	13	14	12	5	6	4
High	P62805	Histone H4 OS=Homo sapiens OX=9606 GN=H4C1 PE=1 SV=2	198	7	7	11.4	52	103	11.36	20	19	28	24	22	23	5	6	7	6	6	6
High	Q92841	Probable ATP-dependent RNA helicase DDX17 OS=Homo sapiens OX=9606 GN=DDX17 PE=1 SV=2	198	29	21	80.2	47	729	8.27	20	18	19	20	21	19	19	17	18	18	18	17
High	Q04695	SWISS-PROT:Q04695 Tax_Id=9606 Gene_Symbol=KRT17 Keratin, type I cytoskeletal 17	198	18	6	48.1	33	432	5.02	38	42	41	11	20	13	15	16	15	7	12	8
High	Q76L83	Putative Polycomb group protein ASXL2 OS=Homo sapiens OX=9606 GN=ASXL2 PE=1 SV=1	196	41	40	153.7	40	1435	8.81				31	32	30				31	30	30
High	P17844	Probable ATP-dependent RNA helicase DDX5 OS=Homo sapiens OX=9606 GN=DDX5 PE=1 SV=1	196	28	20	69.1	44	614	8.92	23	22	22	18	21	19	21	19	20	16	19	16
High	P06733	Alpha-enolase OS=Homo sapiens OX=9606 GN=ENO1 PE=1 SV=2	195	20	20	47.1	57	434	7.39	18	18	24	19	27	19	13	15	16	12	15	12
High	P22626	Heterogeneous nuclear ribonucleoproteins A2/B1 OS=Homo sapiens OX=9606 GN=HNRNPA2B1 PE=1 SV=2	194	18	16	37.4	56	353	8.95	31	39	31	14	14	14	15	17	14	10	12	11
High	P62424	60S ribosomal protein L7a OS=Homo sapiens OX=9606 GN=RPL7A PE=1 SV=2	193	14	14	30	42	266	10.61	14	14	16	17	15	18	10	12	12	11	12	12
High	Q15233	Non-POU domain-containing octamer-binding protein OS=Homo sapiens OX=9606 GN=NONO PE=1 SV=4	193	18	17	54.2	49	471	8.95	17	19	23	19	28	22	13	14	16	15	16	12
High	Q13126	S-methyl-5'-thioadenosine phosphorylase OS=Homo sapiens OX=9606 GN=MTAP PE=1 SV=2	191	16	16	31.2	73	283	7.18	19	18	22	15	20	14	14	14	14	11	12	12
High	Q6P3W7	SCY1-like protein 2 OS=Homo sapiens OX=9606 GN=SCYL2 PE=1 SV=1	191	26	26	103.6	38	929	8.22	19	19	21	13	13	17	18	15	17	12	12	16
High	Q00839	Heterogeneous nuclear ribonucleoprotein U OS=Homo sapiens OX=9606 GN=HNRNPU PE=1 SV=6	191	26	26	90.5	37	825	6	22	25	33	18	17	18	18	19	21	15	15	14
High	O15027	Protein transport protein Sec16A OS=Homo sapiens OX=9606 GN=SEC16A PE=1 SV=4	190	45	45	251.7	31	2357	5.8	20	22	23	11	11	13	18	21	22	11	11	13
High	P08779	SWISS-PROT:P08779 Tax_Id=9606 Gene_Symbol=KRT16 Keratin, type I cytoskeletal 16	188	15	3	51.2	30	473	5.05	30	34	39	11	16	12	9	8	10	7	8	7
High	P52272	Heterogeneous nuclear ribonucleoprotein M OS=Homo sapiens OX=9606 GN=HNRNPM PE=1 SV=3	184	31	31	77.5	48	730	8.7	21	20	18	17	13	17	21	20	17	16	13	15
High	P67936	Tropomyosin alpha-4 chain OS=Homo sapiens OX=9606 GN=TPM4 PE=1 SV=3	183	20	8	28.5	58	248	4.69	24	23	27	14	22	12	12	12	15	11	14	9
High	Q14764	Major vault protein OS=Homo sapiens OX=9606 GN=MVP PE=1 SV=4	180	30	30	99.3	43	893	5.48	13	15	15	17	20	17	13	14	15	16	18	15
High	P09327	Villin-1 OS=Homo sapiens OX=9606 GN=VIL1 PE=1 SV=4	180	29	29	92.6	43	827	6.39	23	26	24	13	17	11	20	22	21	13	14	11
High	P62979	Ubiquitin-40S ribosomal protein S27a OS=Homo sapiens OX=9606 GN=RPS27A PE=1 SV=2	180	7	7	18	51	156	9.64	18	16	22	20	23	19	7	7	7	6	7	7
High	P51659	Peroxisomal multifunctional enzyme type 2 OS=Homo sapiens OX=9606 GN=HSD17B4 PE=1 SV=3	180	24	24	79.6	47	736	8.84	21	24	26	11	13	12	18	19	20	11	12	11
High	Q15291	Retinoblastoma-binding protein 5 OS=Homo sapiens OX=9606 GN=RBBP5 PE=1 SV=2	180	23	23	59.1	51	538	5.1			1	24	25	23			1	16	17	16
High	P07900	Heat shock protein HSP 90-alpha OS=Homo sapiens OX=9606 GN=HSP90AA1 PE=1 SV=5	177	26	14	84.6	44	732	5.02	18	21	21	19	20	18	17	16	16	15	17	14
High	P14625	Endoplasmin OS=Homo sapiens OX=9606 GN=HSP90B1 PE=1 SV=1	176	28	27	92.4	39	803	4.84	22	25	24	13	22	7	17	15	23	13	16	7
High	P46940	Ras GTPase-activating-like protein IQGAP1 OS=Homo sapiens OX=9606 GN=IQGAP1 PE=1 SV=1	176	43	41	189.1	33	1657	6.48	22	21	26	5	11	10	22	19	24	5	10	10
High	Q12906	Interleukin enhancer-binding factor 3 OS=Homo sapiens OX=9606 GN=ILF3 PE=1 SV=3	175	22	20	95.3	32	894	8.76	15	17	16	18	21	17	13	15	14	14	16	14
High	P47756	F-actin-capping protein subunit beta OS=Homo sapiens OX=9606 GN=CAPZB PE=1 SV=4	175	13	13	31.3	43	277	5.59	16	18	23	15	18	12	9	10	12	9	8	7
High	P0CG39	POTE ankyrin domain family member J OS=Homo sapiens OX=9606 GN=POTEJ PE=3 SV=1	174	6	3	117.3	8	1038	5.97	32	32	17	8	23	9	5	4	4	3	3	3
High	P22314	Ubiquitin-like modifier-activating enzyme 1 OS=Homo sapiens OX=9606 GN=UBA1 PE=1 SV=3	174	31	31	117.8	37	1058	5.76	16	14	21	16	18	16	13	13	18	15	16	15
High	P11940	Polyadenylate-binding protein 1 OS=Homo sapiens OX=9606 GN=PABPC1 PE=1 SV=2	174	22	16	70.6	44	636	9.5	16	17	16	16	20	18	14	14	15	14	17	15
High	Q8IZD2	Inactive histone-lysine N-methyltransferase 2E OS=Homo sapiens OX=9606 GN=KMT2E PE=1 SV=1	173	39	38	204.8	28	1858	7.61				25	29	27				22	26	23
High	P53621	Coatomer subunit alpha OS=Homo sapiens OX=9606 GN=COPA PE=1 SV=2	173	36	36	138.3	35	1224	7.66	21	21	20	9	7	10	21	20	20	9	7	10
High	P41250	Glycine--tRNA ligase OS=Homo sapiens OX=9606 GN=GARS1 PE=1 SV=3	171	27	27	83.1	45	739	7.03	29	28	28	11	13	11	21	19	23	11	12	10
High	P25205	DNA replication licensing factor MCM3 OS=Homo sapiens OX=9606 GN=MCM3 PE=1 SV=3	170	30	30	90.9	46	808	5.77	15	12	14	20	22	19	14	11	13	16	19	16
High	P23246	Splicing factor, proline- and glutamine-rich OS=Homo sapiens OX=9606 GN=SFPQ PE=1 SV=2	170	22	21	76.1	31	707	9.44	29	31	30	7	14	6	19	21	20	5	9	6
High	P63244	Receptor of activated protein C kinase 1 OS=Homo sapiens OX=9606 GN=RACK1 PE=1 SV=3	170	19	19	35.1	84	317	7.69	21	22	21	18	16	17	15	17	16	13	11	13
High	Q9Y230	RuvB-like 2 OS=Homo sapiens OX=9606 GN=RUVBL2 PE=1 SV=3	168	19	19	51.1	46	463	5.64	19	20	21	17	15	15	16	17	18	16	14	14
High	O43143	ATP-dependent RNA helicase DHX15 OS=Homo sapiens OX=9606 GN=DHX15 PE=1 SV=2	168	22	21	90.9	30	795	7.46	13	10	15	18	19	20	12	10	13	14	14	16
High	P32119	Peroxiredoxin-2 OS=Homo sapiens OX=9606 GN=PRDX2 PE=1 SV=5	168	11	10	21.9	58	198	5.97	21	21	22	12	15	13	11	11	10	7	8	6
High	Q9UBL3	Set1/Ash2 histone methyltransferase complex subunit ASH2 OS=Homo sapiens OX=9606 GN=ASH2L PE=1 SV=1	167	22	22	68.7	42	628	5.69		1		19	21	17		1		16	16	15
High	P11413	Glucose-6-phosphate 1-dehydrogenase OS=Homo sapiens OX=9606 GN=G6PD PE=1 SV=4	166	25	25	59.2	55	515	6.84	21	15	19	14	14	16	20	15	15	13	13	15
High	P06753	Tropomyosin alpha-3 chain OS=Homo sapiens OX=9606 GN=TPM3 PE=1 SV=2	166	16	5	32.9	37	285	4.72	19	18	21	17	20	17	9	8	10	10	11	9
High	Q9UDY2	Tight junction protein ZO-2 OS=Homo sapiens OX=9606 GN=TJP2 PE=1 SV=2	165	32	32	133.9	37	1190	7.4	14	16	18	10	16	11	14	16	17	10	15	11

High	P54652	Heat shock-related 70 kDa protein 2 OS=Homo sapiens OX=9606 GN=HSPA2 PE=1 SV=1	164	12	1	70	18	639	5.74	14	13	16	18	26	15	9	8	10	8	10	7
High	Q9C0C2	182 kDa tankyrase-1-binding protein OS=Homo sapiens OX=9606 GN=TNKS1BP1 PE=1 SV=4	164	37	37	181.7	33	1729	4.86	10	11	13	15	13	13	10	11	13	15	13	13
High	P06396	Gelsolin OS=Homo sapiens OX=9606 GN=GSN PE=1 SV=1	162	23	10	85.6	39	782	6.28	19	21	25	4	12	8	15	16	18	4	9	8
High	P14923	Junction plakoglobin OS=Homo sapiens OX=9606 GN=JUP PE=1 SV=3	162	23	19	81.7	36	745	6.14	21	15	21	9	16	9	16	14	17	9	15	9
High	Q9P258	Protein RCC2 OS=Homo sapiens OX=9606 GN=RCC2 PE=1 SV=2	162	17	17	56	37	522	8.78	16	15	18	16	17	16	13	13	12	11	12	13
High	P11216	Glycogen phosphorylase, brain form OS=Homo sapiens OX=9606 GN=PYGB PE=1 SV=5	161	32	32	96.6	42	843	6.86	29	29	25	8	8	9	27	28	23	8	8	9
High	O95678	SWISS-PROT:O95678 Tax_Id=9606 Gene_Symbol=KRT75 Keratin, type II cytoskeletal 75	161	10	1	59.5	13	551	7.74	22	28	45	9	11	10	7	7	10	4	4	4
High	P17987	T-complex protein 1 subunit alpha OS=Homo sapiens OX=9606 GN=TCP1 PE=1 SV=1	161	23	23	60.3	51	556	6.11	22	22	25	11	11	10	20	19	22	11	11	10
High	P23526	Adenosylhomocysteinase OS=Homo sapiens OX=9606 GN=AHCY PE=1 SV=4	161	20	19	47.7	49	432	6.34	22	26	13	16	11	15	16	18	11	14	10	13
High	P05023	Sodium/potassium-transporting ATPase subunit alpha-1 OS=Homo sapiens OX=9606 GN=ATP1A1 PE=1 SV=1	160	28	28	112.8	33	1023	5.49	17	18	18	13	15	14	16	18	15	11	13	13
High	Q7Z3B3	KAT8 regulatory NSL complex subunit 1 OS=Homo sapiens OX=9606 GN=KANSL1 PE=1 SV=2	158	31	31	121	42	1105	8.81				23	28	23				19	23	19
High	P14868	Aspartate--tRNA ligase, cytoplasmic OS=Homo sapiens OX=9606 GN=DARS1 PE=1 SV=2	158	27	27	57.1	60	501	6.55	16	14	18	16	15	13	16	13	17	16	15	13
High	P11387	DNA topoisomerase 1 OS=Homo sapiens OX=9606 GN=TOP1 PE=1 SV=2	158	25	25	90.7	32	765	9.31	13	14	14	16	18	16	13	13	13	15	16	14
High	Q01813	ATP-dependent 6-phosphofructokinase, platelet type OS=Homo sapiens OX=9606 GN=PFKP PE=1 SV=2	156	26	22	85.5	41	784	7.55	24	24	22	8	11	8	22	21	19	8	10	8
High	O00571	ATP-dependent RNA helicase DDX3X OS=Homo sapiens OX=9606 GN=DDX3X PE=1 SV=3	155	26	25	73.2	45	662	7.18	20	22	17	13	16	14	17	17	14	12	14	12
High	Q15365	Poly(rC)-binding protein 1 OS=Homo sapiens OX=9606 GN=PCBP1 PE=1 SV=2	154	13	9	37.5	61	356	7.09	13	13	15	17	17	17	11	11	10	9	9	9
High	P30740	Leukocyte elastase inhibitor OS=Homo sapiens OX=9606 GN=SERPINB1 PE=1 SV=1	154	19	18	42.7	53	379	6.28	16	15	19	15	14	12	16	15	14	11	7	11
High	P06748	Nucleophosmin OS=Homo sapiens OX=9606 GN=NPM1 PE=1 SV=2	153	11	11	32.6	52	294	4.78	17	13	15	5	14	7	8	7	7	3	7	5
High	P62701	40S ribosomal protein S4, X isoform OS=Homo sapiens OX=9606 GN=RPS4X PE=1 SV=2	152	16	16	29.6	51	263	10.15	17	15	15	15	17	16	13	12	13	10	13	9
High	O14639	Actin-binding LIM protein 1 OS=Homo sapiens OX=9606 GN=ABLIM1 PE=1 SV=3	152	25	25	87.6	38	778	8.59	21	25	23	8	9	6	18	18	17	8	7	6
High	Q03164	Histone-lysine N-methyltransferase 2A OS=Homo sapiens OX=9606 GN=KMT2A PE=1 SV=5	151	45	41	431.5	16	3969	9.09				22	30	24				22	27	24
High	Q5QNW6	Histone H2B type 2-F OS=Homo sapiens OX=9606 GN=H2BC18 PE=1 SV=3	150	5	2	13.9	36	126	10.32	22	17	26	12	11	11	4	4	5	5	5	5
High	Q14247	Src substrate cortactin OS=Homo sapiens OX=9606 GN=CTTN PE=1 SV=2	150	20	20	61.5	41	550	5.4	17	14	15	12	15	15	15	13	12	11	15	14
High	P09874	Poly [ADP-ribose] polymerase 1 OS=Homo sapiens OX=9606 GN=PARP1 PE=1 SV=4	147	29	29	113	35	1014	8.88	17	16	20	13	14	13	16	16	17	13	14	13
High	Q9UJ72	Annexin A10 OS=Homo sapiens OX=9606 GN=ANXA10 PE=1 SV=3	146	17	17	37.3	66	324	5.33	17	17	14	8	11	8	14	14	12	7	8	7
High	Q7L576	Cytoplasmic FMR1-interacting protein 1 OS=Homo sapiens OX=9606 GN=CYFIP1 PE=1 SV=1	146	29	29	145.1	27	1253	6.9	20	22	21	9	8	10	17	19	16	9	8	10
High	P52907	F-actin-capping protein subunit alpha-1 OS=Homo sapiens OX=9606 GN=CAPZA1 PE=1 SV=3	146	12	10	32.9	65	286	5.69	15	12	16	5	7	7	9	6	10	4	6	5
High	P85037	Forkhead box protein K1 OS=Homo sapiens OX=9606 GN=FOXK1 PE=1 SV=1	145	21	19	75.4	31	733	9.32	5	4	3	19	19	19	5	4	3	15	17	14
High	P55265	Double-stranded RNA-specific adenosine deaminase OS=Homo sapiens OX=9606 GN=ADAR PE=1 SV=4	145	29	29	136	29	1226	8.65	11	12	10	16	17	18	11	12	10	16	15	17
High	P41252	Isoleucine--tRNA ligase, cytoplasmic OS=Homo sapiens OX=9606 GN=IARS1 PE=1 SV=2	145	30	30	144.4	31	1262	6.15	17	14	17	9	7	9	17	14	16	9	7	9
High	P10599	Thioredoxin OS=Homo sapiens OX=9606 GN=TXN PE=1 SV=3	144	5	5	11.7	50	105	4.92	12	16	17	10	23	9	4	5	5	4	4	3
High	P37802	Transgelin-2 OS=Homo sapiens OX=9606 GN=TAGLN2 PE=1 SV=3	144	13	13	22.4	74	199	8.25	16	12	12	16	15	12	12	10	9	9	9	8
High	P56470	Galectin-4 OS=Homo sapiens OX=9606 GN=LGALS4 PE=1 SV=1	144	11	11	35.9	35	323	9.16	17	17	20	6	5	8	9	9	10	5	3	5
High	P49915	GMP synthase [glutamine-hydrolyzing] OS=Homo sapiens OX=9606 GN=GMPS PE=1 SV=1	143	26	26	76.7	48	693	6.87	20	18	17	11	12	8	17	16	14	10	10	8
High	P33992	DNA replication licensing factor MCM5 OS=Homo sapiens OX=9606 GN=MCM5 PE=1 SV=5	142	28	28	82.2	44	734	8.37	14	9	15	9	18	10	14	8	15	9	16	10
High	P06576	ATP synthase subunit beta, mitochondrial OS=Homo sapiens OX=9606 GN=ATP5F1B PE=1 SV=3	141	22	22	56.5	62	529	5.4	22	23	25	12	11	13	17	18	20	10	9	11
High	P63104	14-3-3 protein zeta/delta OS=Homo sapiens OX=9606 GN=YWHAZ PE=1 SV=1	140	14	11	27.7	62	245	4.79	15	15	16	7	15	12	11	11	10	7	9	7
High	Q16778	Histone H2B type 2-E OS=Homo sapiens OX=9606 GN=H2BC21 PE=1 SV=3	140	5	2	13.9	36	126	10.32	20	16	26	11	10	10	4	4	5	5	5	5
High	Q6IFX2	SWISS-PROT:Q6IFX2 Tax_Id=10090 Gene_Symbol=Krt42 Keratin, type I cytoskeletal 42	140	11	1	50.1	18	452	5.16	27	27	32	8	14	7	9	9	11	4	6	4
High	P53618	Coatomer subunit beta OS=Homo sapiens OX=9606 GN=COPB1 PE=1 SV=3	138	28	28	107.1	41	953	6.05	26	27	25	6	7	2	22	22	21	5	6	2
High	P61247	40S ribosomal protein S3a OS=Homo sapiens OX=9606 GN=RPS3A PE=1 SV=2	137	16	16	29.9	53	264	9.73	11	9	11	13	15	15	9	7	10	12	12	13
High	Q92560	Ubiquitin carboxyl-terminal hydrolase BAP1 OS=Homo sapiens OX=9606 GN=BAP1 PE=1 SV=2	137	25	25	80.3	48	729	6.84				15	23	17				13	15	13
High	P62258	14-3-3 protein epsilon OS=Homo sapiens OX=9606 GN=YWHAE PE=1 SV=1	137	16	13	29.2	59	255	4.74	14	16	18	10	12	13	12	12	12	10	10	11
High	O14974	Protein phosphatase 1 regulatory subunit 12A OS=Homo sapiens OX=9606 GN=PPP1R12A PE=1 SV=1	136	22	22	115.2	26	1030	5.4	14	11	19	12	11	11	12	10	16	10	11	11
High	P27348	14-3-3 protein theta OS=Homo sapiens OX=9606 GN=YWHAQ PE=1 SV=1	136	12	8	27.7	53	245	4.78	14	15	16	11	14	14	8	9	8	8	7	8
High	P39023	60S ribosomal protein L3 OS=Homo sapiens OX=9606 GN=RPL3 PE=1 SV=2	135	15	15	46.1	40	403	10.18	13	12	15	7	10	10	10	10	11	7	8	9
High	P12814	Alpha-actinin-1 OS=Homo sapiens OX=9606 GN=ACTN1 PE=1 SV=2	135	23	10	103	32	892	5.41	22	29	24	5	7	6	16	20	17	5	6	6
High	P04843	Dolichyl-diphosphooligosaccharide--protein glycosyltransferase subunit 1 OS=Homo sapiens OX=9606 GN=RPN1 PE=1 SV=1	135	25	25	68.5	52	607	6.38	17	22	21	8	9	8	17	21	18	8	8	8
High	Q92597	Protein NDRG1 OS=Homo sapiens OX=9606 GN=NDRG1 PE=1 SV=1	135	11	11	42.8	54	394	5.82	16	20	13	8	11	7	11	11	8	5	6	5
High	O94973	AP-2 complex subunit alpha-2 OS=Homo sapiens OX=9606 GN=AP2A2 PE=1 SV=2	134	28	18	103.9	40	939	6.96	16	17	16	5	7	5	15	16	16	5	7	5
High	P25705	ATP synthase subunit alpha, mitochondrial OS=Homo sapiens OX=9606 GN=ATP5F1A PE=1 SV=1	134	19	19	59.7	46	553	9.13	22	21	22	7	10	11	17	17	17	7	10	10
High	P05141	ADP/ATP translocase 2 OS=Homo sapiens OX=9606 GN=SLC25A5 PE=1 SV=7	133	16	6	32.8	51	298	9.69	16	15	15	6	11	8	12	13	10	6	10	6
High	P35080	Profilin-2 OS=Homo sapiens OX=9606 GN=PFN2 PE=1 SV=3	133	5	5	15	36	140	6.99	10	11	15	10	15	10	4	4	4	3	5	4
High	O95789	Zinc finger MYM-type protein 6 OS=Homo sapiens OX=9606 GN=ZMYM6 PE=1 SV=2	133	40	40	148	36	1325	8.22												
High	P46013	Proliferation marker protein Ki-67 OS=Homo sapiens OX=9606 GN=MKI67 PE=1 SV=2	132	37	37	358.5	17	3256	9.45	8	8	14	12	13	14	8	8	13	12	13	14
High	O43390	Heterogeneous nuclear ribonucleoprotein R OS=Homo sapiens OX=9606 GN=HNRNPR PE=1 SV=1	131	21	15	70.9	39	633	8.13	13	14	18	8	12	12	13	14	16	8	12	12
High	P38159	RNA-binding motif protein, X chromosome OS=Homo sapiens OX=9606 GN=RBMX PE=1 SV=3	130	16	4	42.3	41	391	10.05	13	16	21	10	12	13	11	13	15	10	10	11
High	P34932	Heat shock 70 kDa protein 4 OS=Homo sapiens OX=9606 GN=HSPA4 PE=1 SV=4	130	26	25	94.3	44	840	5.19	10	13	18	8	7	7	10	12	18	7	7	7
High	P67809	Y-box-binding protein 1 OS=Homo sapiens OX=9606 GN=YBX1 PE=1 SV=3	130	9	6	35.9	48	324	9.88	12	9	14	14	13	14	6	6	7	9	7	9
High	Q92945	Far upstream element-binding protein 2 OS=Homo sapiens OX=9606 GN=KHSRP PE=1 SV=4	130	20	18	73.1	40	711	7.3	11	11	11	12	16	13	11	10	11	12	14	13
High	P43243	Matrin-3 OS=Homo sapiens OX=9606 GN=MATR3 PE=1 SV=2	130	25	25	94.6	38	847	6.25	15	14	16	11	10	9	13	10	14	11	10	9
High	P0DP25	Calmodulin-3 OS=Homo sapiens OX=9606 GN=CALM3 PE=1 SV=1	130	9	9	16.8	52	149	4.22	15	17	15	7	13	6	8	8	7	7	7	6
High	P17812	CTP synthase 1 OS=Homo sapiens OX=9606 GN=CTPS1 PE=1 SV=2	130	19	18	66.6	46	591	6.46	18	13	15	12	12	12	17	12	13	11	10	11
High	P09651	Heterogeneous nuclear ribonucleoprotein A1 OS=Homo sapiens OX=9606 GN=HNRNPA1 PE=1 SV=5	130	16	14	38.7	46	372	9.13	21	21	19	9	9	10	15	14	12	6	7	6
High	O60506	Heterogeneous nuclear ribonucleoprotein Q OS=Homo sapiens OX=9606 GN=SYNCRIP PE=1 SV=2	129	20	14	69.6	41	623	8.59	13	15	16	6	10	11	13	14	14	6	10	10
High	P06703	Protein S100-A6 OS=Homo sapiens OX=9606 GN=S100A6 PE=1 SV=1	129	3	3	10.2	19	90	5.48	13	9	16	13	13	14	2	2	2	3	2	3
High	Q8IXJ9	Polycomb group protein ASXL1 OS=Homo sapiens OX=9606 GN=ASXL1 PE=1 SV=4	128	26	25	165.3	23	1541	6.21				21	23	19				20	21	18
High	P60842	Eukaryotic initiation factor 4A-I OS=Homo sapiens OX=9606 GN=EIF4A1 PE=1 SV=1	128	18	10	46.1	54	406	5.48	17	16	20	9	13	13	13	14	17	9	11	11
High	P35221	Catenin alpha-1 OS=Homo sapiens OX=9606 GN=CTNNA1 PE=1 SV=1	127	28	28	100	40	906	6.29	21	21	17	5	6	7	21	20	17	5	6	7
High	Q03252	Lamin-B2 OS=Homo sapiens OX=9606 GN=LMNB2 PE=1 SV=4	127	27	24	69.9	40	620	5.59	23	20	26	7	8	7	16	16	20	6	6	7
High	P42224	Signal transducer and activator of transcription 1-alpha/beta OS=Homo sapiens OX=9606 GN=STAT1 PE=1 SV=2	126	23	23	87.3	36	750	6.05	17	14	16	9	17	11	16	13	15	9	14	11
High	Q15424	Scaffold attachment factor B1 OS=Homo sapiens OX=9606 GN=SAFB PE=1 SV=4	125	19	12	102.6	22	915	5.47	16	16	19	7	11	10	12	13	15	7	10	9
High	O75694	Nuclear pore complex protein Nup155 OS=Homo sapiens OX=9606 GN=NUP155 PE=1 SV=1	125	23	23	155.1	22	1391	6.16	13	12	17	8	8	6	13	12	14	7	6	6
High	Q15181	Inorganic pyrophosphatase OS=Homo sapiens OX=9606 GN=PPA1 PE=1 SV=2	125	16	15	32.6	71	289	5.86	13	14	16	7	16	10	10	12	13	5	8	7
High	P16144	Integrin beta-4 OS=Homo sapiens OX=9606 GN=ITGB4 PE=1 SV=5	125	32	32	202	24	1822	6.09	15	13	22	7	6	6	15	13	20	7	6	6
High	O60502	Protein O-GlcNAcase OS=Homo sapiens OX=9606 GN=OGA PE=1 SV=2	125	32	32	102.8	44	916	4.91	1	2		9	11	10	1	2		9	11	10
High	Q16891	MICOS complex subunit MIC60 OS=Homo sapiens OX=9606 GN=IMMT PE=1 SV=1	125	25	25	83.6	40	758	6.48	10	10	14	10	16	11	10	10	13	10	15	11
High	Q6WCQ1	Myosin phosphatase Rho-interacting protein OS=Homo sapiens OX=9606 GN=MPRIP PE=1 SV=3	124	37	37	116.5	48	1025	6.21	22	25	22	1		1	19	22	20	1		1
High	P36578	60S ribosomal protein L4 OS=Homo sapiens OX=9606 GN=RPL4 PE=1 SV=5	124	16	16	47.7	41	427	11.06	9	9	11	11	10	13	9	9	11	8	8	9
High	Q9BUJ2	Heterogeneous nuclear ribonucleoprotein U-like protein 1 OS=Homo sapiens OX=9606 GN=HNRNPUL1 PE=1 SV=2	124	20	20	95.7	28	856	6.92	18	17	20	7	6	7	17	15	17	7	6	7
High	Q9UHR4	Brain-specific angiogenesis inhibitor 1-associated protein 2-like protein 1 OS=Homo sapiens OX=9606 GN=BAIAP2L1 PE=1 SV=2	124	23	23	56.8	52	511	8.68	12	13	18	9	11	9	12	12	17	9	11	9
High	P48643	T-complex protein 1 subunit epsilon OS=Homo sapiens OX=9606 GN=CCT5 PE=1 SV=1	124	23	22	59.6	54	541	5.66	16	15	21	9	11	6	13	13	15	8	10	5
High	Q96CW1	AP-2 complex subunit mu OS=Homo sapiens OX=9606 GN=AP2M1 PE=1 SV=2	123	18	18	49.6	42	435	9.54	16	16	15	5	10	7	13	13	12	5	10	7
High	Q9P2N6	KAT8 regulatory NSL complex subunit 3 OS=Homo sapiens OX=9606 GN=KANSL3 PE=1 SV=2	123	25	25	95.9	45	904	9.39				19	22	19				17	18	16
High	Q7Z2W4	Zinc finger CCCH-type antiviral protein 1 OS=Homo sapiens OX=9606 GN=ZC3HAV1 PE=1 SV=3	122	23	23	101.4	33	902	8.4	5	4	5	19	11	19	5	4	5	17	11	17
High	Q9Y2T3	Guanine deaminase OS=Homo sapiens OX=9606 GN=GDA PE=1 SV=1	122	19	19	51	52	454	5.68	20	15	18	9	7	8	15	12	13	8	6	8
High	Q9Y265	RuvB-like 1 OS=Homo sapiens OX=9606 GN=RUVBL1 PE=1 SV=1	122	17	17	50.2	49	456	6.42	15	14	18	6	8	4	13	13	16	6	7	4
High	Q92804	TATA-binding protein-associated factor 2N OS=Homo sapiens OX=9606 GN=TAF15 PE=1 SV=1	122	9	7	61.8	22	592	8.02	14	18	29	6	16	8	9	9	8	4	7	5
High	P11586	C-1-tetrahydrofolate synthase, cytoplasmic OS=Homo sapiens OX=9606 GN=MTHFD1 PE=1 SV=4	122	24	23	101.5	30	935	7.3	15	14	13	5	16	6	13	13	13	5	15	6
High	P27816	Microtubule-associated protein 4 OS=Homo sapiens OX=9606 GN=MAP4 PE=1 SV=3	121	28	28	120.9	35	1152	5.43	5	9	7	13	13	12	5	9	6	13	13	12
High	P54136	Arginine--tRNA ligase, cytoplasmic OS=Homo sapiens OX=9606 GN=RARS1 PE=1 SV=2	121	27	27	75.3	46	660	6.68	12	13	12	11	10	11	12	11	11	10	10	10
High	Q06210	Glutamine--fructose-6-phosphate aminotransferase [isomerizing] 1 OS=Homo sapiens OX=9606 GN=GFPT1 PE=1 SV=3	121	22	19	78.8	39	699	7.11	11	12	13	7	13	11	11	12	12	7	13	10
High	Q9Y5B9	FACT complex subunit SPT16 OS=Homo sapiens OX=9606 GN=SUPT16H PE=1 SV=1	121	25	25	119.8	28	1047	5.66	16	16	13	9	17	12	16	16	13	9	15	12
High	O95994	Anterior gradient protein 2 homolog OS=Homo sapiens OX=9606 GN=AGR2 PE=1 SV=1	120	8	7	20	59	175	9	5	6	5	28	30	30	5	5	4	5	5	4
High	P50990	T-complex protein 1 subunit theta OS=Homo sapiens OX=9606 GN=CCT8 PE=1 SV=4	120	21	21	59.6	43	548	5.6	12	11	15	11	11	11	12	11	15	11	11	11
High	P50995	Annexin A11 OS=Homo sapiens OX=9606 GN=ANXA11 PE=1 SV=1	119	17	16	54.4	34	505	7.65	17	15	17	8	10	7	15	13	14	8	10	7
High	P61964	WD repeat-containing protein 5 OS=Homo sapiens OX=9606 GN=WDR5 PE=1 SV=1	119	11	11	36.6	46	334	8.27	3	1	3	14	20	16	3	1	3	8	10	8
High	Q15366	Poly(rC)-binding protein 2 OS=Homo sapiens OX=9606 GN=PCBP2 PE=1 SV=1	119	13	8	38.6	66	365	6.79	15	13	13	11	11	9	12	10	9	6	7	6
High	O00425	Insulin-like growth factor 2 mRNA-binding protein 3 OS=Homo sapiens OX=9606 GN=IGF2BP3 PE=1 SV=2	119	19	17	63.7	42	579	8.87	11	9	10	8	11	13	11	9	10	8	10	12
High	P49368	T-complex protein 1 subunit gamma OS=Homo sapiens OX=9606 GN=CCT3 PE=1 SV=4	119	22	22	60.5	53	545	6.49	12	13	15	10	8	11	10	12	14	10	8	10
High	P08590	Myosin light chain 3 OS=Homo sapiens OX=9606 GN=MYL3 PE=1 SV=3	118	10	7	21.9	51	195	5.1	13	13	16	10	10	14	7	7	6	8	5	8
High	P35658	Nuclear pore complex protein Nup214 OS=Homo sapiens OX=9606 GN=NUP214 PE=1 SV=2	117	29	29	213.5	22	2090	7.47	3	3	1	10	10	11	3	3	1	10	10	11
High	P31946	14-3-3 protein beta/alpha OS=Homo sapiens OX=9606 GN=YWHAB PE=1 SV=3	116	10	5	28.1	48	246	4.83	13	12	16	11	11	12	8	8	8	8	7	8
High	Q9NSB2	SWISS-PROT:Q9NSB2 Keratin, type II cuticular Hb4 (Hair keratin, type II Hb4) - Homo sapiens (Human).	116	4	1	64.9	4	600	7.74	16	22	32	7	9	8	4	4	3	2	2	2
High	O75533	Splicing factor 3B subunit 1 OS=Homo sapiens OX=9606 GN=SF3B1 PE=1 SV=3	116	26	26	145.7	28	1304	7.09	13	13	10	8	13	9	13	13	10	8	13	9
High	Q9Y2W1	Thyroid hormone receptor-associated protein 3 OS=Homo sapiens OX=9606 GN=THRAP3 PE=1 SV=2	116	22	21	108.6	28	955	10.15	11	11	10	10	16	12	11	11	9	10	15	12
High	P51991	Heterogeneous nuclear ribonucleoprotein A3 OS=Homo sapiens OX=9606 GN=HNRNPA3 PE=1 SV=2	115	17	16	39.6	39	378	9.01	17	14	19	5	12	6	13	13	12	4	9	4
High	P33176	Kinesin-1 heavy chain OS=Homo sapiens OX=9606 GN=KIF5B PE=1 SV=1	115	25	25	109.6	30	963	6.51	1	4	6	18	16	17	1	4	6	16	16	16
High	Q6KB66-1	SWISS-PROT:Q6KB66-1 Tax_Id=9606 Gene_Symbol=KRT80 Isoform 1 of Keratin, type II cytoskeletal 80	115	22	21	50.5	49	452	5.67	23	15	16	11	10	9	16	14	15	11	10	9
High	P31943	Heterogeneous nuclear ribonucleoprotein H OS=Homo sapiens OX=9606 GN=HNRNPH1 PE=1 SV=4	115	10	4	49.2	32	449	6.3	15	12	13	7	12	7	8	8	8	6	8	6
High	O75955	Flotillin-1 OS=Homo sapiens OX=9606 GN=FLOT1 PE=1 SV=3	115	23	23	47.3	68	427	7.49	13	11	12	5	2	3	12	9	12	5	2	3
High	O15020	Spectrin beta chain, non-erythrocytic 2 OS=Homo sapiens OX=9606 GN=SPTBN2 PE=1 SV=3	115	29	19	271.2	15	2390	6.11	19	20	20	4	2	2	15	16	19	4	2	2
High	P27635	60S ribosomal protein L10 OS=Homo sapiens OX=9606 GN=RPL10 PE=1 SV=5	115	10	10	24.6	43	214	10.08	9	11	11	11	11	8	7	8	8	6	6	5
High	P12236	ADP/ATP translocase 3 OS=Homo sapiens OX=9606 GN=SLC25A6 PE=1 SV=4	114	16	6	32.8	57	298	9.74	14	13	15	5	9	5	11	12	11	5	8	4
High	Q92499	ATP-dependent RNA helicase DDX1 OS=Homo sapiens OX=9606 GN=DDX1 PE=1 SV=2	113	22	22	82.4	34	740	7.23	14	12	15	7	13	7	14	12	14	7	13	7
High	P53992	Protein transport protein Sec24C OS=Homo sapiens OX=9606 GN=SEC24C PE=1 SV=3	113	23	22	118.2	27	1094	7.06	8	10	11	10	14	11	8	10	11	9	11	11
High	Q8N1F7	Nuclear pore complex protein Nup93 OS=Homo sapiens OX=9606 GN=NUP93 PE=1 SV=2	113	32	32	93.4	44	819	5.72	23	20	20	2	1	2	23	19	20	2	1	1
High	Q9NSD9	Phenylalanine--tRNA ligase beta subunit OS=Homo sapiens OX=9606 GN=FARSB PE=1 SV=3	112	20	20	66.1	33	589	6.84	13	14	14	9	12	9	13	13	12	9	12	9
High	P62241	40S ribosomal protein S8 OS=Homo sapiens OX=9606 GN=RPS8 PE=1 SV=2	112	10	10	24.2	52	208	10.32	10	9	10	14	11	13	9	9	9	10	9	10
High	P26599	Polypyrimidine tract-binding protein 1 OS=Homo sapiens OX=9606 GN=PTBP1 PE=1 SV=1	111	14	11	57.2	42	531	9.17	10	10	11	9	10	12	9	9	10	8	6	8
High	P52597	Heterogeneous nuclear ribonucleoprotein F OS=Homo sapiens OX=9606 GN=HNRNPF PE=1 SV=3	111	13	11	45.6	49	415	5.58	13	14	12	5	10	8	9	10	7	5	6	6
High	P78371	T-complex protein 1 subunit beta OS=Homo sapiens OX=9606 GN=CCT2 PE=1 SV=4	111	18	18	57.5	48	535	6.46	13	12	13	6	6	8	13	12	11	6	6	8
High	P04083	Annexin A1 OS=Homo sapiens OX=9606 GN=ANXA1 PE=1 SV=2	111	18	18	38.7	60	346	7.02	10	9	13	9	13	9	8	9	11	9	11	9
High	P46109	Crk-like protein OS=Homo sapiens OX=9606 GN=CRKL PE=1 SV=1	110	13	13	33.8	65	303	6.74	9	9	10	7	10	6	9	9	9	6	7	5
High	Q99714	3-hydroxyacyl-CoA dehydrogenase type-2 OS=Homo sapiens OX=9606 GN=HSD17B10 PE=1 SV=3	110	14	14	26.9	74	261	7.78	10	11	7	8	9	10	9	9	6	6	5	8
High	Q07955	Serine/arginine-rich splicing factor 1 OS=Homo sapiens OX=9606 GN=SRSF1 PE=1 SV=2	110	11	11	27.7	38	248	10.36	11	9	11	11	14	10	8	7	8	8	9	8
High	O60701	UDP-glucose 6-dehydrogenase OS=Homo sapiens OX=9606 GN=UGDH PE=1 SV=1	110	19	19	55	54	494	7.12	17	14	18	7	8	9	16	13	16	7	8	8
High	Q99497	Parkinson disease protein 7 OS=Homo sapiens OX=9606 GN=PARK7 PE=1 SV=2	110	10	10	19.9	58	189	6.79	12	14	11	7	14	8	8	8	7	5	7	5
High	Q12929	Epidermal growth factor receptor kinase substrate 8 OS=Homo sapiens OX=9606 GN=EPS8 PE=1 SV=1	109	24	24	91.8	42	822	7.5	13	14	15	1	2	1	13	13	14	1	2	1
High	P47755	F-actin-capping protein subunit alpha-2 OS=Homo sapiens OX=9606 GN=CAPZA2 PE=1 SV=3	109	11	9	32.9	65	286	5.85	11	12	9	3	6	4	8	7	8	3	5	3
High	Q93009	Ubiquitin carboxyl-terminal hydrolase 7 OS=Homo sapiens OX=9606 GN=USP7 PE=1 SV=2	109	22	22	128.2	24	1102	5.55	9	8	5	12	17	12	9	7	5	12	16	12
High	Q9H6S3	Epidermal growth factor receptor kinase substrate 8-like protein 2 OS=Homo sapiens OX=9606 GN=EPS8L2 PE=1 SV=2	108	21	21	80.6	37	715	6.84	11	11	11	7	13	10	11	11	11	7	13	10
High	Q12931	Heat shock protein 75 kDa, mitochondrial OS=Homo sapiens OX=9606 GN=TRAP1 PE=1 SV=3	107	19	18	80.1	37	704	8.21	7	9	14	10	7	7	7	8	10	9	6	6
High	O60437	Periplakin OS=Homo sapiens OX=9606 GN=PPL PE=1 SV=4	107	36	36	204.6	24	1756	5.6	17	22	28	6	5	3	17	21	26	6	5	3
High	O00443	Phosphatidylinositol 4-phosphate 3-kinase C2 domain-containing subunit alpha OS=Homo sapiens OX=9606 GN=PIK3C2A PE=1 SV	106	35	35	190.6	25	1686	8.02	8	12	12	3	2	3	8	12	12	3	2	3
High	P55060	Exportin-2 OS=Homo sapiens OX=9606 GN=CSE1L PE=1 SV=3	106	17	17	110.3	20	971	5.77	10	12	15	9	10	10	9	10	14	8	10	10
High	Q96E39	RNA binding motif protein, X-linked-like-1 OS=Homo sapiens OX=9606 GN=RBMXL1 PE=1 SV=1	106	13	1	42.1	34	390	9.89	10	12	19	7	10	11	8	10	13	7	9	9
High	O60716	Catenin delta-1 OS=Homo sapiens OX=9606 GN=CTNND1 PE=1 SV=1	105	21	21	108.1	31	968	6.23	13	16	15	7	3	6	13	16	14	7	3	6
High	P62280	40S ribosomal protein S11 OS=Homo sapiens OX=9606 GN=RPS11 PE=1 SV=3	105	11	11	18.4	52	158	10.3	11	12	13	8	11	9	9	10	11	8	7	8
High	Q13501	Sequestosome-1 OS=Homo sapiens OX=9606 GN=SQSTM1 PE=1 SV=1	105	12	12	47.7	58	440	5.22	4	5	4	18	26	22	4	5	4	10	11	10
High	Q9UGI8	Testin OS=Homo sapiens OX=9606 GN=TES PE=1 SV=1	104	15	15	48	41	421	7.68	11	12	13	8	11	8	10	11	10	8	10	8
High	P35237	Serpin B6 OS=Homo sapiens OX=9606 GN=SERPINB6 PE=1 SV=3	104	13	12	42.6	47	376	5.27	8	9	16	7	10	6	7	8	13	6	7	6
High	Q9Y608	Leucine-rich repeat flightless-interacting protein 2 OS=Homo sapiens OX=9606 GN=LRRFIP2 PE=1 SV=1	104	15	14	82.1	25	721	6.95	13	13	19	6	7	9	10	10	15	6	6	9
High	P61981	14-3-3 protein gamma OS=Homo sapiens OX=9606 GN=YWHAG PE=1 SV=2	104	9	4	28.3	38	247	4.89	10	11	15	10	11	13	7	8	7	6	6	7
High	P41240	Tyrosine-protein kinase CSK OS=Homo sapiens OX=9606 GN=CSK PE=1 SV=1	104	16	15	50.7	41	450	7.06	9	11	7	10	12	11	9	10	7	10	12	10
High	P10515	Dihydrolipoyllysine-residue acetyltransferase component of pyruvate dehydrogenase complex, mitochondrial OS=Homo sapiens (	104	15	15	69	28	647	7.84	4	4	7	9	10	9	4	4	7	6	8	7
High	Q92621	Nuclear pore complex protein Nup205 OS=Homo sapiens OX=9606 GN=NUP205 PE=1 SV=3	103	34	34	227.8	19	2012	6.19	23	25	27	2	2	1	23	25	25	2	2	1
High	Q10567	AP-1 complex subunit beta-1 OS=Homo sapiens OX=9606 GN=AP1B1 PE=1 SV=3	103	22	7	104.5	25	949	5.06	7	10	14	6	5	8	7	10	13	6	4	8
High	Q7Z794	Keratin, type II cytoskeletal 1b OS=Homo sapiens OX=9606 GN=KRT77 PE=1 SV=3	102	5	1	61.9	6	578	5.99	13	18	31	6	7	8	4	4	4	3	2	3
High	Q9ULA0	Aspartyl aminopeptidase OS=Homo sapiens OX=9606 GN=DNPEP PE=1 SV=2	102	14	14	53.4	33	485	7.58	10	11	10	9	11	9	9	9	8	7	8	8
High	P30101	Protein disulfide-isomerase A3 OS=Homo sapiens OX=9606 GN=PDIA3 PE=1 SV=4	102	20	20	56.7	48	505	6.35	10	12	16	9	8	9	9	12	14	9	8	9
High	Q02878	60S ribosomal protein L6 OS=Homo sapiens OX=9606 GN=RPL6 PE=1 SV=3	101	12	12	32.7	35	288	10.58	9	9	8	11	12	11	9	9	7	10	10	10
High	P13797	Plastin-3 OS=Homo sapiens OX=9606 GN=PLS3 PE=1 SV=4	101	24	16	70.8	47	630	5.6	8	12	10	11	11	13	7	11	10	11	11	13
High	Q69YQ0	Cytospin-A OS=Homo sapiens OX=9606 GN=SPECC1L PE=1 SV=3	101	27	27	124.5	29	1117	5.76	17	22	19		1		17	21	18		1	
High	P61106	Ras-related protein Rab-14 OS=Homo sapiens OX=9606 GN=RAB14 PE=1 SV=4	100	14	13	23.9	78	215	6.21	10	10	9	7	12	7	7	7	7	7	9	7
High	P07744	SWISS-PROT:P07744 Tax_Id=10090 Gene_Symbol=Krt4 Keratin, type II cytoskeletal 4	100	5	1	56.2	6	525	8.15	13	18	30	5	6	7	3	3	4	2	3	2
High	Q13557	Calcium/calmodulin-dependent protein kinase type II subunit delta OS=Homo sapiens OX=9606 GN=CAMK2D PE=1 SV=3	100	14	11	56.3	32	499	7.25	10	11	11	8	9	7	10	11	10	8	9	7
High	Q8WUM4	Programmed cell death 6-interacting protein OS=Homo sapiens OX=9606 GN=PDCD6IP PE=1 SV=1	100	25	25	96	32	868	6.52	9	11	13	5	3	5	9	11	13	5	3	5
High	P18124	60S ribosomal protein L7 OS=Homo sapiens OX=9606 GN=RPL7 PE=1 SV=1	100	17	17	29.2	52	248	10.65	6	7	11	9	10	10	5	6	9	9	9	8
High	P62249	40S ribosomal protein S16 OS=Homo sapiens OX=9606 GN=RPS16 PE=1 SV=2	100	7	7	16.4	44	146	10.21	9	10	10	6	9	7	5	6	7	6	7	5
High	Q15393	Splicing factor 3B subunit 3 OS=Homo sapiens OX=9606 GN=SF3B3 PE=1 SV=4	99	19	19	135.5	18	1217	5.26	2	5	9	14	16	14	2	5	8	13	14	13
High	P04899	Guanine nucleotide-binding protein G(i) subunit alpha-2 OS=Homo sapiens OX=9606 GN=GNAI2 PE=1 SV=3	99	15	9	40.4	59	355	5.54	11	9	11	7	8	8	9	7	10	6	7	7
High	Q5SRE5	Nucleoporin NUP188 OS=Homo sapiens OX=9606 GN=NUP188 PE=1 SV=1	99	27	27	195.9	19	1749	6.73	5	7	6	5	11	6	5	7	6	5	11	5
High	P46087	Probable 28S rRNA (cytosine(4447)-C(5))-methyltransferase OS=Homo sapiens OX=9606 GN=NOP2 PE=1 SV=2	99	19	19	89.2	29	812	9.23	6	3	5	13	16	14	6	3	4	13	16	14
High	P62826	GTP-binding nuclear protein Ran OS=Homo sapiens OX=9606 GN=RAN PE=1 SV=3	99	9	9	24.4	35	216	7.49	7	7	6	5	5	5	6	6	5	5	5	5
High	P55795	Heterogeneous nuclear ribonucleoprotein H2 OS=Homo sapiens OX=9606 GN=HNRNPH2 PE=1 SV=1	99	11	6	49.2	35	449	6.3	13	11	10	7	9	6	8	7	8	6	7	5
High	P45974	Ubiquitin carboxyl-terminal hydrolase 5 OS=Homo sapiens OX=9606 GN=USP5 PE=1 SV=2	98	16	16	95.7	26	858	5.03	10	9	13	10	9	10	10	9	10	10	7	10
High	P50991	T-complex protein 1 subunit delta OS=Homo sapiens OX=9606 GN=CCT4 PE=1 SV=4	98	18	17	57.9	42	539	7.83	7	10	13	8	7	10	7	9	11	8	7	10
High	Q13200	26S proteasome non-ATPase regulatory subunit 2 OS=Homo sapiens OX=9606 GN=PSMD2 PE=1 SV=3	98	22	22	100.1	28	908	5.2	3	2	5	15	16	16	3	2	5	15	15	16

High	Q9UHX1	Poly(U)-binding-splicing factor PUF60 OS=Homo sapiens OX=9606 GN=PUF60 PE=1 SV=1	98	14	14	59.8	35	559	5.29	8	7	9	9	11	10	8	7	9	8	10	10
High	O43684	Mitotic checkpoint protein BUB3 OS=Homo sapiens OX=9606 GN=BUB3 PE=1 SV=1	97	11	11	37.1	35	328	6.84	5	5	4	15	18	13	5	5	4	11	11	9
High	Q9Y3I0	RNA-splicing ligase RtcB homolog OS=Homo sapiens OX=9606 GN=RTCB PE=1 SV=1	97	19	19	55.2	45	505	7.23	12	10	15	6	9	6	12	9	13	6	9	6
High	P33993	DNA replication licensing factor MCM7 OS=Homo sapiens OX=9606 GN=MCM7 PE=1 SV=4	97	26	26	81.3	45	719	6.46	20	20	15	3	6	4	17	19	14	3	6	4
High	P27694	Replication protein A 70 kDa DNA-binding subunit OS=Homo sapiens OX=9606 GN=RPA1 PE=1 SV=2	97	25	25	68.1	44	616	7.21	20	18	22	6	6	6	19	18	21	6	6	6
High	Q13310	Polyadenylate-binding protein 4 OS=Homo sapiens OX=9606 GN=PABPC4 PE=1 SV=1	96	17	11	70.7	29	644	9.26	7	7	8	8	12	11	7	7	8	8	11	10
High	P04406	Glyceraldehyde-3-phosphate dehydrogenase OS=Homo sapiens OX=9606 GN=GAPDH PE=1 SV=3	96	11	11	36	43	335	8.46	13	14	17	5	5	5	9	10	10	5	4	5
High	P14866	Heterogeneous nuclear ribonucleoprotein L OS=Homo sapiens OX=9606 GN=HNRNPL PE=1 SV=2	96	18	17	64.1	48	589	8.22	8	9	10	5	15	8	7	9	10	5	14	8
High	P84243	Histone H3.3 OS=Homo sapiens OX=9606 GN=H3-3A PE=1 SV=2	95	3	1	15.3	17	136	11.27	9	9	17	12	7	14	2	2	3	2	3	2
High	P50402	Emerin OS=Homo sapiens OX=9606 GN=EMD PE=1 SV=1	95	13	13	29	73	254	5.5	16	17	10	5	6	5	12	13	10	4	6	4
High	P07237	Protein disulfide-isomerase OS=Homo sapiens OX=9606 GN=P4HB PE=1 SV=3	95	20	20	57.1	42	508	4.87	10	10	14	3	7	3	8	9	10	3	7	3
High	P18621	60S ribosomal protein L17 OS=Homo sapiens OX=9606 GN=RPL17 PE=1 SV=3	95	7	7	21.4	42	184	10.17	8	9	11	6	6	5	6	6	5	4	4	3
High	P00338	L-lactate dehydrogenase A chain OS=Homo sapiens OX=9606 GN=LDHA PE=1 SV=2	95	14	13	36.7	52	332	8.27	14	16	16	4	8	4	10	12	11	4	8	3
High	Q9UJS0	Calcium-binding mitochondrial carrier protein Aralar2 OS=Homo sapiens OX=9606 GN=SLC25A13 PE=1 SV=2	95	20	15	74.1	38	675	8.62	13	15	19	3	7	6	13	15	17	3	7	6
High	Q13428	Treacle protein OS=Homo sapiens OX=9606 GN=TCOF1 PE=1 SV=3	95	20	20	152	16	1488	9.04	5	6	7	11	10	10	5	5	7	11	9	10
High	P05388	60S acidic ribosomal protein P0 OS=Homo sapiens OX=9606 GN=RPLP0 PE=1 SV=1	94	8	8	34.3	28	317	5.97	8	8	9	10	7	11	7	7	7	7	6	7
High	Q7RTS7	SWISS-PROT:Q7RTS7 Tax_Id=9606 Gene_Symbol=KRT74 Keratin, type II cytoskeletal 74	94	5	1	57.8	7	529	7.71	12	17	30	5	6	7	3	3	5	2	3	2
High	P07947	Tyrosine-protein kinase Yes OS=Homo sapiens OX=9606 GN=YES1 PE=1 SV=3	94	18	10	60.8	39	543	6.74	8	9	10	8	6	8	7	7	7	7	5	8
High	Q9ULV4	Coronin-1C OS=Homo sapiens OX=9606 GN=CORO1C PE=1 SV=1	94	18	17	53.2	46	474	7.08	16	12	15	4	5	4	13	11	13	4	5	4
High	P31629	Transcription factor HIVEP2 OS=Homo sapiens OX=9606 GN=HIVEP2 PE=1 SV=2	94	36	33	268.9	18	2446	6.96				23	27	24				23	27	23
High	Q32MZ4	Leucine-rich repeat flightless-interacting protein 1 OS=Homo sapiens OX=9606 GN=LRRFIP1 PE=1 SV=2	94	15	14	89.2	27	808	4.65	8	7	11	7	10	7	8	7	9	6	8	7
High	P09661	U2 small nuclear ribonucleoprotein A' OS=Homo sapiens OX=9606 GN=SNRPA1 PE=1 SV=2	94	12	12	28.4	47	255	8.62	3	2	1	13	18	13	3	2	1	10	10	8
High	P08865	40S ribosomal protein SA OS=Homo sapiens OX=9606 GN=RPSA PE=1 SV=4	93	10	10	32.8	52	295	4.87	11	10	11	6	7	7	9	9	7	5	6	6
High	Q15437	Protein transport protein Sec23B OS=Homo sapiens OX=9606 GN=SEC23B PE=1 SV=2	93	16	13	86.4	26	767	6.89	11	11	12	7	7	8	10	11	12	7	7	7
High	Q14151	Scaffold attachment factor B2 OS=Homo sapiens OX=9606 GN=SAFB2 PE=1 SV=1	93	18	11	107.4	25	953	6.16	13	17	15	4	4	6	11	13	12	4	3	5
High	P02769	SWISS-PROT:P02769 (Bos taurus) Bovine serum albumin precursor	92	20	16	69.2	39	607	6.18	5	18	3	10	13	9	5	13	3	9	11	8
High	Q04917	14-3-3 protein eta OS=Homo sapiens OX=9606 GN=YWHAH PE=1 SV=4	92	12	8	28.2	49	246	4.84	9	9	12	8	9	9	7	6	6	5	6	6
High	P31040	Succinate dehydrogenase [ubiquinone] flavoprotein subunit, mitochondrial OS=Homo sapiens OX=9606 GN=SDHA PE=1 SV=2	92	18	18	72.6	33	664	7.39	11	11	12	4	11	8	11	11	10	4	11	8
High	O95372	Acyl-protein thioesterase 2 OS=Homo sapiens OX=9606 GN=LYPLA2 PE=1 SV=1	92	9	9	24.7	52	231	7.23	11	14	12	3	7	4	7	8	8	3	6	4
High	O95793	Double-stranded RNA-binding protein Staufen homolog 1 OS=Homo sapiens OX=9606 GN=STAU1 PE=1 SV=2	91	19	17	63.1	41	577	9.44	4	7	8	10	11	9	3	6	7	10	11	9
High	P31947	14-3-3 protein sigma OS=Homo sapiens OX=9606 GN=SFN PE=1 SV=1	91	12	8	27.8	53	248	4.74	7	10	13	7	6	7	5	7	7	6	4	4
High	Q07666	KH domain-containing, RNA-binding, signal transduction-associated protein 1 OS=Homo sapiens OX=9606 GN=KHDRBS1 PE=1 SV=	91	7	7	48.2	24	443	8.66	10	6	11	9	5	11	6	4	7	5	5	5
High	O43795	Unconventional myosin-Ib OS=Homo sapiens OX=9606 GN=MYO1B PE=1 SV=3	91	29	29	131.9	30	1136	9.38	10	10	12	2	1	2	10	9	12	2	1	2
High	Q08J23	RNA cytosine C(5)-methyltransferase NSUN2 OS=Homo sapiens OX=9606 GN=NSUN2 PE=1 SV=2	90	22	22	86.4	40	767	6.77	11	10	11	6	11	9	10	9	8	6	10	9
High	Q9NQX4	Unconventional myosin-Vc OS=Homo sapiens OX=9606 GN=MYO5C PE=1 SV=2	90	27	23	202.7	17	1742	7.71	8	11	11	2	3	3	7	11	9	2	3	3
High	P63279	SUMO-conjugating enzyme UBC9 OS=Homo sapiens OX=9606 GN=UBE2I PE=1 SV=1	90	7	7	18	34	158	8.66	5	6	5	5	5	3	4	4	4	3	3	2
High	Q14254	Flotillin-2 OS=Homo sapiens OX=9606 GN=FLOT2 PE=1 SV=2	90	15	15	47	44	428	5.25	12	11	11	2	2	2	11	11	10	2	2	2
High	P55196	Afadin OS=Homo sapiens OX=9606 GN=AFDN PE=1 SV=3	90	30	30	206.7	23	1824	6.47	11	14	15	4	5	3	11	14	15	4	5	3
High	O00468	Agrin OS=Homo sapiens OX=9606 GN=AGRN PE=1 SV=6	89	27	27	217.2	19	2068	6.39	10	8	4	6	8	4	8	6	4	5	8	3
High	P31930	Cytochrome b-c1 complex subunit 1, mitochondrial OS=Homo sapiens OX=9606 GN=UQCRC1 PE=1 SV=3	89	13	12	52.6	40	480	6.37	12	11	12	9	9	9	10	11	11	8	8	8
High	P56192	Methionine--tRNA ligase, cytoplasmic OS=Homo sapiens OX=9606 GN=MARS1 PE=1 SV=2	88	18	18	101.1	25	900	6.16	10	9	13	1	4	2	9	8	12	1	4	2
High	Q13057	Bifunctional coenzyme A synthase OS=Homo sapiens OX=9606 GN=COASY PE=1 SV=4	88	16	16	62.3	35	564	6.99	7	9	12	6	8	7	7	9	11	6	8	7
High	Q9HCC0	Methylcrotonoyl-CoA carboxylase beta chain, mitochondrial OS=Homo sapiens OX=9606 GN=MCCC2 PE=1 SV=1	88	19	19	61.3	41	563	7.68	8	7	8	8	11	8	7	7	7	7	11	8
High	P42167	Lamina-associated polypeptide 2, isoforms beta/gamma OS=Homo sapiens OX=9606 GN=TMPO PE=1 SV=2	88	12	7	50.6	41	454	9.38	12	12	10	8	7	6	10	9	8	8	7	6
High	O60547	GDP-mannose 4,6 dehydratase OS=Homo sapiens OX=9606 GN=GMDS PE=1 SV=1	88	14	14	41.9	45	372	7.31	9	9	10	6	10	7	9	9	9	6	8	7
High	Q9P0K7	Ankycorbin OS=Homo sapiens OX=9606 GN=RAI14 PE=1 SV=2	88	28	28	110	36	980	6.21	13	18	17				13	18	17			
High	Q6YN16	Hydroxysteroid dehydrogenase-like protein 2 OS=Homo sapiens OX=9606 GN=HSDL2 PE=1 SV=1	88	17	17	45.4	50	418	7.99	11	10	9	4	5	4	9	9	7	4	5	4
High	P12931	Proto-oncogene tyrosine-protein kinase Src OS=Homo sapiens OX=9606 GN=SRC PE=1 SV=3	88	18	10	59.8	41	536	7.42	8	9	10	6	6	8	8	9	9	6	6	7
High	P55786	Puromycin-sensitive aminopeptidase OS=Homo sapiens OX=9606 GN=NPEPPS PE=1 SV=2	88	19	19	103.2	23	919	5.72	13	16	16	5	6	5	13	14	13	5	6	5
High	P62750	60S ribosomal protein L23a OS=Homo sapiens OX=9606 GN=RPL23A PE=1 SV=1	88	8	8	17.7	33	156	10.45	6	8	8	9	12	9	5	7	6	6	5	5
High	Q9UPU5	Ubiquitin carboxyl-terminal hydrolase 24 OS=Homo sapiens OX=9606 GN=USP24 PE=1 SV=3	88	29	29	294.2	14	2620	6.14	14	8	14	2	3	3	14	8	14	2	3	3
High	Q99832	T-complex protein 1 subunit eta OS=Homo sapiens OX=9606 GN=CCT7 PE=1 SV=2	87	17	17	59.3	38	543	7.65	8	9	7	9	11	6	6	8	6	8	11	6
High	Q96PK6	RNA-binding protein 14 OS=Homo sapiens OX=9606 GN=RBM14 PE=1 SV=2	87	17	17	69.4	30	669	9.67	13	11	16	6	7	7	10	8	14	6	6	7
High	Q14677	Clathrin interactor 1 OS=Homo sapiens OX=9606 GN=CLINT1 PE=1 SV=1	87	14	14	68.2	28	625	6.42	5	7	8	3	3	2	5	7	8	3	3	2
High	P22234	Multifunctional protein ADE2 OS=Homo sapiens OX=9606 GN=PAICS PE=1 SV=3	87	13	13	47	39	425	7.23	7	9	14	7	9	8	5	6	9	7	8	8
High	Q9HBD1	Roquin-2 OS=Homo sapiens OX=9606 GN=RC3H2 PE=1 SV=2	87	23	19	131.6	30	1191	6.89				17	16	16				15	14	15
High	P05091	Aldehyde dehydrogenase, mitochondrial OS=Homo sapiens OX=9606 GN=ALDH2 PE=1 SV=2	87	19	16	56.3	44	517	7.05	11	9	11	6	6	6	7	7	9	6	6	6
High	Q9Y6K5	2'-5'-oligoadenylate synthase 3 OS=Homo sapiens OX=9606 GN=OAS3 PE=1 SV=3	87	21	21	121.1	24	1087	8.4	6	5	3	12	14	10	6	5	3	12	14	10
High	Q86VP6	Cullin-associated NEDD8-dissociated protein 1 OS=Homo sapiens OX=9606 GN=CAND1 PE=1 SV=2	86	22	22	136.3	20	1230	5.78	8	12	10	6	7	4	8	11	10	6	7	4
High	Q9P2J5	Leucine--tRNA ligase, cytoplasmic OS=Homo sapiens OX=9606 GN=LARS1 PE=1 SV=2	86	24	24	134.4	24	1176	7.3	13	13	14		5	2	12	12	14		5	2
High	P13489	Ribonuclease inhibitor OS=Homo sapiens OX=9606 GN=RNH1 PE=1 SV=2	86	15	15	49.9	48	461	4.82	11	10	9	6	7	8	11	10	9	5	7	6
High	Q99959	Plakophilin-2 OS=Homo sapiens OX=9606 GN=PKP2 PE=1 SV=2	86	20	20	97.4	30	881	9.33	18	17	14	3	5	4	17	16	12	3	5	4
High	P08754	Guanine nucleotide-binding protein G(i) subunit alpha-3 OS=Homo sapiens OX=9606 GN=GNAI3 PE=1 SV=3	86	12	6	40.5	44	354	5.69	8	7	10	5	7	6	6	5	9	4	6	5
High	P42704	Leucine-rich PPR motif-containing protein, mitochondrial OS=Homo sapiens OX=9606 GN=LRPPRC PE=1 SV=3	85	25	25	157.8	21	1394	6.13	10	7	8	6	7	5	10	7	8	6	7	5
High	P07195	L-lactate dehydrogenase B chain OS=Homo sapiens OX=9606 GN=LDHB PE=1 SV=2	85	13	12	36.6	46	334	6.05	13	15	14	4	4	2	10	12	8	4	4	2
High	O60841	Eukaryotic translation initiation factor 5B OS=Homo sapiens OX=9606 GN=EIF5B PE=1 SV=4	85	16	16	138.7	19	1220	5.49	4	4	7	8	9	9	4	4	7	8	9	9
High	Q16643	Drebrin OS=Homo sapiens OX=9606 GN=DBN1 PE=1 SV=4	85	10	10	71.4	24	649	4.45	13	9	12	6	5	5	10	7	10	5	4	5
High	Q86UE4	Protein LYRIC OS=Homo sapiens OX=9606 GN=MTDH PE=1 SV=2	84	17	17	63.8	38	582	9.32	5	5	6	7	10	9	5	4	6	7	10	9
High	P30153	Serine/threonine-protein phosphatase 2A 65 kDa regulatory subunit A alpha isoform OS=Homo sapiens OX=9606 GN=PPP2R1A PE	84	15	15	65.3	32	589	5.11	8	8	6	10	6	9	7	8	5	9	5	9
High	Q6PKG0	La-related protein 1 OS=Homo sapiens OX=9606 GN=LARP1 PE=1 SV=2	84	21	21	123.4	27	1096	8.82	5	5	8	7	9	8	5	5	8	7	9	8
High	Q5T4S7	E3 ubiquitin-protein ligase UBR4 OS=Homo sapiens OX=9606 GN=UBR4 PE=1 SV=1	84	35	35	573.5	9	5183	6.04	9	9	10	3	3	2	9	9	10	3	3	2
High	Q8NC51	Plasminogen activator inhibitor 1 RNA-binding protein OS=Homo sapiens OX=9606 GN=SERBP1 PE=1 SV=2	83	10	10	44.9	29	408	8.65	7	8	10	8	10	8	5	6	7	5	8	6
High	Q9Y2A7	Nck-associated protein 1 OS=Homo sapiens OX=9606 GN=NCKAP1 PE=1 SV=1	83	23	23	128.7	23	1128	6.62	6	6	9	7	11	6	6	6	7	7	11	6
High	Q9H2U2	Inorganic pyrophosphatase 2, mitochondrial OS=Homo sapiens OX=9606 GN=PPA2 PE=1 SV=2	83	13	12	37.9	49	334	7.39	11	6	9	6	9	6	9	5	8	5	6	6
High	O00567	Nucleolar protein 56 OS=Homo sapiens OX=9606 GN=NOP56 PE=1 SV=4	83	16	16	66	31	594	9.19	4	6	4	8	7	10	3	4	4	8	7	9
High	P05976	Myosin light chain 1/3, skeletal muscle isoform OS=Homo sapiens OX=9606 GN=MYL1 PE=1 SV=3	83	4	2	21.1	18	194	5.03	9	9	13	6	8	10	3	3	3	3	3	4
High	P46977	Dolichyl-diphosphooligosaccharide--protein glycosyltransferase subunit STT3A OS=Homo sapiens OX=9606 GN=STT3A PE=1 SV=2	82	14	13	80.5	18	705	8.07	9	13	11	6	8	5	8	12	10	6	8	5
High	Q9Y446	Plakophilin-3 OS=Homo sapiens OX=9606 GN=PKP3 PE=1 SV=1	82	16	16	87	22	797	9.32	10	10	11	3	9	9	10	10	11	3	8	9
High	O00515	Ladinin-1 OS=Homo sapiens OX=9606 GN=LAD1 PE=1 SV=2	82	15	15	57.1	32	517	9.67	7	4	7	5	7	6	7	4	6	5	7	6
High	P33991	DNA replication licensing factor MCM4 OS=Homo sapiens OX=9606 GN=MCM4 PE=1 SV=5	82	19	19	96.5	26	863	6.74	12	14	18	6	7	7	11	13	15	6	6	7
High	Q9BYB4	Guanine nucleotide-binding protein subunit beta-like protein 1 OS=Homo sapiens OX=9606 GN=GNB1L PE=1 SV=2	82	13	13	35.6	46	327	7.97	9	6	7	8	7	9	7	6	7	6	5	7
High	A8MW92	PHD finger protein 20-like protein 1 OS=Homo sapiens OX=9606 GN=PHF20L1 PE=1 SV=2	81	19	19	114.9	23	1017	6.83				11	17	10				11	14	10
High	P62140	Serine/threonine-protein phosphatase PP1-beta catalytic subunit OS=Homo sapiens OX=9606 GN=PPP1CB PE=1 SV=3	81	9	3	37.2	36	327	6.19	12	9	7	6	5	5	7	7	6	6	5	5
High	Q14839	Chromodomain-helicase-DNA-binding protein 4 OS=Homo sapiens OX=9606 GN=CHD4 PE=1 SV=2	81	22	18	217.9	15	1912	5.86	4	4	8	3	7	5	4	4	8	3	7	5
High	Q5T5P2	Sickle tail protein homolog OS=Homo sapiens OX=9606 GN=KIAA1217 PE=1 SV=2	80	28	28	214	17	1943	7.06	5	8	13	1	4	2	5	8	13	1	4	2
High	P29401	Transketolase OS=Homo sapiens OX=9606 GN=TKT PE=1 SV=3	80	16	16	67.8	33	623	7.66	14	12	10	7	13	8	14	10	10	6	8	7
High	Q8TE68	Epidermal growth factor receptor kinase substrate 8-like protein 1 OS=Homo sapiens OX=9606 GN=EPS8L1 PE=1 SV=3	80	16	16	80.2	25	723	6.04	8	9	6	4	8	9	8	9	6	4	7	9
High	Q96TA1	Protein Niban 2 OS=Homo sapiens OX=9606 GN=NIBAN2 PE=1 SV=3	80	16	16	84.1	27	746	6.19	11	8	10	7	7	8	11	8	10	7	6	8
High	Q3SX14	TREMBL:Q3SX14 (Bos taurus) Similar to Gelsolin	80	14	1	85.6	18	781	6.21	11	11	13	3	9	5	9	9	9	3	6	5
High	Q99816	Tumor susceptibility gene 101 protein OS=Homo sapiens OX=9606 GN=TSG101 PE=1 SV=2	80	13	13	43.9	33	390	6.46	6	8	9	6	8	8	6	8	8	6	8	8
High	P61353	60S ribosomal protein L27 OS=Homo sapiens OX=9606 GN=RPL27 PE=1 SV=2	79	6	6	15.8	43	136	10.56	6	6	8	7	11	9	4	4	4	5	5	6
High	P16402	Histone H1.3 OS=Homo sapiens OX=9606 GN=H1-3 PE=1 SV=2	79	5	5	22.3	15	221	11.02	8	7	8	11	8	8	3	2	2	5	5	3
High	P63096	Guanine nucleotide-binding protein G(i) subunit alpha-1 OS=Homo sapiens OX=9606 GN=GNAI1 PE=1 SV=2	79	10	4	40.3	32	354	5.97	8	7	8	6	7	6	6	5	7	5	6	5
High	O75116	Rho-associated protein kinase 2 OS=Homo sapiens OX=9606 GN=ROCK2 PE=1 SV=4	78	24	21	160.8	21	1388	6.02	4	6	4	7	12	8	4	6	4	7	12	8
High	Q5JWF2	Guanine nucleotide-binding protein G(s) subunit alpha isoforms XLas OS=Homo sapiens OX=9606 GN=GNAS PE=1 SV=2	78	11	10	111	14	1037	5.03	10	8	9	5	7	6	10	8	9	5	7	6
High	P62136	Serine/threonine-protein phosphatase PP1-alpha catalytic subunit OS=Homo sapiens OX=9606 GN=PPP1CA PE=1 SV=1	78	11	4	37.5	42	330	6.33	12	9	8	6	5	5	9	8	7	6	5	5
High	Q9NS86	LanC-like protein 2 OS=Homo sapiens OX=9606 GN=LANCL2 PE=1 SV=1	78	11	11	50.8	32	450	7.43	8	9	7	8	7	7	6	8	7	7	6	6
High	Q8WWM7	Ataxin-2-like protein OS=Homo sapiens OX=9606 GN=ATXN2L PE=1 SV=2	78	16	16	113.3	22	1075	8.59	3	3	6	9	11	7	3	3	5	9	11	7
High	P05455	Lupus La protein OS=Homo sapiens OX=9606 GN=SSB PE=1 SV=2	78	16	16	46.8	33	408	7.12	9	6	8	7	9	5	9	6	8	7	9	5
High	Q6ZRV2	Protein FAM83H OS=Homo sapiens OX=9606 GN=FAM83H PE=1 SV=3	78	27	27	127	33	1179	6.98	17	21	16	2	3	1	17	20	16	1	3	1
High	Q08380	Galectin-3-binding protein OS=Homo sapiens OX=9606 GN=LGALS3BP PE=1 SV=1	78	15	15	65.3	36	585	5.27	12	9	7	3	6	5	11	9	6	3	5	5
High	Q9Y383	Putative RNA-binding protein Luc7-like 2 OS=Homo sapiens OX=9606 GN=LUC7L2 PE=1 SV=2	77	12	8	46.5	32	392	10.01	6	6	5	10	10	9	5	5	5	9	9	8
High	P98160	Basement membrane-specific heparan sulfate proteoglycan core protein OS=Homo sapiens OX=9606 GN=HSPG2 PE=1 SV=4	77	23	23	468.5	7	4391	6.51	7	9	10	1	4	3	7	9	10	1	4	3
High	P07910	Heterogeneous nuclear ribonucleoproteins C1/C2 OS=Homo sapiens OX=9606 GN=HNRNPC PE=1 SV=4	77	8	3	33.7	25	306	5.08	9	9	10	7	7	8	8	7	7	6	6	6
High	P14174	Macrophage migration inhibitory factor OS=Homo sapiens OX=9606 GN=MIF PE=1 SV=4	76	3	3	12.5	37	115	7.88	12	14	7	2	3	2	3	3	2	2	2	2
High	Q8NB78	Lysine-specific histone demethylase 1B OS=Homo sapiens OX=9606 GN=KDM1B PE=1 SV=3	76	20	20	92	30	822	7.87				14	9	14				14	9	14
High	Q05397	Focal adhesion kinase 1 OS=Homo sapiens OX=9606 GN=PTK2 PE=1 SV=2	76	19	19	119.2	22	1052	6.62	8	7	7	10	7	7	7	7	7	9	7	7
High	Q96AB3	Isochorismatase domain-containing protein 2 OS=Homo sapiens OX=9606 GN=ISOC2 PE=1 SV=1	76	6	6	22.3	52	205	7.77	9	9	7	2	1	2	6	5	6	2	1	2
High	P35637	RNA-binding protein FUS OS=Homo sapiens OX=9606 GN=FUS PE=1 SV=1	76	8	6	53.4	22	526	9.36	11	10	12	6	9	6	6	6	7	5	4	4
High	Q9NQW6	Anillin OS=Homo sapiens OX=9606 GN=ANLN PE=1 SV=2	76	21	21	124.1	23	1124	8.07	2	4	8	6	11	5	2	4	8	6	11	5
High	P53396	ATP-citrate synthase OS=Homo sapiens OX=9606 GN=ACLY PE=1 SV=3	76	24	24	120.8	26	1101	7.33	6	5	3	5	8	3	6	5	3	5	8	3
High	P78347	General transcription factor II-I OS=Homo sapiens OX=9606 GN=GTF2I PE=1 SV=2	76	24	24	112.3	26	998	6.39	5	4	6	3	6	4	5	4	6	3	6	4
High	Q96EK4	THAP domain-containing protein 11 OS=Homo sapiens OX=9606 GN=THAP11 PE=1 SV=2	76	6	6	34.4	19	314	8.98				11	13	13				5	6	6
High	P60981	Destrin OS=Homo sapiens OX=9606 GN=DSTN PE=1 SV=3	76	7	7	18.5	36	165	7.85	11	9	9	5	5	4	6	6	6	3	4	3
High	P62906	60S ribosomal protein L10a OS=Homo sapiens OX=9606 GN=RPL10A PE=1 SV=2	75	7	7	24.8	33	217	9.94	6	6	8	8	10	8	6	6	6	6	6	6
High	A0A075B6	Probable non-functional immunoglobulin kappa variable 2D-24 OS=Homo sapiens OX=9606 GN=IGKV2D-24 PE=1 SV=1	75	3	2	13.1	13	120	8.87	8	10	7	6	9	3	2	2	1	1	2	1
High	Q14573	Inositol 1,4,5-trisphosphate receptor type 3 OS=Homo sapiens OX=9606 GN=ITPR3 PE=1 SV=2	75	28	28	303.9	12	2671	6.48	6	5	5	2	3	2	6	5	5	2	3	2
High	P49756	RNA-binding protein 25 OS=Homo sapiens OX=9606 GN=RBM25 PE=1 SV=3	75	16	16	100.1	23	843	6.32	2	5	5	8	8	7	2	5	5	7	8	7
High	Q9NZB2	Constitutive coactivator of PPAR-gamma-like protein 1 OS=Homo sapiens OX=9606 GN=FAM120A PE=1 SV=2	75	18	18	121.8	22	1118	8.88	7	4	7	9	6	7	7	4	6	8	6	7
High	Q5SSJ5	Heterochromatin protein 1-binding protein 3 OS=Homo sapiens OX=9606 GN=HP1BP3 PE=1 SV=1	75	15	15	61.2	28	553	9.67	5	5	5	9	8	9	5	5	5	9	8	9
High	P35555	Fibrillin-1 OS=Homo sapiens OX=9606 GN=FBN1 PE=1 SV=4	74	26	25	312.1	11	2871	4.93				22	27	24				20	22	21
High	P45880	Voltage-dependent anion-selective channel protein 2 OS=Homo sapiens OX=9606 GN=VDAC2 PE=1 SV=2	74	11	11	31.5	50	294	7.56	9	8	10	5	7	5	9	8	9	5	6	5
High	P55011	Solute carrier family 12 member 2 OS=Homo sapiens OX=9606 GN=SLC12A2 PE=1 SV=1	74	18	18	131.4	19	1212	6.4	7	11	9	5	6	4	6	9	8	5	6	4
High	P40227	T-complex protein 1 subunit zeta OS=Homo sapiens OX=9606 GN=CCT6A PE=1 SV=3	74	15	11	58	29	531	6.68	10	11	9	2	6	4	9	10	9	2	6	4
High	Q9ULC5	Long-chain-fatty-acid--CoA ligase 5 OS=Homo sapiens OX=9606 GN=ACSL5 PE=1 SV=1	74	19	19	75.9	36	683	6.92	8	10	10	1	5	4	8	9	10	1	5	4
High	Q9BS26	Endoplasmic reticulum resident protein 44 OS=Homo sapiens OX=9606 GN=ERP44 PE=1 SV=1	74	11	11	46.9	28	406	5.26	4	4	4	9	12	7	4	4	4	7	9	6
High	Q96KR1	Zinc finger RNA-binding protein OS=Homo sapiens OX=9606 GN=ZFR PE=1 SV=2	74	20	20	116.9	24	1074	9.04	1	1	1	9	10	8	1	1	1	9	10	8
High	P61158	Actin-related protein 3 OS=Homo sapiens OX=9606 GN=ACTR3 PE=1 SV=3	74	14	12	47.3	46	418	5.88	11	10	10	4	6	6	11	10	10	4	6	6
High	P15531	Nucleoside diphosphate kinase A OS=Homo sapiens OX=9606 GN=NME1 PE=1 SV=1	73	7	3	17.1	53	152	6.19	7	7	9	9	7	8	5	5	4	4	4	4
High	P28288	ATP-binding cassette sub-family D member 3 OS=Homo sapiens OX=9606 GN=ABCD3 PE=1 SV=1	73	18	18	75.4	30	659	9.36	20	19	18	1		1	15	16	15	1		1
High	Q6NXG1	Epithelial splicing regulatory protein 1 OS=Homo sapiens OX=9606 GN=ESRP1 PE=1 SV=2	73	13	11	75.5	30	681	6.68	6	7	6	5	6	6	6	7	5	5	5	6
High	P62913	60S ribosomal protein L11 OS=Homo sapiens OX=9606 GN=RPL11 PE=1 SV=2	73	5	5	20.2	34	178	9.6	11	11	6	7	9	4	5	5	3	5	3	3
High	P62244	40S ribosomal protein S15a OS=Homo sapiens OX=9606 GN=RPS15A PE=1 SV=2	73	6	6	14.8	42	130	10.13	9	10	7	7	8	6	6	6	5	5	4	4
High	P50914	60S ribosomal protein L14 OS=Homo sapiens OX=9606 GN=RPL14 PE=1 SV=4	72	5	5	23.4	27	215	10.93	3	3	3	8	8	9	3	3	2	5	5	5
High	Q5TEC6	Histone H3-7 OS=Homo sapiens OX=9606 GN=H3-7 PE=1 SV=1	72	3	1	15.4	20	136	11.27	8	7	13	9	6	10	1	1	3	2	3	2
High	P54886	Delta-1-pyrroline-5-carboxylate synthase OS=Homo sapiens OX=9606 GN=ALDH18A1 PE=1 SV=2	72	16	16	87.2	24	795	7.12	11	13	13	2	2	2	11	13	12	2	2	2
High	Q14257	Reticulocalbin-2 OS=Homo sapiens OX=9606 GN=RCN2 PE=1 SV=1	72	13	13	36.9	45	317	4.4	13	14	17	1	2	1	12	11	12	1	2	1
High	Q9NUQ9	CYFIP-related Rac1 interactor B OS=Homo sapiens OX=9606 GN=CYRIB PE=1 SV=1	72	12	12	36.7	52	324	6.06	10	12	11	2	4	3	8	10	8	2	4	3
High	P15880	40S ribosomal protein S2 OS=Homo sapiens OX=9606 GN=RPS2 PE=1 SV=2	72	9	9	31.3	34	293	10.24	9	8	7	2	5	3	7	6	6	2	5	3
High	P12004	Proliferating cell nuclear antigen OS=Homo sapiens OX=9606 GN=PCNA PE=1 SV=1	72	11	11	28.8	50	261	4.69	10	11	9	6	3	6	8	9	8	6	3	6
High	P18754	Regulator of chromosome condensation OS=Homo sapiens OX=9606 GN=RCC1 PE=1 SV=1	71	14	14	44.9	45	421	7.52	2	2	4	4	5	3	2	2	3	4	5	3
High	Q63HN8	E3 ubiquitin-protein ligase RNF213 OS=Homo sapiens OX=9606 GN=RNF213 PE=1 SV=3	71	23	23	591	5	5207	6.48	14	15	11	2	7	4	14	15	10	2	6	4
High	P00558	Phosphoglycerate kinase 1 OS=Homo sapiens OX=9606 GN=PGK1 PE=1 SV=3	71	13	13	44.6	41	417	8.1	9	9	10	4	4	4	8	8	9	4	4	4
High	P63241	Eukaryotic translation initiation factor 5A-1 OS=Homo sapiens OX=9606 GN=EIF5A PE=1 SV=2	71	10	10	16.8	56	154	5.24	9	9	6	4	10	4	7	8	6	4	6	4
High	P46781	40S ribosomal protein S9 OS=Homo sapiens OX=9606 GN=RPS9 PE=1 SV=3	71	10	10	22.6	43	194	10.65	8	9	8	5	8	5	8	8	8	5	8	5
High	Q14240	Eukaryotic initiation factor 4A-II OS=Homo sapiens OX=9606 GN=EIF4A2 PE=1 SV=2	71	10	2	46.4	26	407	5.48	8	8	10	7	6	7	7	7	9	7	6	7
High	P42166	Lamina-associated polypeptide 2, isoform alpha OS=Homo sapiens OX=9606 GN=TMPO PE=1 SV=2	71	16	11	75.4	34	694	7.66	8	8	12	4	4	4	7	7	10	4	4	4
High	Q96HS1	Serine/threonine-protein phosphatase PGAM5, mitochondrial OS=Homo sapiens OX=9606 GN=PGAM5 PE=1 SV=2	71	12	12	32	43	289	8.68	10	10	10	6	6	6	9	10	9	6	6	6
High	P22392	Nucleoside diphosphate kinase B OS=Homo sapiens OX=9606 GN=NME2 PE=1 SV=1	71	7	3	17.3	53	152	8.41	6	6	11	9	5	8	5	5	5	5	3	5
High	P62879	Guanine nucleotide-binding protein G(I)/G(S)/G(T) subunit beta-2 OS=Homo sapiens OX=9606 GN=GNB2 PE=1 SV=3	71	11	5	37.3	46	340	6	11	11	12	5	5	4	10	10	9	5	5	4
High	P18085	ADP-ribosylation factor 4 OS=Homo sapiens OX=9606 GN=ARF4 PE=1 SV=3	70	8	6	20.5	53	180	7.14	9	12	10	5	5	5	6	8	7	5	5	5
High	Q92878	DNA repair protein RAD50 OS=Homo sapiens OX=9606 GN=RAD50 PE=1 SV=1	70	24	24	153.8	19	1312	6.89	7	5	7	2	6	5	7	5	7	2	6	5
High	Q9Y266	Nuclear migration protein nudC OS=Homo sapiens OX=9606 GN=NUDC PE=1 SV=1	70	11	11	38.2	36	331	5.38	7	6	9	6	7	9	7	4	7	5	6	7
High	Q9UQ35	Serine/arginine repetitive matrix protein 2 OS=Homo sapiens OX=9606 GN=SRRM2 PE=1 SV=2	70	19	19	299.4	11	2752	12.06	3	3	5	9	10	8	3	3	5	9	10	8
High	Q12797	Aspartyl/asparaginyl beta-hydroxylase OS=Homo sapiens OX=9606 GN=ASPH PE=1 SV=3	70	16	16	85.8	26	758	5.01	4	4	5	7	8	5	4	4	4	7	8	5
High	P50454	Serpin H1 OS=Homo sapiens OX=9606 GN=SERPINH1 PE=1 SV=2	70	14	14	46.4	37	418	8.69	9	8	11	4	5	4	8	7	9	3	4	3
High	P26641	Elongation factor 1-gamma OS=Homo sapiens OX=9606 GN=EEF1G PE=1 SV=3	69	15	15	50.1	37	437	6.67	10	10	9	5	7	3	9	9	9	5	7	3
High	Q9BYK8	Helicase with zinc finger domain 2 OS=Homo sapiens OX=9606 GN=HELZ2 PE=1 SV=6	69	25	25	294.5	11	2649	7.49	3	3	1	13	10	15	3	3	1	12	10	15
High	O95831	Apoptosis-inducing factor 1, mitochondrial OS=Homo sapiens OX=9606 GN=AIFM1 PE=1 SV=1	69	12	12	66.9	25	613	8.95	8	9	9	4	7	3	8	9	9	4	7	3
High	P23921	Ribonucleoside-diphosphate reductase large subunit OS=Homo sapiens OX=9606 GN=RRM1 PE=1 SV=1	69	16	16	90	23	792	7.15	7	7	5	9	10	6	7	7	5	9	10	6
High	O94905	Erlin-2 OS=Homo sapiens OX=9606 GN=ERLIN2 PE=1 SV=1	69	12	9	37.8	44	339	5.62	10	11	8	4	3	3	9	10	7	4	3	3
High	Q9NW13	RNA-binding protein 28 OS=Homo sapiens OX=9606 GN=RBM28 PE=1 SV=3	69	13	13	85.7	18	759	9.22	3	3	5	8	8	9	3	3	5	8	8	9
High	P61313	60S ribosomal protein L15 OS=Homo sapiens OX=9606 GN=RPL15 PE=1 SV=2	69	8	8	24.1	39	204	11.62	6	5	5	5	8	6	6	5	4	3	6	4
High	P05387	60S acidic ribosomal protein P2 OS=Homo sapiens OX=9606 GN=RPLP2 PE=1 SV=1	69	9	8	11.7	93	115	4.54	5	5	5	4	9	6	5	5	5	4	7	4
High	Q99569	Plakophilin-4 OS=Homo sapiens OX=9606 GN=PKP4 PE=1 SV=2	68	20	20	131.8	24	1192	8.94	6	6	11	1	1	2	6	6	10	1	1	2
High	Q9H444	Charged multivesicular body protein 4b OS=Homo sapiens OX=9606 GN=CHMP4B PE=1 SV=1	68	9	9	24.9	47	224	4.82	5	8	8	3	3	3	5	8	8	3	3	3
High	P35914	Hydroxymethylglutaryl-CoA lyase, mitochondrial OS=Homo sapiens OX=9606 GN=HMGCL PE=1 SV=2	68	10	10	34.3	36	325	8.54	5	6	7	6	6	6	5	6	7	6	6	6
High	P35998	26S proteasome regulatory subunit 7 OS=Homo sapiens OX=9606 GN=PSMC2 PE=1 SV=3	68	17	17	48.6	44	433	5.95	2	3	2	11	11	11	2	3	2	10	11	10
High	P36957	Dihydrolipoyllysine-residue succinyltransferase component of 2-oxoglutarate dehydrogenase complex, mitochondrial OS=Homo s	68	10	10	48.7	28	453	8.95	6	7	9	6	4	3	6	6	8	5	3	3

High	O00148	ATP-dependent RNA helicase DDX39A OS=Homo sapiens OX=9606 GN=DDX39A PE=1 SV=2	68	12	4	49.1	34	427	5.68	10	8	13	5	6	4	9	8	11	4	6	4
High	P09543	2',3'-cyclic-nucleotide 3'-phosphodiesterase OS=Homo sapiens OX=9606 GN=CNP PE=1 SV=2	68	15	15	47.5	34	421	9.07	5	5	9	7	6	9	5	5	8	7	6	9
High	O15438	ATP-binding cassette sub-family C member 3 OS=Homo sapiens OX=9606 GN=ABCC3 PE=1 SV=3	68	22	20	169.2	19	1527	7.2	17	20	18			1	14	17	15			1
High	Q8N1G4	Leucine-rich repeat-containing protein 47 OS=Homo sapiens OX=9606 GN=LRRC47 PE=1 SV=1	68	14	14	63.4	30	583	8.28	7	5	8	6	6	4	7	5	8	6	6	4
High	Q9Y2X3	Nucleolar protein 58 OS=Homo sapiens OX=9606 GN=NOP58 PE=1 SV=1	67	13	13	59.5	31	529	8.92	3	4	7	5	6	6	3	4	7	5	6	6
High	Q15046	Lysine--tRNA ligase OS=Homo sapiens OX=9606 GN=KARS1 PE=1 SV=3	67	13	13	68	25	597	6.35	10	4	8	5	5	5	10	4	7	5	5	5
High	Q13263	Transcription intermediary factor 1-beta OS=Homo sapiens OX=9606 GN=TRIM28 PE=1 SV=5	67	13	13	88.5	26	835	5.77	5	5	8	6	5	6	5	5	8	6	5	6
High	P51114	Fragile X mental retardation syndrome-related protein 1 OS=Homo sapiens OX=9606 GN=FXR1 PE=1 SV=3	67	17	15	69.7	36	621	6.15	6	7	4	8	8	7	6	7	4	7	7	6
High	O43837	Isocitrate dehydrogenase [NAD] subunit beta, mitochondrial OS=Homo sapiens OX=9606 GN=IDH3B PE=1 SV=2	67	14	14	42.2	48	385	8.46	7	8	7	5	6	6	7	8	7	5	6	6
High	P60903	Protein S100-A10 OS=Homo sapiens OX=9606 GN=S100A10 PE=1 SV=2	67	3	3	11.2	18	97	7.37	5	3	5	3	5	4	2	1	2	3	3	3
High	Q6UXN9	WD repeat-containing protein 82 OS=Homo sapiens OX=9606 GN=WDR82 PE=1 SV=1	67	11	11	35.1	35	313	7.69	1	1	1	10	12	11	1	1	1	8	8	9
High	P61204	ADP-ribosylation factor 3 OS=Homo sapiens OX=9606 GN=ARF3 PE=1 SV=2	66	7	4	20.6	48	181	7.43	9	11	11	4	6	4	7	7	7	4	5	4
High	P52789	Hexokinase-2 OS=Homo sapiens OX=9606 GN=HK2 PE=1 SV=2	66	20	17	102.3	25	917	6.05	7	9	10	1	2	1	7	9	10	1	2	1
High	P15170	Eukaryotic peptide chain release factor GTP-binding subunit ERF3A OS=Homo sapiens OX=9606 GN=GSPT1 PE=1 SV=1	66	15	15	55.7	37	499	5.62	8	7	11	3	2	6	7	7	10	3	2	6
High	Q15084	Protein disulfide-isomerase A6 OS=Homo sapiens OX=9606 GN=PDIA6 PE=1 SV=1	66	8	8	48.1	26	440	5.08	9	9	9	5	5	5	7	7	7	5	5	5
High	O15231	Zinc finger protein 185 OS=Homo sapiens OX=9606 GN=ZNF185 PE=1 SV=3	65	18	18	73.5	35	689	7.01	10	8	9	2	3	4	10	8	9	2	3	4
High	Q6XQN6	Nicotinate phosphoribosyltransferase OS=Homo sapiens OX=9606 GN=NAPRT PE=1 SV=2	65	11	11	57.5	33	538	5.68	6	4	9	6	7	7	6	4	9	6	5	7
High	O60264	SWI/SNF-related matrix-associated actin-dependent regulator of chromatin subfamily A member 5 OS=Homo sapiens OX=9606 GI	65	18	18	121.8	18	1052	8.09	6	9	8	8	9	5	6	9	8	8	9	5
High	Q9BVI0	PHD finger protein 20 OS=Homo sapiens OX=9606 GN=PHF20 PE=1 SV=2	65	18	18	115.3	23	1012	6.99				9	10	10				9	10	10
High	Q14697	Neutral alpha-glucosidase AB OS=Homo sapiens OX=9606 GN=GANAB PE=1 SV=3	65	20	20	106.8	25	944	6.14	8	12	11	2	2	4	8	12	11	2	2	4
High	P62829	60S ribosomal protein L23 OS=Homo sapiens OX=9606 GN=RPL23 PE=1 SV=1	65	5	5	14.9	44	140	10.51	4	7	3	4	7	5	3	4	3	3	5	4
High	P36873	Serine/threonine-protein phosphatase PP1-gamma catalytic subunit OS=Homo sapiens OX=9606 GN=PPP1CC PE=1 SV=1	65	8	1	37	31	323	6.54	9	8	7	4	3	3	6	7	6	4	3	3
High	P49589	Cysteine--tRNA ligase, cytoplasmic OS=Homo sapiens OX=9606 GN=CARS1 PE=1 SV=3	65	14	14	85.4	21	748	6.76	5	5	5	7	6	4	5	5	5	6	6	4
High	P35222	Catenin beta-1 OS=Homo sapiens OX=9606 GN=CTNNB1 PE=1 SV=1	65	13	9	85.4	18	781	5.86	10	9	8	2	3	2	8	9	8	2	3	2
High	Q9UQE7	Structural maintenance of chromosomes protein 3 OS=Homo sapiens OX=9606 GN=SMC3 PE=1 SV=2	65	22	22	141.5	22	1217	7.18	6	4	9	2	6	3	6	4	9	2	6	3
High	P30050	60S ribosomal protein L12 OS=Homo sapiens OX=9606 GN=RPL12 PE=1 SV=1	64	5	5	17.8	45	165	9.42	5	5	5	5	8	5	5	5	5	5	5	5
High	O75534	Cold shock domain-containing protein E1 OS=Homo sapiens OX=9606 GN=CSDE1 PE=1 SV=2	64	16	16	88.8	21	798	6.25	3	2	3	8	8	11	3	2	3	8	8	11
High	P35813	Protein phosphatase 1A OS=Homo sapiens OX=9606 GN=PPM1A PE=1 SV=1	64	7	4	42.4	23	382	5.36	6	8	7	6	5	7	4	4	6	3	2	4
High	P62873	Guanine nucleotide-binding protein G(I)/G(S)/G(T) subunit beta-1 OS=Homo sapiens OX=9606 GN=GNB1 PE=1 SV=3	64	11	5	37.4	40	340	6	9	8	8	6	4	5	9	8	7	6	4	5
High	P62888	60S ribosomal protein L30 OS=Homo sapiens OX=9606 GN=RPL30 PE=1 SV=2	64	5	5	12.8	57	115	9.63	5	3	6	5	7	4	4	3	4	5	4	4
High	Q13247	Serine/arginine-rich splicing factor 6 OS=Homo sapiens OX=9606 GN=SRSF6 PE=1 SV=2	64	9	8	39.6	23	344	11.43	7	5	4	13	6	7	7	5	4	9	5	6
High	P24752	Acetyl-CoA acetyltransferase, mitochondrial OS=Homo sapiens OX=9606 GN=ACAT1 PE=1 SV=1	64	12	12	45.2	30	427	8.85	2	4	5	8	7	7	2	4	5	8	7	7
High	P11166	Solute carrier family 2, facilitated glucose transporter member 1 OS=Homo sapiens OX=9606 GN=SLC2A1 PE=1 SV=2	64	7	7	54	12	492	8.72	10	10	11	2	4	5	6	4	6	2	3	4
High	P23284	Peptidyl-prolyl cis-trans isomerase B OS=Homo sapiens OX=9606 GN=PPIB PE=1 SV=2	64	12	12	23.7	50	216	9.41	8	7	7	9	9	8	8	7	7	8	8	8
High	Q09028	Histone-binding protein RBBP4 OS=Homo sapiens OX=9606 GN=RBBP4 PE=1 SV=3	64	9	4	47.6	22	425	4.89	4	3	6	9	8	8	4	3	5	7	7	7
High	P04424	Argininosuccinate lyase OS=Homo sapiens OX=9606 GN=ASL PE=1 SV=4	64	14	14	51.6	35	464	6.48	6	6	5	4	5	4	6	6	5	4	5	4
High	Q99543	DnaJ homolog subfamily C member 2 OS=Homo sapiens OX=9606 GN=DNAJC2 PE=1 SV=4	63	14	14	72	24	621	8.7	4	6	5	5	5	8	4	6	5	5	5	8
High	P17655	Calpain-2 catalytic subunit OS=Homo sapiens OX=9606 GN=CAPN2 PE=1 SV=6	63	14	14	79.9	28	700	4.98	7	6	4	7	3	6	7	6	4	7	3	6
High	P04080	Cystatin-B OS=Homo sapiens OX=9606 GN=CSTB PE=1 SV=2	63	5	5	11.1	77	98	7.56	5	7	8	4	8	5	4	5	4	3	4	3
High	P62263	40S ribosomal protein S14 OS=Homo sapiens OX=9606 GN=RPS14 PE=1 SV=3	63	6	6	16.3	38	151	10.05	6	5	7	9	7	8	4	4	5	5	5	5
High	P62993	Growth factor receptor-bound protein 2 OS=Homo sapiens OX=9606 GN=GRB2 PE=1 SV=1	63	12	12	25.2	61	217	6.32	7	6	10	6	6	6	6	6	8	6	6	6
High	Q96C19	EF-hand domain-containing protein D2 OS=Homo sapiens OX=9606 GN=EFHD2 PE=1 SV=1	63	11	11	26.7	38	240	5.2	8	10	9	5	4	4	8	9	8	5	4	4
High	Q14258	E3 ubiquitin/ISG15 ligase TRIM25 OS=Homo sapiens OX=9606 GN=TRIM25 PE=1 SV=2	63	13	13	70.9	29	630	8.09	10	12	7	4	4	4	9	11	7	4	3	4
High	P09496	Clathrin light chain A OS=Homo sapiens OX=9606 GN=CLTA PE=1 SV=1	63	7	7	27.1	20	248	4.51	7	7	7	4	6	6	6	6	5	4	6	6
High	P11388	DNA topoisomerase 2-alpha OS=Homo sapiens OX=9606 GN=TOP2A PE=1 SV=3	62	16	8	174.3	11	1531	8.72	6	12	12	3	6	3	6	12	10	3	5	3
High	Q04837	Single-stranded DNA-binding protein, mitochondrial OS=Homo sapiens OX=9606 GN=SSBP1 PE=1 SV=1	62	7	7	17.2	52	148	9.6	10	8	8	6	8	6	7	6	7	5	5	5
High	Q9NUQ6	SPATS2-like protein OS=Homo sapiens OX=9606 GN=SPATS2L PE=1 SV=2	62	12	12	61.7	28	558	9.64	4	4	3	6	9	8	4	4	3	6	8	7
High	Q9Y520	Protein PRRC2C OS=Homo sapiens OX=9606 GN=PRRC2C PE=1 SV=4	62	19	19	316.7	8	2896	9.13		1	1	7	9	8		1	1	7	9	8
High	Q99873	Protein arginine N-methyltransferase 1 OS=Homo sapiens OX=9606 GN=PRMT1 PE=1 SV=3	62	10	10	42.4	33	371	5.35	4	4	8	6	6	7	4	4	8	6	6	7
High	Q13596	Sorting nexin-1 OS=Homo sapiens OX=9606 GN=SNX1 PE=1 SV=3	62	12	10	59	27	522	5.15	5	3	6	6	9	8	5	3	6	6	9	7
High	Q96EZ8	Microspherule protein 1 OS=Homo sapiens OX=9606 GN=MCRS1 PE=1 SV=1	62	15	15	51.8	39	462	9.38				9	10	7				9	8	7
High	Q9H7Z6	Histone acetyltransferase KAT8 OS=Homo sapiens OX=9606 GN=KAT8 PE=1 SV=2	62	13	12	52.4	32	458	8.27				6	12	8				6	11	7
High	Q13435	Splicing factor 3B subunit 2 OS=Homo sapiens OX=9606 GN=SF3B2 PE=1 SV=2	62	16	16	100.2	22	895	5.67	2	3	2	5	9	5	2	3	2	5	9	5
High	Q99439	Calponin-2 OS=Homo sapiens OX=9606 GN=CNN2 PE=1 SV=4	62	11	11	33.7	54	309	7.33	6	5	7	5	6	6	5	5	5	5	6	6
High	Q1KMD3	Heterogeneous nuclear ribonucleoprotein U-like protein 2 OS=Homo sapiens OX=9606 GN=HNRNPUL2 PE=1 SV=1	62	13	13	85.1	19	747	4.91	8	7	9	4	5	5	8	7	9	4	5	5
High	P61081	NEDD8-conjugating enzyme Ubc12 OS=Homo sapiens OX=9606 GN=UBE2M PE=1 SV=1	61	7	7	20.9	33	183	7.69	7	7	8	5	5	7	7	6	7	5	5	7
High	Q7L0Y3	tRNA methyltransferase 10 homolog C OS=Homo sapiens OX=9606 GN=TRMT10C PE=1 SV=2	61	16	16	47.3	41	403	9.36	5	8	5	4	8	3	5	8	5	4	8	3
High	Q8WXF1	Paraspeckle component 1 OS=Homo sapiens OX=9606 GN=PSPC1 PE=1 SV=1	61	13	13	58.7	33	523	6.67	1	1	1	9	11	9	1	1	1	9	11	9
High	Q8TEM1	Nuclear pore membrane glycoprotein 210 OS=Homo sapiens OX=9606 GN=NUP210 PE=1 SV=3	61	18	18	205	12	1887	6.81	3	2	4	3	6	7	3	2	4	3	6	7
High	Q02218	2-oxoglutarate dehydrogenase, mitochondrial OS=Homo sapiens OX=9606 GN=OGDH PE=1 SV=3	61	18	18	115.9	21	1023	6.86	5	5	5	3	2	4	5	5	5	3	2	4
High	P26368	Splicing factor U2AF 65 kDa subunit OS=Homo sapiens OX=9606 GN=U2AF2 PE=1 SV=4	61	8	8	53.5	29	475	9.09	6	4	5	5	6	7	6	4	5	5	4	5
High	Q14103	Heterogeneous nuclear ribonucleoprotein D0 OS=Homo sapiens OX=9606 GN=HNRNPD PE=1 SV=1	61	8	6	38.4	24	355	7.81	8	8	9	4	5	3	7	7	7	3	4	2
High	Q9BSH5	Haloacid dehalogenase-like hydrolase domain-containing protein 3 OS=Homo sapiens OX=9606 GN=HDHD3 PE=1 SV=1	61	8	8	28	45	251	6.71	8	8	9	4	3	4	6	6	7	4	3	4
High	P38919	Eukaryotic initiation factor 4A-III OS=Homo sapiens OX=9606 GN=EIF4A3 PE=1 SV=4	61	13	11	46.8	31	411	6.73	8	9	5	5	8	7	8	9	5	5	8	7
High	Q01167	Forkhead box protein K2 OS=Homo sapiens OX=9606 GN=FOXK2 PE=1 SV=3	61	13	11	69	27	660	9.54				8	12	8				8	11	8
High	Q9UBU9	Nuclear RNA export factor 1 OS=Homo sapiens OX=9606 GN=NXF1 PE=1 SV=1	60	23	23	70.1	44	619	8.51												
High	Q16576	Histone-binding protein RBBP7 OS=Homo sapiens OX=9606 GN=RBBP7 PE=1 SV=1	60	10	5	47.8	29	425	5.05	5	4	5	7	6	6	5	4	5	6	5	6
High	P08670	Vimentin OS=Homo sapiens OX=9606 GN=VIM PE=1 SV=4	60	10	6	53.6	20	466	5.12	6	7	7	9	10	8	3	3	3	7	6	7
High	P52948	Nuclear pore complex protein Nup98-Nup96 OS=Homo sapiens OX=9606 GN=NUP98 PE=1 SV=4	60	21	21	197.5	16	1817	6.4	4	2	2	2	1	3	4	2	2	2	1	3
High	P09622	Dihydrolipoyl dehydrogenase, mitochondrial OS=Homo sapiens OX=9606 GN=DLD PE=1 SV=2	60	10	10	54.1	28	509	7.85	10	10	11	3	3	3	8	9	8	3	2	3
High	P26196	Probable ATP-dependent RNA helicase DDX6 OS=Homo sapiens OX=9606 GN=DDX6 PE=1 SV=2	59	11	11	54.4	30	483	8.66	5	5	4	5	7	7	5	5	4	5	7	7
High	Q12888	TP53-binding protein 1 OS=Homo sapiens OX=9606 GN=TP53BP1 PE=1 SV=2	59	20	20	213.4	17	1972	4.7	1	4	4	4	7	6	1	4	4	4	7	6
High	P21964	Catechol O-methyltransferase OS=Homo sapiens OX=9606 GN=COMT PE=1 SV=2	59	11	11	30	61	271	5.47	9	8	7	3	4	3	7	5	6	3	4	2
High	Q86XN7	Proline and serine-rich protein 1 OS=Homo sapiens OX=9606 GN=PROSER1 PE=1 SV=2	59	12	12	95.6	16	944	8.88				11	14	9				8	10	8
High	P62753	40S ribosomal protein S6 OS=Homo sapiens OX=9606 GN=RPS6 PE=1 SV=1	59	6	6	28.7	20	249	10.84	5	4	3	6	8	6	4	4	3	4	4	5
High	A5YKK6	CCR4-NOT transcription complex subunit 1 OS=Homo sapiens OX=9606 GN=CNOT1 PE=1 SV=2	59	21	21	266.8	11	2376	7.11	3	5	2	4	5	4	3	5	2	4	5	4
High	Q92900	Regulator of nonsense transcripts 1 OS=Homo sapiens OX=9606 GN=UPF1 PE=1 SV=2	59	13	13	124.3	12	1129	6.61	3	5	4	9	10	9	3	5	4	8	10	9
High	P09497	Clathrin light chain B OS=Homo sapiens OX=9606 GN=CLTB PE=1 SV=1	59	10	10	25.2	28	229	4.64	5	5	5	6	7	7	5	5	5	6	7	7
High	Q9Y5K6	CD2-associated protein OS=Homo sapiens OX=9606 GN=CD2AP PE=1 SV=1	59	15	15	71.4	29	639	6.4	5	6	4	2	5	3	5	6	4	2	4	3
High	Q5JRX3	Presequence protease, mitochondrial OS=Homo sapiens OX=9606 GN=PITRM1 PE=1 SV=3	59	17	17	117.3	21	1037	6.92	5	5	4	5	6	6	5	5	4	4	5	6
High	Q8WVV4	Protein POF1B OS=Homo sapiens OX=9606 GN=POF1B PE=1 SV=3	59	15	15	68	33	589	6.32	9	7	8	3	4	3	9	7	8	3	4	3
High	O43592	Exportin-T OS=Homo sapiens OX=9606 GN=XPOT PE=1 SV=2	59	19	19	109.9	25	962	5.39	15	14	17		2		15	14	16		2	
High	Q9NS37	CREB/ATF bZIP transcription factor OS=Homo sapiens OX=9606 GN=CREBZF PE=1 SV=2	59	14	14	37.1	44	354	5.24				8	5	8				8	5	8
High	Q9BVP2	Guanine nucleotide-binding protein-like 3 OS=Homo sapiens OX=9606 GN=GNL3 PE=1 SV=2	58	14	13	62	27	549	9.16	2	6	3	5	6	6	2	4	2	4	6	6
High	Q99567	Nuclear pore complex protein Nup88 OS=Homo sapiens OX=9606 GN=NUP88 PE=1 SV=2	58	14	14	83.5	27	741	5.69	2	1	3	5	7	4	2	1	3	5	7	4
High	Q3SX09	TREMBL:Q3SX09 (Bos taurus) similar to HBG protein	58	9	9	22	45	201	9.39	5	6	4	9	10	9	5	5	4	5	7	4
High	Q9UE12	TREMBL:Q9UE12 Type I hair keratin 1 - Homo sapiens (Human).	58	3	1	47.2	6	416	4.88	8	9	12	5	8	4	2	2	2	2	2	2
High	Q04637	Eukaryotic translation initiation factor 4 gamma 1 OS=Homo sapiens OX=9606 GN=EIF4G1 PE=1 SV=4	58	18	15	175.4	13	1599	5.33	2	4	4	4	7	5	2	4	4	4	7	5
High	O95861	3'(2'),5'-bisphosphate nucleotidase 1 OS=Homo sapiens OX=9606 GN=BPNT1 PE=1 SV=1	58	11	11	33.4	50	308	5.69	3	1	3	6	6	6	3	1	3	6	5	6
High	P62917	60S ribosomal protein L8 OS=Homo sapiens OX=9606 GN=RPL8 PE=1 SV=2	58	7	7	28	41	257	11.03	5	5	5	5	6	6	4	5	5	5	4	5
High	P26038	Moesin OS=Homo sapiens OX=9606 GN=MSN PE=1 SV=3	58	12	8	67.8	19	577	6.4	4	6	10	4	6	3	4	6	9	4	6	2
High	P30048	Thioredoxin-dependent peroxide reductase, mitochondrial OS=Homo sapiens OX=9606 GN=PRDX3 PE=1 SV=3	58	7	7	27.7	36	256	7.78	6	6	7	6	3	5	6	5	6	5	3	5
High	Q13242	Serine/arginine-rich splicing factor 9 OS=Homo sapiens OX=9606 GN=SRSF9 PE=1 SV=1	58	10	10	25.5	39	221	8.65	5	6	9	6	5	8	5	5	8	5	5	7
High	Q15436	Protein transport protein Sec23A OS=Homo sapiens OX=9606 GN=SEC23A PE=1 SV=2	58	11	8	86.1	18	765	7.08	4	7	10	3	3	4	4	7	10	3	3	4
High	Q9UG63	ATP-binding cassette sub-family F member 2 OS=Homo sapiens OX=9606 GN=ABCF2 PE=1 SV=2	58	13	13	71.2	21	623	7.37	9	7	6	4	5	3	9	7	6	4	5	3
High	Q09161	Nuclear cap-binding protein subunit 1 OS=Homo sapiens OX=9606 GN=NCBP1 PE=1 SV=1	58	13	13	91.8	22	790	6.43	3	4	5	5	8	8	3	4	5	5	8	8
High	P42330	Aldo-keto reductase family 1 member C3 OS=Homo sapiens OX=9606 GN=AKR1C3 PE=1 SV=4	58	10	4	36.8	42	323	7.94	10	11	9	2	3	2	9	9	8	2	3	2
High	Q9Y3F4	Serine-threonine kinase receptor-associated protein OS=Homo sapiens OX=9606 GN=STRAP PE=1 SV=1	58	16	16	38.4	57	350	5.12	2	2	5	7	8	6	2	2	4	7	8	6
High	P51858	Hepatoma-derived growth factor OS=Homo sapiens OX=9606 GN=HDGF PE=1 SV=1	58	13	13	26.8	61	240	4.73	4	5	6	4	6	7	4	5	6	4	6	7
High	P36551	Oxygen-dependent coproporphyrinogen-III oxidase, mitochondrial OS=Homo sapiens OX=9606 GN=CPOX PE=1 SV=3	58	11	11	50.1	29	454	8.25	5	4	6	4	4	4	5	4	6	4	4	4
High	O75400	Pre-mRNA-processing factor 40 homolog A OS=Homo sapiens OX=9606 GN=PRPF40A PE=1 SV=2	58	13	13	108.7	13	957	7.56	2	2	3	9	8	8	2	2	3	9	7	8
High	Q9NZL9	Methionine adenosyltransferase 2 subunit beta OS=Homo sapiens OX=9606 GN=MAT2B PE=1 SV=1	58	10	10	37.5	39	334	7.36	8	9	7	3	6	4	8	9	7	3	5	4
High	P17980	26S proteasome regulatory subunit 6A OS=Homo sapiens OX=9606 GN=PSMC3 PE=1 SV=3	57	11	11	49.2	33	439	5.24	2	2	4	5	8	8	2	2	3	5	7	7
High	P04183	Thymidine kinase, cytosolic OS=Homo sapiens OX=9606 GN=TK1 PE=1 SV=2	57	8	8	25.5	38	234	8.51	2	3	3	5	9	4	2	3	3	5	8	4
High	Q8WUM0	Nuclear pore complex protein Nup133 OS=Homo sapiens OX=9606 GN=NUP133 PE=1 SV=2	57	16	16	128.9	17	1156	5.1	3	5	6	3	3	2	3	5	6	3	3	2
High	O60832	H/ACA ribonucleoprotein complex subunit DKC1 OS=Homo sapiens OX=9606 GN=DKC1 PE=1 SV=3	57	14	14	57.6	29	514	9.42	3	3	5	3	6	4	3	3	5	3	6	4
High	Q12905	Interleukin enhancer-binding factor 2 OS=Homo sapiens OX=9606 GN=ILF2 PE=1 SV=2	57	10	10	43	37	390	5.26	5	4	6	5	4	5	5	4	6	4	3	4
High	P17858	ATP-dependent 6-phosphofructokinase, liver type OS=Homo sapiens OX=9606 GN=PFKL PE=1 SV=6	57	12	7	85	19	780	7.5	6	9	10	2	5	3	5	7	7	2	4	3
High	Q99575	Ribonucleases P/MRP protein subunit POP1 OS=Homo sapiens OX=9606 GN=POP1 PE=1 SV=2	57	17	17	114.6	24	1024	9.22	3	2	2	3	11	4	3	2	2	3	11	4
High	Q9H223	EH domain-containing protein 4 OS=Homo sapiens OX=9606 GN=EHD4 PE=1 SV=1	56	16	15	61.1	32	541	6.76	9	10	10	1		3	9	9	10	1		3
High	O60313	Dynamin-like 120 kDa protein, mitochondrial OS=Homo sapiens OX=9606 GN=OPA1 PE=1 SV=3	56	16	16	111.6	20	960	7.87	3	4	7	1	3	1	3	4	6	1	3	1
High	P18077	60S ribosomal protein L35a OS=Homo sapiens OX=9606 GN=RPL35A PE=1 SV=2	56	6	6	12.5	39	110	11.06	4	3	7	7	6	8	3	2	5	4	4	4
High	O95425	Supervillin OS=Homo sapiens OX=9606 GN=SVIL PE=1 SV=2	56	23	23	247.6	14	2214	6.98	7	10	10		2		7	10	10		2	
High	Q13439	Golgin subfamily A member 4 OS=Homo sapiens OX=9606 GN=GOLGA4 PE=1 SV=1	56	27	27	261	13	2230	5.39				7	11	7				7	11	7
High	P08237	ATP-dependent 6-phosphofructokinase, muscle type OS=Homo sapiens OX=9606 GN=PFKM PE=1 SV=2	56	14	11	85.1	19	780	7.99	6	7	9	4	3	3	6	7	9	4	3	3
High	P36542	ATP synthase subunit gamma, mitochondrial OS=Homo sapiens OX=9606 GN=ATP5F1C PE=1 SV=1	56	9	9	33	32	298	9.22	8	9	10	2	4	3	7	8	9	2	4	3
High	Q13838	Spliceosome RNA helicase DDX39B OS=Homo sapiens OX=9606 GN=DDX39B PE=1 SV=1	56	12	4	49	36	428	5.67	5	5	10	5	5	4	5	5	9	4	5	4
High	P46776	60S ribosomal protein L27a OS=Homo sapiens OX=9606 GN=RPL27A PE=1 SV=2	56	5	5	16.6	28	148	11	6	5	7	5	7	5	4	4	5	4	5	5
High	A6NHL2	Tubulin alpha chain-like 3 OS=Homo sapiens OX=9606 GN=TUBAL3 PE=1 SV=2	55	4	1	49.9	9	446	6.05	6	8	6	5	6	5	4	4	4	3	3	3
High	Q9UPV9	Trafficking kinesin-binding protein 1 OS=Homo sapiens OX=9606 GN=TRAK1 PE=1 SV=1	55	15	15	106	21	953	5.85				12	14	12				12	13	12
High	Q99836	Myeloid differentiation primary response protein MyD88 OS=Homo sapiens OX=9606 GN=MYD88 PE=1 SV=1	55	11	11	33.2	43	296	6.15	5	6	4	7	8	4	5	6	4	7	8	4
High	Q92616	eIF-2-alpha kinase activator GCN1 OS=Homo sapiens OX=9606 GN=GCN1 PE=1 SV=7	55	24	24	292.5	11	2671	7.43	5	5	7	1	1		5	5	7	1	1	
High	Q9HC35	Echinoderm microtubule-associated protein-like 4 OS=Homo sapiens OX=9606 GN=EML4 PE=1 SV=3	55	16	16	108.8	18	981	6.4	1	4	3	6	4	7	1	4	3	6	4	7
High	Q969S8	Polyamine deacetylase HDAC10 OS=Homo sapiens OX=9606 GN=HDAC10 PE=1 SV=1	55	9	9	71.4	24	669	5.74	6	6	7	3	4	4	6	6	5	3	4	4
High	Q13547	Histone deacetylase 1 OS=Homo sapiens OX=9606 GN=HDAC1 PE=1 SV=1	55	10	6	55.1	29	482	5.48	5	4	7	3	7	6	5	4	7	3	4	5
High	P47897	Glutamine--tRNA ligase OS=Homo sapiens OX=9606 GN=QARS1 PE=1 SV=1	55	15	15	87.7	22	775	7.15	6	6	4	2	6	3	6	6	4	2	5	2
High	P00492	Hypoxanthine-guanine phosphoribosyltransferase OS=Homo sapiens OX=9606 GN=HPRT1 PE=1 SV=2	55	8	8	24.6	52	218	6.68	5	4	7	6	8	6	5	4	6	5	6	6
High	Q96DG6	Carboxymethylenebutenolidase homolog OS=Homo sapiens OX=9606 GN=CMBL PE=1 SV=1	55	8	8	28	29	245	7.18	6	4	5	7	5	5	5	4	4	6	5	5
High	Q9UBL6	Copine-7 OS=Homo sapiens OX=9606 GN=CPNE7 PE=1 SV=1	55	11	10	70.2	21	633	6.38	3	4	4	2	6	4	3	4	4	2	4	4
High	Q9NYF8	Bcl-2-associated transcription factor 1 OS=Homo sapiens OX=9606 GN=BCLAF1 PE=1 SV=2	55	11	10	106.1	18	920	9.98	5	5	3	6	9	4	4	5	3	5	8	4
High	O14979	Heterogeneous nuclear ribonucleoprotein D-like OS=Homo sapiens OX=9606 GN=HNRNPDL PE=1 SV=3	55	8	6	46.4	21	420	9.57	8	9	8	5	4	3	7	8	7	4	2	2
High	Q13555	Calcium/calmodulin-dependent protein kinase type II subunit gamma OS=Homo sapiens OX=9606 GN=CAMK2G PE=1 SV=4	55	13	10	62.6	30	558	7.83	9	7	6	3	4	4	9	7	6	3	4	4
High	P69905	Hemoglobin subunit alpha OS=Homo sapiens OX=9606 GN=HBA1 PE=1 SV=2	54	4	2	15.2	25	142	8.68	6	3	4	10	10	8	3	3	3	4	4	4
High	P52943	Cysteine-rich protein 2 OS=Homo sapiens OX=9606 GN=CRIP2 PE=1 SV=1	54	6	6	22.5	53	208	8.72	5	5	6	6	3	5	5	5	5	3	3	3
High	P16989	Y-box-binding protein 3 OS=Homo sapiens OX=9606 GN=YBX3 PE=1 SV=4	54	6	3	40.1	22	372	9.77	3	3	5	7	5	7	3	3	4	6	4	6
High	P60866	40S ribosomal protein S20 OS=Homo sapiens OX=9606 GN=RPS20 PE=1 SV=1	54	5	5	13.4	29	119	9.94	6	6	5	5	6	5	5	4	4	3	4	3
High	Q9Y490	Talin-1 OS=Homo sapiens OX=9606 GN=TLN1 PE=1 SV=3	54	19	17	269.6	10	2541	6.07	3	2	6	3	4	5	3	2	6	3	4	5
High	P30041	Peroxiredoxin-6 OS=Homo sapiens OX=9606 GN=PRDX6 PE=1 SV=3	54	10	10	25	47	224	6.38	7	6	4	6	4	7	7	6	4	6	4	7
High	Q9UKD2	mRNA turnover protein 4 homolog OS=Homo sapiens OX=9606 GN=MRTO4 PE=1 SV=2	54	12	12	27.5	52	239	8.29	2	3	4	6	4	7	2	3	4	6	4	7
High	Q9P206	Uncharacterized protein KIAA1522 OS=Homo sapiens OX=9606 GN=KIAA1522 PE=1 SV=2	54	16	16	107	22	1035	9.73	7	2	4	3	4	3	7	2	4	3	4	3
High	P49902	Cytosolic purine 5'-nucleotidase OS=Homo sapiens OX=9606 GN=NT5C2 PE=1 SV=1	54	11	11	64.9	21	561	6.14	5	7	5	6	5	6	5	7	5	6	5	6
High	P31153	S-adenosylmethionine synthase isoform type-2 OS=Homo sapiens OX=9606 GN=MAT2A PE=1 SV=1	54	9	9	43.6	28	395	6.48	6	7	5	5	6	5	6	7	5	5	6	5
High	P23588	Eukaryotic translation initiation factor 4B OS=Homo sapiens OX=9606 GN=EIF4B PE=1 SV=2	54	7	7	69.1	13	611	5.73	5	5	7	5	6	4	3	4	5	5	6	4
High	P38117	Electron transfer flavoprotein subunit beta OS=Homo sapiens OX=9606 GN=ETFB PE=1 SV=3	54	10	10	27.8	43	255	8.1	7	9	5	5	3	4	7	9	5	5	3	4
High	P46777	60S ribosomal protein L5 OS=Homo sapiens OX=9606 GN=RPL5 PE=1 SV=3	54	10	10	34.3	38	297	9.72	6	7	5	4	3	4	6	7	5	4	3	4
High	Q8TD06	Anterior gradient protein 3 OS=Homo sapiens OX=9606 GN=AGR3 PE=1 SV=1	54	2	1	19.2	13	166	7.99		1		13	19	14		1		1	1	1
High	Q71UI9	Histone H2A.V OS=Homo sapiens OX=9606 GN=H2AZ2 PE=1 SV=3	53	4	2	13.5	31	128	10.58	6	7	9	6	5	5	4	4	3	2	3	2
High	Q9UKV8	Protein argonaute-2 OS=Homo sapiens OX=9606 GN=AGO2 PE=1 SV=3	53	14	14	97.1	20	859	9.19	2	3	1	6	8	3	2	2	1	6	8	3
High	O15226	NF-kappa-B-repressing factor OS=Homo sapiens OX=9606 GN=NKRF PE=1 SV=2	53	13	13	77.6	21	690	8.79	2	1	3	6	8	7	2	1	3	6	8	7
High	O75477	Erlin-1 OS=Homo sapiens OX=9606 GN=ERLIN1 PE=1 SV=2	53	9	6	39.1	27	348	7.87	5	7	7	3	2	2	5	6	6	3	2	2
High	Q16629	Serine/arginine-rich splicing factor 7 OS=Homo sapiens OX=9606 GN=SRSF7 PE=1 SV=1	53	6	5	27.4	24	238	11.82	6	8	6	4	3	3	6	6	5	4	3	3
High	O76021	Ribosomal L1 domain-containing protein 1 OS=Homo sapiens OX=9606 GN=RSL1D1 PE=1 SV=3	53	10	10	54.9	20	490	10.13	3	3	4	5	7	6	3	3	4	5	7	6
High	Q9H9L4	KAT8 regulatory NSL complex subunit 2 OS=Homo sapiens OX=9606 GN=KANSL2 PE=1 SV=3	53	12	12	55	32	492	6.62				7	4	8				6	4	7
High	P63173	60S ribosomal protein L38 OS=Homo sapiens OX=9606 GN=RPL38 PE=1 SV=2	53	4	4	8.2	41	70	10.1	8	10	7	4	5	4	4	4	4	2	3	2
High	Q9UMN6	Histone-lysine N-methyltransferase 2B OS=Homo sapiens OX=9606 GN=KMT2B PE=1 SV=1	53	17	13	293.3	9	2715	8.22				7	8	5				7	8	5
High	Q14847	LIM and SH3 domain protein 1 OS=Homo sapiens OX=9606 GN=LASP1 PE=1 SV=2	53	8	8	29.7	35	261	7.05	4	5	5	4	7	4	4	4	5	4	7	4
High	Q9UKM9	RNA-binding protein Raly OS=Homo sapiens OX=9606 GN=RALY PE=1 SV=1	53	9	9	32.4	31	306	9.17	4	5	5	6	5	7	4	5	5	6	4	7
High	P63167	Dynein light chain 1, cytoplasmic OS=Homo sapiens OX=9606 GN=DYNLL1 PE=1 SV=1	53	5	2	10.4	58	89	7.4	4	5	7	7	6	5	2	2	5	4	3	3
High	O75439	Mitochondrial-processing peptidase subunit beta OS=Homo sapiens OX=9606 GN=PMPCB PE=1 SV=2	53	9	8	54.3	21	489	6.83	5	5	3	7	7	6	5	5	3	6	6	5
High	Q06124	Tyrosine-protein phosphatase non-receptor type 11 OS=Homo sapiens OX=9606 GN=PTPN11 PE=1 SV=3	52	13	13	68	27	593	7.3	1	2	5	7	7	7	1	2	5	7	7	7
High	P22061	Protein-L-isoaspartate(D-aspartate) O-methyltransferase OS=Homo sapiens OX=9606 GN=PCMT1 PE=1 SV=4	52	10	10	24.6	54	227	7.21	7	8	6	3	4	2	7	8	4	3	4	2
High	Q8IVT2	Mitotic interactor and substrate of PLK1 OS=Homo sapiens OX=9606 GN=MISP PE=1 SV=1	52	17	17	75.3	36	679	6.83	4	6	10	2	2	4	4	6	10	2	2	4
High	P32969	60S ribosomal protein L9 OS=Homo sapiens OX=9606 GN=RPL9 PE=1 SV=1	52	6	6	21.9	43	192	9.95	7	8	10	3	2	3	4	4	6	3	2	3
High	P48444	Coatomer subunit delta OS=Homo sapiens OX=9606 GN=ARCN1 PE=1 SV=1	52	13	13	57.2	29	511	6.21	6	8	6	4	4	3	6	8	6	4	4	3
High	P07737	Profilin-1 OS=Homo sapiens OX=9606 GN=PFN1 PE=1 SV=2	52	7	4	15	59	140	8.27	6	7	8	3	3	2	6	6	6	3	3	2
High	P83731	60S ribosomal protein L24 OS=Homo sapiens OX=9606 GN=RPL24 PE=1 SV=1	52	5	5	17.8	31	157	11.25	3	4	6	5	7	6	3	4	5	4	5	5
High	Q9NXV2	BTB/POZ domain-containing protein KCTD5 OS=Homo sapiens OX=9606 GN=KCTD5 PE=1 SV=1	52	8	8	26.1	58	234	6.24	6	5	5	5	4	5	5	4	4	4	3	4
High	Q9Y2H5	Pleckstrin homology domain-containing family A member 6 OS=Homo sapiens OX=9606 GN=PLEKHA6 PE=1 SV=4	52	19	19	117.1	25	1048	9.1	4	3	8	2	2	1	4	3	8	7	2	1
High	O14818	Proteasome subunit alpha type-7 OS=Homo sapiens OX=9606 GN=PSMA7 PE=1 SV=1	52	9	9	27.9	46	248	8.46	4	5	5	8	8	6	4	5	4	7	7	5

High	Q93050	V-type proton ATPase 116 kDa subunit a 1 OS=Homo sapiens OX=9606 GN=ATP6V0A1 PE=1 SV=3	52	14	14	96.4	20	837	6.43	4	4	6				4	4	5			
High	O15144	Actin-related protein 2/3 complex subunit 2 OS=Homo sapiens OX=9606 GN=ARPC2 PE=1 SV=1	52	11	11	34.3	34	300	7.36	7	5	5	4	5	4	7	5	5	4	5	4
High	P26373	60S ribosomal protein L13 OS=Homo sapiens OX=9606 GN=RPL13 PE=1 SV=4	51	6	6	24.2	28	211	11.65	4	5	4	7	6	6	4	5	4	5	5	5
High	Q92828	Coronin-2A OS=Homo sapiens OX=9606 GN=CORO2A PE=1 SV=2	51	10	9	59.7	21	525	8.05	8	7	5	4	3	3	8	6	4	4	3	3
High	Q9HC84	Mucin-5B OS=Homo sapiens OX=9606 GN=MUC5B PE=1 SV=3	51	11	8	596	4	5762	6.64	2	2	1	10	13	11	2	2	1	7	10	8
High	O43293	Death-associated protein kinase 3 OS=Homo sapiens OX=9606 GN=DAPK3 PE=1 SV=1	51	13	13	52.5	36	454	6.89	9	8	10	1	1	2	9	7	9	1	1	2
High	Q96FW1	Ubiquitin thioesterase OTUB1 OS=Homo sapiens OX=9606 GN=OTUB1 PE=1 SV=2	51	8	8	31.3	42	271	4.94	9	5	5	5	4	4	7	5	5	5	4	4
High	Q13162	Peroxiredoxin-4 OS=Homo sapiens OX=9606 GN=PRDX4 PE=1 SV=1	51	4	2	30.5	13	271	6.29	4	4	4	6	10	8	2	2	2	3	4	4
High	O95433	Activator of 90 kDa heat shock protein ATPase homolog 1 OS=Homo sapiens OX=9606 GN=AHSA1 PE=1 SV=1	51	10	10	38.3	37	338	5.53	7	8	8	3	5	3	5	5	7	3	5	3
High	Q9H4A4	Aminopeptidase B OS=Homo sapiens OX=9606 GN=RNPEP PE=1 SV=2	51	14	14	72.5	27	650	5.74	5	4	5	4	6	1	5	4	5	4	6	1
High	Q00325	Phosphate carrier protein, mitochondrial OS=Homo sapiens OX=9606 GN=SLC25A3 PE=1 SV=2	51	8	8	40.1	25	362	9.38	6	8	6	4	5	4	6	7	6	4	5	4
High	Q15717	ELAV-like protein 1 OS=Homo sapiens OX=9606 GN=ELAVL1 PE=1 SV=2	51	8	8	36.1	30	326	9.17	7	5	6	2	4	3	7	5	6	2	4	3
High	Q9BZF9	Uveal autoantigen with coiled-coil domains and ankyrin repeats OS=Homo sapiens OX=9606 GN=UACA PE=1 SV=2	51	19	19	162.4	16	1416	7.03	8	9	10	1	1	1	8	9	10	1	1	1
High	P04632	Calpain small subunit 1 OS=Homo sapiens OX=9606 GN=CAPNS1 PE=1 SV=1	50	8	8	28.3	39	268	5.2	5	5	7	4	5	5	4	4	6	4	5	5
High	Q16777	Histone H2A type 2-C OS=Homo sapiens OX=9606 GN=H2AC20 PE=1 SV=4	50	3	1	14	27	129	10.9	5	6	10	5	4	4	3	3	2	1	2	1
High	Q14683	Structural maintenance of chromosomes protein 1A OS=Homo sapiens OX=9606 GN=SMC1A PE=1 SV=2	50	16	16	143.1	13	1233	7.64	4	3	4	2	2	7	4	3	4	2	2	7
High	Q9H3U1	Protein unc-45 homolog A OS=Homo sapiens OX=9606 GN=UNC45A PE=1 SV=1	50	14	14	103	18	944	6.07	8	6	9	2	2	2	7	6	9	2	2	2
High	Q01844	RNA-binding protein EWS OS=Homo sapiens OX=9606 GN=EWSR1 PE=1 SV=1	50	7	7	68.4	15	656	9.33	7	4	6	2	8	4	6	4	5	2	5	4
High	O43583	Density-regulated protein OS=Homo sapiens OX=9606 GN=DENR PE=1 SV=2	50	7	7	22.1	37	198	5.3	4	3	7	4	4	3	4	3	7	4	4	3
High	P57721	Poly(rC)-binding protein 3 OS=Homo sapiens OX=9606 GN=PCBP3 PE=1 SV=2	50	5	1	39.4	18	371	8.07	6	5	5	5	6	4	4	3	2	3	3	3
High	O95630	STAM-binding protein OS=Homo sapiens OX=9606 GN=STAMBP PE=1 SV=1	50	10	10	48	31	424	6.29	5	6	6		1		5	6	6		1	
High	P84085	ADP-ribosylation factor 5 OS=Homo sapiens OX=9606 GN=ARF5 PE=1 SV=2	50	6	3	20.5	37	180	6.79	7	9	7	3	3	3	5	6	6	3	3	3
High	P62191	26S proteasome regulatory subunit 4 OS=Homo sapiens OX=9606 GN=PSMC1 PE=1 SV=1	50	14	12	49.2	35	440	6.21		1		8	10	9		1		7	9	8
High	P62633	CCHC-type zinc finger nucleic acid binding protein OS=Homo sapiens OX=9606 GN=CNBP PE=1 SV=1	49	6	6	19.5	44	177	7.71	6	5	4	5	7	5	5	4	3	4	5	4
High	Q9UJU6	Drebrin-like protein OS=Homo sapiens OX=9606 GN=DBNL PE=1 SV=1	49	11	11	48.2	34	430	5.05	5	5	6	4	3	4	4	4	6	4	3	4
High	Q9ULC4	Malignant T-cell-amplified sequence 1 OS=Homo sapiens OX=9606 GN=MCTS1 PE=1 SV=1	49	7	7	20.5	44	181	8.82	5	4	3	4	5	5	4	4	3	3	5	4
High	P54868	Hydroxymethylglutaryl-CoA synthase, mitochondrial OS=Homo sapiens OX=9606 GN=HMGCS2 PE=1 SV=1	49	14	13	56.6	33	508	8.16		1		3	5	4		1		3	5	4
High	P36952	Serpin B5 OS=Homo sapiens OX=9606 GN=SERPINB5 PE=1 SV=2	49	10	10	42.1	31	375	6.05	8	7	6	3	5	3	8	7	6	3	5	3
High	P61160	Actin-related protein 2 OS=Homo sapiens OX=9606 GN=ACTR2 PE=1 SV=1	49	9	9	44.7	27	394	6.74	7	6	7	1	5	3	6	5	7	1	5	3
High	Q14376	UDP-glucose 4-epimerase OS=Homo sapiens OX=9606 GN=GALE PE=1 SV=2	49	10	10	38.3	49	348	6.73	4	5	9	2	2	2	4	5	9	2	2	2
High	P61254	60S ribosomal protein L26 OS=Homo sapiens OX=9606 GN=RPL26 PE=1 SV=1	49	5	5	17.2	32	145	10.55	5	5	4	5	7	6	4	4	4	3	5	4
High	P46063	ATP-dependent DNA helicase Q1 OS=Homo sapiens OX=9606 GN=RECQL PE=1 SV=3	49	12	12	73.4	22	649	7.88	4	6	4	3	6	5	4	6	4	3	6	5
High	Q00341	Vigilin OS=Homo sapiens OX=9606 GN=HDLBP PE=1 SV=2	48	12	12	141.4	10	1268	6.87	5	3	4	4	6	3	5	3	4	4	5	3
High	Q8WVV9	Heterogeneous nuclear ribonucleoprotein L-like OS=Homo sapiens OX=9606 GN=HNRNPLL PE=1 SV=1	48	13	12	60	36	542	7.72	6	3	5	1	6	2	6	3	5	1	6	2
High	Q96HC4	PDZ and LIM domain protein 5 OS=Homo sapiens OX=9606 GN=PDLIM5 PE=1 SV=5	48	11	11	63.9	26	596	8.21	3	6	6	3	3	3	3	5	6	3	3	3
High	Q9P2M7	Cingulin OS=Homo sapiens OX=9606 GN=CGN PE=1 SV=3	48	16	16	137	16	1203	5.52	6	8	7	2	1	3	6	8	7	2	1	3
High	O95757	Heat shock 70 kDa protein 4L OS=Homo sapiens OX=9606 GN=HSPA4L PE=1 SV=3	48	15	12	94.5	25	839	5.88	2	3	4	1	5	1	2	3	4	1	4	1
High	Q9HCE1	Helicase MOV-10 OS=Homo sapiens OX=9606 GN=MOV10 PE=1 SV=2	48	14	14	113.6	16	1003	8.82	4	2	2	8	7	7	4	2	2	8	7	6
High	Q96T37	RNA-binding protein 15 OS=Homo sapiens OX=9606 GN=RBM15 PE=1 SV=2	48	9	9	107.1	11	977	10.08	5	3	5	5	3	5	5	3	5	5	3	5
High	Q07020	60S ribosomal protein L18 OS=Homo sapiens OX=9606 GN=RPL18 PE=1 SV=2	48	6	6	21.6	35	188	11.72	2	4	5	5	5	5	2	3	4	5	5	4
High	Q9Y5X1	Sorting nexin-9 OS=Homo sapiens OX=9606 GN=SNX9 PE=1 SV=1	48	10	10	66.6	26	595	5.58	8	8	6	3	5	3	6	6	4	3	5	3
High	Q15185	Prostaglandin E synthase 3 OS=Homo sapiens OX=9606 GN=PTGES3 PE=1 SV=1	48	7	7	18.7	51	160	4.54	7	7	4	3	4	4	6	6	4	3	4	4
High	Q96B97	SH3 domain-containing kinase-binding protein 1 OS=Homo sapiens OX=9606 GN=SH3KBP1 PE=1 SV=2	48	10	10	73.1	17	665	6.62	5	5	5	5	3	1	4	4	4	4	3	1
High	P60891	Ribose-phosphate pyrophosphokinase 1 OS=Homo sapiens OX=9606 GN=PRPS1 PE=1 SV=2	48	8	4	34.8	31	318	6.98	4	6	7	5	4	5	4	6	7	5	4	5
High	O60884	DnaJ homolog subfamily A member 2 OS=Homo sapiens OX=9606 GN=DNAJA2 PE=1 SV=1	48	10	10	45.7	30	412	6.48	6	4	8	5	3	3	6	4	8	5	3	3
High	Q9H0U4	Ras-related protein Rab-1B OS=Homo sapiens OX=9606 GN=RAB1B PE=1 SV=1	48	8	6	22.2	48	201	5.73	7	8	8	2	4	3	5	6	7	2	3	3
High	P62195	26S proteasome regulatory subunit 8 OS=Homo sapiens OX=9606 GN=PSMC5 PE=1 SV=1	48	11	9	45.6	33	406	7.55	1	3	2	6	8	6	1	3	2	5	6	5
High	P22087	rRNA 2'-O-methyltransferase fibrillarin OS=Homo sapiens OX=9606 GN=FBL PE=1 SV=2	48	10	10	33.8	36	321	10.18	6	6	8	1	6	3	6	6	8	1	5	3
High	P04075	Fructose-bisphosphate aldolase A OS=Homo sapiens OX=9606 GN=ALDOA PE=1 SV=2	47	15	14	39.4	41	364	8.09	7	7	11	2	5	4	7	7	9	2	5	4
High	Q9Y6J0	Calcineurin-binding protein cabin-1 OS=Homo sapiens OX=9606 GN=CABIN1 PE=1 SV=1	47	14	14	246.2	10	2220	6.02	6	9	6	2	2	2	6	9	6	2	2	2
High	Q9NVH6	Trimethyllysine dioxygenase, mitochondrial OS=Homo sapiens OX=9606 GN=TMLHE PE=1 SV=1	47	10	10	49.5	24	421	7.72	6	4	5	3	5	5	6	4	5	3	5	5
High	P25786	Proteasome subunit alpha type-1 OS=Homo sapiens OX=9606 GN=PSMA1 PE=1 SV=1	47	9	9	29.5	36	263	6.61	1	3	2	8	8	8	1	3	2	7	7	7
High	P62269	40S ribosomal protein S18 OS=Homo sapiens OX=9606 GN=RPS18 PE=1 SV=3	47	8	8	17.7	48	152	10.99	4	4	9	2	4	4	4	4	7	2	4	3
High	Q9HA64	Ketosamine-3-kinase OS=Homo sapiens OX=9606 GN=FN3KRP PE=1 SV=2	47	8	8	34.4	28	309	7.33	6	5	6	3	5	5	5	5	6	3	4	5
High	Q96QK1	Vacuolar protein sorting-associated protein 35 OS=Homo sapiens OX=9606 GN=VPS35 PE=1 SV=2	47	12	12	91.6	19	796	5.49	6	8	7	2	6	4	6	8	7	2	6	4
High	O60812	Heterogeneous nuclear ribonucleoprotein C-like 1 OS=Homo sapiens OX=9606 GN=HNRNPCL1 PE=1 SV=1	47	6	1	32.1	16	293	5.06	5	5	5	4	4	5	5	4	4	4	4	4
High	Q53H82	Endoribonuclease LACTB2 OS=Homo sapiens OX=9606 GN=LACTB2 PE=1 SV=2	47	10	10	32.8	49	288	6.8	3	3	6	3	4	2	3	3	5	3	4	2
High	P24534	Elongation factor 1-beta OS=Homo sapiens OX=9606 GN=EEF1B2 PE=1 SV=3	47	6	5	24.7	44	225	4.67	4	6	6	2	2	2	4	5	5	2	2	2
High	Q99661	Kinesin-like protein KIF2C OS=Homo sapiens OX=9606 GN=KIF2C PE=1 SV=2	47	13	12	81.3	22	725	7.83	1	1	2	5	5	6	1	1	2	5	5	6
High	P63208	S-phase kinase-associated protein 1 OS=Homo sapiens OX=9606 GN=SKP1 PE=1 SV=2	47	7	7	18.6	51	163	4.54	4	4	4	4	7	4	4	4	4	3	6	4
High	Q9H910	Jupiter microtubule associated homolog 2 OS=Homo sapiens OX=9606 GN=JPT2 PE=1 SV=1	47	8	8	20.1	63	190	9.26	4	4	6	5	5	5	4	4	6	5	5	5
High	Q13492	Phosphatidylinositol-binding clathrin assembly protein OS=Homo sapiens OX=9606 GN=PICALM PE=1 SV=2	47	12	12	70.7	25	652	7.9	6	4	7	2	4	3	6	4	7	2	4	3
High	P84103	Serine/arginine-rich splicing factor 3 OS=Homo sapiens OX=9606 GN=SRSF3 PE=1 SV=1	47	6	5	19.3	35	164	11.65	3	5	4	3	3	4	3	3	3	3	3	4
High	Q9BWF3	RNA-binding protein 4 OS=Homo sapiens OX=9606 GN=RBM4 PE=1 SV=1	47	8	8	40.3	27	364	7.08	1	3	4	5	6	5	1	3	4	5	4	4
High	Q9Y5S2	Serine/threonine-protein kinase MRCK beta OS=Homo sapiens OX=9606 GN=CDC42BPB PE=1 SV=2	47	17	13	194.2	13	1711	6.37	3	5	4				3	5	4			
High	A0FGR8	Extended synaptotagmin-2 OS=Homo sapiens OX=9606 GN=ESYT2 PE=1 SV=1	47	14	14	102.3	21	921	9.26	9	10	10	1	2		9	10	9	1	2	
High	Q8N680	Zinc finger and BTB domain-containing protein 2 OS=Homo sapiens OX=9606 GN=ZBTB2 PE=1 SV=1	47	10	10	57.3	29	514	6.1				6	5	5				6	5	5
High	Q13148	TAR DNA-binding protein 43 OS=Homo sapiens OX=9606 GN=TARDBP PE=1 SV=1	47	8	8	44.7	28	414	6.19	5	5	6	3	4	3	5	5	5	3	4	2
High	O75223	Gamma-glutamylcyclotransferase OS=Homo sapiens OX=9606 GN=GGCT PE=1 SV=1	47	9	9	21	47	188	5.14	1	3	3	8	5	5	1	3	3	7	5	5
High	Q9Y6M1	Insulin-like growth factor 2 mRNA-binding protein 2 OS=Homo sapiens OX=9606 GN=IGF2BP2 PE=1 SV=2	46	10	8	66.1	22	599	8.46	2	2	7	4	4	3	2	2	7	4	4	3
High	Q9NVP1	ATP-dependent RNA helicase DDX18 OS=Homo sapiens OX=9606 GN=DDX18 PE=1 SV=2	46	11	11	75.4	20	670	9.5	5	4	4	2	3	2	5	4	4	2	3	2
High	Q14444	Caprin-1 OS=Homo sapiens OX=9606 GN=CAPRIN1 PE=1 SV=2	46	6	6	78.3	13	709	5.25	4	4	4	5	7	6	4	4	4	5	6	6
High	P43246	DNA mismatch repair protein Msh2 OS=Homo sapiens OX=9606 GN=MSH2 PE=1 SV=1	46	15	15	104.7	17	934	5.77	3	4	4	3	5	4	3	4	4	3	5	4
High	Q14690	Protein RRP5 homolog OS=Homo sapiens OX=9606 GN=PDCD11 PE=1 SV=3	46	17	17	208.6	10	1871	8.87	1		2	3	3	2	1		2	3	3	2
High	P00367	Glutamate dehydrogenase 1, mitochondrial OS=Homo sapiens OX=9606 GN=GLUD1 PE=1 SV=2	46	10	10	61.4	23	558	7.8	5	6	10	3	4	2	5	6	9	3	4	2
High	Q96GQ7	Probable ATP-dependent RNA helicase DDX27 OS=Homo sapiens OX=9606 GN=DDX27 PE=1 SV=2	46	15	15	89.8	20	796	9.28			1	2	8	4			1	2	8	4
High	P35232	Prohibitin 1 OS=Homo sapiens OX=9606 GN=PHB1 PE=1 SV=1	46	10	10	29.8	42	272	5.76	3	3	6	6	1	5	3	3	6	6	1	5
High	P06730	Eukaryotic translation initiation factor 4E OS=Homo sapiens OX=9606 GN=EIF4E PE=1 SV=2	46	8	8	25.1	34	217	6.15	4	4	5	5	4	5	4	3	4	5	4	5
High	O43242	26S proteasome non-ATPase regulatory subunit 3 OS=Homo sapiens OX=9606 GN=PSMD3 PE=1 SV=2	46	13	13	60.9	24	534	8.44				9	10	9				9	10	9
High	Q9BZK7	F-box-like/WD repeat-containing protein TBL1XR1 OS=Homo sapiens OX=9606 GN=TBL1XR1 PE=1 SV=1	46	7	7	55.6	24	514	5.55	3	2	2	5	4	5	3	2	2	5	4	4
High	Q07157	Tight junction protein ZO-1 OS=Homo sapiens OX=9606 GN=TJP1 PE=1 SV=3	46	12	12	195.3	10	1748	6.7	6	5	6	4	5	2	6	5	6	4	5	2
High	Q9BRX2	Protein pelota homolog OS=Homo sapiens OX=9606 GN=PELO PE=1 SV=2	46	10	10	43.3	33	385	6.34	4	4	6	3	7	4	4	3	6	3	7	4
High	Q9Y3B7	39S ribosomal protein L11, mitochondrial OS=Homo sapiens OX=9606 GN=MRPL11 PE=1 SV=1	46	11	11	20.7	54	192	9.91	4	4	2	4	4	5	4	4	2	4	4	5
High	P08708	40S ribosomal protein S17 OS=Homo sapiens OX=9606 GN=RPS17 PE=1 SV=2	46	5	5	15.5	49	135	9.85	7	7	8	1	1	1	5	5	5	1	1	1
High	Q9H0D6	5'-3' exoribonuclease 2 OS=Homo sapiens OX=9606 GN=XRN2 PE=1 SV=1	46	12	12	108.5	16	950	7.47	4	3	4	6	6	4	4	3	4	6	6	4
High	P68036	Ubiquitin-conjugating enzyme E2 L3 OS=Homo sapiens OX=9606 GN=UBE2L3 PE=1 SV=1	46	7	7	17.9	65	154	8.51	5	8	6	2	4	4	4	6	5	2	4	4
High	P58546	Myotrophin OS=Homo sapiens OX=9606 GN=MTPN PE=1 SV=2	45	5	5	12.9	55	118	5.52	5	4	5	4	5	2	4	3	3	3	3	1
High	Q8NE71	ATP-binding cassette sub-family F member 1 OS=Homo sapiens OX=9606 GN=ABCF1 PE=1 SV=2	45	13	13	95.9	21	845	6.8	4	3	1	5	4	4	4	3	1	5	4	4
High	P82650	28S ribosomal protein S22, mitochondrial OS=Homo sapiens OX=9606 GN=MRPS22 PE=1 SV=1	45	9	9	41.3	26	360	7.9	1	1	2	4	7	5	1	1	2	4	7	5
High	Q0VDF9	Heat shock 70 kDa protein 14 OS=Homo sapiens OX=9606 GN=HSPA14 PE=1 SV=1	45	11	11	54.8	28	509	5.59	4	2	8	6	6	4	4	2	7	5	6	4
High	Q9Y3U8	60S ribosomal protein L36 OS=Homo sapiens OX=9606 GN=RPL36 PE=1 SV=3	45	4	4	12.2	30	105	11.59	4	4	4	5	5	5	3	3	3	4	3	4
High	Q92974	Rho guanine nucleotide exchange factor 2 OS=Homo sapiens OX=9606 GN=ARHGEF2 PE=1 SV=4	45	16	16	111.5	18	986	7.27	10	9	8	2		1	10	8	8	2		1
High	Q658Y4	Protein FAM91A1 OS=Homo sapiens OX=9606 GN=FAM91A1 PE=1 SV=3	45	11	11	93.9	16	838	6.39	5	4	5	2	2	2	5	4	5	2	2	2
High	Q9NZT2	Opioid growth factor receptor OS=Homo sapiens OX=9606 GN=OGFR PE=1 SV=3	45	10	10	73.3	21	677	4.84	5	5	4	4	6	4	5	5	4	4	6	4
High	P17096	High mobility group protein HMG-I/HMG-Y OS=Homo sapiens OX=9606 GN=HMGA1 PE=1 SV=3	45	8	8	11.7	41	107	10.32	6	6	10	3	5	2	5	5	7	3	5	2
High	Q14566	DNA replication licensing factor MCM6 OS=Homo sapiens OX=9606 GN=MCM6 PE=1 SV=1	45	16	16	92.8	23	821	5.41	7	13	6	3	3	5	7	13	6	3	3	5
High	Q9H773	dCTP pyrophosphatase 1 OS=Homo sapiens OX=9606 GN=DCTPP1 PE=1 SV=1	45	10	10	18.7	59	170	5.03	4	3	7	3	3	4	4	3	7	3	3	4
High	Q08945	FACT complex subunit SSRP1 OS=Homo sapiens OX=9606 GN=SSRP1 PE=1 SV=1	45	13	13	81	25	709	6.87	6	6	9	1	4	1	5	5	9	1	4	1
High	P31942	Heterogeneous nuclear ribonucleoprotein H3 OS=Homo sapiens OX=9606 GN=HNRNPH3 PE=1 SV=2	45	6	5	36.9	25	346	6.87	8	6	4	3	5	4	6	5	4	3	4	4
High	P62854	40S ribosomal protein S26 OS=Homo sapiens OX=9606 GN=RPS26 PE=1 SV=3	45	4	4	13	44	115	11	2	3	5	3	3	2	2	2	3	2	2	2
High	P53041	Serine/threonine-protein phosphatase 5 OS=Homo sapiens OX=9606 GN=PPP5C PE=1 SV=1	45	10	10	56.8	24	499	6.28	4	3	3	3	5	4	4	3	3	3	5	4
High	P25789	Proteasome subunit alpha type-4 OS=Homo sapiens OX=9606 GN=PSMA4 PE=1 SV=1	45	8	8	29.5	39	261	7.72	5	3	4	4	8	4	3	2	4	4	6	4
High	Q9ULZ3	Apoptosis-associated speck-like protein containing a CARD OS=Homo sapiens OX=9606 GN=PYCARD PE=1 SV=2	44	8	8	21.6	42	195	6.34	5	7	6	3	5	4	5	7	5	3	5	4
High	Q7L5N7	Lysophosphatidylcholine acyltransferase 2 OS=Homo sapiens OX=9606 GN=LPCAT2 PE=1 SV=1	44	10	10	60.2	26	544	6.55	4	5	4	3	3	4	4	5	4	3	3	4
High	Q92734	Protein TFG OS=Homo sapiens OX=9606 GN=TFG PE=1 SV=2	44	8	8	43.4	33	400	5.1	7	8	8	3	3	2	7	8	7	3	3	2
High	P11277	Spectrin beta chain, erythrocytic OS=Homo sapiens OX=9606 GN=SPTB PE=1 SV=5	44	8	1	246.3	5	2137	5.27	8	10	5	3	1	1	5	6	5	3	1	1
High	Q16881	Thioredoxin reductase 1, cytoplasmic OS=Homo sapiens OX=9606 GN=TXNRD1 PE=1 SV=3	44	10	10	70.9	20	649	7.39	2	5	5	5	2	4	2	5	5	5	2	4
High	Q99460	26S proteasome non-ATPase regulatory subunit 1 OS=Homo sapiens OX=9606 GN=PSMD1 PE=1 SV=2	44	12	12	105.8	18	953	5.39				6	9	7				6	8	7
High	Q68CZ2	Tensin-3 OS=Homo sapiens OX=9606 GN=TNS3 PE=1 SV=2	44	7	6	155.2	8	1445	6.81	4	6	6	3	4	2	3	4	5	3	4	2
High	P57740	Nuclear pore complex protein Nup107 OS=Homo sapiens OX=9606 GN=NUP107 PE=1 SV=1	44	13	13	106.3	19	925	5.43	5	2	4	1		1	3	2	4	1		1
High	P60174	Triosephosphate isomerase OS=Homo sapiens OX=9606 GN=TPI1 PE=1 SV=4	44	9	9	26.7	49	249	6.9	7	6	9	3	3	2	6	5	8	3	3	2
High	Q9H6T0	Epithelial splicing regulatory protein 2 OS=Homo sapiens OX=9606 GN=ESRP2 PE=1 SV=1	44	10	8	78.4	17	727	6.71	2	3	5	3	3	3	2	3	4	3	2	3
High	Q15652	Probable JmjC domain-containing histone demethylation protein 2C OS=Homo sapiens OX=9606 GN=JMJD1C PE=1 SV=2	44	16	16	284.3	7	2540	7.87				6	10	7				6	10	7
High	P48047	ATP synthase subunit O, mitochondrial OS=Homo sapiens OX=9606 GN=ATP5PO PE=1 SV=1	44	8	8	23.3	45	213	9.96	4	4	6	5	5	6	4	4	5	5	4	6
High	P20042	Eukaryotic translation initiation factor 2 subunit 2 OS=Homo sapiens OX=9606 GN=EIF2S2 PE=1 SV=2	44	10	10	38.4	35	333	5.8	2	3	3	5	6	4	2	3	3	5	4	4
High	Q99961	Endophilin-A2 OS=Homo sapiens OX=9606 GN=SH3GL1 PE=1 SV=1	43	9	9	41.5	26	368	5.43	3	2	2	4	7	4	3	2	2	4	7	4
High	Q02880	DNA topoisomerase 2-beta OS=Homo sapiens OX=9606 GN=TOP2B PE=1 SV=3	43	10	2	183.2	6	1626	8	6	7	5	2	5	2	6	7	5	2	4	2
High	Q9P0L0	Vesicle-associated membrane protein-associated protein A OS=Homo sapiens OX=9606 GN=VAPA PE=1 SV=3	43	6	5	27.9	37	249	8.62	4	4	6	2	4	2	4	4	4	1	3	2
High	O00291	Huntingtin-interacting protein 1 OS=Homo sapiens OX=9606 GN=HIP1 PE=1 SV=5	43	15	14	116.1	17	1037	5.3	2	2	3		1		2	2	3		1	
High	Q9UKK3	Protein mono-ADP-ribosyltransferase PARP4 OS=Homo sapiens OX=9606 GN=PARP4 PE=1 SV=3	43	16	16	192.5	11	1724	5.66	4	3	4	5	7	3	4	3	4	5	7	3
High	P46734	Dual specificity mitogen-activated protein kinase kinase 3 OS=Homo sapiens OX=9606 GN=MAP2K3 PE=1 SV=2	43	9	9	39.3	31	347	7.43	6	5	5	2	5	4	6	5	4	2	5	4
High	Q9H9H4	Vacuolar protein sorting-associated protein 37B OS=Homo sapiens OX=9606 GN=VPS37B PE=1 SV=1	43	5	5	31.3	25	285	7.34	5	4	3	4	5	4	5	4	3	4	4	4
High	Q02543	60S ribosomal protein L18a OS=Homo sapiens OX=9606 GN=RPL18A PE=1 SV=2	43	5	5	20.7	30	176	10.71	3	3	4	3	3	4	3	3	4	2	2	2
High	O75362	Zinc finger protein 217 OS=Homo sapiens OX=9606 GN=ZNF217 PE=1 SV=1	43	21	21	115.2	31	1048	8.48						1						1
High	O15355	Protein phosphatase 1G OS=Homo sapiens OX=9606 GN=PPM1G PE=1 SV=1	43	10	10	59.2	27	546	4.36			2	4	5	6			2	4	5	6
High	P62910	60S ribosomal protein L32 OS=Homo sapiens OX=9606 GN=RPL32 PE=1 SV=2	43	6	6	15.9	39	135	11.33	5	4	3	3	3	4	4	3	2	2	2	3
High	Q96H79	Zinc finger CCCH-type antiviral protein 1-like OS=Homo sapiens OX=9606 GN=ZC3HAV1L PE=1 SV=2	43	6	6	32.9	29	300	8.13	3	3	7	3	4	5	3	3	6	2	4	4
High	P25398	40S ribosomal protein S12 OS=Homo sapiens OX=9606 GN=RPS12 PE=1 SV=3	43	6	6	14.5	53	132	7.21	5	5	6	4	4	3	5	5	6	4	4	3
High	P56545	C-terminal-binding protein 2 OS=Homo sapiens OX=9606 GN=CTBP2 PE=1 SV=1	43	8	6	48.9	22	445	6.95	3	4	4	4	3	3	3	4	4	4	3	3
High	P12268	Inosine-5'-monophosphate dehydrogenase 2 OS=Homo sapiens OX=9606 GN=IMPDH2 PE=1 SV=2	43	8	7	55.8	18	514	6.9	3	2	4	2	4	4	3	2	4	2	4	4
High	P19525	Interferon-induced, double-stranded RNA-activated protein kinase OS=Homo sapiens OX=9606 GN=EIF2AK2 PE=1 SV=2	43	9	9	62.1	19	551	8.4	1	3	1	5	6	7	1	3	1	5	6	7
High	Q12792	Twinfilin-1 OS=Homo sapiens OX=9606 GN=TWF1 PE=1 SV=3	42	8	7	40.3	21	350	6.96	5	6	5	3	3	5	4	4	4	3	3	5
High	O14976	Cyclin-G-associated kinase OS=Homo sapiens OX=9606 GN=GAK PE=1 SV=2	42	17	17	143.1	17	1311	5.73	3	3	2		2	1	3	3	2		2	1
High	P19367	Hexokinase-1 OS=Homo sapiens OX=9606 GN=HK1 PE=1 SV=3	42	14	9	102.4	15	917	6.8	6	9	5	1	1		6	9	5	1	1	
High	Q9H0A0	RNA cytidine acetyltransferase OS=Homo sapiens OX=9606 GN=NAT10 PE=1 SV=2	42	12	12	115.7	14	1025	8.27	1	2	4	4	5	2	1	2	4	4	5	2
High	P29353	SHC-transforming protein 1 OS=Homo sapiens OX=9606 GN=SHC1 PE=1 SV=4	42	10	10	62.8	26	583	6.44	2		3	5	4	6	2		3	5	4	6
High	O75179	Ankyrin repeat domain-containing protein 17 OS=Homo sapiens OX=9606 GN=ANKRD17 PE=1 SV=3	42	13	7	274.1	7	2603	6.52				7	8	9				7	8	9
High	Q04828	Aldo-keto reductase family 1 member C1 OS=Homo sapiens OX=9606 GN=AKR1C1 PE=1 SV=1	42	7	1	36.8	25	323	7.88	7	7	6	2	3	2	6	6	5	2	3	2
High	P04844	Dolichyl-diphosphooligosaccharide--protein glycosyltransferase subunit 2 OS=Homo sapiens OX=9606 GN=RPN2 PE=1 SV=3	42	12	12	69.2	30	631	5.69	8	9	3	3	2	1	8	9	3	3	2	1
High	P08240	Signal recognition particle receptor subunit alpha OS=Homo sapiens OX=9606 GN=SRPRA PE=1 SV=2	42	11	11	69.8	20	638	8.95	6	7	7	2	1	1	6	7	7	2	1	1
High	Q5H9F3	BCL-6 corepressor-like protein 1 OS=Homo sapiens OX=9606 GN=BCORL1 PE=1 SV=2	42	14	13	190.4	10	1785	7.85				8	8	7				8	8	7
High	Q9Y4E8	Ubiquitin carboxyl-terminal hydrolase 15 OS=Homo sapiens OX=9606 GN=USP15 PE=1 SV=3	42	12	12	112.3	14	981	5.22	5	2	3	4	7	4	5	2	3	4	7	4
High	Q9BR76	Coronin-1B OS=Homo sapiens OX=9606 GN=CORO1B PE=1 SV=1	42	9	9	54.2	20	489	5.88	4	5	5	3	4	6	4	5	4	3	4	6
High	O75531	Barrier-to-autointegration factor OS=Homo sapiens OX=9606 GN=BANF1 PE=1 SV=1	42	4	4	10.1	51	89	6.09	10	9	11	3	1	2	4	4	4	1	1	1
High	P37108	Signal recognition particle 14 kDa protein OS=Homo sapiens OX=9606 GN=SRP14 PE=1 SV=2	42	5	5	14.6	30	136	10.04	4	4	2	3	6	4	3	3	2	3	4	4
High	O95487	Protein transport protein Sec24B OS=Homo sapiens OX=9606 GN=SEC24B PE=1 SV=2	42	11	9	137.3	10	1268	6.67			1	3	5	3			1	3	5	3
High	Q9UNM6	26S proteasome non-ATPase regulatory subunit 13 OS=Homo sapiens OX=9606 GN=PSMD13 PE=1 SV=2	42	11	11	42.9	29	376	5.81	1		1	6	10	6	1		1	6	10	6
High	A0AVT1	Ubiquitin-like modifier-activating enzyme 6 OS=Homo sapiens OX=9606 GN=UBA6 PE=1 SV=1	42	14	14	117.9	15	1052	6.14	5	5	3	2	2	4	5	5	3	2	2	4
High	Q6P2Q9	Pre-mRNA-processing-splicing factor 8 OS=Homo sapiens OX=9606 GN=PRPF8 PE=1 SV=2	42	18	18	273.4	9	2335	8.84	2	3	2	3	5	2	2	3	2	3	5	2
High	Q06787	Synaptic functional regulator FMR1 OS=Homo sapiens OX=9606 GN=FMR1 PE=1 SV=1	42	8	6	71.1	15	632	7.42	5	4	2	4	6	4	5	4	2	4	6	4
High	Q8NFJ5	Retinoic acid-induced protein 3 OS=Homo sapiens OX=9606 GN=GPRC5A PE=1 SV=2	42	5	5	40.2	14	357	8.15	5	5	7	1	3	1	4	4	5	1	3	1
High	Q9Y2T2	AP-3 complex subunit mu-1 OS=Homo sapiens OX=9606 GN=AP3M1 PE=1 SV=1	42	10	8	46.9	35	418	6.93	6	5	6	3	3	3	6	5	6	3	3	3
High	P80188	Neutrophil gelatinase-associated lipocalin OS=Homo sapiens OX=9606 GN=LCN2 PE=1 SV=2	41	6	6	22.6	40	198	8.91	4	3	4	2	4	3	3	2	3	1	3	2
High	Q71UM5	40S ribosomal protein S27-like OS=Homo sapiens OX=9606 GN=RPS27L PE=1 SV=3	41	5	2	9.5	40	84	9.45	4	5	3	2	3	2	4	4	2	2	3	2
High	P61962	DDB1- and CUL4-associated factor 7 OS=Homo sapiens OX=9606 GN=DCAF7 PE=1 SV=1	41	9	9	38.9	30	342	5.52	1	2	3	5	6	4	1	2	3	4	6	4
High	P06493	Cyclin-dependent kinase 1 OS=Homo sapiens OX=9606 GN=CDK1 PE=1 SV=3	41	7	5	34.1	26	297	8.4	4	5	6	4	3	5	4	4	5	3	2	5
High	P34897	Serine hydroxymethyltransferase, mitochondrial OS=Homo sapiens OX=9606 GN=SHMT2 PE=1 SV=3	41	12	11	56	27	504	8.53	4	7	8	1	2	2	3	7	7	1	2	2
High	P51148	Ras-related protein Rab-5C OS=Homo sapiens OX=9606 GN=RAB5C PE=1 SV=2	41	7	4	23.5	39	216	8.41	4	6	6	2	4	2	4	6	6	2	4	2
High	Q7KZI7	Serine/threonine-protein kinase MARK2 OS=Homo sapiens OX=9606 GN=MARK2 PE=1 SV=2	41	11	8	87.9	18	788	9.72	5	4	5	1	1	1	5	4	5	1	1	1
High	O95816	BAG family molecular chaperone regulator 2 OS=Homo sapiens OX=9606 GN=BAG2 PE=1 SV=1	41	8	8	23.8	51	211	6.7	5	4	5	4	3	2	4	4	4	4	3	2
High	Q9H0W9	Ester hydrolase C11orf54 OS=Homo sapiens OX=9606 GN=C11orf54 PE=1 SV=1	41	7	7	35.1	30	315	6.7	5	6	4	2	3	3	5	6	4	2	3	3
High	O14579	Coatomer subunit epsilon OS=Homo sapiens OX=9606 GN=COPE PE=1 SV=3	41	7	7	34.5	27	308	5.12	5	5	4	1	1		5	5	4	1	1	
High	Q9NSE4	Isoleucine--tRNA ligase, mitochondrial OS=Homo sapiens OX=9606 GN=IARS2 PE=1 SV=2	41	10	10	113.7	12	1012	7.2	10	9	8	1	1	1	9	9	8	1	1	1
High	O00299	Chloride intracellular channel protein 1 OS=Homo sapiens OX=9606 GN=CLIC1 PE=1 SV=4	41	10	10	26.9	61	241	5.17	4	6	5	2	4	2	3	5	5	2	4	2
High	P33316	Deoxyuridine 5'-triphosphate nucleotidohydrolase, mitochondrial OS=Homo sapiens OX=9606 GN=DUT PE=1 SV=4	41	6	6	26.5	31	252	9.36	5	5	4	3	6	4	4	4	4	3	5	4
High	P60900	Proteasome subunit alpha type-6 OS=Homo sapiens OX=9606 GN=PSMA6 PE=1 SV=1	41	8	8	27.4	36	246	6.76	3	1	1	6	11	6	3	1	1	5	8	5
High	P22695	Cytochrome b-c1 complex subunit 2, mitochondrial OS=Homo sapiens OX=9606 GN=UQCRC2 PE=1 SV=3	41	9	9	48.4	29	453	8.63	6	6	6	2	3	2	5	5	6	2	2	2
High	P35573	Glycogen debranching enzyme OS=Homo sapiens OX=9606 GN=AGL PE=1 SV=3	41	11	11	174.7	9	1532	6.76	2	1	2	3	5	3	2	1	2	3	5	3
High	P46778	60S ribosomal protein L21 OS=Homo sapiens OX=9606 GN=RPL21 PE=1 SV=2	41	6	6	18.6	34	160	10.49	2	5	3	4	4	5	2	4	2	3	3	5

High	Q5VWN6	Protein TASOR 2 OS=Homo sapiens OX=9606 GN=TASOR2 PE=1 SV=1	41	16	16	268.7	8	2430	5.9				5	6	7				5	6	7
High	Q9UEW8	STE20/SPS1-related proline-alanine-rich protein kinase OS=Homo sapiens OX=9606 GN=STK39 PE=1 SV=3	41	11	7	59.4	26	545	6.29	6	5	5	2	3	3	6	5	5	2	3	3
High	O14744	Protein arginine N-methyltransferase 5 OS=Homo sapiens OX=9606 GN=PRMT5 PE=1 SV=4	41	12	12	72.6	22	637	6.29	4	2	2	3	6	5	4	2	2	3	6	4
High	P12694	2-oxoisovalerate dehydrogenase subunit alpha, mitochondrial OS=Homo sapiens OX=9606 GN=BCKDHA PE=1 SV=2	41	9	9	50.4	25	445	8.27	6	5	6	2	3	4	5	4	6	2	3	4
High	Q9P2R7	Succinate--CoA ligase [ADP-forming] subunit beta, mitochondrial OS=Homo sapiens OX=9606 GN=SUCLA2 PE=1 SV=3	41	9	9	50.3	18	463	7.42	5	7	7	2	5	2	5	5	7	2	5	2
High	P63000	Ras-related C3 botulinum toxin substrate 1 OS=Homo sapiens OX=9606 GN=RAC1 PE=1 SV=1	41	6	5	21.4	33	192	8.5	3	5	4	3	4	3	3	4	3	3	4	3
High	P35659	Protein DEK OS=Homo sapiens OX=9606 GN=DEK PE=1 SV=1	41	10	10	42.6	33	375	8.56				5	7	6				5	6	6
High	P23193	Transcription elongation factor A protein 1 OS=Homo sapiens OX=9606 GN=TCEA1 PE=1 SV=2	41	9	9	33.9	35	301	8.38	1	1	4	4	4	5	1	1	4	4	4	5
High	Q9UHD8	Septin-9 OS=Homo sapiens OX=9606 GN=SEPTIN9 PE=1 SV=2	41	10	10	65.4	21	586	8.97	5	4	7	3	1	2	5	4	7	3	1	2
High	P15927	Replication protein A 32 kDa subunit OS=Homo sapiens OX=9606 GN=RPA2 PE=1 SV=1	41	7	7	29.2	46	270	6.15	6	6	8	4	4	3	5	5	6	4	4	3
High	Q96QV6	Histone H2A type 1-A OS=Homo sapiens OX=9606 GN=H2AC1 PE=1 SV=3	40	3	1	14.2	30	131	10.86	5	5	7	5	4	4	3	3	2	1	2	1
High	P55212	Caspase-6 OS=Homo sapiens OX=9606 GN=CASP6 PE=1 SV=2	40	8	7	33.3	29	293	6.93	2	4	6	4	4	5	2	4	6	4	4	5
High	Q8NFW8	N-acylneuraminate cytidylyltransferase OS=Homo sapiens OX=9606 GN=CMAS PE=1 SV=2	40	11	11	48.3	30	434	7.93	2	2	3	3	6	2	2	2	3	3	6	2
High	Q7Z3B4	Nucleoporin p54 OS=Homo sapiens OX=9606 GN=NUP54 PE=1 SV=2	40	10	10	55.4	22	507	7.02				3	7	5				3	7	4
High	P14621	Acylphosphatase-2 OS=Homo sapiens OX=9606 GN=ACYP2 PE=1 SV=2	40	5	5	11.1	46	99	9.5	3	4	5	5	4	4	3	4	5	5	4	4
High	Q96I24	Far upstream element-binding protein 3 OS=Homo sapiens OX=9606 GN=FUBP3 PE=1 SV=2	40	10	9	61.6	27	572	8.38	3	4	5	1	1	3	3	4	5	1	1	3
High	Q9BZL6	Serine/threonine-protein kinase D2 OS=Homo sapiens OX=9606 GN=PRKD2 PE=1 SV=3	40	12	12	96.7	19	878	6.84	6	7	9	1	2	2	6	6	9	1	2	2
High	P62333	26S proteasome regulatory subunit 10B OS=Homo sapiens OX=9606 GN=PSMC6 PE=1 SV=1	40	10	10	44.1	31	389	7.49	2	2	2	4	6	6	2	2	2	4	6	6
High	Q9NP81	Serine--tRNA ligase, mitochondrial OS=Homo sapiens OX=9606 GN=SARS2 PE=1 SV=1	40	11	11	58.2	31	518	8.13	2	2	3	3	2	3	2	2	3	3	2	3
High	O75746	Calcium-binding mitochondrial carrier protein Aralar1 OS=Homo sapiens OX=9606 GN=SLC25A12 PE=1 SV=2	40	10	5	74.7	16	678	8.38	8	8	8	2	3	2	8	8	8	2	3	2
High	Q9Y2T7	Y-box-binding protein 2 OS=Homo sapiens OX=9606 GN=YBX2 PE=1 SV=2	40	4	1	38.5	14	364	10.8	2	2	4	5	3	5	2	2	3	4	2	4
High	Q9UBX3	Mitochondrial dicarboxylate carrier OS=Homo sapiens OX=9606 GN=SLC25A10 PE=1 SV=2	40	9	9	31.3	34	287	9.54	8	5	9	1	3	1	6	4	7	1	3	1
High	Q9UQB8	Brain-specific angiogenesis inhibitor 1-associated protein 2 OS=Homo sapiens OX=9606 GN=BAIAP2 PE=1 SV=1	40	11	11	60.8	23	552	8.9	3	5	5		1	1	3	5	5		1	1
High	P48506	Glutamate--cysteine ligase catalytic subunit OS=Homo sapiens OX=9606 GN=GCLC PE=1 SV=2	40	10	10	72.7	19	637	6.09	1	3	2	3	5	2	1	3	2	3	5	2
High	Q9Y314	Nitric oxide synthase-interacting protein OS=Homo sapiens OX=9606 GN=NOSIP PE=1 SV=1	40	8	8	33.2	36	301	8.82	1	2	2	7	5	6	1	2	2	6	5	6
High	Q9H845	Complex I assembly factor ACAD9, mitochondrial OS=Homo sapiens OX=9606 GN=ACAD9 PE=1 SV=1	40	14	14	68.7	23	621	7.96	4	5	6	1	2		4	5	6	1	2	
High	O75083	WD repeat-containing protein 1 OS=Homo sapiens OX=9606 GN=WDR1 PE=1 SV=4	40	9	9	66.2	21	606	6.65	5	6	6	1	2	2	5	6	6	1	2	2
High	Q7KZF4	Staphylococcal nuclease domain-containing protein 1 OS=Homo sapiens OX=9606 GN=SND1 PE=1 SV=1	40	15	15	101.9	20	910	7.17	4	3	3	1	3	2	4	3	3	1	3	2
High	P36969	Phospholipid hydroperoxide glutathione peroxidase OS=Homo sapiens OX=9606 GN=GPX4 PE=1 SV=3	40	9	9	22.2	44	197	8.37	3	3	2	3	4	3	3	3	2	3	4	3
High	O00763	Acetyl-CoA carboxylase 2 OS=Homo sapiens OX=9606 GN=ACACB PE=1 SV=3	40	8	1	276.4	4	2458	6.49	5	5	4	4	2	4	5	5	4	4	2	4
High	Q86X55	Histone-arginine methyltransferase CARM1 OS=Homo sapiens OX=9606 GN=CARM1 PE=1 SV=3	39	9	9	65.8	17	608	6.73	3	3	3	4	5	4	3	3	3	4	5	4
High	P09012	U1 small nuclear ribonucleoprotein A OS=Homo sapiens OX=9606 GN=SNRPA PE=1 SV=3	39	6	4	31.3	27	282	9.83	4	4	3	4	4	5	4	4	3	4	3	5
High	Q86X29	Lipolysis-stimulated lipoprotein receptor OS=Homo sapiens OX=9606 GN=LSR PE=1 SV=4	39	9	9	71.4	18	649	7.97	3	3	7	1	1	1	3	3	7	1	1	1
High	Q13151	Heterogeneous nuclear ribonucleoprotein A0 OS=Homo sapiens OX=9606 GN=HNRNPA0 PE=1 SV=1	39	8	7	30.8	31	305	9.29	6	8	8	2	1	1	5	7	6	2	1	1
High	Q53H96	Pyrroline-5-carboxylate reductase 3 OS=Homo sapiens OX=9606 GN=PYCR3 PE=1 SV=3	39	7	7	28.6	36	274	7.72	5	3	3	3	3	3	4	3	2	3	3	3
High	Q99829	Copine-1 OS=Homo sapiens OX=9606 GN=CPNE1 PE=1 SV=1	39	9	9	59	17	537	5.83	5	5	4	3	2	4	5	5	4	3	2	4
High	Q9GZP4	PITH domain-containing protein 1 OS=Homo sapiens OX=9606 GN=PITHD1 PE=1 SV=1	39	8	8	24.2	52	211	5.74	4	5	6	2	3	3	4	5	5	2	3	3
High	P06744	Glucose-6-phosphate isomerase OS=Homo sapiens OX=9606 GN=GPI PE=1 SV=4	39	10	10	63.1	22	558	8.32	7	5	9				6	5	8			
High	P16401	Histone H1.5 OS=Homo sapiens OX=9606 GN=H1-5 PE=1 SV=3	39	4	4	22.6	15	226	10.92	1	4	4	3	6	5	1	3	3	2	3	4
High	O75608	Acyl-protein thioesterase 1 OS=Homo sapiens OX=9606 GN=LYPLA1 PE=1 SV=1	39	5	5	24.7	28	230	6.77	4	4	5	3	4	4	4	4	5	3	4	4
High	P08195	4F2 cell-surface antigen heavy chain OS=Homo sapiens OX=9606 GN=SLC3A2 PE=1 SV=3	39	10	10	68	22	630	5.01	7	3	6	4	5	4	7	3	6	4	4	4
High	P41091	Eukaryotic translation initiation factor 2 subunit 3 OS=Homo sapiens OX=9606 GN=EIF2S3 PE=1 SV=3	39	8	8	51.1	18	472	8.4	3	2	2	3	6	5	3	2	2	3	6	4
High	Q56VL3	OCIA domain-containing protein 2 OS=Homo sapiens OX=9606 GN=OCIAD2 PE=1 SV=1	39	6	6	16.9	38	154	9.03	6	5	4	3	4	3	5	5	4	3	4	3
High	Q9UPN3	Microtubule-actin cross-linking factor 1, isoforms 1/2/3/5 OS=Homo sapiens OX=9606 GN=MACF1 PE=1 SV=4	39	13	12	837.8	2	7388	5.39	3	5	4		2	1	3	5	4		2	1
High	O60296	Trafficking kinesin-binding protein 2 OS=Homo sapiens OX=9606 GN=TRAK2 PE=1 SV=2	39	17	17	101.4	26	914	5.24				9	14	10				9	13	10
High	Q9HD42	Charged multivesicular body protein 1a OS=Homo sapiens OX=9606 GN=CHMP1A PE=1 SV=1	39	6	6	21.7	25	196	8.06	5	4	6	3	3	3	5	4	5	3	3	3
High	O95758	Polypyrimidine tract-binding protein 3 OS=Homo sapiens OX=9606 GN=PTBP3 PE=1 SV=2	39	7	4	59.7	16	552	9.04	3	4	4	4	5	5	3	4	4	3	3	3
High	Q9BXP5	Serrate RNA effector molecule homolog OS=Homo sapiens OX=9606 GN=SRRT PE=1 SV=1	39	10	10	100.6	15	876	5.96	3	2	5	3	4	4	3	2	5	3	4	4
High	Q96IZ0	PRKC apoptosis WT1 regulator protein OS=Homo sapiens OX=9606 GN=PAWR PE=1 SV=1	39	8	8	36.5	43	340	5.41	5	4	7	1	4	1	5	4	5	1	4	1
High	P53814	Smoothelin OS=Homo sapiens OX=9606 GN=SMTN PE=1 SV=7	39	9	9	99	11	917	9.07	4	3	3	3	4	2	4	3	3	3	4	2
High	P01966	SWISS-PROT:P01966 (Bos taurus) Hemoglobin subunit alpha	38	3	1	15.2	17	142	8.44	1	2	5	5	7	9	1	2	2	3	3	3
High	Q96BW5	Phosphotriesterase-related protein OS=Homo sapiens OX=9606 GN=PTER PE=1 SV=1	38	8	8	39	24	349	6.52	4	2	4	6	3	6	4	2	4	6	3	6
High	Q9Y3Z3	Deoxynucleoside triphosphate triphosphohydrolase SAMHD1 OS=Homo sapiens OX=9606 GN=SAMHD1 PE=1 SV=2	38	13	13	72.2	23	626	7.14	2	3	1	8	11	7	2	3	1	8	11	7
High	P49207	60S ribosomal protein L34 OS=Homo sapiens OX=9606 GN=RPL34 PE=1 SV=3	38	6	6	13.3	37	117	11.47	3	3	6	3	3	3	3	3	5	3	3	3
High	O60610	Protein diaphanous homolog 1 OS=Homo sapiens OX=9606 GN=DIAPH1 PE=1 SV=2	38	12	12	141.3	11	1272	5.41	1	3	5		3	1	1	3	5		3	1
High	O14524	Nuclear envelope integral membrane protein 1 OS=Homo sapiens OX=9606 GN=NEMP1 PE=1 SV=2	38	10	10	50.6	19	444	6.93					1	1					1	1
High	O95395	Beta-1,3-galactosyl-O-glycosyl-glycoprotein beta-1,6-N-acetylglucosaminyltransferase 3 OS=Homo sapiens OX=9606 GN=GCNT3 P	38	11	11	50.8	33	438	8.25	11	10	10				11	10	10			
High	Q4G176	Malonate--CoA ligase ACSF3, mitochondrial OS=Homo sapiens OX=9606 GN=ACSF3 PE=1 SV=3	38	10	10	64.1	22	576	8.37	3		2	4	5	3	3		2	4	5	3
High	Q14166	Tubulin--tyrosine ligase-like protein 12 OS=Homo sapiens OX=9606 GN=TTLL12 PE=1 SV=2	38	9	9	74.4	18	644	5.53	2	3	5	4	3	4	2	3	5	4	3	4
High	O15014	Zinc finger protein 609 OS=Homo sapiens OX=9606 GN=ZNF609 PE=1 SV=2	38	10	10	151.1	11	1411	8.03	2	2	3	3	6	3	2	2	3	3	6	3
High	Q12933	TNF receptor-associated factor 2 OS=Homo sapiens OX=9606 GN=TRAF2 PE=1 SV=2	38	10	10	55.8	23	501	7.53	4	3	4	2	3	1	4	3	4	2	3	1
High	Q96FJ2	Dynein light chain 2, cytoplasmic OS=Homo sapiens OX=9606 GN=DYNLL2 PE=1 SV=1	38	5	2	10.3	58	89	7.37	4	5	5	2	3	2	2	2	3	2	2	2
High	O15269	Serine palmitoyltransferase 1 OS=Homo sapiens OX=9606 GN=SPTLC1 PE=1 SV=1	38	9	9	52.7	25	473	6.01	5	5	4	2	1	1	5	5	4	2	1	1
High	Q9Y6Y0	Influenza virus NS1A-binding protein OS=Homo sapiens OX=9606 GN=IVNS1ABP PE=1 SV=3	38	10	10	71.7	17	642	5.53			1	6	6	6			1	6	6	6
High	P49916	DNA ligase 3 OS=Homo sapiens OX=9606 GN=LIG3 PE=1 SV=2	38	13	13	112.8	15	1009	9.01	2	1	2	2	3		1	1	2	2	3	
High	Q16630	Cleavage and polyadenylation specificity factor subunit 6 OS=Homo sapiens OX=9606 GN=CPSF6 PE=1 SV=2	38	8	8	59.2	17	551	7.15	2	3	3	5	5	4	2	3	2	5	5	4
High	P25189	Myelin protein P0 OS=Homo sapiens OX=9606 GN=MPZ PE=1 SV=1	38	6	6	27.5	25	248	9.54				11	15	12				6	6	6
High	Q15646	2'-5'-oligoadenylate synthase-like protein OS=Homo sapiens OX=9606 GN=OASL PE=1 SV=2	38	9	9	59.2	23	514	7.87	2	3	3	3	4	3	2	3	3	3	4	3
High	Q01130	Serine/arginine-rich splicing factor 2 OS=Homo sapiens OX=9606 GN=SRSF2 PE=1 SV=4	38	5	5	25.5	19	221	11.85	3	3	4	3	3	4	3	3	3	3	3	4
High	Q13185	Chromobox protein homolog 3 OS=Homo sapiens OX=9606 GN=CBX3 PE=1 SV=4	37	5	4	20.8	29	183	5.33	5	5	4	3	4	3	4	4	4	3	4	3
High	A0A0B4J2	Putative glutamine amidotransferase-like class 1 domain-containing protein 3B, mitochondrial OS=Homo sapiens OX=9606 GN=G	37	9	9	28.1	56	268	8.27	4	4	4	2	6	2	3	3	4	2	4	2
High	Q99729	Heterogeneous nuclear ribonucleoprotein A/B OS=Homo sapiens OX=9606 GN=HNRNPAB PE=1 SV=2	37	6	5	36.2	21	332	8.21	6	5	5	3	3	3	5	4	4	2	2	2
High	P51532	Transcription activator BRG1 OS=Homo sapiens OX=9606 GN=SMARCA4 PE=1 SV=2	37	12	9	184.5	8	1647	7.88	1	2	4	3	3	4	1	2	4	3	3	4
High	O00629	Importin subunit alpha-3 OS=Homo sapiens OX=9606 GN=KPNA4 PE=1 SV=1	37	9	6	57.9	27	521	4.96		2	3	3	3	2		2	3	3	3	2
High	Q16531	DNA damage-binding protein 1 OS=Homo sapiens OX=9606 GN=DDB1 PE=1 SV=1	37	15	15	126.9	14	1140	5.26	1	3	1	8	5	8	1	3	1	8	5	8
High	P04626	Receptor tyrosine-protein kinase erbB-2 OS=Homo sapiens OX=9606 GN=ERBB2 PE=1 SV=1	37	14	13	137.8	15	1255	5.91	9	10	9				8	9	9			
High	Q92888	Rho guanine nucleotide exchange factor 1 OS=Homo sapiens OX=9606 GN=ARHGEF1 PE=1 SV=2	37	10	10	102.4	14	912	5.66	6	5	6	1	1	1	6	5	6	1	1	1
High	P42677	40S ribosomal protein S27 OS=Homo sapiens OX=9606 GN=RPS27 PE=1 SV=3	37	4	1	9.5	39	84	9.45	2	4	1	4	4	4	2	3	1	3	3	3
High	Q14694	Ubiquitin carboxyl-terminal hydrolase 10 OS=Homo sapiens OX=9606 GN=USP10 PE=1 SV=2	37	11	11	87.1	20	798	5.31	3	1	2	2	7	2	3	1	2	2	7	2
High	P46779	60S ribosomal protein L28 OS=Homo sapiens OX=9606 GN=RPL28 PE=1 SV=3	37	4	4	15.7	28	137	12.02	4	3	4	4	4	3	3	3	4	3	3	2
High	P40939	Trifunctional enzyme subunit alpha, mitochondrial OS=Homo sapiens OX=9606 GN=HADHA PE=1 SV=2	37	11	11	82.9	21	763	9.04	4	3	2	4	5	5	4	3	2	4	5	5
High	Q14684	Ribosomal RNA processing protein 1 homolog B OS=Homo sapiens OX=9606 GN=RRP1B PE=1 SV=3	37	11	11	84.4	22	758	9.76			1	3	6	5			1	3	6	5
High	Q96RQ3	Methylcrotonoyl-CoA carboxylase subunit alpha, mitochondrial OS=Homo sapiens OX=9606 GN=MCCC1 PE=1 SV=3	37	8	8	80.4	17	725	7.78	5	5	6	2	3	2	5	4	6	2	3	2
High	P50570	Dynamin-2 OS=Homo sapiens OX=9606 GN=DNM2 PE=1 SV=2	37	11	6	98	15	870	7.44	8	6	6			2	8	6	6			2
High	Q9BW92	Threonine--tRNA ligase, mitochondrial OS=Homo sapiens OX=9606 GN=TARS2 PE=1 SV=1	37	9	9	81	13	718	7.3	1	2	3	1	6	4	1	2	3	1	6	4
High	Q01081	Splicing factor U2AF 35 kDa subunit OS=Homo sapiens OX=9606 GN=U2AF1 PE=1 SV=3	37	6	6	27.9	29	240	8.81	3	4	3	4	4	4	3	4	3	4	4	4
High	O95347	Structural maintenance of chromosomes protein 2 OS=Homo sapiens OX=9606 GN=SMC2 PE=1 SV=2	37	10	10	135.6	9	1197	8.43	2	3	6	3	4	3	2	3	6	3	4	3
High	Q15029	116 kDa U5 small nuclear ribonucleoprotein component OS=Homo sapiens OX=9606 GN=EFTUD2 PE=1 SV=1	37	11	10	109.4	17	972	5	6	4	5	3	2	1	5	3	5	3	2	1
High	O95336	6-phosphogluconolactonase OS=Homo sapiens OX=9606 GN=PGLS PE=1 SV=2	37	8	8	27.5	44	258	6.05	3	3	4	1	3	2	3	3	3	1	3	2
High	P43487	Ran-specific GTPase-activating protein OS=Homo sapiens OX=9606 GN=RANBP1 PE=1 SV=1	36	6	6	23.3	52	201	5.29	3	3	5	3	4	3	3	3	3	3	3	3
High	Q00535	Cyclin-dependent-like kinase 5 OS=Homo sapiens OX=9606 GN=CDK5 PE=1 SV=3	36	6	5	33.3	27	292	7.66	3	6	6	3	4	2	3	4	5	2	3	2
High	Q16555	Dihydropyrimidinase-related protein 2 OS=Homo sapiens OX=9606 GN=DPYSL2 PE=1 SV=1	36	9	9	62.3	22	572	6.38	4	3	3	5	3	5	4	3	3	5	3	5
High	Q9UN86	Ras GTPase-activating protein-binding protein 2 OS=Homo sapiens OX=9606 GN=G3BP2 PE=1 SV=2	36	8	8	54.1	20	482	5.55	2	2	2	5	6	5	2	2	2	5	6	5
High	P61006	Ras-related protein Rab-8A OS=Homo sapiens OX=9606 GN=RAB8A PE=1 SV=1	36	4	1	23.7	21	207	9.07	4	6	4	3	4	3	3	4	3	3	3	3
High	O00139	Kinesin-like protein KIF2A OS=Homo sapiens OX=9606 GN=KIF2A PE=1 SV=3	36	10	9	79.9	14	706	6.68	2		2	5	3	4	2		2	5	3	4
High	P13987	CD59 glycoprotein OS=Homo sapiens OX=9606 GN=CD59 PE=1 SV=1	36	4	4	14.2	25	128	6.48	5	5	4	2	2	2	4	4	3	2	2	2
High	P62081	40S ribosomal protein S7 OS=Homo sapiens OX=9606 GN=RPS7 PE=1 SV=1	36	6	6	22.1	36	194	10.1	6	6	5	4	1	3	5	5	4	3	1	3
High	P26639	Threonine--tRNA ligase 1, cytoplasmic OS=Homo sapiens OX=9606 GN=TARS1 PE=1 SV=3	36	11	11	83.4	18	723	6.67	4	4	4		1	3	4	4	4		1	3
High	Q3ZBS7	TREMBL:Q3ZBS7 (Bos taurus) Vitronectin	36	7	7	54.1	15	484	6.16	8	6	8	1	2	2	7	6	6	1	2	2
High	Q96EY7	Pentatricopeptide repeat domain-containing protein 3, mitochondrial OS=Homo sapiens OX=9606 GN=PTCD3 PE=1 SV=3	36	9	9	78.5	18	689	6.42	4	2	4	2	4	2	4	2	4	2	3	2
High	P10916	Myosin regulatory light chain 2, ventricular/cardiac muscle isoform OS=Homo sapiens OX=9606 GN=MYL2 PE=1 SV=3	36	9	9	18.8	52	166	5	5	4	4	6	2	6	4	4	4	6	2	6
High	Q01518	Adenylyl cyclase-associated protein 1 OS=Homo sapiens OX=9606 GN=CAP1 PE=1 SV=5	36	8	8	51.9	23	475	8.06	5	3	4	5	3	3	5	3	4	4	3	3
High	Q9NVX2	Notchless protein homolog 1 OS=Homo sapiens OX=9606 GN=NLE1 PE=1 SV=4	36	6	6	53.3	14	485	7.34	3	2	3	5	4	4	3	2	3	5	4	4
High	Q9UBN7	Histone deacetylase 6 OS=Homo sapiens OX=9606 GN=HDAC6 PE=1 SV=2	36	7	7	131.3	8	1215	5.3	3	3	3	3	4	4	3	3	3	3	3	4
High	O15042	U2 snRNP-associated SURP motif-containing protein OS=Homo sapiens OX=9606 GN=U2SURP PE=1 SV=2	36	10	10	118.2	14	1029	8.47	3	3	2	3	5	3	3	3	2	3	5	3
High	P29966	Myristoylated alanine-rich C-kinase substrate OS=Homo sapiens OX=9606 GN=MARCKS PE=1 SV=4	36	5	5	31.5	23	332	4.45	5	5	4	2	2	3	5	5	4	2	2	3
High	Q13228	Methanethiol oxidase OS=Homo sapiens OX=9606 GN=SELENBP1 PE=1 SV=2	35	11	11	52.4	24	472	6.37	3	3	3	3	6	5	3	3	2	3	6	5
High	P28074	Proteasome subunit beta type-5 OS=Homo sapiens OX=9606 GN=PSMB5 PE=1 SV=3	35	7	7	28.5	31	263	6.92	1	2	1	8	5	6	1	2	1	7	5	6
High	Q8TAQ2	SWI/SNF complex subunit SMARCC2 OS=Homo sapiens OX=9606 GN=SMARCC2 PE=1 SV=1	35	10	8	132.8	13	1214	5.69	2	1		1	3	3	2	1		1	3	3
High	Q9Y224	RNA transcription, translation and transport factor protein OS=Homo sapiens OX=9606 GN=RTRAF PE=1 SV=1	35	7	7	28.1	30	244	6.65	3	3	5	3	5	3	3	3	5	3	4	3
High	Q15758	Neutral amino acid transporter B(0) OS=Homo sapiens OX=9606 GN=SLC1A5 PE=1 SV=2	35	7	7	56.6	18	541	5.48	5	7	6	2	3	1	5	6	6	2	3	1
High	Q99848	Probable rRNA-processing protein EBP2 OS=Homo sapiens OX=9606 GN=EBNA1BP2 PE=1 SV=2	35	8	8	34.8	29	306	10.1	1	1	2	5	3	6	1	1	2	5	3	6
High	Q9H3K6	BolA-like protein 2 OS=Homo sapiens OX=9606 GN=BOLA2 PE=1 SV=1	35	4	4	10.1	62	86	6.52	3	5	4	4	4	2	3	4	4	3	3	2
High	A0AV96	RNA-binding protein 47 OS=Homo sapiens OX=9606 GN=RBM47 PE=1 SV=2	35	9	9	64.1	19	593	7.68	1	1		3	3	3	1	1		3	3	3
High	Q9Y4W6	AFG3-like protein 2 OS=Homo sapiens OX=9606 GN=AFG3L2 PE=1 SV=2	35	14	14	88.5	18	797	8.66	5	6	8	1	1		5	6	8	1	1	
High	Q9Y678	Coatomer subunit gamma-1 OS=Homo sapiens OX=9606 GN=COPG1 PE=1 SV=1	35	10	10	97.7	15	874	5.47	3	3	7	2	1	2	3	3	5	2	1	2
High	Q9UHV9	Prefoldin subunit 2 OS=Homo sapiens OX=9606 GN=PFDN2 PE=1 SV=1	35	5	5	16.6	36	154	6.58	2	1	3	4	5	5	2	1	3	3	4	4
High	Q96RP9	Elongation factor G, mitochondrial OS=Homo sapiens OX=9606 GN=GFM1 PE=1 SV=2	35	10	10	83.4	17	751	7.01	2	2	2	2	3	3	2	2	2	2	3	3
High	Q99426	Tubulin-folding cofactor B OS=Homo sapiens OX=9606 GN=TBCB PE=1 SV=2	35	5	5	27.3	23	244	5.15	4	4	4	4	5	4	4	4	3	4	5	4
High	Q15477	Helicase SKI2W OS=Homo sapiens OX=9606 GN=SKIV2L PE=1 SV=3	35	14	14	137.7	16	1246	6.06	9	8	9	1		1	9	8	9	1		1
High	P55884	Eukaryotic translation initiation factor 3 subunit B OS=Homo sapiens OX=9606 GN=EIF3B PE=1 SV=3	35	8	8	92.4	12	814	5	5	4	4	2	2	2	5	4	4	2	2	2
High	Q92769	Histone deacetylase 2 OS=Homo sapiens OX=9606 GN=HDAC2 PE=1 SV=2	35	7	3	55.3	19	488	5.91	3	2	3	1	6	4	3	2	3	1	4	3
High	P49790	Nuclear pore complex protein Nup153 OS=Homo sapiens OX=9606 GN=NUP153 PE=1 SV=2	35	18	18	153.8	16	1475	8.73				1	1	2				1	1	2
High	Q96CN7	Isochorismatase domain-containing protein 1 OS=Homo sapiens OX=9606 GN=ISOC1 PE=1 SV=3	35	7	7	32.2	32	298	7.39	4	4	4	3	5	4	4	4	4	3	5	4
High	Q9H4G0	Band 4.1-like protein 1 OS=Homo sapiens OX=9606 GN=EPB41L1 PE=1 SV=2	35	13	13	98.4	20	881	5.62	5	6	6		2		5	6	6		2	
High	Q2TAA2	Isoamyl acetate-hydrolyzing esterase 1 homolog OS=Homo sapiens OX=9606 GN=IAH1 PE=1 SV=1	34	6	6	27.6	31	248	5.3	4	4	2	3	3	2	4	4	2	3	3	2
High	P63220	40S ribosomal protein S21 OS=Homo sapiens OX=9606 GN=RPS21 PE=1 SV=1	34	5	5	9.1	59	83	8.5	4	3	2	2	5	3	3	3	2	2	5	3
High	Q8IX04	Ubiquitin-conjugating enzyme E2 variant 3 OS=Homo sapiens OX=9606 GN=UEVLD PE=1 SV=2	34	7	7	52.2	19	471	7.09	3	4	5	3	3	4	3	4	5	3	3	4
High	P07858	Cathepsin B OS=Homo sapiens OX=9606 GN=CTSB PE=1 SV=3	34	5	5	37.8	15	339	6.3	5	3	5	4	3	3	4	3	5	4	3	3
High	Q9Y3D2	Methionine-R-sulfoxide reductase B2, mitochondrial OS=Homo sapiens OX=9606 GN=MSRB2 PE=1 SV=2	34	6	6	19.5	51	182	8.63	1	1	2	2	6	3	1	1	2	2	4	3
High	Q99623	Prohibitin-2 OS=Homo sapiens OX=9606 GN=PHB2 PE=1 SV=2	34	8	8	33.3	31	299	9.83	4	3	4	2	3	2	4	3	4	2	2	2
High	P21980	Protein-glutamine gamma-glutamyltransferase 2 OS=Homo sapiens OX=9606 GN=TGM2 PE=1 SV=2	34	11	11	77.3	20	687	5.22	1	3	1	6	8	7	1	3	1	6	8	7
High	P26447	Protein S100-A4 OS=Homo sapiens OX=9606 GN=S100A4 PE=1 SV=1	34	3	3	11.7	28	101	6.11	4	5	4	2	2	2	3	3	3	2	2	2
High	Q8NEZ5	F-box only protein 22 OS=Homo sapiens OX=9606 GN=FBXO22 PE=1 SV=1	34	7	7	44.5	19	403	7.03	5	4	4	4	3	3	4	3	4	4	3	3
High	P49588	Alanine--tRNA ligase, cytoplasmic OS=Homo sapiens OX=9606 GN=AARS1 PE=1 SV=2	34	14	14	106.7	21	968	5.53	2	3	9	1	1		2	3	8	1	1	
High	Q3ZCQ8	Mitochondrial import inner membrane translocase subunit TIM50 OS=Homo sapiens OX=9606 GN=TIMM50 PE=1 SV=2	34	8	8	39.6	27	353	8.37	5	5	5	1	1	1	5	5	5	1	1	1
High	Q9UK41	Vacuolar protein sorting-associated protein 28 homolog OS=Homo sapiens OX=9606 GN=VPS28 PE=1 SV=1	34	8	8	25.4	49	221	5.54	1	3	5	3	4	3	1	3	5	3	3	3
High	Q01780	Exosome component 10 OS=Homo sapiens OX=9606 GN=EXOSC10 PE=1 SV=2	34	9	9	100.8	10	885	8.46				5	5	4				5	5	4
High	Q8IY21	Probable ATP-dependent RNA helicase DDX60 OS=Homo sapiens OX=9606 GN=DDX60 PE=1 SV=3	34	13	13	197.7	9	1712	7.59		1	1	6	8	6		1	1	6	8	6
High	P50579	Methionine aminopeptidase 2 OS=Homo sapiens OX=9606 GN=METAP2 PE=1 SV=1	34	2	2	52.9	9	478	5.82	7	8	3		1	1	2	2	2		1	1
High	O14933	Ubiquitin/ISG15-conjugating enzyme E2 L6 OS=Homo sapiens OX=9606 GN=UBE2L6 PE=1 SV=4	34	5	5	17.8	41	153	7.88	4	3	4	3	4	3	3	3	2	1	2	2
High	Q12769	Nuclear pore complex protein Nup160 OS=Homo sapiens OX=9606 GN=NUP160 PE=1 SV=3	33	13	13	162	11	1436	5.5	2	4	3		1		2	4	3		1	
High	P05026	Sodium/potassium-transporting ATPase subunit beta-1 OS=Homo sapiens OX=9606 GN=ATP1B1 PE=1 SV=1	33	6	6	35	25	303	8.53	3	2	5	1	2	3	3	2	4	1	2	3
High	P13667	Protein disulfide-isomerase A4 OS=Homo sapiens OX=9606 GN=PDIA4 PE=1 SV=2	33	9	9	72.9	20	645	5.07	7	8	7				4	5	6			
High	P18669	Phosphoglycerate mutase 1 OS=Homo sapiens OX=9606 GN=PGAM1 PE=1 SV=2	33	7	7	28.8	38	254	7.18	5	4	5	1	1		5	4	5	1	1	
High	P54819	Adenylate kinase 2, mitochondrial OS=Homo sapiens OX=9606 GN=AK2 PE=1 SV=2	33	7	7	26.5	33	239	7.81	2	3	1	3	4	3	2	3	1	3	4	3
High	Q8IWX8	Calcium homeostasis endoplasmic reticulum protein OS=Homo sapiens OX=9606 GN=CHERP PE=1 SV=3	33	8	8	103.6	9	916	9.04	4	2	1	3	3	2	4	2	1	3	3	2
High	Q14134	Tripartite motif-containing protein 29 OS=Homo sapiens OX=9606 GN=TRIM29 PE=1 SV=2	33	14	14	65.8	24	588	7.15	9	10	10	1	1	1	8	9	10	1	1	1
High	P28066	Proteasome subunit alpha type-5 OS=Homo sapiens OX=9606 GN=PSMA5 PE=1 SV=3	33	5	5	26.4	29	241	4.79	3	3	4	3	3	4	3	3	4	3	3	4
High	Q9UHB9	Signal recognition particle subunit SRP68 OS=Homo sapiens OX=9606 GN=SRP68 PE=1 SV=2	33	13	13	70.7	21	627	8.56			2	2	1	1			2	2	1	1
High	O76094	Signal recognition particle subunit SRP72 OS=Homo sapiens OX=9606 GN=SRP72 PE=1 SV=3	33	14	14	74.6	24	671	9.26	1	1	1	3	3	2	1	1	1	3	3	2
High	P49023	Paxillin OS=Homo sapiens OX=9606 GN=PXN PE=1 SV=3	33	8	8	64.5	16	591	6.19	4	3	4	3	2	3	4	3	4	3	2	3
High	Q9HCY8	Protein S100-A14 OS=Homo sapiens OX=9606 GN=S100A14 PE=1 SV=1	33	5	5	11.7	63	104	5.24	5	3	4	2	1	1	4	3	4	2	1	1
High	Q15418	Ribosomal protein S6 kinase alpha-1 OS=Homo sapiens OX=9606 GN=RPS6KA1 PE=1 SV=2	33	7	3	82.7	10	735	7.83	2	2	4	4	3	2	2	2	4	3	2	2
High	O15143	Actin-related protein 2/3 complex subunit 1B OS=Homo sapiens OX=9606 GN=ARPC1B PE=1 SV=3	33	6	6	40.9	17	372	8.35	4	6	4	4	3	3	4	5	4	4	3	3
High	P13533	Myosin-6 OS=Homo sapiens OX=9606 GN=MYH6 PE=1 SV=5	33	14	9	223.6	9	1939	5.73	3	4	4	6	6	5	3	3	2	6	6	5
High	Q15286	Ras-related protein Rab-35 OS=Homo sapiens OX=9606 GN=RAB35 PE=1 SV=1	33	4	2	23	22	201	8.29	4	5	4	2	3	2	3	3	3	2	2	2
High	P06731	Carcinoembryonic antigen-related cell adhesion molecule 5 OS=Homo sapiens OX=9606 GN=CEACAM5 PE=1 SV=4	32	6	4	76.7	13	702	5.69	4	4	4	2	2	1	4	4	4	2	2	1
High	P35251	Replication factor C subunit 1 OS=Homo sapiens OX=9606 GN=RFC1 PE=1 SV=4	32	11	11	128.2	11	1148	9.36	3	2	4	3	3	2	3	2	4	3	3	2
High	Q96EY1	DnaJ homolog subfamily A member 3, mitochondrial OS=Homo sapiens OX=9606 GN=DNAJA3 PE=1 SV=2	32	6	6	52.5	19	480	9.26	5	3	4	1	1	1	5	3	4	1	1	1
High	P55072	Transitional endoplasmic reticulum ATPase OS=Homo sapiens OX=9606 GN=VCP PE=1 SV=4	32	12	12	89.3	19	806	5.26	4	6	4	1	2	2	4	6	4	1	2	2
High	Q92522	Histone H1.10 OS=Homo sapiens OX=9606 GN=H1-10 PE=1 SV=1	32	5	5	22.5	23	213	10.76		2	4	5	3	2		2	4	4	3	2
High	Q9NQ29	Putative RNA-binding protein Luc7-like 1 OS=Homo sapiens OX=9606 GN=LUC7L PE=1 SV=1	32	5	1	43.7	17	371	9.92	3	3	2	3	3	4	2	2	2	3	3	3
High	Q9Y4P3	Transducin beta-like protein 2 OS=Homo sapiens OX=9606 GN=TBL2 PE=1 SV=1	32	9	9	49.8	25	447	9.44	2	3	2	3	6	4	2	3	2	3	5	4
High	P20618	Proteasome subunit beta type-1 OS=Homo sapiens OX=9606 GN=PSMB1 PE=1 SV=2	32	6	6	26.5	32	241	8.13	2	2	2	5	5	6	2	2	2	5	3	6
High	Q8TDN6	Ribosome biogenesis protein BRX1 homolog OS=Homo sapiens OX=9606 GN=BRIX1 PE=1 SV=2	32	12	12	41.4	38	353	9.92	3	3	1	3	6	2	3	3	1	3	6	2
High	Q8IZW8	Tensin-4 OS=Homo sapiens OX=9606 GN=TNS4 PE=1 SV=3	32	9	9	76.7	17	715	7.34	4	3	2	5	6	5	4	3	2	5	6	5
High	O00232	26S proteasome non-ATPase regulatory subunit 12 OS=Homo sapiens OX=9606 GN=PSMD12 PE=1 SV=3	32	8	8	52.9	22	456	7.65	2	1	3	1	7	1	2	1	3	1	6	1
High	P28482	Mitogen-activated protein kinase 1 OS=Homo sapiens OX=9606 GN=MAPK1 PE=1 SV=3	32	7	4	41.4	23	360	6.98		1	3	3	2	4		1	3	3	2	4

High	P21359	Neurofibromin OS=Homo sapiens OX=9606 GN=NF1 PE=1 SV=2	32	16	16	319.2	7	2839	7.39	7	7	10				7	7	10			
High	P33527	Multidrug resistance-associated protein 1 OS=Homo sapiens OX=9606 GN=ABCC1 PE=1 SV=3	32	11	9	171.5	8	1531	7.11	5	6	9			2	5	5	8			2
High	P05161	Ubiquitin-like protein ISG15 OS=Homo sapiens OX=9606 GN=ISG15 PE=1 SV=5	32	4	4	17.9	30	165	7.44	3	3	3	4	5	2	3	3	3	4	4	2
High	Q9NVI7	ATPase family AAA domain-containing protein 3A OS=Homo sapiens OX=9606 GN=ATAD3A PE=1 SV=2	32	8	8	71.3	12	634	8.98	2	2	3	1	3	2	2	2	3	1	3	2
High	P63151	Serine/threonine-protein phosphatase 2A 55 kDa regulatory subunit B alpha isoform OS=Homo sapiens OX=9606 GN=PPP2R2A PE	32	7	7	51.7	21	447	6.2	5	4	2	4	3	4	5	4	2	4	3	4
High	Q9BUH6	Protein PAXX OS=Homo sapiens OX=9606 GN=PAXX PE=1 SV=2	31	6	6	21.6	33	204	5.48	3	3	3	3	3	3	3	3	3	3	3	3
High	Q14192	Four and a half LIM domains protein 2 OS=Homo sapiens OX=9606 GN=FHL2 PE=1 SV=3	31	10	10	32.2	41	279	7.55			1	2	3	6			1	2	3	6
High	Q9BZE4	GTP-binding protein 4 OS=Homo sapiens OX=9606 GN=GTPBP4 PE=1 SV=3	31	12	12	73.9	18	634	9.5		1		3	5	4		1		3	5	4
High	P04792	Heat shock protein beta-1 OS=Homo sapiens OX=9606 GN=HSPB1 PE=1 SV=2	31	7	7	22.8	39	205	6.4	2	3	2	3	4	2	2	3	2	3	4	2
High	O00161	Synaptosomal-associated protein 23 OS=Homo sapiens OX=9606 GN=SNAP23 PE=1 SV=1	31	10	10	23.3	62	211	5.01	2	1	3		1		2	1	2		1	
High	Q8TCJ2	Dolichyl-diphosphooligosaccharide--protein glycosyltransferase subunit STT3B OS=Homo sapiens OX=9606 GN=STT3B PE=1 SV=1	31	9	8	93.6	10	826	8.91	3	5	5	1	1	2	2	4	4	1	1	2
High	P62316	Small nuclear ribonucleoprotein Sm D2 OS=Homo sapiens OX=9606 GN=SNRPD2 PE=1 SV=1	31	6	6	13.5	56	118	9.91	4	3	2	3	2	4	4	3	2	3	2	4
High	Q8TDB6	E3 ubiquitin-protein ligase DTX3L OS=Homo sapiens OX=9606 GN=DTX3L PE=1 SV=1	31	9	9	83.5	16	740	8.06	4	3	4	4	4	3	4	3	4	4	4	3
High	P49959	Double-strand break repair protein MRE11 OS=Homo sapiens OX=9606 GN=MRE11 PE=1 SV=3	31	11	11	80.5	20	708	5.9	2	4	4	1	1	2	2	4	4	1	1	2
High	Q13619	Cullin-4A OS=Homo sapiens OX=9606 GN=CUL4A PE=1 SV=3	31	12	12	87.6	16	759	8.13	7	8	3		3	1	7	8	3		3	1
High	Q16831	Uridine phosphorylase 1 OS=Homo sapiens OX=9606 GN=UPP1 PE=1 SV=1	31	7	7	33.9	22	310	7.88	1		1	6	5	6	1		1	6	5	5
High	Q13136	Liprin-alpha-1 OS=Homo sapiens OX=9606 GN=PPFIA1 PE=1 SV=1	31	11	11	135.7	13	1202	6.29	5	6	4		1		5	6	4		1	
High	Q96CU9	FAD-dependent oxidoreductase domain-containing protein 1 OS=Homo sapiens OX=9606 GN=FOXRED1 PE=1 SV=2	31	7	7	53.8	19	486	7.78		2	3	3	3	3		2	3	3	3	3
High	P62851	40S ribosomal protein S25 OS=Homo sapiens OX=9606 GN=RPS25 PE=1 SV=1	31	4	4	13.7	28	125	10.11	3	4	3	4	4	4	3	4	3	4	4	4
High	P11177	Pyruvate dehydrogenase E1 component subunit beta, mitochondrial OS=Homo sapiens OX=9606 GN=PDHB PE=1 SV=3	31	9	9	39.2	31	359	6.65	3	5	1		1	1	3	3	1		1	1
High	P43490	Nicotinamide phosphoribosyltransferase OS=Homo sapiens OX=9606 GN=NAMPT PE=1 SV=1	31	9	9	55.5	26	491	7.15	6	5	8	1	3	1	6	5	8	1	3	1
High	Q7LBC6	Lysine-specific demethylase 3B OS=Homo sapiens OX=9606 GN=KDM3B PE=1 SV=2	31	12	12	191.5	9	1761	7.18	1	1		1	4		1	1		1	4	
High	Q9H936	Mitochondrial glutamate carrier 1 OS=Homo sapiens OX=9606 GN=SLC25A22 PE=1 SV=1	31	7	7	34.4	21	323	9.29	6	5	3	1	2	1	6	5	3	1	2	1
High	Q9NZN4	EH domain-containing protein 2 OS=Homo sapiens OX=9606 GN=EHD2 PE=1 SV=2	31	9	7	61.1	19	543	6.46	6	6	4	2	3	3	6	6	4	2	3	3
High	O43852	Calumenin OS=Homo sapiens OX=9606 GN=CALU PE=1 SV=2	31	11	11	37.1	47	315	4.64	7	7	6	2	4	2	7	7	6	2	3	2
High	Q7Z5J4	Retinoic acid-induced protein 1 OS=Homo sapiens OX=9606 GN=RAI1 PE=1 SV=2	31	12	12	203.2	11	1906	8.79		1	1	5	6	7		1	1	5	6	6
High	P62995	Transformer-2 protein homolog beta OS=Homo sapiens OX=9606 GN=TRA2B PE=1 SV=1	31	5	5	33.6	22	288	11.25	3	4	6	3	3	2	3	3	5	3	3	2
High	O95049	Tight junction protein ZO-3 OS=Homo sapiens OX=9606 GN=TJP3 PE=1 SV=3	31	10	10	101.3	13	919	6.76	5	4	4	1	2	1	5	4	4	1	2	1
High	Q14203	Dynactin subunit 1 OS=Homo sapiens OX=9606 GN=DCTN1 PE=1 SV=3	30	10	10	141.6	12	1278	5.81	4	3	5	2		1	4	3	5	2		1
High	P24941	Cyclin-dependent kinase 2 OS=Homo sapiens OX=9606 GN=CDK2 PE=1 SV=2	30	5	3	33.9	17	298	8.68	2	4	5	2	4	2	2	3	4	1	3	2
High	Q8IYB3	Serine/arginine repetitive matrix protein 1 OS=Homo sapiens OX=9606 GN=SRRM1 PE=1 SV=2	30	8	8	102.3	14	904	11.84	1	1	2	4	4	4	1	1	2	4	4	4
High	P62277	40S ribosomal protein S13 OS=Homo sapiens OX=9606 GN=RPS13 PE=1 SV=2	30	5	5	17.2	30	151	10.54	3	4	4	3	3	3	3	4	4	3	3	3
High	Q15370	Elongin-B OS=Homo sapiens OX=9606 GN=ELOB PE=1 SV=1	30	5	5	13.1	49	118	4.88	4	3	3	2	4	2	3	3	3	2	4	2
High	Q9NTJ3	Structural maintenance of chromosomes protein 4 OS=Homo sapiens OX=9606 GN=SMC4 PE=1 SV=2	30	10	10	147.1	9	1288	6.79	4	4	4	2	1	2	4	4	4	2	1	2
High	Q4G0J3	La-related protein 7 OS=Homo sapiens OX=9606 GN=LARP7 PE=1 SV=1	30	8	8	66.9	17	582	9.55	1	2	1	2	2	2	1	2	1	2	2	2
High	Q9UMS4	Pre-mRNA-processing factor 19 OS=Homo sapiens OX=9606 GN=PRPF19 PE=1 SV=1	30	7	7	55.1	17	504	6.61	1	1	1	6	4	5	1	1	1	6	3	5
High	Q9HAN9	Nicotinamide/nicotinic acid mononucleotide adenylyltransferase 1 OS=Homo sapiens OX=9606 GN=NMNAT1 PE=1 SV=1	30	6	6	31.9	29	279	8.87	2	1	2	3	2	4	2	1	2	3	2	3
High	O94901	SUN domain-containing protein 1 OS=Homo sapiens OX=9606 GN=SUN1 PE=1 SV=4	30	11	11	87.1	17	785	6.93	6	7	5	1	2	1	5	6	5	1	2	1
High	O00231	26S proteasome non-ATPase regulatory subunit 11 OS=Homo sapiens OX=9606 GN=PSMD11 PE=1 SV=3	30	12	12	47.4	33	422	6.48		2	1	4	3	5		2	1	4	3	5
High	P55769	NHP2-like protein 1 OS=Homo sapiens OX=9606 GN=SNU13 PE=1 SV=3	30	5	5	14.2	46	128	8.46	2	2	3	4	4	4	2	2	3	3	3	3
High	Q8IWS0	PHD finger protein 6 OS=Homo sapiens OX=9606 GN=PHF6 PE=1 SV=1	30	4	4	41.3	13	365	8.68	3	2	3	3	4	3	3	2	3	3	4	3
High	Q9NZ08	Endoplasmic reticulum aminopeptidase 1 OS=Homo sapiens OX=9606 GN=ERAP1 PE=1 SV=3	30	11	11	107.2	16	941	6.46	7	10	9	1		1	7	9	9	1		1
High	Q9ULX6	A-kinase anchor protein 8-like OS=Homo sapiens OX=9606 GN=AKAP8L PE=1 SV=4	30	6	6	71.6	13	646	5.05	7	7	6				6	6	5			
High	P27824	Calnexin OS=Homo sapiens OX=9606 GN=CANX PE=1 SV=2	30	12	12	67.5	24	592	4.6	3	7	3				3	6	3			
High	Q86V81	THO complex subunit 4 OS=Homo sapiens OX=9606 GN=ALYREF PE=1 SV=3	30	4	4	26.9	23	257	11.15	3	3	2	2	4	2	3	3	2	2	3	2
High	Q9NX58	Cell growth-regulating nucleolar protein OS=Homo sapiens OX=9606 GN=LYAR PE=1 SV=3	30	8	8	43.6	25	379	9.57	1	1	2	2	4	3	1	1	2	2	3	3
High	P21589	5'-nucleotidase OS=Homo sapiens OX=9606 GN=NT5E PE=1 SV=1	30	10	10	63.3	24	574	7.03	1		3		1		1		3		1	
High	Q92600	CCR4-NOT transcription complex subunit 9 OS=Homo sapiens OX=9606 GN=CNOT9 PE=1 SV=1	30	7	7	33.6	24	299	8.03			4	2	3	3			4	2	3	3
High	Q9Y277	Voltage-dependent anion-selective channel protein 3 OS=Homo sapiens OX=9606 GN=VDAC3 PE=1 SV=1	30	5	4	30.6	19	283	8.66	1	3	2	1	3	3	1	3	2	1	3	3
High	P56537	Eukaryotic translation initiation factor 6 OS=Homo sapiens OX=9606 GN=EIF6 PE=1 SV=1	30	5	5	26.6	36	245	4.68	3	1	3	3	3	3	3	1	3	3	3	3
High	P35611	Alpha-adducin OS=Homo sapiens OX=9606 GN=ADD1 PE=1 SV=2	30	10	10	80.9	17	737	5.83	3	1	4	2	1	4	3	1	4	2	1	4
High	O15212	Prefoldin subunit 6 OS=Homo sapiens OX=9606 GN=PFDN6 PE=1 SV=1	30	4	4	14.6	36	129	8.88	2	2	2	4	4	3	2	2	2	2	2	2
High	P61026	Ras-related protein Rab-10 OS=Homo sapiens OX=9606 GN=RAB10 PE=1 SV=1	29	5	3	22.5	32	200	8.38	4	5	3	2	3	2	3	3	2	2	2	2
High	P29692	Elongation factor 1-delta OS=Homo sapiens OX=9606 GN=EEF1D PE=1 SV=5	29	9	8	31.1	43	281	5.01	3	3	2	3	3	2	3	2	2	3	3	2
High	P54578	Ubiquitin carboxyl-terminal hydrolase 14 OS=Homo sapiens OX=9606 GN=USP14 PE=1 SV=3	29	9	9	56	23	494	5.3	4	1	2	1	2	2	4	1	2	1	2	2
High	P07305	Histone H1.0 OS=Homo sapiens OX=9606 GN=H1-0 PE=1 SV=3	29	5	5	20.9	21	194	10.84	3	1	3	3	1	4	3	1	3	2	1	3
High	Q15067	Peroxisomal acyl-coenzyme A oxidase 1 OS=Homo sapiens OX=9606 GN=ACOX1 PE=1 SV=3	29	9	9	74.4	21	660	8.16	4	3	3		3	2	4	3	3		3	2
High	O95292	Vesicle-associated membrane protein-associated protein B/C OS=Homo sapiens OX=9606 GN=VAPB PE=1 SV=3	29	5	4	27.2	29	243	7.3	2	2	4	2	1	4	2	2	4	2	1	4
High	Q9H0U3	Magnesium transporter protein 1 OS=Homo sapiens OX=9606 GN=MAGT1 PE=1 SV=1	29	5	5	38	15	335	9.63	4	4	5	3	2	3	4	4	4	3	2	3
High	Q86VM9	Zinc finger CCCH domain-containing protein 18 OS=Homo sapiens OX=9606 GN=ZC3H18 PE=1 SV=2	29	9	9	106.3	12	953	8.32			1	4	3	5			1	4	3	5
High	P26640	Valine--tRNA ligase OS=Homo sapiens OX=9606 GN=VARS1 PE=1 SV=4	29	9	9	140.4	11	1264	7.59	2	3	3	2	3	2	2	3	3	2	3	2
High	Q5MNZ6	WD repeat domain phosphoinositide-interacting protein 3 OS=Homo sapiens OX=9606 GN=WDR45B PE=1 SV=2	29	5	5	38.1	16	344	7.59	2	3	2	3	3	3	2	3	2	3	3	3
High	P59998	Actin-related protein 2/3 complex subunit 4 OS=Homo sapiens OX=9606 GN=ARPC4 PE=1 SV=3	29	4	4	19.7	23	168	8.43	3	3	3	3	3	3	3	3	3	3	3	3
High	Q12904	Aminoacyl tRNA synthase complex-interacting multifunctional protein 1 OS=Homo sapiens OX=9606 GN=AIMP1 PE=1 SV=2	29	6	6	34.3	28	312	8.43	4	4	4	1	2	2	3	3	3	1	2	2
High	Q96N67	Dedicator of cytokinesis protein 7 OS=Homo sapiens OX=9606 GN=DOCK7 PE=1 SV=4	29	10	10	242.4	6	2140	6.8	2	4	2		2	1	2	4	2		2	1
High	P49321	Nuclear autoantigenic sperm protein OS=Homo sapiens OX=9606 GN=NASP PE=1 SV=2	29	7	7	85.2	9	788	4.3	1		2	4	4	3	1		2	4	4	3
High	P15311	Ezrin OS=Homo sapiens OX=9606 GN=EZR PE=1 SV=4	29	9	5	69.4	15	586	6.27	2	3	7	2	2	2	2	3	6	2	2	2
High	O43670	BUB3-interacting and GLEBS motif-containing protein ZNF207 OS=Homo sapiens OX=9606 GN=ZNF207 PE=1 SV=1	29	3	3	50.7	9	478	9.1	1	2	2	4	4	3	1	2	2	2	2	2
High	Q27J81	Inverted formin-2 OS=Homo sapiens OX=9606 GN=INF2 PE=1 SV=2	29	12	12	135.5	15	1249	5.38	4	5	2	1	2	2	4	5	2	1	2	2
High	O43633	Charged multivesicular body protein 2a OS=Homo sapiens OX=9606 GN=CHMP2A PE=1 SV=1	29	6	6	25.1	25	222	5.97	3	3	4	1	2	1	3	3	4	1	2	1
High	Q15637	Splicing factor 1 OS=Homo sapiens OX=9606 GN=SF1 PE=1 SV=4	29	6	6	68.3	13	639	8.98	3	3	4	2	3		3	3	4	1	2	
High	Q9BQL6	Fermitin family homolog 1 OS=Homo sapiens OX=9606 GN=FERMT1 PE=1 SV=1	29	11	11	77.4	19	677	6.28	7	9	8				7	9	8			
High	Q8TE67	Epidermal growth factor receptor kinase substrate 8-like protein 3 OS=Homo sapiens OX=9606 GN=EPS8L3 PE=1 SV=2	29	10	10	66.8	24	593	7.99	1	2	4	1	2	1	1	2	3	1	2	1
High	Q6IBS0	Twinfilin-2 OS=Homo sapiens OX=9606 GN=TWF2 PE=1 SV=2	29	7	6	39.5	22	349	6.84	3	3	2	2	3	2	3	3	2	2	2	2
High	O96019	Actin-like protein 6A OS=Homo sapiens OX=9606 GN=ACTL6A PE=1 SV=1	29	7	7	47.4	20	429	5.6	2		3	3	3	2	2		3	3	3	2
High	Q9HD45	Transmembrane 9 superfamily member 3 OS=Homo sapiens OX=9606 GN=TM9SF3 PE=1 SV=2	29	9	9	67.8	16	589	7.21	6	4	4		1		6	4	4		1	
High	Q93084	Sarcoplasmic/endoplasmic reticulum calcium ATPase 3 OS=Homo sapiens OX=9606 GN=ATP2A3 PE=1 SV=3	29	6	1	109.2	7	999	5.52	3	3	4	2	3	2	3	3	3	2	3	2
High	Q9UL46	Proteasome activator complex subunit 2 OS=Homo sapiens OX=9606 GN=PSME2 PE=1 SV=4	29	5	5	27.4	23	239	5.73	2	3	1	3	4	4	2	3	1	3	4	4
High	O43809	Cleavage and polyadenylation specificity factor subunit 5 OS=Homo sapiens OX=9606 GN=NUDT21 PE=1 SV=1	29	6	6	26.2	27	227	8.82	2	2	3	2	4	2	2	2	3	2	3	2
High	Q6P1X6	UPF0598 protein C8orf82 OS=Homo sapiens OX=9606 GN=C8orf82 PE=1 SV=2	29	5	5	23.9	31	216	9.14	4	5	3	1	1		4	3	3	1	1	
High	Q9NP79	Vacuolar protein sorting-associated protein VTA1 homolog OS=Homo sapiens OX=9606 GN=VTA1 PE=1 SV=1	29	8	8	33.9	30	307	6.29	4	4	3		2		4	4	2		2	
High	P34896	Serine hydroxymethyltransferase, cytosolic OS=Homo sapiens OX=9606 GN=SHMT1 PE=1 SV=1	29	9	8	53	22	483	7.71	4	2	4	2	2	2	3	2	3	2	2	2
High	P07203	Glutathione peroxidase 1 OS=Homo sapiens OX=9606 GN=GPX1 PE=1 SV=4	29	6	6	22.1	36	203	6.55	3	4	4	2	4	1	3	4	4	2	4	1
High	Q15208	Serine/threonine-protein kinase 38 OS=Homo sapiens OX=9606 GN=STK38 PE=1 SV=1	29	9	7	54.2	23	465	7.15	2	4	5	2	2	2	2	4	4	2	2	2
High	Q13627	Dual specificity tyrosine-phosphorylation-regulated kinase 1A OS=Homo sapiens OX=9606 GN=DYRK1A PE=1 SV=2	29	11	11	85.5	17	763	8.75		1		2	3	3		1		2	3	3
High	P30837	Aldehyde dehydrogenase X, mitochondrial OS=Homo sapiens OX=9606 GN=ALDH1B1 PE=1 SV=4	29	4	2	57.2	9	517	6.99	7	5	4	2	1	2	3	3	3	2	1	2
High	P51116	Fragile X mental retardation syndrome-related protein 2 OS=Homo sapiens OX=9606 GN=FXR2 PE=1 SV=2	29	6	3	74.2	11	673	6.23	3	4	2	3	3	3	3	4	2	3	3	3
High	Q9UBS4	DnaJ homolog subfamily B member 11 OS=Homo sapiens OX=9606 GN=DNAJB11 PE=1 SV=1	28	4	4	40.5	19	358	6.18	3	5	5		1		3	3	3		1	
High	P29373	Cellular retinoic acid-binding protein 2 OS=Homo sapiens OX=9606 GN=CRABP2 PE=1 SV=2	28	5	5	15.7	36	138	5.4	4	3	3	3	2	3	4	3	3	3	2	3
High	O15427	Monocarboxylate transporter 4 OS=Homo sapiens OX=9606 GN=SLC16A3 PE=1 SV=1	28	6	6	49.4	12	465	7.96	6	6	6	1	2		6	6	6	1	2	
High	P62318	Small nuclear ribonucleoprotein Sm D3 OS=Homo sapiens OX=9606 GN=SNRPD3 PE=1 SV=1	28	3	3	13.9	32	126	10.32	4	2	3	3	3	2	3	2	3	2	2	2
High	P07948	Tyrosine-protein kinase Lyn OS=Homo sapiens OX=9606 GN=LYN PE=1 SV=3	28	6	3	58.5	10	512	7.11	3	4	3	3	2	1	3	3	2	2	1	1
High	P62847	40S ribosomal protein S24 OS=Homo sapiens OX=9606 GN=RPS24 PE=1 SV=1	28	4	4	15.4	35	133	10.78	3	2	2	2	3	1	3	2	2	2	2	1
High	P51608	Methyl-CpG-binding protein 2 OS=Homo sapiens OX=9606 GN=MECP2 PE=1 SV=1	28	9	9	52.4	25	486	9.95	8	6	8	1	2	1	7	5	6	1	2	1
High	O95573	Fatty acid CoA ligase Acsl3 OS=Homo sapiens OX=9606 GN=ACSL3 PE=1 SV=3	28	10	9	80.4	19	720	8.38	3	3	5				3	3	4			
High	P20073	Annexin A7 OS=Homo sapiens OX=9606 GN=ANXA7 PE=1 SV=3	28	6	6	52.7	15	488	5.68	4	2	3	3	3	4	4	2	3	3	3	4
High	P53999	Activated RNA polymerase II transcriptional coactivator p15 OS=Homo sapiens OX=9606 GN=SUB1 PE=1 SV=3	28	4	4	14.4	37	127	9.6	2	2	3	2	4	3	2	2	3	2	4	3
High	Q8IWV7	E3 ubiquitin-protein ligase UBR1 OS=Homo sapiens OX=9606 GN=UBR1 PE=1 SV=1	28	13	13	200.1	10	1749	6.01	8	8	8				8	8	8			
High	Q6UB35	Monofunctional C1-tetrahydrofolate synthase, mitochondrial OS=Homo sapiens OX=9606 GN=MTHFD1L PE=1 SV=1	28	10	9	105.7	11	978	8.06			1	5	5	2			1	5	5	2
High	Q9UHD1	Cysteine and histidine-rich domain-containing protein 1 OS=Homo sapiens OX=9606 GN=CHORDC1 PE=1 SV=2	28	9	9	37.5	33	332	7.87	2	3	3	3	3	1	2	3	3	3	3	1
High	Q9C005	Protein dpy-30 homolog OS=Homo sapiens OX=9606 GN=DPY30 PE=1 SV=1	28	2	2	11.2	38	99	4.88			1	1	3	2			1	1	2	1
High	Q92747	Actin-related protein 2/3 complex subunit 1A OS=Homo sapiens OX=9606 GN=ARPC1A PE=1 SV=2	28	8	8	41.5	24	370	8.18	2	4	3	1	3	2	2	4	3	1	3	2
High	O60341	Lysine-specific histone demethylase 1A OS=Homo sapiens OX=9606 GN=KDM1A PE=1 SV=2	28	15	15	92.8	21	852	6.52	1	1	1	1	1		1	1	1	1	1	
High	O75367	Core histone macro-H2A.1 OS=Homo sapiens OX=9606 GN=MACROH2A1 PE=1 SV=4	28	8	8	39.6	26	372	9.79	4	3	3	5	3	5	4	3	3	5	3	5
High	P31689	DnaJ homolog subfamily A member 1 OS=Homo sapiens OX=9606 GN=DNAJA1 PE=1 SV=2	28	9	9	44.8	34	397	7.08	6	5	3	1	2	2	5	5	3	1	2	2
High	P48729	Casein kinase I isoform alpha OS=Homo sapiens OX=9606 GN=CSNK1A1 PE=1 SV=2	28	8	8	38.9	25	337	9.57	3	4	6	1	2	1	3	4	6	1	2	1
High	Q9BW04	Specifically androgen-regulated gene protein OS=Homo sapiens OX=9606 GN=SARG PE=1 SV=2	28	7	7	63.9	17	601	8.62	2	2	2	3	4	3	2	2	2	3	4	3
High	P84022	Mothers against decapentaplegic homolog 3 OS=Homo sapiens OX=9606 GN=SMAD3 PE=1 SV=1	28	5	4	48.1	16	425	7.15	2	2	2	4	3	3	2	2	2	4	3	3
High	Q9UBM7	7-dehydrocholesterol reductase OS=Homo sapiens OX=9606 GN=DHCR7 PE=1 SV=1	28	6	6	54.5	15	475	8.7	5	2	4	3	1	1	5	2	4	3	1	1
High	Q52LJ0	Protein FAM98B OS=Homo sapiens OX=9606 GN=FAM98B PE=1 SV=2	28	6	6	45.5	21	433	8.69			1	3	3	3			1	3	3	2
High	Q06323	Proteasome activator complex subunit 1 OS=Homo sapiens OX=9606 GN=PSME1 PE=1 SV=1	28	8	8	28.7	37	249	6.02	2	3	1	3	6	2	2	3	1	3	4	2
High	Q9Y285	Phenylalanine--tRNA ligase alpha subunit OS=Homo sapiens OX=9606 GN=FARSA PE=1 SV=3	28	11	11	57.5	29	508	7.8	2	4	4	2	1	2	2	4	4	2	1	2
High	Q13283	Ras GTPase-activating protein-binding protein 1 OS=Homo sapiens OX=9606 GN=G3BP1 PE=1 SV=1	27	7	7	52.1	23	466	5.52	3	3	5	1	4	1	3	3	5	1	2	1
High	Q9C0A6	Histone-lysine N-methyltransferase SETD5 OS=Homo sapiens OX=9606 GN=SETD5 PE=1 SV=2	27	6	5	157.4	6	1442	8.46				4	4	4				4	4	4
High	Q9UNF1	Melanoma-associated antigen D2 OS=Homo sapiens OX=9606 GN=MAGED2 PE=1 SV=2	27	8	7	64.9	20	606	9.32	3	3	3	1			3	3	3	1		
High	Q9GZR7	ATP-dependent RNA helicase DDX24 OS=Homo sapiens OX=9606 GN=DDX24 PE=1 SV=1	27	9	9	96.3	17	859	9.06	1	1	1	2	2	2	1	1	1	2	2	2
High	P21291	Cysteine and glycine-rich protein 1 OS=Homo sapiens OX=9606 GN=CSRP1 PE=1 SV=3	27	5	5	20.6	38	193	8.57	3	3	2	3	2	3	3	3	2	3	2	3
High	P57764	Gasdermin-D OS=Homo sapiens OX=9606 GN=GSDMD PE=1 SV=1	27	7	7	52.8	20	484	5.08	3	3	6	2	4	1	2	3	6	2	4	1
High	Q5VZK9	F-actin-uncapping protein LRRC16A OS=Homo sapiens OX=9606 GN=CARMIL1 PE=1 SV=1	27	13	13	151.5	11	1371	7.85	6	7	6			1	6	7	6			1
High	Q9Y6W5	Wiskott-Aldrich syndrome protein family member 2 OS=Homo sapiens OX=9606 GN=WASF2 PE=1 SV=3	27	6	6	54.3	14	498	5.53	3	3	3	1	4	2	3	3	3	1	3	2
High	P51812	Ribosomal protein S6 kinase alpha-3 OS=Homo sapiens OX=9606 GN=RPS6KA3 PE=1 SV=1	27	8	4	83.7	13	740	6.89	1	2	1	3	4	1	1	2	1	2	3	1
High	P30044	Peroxiredoxin-5, mitochondrial OS=Homo sapiens OX=9606 GN=PRDX5 PE=1 SV=4	27	6	6	22.1	39	214	8.7	4	1	4	1	1	3	4	1	4	1	1	3
High	Q9NSY1	BMP-2-inducible protein kinase OS=Homo sapiens OX=9606 GN=BMP2K PE=1 SV=2	27	9	9	129.1	12	1161	6.51	4	3	4				4	3	4			
High	P21796	Voltage-dependent anion-selective channel protein 1 OS=Homo sapiens OX=9606 GN=VDAC1 PE=1 SV=2	27	6	5	30.8	26	283	8.54	2	2	3		2	1	2	2	3		2	1
High	P51149	Ras-related protein Rab-7a OS=Homo sapiens OX=9606 GN=RAB7A PE=1 SV=1	27	6	6	23.5	33	207	6.7	5	4	4		2		5	4	4		2	
High	O94906	Pre-mRNA-processing factor 6 OS=Homo sapiens OX=9606 GN=PRPF6 PE=1 SV=1	27	11	11	106.9	14	941	8.25	1	2	1		2	1	1	2	1		2	1
High	P57735	Ras-related protein Rab-25 OS=Homo sapiens OX=9606 GN=RAB25 PE=1 SV=2	27	4	3	23.5	19	213	5.96	2	3	4	2	3	3	2	3	3	2	3	3
High	Q9H074	Polyadenylate-binding protein-interacting protein 1 OS=Homo sapiens OX=9606 GN=PAIP1 PE=1 SV=1	27	6	6	53.5	14	479	4.81	2	2	3	2	2	3	2	2	3	2	2	2
High	Q3YEC7	Rab-like protein 6 OS=Homo sapiens OX=9606 GN=RABL6 PE=1 SV=2	27	7	7	79.5	13	729	5.22	4	2	3	2	2	1	4	2	3	2	2	1
High	Q2TB90	Hexokinase HKDC1 OS=Homo sapiens OX=9606 GN=HKDC1 PE=1 SV=3	27	11	8	102.5	11	917	7.12	3	3	3	1	1		3	3	3	1	1	
High	Q15645	Pachytene checkpoint protein 2 homolog OS=Homo sapiens OX=9606 GN=TRIP13 PE=1 SV=2	27	11	11	48.5	26	432	6.09	4	5	7		1		4	5	7		1	
High	Q15008	26S proteasome non-ATPase regulatory subunit 6 OS=Homo sapiens OX=9606 GN=PSMD6 PE=1 SV=1	27	8	8	45.5	21	389	5.62	1	2	1	3	4	4	1	2	1	3	4	4
High	Q14126	Desmoglein-2 OS=Homo sapiens OX=9606 GN=DSG2 PE=1 SV=2	27	7	7	122.2	11	1118	5.24	4	5	3	2	1	1	4	5	3	2	1	1
High	P50416	Carnitine O-palmitoyltransferase 1, liver isoform OS=Homo sapiens OX=9606 GN=CPT1A PE=1 SV=2	27	10	10	88.3	14	773	8.65	7	4	5				7	4	5			
High	Q96I25	Splicing factor 45 OS=Homo sapiens OX=9606 GN=RBM17 PE=1 SV=1	27	6	6	44.9	16	401	5.97	2	1		3	3	3	2	1		3	3	3
High	P27797	Calreticulin OS=Homo sapiens OX=9606 GN=CALR PE=1 SV=1	27	7	7	48.1	23	417	4.44	7	5	5	1	1	1	6	5	5	1	1	1
High	Q9BQ67	Glutamate-rich WD repeat-containing protein 1 OS=Homo sapiens OX=9606 GN=GRWD1 PE=1 SV=1	26	5	5	49.4	17	446	4.92	2	2	1	3	3	3	2	2	1	3	3	3
High	P61758	Prefoldin subunit 3 OS=Homo sapiens OX=9606 GN=VBP1 PE=1 SV=4	26	6	6	22.6	26	197	7.11	1		2	4	5	5	1		2	4	5	5
High	Q6PGP7	Tetratricopeptide repeat protein 37 OS=Homo sapiens OX=9606 GN=TTC37 PE=1 SV=1	26	12	12	175.4	11	1564	7.53	9	6	7				9	6	7			
High	P21912	Succinate dehydrogenase [ubiquinone] iron-sulfur subunit, mitochondrial OS=Homo sapiens OX=9606 GN=SDHB PE=1 SV=3	26	6	6	31.6	22	280	8.76	3	3	1	1	5	3	3	3	1	1	4	3
High	Q5T440	Putative transferase CAF17, mitochondrial OS=Homo sapiens OX=9606 GN=IBA57 PE=1 SV=1	26	4	4	38.1	11	356	9.83		1	3	3	3	4		1	3	3	3	4
High	P02768-1	SWISS-PROT:P02768-1 Tax_Id=9606 Gene_Symbol=ALB Isoform 1 of Serum albumin precursor	26	7	3	69.3	10	609	6.28	2	3	1	3	6	2	2	3	1	3	6	2
High	Q8N4T8	3-oxoacyl-[acyl-carrier-protein] reductase OS=Homo sapiens OX=9606 GN=CBR4 PE=1 SV=3	26	6	6	25.3	35	237	9.33	4	2	2	2	3	2	4	2	2	2	3	2
High	P61619	Protein transport protein Sec61 subunit alpha isoform 1 OS=Homo sapiens OX=9606 GN=SEC61A1 PE=1 SV=2	26	5	5	52.2	11	476	8.06	2	2	2	3	2	3	2	2	2	3	2	3
High	Q12800	Alpha-globin transcription factor CP2 OS=Homo sapiens OX=9606 GN=TFCP2 PE=1 SV=2	26	7	6	57.2	21	502	5.8	6	7	5		1		6	7	5		1	
High	Q9Y6E2	eIF5-mimic protein 1 OS=Homo sapiens OX=9606 GN=BZW2 PE=1 SV=1	26	10	9	48.1	24	419	6.68	2	1	3	2	4	2	2	1	3	2	4	2
High	Q8N684	Cleavage and polyadenylation specificity factor subunit 7 OS=Homo sapiens OX=9606 GN=CPSF7 PE=1 SV=1	26	7	7	52	16	471	8				3	6	4				3	6	4
High	P13796	Plastin-2 OS=Homo sapiens OX=9606 GN=LCP1 PE=1 SV=6	26	7	1	70.2	11	627	5.43	1	2	2	3	5	5	1	2	2	3	5	5
High	O14618	Copper chaperone for superoxide dismutase OS=Homo sapiens OX=9606 GN=CCS PE=1 SV=1	26	5	5	29	16	274	5.58	1	1		2	5	3	1	1		2	5	3
High	P52292	Importin subunit alpha-1 OS=Homo sapiens OX=9606 GN=KPNA2 PE=1 SV=1	26	7	7	57.8	16	529	5.4	1		3	3	4	4	1		3	3	4	4
High	Q13765	Nascent polypeptide-associated complex subunit alpha OS=Homo sapiens OX=9606 GN=NACA PE=1 SV=1	26	3	3	23.4	20	215	4.56	3	2	3	3	2	2	2	1	2	2	1	1
High	O94903	Pyridoxal phosphate homeostasis protein OS=Homo sapiens OX=9606 GN=PLPBP PE=1 SV=1	26	7	7	30.3	29	275	7.5	1	3	3	4		4	1	3	2	4		4
High	O00505	Importin subunit alpha-4 OS=Homo sapiens OX=9606 GN=KPNA3 PE=1 SV=2	26	6	3	57.8	13	521	4.94		1	2	2	3	2		1	2	2	3	2
High	Q9NQ88	Fructose-2,6-bisphosphatase TIGAR OS=Homo sapiens OX=9606 GN=TIGAR PE=1 SV=1	26	5	5	30	26	270	7.69	1	2	2	2	3	2	1	2	2	2	3	2
High	Q8NAP3	Zinc finger and BTB domain-containing protein 38 OS=Homo sapiens OX=9606 GN=ZBTB38 PE=1 SV=2	26	8	8	134.2	7	1195	8.03				3	4	3				3	4	3
High	O15305	Phosphomannomutase 2 OS=Homo sapiens OX=9606 GN=PMM2 PE=1 SV=1	26	6	6	28.1	23	246	6.77	1	1	2	3	3	2	1	1	2	3	3	2
High	Q12849	G-rich sequence factor 1 OS=Homo sapiens OX=9606 GN=GRSF1 PE=1 SV=3	26	7	7	53.1	19	480	6.19		2	1	3	3	3		2	1	3	3	3
High	Q02750	Dual specificity mitogen-activated protein kinase kinase 1 OS=Homo sapiens OX=9606 GN=MAP2K1 PE=1 SV=2	26	7	5	43.4	25	393	6.62	4	4	4	1	2	1	4	4	3	1	2	1
High	P40926	Malate dehydrogenase, mitochondrial OS=Homo sapiens OX=9606 GN=MDH2 PE=1 SV=3	26	8	8	35.5	29	338	8.68	3	1	6	5	3	5	3	1	6	5	2	5
High	Q8N2K0	Lysophosphatidylserine lipase ABHD12 OS=Homo sapiens OX=9606 GN=ABHD12 PE=1 SV=2	26	7	7	45.1	21	398	8.65	4	4	4	1	2	1	4	4	2	1	2	1
High	P62899	60S ribosomal protein L31 OS=Homo sapiens OX=9606 GN=RPL31 PE=1 SV=1	26	3	3	14.5	25	125	10.54	3	3	3	2	2	2	3	3	2	2	2	2
High	P53007	Tricarboxylate transport protein, mitochondrial OS=Homo sapiens OX=9606 GN=SLC25A1 PE=1 SV=2	26	5	5	34	17	311	9.89	3	4	2	2	1	1	3	3	1	2	1	1
High	Q13573	SNW domain-containing protein 1 OS=Homo sapiens OX=9606 GN=SNW1 PE=1 SV=1	26	7	7	61.5	15	536	9.52	3	3	2	1	2	3	3	3	2	1	2	3
High	O43823	A-kinase anchor protein 8 OS=Homo sapiens OX=9606 GN=AKAP8 PE=1 SV=1	26	10	10	76.1	22	692	5.15	1	3	3		1		1	3	3		1	
High	Q12789	General transcription factor 3C polypeptide 1 OS=Homo sapiens OX=9606 GN=GTF3C1 PE=1 SV=4	26	13	13	238.7	8	2109	7.3	1		2		5	2	1		2		5	2
High	O60381	HMG box-containing protein 1 OS=Homo sapiens OX=9606 GN=HBP1 PE=1 SV=2	26	9	9	57.6	25	514	6.19				2	4	2				2	4	2
High	Q9NX24	H/ACA ribonucleoprotein complex subunit 2 OS=Homo sapiens OX=9606 GN=NHP2 PE=1 SV=1	26	3	3	17.2	30	153	8.22	2	2	3	1	2	1	2	2	3	1	2	1
High	Q9P287	BRCA2 and CDKN1A-interacting protein OS=Homo sapiens OX=9606 GN=BCCIP PE=1 SV=1	26	6	6	36	25	314	4.61	4	4	4	2	1	1	4	4	4	2	1	1
High	Q96IU4	Protein ABHD14B OS=Homo sapiens OX=9606 GN=ABHD14B PE=1 SV=1	26	8	8	22.3	48	210	6.4	5	5	5	1	1		5	5	5	1	1	

High	P50851	Lipopolysaccharide-responsive and beige-like anchor protein OS=Homo sapiens OX=9606 GN=LRBA PE=1 SV=4	26	12	12	318.9	5	2863	5.6	2	2	7	1		1	2	2	7	1		1
High	P16455	Methylated-DNA--protein-cysteine methyltransferase OS=Homo sapiens OX=9606 GN=MGMT PE=1 SV=1	26	6	6	21.6	33	207	8.1	3	3	2	2	3	2	3	3	2	2	3	2
High	Q92552	28S ribosomal protein S27, mitochondrial OS=Homo sapiens OX=9606 GN=MRPS27 PE=1 SV=3	26	9	9	47.6	25	414	6.18	2	3	1	2	2	3	2	3	1	2	2	3
High	Q9BRK5	45 kDa calcium-binding protein OS=Homo sapiens OX=9606 GN=SDF4 PE=1 SV=1	26	6	6	41.8	20	362	4.86	5	5	6	2	1	2	4	4	6	2	1	2
High	Q96SB4	SRSF protein kinase 1 OS=Homo sapiens OX=9606 GN=SRPK1 PE=1 SV=2	26	9	7	74.3	17	655	6.16			2	2	4	3			2	2	4	3
High	P62857	40S ribosomal protein S28 OS=Homo sapiens OX=9606 GN=RPS28 PE=1 SV=1	26	2	2	7.8	30	69	10.7	3	2	3	2	3	3	2	2	2	1	2	2
High	O00410	Importin-5 OS=Homo sapiens OX=9606 GN=IPO5 PE=1 SV=4	26	7	7	123.6	9	1097	4.94	3	2		1		2	3	2		1		2
High	Q9NY12	H/ACA ribonucleoprotein complex subunit 1 OS=Homo sapiens OX=9606 GN=GAR1 PE=1 SV=1	25	4	4	22.3	25	217	10.92	3	3	4	2	1	1	3	3	4	2	1	1
High	P43304	Glycerol-3-phosphate dehydrogenase, mitochondrial OS=Homo sapiens OX=9606 GN=GPD2 PE=1 SV=3	25	10	10	80.8	15	727	7.69				2	2	4				2	2	4
High	Q9UKV3	Apoptotic chromatin condensation inducer in the nucleus OS=Homo sapiens OX=9606 GN=ACIN1 PE=1 SV=2	25	8	8	151.8	8	1341	6.43	2	1	2	4	5	2	2	1	2	4	4	2
High	P63165	Small ubiquitin-related modifier 1 OS=Homo sapiens OX=9606 GN=SUMO1 PE=1 SV=1	25	3	3	11.6	28	101	5.52				1	2	1				1	2	1
High	O60784	Target of Myb protein 1 OS=Homo sapiens OX=9606 GN=TOM1 PE=1 SV=2	25	7	7	53.8	25	492	4.7	3	3	3				3	3	3			
High	Q14244	Ensconsin OS=Homo sapiens OX=9606 GN=MAP7 PE=1 SV=1	25	5	5	84	7	749	9.61	2	3	3	2	3	2	2	3	3	2	3	2
High	O15145	Actin-related protein 2/3 complex subunit 3 OS=Homo sapiens OX=9606 GN=ARPC3 PE=1 SV=3	25	4	4	20.5	21	178	8.59	4	3	3	1	2	1	4	3	3	1	2	1
High	Q9Y5M8	Signal recognition particle receptor subunit beta OS=Homo sapiens OX=9606 GN=SRPRB PE=1 SV=3	25	6	6	29.7	27	271	9.04	3	2	4	2	2	2	3	2	4	2	2	2
High	P62070	Ras-related protein R-Ras2 OS=Homo sapiens OX=9606 GN=RRAS2 PE=1 SV=1	25	5	3	23.4	31	204	6.01	3	4	3	1	1	1	3	4	2	1	1	1
High	P08559	Pyruvate dehydrogenase E1 component subunit alpha, somatic form, mitochondrial OS=Homo sapiens OX=9606 GN=PDHA1 PE=1	25	9	9	43.3	22	390	8.06	1			2	3	1	1			2	3	1
High	Q9NR19	Acetyl-coenzyme A synthetase, cytoplasmic OS=Homo sapiens OX=9606 GN=ACSS2 PE=1 SV=1	25	5	5	78.5	6	701	6.46	3	3	4	2	2	1	3	3	4	2	2	1
High	Q99471	Prefoldin subunit 5 OS=Homo sapiens OX=9606 GN=PFDN5 PE=1 SV=2	25	5	5	17.3	38	154	6.33	2	2	2	2	2	2	2	2	2	2	2	2
High	Q92925	SWI/SNF-related matrix-associated actin-dependent regulator of chromatin subfamily D member 2 OS=Homo sapiens OX=9606 GI	25	8	7	58.9	19	531	9.64	1	1	1	2	2	1	1	1	1	2	2	1
High	O95299	NADH dehydrogenase [ubiquinone] 1 alpha subcomplex subunit 10, mitochondrial OS=Homo sapiens OX=9606 GN=NDUFA10 PE=	25	4	4	40.7	11	355	8.48	2	3	3	2	2	3	2	3	3	2	2	3
High	O75663	TIP41-like protein OS=Homo sapiens OX=9606 GN=TIPRL PE=1 SV=2	25	7	7	31.4	38	272	5.91	4	3	3	1	2		4	3	3	1	2	
High	Q05193	Dynamin-1 OS=Homo sapiens OX=9606 GN=DNM1 PE=1 SV=2	25	6	1	97.3	7	864	7.17	4	3	5			2	4	3	5			2
High	P60953	Cell division control protein 42 homolog OS=Homo sapiens OX=9606 GN=CDC42 PE=1 SV=2	25	4	3	21.2	26	191	6.55	3	5	2	2	2	2	3	4	2	2	2	2
High	O00193	Small acidic protein OS=Homo sapiens OX=9606 GN=SMAP PE=1 SV=1	25	4	4	20.3	22	183	4.72	1	1	3	2	4	3	1	1	3	2	4	2
High	O94776	Metastasis-associated protein MTA2 OS=Homo sapiens OX=9606 GN=MTA2 PE=1 SV=1	25	8	7	75	14	668	9.66	1	2	4	2	3	1	1	2	3	2	3	1
High	Q8TD30	Alanine aminotransferase 2 OS=Homo sapiens OX=9606 GN=GPT2 PE=1 SV=1	25	8	8	57.9	25	523	7.71	1	4	4		2		1	4	4		2	
High	Q93052	Lipoma-preferred partner OS=Homo sapiens OX=9606 GN=LPP PE=1 SV=1	25	6	6	65.7	17	612	7.37	2	2	2	3	3	2	2	2	2	3	3	2
High	Q96FJ0	AMSH-like protease OS=Homo sapiens OX=9606 GN=STAMBPL1 PE=1 SV=2	25	6	6	49.8	20	436	7.23	3	3	4	1		1	3	3	4	1		1
High	P98082	Disabled homolog 2 OS=Homo sapiens OX=9606 GN=DAB2 PE=1 SV=3	25	7	7	82.4	17	770	5.53	2	2	4				2	2	4			
High	O00469	Procollagen-lysine,2-oxoglutarate 5-dioxygenase 2 OS=Homo sapiens OX=9606 GN=PLOD2 PE=1 SV=2	25	10	10	84.6	16	737	6.71	2		3		2		2		3		2	
High	Q14498	RNA-binding protein 39 OS=Homo sapiens OX=9606 GN=RBM39 PE=1 SV=2	25	5	5	59.3	12	530	10.1	2	2	2	2	2	2	2	2	2	2	2	2
High	P31948	Stress-induced-phosphoprotein 1 OS=Homo sapiens OX=9606 GN=STIP1 PE=1 SV=1	25	7	7	62.6	16	543	6.8	4	3	5	1	2	1	4	3	5	1	2	1
High	Q92506	(3R)-3-hydroxyacyl-CoA dehydrogenase OS=Homo sapiens OX=9606 GN=HSD17B8 PE=1 SV=2	25	5	5	27	25	261	6.54	1	1	1	3	3	3	1	1	1	3	3	3
High	P09211	Glutathione S-transferase P OS=Homo sapiens OX=9606 GN=GSTP1 PE=1 SV=2	25	5	5	23.3	36	210	5.64	3	3	3	1	2	1	3	3	3	1	2	1
High	O75436	Vacuolar protein sorting-associated protein 26A OS=Homo sapiens OX=9606 GN=VPS26A PE=1 SV=2	25	5	4	38.1	22	327	6.57	2	3	3	3	4	2	2	3	3	3	4	2
High	Q9NQT8	Kinesin-like protein KIF13B OS=Homo sapiens OX=9606 GN=KIF13B PE=1 SV=2	25	7	6	202.7	5	1826	5.88	4	4	2		2		4	4	2		2	
High	Q16543	Hsp90 co-chaperone Cdc37 OS=Homo sapiens OX=9606 GN=CDC37 PE=1 SV=1	25	7	7	44.4	21	378	5.25	2	2	5	4	2	2	2	2	5	4	2	2
High	Q9Y6R7	IgGFc-binding protein OS=Homo sapiens OX=9606 GN=FCGBP PE=1 SV=3	25	11	11	571.6	6	5405	5.34												
High	Q9Y5A9	YTH domain-containing family protein 2 OS=Homo sapiens OX=9606 GN=YTHDF2 PE=1 SV=2	25	4	3	62.3	7	579	8.79	3	3	3	3	3	2	3	3	3	3	3	2
High	Q9UHD2	Serine/threonine-protein kinase TBK1 OS=Homo sapiens OX=9606 GN=TBK1 PE=1 SV=1	25	9	9	83.6	14	729	6.79	2	1	2	1	2		2	1	2	1	2	
High	Q8TCT9	Minor histocompatibility antigen H13 OS=Homo sapiens OX=9606 GN=HM13 PE=1 SV=1	25	5	5	41.5	16	377	6.43	2	3	3		3	1	2	3	3		3	1
High	O75643	U5 small nuclear ribonucleoprotein 200 kDa helicase OS=Homo sapiens OX=9606 GN=SNRNP200 PE=1 SV=2	25	15	15	244.4	10	2136	6.06	3		2	1	3		3		2	1	3	
High	Q92905	COP9 signalosome complex subunit 5 OS=Homo sapiens OX=9606 GN=COPS5 PE=1 SV=4	25	6	6	37.6	23	334	6.54	2	2	4		3		2	2	4		3	
High	P08758	Annexin A5 OS=Homo sapiens OX=9606 GN=ANXA5 PE=1 SV=2	25	8	8	35.9	26	320	5.05	3	2	3	4	5	4	3	2	3	2	5	3
High	Q9NZ01	Very-long-chain enoyl-CoA reductase OS=Homo sapiens OX=9606 GN=TECR PE=1 SV=1	25	8	8	36	25	308	9.45	5	6	3			1	5	5	3			1
High	Q9H7C9	Mth938 domain-containing protein OS=Homo sapiens OX=9606 GN=AAMDC PE=1 SV=1	25	5	5	13.3	43	122	8.46	2		3	3		4	2		3	3		3
High	Q9P035	Very-long-chain (3R)-3-hydroxyacyl-CoA dehydratase 3 OS=Homo sapiens OX=9606 GN=HACD3 PE=1 SV=2	24	5	5	43.1	14	362	8.94	5	4	2	1	2		5	4	2	1	2	
High	P28331	NADH-ubiquinone oxidoreductase 75 kDa subunit, mitochondrial OS=Homo sapiens OX=9606 GN=NDUFS1 PE=1 SV=3	24	11	11	79.4	19	727	6.23	1	2	3	1	2		1	2	3	1	2	
High	A3KMH1	von Willebrand factor A domain-containing protein 8 OS=Homo sapiens OX=9606 GN=VWA8 PE=1 SV=2	24	7	7	214.7	4	1905	7.4	7	6	3		1		7	6	3		1	
High	P11908	Ribose-phosphate pyrophosphokinase 2 OS=Homo sapiens OX=9606 GN=PRPS2 PE=1 SV=2	24	5	1	34.7	20	318	6.61	3	3	3	3	1	3	3	3	3	3	1	3
High	Q9UBV8	Peflin OS=Homo sapiens OX=9606 GN=PEF1 PE=1 SV=1	24	4	4	30.4	13	284	6.54	3	3	3	3	2	1	3	3	3	3	2	1
High	Q9H2P0	Activity-dependent neuroprotector homeobox protein OS=Homo sapiens OX=9606 GN=ADNP PE=1 SV=1	24	7	7	123.5	8	1102	7.34	3	2	3	2	2	3	3	2	3	2	2	3
High	Q8IYS1	Xaa-Arg dipeptidase OS=Homo sapiens OX=9606 GN=PM20D2 PE=1 SV=2	24	3	3	47.7	11	436	5.85	2	3	2	2	2	2	2	3	2	2	2	2
High	P62837	Ubiquitin-conjugating enzyme E2 D2 OS=Homo sapiens OX=9606 GN=UBE2D2 PE=1 SV=1	24	4	3	16.7	41	147	7.83	2	4	4	1	3	2	2	4	3	1	2	2
High	P40429	60S ribosomal protein L13a OS=Homo sapiens OX=9606 GN=RPL13A PE=1 SV=2	24	4	4	23.6	21	203	10.93	2	1	2	2	2	2	2	1	1	2	2	2
High	O14980	Exportin-1 OS=Homo sapiens OX=9606 GN=XPO1 PE=1 SV=1	24	9	9	123.3	9	1071	6.06	3	4	2		2		2	4	2		2	
High	P12882	Myosin-1 OS=Homo sapiens OX=9606 GN=MYH1 PE=1 SV=3	24	9	4	223	6	1939	5.74	2	2	4	3	6	3	2	1	2	3	6	3
High	P62266	40S ribosomal protein S23 OS=Homo sapiens OX=9606 GN=RPS23 PE=1 SV=3	24	3	3	15.8	21	143	10.49	2	2	3	2	2	3	2	2	3	2	2	2
High	P02452	Collagen alpha-1(I) chain OS=Homo sapiens OX=9606 GN=COL1A1 PE=1 SV=6	24	4	4	138.8	3	1464	5.8				5	14	5				3	4	3
High	Q7L2E3	ATP-dependent RNA helicase DHX30 OS=Homo sapiens OX=9606 GN=DHX30 PE=1 SV=1	24	8	8	133.9	9	1194	8.78	1	1	1	2	2	3	1	1	1	2	2	3
High	Q9Y3A5	Ribosome maturation protein SBDS OS=Homo sapiens OX=9606 GN=SBDS PE=1 SV=4	24	6	6	28.7	24	250	8.75	4	3	3	1	1	1	4	3	3	1	1	1
High	Q96HE7	ERO1-like protein alpha OS=Homo sapiens OX=9606 GN=ERO1A PE=1 SV=2	24	9	9	54.4	27	468	5.68	2	3	2	2	1		2	3	2	2	1	
High	P02584	SWISS-PROT:P02584 (Bos taurus) Profilin-1	24	4	1	15	31	140	8.28	3	3	3	3	2	2	3	3	3	3	2	2
High	Q99700	Ataxin-2 OS=Homo sapiens OX=9606 GN=ATXN2 PE=1 SV=2	24	5	5	140.2	5	1313	9.57	1	1	1	4	2	4	1	1	1	4	2	4
High	Q02978	Mitochondrial 2-oxoglutarate/malate carrier protein OS=Homo sapiens OX=9606 GN=SLC25A11 PE=1 SV=3	24	7	7	34	23	314	9.91	5	4	7				5	4	7			
High	Q15382	GTP-binding protein Rheb OS=Homo sapiens OX=9606 GN=RHEB PE=1 SV=1	24	5	5	20.5	28	184	5.92	2	3	2	1	2	1	2	3	2	1	2	1
High	Q9HC78	Zinc finger and BTB domain-containing protein 20 OS=Homo sapiens OX=9606 GN=ZBTB20 PE=1 SV=3	24	10	10	81	20	741	6.49												
High	P20340	Ras-related protein Rab-6A OS=Homo sapiens OX=9606 GN=RAB6A PE=1 SV=3	24	5	4	23.6	25	208	5.54	3	2	3	2	2	1	2	1	2	2	1	1
High	Q9UEY8	Gamma-adducin OS=Homo sapiens OX=9606 GN=ADD3 PE=1 SV=1	24	9	9	79.1	16	706	6.32		1	2		4			1	2		4	
High	Q9BY42	Replication termination factor 2 OS=Homo sapiens OX=9606 GN=RTF2 PE=1 SV=3	24	6	6	33.9	25	306	8.59	1	3	2	4	4	3	1	3	2	4	4	3
High	P38606	V-type proton ATPase catalytic subunit A OS=Homo sapiens OX=9606 GN=ATP6V1A PE=1 SV=2	23	8	8	68.3	16	617	5.52	4	4	2			1	4	4	2			1
High	Q9BQA1	Methylosome protein 50 OS=Homo sapiens OX=9606 GN=WDR77 PE=1 SV=1	23	6	6	36.7	25	342	5.17		1	2	4	4	2		1	2	3	3	2
High	Q9BV57	1,2-dihydroxy-3-keto-5-methylthiopentene dioxygenase OS=Homo sapiens OX=9606 GN=ADI1 PE=1 SV=1	23	4	4	21.5	24	179	5.68	1	3	2	2	2	2	1	2	2	2	2	2
High	Q8IZP0	Abl interactor 1 OS=Homo sapiens OX=9606 GN=ABI1 PE=1 SV=4	23	7	4	55	18	508	7.06	2	2		1	1	1	2	2		1	1	1
High	Q9BW27	Nuclear pore complex protein Nup85 OS=Homo sapiens OX=9606 GN=NUP85 PE=1 SV=1	23	10	10	75	21	656	5.55	1	2	2			1	1	2	2			1
High	Q8WXI9	Transcriptional repressor p66-beta OS=Homo sapiens OX=9606 GN=GATAD2B PE=1 SV=1	23	5	4	65.2	13	593	9.7		2	2	2	3	3		2	2	2	3	3
High	Q9UBE0	SUMO-activating enzyme subunit 1 OS=Homo sapiens OX=9606 GN=SAE1 PE=1 SV=1	23	8	8	38.4	30	346	5.3	3	3	6		1		3	3	6		1	
High	Q9Y263	Phospholipase A-2-activating protein OS=Homo sapiens OX=9606 GN=PLAA PE=1 SV=2	23	8	8	87.1	16	795	6.37	1		3	2	2	2	1		3	2	2	2
High	Q5TC82	Roquin-1 OS=Homo sapiens OX=9606 GN=RC3H1 PE=1 SV=1	23	9	5	125.7	12	1133	7.27				5	2	4				4	2	4
High	O43615	Mitochondrial import inner membrane translocase subunit TIM44 OS=Homo sapiens OX=9606 GN=TIMM44 PE=1 SV=2	23	10	10	51.3	25	452	8.32	4	4	6		1		4	4	6		1	
High	O43175	D-3-phosphoglycerate dehydrogenase OS=Homo sapiens OX=9606 GN=PHGDH PE=1 SV=4	23	10	10	56.6	21	533	6.71	1	2	1			1	1	2	1			1
High	Q14653	Interferon regulatory factor 3 OS=Homo sapiens OX=9606 GN=IRF3 PE=1 SV=1	23	6	6	47.2	20	427	5.34	3	4	6	1		1	3	3	5	1		1
High	P42566	Epidermal growth factor receptor substrate 15 OS=Homo sapiens OX=9606 GN=EPS15 PE=1 SV=2	23	7	7	98.6	8	896	4.64	2	1	1		1		2	1	1		1	
High	Q9UBI6	Guanine nucleotide-binding protein G(I)/G(S)/G(O) subunit gamma-12 OS=Homo sapiens OX=9606 GN=GNG12 PE=1 SV=3	23	4	4	8	69	72	8.97	2	3	3	1	1	2	2	3	3	1	1	2
High	Q96C86	m7GpppX diphosphatase OS=Homo sapiens OX=9606 GN=DCPS PE=1 SV=2	23	6	6	38.6	21	337	6.38	1	1	1	3	5	1	1	1	1	3	5	1
High	P49006	MARCKS-related protein OS=Homo sapiens OX=9606 GN=MARCKSL1 PE=1 SV=2	23	5	5	19.5	55	195	4.67	2	2	2	1	2	1	2	2	2	1	2	1
High	Q9H6Q4	Cytosolic iron-sulfur assembly component 3 OS=Homo sapiens OX=9606 GN=CIAO3 PE=1 SV=1	23	8	8	53	26	476	7.23	2	2	3	3	1	3	2	2	3	3	1	3
High	P40121	Macrophage-capping protein OS=Homo sapiens OX=9606 GN=CAPG PE=1 SV=2	23	6	6	38.5	23	348	6.19	3	3	4	1	1	1	3	3	4	1	1	1
High	O75947	ATP synthase subunit d, mitochondrial OS=Homo sapiens OX=9606 GN=ATP5PD PE=1 SV=3	23	6	6	18.5	43	161	5.3	1	1	1	2	3	3	1	1	1	2	3	3
High	Q86V15	Zinc finger protein castor homolog 1 OS=Homo sapiens OX=9606 GN=CASZ1 PE=1 SV=4	23	12	12	189.9	10	1759	7.03	7	9	7				7	9	7			
High	P28799	Progranulin OS=Homo sapiens OX=9606 GN=GRN PE=1 SV=2	23	5	5	63.5	16	593	6.83	2	2		1	2	1	2	2		1	2	1
High	Q8NI27	THO complex subunit 2 OS=Homo sapiens OX=9606 GN=THOC2 PE=1 SV=2	23	10	10	182.7	7	1593	8.44	2		1	1	3	3	2		1	1	3	3
High	Q14966	Zinc finger protein 638 OS=Homo sapiens OX=9606 GN=ZNF638 PE=1 SV=2	23	10	10	220.5	7	1978	6.38	2	3	2	2	1	2	2	3	2	2	1	2
High	Q9Y3E7	Charged multivesicular body protein 3 OS=Homo sapiens OX=9606 GN=CHMP3 PE=1 SV=3	23	6	6	25.1	34	222	5.12	2	3	2				2	3	2			
High	P40938	Replication factor C subunit 3 OS=Homo sapiens OX=9606 GN=RFC3 PE=1 SV=2	23	5	5	40.5	22	356	8.34	4	4	4			1	4	4	3			1
High	Q9HB71	Calcyclin-binding protein OS=Homo sapiens OX=9606 GN=CACYBP PE=1 SV=2	23	4	4	26.2	22	228	8.25	1	1	2	3	2	3	1	1	2	3	2	3
High	P50452	Serpin B8 OS=Homo sapiens OX=9606 GN=SERPINB8 PE=1 SV=2	23	6	4	42.7	15	374	5.57	2	2	2	1	5	4	2	2	2	1	4	4
High	Q9Y2Z0	Protein SGT1 homolog OS=Homo sapiens OX=9606 GN=SUGT1 PE=1 SV=3	23	8	8	41	27	365	5.16	1		3	2	3	2	1		3	2	3	2
High	O95232	Luc7-like protein 3 OS=Homo sapiens OX=9606 GN=LUC7L3 PE=1 SV=2	23	5	5	51.4	13	432	9.79		1	1	5	5	4		1	1	5	4	4
High	Q9NX63	MICOS complex subunit MIC19 OS=Homo sapiens OX=9606 GN=CHCHD3 PE=1 SV=1	23	5	5	26.1	25	227	8.28	1	1	1	2	3	3	1	1	1	2	3	3
High	P04439	HLA class I histocompatibility antigen, A alpha chain OS=Homo sapiens OX=9606 GN=HLA-A PE=1 SV=2	23	5	4	40.8	22	365	6	3	3	1	2	3	2	2	2	1	2	2	2
High	P55084	Trifunctional enzyme subunit beta, mitochondrial OS=Homo sapiens OX=9606 GN=HADHB PE=1 SV=3	23	8	8	51.3	23	474	9.41	2	3	2	3	6	3	2	3	2	3	6	3
High	P41212	Transcription factor ETV6 OS=Homo sapiens OX=9606 GN=ETV6 PE=1 SV=1	23	6	6	53	11	452	7.43	1	1	1	2	2	2	1	1	1	2	2	2
High	P24666	Low molecular weight phosphotyrosine protein phosphatase OS=Homo sapiens OX=9606 GN=ACP1 PE=1 SV=3	23	5	5	18	47	158	6.74	2	2	3	1	2	1	2	2	3	1	2	1
High	O43290	U4/U6.U5 tri-snRNP-associated protein 1 OS=Homo sapiens OX=9606 GN=SART1 PE=1 SV=1	23	9	9	90.2	14	800	6.13	2	1		4	2	3	2	1		4	2	3
High	O43813	Glutathione S-transferase LANCL1 OS=Homo sapiens OX=9606 GN=LANCL1 PE=1 SV=1	23	6	6	45.3	16	399	7.75	2		3	1	3	3	2		2	1	3	2
High	O75153	Clustered mitochondria protein homolog OS=Homo sapiens OX=9606 GN=CLUH PE=1 SV=2	23	9	9	146.6	8	1309	6.13	2	2	3		5		2	2	3		5	
High	O00170	AH receptor-interacting protein OS=Homo sapiens OX=9606 GN=AIP PE=1 SV=3	23	6	6	37.6	24	330	6.42	1	1	1	2	3	2	1	1	1	2	3	2
High	Q9Y2R4	Probable ATP-dependent RNA helicase DDX52 OS=Homo sapiens OX=9606 GN=DDX52 PE=1 SV=4	23	6	5	67.4	12	599	9.67	1	1	1	2	2	2	1	1	1	2	2	2
High	P50750	Cyclin-dependent kinase 9 OS=Homo sapiens OX=9606 GN=CDK9 PE=1 SV=3	23	5	4	42.8	17	372	8.79	2	4	3	3	3	2	2	3	2	2	2	2
High	P18887	DNA repair protein XRCC1 OS=Homo sapiens OX=9606 GN=XRCC1 PE=1 SV=3	23	5	5	69.5	8	633	6.28	2	2	1	3	3	3	2	2	1	3	3	3
High	Q8IZ83	Aldehyde dehydrogenase family 16 member A1 OS=Homo sapiens OX=9606 GN=ALDH16A1 PE=1 SV=2	22	6	6	85.1	8	802	6.79		1	3	1	3	4		1	3	1	3	4
High	O95747	Serine/threonine-protein kinase OSR1 OS=Homo sapiens OX=9606 GN=OXSR1 PE=1 SV=1	22	6	2	58	11	527	6.43	2	3	2		2	1	2	3	2		2	1
High	O43865	S-adenosylhomocysteine hydrolase-like protein 1 OS=Homo sapiens OX=9606 GN=AHCYL1 PE=1 SV=2	22	6	5	58.9	11	530	6.89	1	1	1	1	1	1	1	1	1	1	1	1
High	Q01581	Hydroxymethylglutaryl-CoA synthase, cytoplasmic OS=Homo sapiens OX=9606 GN=HMGCS1 PE=1 SV=2	22	6	5	57.3	13	520	5.41	1	2	3	3		4	1	2	3	3		4
High	P61421	V-type proton ATPase subunit d 1 OS=Homo sapiens OX=9606 GN=ATP6V0D1 PE=1 SV=1	22	7	7	40.3	28	351	5	4	3	2				4	3	2			
High	P09972	Fructose-bisphosphate aldolase C OS=Homo sapiens OX=9606 GN=ALDOC PE=1 SV=2	22	3	2	39.4	9	364	6.87	2	3	3	1	3	2	2	3	3	1	3	2
High	O14929	Histone acetyltransferase type B catalytic subunit OS=Homo sapiens OX=9606 GN=HAT1 PE=1 SV=2	22	4	4	49.5	14	419	5.69	2	2	1	3	2	3	2	2	1	3	2	3
High	Q86U86	Protein polybromo-1 OS=Homo sapiens OX=9606 GN=PBRM1 PE=1 SV=1	22	8	8	192.8	6	1689	6.89				3	5	4				3	5	4
High	P05386	60S acidic ribosomal protein P1 OS=Homo sapiens OX=9606 GN=RPLP1 PE=1 SV=1	22	3	2	11.5	67	114	4.32	3	3	4				1	1	2			
High	Q9BY32	Inosine triphosphate pyrophosphatase OS=Homo sapiens OX=9606 GN=ITPA PE=1 SV=2	22	5	5	21.4	45	194	5.66	4	4	4	1	1	1	4	4	3	1	1	1
High	Q53GQ0	Very-long-chain 3-oxoacyl-CoA reductase OS=Homo sapiens OX=9606 GN=HSD17B12 PE=1 SV=2	22	8	8	34.3	30	312	9.32	5	5	3				5	5	3			
High	Q8N6T3	ADP-ribosylation factor GTPase-activating protein 1 OS=Homo sapiens OX=9606 GN=ARFGAP1 PE=1 SV=2	22	5	5	44.6	16	406	5.66	1	1	2	3	2	3	1	1	2	3	2	3
High	P35244	Replication protein A 14 kDa subunit OS=Homo sapiens OX=9606 GN=RPA3 PE=1 SV=1	22	4	4	13.6	51	121	5.08	4	5	7	1	1	1	3	4	4	1	1	1
High	Q96EE3	Nucleoporin SEH1 OS=Homo sapiens OX=9606 GN=SEH1L PE=1 SV=3	22	6	6	39.6	23	360	8.09	1	2	2	2	1	2	1	2	2	2	1	2
High	Q12873	Chromodomain-helicase-DNA-binding protein 3 OS=Homo sapiens OX=9606 GN=CHD3 PE=1 SV=3	22	5	1	226.5	4	2000	7.3	1	2	4	1	3	1	1	2	4	1	3	1
High	Q9BYG3	MKI67 FHA domain-interacting nucleolar phosphoprotein OS=Homo sapiens OX=9606 GN=NIFK PE=1 SV=1	22	5	5	34.2	23	293	9.88	1	1	1	2	3	2	1	1	1	2	3	2
High	Q04323	UBX domain-containing protein 1 OS=Homo sapiens OX=9606 GN=UBXN1 PE=1 SV=2	22	4	4	33.3	23	297	5.25	3	1	2	1	3	2	3	1	2	1	3	2
High	O76003	Glutaredoxin-3 OS=Homo sapiens OX=9606 GN=GLRX3 PE=1 SV=2	22	4	4	37.4	13	335	5.39	1	4	2	2	2	2	1	3	2	2	2	2
High	P02686	Myelin basic protein OS=Homo sapiens OX=9606 GN=MBP PE=1 SV=3	22	3	3	33.1	11	304	9.79				6	8	8				3	3	3
High	P17931	Galectin-3 OS=Homo sapiens OX=9606 GN=LGALS3 PE=1 SV=5	22	5	5	26.1	22	250	8.56	4	3	4				4	3	4			
High	P43897	Elongation factor Ts, mitochondrial OS=Homo sapiens OX=9606 GN=TSFM PE=1 SV=2	22	6	6	35.4	24	325	8.38	2	1	3	2		2	2	1	3	2		2
High	Q9BTT6	Leucine-rich repeat-containing protein 1 OS=Homo sapiens OX=9606 GN=LRRC1 PE=1 SV=1	22	4	2	59.2	11	524	5.02	4	4	4		1		4	4	4		1	
High	Q96RS6	NudC domain-containing protein 1 OS=Homo sapiens OX=9606 GN=NUDCD1 PE=1 SV=2	22	8	8	66.7	16	583	5.11	1	1		2	2	1	1	1		2	2	1
High	Q99798	Aconitate hydratase, mitochondrial OS=Homo sapiens OX=9606 GN=ACO2 PE=1 SV=2	22	7	7	85.4	13	780	7.61	2	2	2	3	2	2	2	2	2	3	2	2
High	Q8N1N4	Keratin, type II cytoskeletal 78 OS=Homo sapiens OX=9606 GN=KRT78 PE=1 SV=2	22	4	1	56.8	7	520	6.02	3	3	5	1	1	1	2	2	4	1	1	1
High	Q86UP2	Kinectin OS=Homo sapiens OX=9606 GN=KTN1 PE=1 SV=1	22	7	7	156.2	6	1357	5.64	2	1	2	1	2	1	2	1	2	1	2	1
High	Q9UJZ1	Stomatin-like protein 2, mitochondrial OS=Homo sapiens OX=9606 GN=STOML2 PE=1 SV=1	22	7	7	38.5	29	356	7.39	2	1	4				2	1	4			
High	Q06203	Amidophosphoribosyltransferase OS=Homo sapiens OX=9606 GN=PPAT PE=1 SV=1	22	6	6	57.4	13	517	6.76	1	2	4	2	2	1	1	2	4	2	2	1
High	Q8IWZ3	Ankyrin repeat and KH domain-containing protein 1 OS=Homo sapiens OX=9606 GN=ANKHD1 PE=1 SV=1	21	7	1	269.3	3	2542	5.73			1	2	2	3			1	2	2	3
High	P11172	Uridine 5'-monophosphate synthase OS=Homo sapiens OX=9606 GN=UMPS PE=1 SV=1	21	6	6	52.2	10	480	7.24	1		3		2		1		3		2	
High	Q96T51	RUN and FYVE domain-containing protein 1 OS=Homo sapiens OX=9606 GN=RUFY1 PE=1 SV=2	21	7	7	79.8	10	708	5.74		2	2	1	2			2	2	1	2	
High	P09382	Galectin-1 OS=Homo sapiens OX=9606 GN=LGALS1 PE=1 SV=2	21	4	4	14.7	28	135	5.5		1	2	2	3	4		1	2	2	3	4
High	O00483	Cytochrome c oxidase subunit NDUFA4 OS=Homo sapiens OX=9606 GN=NDUFA4 PE=1 SV=1	21	3	3	9.4	32	81	9.38	2	2	2	3	3	3	2	2	2	3	3	3
High	Q15050	Ribosome biogenesis regulatory protein homolog OS=Homo sapiens OX=9606 GN=RRS1 PE=1 SV=2	21	6	6	41.2	20	365	10.7	1	1	1	1	2	1	1	1	1	1	2	1
High	O60763	General vesicular transport factor p115 OS=Homo sapiens OX=9606 GN=USO1 PE=1 SV=2	21	8	8	107.8	10	962	4.91	2	1	3	1		1	2	1	3	1		1
High	P20592	Interferon-induced GTP-binding protein Mx2 OS=Homo sapiens OX=9606 GN=MX2 PE=1 SV=1	21	10	10	82	15	715	8.76				6	8	6				6	8	6
High	P54198	Protein HIRA OS=Homo sapiens OX=9606 GN=HIRA PE=1 SV=2	21	8	8	111.8	10	1017	8.07	4	6	3		1	1	4	6	3		1	1
High	P82673	28S ribosomal protein S35, mitochondrial OS=Homo sapiens OX=9606 GN=MRPS35 PE=1 SV=1	21	6	6	36.8	27	323	8.24	1	1	3	1	3	2	1	1	3	1	3	2
High	Q15599	Na(+)/H(+) exchange regulatory cofactor NHE-RF2 OS=Homo sapiens OX=9606 GN=SLC9A3R2 PE=1 SV=2	21	6	6	37.4	23	337	7.93		2	2	1	3	1		2	2	1	3	1
High	Q96A33	PAT complex subunit CCDC47 OS=Homo sapiens OX=9606 GN=CCDC47 PE=1 SV=1	21	5	5	55.8	14	483	4.87	3	5	2				3	3	2			
High	Q9Y221	60S ribosome subunit biogenesis protein NIP7 homolog OS=Homo sapiens OX=9606 GN=NIP7 PE=1 SV=1	21	6	6	20.4	38	180	8.51	1	1	1	4	3	2	1	1	1	4	3	2
High	Q00534	Cyclin-dependent kinase 6 OS=Homo sapiens OX=9606 GN=CDK6 PE=1 SV=1	21	4	3	36.9	11	326	6.46	2	3	3	3	2	2	2	2	2	2	1	2
High	O14908	PDZ domain-containing protein GIPC1 OS=Homo sapiens OX=9606 GN=GIPC1 PE=1 SV=2	21	7	7	36	26	333	6.28		1	3		1	1		1	3		1	1
High	P25788	Proteasome subunit alpha type-3 OS=Homo sapiens OX=9606 GN=PSMA3 PE=1 SV=2	21	4	4	28.4	16	255	5.33			2	4	3	3			2	4	3	3
High	P19623	Spermidine synthase OS=Homo sapiens OX=9606 GN=SRM PE=1 SV=1	21	8	8	33.8	38	302	5.49	1	1	2	1		2	1	1	2	1		2
High	P51153	Ras-related protein Rab-13 OS=Homo sapiens OX=9606 GN=RAB13 PE=1 SV=1	21	4	2	22.8	21	203	9.19	2	3	2	2	2	2	2	2	2	2	2	2
High	O43776	Asparagine--tRNA ligase, cytoplasmic OS=Homo sapiens OX=9606 GN=NARS1 PE=1 SV=1	21	8	8	62.9	19	548	6.25		2	4	1	1	1		2	4	1	1	1
High	Q9UJA5	tRNA (adenine(58)-N(1))-methyltransferase non-catalytic subunit TRM6 OS=Homo sapiens OX=9606 GN=TRMT6 PE=1 SV=1	21	6	6	55.8	15	497	7.55	2	2	1	2	1	3	2	2	1	2	1	3
High	Q96QR8	Transcriptional activator protein Pur-beta OS=Homo sapiens OX=9606 GN=PURB PE=1 SV=3	21	5	5	33.2	27	312	5.43	1	1	1	1	2	1	1	1	1	1	2	1
High	P04637	Cellular tumor antigen p53 OS=Homo sapiens OX=9606 GN=TP53 PE=1 SV=4	21	7	7	43.6	27	393	6.79	1		1	2	3	2	1		1	2	3	2
High	Q9UKF6	Cleavage and polyadenylation specificity factor subunit 3 OS=Homo sapiens OX=9606 GN=CPSF3 PE=1 SV=1	21	5	5	77.4	9	684	5.6	2	1	1	2	3	2	2	1	1	2	3	2
High	P13861	cAMP-dependent protein kinase type II-alpha regulatory subunit OS=Homo sapiens OX=9606 GN=PRKAR2A PE=1 SV=2	21	5	5	45.5	15	404	5.07	1	1	2	1	2	2	1	1	2	1	2	2
High	P17029	Zinc finger protein with KRAB and SCAN domains 1 OS=Homo sapiens OX=9606 GN=ZKSCAN1 PE=1 SV=3	21	7	7	63.6	17	563	7.05	4	5	4				4	5	4			
High	P48735	Isocitrate dehydrogenase [NADP], mitochondrial OS=Homo sapiens OX=9606 GN=IDH2 PE=1 SV=2	21	7	7	50.9	19	452	8.69	2	2	5	1	1	1	2	2	5	1	1	1
High	P78362	SRSF protein kinase 2 OS=Homo sapiens OX=9606 GN=SRPK2 PE=1 SV=3	21	8	6	77.5	16	688	4.97	1	1	1	4	3	3	1	1	1	4	3	3
High	P46782	40S ribosomal protein S5 OS=Homo sapiens OX=9606 GN=RPS5 PE=1 SV=4	21	4	4	22.9	23	204	9.72	1	2	2	1	1	1	1	2	2	1	1	1
High	Q5H9R7	Serine/threonine-protein phosphatase 6 regulatory subunit 3 OS=Homo sapiens OX=9606 GN=PPP6R3 PE=1 SV=2	21	7	7	97.6	10	873	4.6	2	2	1	1	2	2	2	2	1	1	2	2

High	P61586	Transforming protein RhoA OS=Homo sapiens OX=9606 GN=RHOA PE=1 SV=1	21	4	3	21.8	21	193	6.1	3	3	2	2	2	2	2	2	1	2	2	2
High	P31146	Coronin-1A OS=Homo sapiens OX=9606 GN=CORO1A PE=1 SV=4	21	5	4	51	11	461	6.68	2	2	2	3	4	3	2	2	2	3	4	3
High	P35613	Basigin OS=Homo sapiens OX=9606 GN=BSG PE=1 SV=2	21	5	5	42.2	18	385	5.66	3	3	4		1		3	3	4		1	
High	Q7L014	Probable ATP-dependent RNA helicase DDX46 OS=Homo sapiens OX=9606 GN=DDX46 PE=1 SV=2	21	10	10	117.3	10	1031	9.29				2	5	4				2	5	4
High	P40616	ADP-ribosylation factor-like protein 1 OS=Homo sapiens OX=9606 GN=ARL1 PE=1 SV=1	21	6	6	20.4	53	181	5.72	5	2	5	1	1	1	5	2	5	1	1	1
High	P47914	60S ribosomal protein L29 OS=Homo sapiens OX=9606 GN=RPL29 PE=1 SV=2	21	2	2	17.7	14	159	11.66	1	1	2	2	3	3	1	1	1	2	2	2
High	Q14165	Malectin OS=Homo sapiens OX=9606 GN=MLEC PE=1 SV=1	20	6	6	32.2	24	292	5.41			3			1			3			1
High	Q9P2K3	REST corepressor 3 OS=Homo sapiens OX=9606 GN=RCOR3 PE=1 SV=2	20	5	1	55.5	11	495	8.27	1		1	1	2	1	1		1	1	2	1
High	P36507	Dual specificity mitogen-activated protein kinase kinase 2 OS=Homo sapiens OX=9606 GN=MAP2K2 PE=1 SV=1	20	5	3	44.4	15	400	6.55	2	2	2	1	2	1	2	2	2	1	2	1
High	P61513	60S ribosomal protein L37a OS=Homo sapiens OX=9606 GN=RPL37A PE=1 SV=2	20	3	3	10.3	41	92	10.43	1	1	2	2	3	2	1	1	2	2	2	2
High	Q9BRJ6	Uncharacterized protein C7orf50 OS=Homo sapiens OX=9606 GN=C7orf50 PE=1 SV=1	20	5	5	22.1	42	194	9.64			1	5	4	3			1	4	4	3
High	O95486	Protein transport protein Sec24A OS=Homo sapiens OX=9606 GN=SEC24A PE=1 SV=2	20	7	5	119.7	6	1093	7.66				2	3	2				2	3	2
High	Q9NWH9	SAFB-like transcription modulator OS=Homo sapiens OX=9606 GN=SLTM PE=1 SV=2	20	6	6	117.1	8	1034	7.87	2	2	3	1	3	1	2	2	3	1	3	1
High	Q9H4I2	Zinc fingers and homeoboxes protein 3 OS=Homo sapiens OX=9606 GN=ZHX3 PE=1 SV=3	20	7	6	104.6	12	956	6.07				3		2				3		2
High	Q9UKL0	REST corepressor 1 OS=Homo sapiens OX=9606 GN=RCOR1 PE=1 SV=2	20	7	3	53.3	18	485	7.03	1		1	1	1	1	1		1	1	1	1
High	Q13576	Ras GTPase-activating-like protein IQGAP2 OS=Homo sapiens OX=9606 GN=IQGAP2 PE=1 SV=4	20	8	6	180.5	6	1575	5.64	1	2	2	1	1	1	1	1	2	1	1	1
High	P41743	Protein kinase C iota type OS=Homo sapiens OX=9606 GN=PRKCI PE=1 SV=2	20	6	6	68.2	13	596	5.85	2	1	2	2	1	2	2	1	2	2	1	2
High	P55036	26S proteasome non-ATPase regulatory subunit 4 OS=Homo sapiens OX=9606 GN=PSMD4 PE=1 SV=1	20	7	7	40.7	31	377	4.79	1			4	4	4	1			4	4	4
High	O00233	26S proteasome non-ATPase regulatory subunit 9 OS=Homo sapiens OX=9606 GN=PSMD9 PE=1 SV=3	20	6	6	24.7	27	223	6.95	2	1	1	2	1	2	2	1	1	2	1	2
High	Q15020	Squamous cell carcinoma antigen recognized by T-cells 3 OS=Homo sapiens OX=9606 GN=SART3 PE=1 SV=1	20	5	5	109.9	6	963	5.57			1	3	1	2			1	3	1	2
High	P42285	Exosome RNA helicase MTR4 OS=Homo sapiens OX=9606 GN=MTREX PE=1 SV=3	20	9	9	117.7	9	1042	6.52	1			3	2	2	1			3	2	2
High	Q9BT17	Mitochondrial ribosome-associated GTPase 1 OS=Homo sapiens OX=9606 GN=MTG1 PE=1 SV=2	20	3	3	37.2	11	334	9.47	3	3	2	2	2	1	3	3	2	2	2	1
High	O75351	Vacuolar protein sorting-associated protein 4B OS=Homo sapiens OX=9606 GN=VPS4B PE=1 SV=2	20	6	4	49.3	18	444	7.23	1	1	3	1	2	2	1	1	3	1	2	2
High	P23258	Tubulin gamma-1 chain OS=Homo sapiens OX=9606 GN=TUBG1 PE=1 SV=2	20	6	6	51.1	22	451	6.14	5	4	5				4	3	4			
High	P10301	Ras-related protein R-Ras OS=Homo sapiens OX=9606 GN=RRAS PE=1 SV=1	20	4	2	23.5	21	218	6.93	3	3	3	1	1	1	3	3	2	1	1	1
High	P61604	10 kDa heat shock protein, mitochondrial OS=Homo sapiens OX=9606 GN=HSPE1 PE=1 SV=2	20	4	4	10.9	38	102	8.92	3	2	4	1	2	3	3	2	4	1	2	3
High	P13807	Glycogen [starch] synthase, muscle OS=Homo sapiens OX=9606 GN=GYS1 PE=1 SV=2	20	5	5	83.7	8	737	6.18	1		1	2	3	2	1		1	2	3	2
High	O94808	Glutamine--fructose-6-phosphate aminotransferase [isomerizing] 2 OS=Homo sapiens OX=9606 GN=GFPT2 PE=1 SV=3	20	4	1	76.9	6	682	7.37	3	3	3	2	1	2	3	3	3	2	1	2
High	P43307	Translocon-associated protein subunit alpha OS=Homo sapiens OX=9606 GN=SSR1 PE=1 SV=3	20	4	4	32.2	21	286	4.49	2	2	4	2		2	2	2	4	2		2
High	P61221	ATP-binding cassette sub-family E member 1 OS=Homo sapiens OX=9606 GN=ABCE1 PE=1 SV=1	20	9	9	67.3	15	599	8.34					3	1					3	1
High	P61626	Lysozyme C OS=Homo sapiens OX=9606 GN=LYZ PE=1 SV=1	20	3	3	16.5	18	148	9.16	3	1	2	2	3	3	3	1	2	2	3	2
High	Q9BW19	Kinesin-like protein KIFC1 OS=Homo sapiens OX=9606 GN=KIFC1 PE=1 SV=2	20	6	6	73.7	11	673	8.98	1			3	2	3	1			3	2	3
High	Q96SI9	Spermatid perinuclear RNA-binding protein OS=Homo sapiens OX=9606 GN=STRBP PE=1 SV=1	20	3	1	73.6	4	672	8.72	2	2	1	2	2	2	2	2	1	2	2	2
High	P31949	Protein S100-A11 OS=Homo sapiens OX=9606 GN=S100A11 PE=1 SV=2	20	4	4	11.7	43	105	7.12	3	3	3	2	1	2	2	2	2	2	1	2
High	P43686	26S proteasome regulatory subunit 6B OS=Homo sapiens OX=9606 GN=PSMC4 PE=1 SV=2	20	9	8	47.3	24	418	5.21	1			2	6	3	1			2	6	3
High	Q9BQG0	Myb-binding protein 1A OS=Homo sapiens OX=9606 GN=MYBBP1A PE=1 SV=2	20	8	8	148.8	7	1328	9.28				1		2				1		2
High	O95163	Elongator complex protein 1 OS=Homo sapiens OX=9606 GN=ELP1 PE=1 SV=3	20	6	6	150.2	6	1332	5.94	2	2	1	1	2	2	2	2	1	1	2	2
High	Q9HAV7	GrpE protein homolog 1, mitochondrial OS=Homo sapiens OX=9606 GN=GRPEL1 PE=1 SV=2	20	4	4	24.3	23	217	8.12	2	3	2	1	1	1	2	3	2	1	1	1
High	P52294	Importin subunit alpha-5 OS=Homo sapiens OX=9606 GN=KPNA1 PE=1 SV=3	20	5	2	60.2	13	538	5.01	2	3	1	2	3	1	2	3	1	2	3	1
High	P35606	Coatomer subunit beta' OS=Homo sapiens OX=9606 GN=COPB2 PE=1 SV=2	20	6	6	102.4	9	906	5.27	3	5	3		1	1	3	4	3		1	1
High	Q14157	Ubiquitin-associated protein 2-like OS=Homo sapiens OX=9606 GN=UBAP2L PE=1 SV=2	20	7	7	114.5	10	1087	7.11	4	5	4				4	5	4			
High	Q9NUL3	Double-stranded RNA-binding protein Staufen homolog 2 OS=Homo sapiens OX=9606 GN=STAU2 PE=1 SV=2	20	7	5	62.6	15	570	9.61		1	2	3	2	1		1	2	3	2	1
High	Q8IXI2	Mitochondrial Rho GTPase 1 OS=Homo sapiens OX=9606 GN=RHOT1 PE=1 SV=2	20	5	4	70.7	9	618	6.27		1	1	3	4	3		1	1	3	4	3
High	Q9Y6Y8	SEC23-interacting protein OS=Homo sapiens OX=9606 GN=SEC23IP PE=1 SV=1	20	6	6	111	9	1000	5.54	2	1	2		1	2	2	1	2		1	2
High	P82930	28S ribosomal protein S34, mitochondrial OS=Homo sapiens OX=9606 GN=MRPS34 PE=1 SV=2	20	5	5	25.6	26	218	9.98	1	1	2	1	2	1	1	1	2	1	2	1
High	P61289	Proteasome activator complex subunit 3 OS=Homo sapiens OX=9606 GN=PSME3 PE=1 SV=1	20	4	4	29.5	17	254	5.95	3	2	2	1	2	1	3	2	2	1	2	1
High	Q5T6F2	Ubiquitin-associated protein 2 OS=Homo sapiens OX=9606 GN=UBAP2 PE=1 SV=1	20	7	7	117	9	1119	7.34			1	1	3	1			1	1	3	1
High	P30084	Enoyl-CoA hydratase, mitochondrial OS=Homo sapiens OX=9606 GN=ECHS1 PE=1 SV=4	20	7	7	31.4	33	290	8.07	4	3	3		2	1	4	3	3		2	1
High	Q9NYB9	Abl interactor 2 OS=Homo sapiens OX=9606 GN=ABI2 PE=1 SV=1	19	5	2	55.6	13	513	6.16	1	1	1	1	1	1	1	1	1	1	1	1
High	P78417	Glutathione S-transferase omega-1 OS=Homo sapiens OX=9606 GN=GSTO1 PE=1 SV=2	19	4	4	27.5	16	241	6.6	2	2	1	1	2	2	2	2	1	1	2	2
High	Q9BXJ9	N-alpha-acetyltransferase 15, NatA auxiliary subunit OS=Homo sapiens OX=9606 GN=NAA15 PE=1 SV=1	19	6	6	101.2	8	866	7.42	2	2	1	2	2	2	2	2	1	2	2	2
High	Q08495	Dematin OS=Homo sapiens OX=9606 GN=DMTN PE=1 SV=3	19	6	6	45.5	18	405	8.88	2	2	3				2	2	3			
High	Q13257	Mitotic spindle assembly checkpoint protein MAD2A OS=Homo sapiens OX=9606 GN=MAD2L1 PE=1 SV=1	19	4	4	23.5	17	205	5.08	1	2	3	1	3	2	1	2	3	1	3	2
High	O43432	Eukaryotic translation initiation factor 4 gamma 3 OS=Homo sapiens OX=9606 GN=EIF4G3 PE=1 SV=2	19	9	6	176.5	7	1585	5.38		2	2	1	1	1		2	2	1	1	1
High	Q99459	Cell division cycle 5-like protein OS=Homo sapiens OX=9606 GN=CDC5L PE=1 SV=2	19	8	8	92.2	15	802	8.18	3	2	3	1	2	1	3	2	3	1	2	1
High	Q08209	Serine/threonine-protein phosphatase 2B catalytic subunit alpha isoform OS=Homo sapiens OX=9606 GN=PPP3CA PE=1 SV=1	19	5	5	58.7	11	521	5.86	1	1	1	1	2	2	1	1	1	1	2	2
High	Q96T58	Msx2-interacting protein OS=Homo sapiens OX=9606 GN=SPEN PE=1 SV=1	19	3	3	402	1	3664	7.64	3	1	3	2			2	1	2	1		
High	Q7Z417	Nuclear fragile X mental retardation-interacting protein 2 OS=Homo sapiens OX=9606 GN=NUFIP2 PE=1 SV=1	19	7	7	76.1	17	695	8.7	1	1	2		3	2	1	1	2		3	2
High	P28072	Proteasome subunit beta type-6 OS=Homo sapiens OX=9606 GN=PSMB6 PE=1 SV=4	19	4	4	25.3	22	239	4.92	2			3	3	2	2			3	3	2
High	Q5GLZ8	Probable E3 ubiquitin-protein ligase HERC4 OS=Homo sapiens OX=9606 GN=HERC4 PE=1 SV=1	19	5	5	118.5	7	1057	6.19	1	2	2		2	2	1	2	2		2	2
High	Q9Y4K1	Beta/gamma crystallin domain-containing protein 1 OS=Homo sapiens OX=9606 GN=CRYBG1 PE=1 SV=3	19	8	8	188.6	7	1723	5.86	5	7	3				5	7	3			
High	O15371	Eukaryotic translation initiation factor 3 subunit D OS=Homo sapiens OX=9606 GN=EIF3D PE=1 SV=1	19	5	5	63.9	16	548	6.05	3	3	4		1		3	3	4	1		
High	Q5BKZ1	DBIRD complex subunit ZNF326 OS=Homo sapiens OX=9606 GN=ZNF326 PE=1 SV=2	19	5	5	65.6	11	582	5.15	2	2	3	1	2		2	2	2	1	2	
High	Q7LBR1	Charged multivesicular body protein 1b OS=Homo sapiens OX=9606 GN=CHMP1B PE=1 SV=1	19	3	3	22.1	12	199	8.1	2	3	3	2	2	2	2	2	2	2	2	2
High	P52888	Thimet oligopeptidase OS=Homo sapiens OX=9606 GN=THOP1 PE=1 SV=2	19	6	6	78.8	10	689	6.05	1	1		2	3	2	1	1		2	3	2
High	O75475	PC4 and SFRS1-interacting protein OS=Homo sapiens OX=9606 GN=PSIP1 PE=1 SV=1	19	9	9	60.1	19	530	9.13	4	2	5		2		3	2	5		2	
High	P00973	2'-5'-oligoadenylate synthase 1 OS=Homo sapiens OX=9606 GN=OAS1 PE=1 SV=4	19	5	5	46	13	400	8.22		1	1	3	1	3		1	1	3	1	3
High	Q8N1W1	Rho guanine nucleotide exchange factor 28 OS=Homo sapiens OX=9606 GN=ARHGEF28 PE=1 SV=3	19	8	8	191.8	6	1705	6.04	3	3	8				3	3	8			
High	Q0JRZ9	F-BAR domain only protein 2 OS=Homo sapiens OX=9606 GN=FCHO2 PE=1 SV=2	19	6	6	88.9	8	810	6.86	1	2	1	1		1	1	2	1	1		1
High	Q14789	Golgin subfamily B member 1 OS=Homo sapiens OX=9606 GN=GOLGB1 PE=1 SV=2	19	9	8	375.8	3	3259	5		1				1		1				1
High	Q9BZH6	WD repeat-containing protein 11 OS=Homo sapiens OX=9606 GN=WDR11 PE=1 SV=1	19	9	9	136.6	10	1224	6.92		1	1		2			1	1		2	
High	O60333	Kinesin-like protein KIF1B OS=Homo sapiens OX=9606 GN=KIF1B PE=1 SV=5	19	6	5	204.3	5	1816	5.6	4	4	4		2		4	4	4		2	
High	Q9BYJ9	YTH domain-containing family protein 1 OS=Homo sapiens OX=9606 GN=YTHDF1 PE=1 SV=1	19	5	4	60.8	9	559	8.79	4	4	2	1	2		4	4	2	1	2	
High	P62834	Ras-related protein Rap-1A OS=Homo sapiens OX=9606 GN=RAP1A PE=1 SV=1	19	4	2	21	28	184	6.67	2		1	1	3	1	2		1	1	3	1
High	P61020	Ras-related protein Rab-5B OS=Homo sapiens OX=9606 GN=RAB5B PE=1 SV=1	19	5	2	23.7	27	215	8.13	2	3	2	1	2	1	2	3	2	1	2	1
High	O43889	Cyclic AMP-responsive element-binding protein 3 OS=Homo sapiens OX=9606 GN=CREB3 PE=1 SV=2	19	4	4	41.4	14	371	4.91				3	2	3				3	2	3
High	Q9NVE7	4'-phosphopantetheine phosphatase OS=Homo sapiens OX=9606 GN=PANK4 PE=1 SV=1	19	8	8	85.9	12	773	6.28	5	4	6				5	4	6			
High	Q8IXB1	DnaJ homolog subfamily C member 10 OS=Homo sapiens OX=9606 GN=DNAJC10 PE=1 SV=2	19	4	4	91	7	793	7.18	1	2	1	2	1	3	1	2	1	2	1	3
High	Q13363	C-terminal-binding protein 1 OS=Homo sapiens OX=9606 GN=CTBP1 PE=1 SV=2	19	4	2	47.5	10	440	6.77	1	1	2	2	1	2	1	1	2	2	1	2
High	Q6DD88	Atlastin-3 OS=Homo sapiens OX=9606 GN=ATL3 PE=1 SV=1	19	7	7	60.5	21	541	5.66	5	3	5				5	3	4			
High	Q96PY5	Formin-like protein 2 OS=Homo sapiens OX=9606 GN=FMNL2 PE=1 SV=3	19	10	10	123.2	10	1086	7.4	2	3	2	1		1	2	3	2	1		1
High	P49841	Glycogen synthase kinase-3 beta OS=Homo sapiens OX=9606 GN=GSK3B PE=1 SV=2	19	4	4	46.7	15	420	8.78	2	2	2	3	2	2	2	2	2	3	2	2
High	P28340	DNA polymerase delta catalytic subunit OS=Homo sapiens OX=9606 GN=POLD1 PE=1 SV=2	19	7	6	123.6	7	1107	7.03	2	2	2			1	2	2	2			1
High	P40763	Signal transducer and activator of transcription 3 OS=Homo sapiens OX=9606 GN=STAT3 PE=1 SV=2	18	6	6	88	13	770	6.3	3	2	3		3		3	2	3		3	
High	P12763	SWISS-PROT:P12763 (Bos taurus) Alpha-2-HS-glycoprotein precursor	18	4	4	38.4	13	359	5.5	1	1	2	1		1	1	1	2	1		1
High	P52298	Nuclear cap-binding protein subunit 2 OS=Homo sapiens OX=9606 GN=NCBP2 PE=1 SV=1	18	3	3	18	17	156	8.21	1	1	3	2	2	2	1	1	2	2	2	2
High	P82675	28S ribosomal protein S5, mitochondrial OS=Homo sapiens OX=9606 GN=MRPS5 PE=1 SV=2	18	5	5	48	11	430	9.92	1	2		3	1	2	1	2		3	1	2
High	P42685	Tyrosine-protein kinase FRK OS=Homo sapiens OX=9606 GN=FRK PE=1 SV=1	18	3	1	58.2	5	505	6.67	2	3	1	1	2	2	2	2	1	1	2	2
High	Q9Y508	E3 ubiquitin-protein ligase RNF114 OS=Homo sapiens OX=9606 GN=RNF114 PE=1 SV=1	18	5	5	25.7	26	228	7.25		1	1	2	3	2		1	1	2	3	2
High	Q9H0C8	Integrin-linked kinase-associated serine/threonine phosphatase 2C OS=Homo sapiens OX=9606 GN=ILKAP PE=1 SV=1	18	6	6	42.9	15	392	7.09	1	1	2	3		2	1	1	2	3		2
High	O60218	Aldo-keto reductase family 1 member B10 OS=Homo sapiens OX=9606 GN=AKR1B10 PE=1 SV=2	18	5	5	36	16	316	7.84	2	3	2	1	2	2	2	3	2	1	2	2
High	Q15369	Elongin-C OS=Homo sapiens OX=9606 GN=ELOC PE=1 SV=1	18	3	3	12.5	41	112	4.78	2	2	1	1	2	1	2	2	1	1	2	1
High	P40199	Carcinoembryonic antigen-related cell adhesion molecule 6 OS=Homo sapiens OX=9606 GN=CEACAM6 PE=1 SV=4	18	5	4	37.2	24	344	5.82			2			1			2			1
High	P51571	Translocon-associated protein subunit delta OS=Homo sapiens OX=9606 GN=SSR4 PE=1 SV=1	18	4	4	19	31	173	6.15	1	3	4	1	1	1	1	3	3	1	1	1
High	Q9Y295	Developmentally-regulated GTP-binding protein 1 OS=Homo sapiens OX=9606 GN=DRG1 PE=1 SV=1	18	4	4	40.5	13	367	8.9	1		2	1	4	1	1		2	1	4	1
High	Q96SZ5	2-aminoethanethiol dioxygenase OS=Homo sapiens OX=9606 GN=ADO PE=1 SV=2	18	5	5	29.7	19	270	6.04	1	1	2	2	2	2	1	1	2	2	2	2
High	Q9Y570	Protein phosphatase methylesterase 1 OS=Homo sapiens OX=9606 GN=PPME1 PE=1 SV=3	18	7	7	42.3	21	386	5.97	1	2	2	1	1		1	2	2	1	1	
High	Q9NWB6	Arginine and glutamate-rich protein 1 OS=Homo sapiens OX=9606 GN=ARGLU1 PE=1 SV=1	18	3	3	33.2	8	273	10.35			3	2	3	2			3	2	3	2
High	Q7L2J0	7SK snRNA methylphosphate capping enzyme OS=Homo sapiens OX=9606 GN=MEPCE PE=1 SV=1	18	6	6	74.3	13	689	9.57	1	1	2	1	2		1	1	2	1	2	
High	P49748	Very long-chain specific acyl-CoA dehydrogenase, mitochondrial OS=Homo sapiens OX=9606 GN=ACADVL PE=1 SV=1	18	8	8	70.3	14	655	8.75	1	2	3			1	1	2	3			1
High	P06868	SWISS-PROT:P06868 (Bos taurus) Plasminogen precursor	18	8	8	91.2	18	812	7.5	1	3	1	1	3	3	1	3	1	1	3	3
High	P15559	NAD(P)H dehydrogenase [quinone] 1 OS=Homo sapiens OX=9606 GN=NQO1 PE=1 SV=1	18	5	5	30.8	25	274	8.88	5	4	2	1			4	3	2	1		
High	Q07954	Prolow-density lipoprotein receptor-related protein 1 OS=Homo sapiens OX=9606 GN=LRP1 PE=1 SV=2	18	7	7	504.3	2	4544	5.39	1	1	1	1	1		1	1	1	1	1	
High	Q92485	Acid sphingomyelinase-like phosphodiesterase 3b OS=Homo sapiens OX=9606 GN=SMPDL3B PE=1 SV=2	18	4	4	50.8	12	455	5.64	3	4	2	1	1	1	3	4	2	1	1	1
High	Q53EL6	Programmed cell death protein 4 OS=Homo sapiens OX=9606 GN=PDCD4 PE=1 SV=2	18	10	10	51.7	31	469	5.21	1	1	2	1		1	1	1	2	1		1
High	P51531	Probable global transcription activator SNF2L2 OS=Homo sapiens OX=9606 GN=SMARCA2 PE=1 SV=2	18	4	1	181.2	3	1590	7.2	2	1	2	2	1	2	2	1	2	2	1	2
High	Q969V3	Nicalin OS=Homo sapiens OX=9606 GN=NCLN PE=1 SV=2	18	3	3	62.9	6	563	6.89	2	2	3		2	3	2	2	3		2	2
High	Q6UB28	Methionine aminopeptidase 1D, mitochondrial OS=Homo sapiens OX=9606 GN=METAP1D PE=1 SV=1	18	2	2	37.1	9	335	6.84	2	2	2	1		2	2	2	2	1		2
High	Q9GZR2	RNA exonuclease 4 OS=Homo sapiens OX=9606 GN=REXO4 PE=1 SV=2	18	5	5	46.6	14	422	9.77				2	2	2				2	2	2
High	P81605	Dermcidin OS=Homo sapiens OX=9606 GN=DCD PE=1 SV=2	18	2	2	11.3	20	110	6.54	1	1	2	4	3	3	1	1	2	2	2	2
High	P23229	Integrin alpha-6 OS=Homo sapiens OX=9606 GN=ITGA6 PE=1 SV=5	18	10	10	126.5	9	1130	6.61	3	1	4	1	1	1	3	1	4	1	1	1
High	O75340	Programmed cell death protein 6 OS=Homo sapiens OX=9606 GN=PDCD6 PE=1 SV=1	18	6	6	21.9	44	191	5.4	6	3	3		1		6	3	3		1	
High	Q969Q0	60S ribosomal protein L36a-like OS=Homo sapiens OX=9606 GN=RPL36AL PE=1 SV=3	18	3	1	12.5	24	106	10.65	1	1	1	2	3	2	1	1	1	2	3	2
High	Q13418	Integrin-linked protein kinase OS=Homo sapiens OX=9606 GN=ILK PE=1 SV=2	18	4	4	51.4	8	452	8.07	3	2	3		1	1	3	2	3		1	1
High	Q9UGV2	Protein NDRG3 OS=Homo sapiens OX=9606 GN=NDRG3 PE=1 SV=2	18	4	4	41.4	14	375	5.31	3	2	1	1	2	1	3	2	1	1	2	1
High	Q6P1Q9	tRNA N(3)-methylcytidine methyltransferase METTL2B OS=Homo sapiens OX=9606 GN=METTL2B PE=1 SV=3	18	5	5	43.4	13	378	5.86	1	1	1	3	1	2	1	1	1	3	1	2
High	P61088	Ubiquitin-conjugating enzyme E2 N OS=Homo sapiens OX=9606 GN=UBE2N PE=1 SV=1	18	6	6	17.1	47	152	6.57	3	3	2	1	3	2	3	3	2	1	3	2
High	Q9Y676	28S ribosomal protein S18b, mitochondrial OS=Homo sapiens OX=9606 GN=MRPS18B PE=1 SV=1	18	3	3	29.4	15	258	9.38	1		2	2	2	3	1		2	2	2	3
High	Q92526	T-complex protein 1 subunit zeta-2 OS=Homo sapiens OX=9606 GN=CCT6B PE=1 SV=5	18	5	1	57.8	9	530	7.24	3	3	2	1	3	1	3	3	2	1	3	1
High	P82933	28S ribosomal protein S9, mitochondrial OS=Homo sapiens OX=9606 GN=MRPS9 PE=1 SV=2	18	6	6	45.8	19	396	9.51	2		2	1	1		2		2	1	1	
High	Q8WVY7	Ubiquitin-like domain-containing CTD phosphatase 1 OS=Homo sapiens OX=9606 GN=UBLCP1 PE=1 SV=2	18	6	6	36.8	17	318	6.46	1	1	2	2	6	2	1	1	1	2	6	2
High	Q14562	ATP-dependent RNA helicase DHX8 OS=Homo sapiens OX=9606 GN=DHX8 PE=1 SV=1	18	3	2	139.2	2	1220	8.32	1	1	1	3	3	3	1	1	1	2	2	2
High	Q16401	26S proteasome non-ATPase regulatory subunit 5 OS=Homo sapiens OX=9606 GN=PSMD5 PE=1 SV=3	18	6	6	56.2	15	504	5.48	5	3	5				5	3	5			
High	Q6P1M3	LLGL scribble cell polarity complex component 2 OS=Homo sapiens OX=9606 GN=LLGL2 PE=1 SV=2	18	5	5	113.4	6	1020	7.52	1	2	1	2	1	1	1	2	1	2	1	1
High	Q8NBF2	NHL repeat-containing protein 2 OS=Homo sapiens OX=9606 GN=NHLRC2 PE=1 SV=1	18	7	7	79.4	10	726	5.55		2	1	3	1	1		2	1	3	1	1
High	O75381	Peroxisomal membrane protein PEX14 OS=Homo sapiens OX=9606 GN=PEX14 PE=1 SV=1	17	4	4	41.2	16	377	4.94	2	3	4				2	3	4			
High	Q86YP4	Transcriptional repressor p66-alpha OS=Homo sapiens OX=9606 GN=GATAD2A PE=1 SV=1	17	4	3	68	9	633	9.94	1	1	2		4	1	1	1	2		4	1
High	Q9Y6N5	Sulfide:quinone oxidoreductase, mitochondrial OS=Homo sapiens OX=9606 GN=SQOR PE=1 SV=1	17	6	6	49.9	18	450	9.11	3	5	3				3	5	3			
High	Q8IYQ7	Threonine synthase-like 1 OS=Homo sapiens OX=9606 GN=THNSL1 PE=1 SV=2	17	6	6	83	11	743	7.12	1	1	1	2	2		1	1	1	2	2	
High	Q15262	Receptor-type tyrosine-protein phosphatase kappa OS=Homo sapiens OX=9606 GN=PTPRK PE=1 SV=2	17	6	6	162	6	1439	5.9	1	1	1		1	1	1	1	1		1	1
High	P27449	V-type proton ATPase 16 kDa proteolipid subunit c OS=Homo sapiens OX=9606 GN=ATP6V0C PE=1 SV=1	17	1	1	15.7	12	155	8.44	1	1	1		1		1	1	1		1	
High	Q9UJV9	Probable ATP-dependent RNA helicase DDX41 OS=Homo sapiens OX=9606 GN=DDX41 PE=1 SV=2	17	7	7	69.8	11	622	6.84	2	1		4	5	3	2	1		4	5	3
High	P62714	Serine/threonine-protein phosphatase 2A catalytic subunit beta isoform OS=Homo sapiens OX=9606 GN=PPP2CB PE=1 SV=1	17	5	5	35.6	22	309	5.43	1	1	1		2	2	1	1	1		2	2
High	P49720	Proteasome subunit beta type-3 OS=Homo sapiens OX=9606 GN=PSMB3 PE=1 SV=2	17	5	5	22.9	25	205	6.55	2	1	1	3	4	2	2	1	1	3	4	2
High	O75131	Copine-3 OS=Homo sapiens OX=9606 GN=CPNE3 PE=1 SV=1	17	4	3	60.1	9	537	5.85	1	1	2	1	1	3	1	1	2	1	1	3
High	P39656	Dolichyl-diphosphooligosaccharide--protein glycosyltransferase 48 kDa subunit OS=Homo sapiens OX=9606 GN=DDOST PE=1 SV=4	17	7	7	50.8	23	456	6.55	4	2	4	1	2	1	4	2	4	1	2	1
High	P82932	28S ribosomal protein S6, mitochondrial OS=Homo sapiens OX=9606 GN=MRPS6 PE=1 SV=3	17	4	4	14.2	30	125	9.26	1	1		2	2	2	1	1		2	2	2
High	Q9BSJ8	Extended synaptotagmin-1 OS=Homo sapiens OX=9606 GN=ESYT1 PE=1 SV=1	17	4	4	122.8	5	1104	5.83	1	2	3	1	1	2	1	2	3	1	1	2
High	P00403	Cytochrome c oxidase subunit 2 OS=Homo sapiens OX=9606 GN=MT-CO2 PE=1 SV=1	17	4	4	25.5	23	227	4.82	3	3	3	1	1		3	3	3	1	1	
High	Q9Y2F5	Little elongation complex subunit 1 OS=Homo sapiens OX=9606 GN=ICE1 PE=1 SV=5	17	8	8	247.7	5	2266	5.48				2	2	5				2	2	5
High	Q9NRF8	CTP synthase 2 OS=Homo sapiens OX=9606 GN=CTPS2 PE=1 SV=1	17	7	6	65.6	15	586	6.9	2	2	1	1		1	2	2	1	1		1
High	P53634	Dipeptidyl peptidase 1 OS=Homo sapiens OX=9606 GN=CTSC PE=1 SV=2	17	5	5	51.8	16	463	6.99	2	1	3	1	1	2	2	1	3	1	1	2
High	Q8NBQ5	Estradiol 17-beta-dehydrogenase 11 OS=Homo sapiens OX=9606 GN=HSD17B11 PE=1 SV=3	17	5	5	32.9	19	300	9.07	1		3		1		1		3		1	
High	Q9H2D6	TRIO and F-actin-binding protein OS=Homo sapiens OX=9606 GN=TRIOBP PE=1 SV=3	17	5	5	261.2	3	2365	8.48	3	3	4		1		3	3	4		1	
High	P84098	60S ribosomal protein L19 OS=Homo sapiens OX=9606 GN=RPL19 PE=1 SV=1	17	3	3	23.5	18	196	11.47	3	2	1	1	2	2	3	1	1	1	2	2
High	P49721	Proteasome subunit beta type-2 OS=Homo sapiens OX=9606 GN=PSMB2 PE=1 SV=1	17	3	3	22.8	12	201	7.02				3	3	3				3	3	3
High	Q9BY43	Charged multivesicular body protein 4a OS=Homo sapiens OX=9606 GN=CHMP4A PE=1 SV=3	17	4	4	25.1	22	222	4.7	1	1	1				1	1	1			
High	Q96PV6	Leukocyte receptor cluster member 8 OS=Homo sapiens OX=9606 GN=LENG8 PE=1 SV=3	17	7	7	88.1	15	800	9.11	3	2	2		1		3	2	2		1	
High	Q92572	AP-3 complex subunit sigma-1 OS=Homo sapiens OX=9606 GN=AP3S1 PE=1 SV=1	17	4	4	21.7	23	193	5.39	1	1	3	1	2	1	1	1	3	1	2	1
High	Q9Y2Q3	Glutathione S-transferase kappa 1 OS=Homo sapiens OX=9606 GN=GSTK1 PE=1 SV=3	17	3	3	25.5	18	226	8.41	2	2	3	1	1	1	2	2	3	1	1	1
High	Q92785	Zinc finger protein ubi-d4 OS=Homo sapiens OX=9606 GN=DPF2 PE=1 SV=2	17	5	5	44.1	17	391	6.33	3	1	1		1		3	1	1		1	
High	Q8NBS9	Thioredoxin domain-containing protein 5 OS=Homo sapiens OX=9606 GN=TXNDC5 PE=1 SV=2	17	5	5	47.6	15	432	5.97	1	1	2	2		1	1	1	2	2		1
High	P12036	Neurofilament heavy polypeptide OS=Homo sapiens OX=9606 GN=NEFH PE=1 SV=4	17	4	1	112.4	3	1026	6.18	1	1	2	3	4	3	1	1	2	3	3	3
High	P14678	Small nuclear ribonucleoprotein-associated proteins B and B' OS=Homo sapiens OX=9606 GN=SNRPB PE=1 SV=2	17	4	4	24.6	15	240	11.19	3	2	2		3	1	3	2	2		2	1
High	Q8WUJ3	Cell migration-inducing and hyaluronan-binding protein OS=Homo sapiens OX=9606 GN=CEMIP PE=1 SV=2	17	7	7	152.9	7	1361	7.85	4	4	7				4	4	6			
High	Q9Y4F1	FERM, ARHGEF and pleckstrin domain-containing protein 1 OS=Homo sapiens OX=9606 GN=FARP1 PE=1 SV=1	17	6	6	118.6	8	1045	8.15	3	3	3	2		1	3	3	3	2		1
High	Q7Z5K2	Wings apart-like protein homolog OS=Homo sapiens OX=9606 GN=WAPL PE=1 SV=1	17	7	7	132.9	7	1190	5.44	1	1		3	2	1	1	1		3	2	1
High	P82663	28S ribosomal protein S25, mitochondrial OS=Homo sapiens OX=9606 GN=MRPS25 PE=1 SV=1	17	4	4	20.1	29	173	8.82	1	1	1	1	2		1	1	1	1	2	
High	Q8IXT5	RNA-binding protein 12B OS=Homo sapiens OX=9606 GN=RBM12B PE=1 SV=2	17	6	6	118	6	1001	6.81	5	6	2				5	6	2			
High	Q5T1R4	Transcription factor HIVEP3 OS=Homo sapiens OX=9606 GN=HIVEP3 PE=2 SV=1	17	6	3	259.3	3	2406	7.81				4	4	5				4	4	5
High	O95671	Probable bifunctional dTTP/UTP pyrophosphatase/methyltransferase protein OS=Homo sapiens OX=9606 GN=ASMTL PE=1 SV=3	17	5	5	68.8	12	621	6.07	1	1	2	1	1	1	1	1	2	1	1	1
High	P61011	Signal recognition particle 54 kDa protein OS=Homo sapiens OX=9606 GN=SRP54 PE=1 SV=1	17	5	5	55.7	10	504	8.75	1		1	2	2	3	1		1	2	2	3
High	Q96FZ2	Abasic site processing protein HMCES OS=Homo sapiens OX=9606 GN=HMCES PE=1 SV=1	17	5	5	40.5	15	354	8.15	3	2	1	2	1	2	3	2	1	2	1	2
High	Q9P2N5	RNA-binding protein 27 OS=Homo sapiens OX=9606 GN=RBM27 PE=1 SV=2	17	6	5	118.6	6	1060	9.19				1	2	1				1	2	
High	Q13464	Rho-associated protein kinase 1 OS=Homo sapiens OX=9606 GN=ROCK1 PE=1 SV=1	17	4	1	158.1	3	1354	5.9	1		2	1	1	2	1		1	1	2	1
High	O95571	Persulfide dioxygenase ETHE1, mitochondrial OS=Homo sapiens OX=9606 GN=ETHE1 PE=1 SV=2	17	6	6	27.9	36	254	6.83	2	2	1	1			2		1			
High	Q8IYB5	Stromal membrane-associated protein 1 OS=Homo sapiens OX=9606 GN=SMAP1 PE=1 SV=2	17	5	5	50.4	15	467	8.75	1	1	2	1	2	2	1	1	2	1	2	2
High	P20839	Inosine-5'-monophosphate dehydrogenase 1 OS=Homo sapiens OX=9606 GN=IMPDH1 PE=1 SV=2	17	4	3	55.4	11	514	6.9		1		2	3	3				2	3	3
High	P02786	Transferrin receptor protein 1 OS=Homo sapiens OX=9606 GN=TFRC PE=1 SV=2	17	8	8	84.8	13	760	6.61		2	2	1		1		2	2	1		1

High	P08579	U2 small nuclear ribonucleoprotein B'' OS=Homo sapiens OX=9606 GN=SNRPB2 PE=1 SV=1	17	3	1	25.5	12	225	9.72	2	1	1	1	3	2	2	1	1	1	2	2
High	P46379	Large proline-rich protein BAG6 OS=Homo sapiens OX=9606 GN=BAG6 PE=1 SV=2	17	5	5	119.3	7	1132	5.6				3	2	4				3	2	4
High	Q13243	Serine/arginine-rich splicing factor 5 OS=Homo sapiens OX=9606 GN=SRSF5 PE=1 SV=1	17	5	4	31.2	25	272	11.59	2	2	1	2		2	2	2	1	2		2
High	O00330	Pyruvate dehydrogenase protein X component, mitochondrial OS=Homo sapiens OX=9606 GN=PDHX PE=1 SV=3	16	5	5	54.1	12	501	8.66		1		1	1			1		1	1	
High	Q14651	Plastin-1 OS=Homo sapiens OX=9606 GN=PLS1 PE=1 SV=2	16	5	1	70.2	11	629	5.41	2	2	2	1	2	2	2	2	2	1	2	2
High	Q96ST3	Paired amphipathic helix protein Sin3a OS=Homo sapiens OX=9606 GN=SIN3A PE=1 SV=2	16	8	8	145.1	6	1273	7.25				1	3	1				1	3	1
High	Q9NXH8	Torsin-4A OS=Homo sapiens OX=9606 GN=TOR4A PE=1 SV=2	16	6	6	46.9	15	423	9.94	1		4		2	1	1		4		2	1
High	Q9BPX5	Actin-related protein 2/3 complex subunit 5-like protein OS=Homo sapiens OX=9606 GN=ARPC5L PE=1 SV=1	16	4	3	16.9	34	153	6.6	2	2	1	1	2	1	2	2	1	1	1	1
High	Q5JPE7	Nodal modulator 2 OS=Homo sapiens OX=9606 GN=NOMO2 PE=1 SV=1	16	4	4	139.4	3	1267	5.76	3	3	2				3	3	2			
High	Q06330	Recombining binding protein suppressor of hairless OS=Homo sapiens OX=9606 GN=RBPJ PE=1 SV=3	16	5	5	55.6	12	500	7.18				2	3	3				2	3	3
High	Q96GM8	Target of EGR1 protein 1 OS=Homo sapiens OX=9606 GN=TOE1 PE=1 SV=1	16	4	4	56.5	13	510	7.18	2	1	2	1	1	1	2	1	2	1	1	1
High	Q92922	SWI/SNF complex subunit SMARCC1 OS=Homo sapiens OX=9606 GN=SMARCC1 PE=1 SV=3	16	5	3	122.8	7	1105	5.76			1		1	1			1		1	1
High	P09525	Annexin A4 OS=Homo sapiens OX=9606 GN=ANXA4 PE=1 SV=4	16	5	5	35.9	16	319	6.13	2	2	1	1	1	2	2	2	1	1	1	2
High	P10620	Microsomal glutathione S-transferase 1 OS=Homo sapiens OX=9606 GN=MGST1 PE=1 SV=1	16	3	3	17.6	26	155	9.39	3	3	3		1		3	3	3		1	
High	Q96EP5	DAZ-associated protein 1 OS=Homo sapiens OX=9606 GN=DAZAP1 PE=1 SV=1	16	3	3	43.4	10	407	8.56	2	2	2	1	2	1	2	2	2	1	2	1
High	P09234	U1 small nuclear ribonucleoprotein C OS=Homo sapiens OX=9606 GN=SNRPC PE=1 SV=1	16	2	2	17.4	19	159	9.67	1	2	2	2	2	2	1	2	2	2	2	2
High	A8CG34	Nuclear envelope pore membrane protein POM 121C OS=Homo sapiens OX=9606 GN=POM121C PE=1 SV=3	16	5	5	125	6	1229	10.37			1		1	1			1		1	1
High	Q96P47	Arf-GAP with GTPase, ANK repeat and PH domain-containing protein 3 OS=Homo sapiens OX=9606 GN=AGAP3 PE=1 SV=2	16	3	3	95	5	875	7.97	2	3	3	1	1	1	2	3	2	1	1	1
High	O00182	Galectin-9 OS=Homo sapiens OX=9606 GN=LGALS9 PE=1 SV=2	16	5	2	39.5	15	355	9.17	1		1	2	3	2	1		1	2	3	2
High	Q9GZS3	WD repeat-containing protein 61 OS=Homo sapiens OX=9606 GN=WDR61 PE=1 SV=1	16	4	4	33.6	14	305	5.47	1	2	3		2	1	1	2	3		2	1
High	Q96A65	Exocyst complex component 4 OS=Homo sapiens OX=9606 GN=EXOC4 PE=1 SV=1	16	8	8	110.4	11	974	6.49	5	4	5				5	4	5			
High	O60925	Prefoldin subunit 1 OS=Homo sapiens OX=9606 GN=PFDN1 PE=1 SV=2	16	3	3	14.2	21	122	6.81		1	1	3	1	3		1	1	3	1	3
High	Q6P996	Pyridoxal-dependent decarboxylase domain-containing protein 1 OS=Homo sapiens OX=9606 GN=PDXDC1 PE=1 SV=2	16	7	7	86.7	12	788	5.38	2	1	3		1		2	1	3		1	
High	Q96DN6	Methyl-CpG-binding domain protein 6 OS=Homo sapiens OX=9606 GN=MBD6 PE=1 SV=2	16	6	6	101.1	10	1003	9.63				3	2	2				3	2	2
High	Q13404	Ubiquitin-conjugating enzyme E2 variant 1 OS=Homo sapiens OX=9606 GN=UBE2V1 PE=1 SV=2	16	3	3	16.5	18	147	7.93	1	1		2	3	2	1	1		2	3	2
High	P12277	Creatine kinase B-type OS=Homo sapiens OX=9606 GN=CKB PE=1 SV=1	16	4	4	42.6	20	381	5.59	2	3	1				2	3	1			
High	P27361	Mitogen-activated protein kinase 3 OS=Homo sapiens OX=9606 GN=MAPK3 PE=1 SV=4	16	4	1	43.1	15	379	6.74		1	2	1	1	2		1	2	1	1	2
High	O60762	Dolichol-phosphate mannosyltransferase subunit 1 OS=Homo sapiens OX=9606 GN=DPM1 PE=1 SV=1	16	6	6	29.6	28	260	9.57	3	2	5				3	2	5			
High	Q96SQ9	Cytochrome P450 2S1 OS=Homo sapiens OX=9606 GN=CYP2S1 PE=1 SV=2	16	4	4	55.8	8	504	8.62	4	4	3				4	4	3			
High	Q99828	Calcium and integrin-binding protein 1 OS=Homo sapiens OX=9606 GN=CIB1 PE=1 SV=4	16	3	3	21.7	20	191	4.78	2	2	2		1		2	2	2		1	
High	Q9NZJ9	Diphosphoinositol polyphosphate phosphohydrolase 2 OS=Homo sapiens OX=9606 GN=NUDT4 PE=1 SV=2	16	4	4	20.3	31	180	6.35	2	5	2		1		2	4	2		1	
High	Q9UN37	Vacuolar protein sorting-associated protein 4A OS=Homo sapiens OX=9606 GN=VPS4A PE=1 SV=1	16	4	2	48.9	11	437	7.8		1		1	2	2		1		1	2	2
High	P42338	Phosphatidylinositol 4,5-bisphosphate 3-kinase catalytic subunit beta isoform OS=Homo sapiens OX=9606 GN=PIK3CB PE=1 SV=1	16	5	5	122.7	5	1070	7.09	4	3	3		1	1	4	3	3		1	1
High	P52701	DNA mismatch repair protein Msh6 OS=Homo sapiens OX=9606 GN=MSH6 PE=1 SV=2	16	8	8	152.7	7	1360	6.9		1		1		1		1		1		1
High	P31939	Bifunctional purine biosynthesis protein ATIC OS=Homo sapiens OX=9606 GN=ATIC PE=1 SV=3	16	8	8	64.6	18	592	6.71	2	2	4				2	2	4			
High	O95373	Importin-7 OS=Homo sapiens OX=9606 GN=IPO7 PE=1 SV=1	16	10	10	119.4	13	1038	4.82	5	2	3		1		5	2	3		1	
High	P35250	Replication factor C subunit 2 OS=Homo sapiens OX=9606 GN=RFC2 PE=1 SV=3	16	7	7	39.1	23	354	6.44	1	1		1	3	1	1	1		1	3	1
High	Q9UI30	Multifunctional methyltransferase subunit TRM112-like protein OS=Homo sapiens OX=9606 GN=TRMT112 PE=1 SV=1	16	2	2	14.2	22	125	5.26	2	1	1	2	2	2	2	1	1	2	2	2
High	O43148	mRNA cap guanine-N7 methyltransferase OS=Homo sapiens OX=9606 GN=RNMT PE=1 SV=1	16	4	4	54.8	10	476	6.61		1	3	2	1	1		1	3	2	1	1
High	Q14919	Dr1-associated corepressor OS=Homo sapiens OX=9606 GN=DRAP1 PE=1 SV=3	16	3	3	22.3	21	205	5.17	1	2	2	1	1	2	1	2	2	1	1	2
High	P83881	60S ribosomal protein L36a OS=Homo sapiens OX=9606 GN=RPL36A PE=1 SV=2	16	3	1	12.4	24	106	10.58	1	1	1	2	3	2	1	1	1	2	3	2
High	O15446	DNA-directed RNA polymerase I subunit RPA34 OS=Homo sapiens OX=9606 GN=POLR1G PE=1 SV=1	16	5	5	55	15	510	8.51			1	1	2	1			1	1	2	1
High	Q14451	Growth factor receptor-bound protein 7 OS=Homo sapiens OX=9606 GN=GRB7 PE=1 SV=2	16	7	7	59.6	18	532	8.5	4	2	1	1			4	2	1	1		
High	P08174	Complement decay-accelerating factor OS=Homo sapiens OX=9606 GN=CD55 PE=1 SV=4	16	4	4	41.4	9	381	7.59	2	2	1				2	2	1			
High	Q8TBC3	SH3KBP1-binding protein 1 OS=Homo sapiens OX=9606 GN=SHKBP1 PE=1 SV=2	16	4	3	76.3	8	707	8.28				3	2	3				3	2	3
High	Q9NQC3	Reticulon-4 OS=Homo sapiens OX=9606 GN=RTN4 PE=1 SV=2	16	5	5	129.9	8	1192	4.5	1	2	2		1		1	2	1		1	
High	P50238	Cysteine-rich protein 1 OS=Homo sapiens OX=9606 GN=CRIP1 PE=1 SV=3	16	3	3	8.5	56	77	8.75	3	2	2		2	1	1	2	2		2	1
High	O43447	Peptidyl-prolyl cis-trans isomerase H OS=Homo sapiens OX=9606 GN=PPIH PE=1 SV=1	16	4	4	19.2	23	177	8.07	2	1	2	1	1	1	2	1	2	1	1	1
High	Q9NRX3	NADH dehydrogenase [ubiquinone] 1 alpha subcomplex subunit 4-like 2 OS=Homo sapiens OX=9606 GN=NDUFA4L2 PE=3 SV=1	16	3	3	10	29	87	9.92	1	3	3				1	3	3			
High	Q9P0J7	E3 ubiquitin-protein ligase KCMF1 OS=Homo sapiens OX=9606 GN=KCMF1 PE=1 SV=2	16	4	4	41.9	19	381	5.66	1	1	1	1	1	1	1	1	1	1	1	1
High	Q9C0J8	pre-mRNA 3' end processing protein WDR33 OS=Homo sapiens OX=9606 GN=WDR33 PE=1 SV=2	15	5	5	145.8	6	1336	9.17				2	3	2				2	3	2
High	Q86SX6	Glutaredoxin-related protein 5, mitochondrial OS=Homo sapiens OX=9606 GN=GLRX5 PE=1 SV=2	15	3	3	16.6	28	157	6.79	2	1	1	1	2	1	2	1	1	1	2	1
High	P08621	U1 small nuclear ribonucleoprotein 70 kDa OS=Homo sapiens OX=9606 GN=SNRNP70 PE=1 SV=2	15	4	4	51.5	12	437	9.94	1	1	2	1	1	1	1	1	2	1	1	1
High	O43760	Synaptogyrin-2 OS=Homo sapiens OX=9606 GN=SYNGR2 PE=1 SV=1	15	4	4	24.8	17	224	4.94	2	3	1		1		2	3	1		1	
High	Q9NTK5	Obg-like ATPase 1 OS=Homo sapiens OX=9606 GN=OLA1 PE=1 SV=2	15	5	5	44.7	11	396	7.81			1	2	1	1			1	2	1	1
High	Q9UBC2	Epidermal growth factor receptor substrate 15-like 1 OS=Homo sapiens OX=9606 GN=EPS15L1 PE=1 SV=1	15	5	5	94.2	5	864	5.11			3		1	1			3		1	1
High	Q99615	DnaJ homolog subfamily C member 7 OS=Homo sapiens OX=9606 GN=DNAJC7 PE=1 SV=2	15	6	6	56.4	15	494	6.96	1	1	1	2	1	3	1	1	1	2	1	3
High	P25787	Proteasome subunit alpha type-2 OS=Homo sapiens OX=9606 GN=PSMA2 PE=1 SV=2	15	4	4	25.9	23	234	7.43	1	1	1	2	3	2	1	1	1	2	2	2
High	Q13769	THO complex subunit 5 homolog OS=Homo sapiens OX=9606 GN=THOC5 PE=1 SV=2	15	5	5	78.5	9	683	6.87	1	1		1		1	1	1		1		1
High	Q7Z434	Mitochondrial antiviral-signaling protein OS=Homo sapiens OX=9606 GN=MAVS PE=1 SV=2	15	4	4	56.5	12	540	5.52	1	2		1	2	2	1	2		1	2	2
High	Q13098	COP9 signalosome complex subunit 1 OS=Homo sapiens OX=9606 GN=GPS1 PE=1 SV=4	15	6	6	55.5	16	491	6.74	1	1		1	1	1	1	1		1	1	1
High	O94916	Nuclear factor of activated T-cells 5 OS=Homo sapiens OX=9606 GN=NFAT5 PE=1 SV=1	15	7	7	165.7	7	1531	5.24	2	2	8				2	2	7			
High	Q8TF72	Protein Shroom3 OS=Homo sapiens OX=9606 GN=SHROOM3 PE=1 SV=2	15	8	8	216.7	7	1996	7.8			1		1				1		1	
High	O15551	Claudin-3 OS=Homo sapiens OX=9606 GN=CLDN3 PE=1 SV=1	15	3	3	23.3	15	220	8.05		1	2	2	2	2		1	2	2	2	2
High	Q9UKY1	Zinc fingers and homeoboxes protein 1 OS=Homo sapiens OX=9606 GN=ZHX1 PE=1 SV=1	15	6	6	98	15	873	6.05	1		1				1		1			
High	Q8IY37	Probable ATP-dependent RNA helicase DHX37 OS=Homo sapiens OX=9606 GN=DHX37 PE=1 SV=1	15	8	8	129.5	8	1157	8.1	2	1	1	1	2	2	2	1	1	1	2	2
High	Q96FQ6	Protein S100-A16 OS=Homo sapiens OX=9606 GN=S100A16 PE=1 SV=1	15	3	3	11.8	34	103	6.79	2	2	3	1	1	1	2	2	1	1	1	1
High	P47985	Cytochrome b-c1 complex subunit Rieske, mitochondrial OS=Homo sapiens OX=9606 GN=UQCRFS1 PE=1 SV=2	15	5	1	29.6	20	274	8.32	3	1	4	2	1	1	3	1	4	2	1	1
High	Q9ULW0	Targeting protein for Xklp2 OS=Homo sapiens OX=9606 GN=TPX2 PE=1 SV=2	15	7	7	85.6	15	747	9.23	1	1	1				1	1	1			
High	Q9UKB1	F-box/WD repeat-containing protein 11 OS=Homo sapiens OX=9606 GN=FBXW11 PE=1 SV=1	15	6	6	62.1	11	542	7.12				3	5	3				3	5	3
High	P53677	AP-3 complex subunit mu-2 OS=Homo sapiens OX=9606 GN=AP3M2 PE=1 SV=1	15	3	1	46.9	9	418	7.56	2	2	2	1	2	1	2	2	2	1	2	1
High	P83916	Chromobox protein homolog 1 OS=Homo sapiens OX=9606 GN=CBX1 PE=1 SV=1	15	2	1	21.4	15	185	4.93	2	2	1		1		1	1	1		1	
High	P48634	Protein PRRC2A OS=Homo sapiens OX=9606 GN=PRRC2A PE=1 SV=3	15	6	6	228.7	6	2157	9.45			1	1	1	2			1	1	1	2
High	Q9UNS2	COP9 signalosome complex subunit 3 OS=Homo sapiens OX=9606 GN=COPS3 PE=1 SV=3	15	5	5	47.8	17	423	6.65	1	1	2	1	2	1	1	1	2	1	2	1
High	Q6UN15	Pre-mRNA 3'-end-processing factor FIP1 OS=Homo sapiens OX=9606 GN=FIP1L1 PE=1 SV=1	15	5	5	66.5	13	594	5.59	1	2		1	2	3	1	2		1	2	3
High	O60216	Double-strand-break repair protein rad21 homolog OS=Homo sapiens OX=9606 GN=RAD21 PE=1 SV=2	15	5	5	71.6	9	631	4.65	2	2	1	2	1		2	2	1	2	1	
High	Q8N0W3	L-fucose kinase OS=Homo sapiens OX=9606 GN=FCSK PE=1 SV=2	15	6	6	117.5	8	1084	6.25	5	6	3				5	6	3			
High	P28070	Proteasome subunit beta type-4 OS=Homo sapiens OX=9606 GN=PSMB4 PE=1 SV=4	15	3	3	29.2	13	264	5.97	1	1	1	3	3	3	1	1	1	3	3	3
High	Q07021	Complement component 1 Q subcomponent-binding protein, mitochondrial OS=Homo sapiens OX=9606 GN=C1QBP PE=1 SV=1	15	4	4	31.3	26	282	4.84	1	2	3		2		1	2	3		2	
High	Q9NZZ3	Charged multivesicular body protein 5 OS=Homo sapiens OX=9606 GN=CHMP5 PE=1 SV=1	15	3	3	24.6	20	219	4.83	2	2	1		1	1	2	2	1		1	1
High	Q9NZN5	Rho guanine nucleotide exchange factor 12 OS=Homo sapiens OX=9606 GN=ARHGEF12 PE=1 SV=1	15	8	8	173.1	7	1544	5.74												
High	P37198	Nuclear pore glycoprotein p62 OS=Homo sapiens OX=9606 GN=NUP62 PE=1 SV=3	15	6	6	53.2	19	522	5.31	1	1		1	1	1	1	1		1	1	1
High	Q15942	Zyxin OS=Homo sapiens OX=9606 GN=ZYX PE=1 SV=1	15	6	6	61.2	16	572	6.67	3	3	2	3			3	3	2	3		
High	Q96PU5	E3 ubiquitin-protein ligase NEDD4-like OS=Homo sapiens OX=9606 GN=NEDD4L PE=1 SV=2	15	4	3	111.9	4	975	5.82	1	1	1	3	1	2	1	1	1	3	1	2
High	P23458	Tyrosine-protein kinase JAK1 OS=Homo sapiens OX=9606 GN=JAK1 PE=1 SV=2	15	5	5	133.2	6	1154	7.55	3	3	2				3	3	2			
High	P68400	Casein kinase II subunit alpha OS=Homo sapiens OX=9606 GN=CSNK2A1 PE=1 SV=1	15	5	5	45.1	21	391	7.74	3	3	3	1		1	3	3	3	1		1
High	O95239	Chromosome-associated kinesin KIF4A OS=Homo sapiens OX=9606 GN=KIF4A PE=1 SV=3	15	7	7	139.8	8	1232	6.27	1		1	1	2	1	1		1	1	2	1
High	P25490	Transcriptional repressor protein YY1 OS=Homo sapiens OX=9606 GN=YY1 PE=1 SV=2	15	3	3	44.7	7	414	6.25		1	2	2	1	2		1	2	2	1	2
High	Q9Y399	28S ribosomal protein S2, mitochondrial OS=Homo sapiens OX=9606 GN=MRPS2 PE=1 SV=1	15	5	5	33.2	14	296	9.26			1	1	3	1			1	1	3	1
High	Q8WVB6	Chromosome transmission fidelity protein 18 homolog OS=Homo sapiens OX=9606 GN=CHTF18 PE=1 SV=1	15	3	3	107.3	4	975	7.21	1	2	1	1	1	1	1	2	1	1	1	1
High	Q9Y618	Nuclear receptor corepressor 2 OS=Homo sapiens OX=9606 GN=NCOR2 PE=1 SV=3	15	8	8	273.5	4	2514	7.59	1		1	1	2	1	1		1	1	2	1
High	Q14558	Phosphoribosyl pyrophosphate synthase-associated protein 1 OS=Homo sapiens OX=9606 GN=PRPSAP1 PE=1 SV=2	15	4	3	39.4	15	356	7.2	3	3	2		1	1	3	3	2		1	1
High	P40222	Alpha-taxilin OS=Homo sapiens OX=9606 GN=TXLNA PE=1 SV=3	15	7	7	61.9	19	546	6.52		2	2	1	3	2		2	2	1	3	2
High	Q9BRU9	rRNA-processing protein UTP23 homolog OS=Homo sapiens OX=9606 GN=UTP23 PE=1 SV=2	15	3	3	28.4	14	249	10.07			1	1	2	2			1	1	2	2
High	P29034	Protein S100-A2 OS=Homo sapiens OX=9606 GN=S100A2 PE=1 SV=3	15	2	2	11.1	19	98	4.78	2	2	2	1	1		2	2	2	1	1	
High	P50552	Vasodilator-stimulated phosphoprotein OS=Homo sapiens OX=9606 GN=VASP PE=1 SV=3	15	5	5	39.8	16	380	8.94	3	2	2				3	2	2			
High	O00154	Cytosolic acyl coenzyme A thioester hydrolase OS=Homo sapiens OX=9606 GN=ACOT7 PE=1 SV=3	15	4	4	41.8	11	380	8.54	2	3	2		2		2	3	2		2	
High	Q9P1Y5	Calmodulin-regulated spectrin-associated protein 3 OS=Homo sapiens OX=9606 GN=CAMSAP3 PE=1 SV=2	15	5	5	134.7	5	1249	8.35	1	1	2	1	1	1	1	1	2	1	1	1
High	Q9H4M9	EH domain-containing protein 1 OS=Homo sapiens OX=9606 GN=EHD1 PE=1 SV=2	15	5	4	60.6	13	534	6.83	2	3	3	1	1	1	2	3	2	1	1	1
High	Q9NVS9	Pyridoxine-5'-phosphate oxidase OS=Homo sapiens OX=9606 GN=PNPO PE=1 SV=1	15	6	6	30	26	261	7.06			1	1	2	2			1	1	2	2
High	Q9Y6G9	Cytoplasmic dynein 1 light intermediate chain 1 OS=Homo sapiens OX=9606 GN=DYNC1LI1 PE=1 SV=3	15	4	4	56.5	8	523	6.42	2	2	2		1		2	2	2		1	
High	P53985	Monocarboxylate transporter 1 OS=Homo sapiens OX=9606 GN=SLC16A1 PE=1 SV=3	15	2	2	53.9	6	500	8.66	2	2	2	1	1	1	2	2	1	1	1	1
High	Q9H3P7	Golgi resident protein GCP60 OS=Homo sapiens OX=9606 GN=ACBD3 PE=1 SV=4	15	6	6	60.6	14	528	5.06		1	1		2			1	1		2	
High	P78385	SWISS-PROT:P78385 Keratin, type II cuticular Hb3 (Hair keratin, type II Hb3) - Homo sapiens (Human).	14	2	1	54.2	4	493	5.4	2	2	2	1	1	1	1	1	1	1	1	1
High	Q12815	Tastin OS=Homo sapiens OX=9606 GN=TROAP PE=1 SV=3	14	8	8	83.8	13	778	7.36					1						1	
High	Q9HAU0	Pleckstrin homology domain-containing family A member 5 OS=Homo sapiens OX=9606 GN=PLEKHA5 PE=1 SV=1	14	5	5	127.4	5	1116	7.53	1	1	2		2		1	1	2		2	
High	Q96AE4	Far upstream element-binding protein 1 OS=Homo sapiens OX=9606 GN=FUBP1 PE=1 SV=3	14	4	2	67.5	6	644	7.61	1	2	1		1	2	1	2	1		1	2
High	Q13131	5'-AMP-activated protein kinase catalytic subunit alpha-1 OS=Homo sapiens OX=9606 GN=PRKAA1 PE=1 SV=4	14	5	5	64	11	559	8.12	1	1	1		3	1	1	1	1		3	1
High	Q14517	Protocadherin Fat 1 OS=Homo sapiens OX=9606 GN=FAT1 PE=1 SV=2	14	6	6	506	2	4588	5	2	4	4		2		2	4	4		2	
High	P55199	RNA polymerase II elongation factor ELL OS=Homo sapiens OX=9606 GN=ELL PE=1 SV=1	14	5	5	68.2	10	621	9.33				2	3	3				2	3	3
High	Q8N6M0	Deubiquitinase OTUD6B OS=Homo sapiens OX=9606 GN=OTUD6B PE=1 SV=1	14	3	3	33.8	11	293	6.05	1	2	2	2	2	1	1	2	2	2	2	1
High	Q05086	Ubiquitin-protein ligase E3A OS=Homo sapiens OX=9606 GN=UBE3A PE=1 SV=4	14	5	5	100.6	7	875	5.22			3	2	1	2			3	2	1	2
High	Q9NUI1	Peroxisomal 2,4-dienoyl-CoA reductase [(3E)-enoyl-CoA-producing] OS=Homo sapiens OX=9606 GN=DECR2 PE=1 SV=1	14	6	6	30.8	21	292	9.22	1			1	2		1			1	2	
High	Q01105	Protein SET OS=Homo sapiens OX=9606 GN=SET PE=1 SV=3	14	3	3	33.5	12	290	4.32			2	3	1	2			2	3	1	2
High	Q4G0N4	NAD kinase 2, mitochondrial OS=Homo sapiens OX=9606 GN=NADK2 PE=1 SV=2	14	6	6	49.4	13	442	8.18				2	1	2				2	1	2
High	Q8NFF5	FAD synthase OS=Homo sapiens OX=9606 GN=FLAD1 PE=1 SV=1	14	8	8	65.2	19	587	6.93	1			2	3	2	1			2	3	2
High	Q8IUC4	Rhophilin-2 OS=Homo sapiens OX=9606 GN=RHPN2 PE=1 SV=1	14	5	5	76.9	11	686	6.8	2	1	2				2	1	2			
High	Q86UE8	Serine/threonine-protein kinase tousled-like 2 OS=Homo sapiens OX=9606 GN=TLK2 PE=1 SV=2	14	3	2	87.6	3	772	8.41			1	2	2	1			1	1	1	1
High	P49184	Deoxyribonuclease-1-like 1 OS=Homo sapiens OX=9606 GN=DNASE1L1 PE=1 SV=1	14	3	3	33.9	15	302	5.74	2	2	3				2	2	3			
High	P13688	Carcinoembryonic antigen-related cell adhesion molecule 1 OS=Homo sapiens OX=9606 GN=CEACAM1 PE=1 SV=2	14	3	2	57.5	8	526	5.97	2	1	1	1	1	1	2	1	1	1	1	1
High	Q02338	D-beta-hydroxybutyrate dehydrogenase, mitochondrial OS=Homo sapiens OX=9606 GN=BDH1 PE=1 SV=3	14	6	6	38.1	22	343	8.95	1	1					1	1				
High	Q6P3X3	Tetratricopeptide repeat protein 27 OS=Homo sapiens OX=9606 GN=TTC27 PE=1 SV=1	14	6	6	96.6	9	843	5.59	4	4	5				4	4	5			
High	Q9NS69	Mitochondrial import receptor subunit TOM22 homolog OS=Homo sapiens OX=9606 GN=TOMM22 PE=1 SV=3	14	4	4	15.5	51	142	4.34	3	3	4	1		1	2	2	3	1		1
High	P62312	U6 snRNA-associated Sm-like protein LSm6 OS=Homo sapiens OX=9606 GN=LSM6 PE=1 SV=1	14	2	2	9.1	19	80	9.58	1		1	2	2	2	1		1	2	2	2
High	P35249	Replication factor C subunit 4 OS=Homo sapiens OX=9606 GN=RFC4 PE=1 SV=2	14	7	7	39.7	19	363	8.02			1		2				1		1	
High	P49757	Protein numb homolog OS=Homo sapiens OX=9606 GN=NUMB PE=1 SV=2	14	5	5	70.8	9	651	8.51	2	2		1	2	1	2	2		1	2	1
High	Q9BUA3	Spindlin interactor and repressor of chromatin-binding protein OS=Homo sapiens OX=9606 GN=SPINDOC PE=1 SV=3	14	6	6	41	20	381	5.01	4	4	5		1		4	4	5		1	
High	P0C7P4	Putative cytochrome b-c1 complex subunit Rieske-like protein 1 OS=Homo sapiens OX=9606 GN=UQCRFS1P1 PE=5 SV=1	14	5	1	30.8	17	283	8.87	2	1	4	2	1	1	2	1	4	2	1	1
High	P23919	Thymidylate kinase OS=Homo sapiens OX=9606 GN=DTYMK PE=1 SV=4	14	4	4	23.8	17	212	8.27		1	2	1		1		1	2	1		1
High	Q8IXI1	Mitochondrial Rho GTPase 2 OS=Homo sapiens OX=9606 GN=RHOT2 PE=1 SV=2	14	6	5	68.1	17	618	5.86	1	1	1	2	3	2	1	1	1	2	3	2
High	Q8IWU2	Serine/threonine-protein kinase LMTK2 OS=Homo sapiens OX=9606 GN=LMTK2 PE=1 SV=2	14	5	5	164.8	5	1503	4.48	1	1	4				1	1	3			
High	Q8TDD1	ATP-dependent RNA helicase DDX54 OS=Homo sapiens OX=9606 GN=DDX54 PE=1 SV=2	14	7	6	98.5	10	881	10.02												
High	P22059	Oxysterol-binding protein 1 OS=Homo sapiens OX=9606 GN=OSBP PE=1 SV=1	14	4	4	89.4	6	807	7.3	1	1	2		1		1	1	2		1	
High	Q92643	GPI-anchor transamidase OS=Homo sapiens OX=9606 GN=PIGK PE=1 SV=2	14	5	5	45.2	16	395	6.16	2	3	3	1	1	1	2	3	3	1	1	1
High	Q8TEQ6	Gem-associated protein 5 OS=Homo sapiens OX=9606 GN=GEMIN5 PE=1 SV=3	14	5	5	168.5	4	1508	6.62		1	1		2			1	1		2	
High	Q9NX20	39S ribosomal protein L16, mitochondrial OS=Homo sapiens OX=9606 GN=MRPL16 PE=1 SV=1	14	5	5	28.4	20	251	10.13	3	1	2		1		3	1	2		1	
High	Q9BXS6	Nucleolar and spindle-associated protein 1 OS=Homo sapiens OX=9606 GN=NUSAP1 PE=1 SV=1	14	4	4	49.4	13	441	9.91	1	1		1	2	1	1	1		1	2	1
High	P60468	Protein transport protein Sec61 subunit beta OS=Homo sapiens OX=9606 GN=SEC61B PE=1 SV=2	14	2	2	10	22	96	11.56	2	2	1	1	1	1	2	2	1	1	1	1
High	Q8WVJ2	NudC domain-containing protein 2 OS=Homo sapiens OX=9606 GN=NUDCD2 PE=1 SV=1	14	2	2	17.7	13	157	5.07	3	2	2		1	1	2	1	2		1	1
High	Q8NB16	Mixed lineage kinase domain-like protein OS=Homo sapiens OX=9606 GN=MLKL PE=1 SV=1	14	5	5	54.4	14	471	8.82	1	2	1		1		1	2	1		1	
High	Q9Y316	Protein MEMO1 OS=Homo sapiens OX=9606 GN=MEMO1 PE=1 SV=1	14	5	5	33.7	18	297	7.14	2	1	2	1		1	2	1	2	1		1
High	Q92530	Proteasome inhibitor PI31 subunit OS=Homo sapiens OX=9606 GN=PSMF1 PE=1 SV=2	14	3	3	29.8	14	271	5.74	1	2	1	1	2	1	1	2	1	1	2	1
High	Q13155	Aminoacyl tRNA synthase complex-interacting multifunctional protein 2 OS=Homo sapiens OX=9606 GN=AIMP2 PE=1 SV=2	14	5	5	35.3	23	320	8.22	1		2	1	1		1		2	1	1	
High	Q9BT78	COP9 signalosome complex subunit 4 OS=Homo sapiens OX=9606 GN=COPS4 PE=1 SV=1	14	6	6	46.2	18	406	5.83	2		2	1		2	2		2	1		2
High	P62861	FAU ubiquitin-like and ribosomal protein S30 OS=Homo sapiens OX=9606 GN=FAU PE=1 SV=2	14	2	2	14.4	8	133	10.17	1	1	2	1	1	1	1	1	1	1	1	1
High	Q02241	Kinesin-like protein KIF23 OS=Homo sapiens OX=9606 GN=KIF23 PE=1 SV=3	14	5	5	110	7	960	8.51	2	3	2				2	3	2			
High	Q14004	Cyclin-dependent kinase 13 OS=Homo sapiens OX=9606 GN=CDK13 PE=1 SV=2	14	2	1	164.8	1	1512	9.69	1	2	2	2	2	1	1	1	1	1	1	1
High	Q9BQ39	ATP-dependent RNA helicase DDX50 OS=Homo sapiens OX=9606 GN=DDX50 PE=1 SV=1	14	4	2	82.5	6	737	9.17				1	4	1				1	3	1
High	Q9NZI7	Upstream-binding protein 1 OS=Homo sapiens OX=9606 GN=UBP1 PE=1 SV=1	14	5	4	60.5	13	540	6.35	4	3	4				4	3	4			
High	Q9BWE0	Replication initiator 1 OS=Homo sapiens OX=9606 GN=REPIN1 PE=1 SV=1	14	3	3	63.5	8	567	9.98				1		2				1		2
High	Q8WV41	Sorting nexin-33 OS=Homo sapiens OX=9606 GN=SNX33 PE=1 SV=1	14	4	4	65.2	9	574	6.79			2	2	1	2			2	2	1	2
High	Q9UID6	Zinc finger protein 639 OS=Homo sapiens OX=9606 GN=ZNF639 PE=1 SV=1	14	7	7	56	17	485	5.57				2	2	2				2	2	2
High	Q12830	Nucleosome-remodeling factor subunit BPTF OS=Homo sapiens OX=9606 GN=BPTF PE=1 SV=3	14	7	7	338.1	2	3046	6.54				2	4	3				2	4	3
High	Q02790	Peptidyl-prolyl cis-trans isomerase FKBP4 OS=Homo sapiens OX=9606 GN=FKBP4 PE=1 SV=3	14	5	5	51.8	9	459	5.43	2	3	3	1			2	3	3	1		
High	Q5TFE4	5'-nucleotidase domain-containing protein 1 OS=Homo sapiens OX=9606 GN=NT5DC1 PE=1 SV=1	14	3	3	51.8	8	455	6.35	1	1	1	1	3	1	1	1	1	1	3	1
High	Q16875	6-phosphofructo-2-kinase/fructose-2,6-bisphosphatase 3 OS=Homo sapiens OX=9606 GN=PFKFB3 PE=1 SV=1	14	7	5	59.6	16	520	8.21			1	1	1				1	1	1	
High	Q9NYV4	Cyclin-dependent kinase 12 OS=Homo sapiens OX=9606 GN=CDK12 PE=1 SV=2	14	2	1	164.1	1	1490	9.44	1	2	2	2	2	1	1	1	1	1	1	1
High	P06400	Retinoblastoma-associated protein OS=Homo sapiens OX=9606 GN=RB1 PE=1 SV=2	14	5	5	106.1	6	928	7.94	2	3		1	2		2	3		1	2	
High	Q9P016	Thymocyte nuclear protein 1 OS=Homo sapiens OX=9606 GN=THYN1 PE=1 SV=1	14	4	4	25.7	16	225	9.25	1	1	2	1	2	1	1	1	2	1	2	1
High	Q14152	Eukaryotic translation initiation factor 3 subunit A OS=Homo sapiens OX=9606 GN=EIF3A PE=1 SV=1	14	5	5	166.5	4	1382	6.79	1		2	2	2	1	1		2	2	2	1
High	Q9HB21	Pleckstrin homology domain-containing family A member 1 OS=Homo sapiens OX=9606 GN=PLEKHA1 PE=1 SV=2	14	4	4	45.5	13	404	8.56	2	2	1				2	2	1			
High	Q9BWM7	Sideroflexin-3 OS=Homo sapiens OX=9606 GN=SFXN3 PE=1 SV=3	14	3	3	35.5	10	321	9.1	2	2	3	1		1	2	2	3	1		1
High	Q9Y3B4	Splicing factor 3B subunit 6 OS=Homo sapiens OX=9606 GN=SF3B6 PE=1 SV=1	14	4	4	14.6	38	125	9.38	1	1	1	1	2	1	1	1	1	1	2	1
High	P49750	YLP motif-containing protein 1 OS=Homo sapiens OX=9606 GN=YLPM1 PE=1 SV=4	14	5	5	241.5	4	2146	6.55	3	3	2	1			3	3	2	1		
High	P52434	DNA-directed RNA polymerases I, II, and III subunit RPABC3 OS=Homo sapiens OX=9606 GN=POLR2H PE=1 SV=4	14	3	3	17.1	27	150	4.68		1	1	1	2	2		1	1	1	2	2
High	Q96JP5	E3 ubiquitin-protein ligase ZFP91 OS=Homo sapiens OX=9606 GN=ZFP91 PE=1 SV=1	14	4	4	63.4	10	570	7.36				1		1				1		1
High	Q9P1Z2	Calcium-binding and coiled-coil domain-containing protein 1 OS=Homo sapiens OX=9606 GN=CALCOCO1 PE=1 SV=2	14	4	4	77.3	6	691	4.82	1		2	1	1	1	1		2	1	1	1
High	O60307	Microtubule-associated serine/threonine-protein kinase 3 OS=Homo sapiens OX=9606 GN=MAST3 PE=1 SV=2	14	2	1	143	1	1309	8.06			1	2	3	1			1	1	2	1
High	Q9HCD5	Nuclear receptor coactivator 5 OS=Homo sapiens OX=9606 GN=NCOA5 PE=1 SV=2	14	6	6	65.5	11	579	9.6	4	4	2	1	2	1	4	4	2	1	2	1

High	Q6Y7W6	GRB10-interacting GYF protein 2 OS=Homo sapiens OX=9606 GN=GIGYF2 PE=1 SV=1	14	5	5	150	6	1299	5.54	1	1	2				1	1	2			
High	Q53EP0	Fibronectin type III domain-containing protein 3B OS=Homo sapiens OX=9606 GN=FNDC3B PE=1 SV=2	13	4	4	132.8	4	1204	5.95	1	1	2		2		1	1	2		2	
High	Q96FV9	THO complex subunit 1 OS=Homo sapiens OX=9606 GN=THOC1 PE=1 SV=1	13	5	5	75.6	9	657	4.98				2	1	2				2	1	2
High	Q9Y6D5	Brefeldin A-inhibited guanine nucleotide-exchange protein 2 OS=Homo sapiens OX=9606 GN=ARFGEF2 PE=1 SV=3	13	5	4	201.9	3	1785	6.33	1	1	3				1	1	3			
High	P31944	Caspase-14 OS=Homo sapiens OX=9606 GN=CASP14 PE=1 SV=2	13	6	6	27.7	28	242	5.58				4	5	4				4	5	4
High	O43824	Putative GTP-binding protein 6 OS=Homo sapiens OX=9606 GN=GTPBP6 PE=1 SV=4	13	4	4	56.9	7	516	9.42	1	2		1	1	1	1	2		1	1	1
High	P28838	Cytosol aminopeptidase OS=Homo sapiens OX=9606 GN=LAP3 PE=1 SV=3	13	4	4	56.1	11	519	7.93	2	3		1	1		2	3		1	1	
High	P23497	Nuclear autoantigen Sp-100 OS=Homo sapiens OX=9606 GN=SP100 PE=1 SV=3	13	5	5	100.4	8	879	8.22				1	2	3				1	2	3
High	Q96I15	Selenocysteine lyase OS=Homo sapiens OX=9606 GN=SCLY PE=1 SV=4	13	5	5	48.1	18	445	7.12	1		2			1	1		2			1
High	P30085	UMP-CMP kinase OS=Homo sapiens OX=9606 GN=CMPK1 PE=1 SV=3	13	5	5	22.2	22	196	5.57			1			1			1			1
High	Q9H0H5	Rac GTPase-activating protein 1 OS=Homo sapiens OX=9606 GN=RACGAP1 PE=1 SV=1	13	3	3	71	5	632	8.88	2	2	3		1	1	2	2	3		1	1
High	Q9Y2L1	Exosome complex exonuclease RRP44 OS=Homo sapiens OX=9606 GN=DIS3 PE=1 SV=2	13	5	5	108.9	9	958	7.14	1	1	1				1	1	1			
High	Q10589	Bone marrow stromal antigen 2 OS=Homo sapiens OX=9606 GN=BST2 PE=1 SV=1	13	2	2	19.8	13	180	5.6	2	1	1	1	2	1	1	1	1	1	1	1
High	Q5VT25	Serine/threonine-protein kinase MRCK alpha OS=Homo sapiens OX=9606 GN=CDC42BPA PE=1 SV=1	13	7	3	197.2	5	1732	6.58			1						1			
High	Q15007	Pre-mRNA-splicing regulator WTAP OS=Homo sapiens OX=9606 GN=WTAP PE=1 SV=2	13	5	5	44.2	17	396	5.19		1	1	3	2	2		1	1	3	2	2
High	O95394	Phosphoacetylglucosamine mutase OS=Homo sapiens OX=9606 GN=PGM3 PE=1 SV=1	13	5	5	59.8	8	542	6.25	1	2		2	1		1	2		2	1	
High	P12829	Myosin light chain 4 OS=Homo sapiens OX=9606 GN=MYL4 PE=1 SV=3	13	5	4	21.6	24	197	5.03	1	1	1	5		4	1	1	1	5		4
High	Q9NP72	Ras-related protein Rab-18 OS=Homo sapiens OX=9606 GN=RAB18 PE=1 SV=1	13	5	5	23	30	206	5.24	1	1	1	1		2	1	1	1	1		2
High	Q96RT1	Erbin OS=Homo sapiens OX=9606 GN=ERBIN PE=1 SV=2	13	6	6	158.2	6	1412	5.5	2	3	4		1		2	3	4		1	
High	O75164	Lysine-specific demethylase 4A OS=Homo sapiens OX=9606 GN=KDM4A PE=1 SV=2	13	8	8	120.6	8	1064	5.85					3	2					3	2
High	P51665	26S proteasome non-ATPase regulatory subunit 7 OS=Homo sapiens OX=9606 GN=PSMD7 PE=1 SV=2	13	3	3	37	10	324	6.77			1	2	2	2			1	2	2	2
High	Q96EY5	Multivesicular body subunit 12A OS=Homo sapiens OX=9606 GN=MVB12A PE=1 SV=1	13	4	4	28.8	26	273	8.91	1	1	2	1			1	1	2	1		
High	P63218	Guanine nucleotide-binding protein G(I)/G(S)/G(O) subunit gamma-5 OS=Homo sapiens OX=9606 GN=GNG5 PE=1 SV=3	13	2	2	7.3	24	68	9.85	1	1	2	2	1	1	1	1	2	2	1	1
High	P00533	Epidermal growth factor receptor OS=Homo sapiens OX=9606 GN=EGFR PE=1 SV=2	13	4	3	134.2	6	1210	6.68	2	3	2				2	3	2			
High	P14550	Aldo-keto reductase family 1 member A1 OS=Homo sapiens OX=9606 GN=AKR1A1 PE=1 SV=3	13	2	2	36.6	5	325	6.79	1	1	2	2	2	2	1	1	2	2	2	2
High	O15067	Phosphoribosylformylglycinamidine synthase OS=Homo sapiens OX=9606 GN=PFAS PE=1 SV=4	13	8	8	144.6	7	1338	5.76		1	1		3	1		1	1		3	1
High	Q5RKV6	Exosome complex component MTR3 OS=Homo sapiens OX=9606 GN=EXOSC6 PE=1 SV=1	13	4	4	28.2	16	272	6.28		1	1	2	2	1		1	1	2	2	1
High	Q9BUQ8	Probable ATP-dependent RNA helicase DDX23 OS=Homo sapiens OX=9606 GN=DDX23 PE=1 SV=3	13	4	4	95.5	4	820	9.55		1		1	1	2		1		1	1	1
High	Q6DKI2	Galectin-9C OS=Homo sapiens OX=9606 GN=LGALS9C PE=1 SV=2	13	4	1	39.6	13	356	9.19	1		1	1	2	2	1		1	1	2	2
High	Q9BVL2	Nucleoporin p58/p45 OS=Homo sapiens OX=9606 GN=NUP58 PE=1 SV=1	13	5	5	60.9	9	599	9.33		1		1		1		1		1		1
High	O15511	Actin-related protein 2/3 complex subunit 5 OS=Homo sapiens OX=9606 GN=ARPC5 PE=1 SV=3	13	4	3	16.3	34	151	5.67	2	1	2		1		2	1	2		1	
High	P61224	Ras-related protein Rap-1b OS=Homo sapiens OX=9606 GN=RAP1B PE=1 SV=1	13	3	1	20.8	21	184	5.78	2		1	1	2	1	2		1	1	2	1
High	Q9HBH1	Peptide deformylase, mitochondrial OS=Homo sapiens OX=9606 GN=PDF PE=1 SV=1	13	4	4	27	20	243	9.16	1	1	1				1	1	1			
High	Q8WYP5	Protein ELYS OS=Homo sapiens OX=9606 GN=AHCTF1 PE=1 SV=3	13	5	5	252.3	3	2266	6.6			1	1	1				1	1	1	
High	P02675	Fibrinogen beta chain OS=Homo sapiens OX=9606 GN=FGB PE=1 SV=2	13	6	3	55.9	12	491	8.27				4	4	5				4	4	5
High	P78312	Protein FAM193A OS=Homo sapiens OX=9606 GN=FAM193A PE=1 SV=2	13	6	6	139.9	6	1265	6.48				4	4	4				3	4	4
High	O96017	Serine/threonine-protein kinase Chk2 OS=Homo sapiens OX=9606 GN=CHEK2 PE=1 SV=1	13	5	4	60.9	9	543	5.91	1	1	1	1	3		1	1	1	1	3	
High	Q86YT6	E3 ubiquitin-protein ligase MIB1 OS=Homo sapiens OX=9606 GN=MIB1 PE=1 SV=1	13	4	4	110.1	4	1006	6.92	2	1	2		1		2	1	2		1	
High	P17676	CCAAT/enhancer-binding protein beta OS=Homo sapiens OX=9606 GN=CEBPB PE=1 SV=2	13	4	4	36.1	17	345	8.31	2	2	2		1	2	2	2	2		1	2
High	O43663	Protein regulator of cytokinesis 1 OS=Homo sapiens OX=9606 GN=PRC1 PE=1 SV=2	13	3	3	71.6	6	620	6.68			1	1	2	1			1	1	2	1
High	O14735	CDP-diacylglycerol--inositol 3-phosphatidyltransferase OS=Homo sapiens OX=9606 GN=CDIPT PE=1 SV=1	13	2	2	23.5	10	213	8.03	2	1	1	2	1		2	1	1	2	1	
High	Q16762	Thiosulfate sulfurtransferase OS=Homo sapiens OX=9606 GN=TST PE=1 SV=4	13	6	6	33.4	33	297	7.25	2	3	1				2	3	1			
High	P05198	Eukaryotic translation initiation factor 2 subunit 1 OS=Homo sapiens OX=9606 GN=EIF2S1 PE=1 SV=3	13	6	6	36.1	21	315	5.08	1	2	1	2	1	1	1	2	1	2	1	1
High	O75909	Cyclin-K OS=Homo sapiens OX=9606 GN=CCNK PE=1 SV=2	13	4	4	64.2	11	580	8.41				2	2	1				2	2	1
High	Q6IAA8	Ragulator complex protein LAMTOR1 OS=Homo sapiens OX=9606 GN=LAMTOR1 PE=1 SV=2	13	2	2	17.7	15	161	5.15	2	1	2		1		2	1	2		1	
High	P55735	Protein SEC13 homolog OS=Homo sapiens OX=9606 GN=SEC13 PE=1 SV=3	13	3	3	35.5	15	322	5.48	1			1	1		1			1	1	
High	Q16769	Glutaminyl-peptide cyclotransferase OS=Homo sapiens OX=9606 GN=QPCT PE=1 SV=1	13	3	3	40.9	12	361	6.61	1	1	1	1	2	2	1	1	1	1	2	2
High	O95218	Zinc finger Ran-binding domain-containing protein 2 OS=Homo sapiens OX=9606 GN=ZRANB2 PE=1 SV=2	13	6	6	37.4	24	330	10.01		1	1		2			1	1		2	
High	P13804	Electron transfer flavoprotein subunit alpha, mitochondrial OS=Homo sapiens OX=9606 GN=ETFA PE=1 SV=1	13	6	6	35.1	26	333	8.38	3	4	3				3	4	3			
High	O43772	Mitochondrial carnitine/acylcarnitine carrier protein OS=Homo sapiens OX=9606 GN=SLC25A20 PE=1 SV=1	13	5	5	32.9	19	301	9.41	2	1	4				2	1	4			
High	P41229	Lysine-specific demethylase 5C OS=Homo sapiens OX=9606 GN=KDM5C PE=1 SV=2	13	7	6	175.6	6	1560	5.58	1	1	2		1		1	1	2		1	
High	Q9UQ80	Proliferation-associated protein 2G4 OS=Homo sapiens OX=9606 GN=PA2G4 PE=1 SV=3	13	4	4	43.8	18	394	6.55	1	1	1		3		1	1	1		2	
High	Q9P1U1	Actin-related protein 3B OS=Homo sapiens OX=9606 GN=ACTR3B PE=2 SV=1	13	3	1	47.6	10	418	5.88	2	2	1	1	1	2	2	2	1	1	1	2
High	P18283	Glutathione peroxidase 2 OS=Homo sapiens OX=9606 GN=GPX2 PE=1 SV=3	13	5	5	21.9	33	190	7.78			3	1	2				3	1	2	
High	Q14344	Guanine nucleotide-binding protein subunit alpha-13 OS=Homo sapiens OX=9606 GN=GNA13 PE=1 SV=2	13	3	1	44	8	377	8	1	2	1	2	1	2	1	2	1	2	1	2
High	Q86X76	Deaminated glutathione amidase OS=Homo sapiens OX=9606 GN=NIT1 PE=1 SV=2	13	4	4	35.9	15	327	7.74	1		2		1		1		2		1	
High	Q9UJC5	SH3 domain-binding glutamic acid-rich-like protein 2 OS=Homo sapiens OX=9606 GN=SH3BGRL2 PE=1 SV=2	13	3	3	12.3	36	107	6.71	1	2	3	1	1	1	1	2	3	1	1	1
High	Q14692	Ribosome biogenesis protein BMS1 homolog OS=Homo sapiens OX=9606 GN=BMS1 PE=1 SV=1	13	6	6	145.7	6	1282	6.44					2						2	
High	P09110	3-ketoacyl-CoA thiolase, peroxisomal OS=Homo sapiens OX=9606 GN=ACAA1 PE=1 SV=2	13	7	7	44.3	30	424	8.44		2	1		2			2	1		2	
High	Q9Y3A4	Ribosomal RNA-processing protein 7 homolog A OS=Homo sapiens OX=9606 GN=RRP7A PE=1 SV=2	13	4	4	32.3	23	280	9.58	1	1			1	2	1	1			1	2
High	P39748	Flap endonuclease 1 OS=Homo sapiens OX=9606 GN=FEN1 PE=1 SV=1	13	2	2	42.6	8	380	8.62	2	2	2	1	1	1	2	2	2	1	1	1
High	Q96BS2	Calcineurin B homologous protein 3 OS=Homo sapiens OX=9606 GN=TESC PE=1 SV=3	13	4	4	24.7	21	214	4.93	3	3	2	1		1	3	3	2	1		1
High	O15031	Plexin-B2 OS=Homo sapiens OX=9606 GN=PLXNB2 PE=1 SV=3	13	5	5	205	4	1838	6.24	2	2	2				2	2	2			
High	Q96J01	THO complex subunit 3 OS=Homo sapiens OX=9606 GN=THOC3 PE=1 SV=1	13	3	3	38.7	9	351	6.09		1	2	1	1	1		1	2	1	1	1
High	Q6P179	Endoplasmic reticulum aminopeptidase 2 OS=Homo sapiens OX=9606 GN=ERAP2 PE=1 SV=2	13	7	7	110.4	8	960	6.71	4	1	4			1	4	1	4			1
High	Q9Y3D3	28S ribosomal protein S16, mitochondrial OS=Homo sapiens OX=9606 GN=MRPS16 PE=1 SV=1	13	3	3	15.3	25	137	9.5				2	3	2				2	3	2
High	Q6ISB3	Grainyhead-like protein 2 homolog OS=Homo sapiens OX=9606 GN=GRHL2 PE=1 SV=1	13	5	5	71.1	12	625	6.44	1	1	3	1	1	1	1	1	3	1	1	1
High	Q9BSD7	Cancer-related nucleoside-triphosphatase OS=Homo sapiens OX=9606 GN=NTPCR PE=1 SV=1	13	2	2	20.7	13	190	9.54	1		2	2	1	1	1		2	1	1	1
High	Q8TE77	Protein phosphatase Slingshot homolog 3 OS=Homo sapiens OX=9606 GN=SSH3 PE=1 SV=2	13	4	4	73	6	659	5.3			3	1		1			2	1		1
High	Q9C0F0	Putative Polycomb group protein ASXL3 OS=Homo sapiens OX=9606 GN=ASXL3 PE=2 SV=3	13	3	1	241.8	1	2248	6.14				2	2	2				2	2	2
High	Q8N983	39S ribosomal protein L43, mitochondrial OS=Homo sapiens OX=9606 GN=MRPL43 PE=1 SV=1	13	4	4	23.4	17	215	8.65		1			2	1		1			2	1
High	O14647	Chromodomain-helicase-DNA-binding protein 2 OS=Homo sapiens OX=9606 GN=CHD2 PE=1 SV=2	12	5	4	211.2	3	1828	8.1	4	3	4		1		4	3	4		1	
High	P20020	Plasma membrane calcium-transporting ATPase 1 OS=Homo sapiens OX=9606 GN=ATP2B1 PE=1 SV=4	12	6	6	134.6	7	1220	5.91	2	2	2		1		2	2	2		1	
High	Q13232	Nucleoside diphosphate kinase 3 OS=Homo sapiens OX=9606 GN=NME3 PE=1 SV=2	12	4	4	19	28	169	7.84	2	2		1	1	1	2	2		1	1	1
High	O75940	Survival of motor neuron-related-splicing factor 30 OS=Homo sapiens OX=9606 GN=SMNDC1 PE=1 SV=1	12	3	3	26.7	18	238	7.24				1	2	2				1	2	2
High	Q9H6R4	Nucleolar protein 6 OS=Homo sapiens OX=9606 GN=NOL6 PE=1 SV=2	12	4	4	127.5	5	1146	7.64			1		1	2			1		1	2
High	P10155	RNA-binding protein RO60 OS=Homo sapiens OX=9606 GN=RO60 PE=1 SV=2	12	3	3	60.6	5	538	8.03	1	1	2	1	1	1	1	1	2	1	1	1
High	P21281	V-type proton ATPase subunit B, brain isoform OS=Homo sapiens OX=9606 GN=ATP6V1B2 PE=1 SV=3	12	2	2	56.5	6	511	5.81	2	2	1	1	1	1	2	2	1	1	1	1
High	P02754	SWISS-PROT:P02754 Beta-lactoglobulin - Bos taurus (Bovine).	12	5	5	18.3	31	162	4.92				1	1	1				1	1	1
High	P11498	Pyruvate carboxylase, mitochondrial OS=Homo sapiens OX=9606 GN=PC PE=1 SV=2	12	5	5	129.6	6	1178	6.84	1	2		1		1	1	2		1		1
High	Q8N1B4	Vacuolar protein sorting-associated protein 52 homolog OS=Homo sapiens OX=9606 GN=VPS52 PE=1 SV=1	12	5	5	82.2	7	723	5.99	4	2	5				3	2	5			
High	O95819	Mitogen-activated protein kinase kinase kinase kinase 4 OS=Homo sapiens OX=9606 GN=MAP4K4 PE=1 SV=2	12	5	5	142	5	1239	7.46	1		3	1	2	1	1		3	1	2	1
High	Q99436	Proteasome subunit beta type-7 OS=Homo sapiens OX=9606 GN=PSMB7 PE=1 SV=1	12	3	3	29.9	10	277	7.68				3	3	2				3	3	2
High	Q16698	2,4-dienoyl-CoA reductase [(3E)-enoyl-CoA-producing], mitochondrial OS=Homo sapiens OX=9606 GN=DECR1 PE=1 SV=1	12	3	3	36	14	335	9.28	1	2	2	1	1	1	1	2	2	1	1	1
High	P62491	Ras-related protein Rab-11A OS=Homo sapiens OX=9606 GN=RAB11A PE=1 SV=3	12	3	2	24.4	14	216	6.57	1	1	1	1	1	2	1	1	1	1	1	2
High	Q14739	Delta(14)-sterol reductase LBR OS=Homo sapiens OX=9606 GN=LBR PE=1 SV=2	12	4	4	70.7	7	615	9.36	3	3	2	1	1	1	3	3	2	1	1	1
High	Q92673	Sortilin-related receptor OS=Homo sapiens OX=9606 GN=SORL1 PE=1 SV=2	12	6	6	248.3	3	2214	5.55			1						1			
High	Q53GT1	Kelch-like protein 22 OS=Homo sapiens OX=9606 GN=KLHL22 PE=1 SV=2	12	8	8	71.6	12	634	5.49				2	7	3				2	7	3
High	O43488	Aflatoxin B1 aldehyde reductase member 2 OS=Homo sapiens OX=9606 GN=AKR7A2 PE=1 SV=3	12	4	3	39.6	14	359	7.17		1	2	1		2		1	2	1		2
High	P55209	Nucleosome assembly protein 1-like 1 OS=Homo sapiens OX=9606 GN=NAP1L1 PE=1 SV=1	12	4	3	45.3	14	391	4.46	1	3	2	1			1	3	2	1		
High	O75175	CCR4-NOT transcription complex subunit 3 OS=Homo sapiens OX=9606 GN=CNOT3 PE=1 SV=1	12	3	3	81.8	9	753	6.2	1	2	1	1	1	1	1	2	1	1	1	1
High	Q969G3	SWI/SNF-related matrix-associated actin-dependent regulator of chromatin subfamily E member 1 OS=Homo sapiens OX=9606 GN	12	5	5	46.6	15	411	4.88	1			1			1			1		
High	Q8N122	Regulatory-associated protein of mTOR OS=Homo sapiens OX=9606 GN=RPTOR PE=1 SV=1	12	5	5	148.9	5	1335	6.89	2	3	4				2	3	4			
High	P51398	28S ribosomal protein S29, mitochondrial OS=Homo sapiens OX=9606 GN=DAP3 PE=1 SV=1	12	7	7	45.5	22	398	8.88					1	1					1	1
High	O15484	Calpain-5 OS=Homo sapiens OX=9606 GN=CAPN5 PE=1 SV=2	12	3	3	73.1	7	640	7.64	1	1	3				1	1	2			
High	Q9HAW8	UDP-glucuronosyltransferase 1A10 OS=Homo sapiens OX=9606 GN=UGT1A10 PE=1 SV=1	12	4	4	59.8	8	530	7.3	3	3	2				3	3	2			
High	Q9Y657	Spindlin-1 OS=Homo sapiens OX=9606 GN=SPIN1 PE=1 SV=3	12	4	4	29.6	26	262	6.96	3	4	2	1		1	3	4	2	1		1
High	O00560	Syntenin-1 OS=Homo sapiens OX=9606 GN=SDCBP PE=1 SV=1	12	3	3	32.4	22	298	7.53	2	2	2		1		2	2	2		1	
High	Q9BRP1	Programmed cell death protein 2-like OS=Homo sapiens OX=9606 GN=PDCD2L PE=1 SV=1	12	4	4	39.4	19	358	4.86			1		1	1			1		1	1
High	Q9BYM8	RanBP-type and C3HC4-type zinc finger-containing protein 1 OS=Homo sapiens OX=9606 GN=RBCK1 PE=1 SV=2	12	4	4	57.5	7	510	5.67			2		2	1			2		2	1
High	Q16563	Synaptophysin-like protein 1 OS=Homo sapiens OX=9606 GN=SYPL1 PE=1 SV=1	12	3	3	28.5	20	259	8.43	2	2	4	1		1	2	2	3	1		1
High	Q96LR5	Ubiquitin-conjugating enzyme E2 E2 OS=Homo sapiens OX=9606 GN=UBE2E2 PE=1 SV=1	12	3	3	22.2	17	201	7.71	2	1	1	2	2	2	2	1	1	2	2	2
High	Q9ULD2	Microtubule-associated tumor suppressor 1 OS=Homo sapiens OX=9606 GN=MTUS1 PE=1 SV=2	12	3	3	141.3	3	1270	7.5	1	2	1		1		1	2	1		1	
High	O75396	Vesicle-trafficking protein SEC22b OS=Homo sapiens OX=9606 GN=SEC22B PE=1 SV=5	12	3	3	24.7	16	215	8.51	1			1	1	1	1			1	1	1
High	P78346	Ribonuclease P protein subunit p30 OS=Homo sapiens OX=9606 GN=RPP30 PE=1 SV=1	12	3	3	29.3	15	268	8.91	2	1		2	1	2	2	1		2	1	2
High	Q8WWC4	m-AAA protease-interacting protein 1, mitochondrial OS=Homo sapiens OX=9606 GN=MAIP1 PE=1 SV=1	12	5	5	32.5	19	291	9.17	2	1	5				2	1	5			
High	Q9P013	Spliceosome-associated protein CWC15 homolog OS=Homo sapiens OX=9606 GN=CWC15 PE=1 SV=2	12	3	3	26.6	19	229	5.71	1	2	2		2	1	1	2	2		2	1
High	P84095	Rho-related GTP-binding protein RhoG OS=Homo sapiens OX=9606 GN=RHOG PE=1 SV=1	12	3	2	21.3	23	191	8.12	1	2	1	1	1	1	1	1	1	1	1	1
High	Q8N9U0	Tandem C2 domains nuclear protein OS=Homo sapiens OX=9606 GN=TC2N PE=1 SV=2	12	3	3	55.2	7	490	9.19	1	1			2	1	1	1			2	1
High	P84090	Enhancer of rudimentary homolog OS=Homo sapiens OX=9606 GN=ERH PE=1 SV=1	12	2	2	12.3	27	104	5.92	1	1	1	1	2	1	1	1	1	1	1	1
High	Q9ULX9	Transcription factor MafF OS=Homo sapiens OX=9606 GN=MAFF PE=1 SV=2	12	3	3	17.7	29	164	9.8	1	1	1	2	1	1	1	1	1	2	1	1
High	Q9NYK5	39S ribosomal protein L39, mitochondrial OS=Homo sapiens OX=9606 GN=MRPL39 PE=1 SV=3	12	4	4	38.7	12	338	7.65					2	1					2	1
High	Q8WUA4	General transcription factor 3C polypeptide 2 OS=Homo sapiens OX=9606 GN=GTF3C2 PE=1 SV=2	12	6	6	100.6	8	911	7.31					2	1					2	1
High	Q86U90	YrdC domain-containing protein, mitochondrial OS=Homo sapiens OX=9606 GN=YRDC PE=1 SV=1	12	4	4	29.3	23	279	8.57				1	1	1				1	1	1
High	P08123	Collagen alpha-2(I) chain OS=Homo sapiens OX=9606 GN=COL1A2 PE=1 SV=7	12	2	2	129.2	1	1366	8.95				5	3	4				2	2	1
High	P02679	Fibrinogen gamma chain OS=Homo sapiens OX=9606 GN=FGG PE=1 SV=3	12	3	3	51.5	8	453	5.62				4	4	4				3	3	3
High	P45984	Mitogen-activated protein kinase 9 OS=Homo sapiens OX=9606 GN=MAPK9 PE=1 SV=2	12	2	1	48.1	4	424	5.63		1	1	2		2		1	1	2		2
High	Q9Y2L9	Leucine-rich repeat and calponin homology domain-containing protein 1 OS=Homo sapiens OX=9606 GN=LRCH1 PE=1 SV=3	12	4	4	80.8	10	728	5.94	2	2	3				2	2	3			
High	Q16181	Septin-7 OS=Homo sapiens OX=9606 GN=SEPTIN7 PE=1 SV=2	12	4	3	50.6	13	437	8.63	2	2	2	2		1	2	2	2	2		1
High	Q9UQN3	Charged multivesicular body protein 2b OS=Homo sapiens OX=9606 GN=CHMP2B PE=1 SV=1	12	3	2	23.9	13	213	8.76	1	1	1				1	1	1			
High	P62306	Small nuclear ribonucleoprotein F OS=Homo sapiens OX=9606 GN=SNRPF PE=1 SV=1	12	2	2	9.7	24	86	4.67	1	1	1	2	2	1	1	1	1	2	2	1
High	Q9Y6C9	Mitochondrial carrier homolog 2 OS=Homo sapiens OX=9606 GN=MTCH2 PE=1 SV=1	12	5	5	33.3	16	303	7.97	1		1				1		1			
High	Q8WY36	HMG box transcription factor BBX OS=Homo sapiens OX=9606 GN=BBX PE=1 SV=1	12	6	6	105.1	9	941	8.79		1	1		2	1		1	1		2	1
High	Q9NUP9	Protein lin-7 homolog C OS=Homo sapiens OX=9606 GN=LIN7C PE=1 SV=1	12	5	5	21.8	25	197	8.43	1	1	1	2	1	1	1	1	1	2	1	1
High	Q08379	Golgin subfamily A member 2 OS=Homo sapiens OX=9606 GN=GOLGA2 PE=1 SV=3	12	4	4	113	6	1002	5.02	1			1	2		1			1	2	
High	Q13616	Cullin-1 OS=Homo sapiens OX=9606 GN=CUL1 PE=1 SV=2	12	4	4	89.6	6	776	8	1		1	1	1	1	1		1	1	1	1
High	Q15397	Pumilio homolog 3 OS=Homo sapiens OX=9606 GN=PUM3 PE=1 SV=3	12	4	4	73.5	5	648	9.64				2	3					2	3	
High	P10321	HLA class I histocompatibility antigen, C alpha chain OS=Homo sapiens OX=9606 GN=HLA-C PE=1 SV=3	12	3	2	40.6	12	366	6.04				1	1	2				1	1	2
High	Q96P70	Importin-9 OS=Homo sapiens OX=9606 GN=IPO9 PE=1 SV=3	12	5	5	115.9	6	1041	4.81	4	1	4				4	1	4			
High	Q9UPN7	Serine/threonine-protein phosphatase 6 regulatory subunit 1 OS=Homo sapiens OX=9606 GN=PPP6R1 PE=1 SV=5	12	4	4	96.7	8	881	4.55			3	1		1			3	1		1
High	O75607	Nucleoplasmin-3 OS=Homo sapiens OX=9606 GN=NPM3 PE=1 SV=3	12	3	3	19.3	26	178	4.63	1	1	1				1	1	1			
High	Q9Y3B9	RRP15-like protein OS=Homo sapiens OX=9606 GN=RRP15 PE=1 SV=2	12	4	4	31.5	15	282	5.52		1	1					1	1			
High	P29992	Guanine nucleotide-binding protein subunit alpha-11 OS=Homo sapiens OX=9606 GN=GNA11 PE=1 SV=2	12	4	1	42.1	9	359	5.69		1	1	3	2	2		1	1	3	2	2
High	Q8TB45	DEP domain-containing mTOR-interacting protein OS=Homo sapiens OX=9606 GN=DEPTOR PE=1 SV=2	12	6	6	46.3	19	409	8.07	1						1					
High	O15084	Serine/threonine-protein phosphatase 6 regulatory ankyrin repeat subunit A OS=Homo sapiens OX=9606 GN=ANKRD28 PE=1 SV=	12	2	2	112.9	2	1053	6.25	1	1	2		3		1	1	2		2	
High	Q15124	Phosphoglucomutase-like protein 5 OS=Homo sapiens OX=9606 GN=PGM5 PE=1 SV=2	12	2	2	62.2	4	567	7.21	1	1	2	1	1	1	1	1	1	1	1	1
High	Q15293	Reticulocalbin-1 OS=Homo sapiens OX=9606 GN=RCN1 PE=1 SV=1	12	5	5	38.9	18	331	5	3	4	2				3	4	2			
High	O95881	Thioredoxin domain-containing protein 12 OS=Homo sapiens OX=9606 GN=TXNDC12 PE=1 SV=1	11	3	3	19.2	18	172	5.4			1	2	3	1			1	2	3	1
High	Q9BYG5	Partitioning defective 6 homolog beta OS=Homo sapiens OX=9606 GN=PARD6B PE=1 SV=1	11	2	2	41.2	6	372	5.58	1	1	2	1	1	1	1	1	1	1	1	1
High	P53597	Succinate--CoA ligase [ADP/GDP-forming] subunit alpha, mitochondrial OS=Homo sapiens OX=9606 GN=SUCLG1 PE=1 SV=4	11	2	2	36.2	7	346	8.79	1	1	1	2	1	2	1	1	1	2	1	2
High	Q8ND24	RING finger protein 214 OS=Homo sapiens OX=9606 GN=RNF214 PE=1 SV=2	11	4	4	77.6	5	703	6.95	1	1					1	1				
High	Q15631	Translin OS=Homo sapiens OX=9606 GN=TSN PE=1 SV=1	11	2	2	26.2	11	228	6.44	1		2	1	1		1		2	1	1	
High	P48556	26S proteasome non-ATPase regulatory subunit 8 OS=Homo sapiens OX=9606 GN=PSMD8 PE=1 SV=2	11	3	3	39.6	7	350	9.7				2	2	1				2	2	1
High	Q9C0B1	Alpha-ketoglutarate-dependent dioxygenase FTO OS=Homo sapiens OX=9606 GN=FTO PE=1 SV=3	11	5	5	58.2	11	505	5.22		1	1					1	1			
High	O00217	NADH dehydrogenase [ubiquinone] iron-sulfur protein 8, mitochondrial OS=Homo sapiens OX=9606 GN=NDUFS8 PE=1 SV=1	11	2	2	23.7	8	210	6.34	2	2	1	1	1	1	2	2	1	1	1	1
High	Q96T76	MMS19 nucleotide excision repair protein homolog OS=Homo sapiens OX=9606 GN=MMS19 PE=1 SV=2	11	3	3	113.2	3	1030	6.35	1		3				1		2			
High	Q96ME7	Zinc finger protein 512 OS=Homo sapiens OX=9606 GN=ZNF512 PE=1 SV=2	11	3	3	64.6	7	567	9.76			1		1	1			1		1	1
High	P18031	Tyrosine-protein phosphatase non-receptor type 1 OS=Homo sapiens OX=9606 GN=PTPN1 PE=1 SV=1	11	4	4	49.9	14	435	6.27	3	2	1		1		3	2	1		1	
High	P41227	N-alpha-acetyltransferase 10 OS=Homo sapiens OX=9606 GN=NAA10 PE=1 SV=1	11	4	4	26.4	20	235	5.64	1	1	1	1	1	2	1	1	1	1	1	2
High	Q9NR50	Translation initiation factor eIF-2B subunit gamma OS=Homo sapiens OX=9606 GN=EIF2B3 PE=1 SV=1	11	2	2	50.2	9	452	6.47	1	1	2	1	1		1	1	2	1	1	
High	Q8WTV1	THAP domain-containing protein 3 OS=Homo sapiens OX=9606 GN=THAP3 PE=1 SV=1	11	4	4	27	28	239	10.24				1	2	1				1	2	1
High	Q9Y5Y2	Cytosolic Fe-S cluster assembly factor NUBP2 OS=Homo sapiens OX=9606 GN=NUBP2 PE=1 SV=1	11	3	3	28.8	23	271	5.83	2	2	2		1		2	2	2		1	
High	Q9NZW5	Protein PALS2 OS=Homo sapiens OX=9606 GN=PALS2 PE=1 SV=2	11	3	2	61.1	6	540	6.18	2	2	1		1	1	2	2	1		1	1
High	Q5T8P6	RNA-binding protein 26 OS=Homo sapiens OX=9606 GN=RBM26 PE=1 SV=3	11	3	2	113.5	3	1007	9.16				1	2	1				1	2	1
High	Q7RTV0	PHD finger-like domain-containing protein 5A OS=Homo sapiens OX=9606 GN=PHF5A PE=1 SV=1	11	3	3	12.4	25	110	8.41				2	2	1				2	2	1
High	Q96JQ2	Calmin OS=Homo sapiens OX=9606 GN=CLMN PE=1 SV=1	11	3	3	111.6	3	1002	4.94	2	2	3			1	2	2	3			1
High	Q8WXE9	Stonin-2 OS=Homo sapiens OX=9606 GN=STON2 PE=1 SV=1	11	1	1	101.1	1	905	5.39	2	4	2				1	1	1			
High	P48507	Glutamate--cysteine ligase regulatory subunit OS=Homo sapiens OX=9606 GN=GCLM PE=1 SV=1	11	4	4	30.7	19	274	6.02	1	1	3		1	1	1	1	3		1	1
High	P31151	Protein S100-A7 OS=Homo sapiens OX=9606 GN=S100A7 PE=1 SV=4	11	3	3	11.5	24	101	6.77				3	4	4				3	3	3
High	P67870	Casein kinase II subunit beta OS=Homo sapiens OX=9606 GN=CSNK2B PE=1 SV=1	11	3	3	24.9	20	215	5.55			1	2	2	1			1	2	2	1
High	P29317	Ephrin type-A receptor 2 OS=Homo sapiens OX=9606 GN=EPHA2 PE=1 SV=2	11	6	4	108.2	7	976	6.23		2	1	1	1	1		2	1	1	1	1
High	Q86VP1	Tax1-binding protein 1 OS=Homo sapiens OX=9606 GN=TAX1BP1 PE=1 SV=2	11	5	5	90.8	6	789	5.43	1	1	1		1		1	1	1		1	
High	Q9UK59	Lariat debranching enzyme OS=Homo sapiens OX=9606 GN=DBR1 PE=1 SV=2	11	3	3	61.5	6	544	5.47	1	1	1	1	2	1	1	1	1	1	2	1
High	O14745	Na(+)/H(+) exchange regulatory cofactor NHE-RF1 OS=Homo sapiens OX=9606 GN=SLC9A3R1 PE=1 SV=4	11	3	3	38.8	11	358	5.77		1	2	1		1		1	2	1		1
High	P49458	Signal recognition particle 9 kDa protein OS=Homo sapiens OX=9606 GN=SRP9 PE=1 SV=2	11	2	2	10.1	22	86	7.97	1	2		1	1	2	1	2		1	1	2
High	Q96DV4	39S ribosomal protein L38, mitochondrial OS=Homo sapiens OX=9606 GN=MRPL38 PE=1 SV=2	11	3	3	44.6	10	380	7.53	1	1	2	1	1	1	1	1	2	1	1	1
High	Q9P270	SLAIN motif-containing protein 2 OS=Homo sapiens OX=9606 GN=SLAIN2 PE=1 SV=2	11	3	3	62.5	8	581	9.45		2	2		1	1		2	2		1	1
High	P61163	Alpha-centractin OS=Homo sapiens OX=9606 GN=ACTR1A PE=1 SV=1	11	3	3	42.6	11	376	6.64	2	1					2	1				
High	Q01650	Large neutral amino acids transporter small subunit 1 OS=Homo sapiens OX=9606 GN=SLC7A5 PE=1 SV=2	11	3	3	55	8	507	7.72	1	1	1	2	1	1	1	1	1	2	1	1

High	P54920	Alpha-soluble NSF attachment protein OS=Homo sapiens OX=9606 GN=NAPA PE=1 SV=3	11	4	4	33.2	16	295	5.36	1	1			1	1	1	1			1	1
High	Q10570	Cleavage and polyadenylation specificity factor subunit 1 OS=Homo sapiens OX=9606 GN=CPSF1 PE=1 SV=2	11	5	5	160.8	4	1443	6.4		1			2			1			2	
High	Q3ZAQ7	Vacuolar ATPase assembly integral membrane protein VMA21 OS=Homo sapiens OX=9606 GN=VMA21 PE=1 SV=1	11	2	2	11.3	22	101	7.24	1	1	1	1		1	1	1	1	1		1
High	P47813	Eukaryotic translation initiation factor 1A, X-chromosomal OS=Homo sapiens OX=9606 GN=EIF1AX PE=1 SV=2	11	2	2	16.5	15	144	5.24	1		1	2	2	2	1		1	2	2	2
High	Q9H2G2	STE20-like serine/threonine-protein kinase OS=Homo sapiens OX=9606 GN=SLK PE=1 SV=1	11	4	4	142.6	5	1235	5.15	1	1	2	1		1	1	1	2	1		1
High	Q93008	Probable ubiquitin carboxyl-terminal hydrolase FAF-X OS=Homo sapiens OX=9606 GN=USP9X PE=1 SV=4	11	7	4	290.3	3	2554	5.8	1	2	2				1	2	2			
High	O60749	Sorting nexin-2 OS=Homo sapiens OX=9606 GN=SNX2 PE=1 SV=2	11	4	2	58.4	8	519	5.12	1				3		1				3	
High	P04732	Metallothionein-1E OS=Homo sapiens OX=9606 GN=MT1E PE=1 SV=1	11	2	1	6	52	61	7.96	1	1	1	2	2	1	1	1	1	2	2	1
High	O14578	Citron Rho-interacting kinase OS=Homo sapiens OX=9606 GN=CIT PE=1 SV=2	11	4	4	231.3	2	2027	6.57	1	1		1	4	2	1	1		1	4	2
High	Q14596	Next to BRCA1 gene 1 protein OS=Homo sapiens OX=9606 GN=NBR1 PE=1 SV=3	11	2	2	107.3	3	966	5.12	1	1	2				1	1	1			
High	Q14008	Cytoskeleton-associated protein 5 OS=Homo sapiens OX=9606 GN=CKAP5 PE=1 SV=3	11	3	3	225.4	1	2032	7.8			1		2	2			1		2	2
High	Q8NFQ8	Torsin-1A-interacting protein 2 OS=Homo sapiens OX=9606 GN=TOR1AIP2 PE=1 SV=1	11	3	3	51.2	9	470	4.96	1	1	1	1	1	1	1	1	1	1	1	1
High	Q99733	Nucleosome assembly protein 1-like 4 OS=Homo sapiens OX=9606 GN=NAP1L4 PE=1 SV=1	11	5	4	42.8	17	375	4.69		2	3	1	1			2	3	1	1	
High	Q9H9A6	Leucine-rich repeat-containing protein 40 OS=Homo sapiens OX=9606 GN=LRRC40 PE=1 SV=1	11	3	3	68.2	5	602	6.43	1	1	1	3	2	2	1	1	1	3	2	2
High	O00264	Membrane-associated progesterone receptor component 1 OS=Homo sapiens OX=9606 GN=PGRMC1 PE=1 SV=3	11	3	3	21.7	16	195	4.7	1	1	1		1	1	1	1	1		1	1
High	Q96S19	Methyltransferase-like 26 OS=Homo sapiens OX=9606 GN=METTL26 PE=1 SV=2	11	4	4	22.6	24	204	7.83			2	1	2	1			2	1	2	1
High	P98175	RNA-binding protein 10 OS=Homo sapiens OX=9606 GN=RBM10 PE=1 SV=3	11	6	6	103.5	7	930	5.97												
High	Q9H0E2	Toll-interacting protein OS=Homo sapiens OX=9606 GN=TOLLIP PE=1 SV=1	11	3	3	30.3	15	274	5.97	3	3	2				3	3	2			
High	Q00059	Transcription factor A, mitochondrial OS=Homo sapiens OX=9606 GN=TFAM PE=1 SV=1	11	2	2	29.1	7	246	9.72			1	1	1	2			1	1	1	2
High	Q2TAL8	Transcriptional regulator QRICH1 OS=Homo sapiens OX=9606 GN=QRICH1 PE=1 SV=1	11	3	3	86.4	3	776	5.87				2	1	2				2	1	2
High	Q13643	Four and a half LIM domains protein 3 OS=Homo sapiens OX=9606 GN=FHL3 PE=1 SV=4	11	5	5	31.2	20	280	6.2				1	3	1				1	3	1
High	P01100	Protein c-Fos OS=Homo sapiens OX=9606 GN=FOS PE=1 SV=1	11	3	3	40.7	11	380	4.87		1			2			1			2	
High	Q00796	Sorbitol dehydrogenase OS=Homo sapiens OX=9606 GN=SORD PE=1 SV=4	11	4	4	38.3	15	357	7.97	1	1	2	1		1	1	1	2	1		1
High	Q92667	A-kinase anchor protein 1, mitochondrial OS=Homo sapiens OX=9606 GN=AKAP1 PE=1 SV=1	11	3	3	97.3	4	903	4.94	1		1	1	1	1	1		1	1	1	1
High	P29144	Tripeptidyl-peptidase 2 OS=Homo sapiens OX=9606 GN=TPP2 PE=1 SV=4	11	6	6	138.3	7	1249	6.32		1			1			1			1	
High	Q8N3C0	Activating signal cointegrator 1 complex subunit 3 OS=Homo sapiens OX=9606 GN=ASCC3 PE=1 SV=3	11	5	5	251.3	3	2202	7.09	2	3	1				2	3	1			
High	P19388	DNA-directed RNA polymerases I, II, and III subunit RPABC1 OS=Homo sapiens OX=9606 GN=POLR2E PE=1 SV=4	11	4	4	24.5	23	210	5.95			1	1		2			1	1		2
High	O95837	Guanine nucleotide-binding protein subunit alpha-14 OS=Homo sapiens OX=9606 GN=GNA14 PE=1 SV=1	11	4	1	41.5	9	355	6.07		1	1	3	1	2		1	1	3	1	2
High	P78406	mRNA export factor RAE1 OS=Homo sapiens OX=9606 GN=RAE1 PE=1 SV=1	11	6	6	40.9	24	368	7.83	1				1		1				1	
High	Q9HBL0	Tensin-1 OS=Homo sapiens OX=9606 GN=TNS1 PE=1 SV=2	11	3	2	185.6	2	1735	7.75	1	2	1	1	2	1	1	1	1	1	2	1
High	Q659A1	Little elongation complex subunit 2 OS=Homo sapiens OX=9606 GN=ICE2 PE=1 SV=2	11	5	5	109.9	5	982	7.09				3	3	3				3	3	3
High	Q9H4A3	Serine/threonine-protein kinase WNK1 OS=Homo sapiens OX=9606 GN=WNK1 PE=1 SV=2	11	5	5	250.6	2	2382	6.34			1		1	1			1		1	1
High	Q9NPG3	Ubinuclein-1 OS=Homo sapiens OX=9606 GN=UBN1 PE=1 SV=2	11	3	3	121.4	3	1134	9.33	2	2	1	1	1	1	2	2	1	1	1	1
High	Q9H6Z4	Ran-binding protein 3 OS=Homo sapiens OX=9606 GN=RANBP3 PE=1 SV=1	11	3	3	60.2	7	567	4.78	1	1	1	1	1	1	1	1	1	1	1	1
High	Q9NYL2	Mitogen-activated protein kinase kinase kinase 20 OS=Homo sapiens OX=9606 GN=MAP3K20 PE=1 SV=3	11	4	4	91.1	5	800	7.87	2	1	1	1	1	1	2	1	1	1	1	1
High	Q16186	Proteasomal ubiquitin receptor ADRM1 OS=Homo sapiens OX=9606 GN=ADRM1 PE=1 SV=2	11	3	3	42.1	11	407	5.07			1	2	1	1			1	2	1	1
High	Q9UKN8	General transcription factor 3C polypeptide 4 OS=Homo sapiens OX=9606 GN=GTF3C4 PE=1 SV=2	11	4	4	91.9	6	822	6.65	1	1	1		1		1	1	1		1	
High	O75494	Serine/arginine-rich splicing factor 10 OS=Homo sapiens OX=9606 GN=SRSF10 PE=1 SV=1	11	4	4	31.3	16	262	11.27	2	1	1	1		1	2	1	1	1		1
High	Q8TBB5	Kelch domain-containing protein 4 OS=Homo sapiens OX=9606 GN=KLHDC4 PE=1 SV=1	11	2	2	57.9	4	520	5.72		1	1	1	1	1		1	1	1	1	1
High	P01111	GTPase NRas OS=Homo sapiens OX=9606 GN=NRAS PE=1 SV=1	11	3	3	21.2	17	189	5.17	1	1	3		1		1	1	2		1	
High	P30566	Adenylosuccinate lyase OS=Homo sapiens OX=9606 GN=ADSL PE=1 SV=2	11	4	4	54.9	13	484	7.11			2	1	1	1			2	1	1	1
High	Q9H307	Pinin OS=Homo sapiens OX=9606 GN=PNN PE=1 SV=5	11	3	3	81.6	4	717	7.14	1		1	1	3	2	1		1	1	3	2
High	P02676	SWISS-PROT:P02676 (Bos taurus) similar to Fibrinogen beta chain precursor	11	4	1	56.4	8	495	8.22				4	3	4				4	3	4
High	Q99613	Eukaryotic translation initiation factor 3 subunit C OS=Homo sapiens OX=9606 GN=EIF3C PE=1 SV=1	11	3	3	105.3	4	913	5.68	2	2	1				2	2	1			
High	Q9BPW8	Protein NipSnap homolog 1 OS=Homo sapiens OX=9606 GN=NIPSNAP1 PE=1 SV=1	11	2	2	33.3	7	284	9.31	2	1	1	1	1	1	2	1	1	1	1	1
High	P31350	Ribonucleoside-diphosphate reductase subunit M2 OS=Homo sapiens OX=9606 GN=RRM2 PE=1 SV=1	11	6	6	44.8	22	389	5.38	1	2	1		1	1	1	2	1		1	1
High	O94929	Actin-binding LIM protein 3 OS=Homo sapiens OX=9606 GN=ABLIM3 PE=1 SV=3	11	5	5	77.8	10	683	8.54	3	2	3				3	2	3			
High	Q9BZL4	Protein phosphatase 1 regulatory subunit 12C OS=Homo sapiens OX=9606 GN=PPP1R12C PE=1 SV=1	11	4	4	84.8	10	782	5.57	1	2	3				1	2	3			
High	Q9NVI1	Fanconi anemia group I protein OS=Homo sapiens OX=9606 GN=FANCI PE=1 SV=4	10	3	3	149.2	3	1328	6.74	1	1	1		1		1	1	1		1	
High	O95251	Histone acetyltransferase KAT7 OS=Homo sapiens OX=9606 GN=KAT7 PE=1 SV=1	10	3	2	70.6	6	611	8.85		1		1	1	1		1		1	1	1
High	Q9UQ88	Cyclin-dependent kinase 11A OS=Homo sapiens OX=9606 GN=CDK11A PE=1 SV=4	10	5	4	91.3	7	783	5.36					4	1					4	1
High	Q9NPE3	H/ACA ribonucleoprotein complex subunit 3 OS=Homo sapiens OX=9606 GN=NOP10 PE=1 SV=1	10	2	2	7.7	44	64	9.99			2	1	1	1			2	1	1	1
High	O75165	DnaJ homolog subfamily C member 13 OS=Homo sapiens OX=9606 GN=DNAJC13 PE=1 SV=5	10	6	6	254.3	4	2243	6.74	4	2	2				2	2	2			
High	O60343	TBC1 domain family member 4 OS=Homo sapiens OX=9606 GN=TBC1D4 PE=1 SV=2	10	6	6	146.5	6	1298	7.01	1						1					
High	P98172	Ephrin-B1 OS=Homo sapiens OX=9606 GN=EFNB1 PE=1 SV=1	10	2	2	38	9	346	8.94	1	1	1	2		2	1	1	1	2		2
High	Q9H8H2	Probable ATP-dependent RNA helicase DDX31 OS=Homo sapiens OX=9606 GN=DDX31 PE=1 SV=2	10	5	5	94	6	851	9.99												
High	Q9UNH7	Sorting nexin-6 OS=Homo sapiens OX=9606 GN=SNX6 PE=1 SV=1	10	3	3	46.6	9	406	6.16			2	2	3	1			2	1	2	1
High	P16333	Cytoplasmic protein NCK1 OS=Homo sapiens OX=9606 GN=NCK1 PE=1 SV=1	10	3	3	42.8	13	377	6.47	3	3	3				3	3	3			
High	P62273	40S ribosomal protein S29 OS=Homo sapiens OX=9606 GN=RPS29 PE=1 SV=2	10	1	1	6.7	13	56	10.13	1	1	1	2	1	1	1	1	1	1	1	1
High	Q06265	Exosome complex component RRP45 OS=Homo sapiens OX=9606 GN=EXOSC9 PE=1 SV=3	10	2	2	48.9	5	439	5.29				2	2	1				2	2	1
High	P01859	Immunoglobulin heavy constant gamma 2 OS=Homo sapiens OX=9606 GN=IGHG2 PE=1 SV=2	10	2	1	35.9	6	326	7.59		1		2	2	2		1		2	2	2
High	Q04760	Lactoylglutathione lyase OS=Homo sapiens OX=9606 GN=GLO1 PE=1 SV=4	10	4	4	20.8	23	184	5.31	2	3	1				2	3	1			
High	O43818	U3 small nucleolar RNA-interacting protein 2 OS=Homo sapiens OX=9606 GN=RRP9 PE=1 SV=1	10	2	2	51.8	4	475	7.85				1	1	2				1	1	2
High	O75376	Nuclear receptor corepressor 1 OS=Homo sapiens OX=9606 GN=NCOR1 PE=1 SV=2	10	6	6	270	4	2440	7.11					1						1	
High	Q9Y6Q5	AP-1 complex subunit mu-2 OS=Homo sapiens OX=9606 GN=AP1M2 PE=1 SV=4	10	2	2	48.1	4	423	8.22	2	2	2				2	2	2			
High	Q9Y6K9	NF-kappa-B essential modulator OS=Homo sapiens OX=9606 GN=IKBKG PE=1 SV=2	10	2	2	48.2	7	419	5.71	2	1	2		1		2	1	2		1	
High	P53582	Methionine aminopeptidase 1 OS=Homo sapiens OX=9606 GN=METAP1 PE=1 SV=2	10	2	2	43.2	5	386	7.17	1	1	1		1	1	1	1	1		1	1
High	Q13356	RING-type E3 ubiquitin-protein ligase PPIL2 OS=Homo sapiens OX=9606 GN=PPIL2 PE=1 SV=1	10	5	5	58.8	12	520	8.78	1	1	1	1	1	1	1	1	1	1	1	1
High	Q9H2U1	ATP-dependent DNA/RNA helicase DHX36 OS=Homo sapiens OX=9606 GN=DHX36 PE=1 SV=2	10	4	4	114.7	5	1008	7.68		1	2		1	1		1	2		1	1
High	Q9Y6M4	Casein kinase I isoform gamma-3 OS=Homo sapiens OX=9606 GN=CSNK1G3 PE=1 SV=2	10	3	1	51.4	6	447	9.22	2	2	3				1	1	2			
High	Q96S97	Myeloid-associated differentiation marker OS=Homo sapiens OX=9606 GN=MYADM PE=1 SV=2	10	2	2	35.3	10	322	8.15	1	1	1			1	1	1	1			1
High	Q86W42	THO complex subunit 6 homolog OS=Homo sapiens OX=9606 GN=THOC6 PE=1 SV=1	10	5	5	37.5	18	341	7.43				1	1					1	1	
High	Q9UKX7	Nuclear pore complex protein Nup50 OS=Homo sapiens OX=9606 GN=NUP50 PE=1 SV=2	10	4	4	50.1	15	468	7.06			1	1					1	1		
High	P60228	Eukaryotic translation initiation factor 3 subunit E OS=Homo sapiens OX=9606 GN=EIF3E PE=1 SV=1	10	5	5	52.2	15	445	6.04	2	1	2				2	1	2			
High	Q9NPH0	Lysophosphatidic acid phosphatase type 6 OS=Homo sapiens OX=9606 GN=ACP6 PE=1 SV=3	10	4	4	48.9	12	428	6.47					1						1	
High	P49736	DNA replication licensing factor MCM2 OS=Homo sapiens OX=9606 GN=MCM2 PE=1 SV=4	10	2	2	101.8	3	904	5.52	1	2	1	1	1		1	2	1	1	1	
High	Q6DD87	Zinc finger protein 787 OS=Homo sapiens OX=9606 GN=ZNF787 PE=1 SV=4	10	4	4	40.4	15	382	7.96		1	1		1	1		1	1		1	1
High	Q9BQ61	Telomerase RNA component interacting RNase OS=Homo sapiens OX=9606 GN=TRIR PE=1 SV=1	10	2	2	18.4	12	176	9.44	1	1	1	1	2	1	1	1	1	1	2	1
High	P27216	Annexin A13 OS=Homo sapiens OX=9606 GN=ANXA13 PE=1 SV=3	10	4	3	35.4	15	316	5.6	1	1	1	1			1	1	1	1		
High	P27448	MAP/microtubule affinity-regulating kinase 3 OS=Homo sapiens OX=9606 GN=MARK3 PE=1 SV=5	10	4	1	84.4	6	753	9.57	2	1	1				2	1	1			
High	Q7Z3C6	Autophagy-related protein 9A OS=Homo sapiens OX=9606 GN=ATG9A PE=1 SV=3	10	3	3	94.4	5	839	6.67	3	3	1				3	3	1			
High	Q9NTI5	Sister chromatid cohesion protein PDS5 homolog B OS=Homo sapiens OX=9606 GN=PDS5B PE=1 SV=1	10	4	4	164.6	3	1447	8.47	2	2	2				2	2	1			
High	Q9Y512	Sorting and assembly machinery component 50 homolog OS=Homo sapiens OX=9606 GN=SAMM50 PE=1 SV=3	10	5	5	51.9	18	469	6.9	1	1			1		1	1			1	
High	A1L0T0	2-hydroxyacyl-CoA lyase 2 OS=Homo sapiens OX=9606 GN=ILVBL PE=1 SV=2	10	6	6	67.8	19	632	8.15	1	1	2				1	1	2			
High	O75489	NADH dehydrogenase [ubiquinone] iron-sulfur protein 3, mitochondrial OS=Homo sapiens OX=9606 GN=NDUFS3 PE=1 SV=1	10	4	4	30.2	20	264	7.5	2	2	2		1	1	2	2	2		1	1
High	P52815	39S ribosomal protein L12, mitochondrial OS=Homo sapiens OX=9606 GN=MRPL12 PE=1 SV=2	10	2	2	21.3	11	198	8.87				1	2	1				1	2	1
High	P61966	AP-1 complex subunit sigma-1A OS=Homo sapiens OX=9606 GN=AP1S1 PE=1 SV=1	10	3	3	18.7	20	158	5.73	1	1	1	1	1	1	1	1	1	1	1	1
High	Q15025	TNFAIP3-interacting protein 1 OS=Homo sapiens OX=9606 GN=TNIP1 PE=1 SV=2	10	4	4	71.8	9	636	6.62	1	1	2				1	1	2			
High	Q8IX12	Cell division cycle and apoptosis regulator protein 1 OS=Homo sapiens OX=9606 GN=CCAR1 PE=1 SV=2	10	2	2	132.7	1	1150	5.76	1	1	1		2		1	1	1		2	
High	Q9BYN8	28S ribosomal protein S26, mitochondrial OS=Homo sapiens OX=9606 GN=MRPS26 PE=1 SV=1	10	5	5	24.2	22	205	10.39						1						1
High	Q96CP2	FLYWCH family member 2 OS=Homo sapiens OX=9606 GN=FLYWCH2 PE=1 SV=1	10	2	2	14.6	16	140	8.46	2	1	2	1	1		2	1	2	1	1	
High	Q6IA17	Single Ig IL-1-related receptor OS=Homo sapiens OX=9606 GN=SIGIRR PE=1 SV=3	10	4	4	45.7	14	410	6.46		1	1					1	1			
High	Q13011	Delta(3,5)-Delta(2,4)-dienoyl-CoA isomerase, mitochondrial OS=Homo sapiens OX=9606 GN=ECH1 PE=1 SV=2	10	3	3	35.8	11	328	8	1	1	3	1		1	1	1	3	1		1
High	Q9UL25	Ras-related protein Rab-21 OS=Homo sapiens OX=9606 GN=RAB21 PE=1 SV=3	10	2	2	24.3	9	225	7.94		1	2		1	1		1	2		1	1
High	Q15019	Septin-2 OS=Homo sapiens OX=9606 GN=SEPTIN2 PE=1 SV=1	10	4	4	41.5	11	361	6.6	3	2	2				3	2	2			
High	Q9BVJ7	Dual specificity protein phosphatase 23 OS=Homo sapiens OX=9606 GN=DUSP23 PE=1 SV=1	10	3	3	16.6	26	150	8.21				1	1	1				1	1	1
High	P49674	Casein kinase I isoform epsilon OS=Homo sapiens OX=9606 GN=CSNK1E PE=1 SV=1	10	3	3	47.3	9	416	9.66			1		1	1			1		1	1
High	Q13206	Probable ATP-dependent RNA helicase DDX10 OS=Homo sapiens OX=9606 GN=DDX10 PE=1 SV=2	10	1	1	100.8	2	875	8.63	1	1	1	1		1	1	1	1	1		1
High	Q9Y3P9	Rab GTPase-activating protein 1 OS=Homo sapiens OX=9606 GN=RABGAP1 PE=1 SV=3	10	3	3	121.7	4	1069	5.25	1	1	1		1		1	1	1		1	
High	A0MZ66	Shootin-1 OS=Homo sapiens OX=9606 GN=SHTN1 PE=1 SV=4	10	3	3	71.6	6	631	5.33	1	1	2		1	2	1	1	2		1	2
High	Q9Y6D6	Brefeldin A-inhibited guanine nucleotide-exchange protein 1 OS=Homo sapiens OX=9606 GN=ARFGEF1 PE=1 SV=2	10	3	2	208.6	1	1849	5.85	1	1	2				1	1	2			
High	O76031	ATP-dependent Clp protease ATP-binding subunit clpX-like, mitochondrial OS=Homo sapiens OX=9606 GN=CLPX PE=1 SV=2	10	7	7	69.2	13	633	7.58	1	4	3				1	4	3			
High	Q9H0U6	39S ribosomal protein L18, mitochondrial OS=Homo sapiens OX=9606 GN=MRPL18 PE=1 SV=1	10	2	2	20.6	9	180	9.54	2	1	2		1		2	1	2		1	
High	O60684	Importin subunit alpha-7 OS=Homo sapiens OX=9606 GN=KPNA6 PE=1 SV=1	10	4	1	60	10	536	4.98	1	3			1		1	3			1	
High	Q86Y56	Dynein axonemal assembly factor 5 OS=Homo sapiens OX=9606 GN=DNAAF5 PE=1 SV=4	10	5	5	93.5	9	855	6.42	1	2	1				1	2	1			
High	Q6P1X5	Transcription initiation factor TFIID subunit 2 OS=Homo sapiens OX=9606 GN=TAF2 PE=1 SV=3	10	3	3	136.9	4	1199	8.19	1	1	2				1	1	2			
High	Q9Y3C6	Peptidyl-prolyl cis-trans isomerase-like 1 OS=Homo sapiens OX=9606 GN=PPIL1 PE=1 SV=1	10	2	2	18.2	19	166	7.99	1	1	1	1	1	1	1	1	1	1	1	1
High	Q9P267	Methyl-CpG-binding domain protein 5 OS=Homo sapiens OX=9606 GN=MBD5 PE=1 SV=3	10	3	3	159.8	4	1494	8.98				1	1					1	1	
High	Q6PD62	RNA polymerase-associated protein CTR9 homolog OS=Homo sapiens OX=9606 GN=CTR9 PE=1 SV=1	10	3	3	133.4	3	1173	6.77					1	1					1	1
High	Q92665	28S ribosomal protein S31, mitochondrial OS=Homo sapiens OX=9606 GN=MRPS31 PE=1 SV=3	10	3	3	45.3	7	395	9.29			1	2	1	2			1	2	1	2
High	Q16539	Mitogen-activated protein kinase 14 OS=Homo sapiens OX=9606 GN=MAPK14 PE=1 SV=3	10	2	1	41.3	4	360	5.78		1	1	1		2		1	1	1		2
High	Q6P161	39S ribosomal protein L54, mitochondrial OS=Homo sapiens OX=9606 GN=MRPL54 PE=1 SV=1	10	1	1	15.8	7	138	9.6		1	1	1	1	1		1	1	1	1	1
High	P17480	Nucleolar transcription factor 1 OS=Homo sapiens OX=9606 GN=UBTF PE=1 SV=1	10	6	6	89.4	10	764	5.81	2	2	1			1	2	2	1			1
High	P51798	H(+)/Cl(-) exchange transporter 7 OS=Homo sapiens OX=9606 GN=CLCN7 PE=1 SV=2	10	3	3	88.6	3	805	8.43	3	3	1	1			3	3	1	1		
High	Q92615	La-related protein 4B OS=Homo sapiens OX=9606 GN=LARP4B PE=1 SV=3	10	6	6	80.5	9	738	6.92	1	1	1		1		1	1	1		1	
High	Q5T9L3	Protein wntless homolog OS=Homo sapiens OX=9606 GN=WLS PE=1 SV=2	10	3	3	62.2	7	541	7.36	2	2	2				2	2	2			
High	Q8NI36	WD repeat-containing protein 36 OS=Homo sapiens OX=9606 GN=WDR36 PE=1 SV=1	10	6	6	105.3	8	951	7.53			1		2				1		2	
High	Q9Y5X3	Sorting nexin-5 OS=Homo sapiens OX=9606 GN=SNX5 PE=1 SV=1	10	2	2	46.8	4	404	6.76	2	1	1	1	1	1	2	1	1	1	1	1
High	Q9HCP0	Casein kinase I isoform gamma-1 OS=Homo sapiens OX=9606 GN=CSNK1G1 PE=1 SV=1	10	3	1	48.5	9	422	9.01	2	2	2				1	1	1			
High	P02795	Metallothionein-2 OS=Homo sapiens OX=9606 GN=MT2A PE=1 SV=1	10	2	1	6	52	61	7.83	1	1	1	1	2	1	1	1	1	1	2	1
High	P12270	Nucleoprotein TPR OS=Homo sapiens OX=9606 GN=TPR PE=1 SV=3	10	3	3	267.1	2	2363	5.02	1	1	1				1	1	1			
High	P53680	AP-2 complex subunit sigma OS=Homo sapiens OX=9606 GN=AP2S1 PE=1 SV=2	10	2	2	17	11	142	6.18	2	1	1	1	1	1	2	1	1	1	1	1
High	Q13907	Isopentenyl-diphosphate Delta-isomerase 1 OS=Homo sapiens OX=9606 GN=IDI1 PE=1 SV=2	10	3	3	26.3	18	227	6.34	2	3	3				2	3	3			
High	P04040	Catalase OS=Homo sapiens OX=9606 GN=CAT PE=1 SV=3	10	4	4	59.7	10	527	7.39	2	2	2		1		2	2	2		1	
High	O00192	Armadillo repeat protein deleted in velo-cardio-facial syndrome OS=Homo sapiens OX=9606 GN=ARVCF PE=1 SV=1	10	3	3	104.6	6	962	6.81	2	2	2				2	2	2			
High	Q96CF2	Charged multivesicular body protein 4c OS=Homo sapiens OX=9606 GN=CHMP4C PE=1 SV=1	10	2	2	26.4	11	233	6.07	1	2	1				1	1	1			
High	Q05209	Tyrosine-protein phosphatase non-receptor type 12 OS=Homo sapiens OX=9606 GN=PTPN12 PE=1 SV=3	10	7	7	88.1	12	780	5.62	1	1	1		2	2	1	1	1		2	2
High	Q9Y4G6	Talin-2 OS=Homo sapiens OX=9606 GN=TLN2 PE=1 SV=4	9	3	1	271.4	1	2542	5.57	1		1		1		1		1		1	
High	Q6ZMG9	Ceramide synthase 6 OS=Homo sapiens OX=9606 GN=CERS6 PE=1 SV=1	9	1	1	44.9	2	384	7.65	1	1	1	1	1	1	1	1	1	1	1	1
High	Q92567	Protein FAM168A OS=Homo sapiens OX=9606 GN=FAM168A PE=1 SV=2	9	2	2	26.2	7	244	8.98	1	1	1	1	1	2	1	1	1	1	1	2
High	P80297	Metallothionein-1X OS=Homo sapiens OX=9606 GN=MT1X PE=1 SV=1	9	2	1	6.1	52	61	7.96	1	1	1	2	1		1	1	1	2	1	
High	Q9BVM4	Gamma-glutamylaminecyclotransferase OS=Homo sapiens OX=9606 GN=GGACT PE=1 SV=2	9	2	1	17.3	14	153	6.87				1	1	1				1	1	1
High	Q9NXE4	Sphingomyelin phosphodiesterase 4 OS=Homo sapiens OX=9606 GN=SMPD4 PE=1 SV=3	9	4	4	97.7	7	866	8.27	2	1	2				2	1	2			
High	P62891	60S ribosomal protein L39 OS=Homo sapiens OX=9606 GN=RPL39 PE=1 SV=2	9	1	1	6.4	20	51	12.56	1	1	1	1	1	1	1	1	1	1	1	1
High	Q03393	6-pyruvoyl tetrahydrobiopterin synthase OS=Homo sapiens OX=9606 GN=PTS PE=1 SV=1	9	3	3	16.4	21	145	6.68	1	1	1	1	1	1	1	1	1	1	1	1
High	Q16658	Fascin OS=Homo sapiens OX=9606 GN=FSCN1 PE=1 SV=3	9	2	2	54.5	5	493	7.24	1	1	1	2	2	2	1	1	1	2	2	2
High	Q96N66	Lysophospholipid acyltransferase 7 OS=Homo sapiens OX=9606 GN=MBOAT7 PE=1 SV=2	9	3	3	52.7	8	472	8.97	1	1	1				1	1	1			
High	Q9Y2S7	Polymerase delta-interacting protein 2 OS=Homo sapiens OX=9606 GN=POLDIP2 PE=1 SV=1	9	4	4	42	14	368	8.63	1	1			1	1	1	1			1	1
High	Q9BXW7	Haloacid dehalogenase-like hydrolase domain-containing 5 OS=Homo sapiens OX=9606 GN=HDHD5 PE=1 SV=1	9	3	3	46.3	9	423	8.13		1		1		1		1		1		1
High	P36405	ADP-ribosylation factor-like protein 3 OS=Homo sapiens OX=9606 GN=ARL3 PE=1 SV=2	9	4	4	20.4	35	182	7.24	1	2					1	2				
High	Q6P6C2	RNA demethylase ALKBH5 OS=Homo sapiens OX=9606 GN=ALKBH5 PE=1 SV=2	9	2	2	44.2	5	394	9.09			1	2	2	1			1	2	2	1
High	O15160	DNA-directed RNA polymerases I and III subunit RPAC1 OS=Homo sapiens OX=9606 GN=POLR1C PE=1 SV=1	9	3	3	39.2	10	346	5.5			1			1			1			1
High	Q15334	Lethal(2) giant larvae protein homolog 1 OS=Homo sapiens OX=9606 GN=LLGL1 PE=1 SV=3	9	4	4	115.3	5	1064	6.29			1		1	1			1		1	1
High	P42766	60S ribosomal protein L35 OS=Homo sapiens OX=9606 GN=RPL35 PE=1 SV=2	9	1	1	14.5	8	123	11.05	1	1	1	1	1	1	1	1	1	1	1	1
High	Q9Y4L1	Hypoxia up-regulated protein 1 OS=Homo sapiens OX=9606 GN=HYOU1 PE=1 SV=1	9	4	4	111.3	8	999	5.22	2	1	3				2	1	3			
High	Q12834	Cell division cycle protein 20 homolog OS=Homo sapiens OX=9606 GN=CDC20 PE=1 SV=2	9	1	1	54.7	3	499	9.23	1	1	1	1	1	1	1	1	1	1	1	1
High	Q96JH7	Deubiquitinating protein VCPIP1 OS=Homo sapiens OX=9606 GN=VCPIP1 PE=1 SV=2	9	3	3	134.2	2	1222	7.2			1		1	2			1		1	2
High	Q13595	Transformer-2 protein homolog alpha OS=Homo sapiens OX=9606 GN=TRA2A PE=1 SV=1	9	5	5	32.7	20	282	11.27	1	1	2		1	2	1	1	2		1	2
High	Q9UMZ2	Synergin gamma OS=Homo sapiens OX=9606 GN=SYNRG PE=1 SV=2	9	2	2	140.6	2	1314	5.03	2	2	1		1		2	2	1		1	
High	Q8WXG6	MAP kinase-activating death domain protein OS=Homo sapiens OX=9606 GN=MADD PE=1 SV=2	9	3	3	183.2	3	1647	6.04	1	1	2				1	1	2			
High	P62304	Small nuclear ribonucleoprotein E OS=Homo sapiens OX=9606 GN=SNRPE PE=1 SV=1	9	2	2	10.8	25	92	9.44	1	1	2	2	1		1	1	2	1	1	
High	Q9Y6E0	Serine/threonine-protein kinase 24 OS=Homo sapiens OX=9606 GN=STK24 PE=1 SV=1	9	4	4	49.3	9	443	5.69												
High	Q96AG4	Leucine-rich repeat-containing protein 59 OS=Homo sapiens OX=9606 GN=LRRC59 PE=1 SV=1	9	4	4	34.9	15	307	9.57			2	1	1	1			2	1	1	1
High	Q9BVJ6	U3 small nucleolar RNA-associated protein 14 homolog A OS=Homo sapiens OX=9606 GN=UTP14A PE=1 SV=1	9	3	3	87.9	6	771	7.87		1	1	1	3	1		1	1	1	3	1
High	Q9Y2D5	A-kinase anchor protein 2 OS=Homo sapiens OX=9606 GN=AKAP2 PE=1 SV=3	9	4	4	94.6	4	859	5.11	1	3	2		1		1	3	2		1	
High	Q8N9T8	Protein KRI1 homolog OS=Homo sapiens OX=9606 GN=KRI1 PE=1 SV=3	9	3	3	82.5	7	703	5.14	2						1					
High	Q8NEY8	Periphilin-1 OS=Homo sapiens OX=9606 GN=PPHLN1 PE=1 SV=2	9	2	2	52.7	4	458	9.11	1			1	2	1	1			1	2	1
High	Q9P2X0	Dolichol-phosphate mannosyltransferase subunit 3 OS=Homo sapiens OX=9606 GN=DPM3 PE=1 SV=2	9	2	2	10.1	24	92	5.94	1	1	1	1		1	1	1	1	1		1
High	Q15014	Mortality factor 4-like protein 2 OS=Homo sapiens OX=9606 GN=MORF4L2 PE=1 SV=1	9	3	2	32.3	11	288	9.72				3	1	1				3	1	1
High	O14561	Acyl carrier protein, mitochondrial OS=Homo sapiens OX=9606 GN=NDUFAB1 PE=1 SV=3	9	1	1	17.4	9	156	4.93	1	1	2				1	1	1			
High	O75146	Huntingtin-interacting protein 1-related protein OS=Homo sapiens OX=9606 GN=HIP1R PE=1 SV=2	9	4	3	119.3	4	1068	6.67		1	1					1	1			
High	P18510	Interleukin-1 receptor antagonist protein OS=Homo sapiens OX=9606 GN=IL1RN PE=1 SV=1	9	3	3	20	19	177	6.19				2	1	1				2	1	1
High	Q9Y3B2	Exosome complex component CSL4 OS=Homo sapiens OX=9606 GN=EXOSC1 PE=1 SV=1	9	3	3	21.4	17	195	8.24	1				2	1	1				2	1
High	Q9NVJ2	ADP-ribosylation factor-like protein 8B OS=Homo sapiens OX=9606 GN=ARL8B PE=1 SV=1	9	3	3	21.5	18	186	8.43	1	2	2				1	2	2			
High	Q96BR5	Cytochrome c oxidase assembly factor 7 OS=Homo sapiens OX=9606 GN=COA7 PE=1 SV=2	9	3	3	25.7	14	231	6.02			1	1	1	1			1	1	1	1
High	Q07866	Kinesin light chain 1 OS=Homo sapiens OX=9606 GN=KLC1 PE=1 SV=2	9	3	1	65.3	4	573	6.2	1	1	1	1	1		1	1	1	1	1	
High	P49711	Transcriptional repressor CTCF OS=Homo sapiens OX=9606 GN=CTCF PE=1 SV=1	9	4	4	82.7	8	727	6.96				1						1		
High	Q8TBB0	THAP domain-containing protein 6 OS=Homo sapiens OX=9606 GN=THAP6 PE=1 SV=1	9	3	3	25.7	20	222	8.7				1	1	1				1	1	1
High	Q10713	Mitochondrial-processing peptidase subunit alpha OS=Homo sapiens OX=9606 GN=PMPCA PE=1 SV=2	9	4	4	58.2	7	525	6.92	3	3	3				3	3	3			

High	Q9Y2R9	28S ribosomal protein S7, mitochondrial OS=Homo sapiens OX=9606 GN=MRPS7 PE=1 SV=2	9	5	5	28.1	26	242	9.99				1	1	1				1	1	1
High	Q7Z6K1	THAP domain-containing protein 5 OS=Homo sapiens OX=9606 GN=THAP5 PE=1 SV=2	9	1	1	45.4	2	395	6.71	1	1	1	1	1	1	1	1	1	1	1	1
High	Q16854	Deoxyguanosine kinase, mitochondrial OS=Homo sapiens OX=9606 GN=DGUOK PE=1 SV=2	9	3	3	32	15	277	8.66	1		2	1	1	2	1		2	1	1	2
High	Q14669	E3 ubiquitin-protein ligase TRIP12 OS=Homo sapiens OX=9606 GN=TRIP12 PE=1 SV=1	9	5	5	220.3	3	1992	8.48				1		1				1		1
High	P35268	60S ribosomal protein L22 OS=Homo sapiens OX=9606 GN=RPL22 PE=1 SV=2	9	1	1	14.8	9	128	9.19	1	1	1	1	1	1	1	1	1	1	1	1
High	Q8IVF2	Protein AHNAK2 OS=Homo sapiens OX=9606 GN=AHNAK2 PE=1 SV=2	9	2	2	616.2	3	5795	5.36	1	1	2		1	1	1	1	2		1	1
High	O00541	Pescadillo homolog OS=Homo sapiens OX=9606 GN=PES1 PE=1 SV=1	9	3	3	68	6	588	7.33	1	1	1	1		1	1	1	1	1		1
High	Q14493	Histone RNA hairpin-binding protein OS=Homo sapiens OX=9606 GN=SLBP PE=1 SV=1	9	3	3	31.3	14	270	7.47			1	1					1	1		
High	Q15154	Pericentriolar material 1 protein OS=Homo sapiens OX=9606 GN=PCM1 PE=1 SV=5	9	3	3	228.4	2	2024	5.02	2	1	1				2	1	1			
High	Q96RQ1	Endoplasmic reticulum-Golgi intermediate compartment protein 2 OS=Homo sapiens OX=9606 GN=ERGIC2 PE=1 SV=2	9	4	4	42.5	15	377	6.77	1	3	3			1	1	3	3			1
High	P51553	Isocitrate dehydrogenase [NAD] subunit gamma, mitochondrial OS=Homo sapiens OX=9606 GN=IDH3G PE=1 SV=1	9	2	2	42.8	4	393	8.5	2	2		1	1	1	2	2		1	1	1
High	Q9H8V3	Protein ECT2 OS=Homo sapiens OX=9606 GN=ECT2 PE=1 SV=4	9	4	4	103.4	7	914	7.71	1	1	1				1	1	1			
High	Q9Y6X8	Zinc fingers and homeoboxes protein 2 OS=Homo sapiens OX=9606 GN=ZHX2 PE=1 SV=1	9	4	3	92.3	5	837	6.86				2		1				2		1
High	Q01968	Inositol polyphosphate 5-phosphatase OCRL OS=Homo sapiens OX=9606 GN=OCRL PE=1 SV=3	9	2	2	104.1	2	901	6.55	1	1	1	1	1		1	1	1	1	1	
High	Q9BU76	Multiple myeloma tumor-associated protein 2 OS=Homo sapiens OX=9606 GN=MMTAG2 PE=1 SV=1	9	5	5	29.4	20	263	10.02					2	2					2	2
High	Q969S3	Zinc finger protein 622 OS=Homo sapiens OX=9606 GN=ZNF622 PE=1 SV=1	9	3	3	54.2	8	477	6.15	1	1	1	1			1	1	1	1		
High	Q14671	Pumilio homolog 1 OS=Homo sapiens OX=9606 GN=PUM1 PE=1 SV=3	9	5	5	126.4	6	1186	6.84		2	4					2	4			
High	P54259	Atrophin-1 OS=Homo sapiens OX=9606 GN=ATN1 PE=1 SV=3	9	2	2	125.3	3	1190	8.98	1	1	2	1	1		1	1	2	1	1	
High	Q9C0B0	RING finger protein unkempt homolog OS=Homo sapiens OX=9606 GN=UNK PE=1 SV=2	9	3	3	88	5	810	6.86	2	3	1				2	2	1			
High	O94855	Protein transport protein Sec24D OS=Homo sapiens OX=9606 GN=SEC24D PE=1 SV=2	9	5	4	112.9	6	1032	7.25		1		1		1		1		1		1
High	Q9H7E9	UPF0488 protein C8orf33 OS=Homo sapiens OX=9606 GN=C8orf33 PE=1 SV=1	9	3	3	25	19	229	9.95				1	2	1				1	2	1
High	Q6R327	Rapamycin-insensitive companion of mTOR OS=Homo sapiens OX=9606 GN=RICTOR PE=1 SV=1	9	4	4	192.1	4	1708	7.47	3	2	3				3	2	3			
High	P61019	Ras-related protein Rab-2A OS=Homo sapiens OX=9606 GN=RAB2A PE=1 SV=1	9	4	4	23.5	22	212	6.54	1		1	2		2	1		1	2		2
High	Q9NXV6	CDKN2A-interacting protein OS=Homo sapiens OX=9606 GN=CDKN2AIP PE=1 SV=3	9	3	3	61.1	6	580	9.01	1				1		1				1	
High	Q99081	Transcription factor 12 OS=Homo sapiens OX=9606 GN=TCF12 PE=1 SV=1	9	2	2	72.9	6	682	7.02	1	1	1	1	1	1	1	1	1	1	1	1
High	P42574	Caspase-3 OS=Homo sapiens OX=9606 GN=CASP3 PE=1 SV=2	9	2	1	31.6	7	277	6.54				1	1	2				1	1	2
High	Q6P1L8	39S ribosomal protein L14, mitochondrial OS=Homo sapiens OX=9606 GN=MRPL14 PE=1 SV=1	9	2	2	15.9	17	145	10.24	1	1	1		1		1	1	1		1	
High	Q8IXQ6	Protein mono-ADP-ribosyltransferase PARP9 OS=Homo sapiens OX=9606 GN=PARP9 PE=1 SV=2	9	2	2	96.3	2	854	7.91	1	1	1	2	1		1	1	1	2	1	
High	Q969N2	GPI transamidase component PIG-T OS=Homo sapiens OX=9606 GN=PIGT PE=1 SV=1	9	6	6	65.7	12	578	8.38	2	2	3				2	2	3			
High	P16930	Fumarylacetoacetase OS=Homo sapiens OX=9606 GN=FAH PE=1 SV=2	9	3	3	46.3	10	419	6.95	2	2			1		2	2			1	
High	Q9Y580	RNA-binding protein 7 OS=Homo sapiens OX=9606 GN=RBM7 PE=1 SV=1	9	2	2	30.5	6	266	9.57	1	1	1		1		1	1	1		1	
High	P08581	Hepatocyte growth factor receptor OS=Homo sapiens OX=9606 GN=MET PE=1 SV=4	9	2	1	155.4	2	1390	7.33	1	1	1		1	1	1	1	1		1	1
High	P07339	Cathepsin D OS=Homo sapiens OX=9606 GN=CTSD PE=1 SV=1	9	6	6	44.5	21	412	6.54			2	1		1			2	1		1
High	P48200	Iron-responsive element-binding protein 2 OS=Homo sapiens OX=9606 GN=IREB2 PE=1 SV=4	9	4	4	105	5	963	7.05	1	1					1	1				
High	P78344	Eukaryotic translation initiation factor 4 gamma 2 OS=Homo sapiens OX=9606 GN=EIF4G2 PE=1 SV=1	9	5	5	102.3	5	907	7.14	2	2	3				2	2	3			
High	O15379	Histone deacetylase 3 OS=Homo sapiens OX=9606 GN=HDAC3 PE=1 SV=2	9	3	2	48.8	9	428	5.16	1	1	2			1	1	1	2			1
High	P10586	Receptor-type tyrosine-protein phosphatase F OS=Homo sapiens OX=9606 GN=PTPRF PE=1 SV=2	9	4	4	212.7	3	1907	6.3	1	1	1		1		1	1	1		1	
High	Q96JJ7	Protein disulfide-isomerase TMX3 OS=Homo sapiens OX=9606 GN=TMX3 PE=1 SV=2	9	3	3	51.8	6	454	4.91			1		1				1		1	
High	Q6NZY4	Zinc finger CCHC domain-containing protein 8 OS=Homo sapiens OX=9606 GN=ZCCHC8 PE=1 SV=2	9	3	3	78.5	7	707	4.87	1	1	1		1	1	1	1	1		1	1
High	Q9BZE1	39S ribosomal protein L37, mitochondrial OS=Homo sapiens OX=9606 GN=MRPL37 PE=1 SV=2	9	4	4	48.1	11	423	8.59				1	3					1	3	
High	P29728	2'-5'-oligoadenylate synthase 2 OS=Homo sapiens OX=9606 GN=OAS2 PE=1 SV=3	8	2	2	82.4	4	719	8.25				1	2	1				1	2	1
High	Q9BWJ5	Splicing factor 3B subunit 5 OS=Homo sapiens OX=9606 GN=SF3B5 PE=1 SV=1	8	2	2	10.1	33	86	6.35	1	1			1	1	1	1			1	1
High	Q9P003	Protein cornichon homolog 4 OS=Homo sapiens OX=9606 GN=CNIH4 PE=1 SV=1	8	1	1	16.1	14	139	6.65	3	2	1				1	1	1			
High	P21741	Midkine OS=Homo sapiens OX=9606 GN=MDK PE=1 SV=1	8	3	3	15.6	26	143	9.79				1	1	1				1	1	1
High	P98194	Calcium-transporting ATPase type 2C member 1 OS=Homo sapiens OX=9606 GN=ATP2C1 PE=1 SV=3	8	4	4	100.5	5	919	6.74	1	2	2				1	2	2			
High	Q8TAA9	Vang-like protein 1 OS=Homo sapiens OX=9606 GN=VANGL1 PE=1 SV=1	8	3	3	59.9	8	524	8.81				1	2	2				1	2	2
High	O96013	Serine/threonine-protein kinase PAK 4 OS=Homo sapiens OX=9606 GN=PAK4 PE=1 SV=1	8	3	3	64	7	591	9.73	2	1	1				2	1	1			
High	P51668	Ubiquitin-conjugating enzyme E2 D1 OS=Homo sapiens OX=9606 GN=UBE2D1 PE=1 SV=1	8	2	1	16.6	20	147	7.42	2	2	1	1	1	1	2	2	1	1	1	1
High	Q9BRZ2	E3 ubiquitin-protein ligase TRIM56 OS=Homo sapiens OX=9606 GN=TRIM56 PE=1 SV=3	8	6	6	81.4	10	755	7.74			1		1				1		1	
High	Q92575	UBX domain-containing protein 4 OS=Homo sapiens OX=9606 GN=UBXN4 PE=1 SV=2	8	2	2	56.7	7	508	6.38	1		1				1		1			
High	Q1ED39	Lysine-rich nucleolar protein 1 OS=Homo sapiens OX=9606 GN=KNOP1 PE=1 SV=1	8	5	5	51.6	16	458	9.86					1						1	
High	Q9NW64	Pre-mRNA-splicing factor RBM22 OS=Homo sapiens OX=9606 GN=RBM22 PE=1 SV=1	8	2	2	46.9	5	420	8.54		1	1		1	1		1	1		1	1
High	Q9P265	Disco-interacting protein 2 homolog B OS=Homo sapiens OX=9606 GN=DIP2B PE=1 SV=3	8	4	4	171.4	3	1576	8.09	1	1	2				1	1	2			
High	Q9NY93	Probable ATP-dependent RNA helicase DDX56 OS=Homo sapiens OX=9606 GN=DDX56 PE=1 SV=1	8	5	5	61.6	12	547	9.26		1		1	1	2		1		1	1	2
High	Q13451	Peptidyl-prolyl cis-trans isomerase FKBP5 OS=Homo sapiens OX=9606 GN=FKBP5 PE=1 SV=2	8	4	4	51.2	11	457	5.9	1	1	1				1	1	1			
High	Q96CB8	Integrator complex subunit 12 OS=Homo sapiens OX=9606 GN=INTS12 PE=1 SV=1	8	2	2	48.8	7	462	9.69	2	2	1				2	2	1			
High	Q8TEA1	tRNA (cytosine(72)-C(5))-methyltransferase NSUN6 OS=Homo sapiens OX=9606 GN=NSUN6 PE=1 SV=1	8	1	1	51.7	4	469	7.96	1	1	1				1	1	1			
High	Q9UHR5	SAP30-binding protein OS=Homo sapiens OX=9606 GN=SAP30BP PE=1 SV=1	8	1	1	33.9	3	308	4.84	1	1		1	1	1	1	1		1	1	1
High	P28290	Protein ITPRID2 OS=Homo sapiens OX=9606 GN=ITPRID2 PE=1 SV=3	8	4	4	138.3	4	1259	5.19												
High	Q92879	CUGBP Elav-like family member 1 OS=Homo sapiens OX=9606 GN=CELF1 PE=1 SV=2	8	3	3	52	6	486	8.46		1	1					1	1			
High	P49591	Serine--tRNA ligase, cytoplasmic OS=Homo sapiens OX=9606 GN=SARS1 PE=1 SV=3	8	2	2	58.7	9	514	6.43	2	1	2				2	1	2			
High	O15230	Laminin subunit alpha-5 OS=Homo sapiens OX=9606 GN=LAMA5 PE=1 SV=8	8	2	2	399.5	1	3695	7.02			1	1	2	1			1	1	2	1
High	Q96CW5	Gamma-tubulin complex component 3 OS=Homo sapiens OX=9606 GN=TUBGCP3 PE=1 SV=2	8	4	4	103.5	8	907	8.12	1	2	2				1	2	2			
High	Q6PJG2	Mitotic deacetylase-associated SANT domain protein OS=Homo sapiens OX=9606 GN=MIDEAS PE=1 SV=2	8	5	5	114.9	9	1045	9.19												
High	Q9NRN7	L-aminoadipate-semialdehyde dehydrogenase-phosphopantetheinyl transferase OS=Homo sapiens OX=9606 GN=AASDHPPT PE=1	8	2	2	35.8	10	309	6.8	2		1				2		1			
High	Q5VV67	Peroxisome proliferator-activated receptor gamma coactivator-related protein 1 OS=Homo sapiens OX=9606 GN=PPRC1 PE=1 SV=	8	5	5	177.4	5	1664	6.51				3	4	1				3	4	1
High	P36639	Oxidized purine nucleoside triphosphate hydrolase OS=Homo sapiens OX=9606 GN=NUDT1 PE=1 SV=4	8	2	2	17.9	16	156	5.08	1	1	2			1	1	1	2			1
High	Q9NPD3	Exosome complex component RRP41 OS=Homo sapiens OX=9606 GN=EXOSC4 PE=1 SV=3	8	2	2	26.4	9	245	6.52	1			1	1	1	1			1	1	1
High	Q96GM5	SWI/SNF-related matrix-associated actin-dependent regulator of chromatin subfamily D member 1 OS=Homo sapiens OX=9606 GI	8	3	2	58.2	8	515	9.25			1	1					1	1		
High	Q9P2I0	Cleavage and polyadenylation specificity factor subunit 2 OS=Homo sapiens OX=9606 GN=CPSF2 PE=1 SV=2	8	4	4	88.4	5	782	5.11	1	1	1				1	1	1			
High	Q6IA86	Elongator complex protein 2 OS=Homo sapiens OX=9606 GN=ELP2 PE=1 SV=2	8	4	4	92.4	6	826	5.96	1	1	1				1	1	1			
High	Q9BRT6	Protein LLP homolog OS=Homo sapiens OX=9606 GN=LLPH PE=1 SV=1	8	2	2	15.2	19	129	10.37			2	1	1	1			2	1	1	1
High	Q13541	Eukaryotic translation initiation factor 4E-binding protein 1 OS=Homo sapiens OX=9606 GN=EIF4EBP1 PE=1 SV=3	8	3	3	12.6	59	118	5.48			2						2			
High	Q15796	Mothers against decapentaplegic homolog 2 OS=Homo sapiens OX=9606 GN=SMAD2 PE=1 SV=1	8	2	1	52.3	5	467	6.58				1	2	1				1	2	1
High	Q9NP66	High mobility group protein 20A OS=Homo sapiens OX=9606 GN=HMG20A PE=1 SV=1	8	4	4	40.1	24	347	6.49	1	2	5				1	2	4			
High	Q00587	Cdc42 effector protein 1 OS=Homo sapiens OX=9606 GN=CDC42EP1 PE=1 SV=1	8	3	3	40.3	8	391	7.15	1	1	1	1		1	1	1	1	1		1
High	Q8N3E9	1-phosphatidylinositol 4,5-bisphosphate phosphodiesterase delta-3 OS=Homo sapiens OX=9606 GN=PLCD3 PE=1 SV=3	8	3	3	89.2	7	789	6.98		1	2					1	2			
High	Q96SK2	Transmembrane protein 209 OS=Homo sapiens OX=9606 GN=TMEM209 PE=1 SV=2	8	5	5	62.9	14	561	8.63			3						3			
High	Q12774	Rho guanine nucleotide exchange factor 5 OS=Homo sapiens OX=9606 GN=ARHGEF5 PE=1 SV=3	8	2	2	176.7	2	1597	5.53			1	1	1	2			1	1	1	2
High	Q9H8W4	Pleckstrin homology domain-containing family F member 2 OS=Homo sapiens OX=9606 GN=PLEKHF2 PE=1 SV=1	8	2	2	27.8	12	249	8.21		1		1	1	1		1		1	1	1
High	P19256	Lymphocyte function-associated antigen 3 OS=Homo sapiens OX=9606 GN=CD58 PE=1 SV=1	8	2	2	28.1	7	250	6.76	1	1	1		1		1	1	1		1	
High	P62330	ADP-ribosylation factor 6 OS=Homo sapiens OX=9606 GN=ARF6 PE=1 SV=2	8	2	2	20.1	15	175	8.95	2	1	2				2	1	2			
High	Q9ULU4	Protein kinase C-binding protein 1 OS=Homo sapiens OX=9606 GN=ZMYND8 PE=1 SV=2	8	4	4	131.6	5	1186	7.2	1		1	1		1	1		1	1		1
High	P55263	Adenosine kinase OS=Homo sapiens OX=9606 GN=ADK PE=1 SV=2	8	2	2	40.5	8	362	6.7		1	1		1	1		1	1		1	1
High	P20674	Cytochrome c oxidase subunit 5A, mitochondrial OS=Homo sapiens OX=9606 GN=COX5A PE=1 SV=2	8	3	3	16.8	21	150	6.79	2	2	1		1		2	2	1		1	
High	O75363	Breast carcinoma-amplified sequence 1 OS=Homo sapiens OX=9606 GN=BCAS1 PE=1 SV=2	8	2	2	61.7	5	584	5.1	1	1	1				1	1	1			
High	O75506	Heat shock factor-binding protein 1 OS=Homo sapiens OX=9606 GN=HSBP1 PE=1 SV=1	8	2	2	8.5	58	76	4.36	2	1	3				2	1	2			
High	Q9NZQ3	NCK-interacting protein with SH3 domain OS=Homo sapiens OX=9606 GN=NCKIPSD PE=1 SV=1	8	4	4	78.9	7	722	6.38	3	3	2				3	3	2			
High	Q5VYY1	Ankyrin repeat domain-containing protein 22 OS=Homo sapiens OX=9606 GN=ANKRD22 PE=2 SV=1	8	2	2	21.8	9	191	8.84	2	2					2	2				
High	P06702	Protein S100-A9 OS=Homo sapiens OX=9606 GN=S100A9 PE=1 SV=1	8	3	3	13.2	31	114	6.13				2	3	2				2	3	2
High	Q13887	Krueppel-like factor 5 OS=Homo sapiens OX=9606 GN=KLF5 PE=1 SV=2	8	3	3	50.8	15	457	8.6	1	1	1				1	1	1			
High	Q92896	Golgi apparatus protein 1 OS=Homo sapiens OX=9606 GN=GLG1 PE=1 SV=2	8	4	4	134.5	3	1179	6.9					2						2	
High	Q12857	Nuclear factor 1 A-type OS=Homo sapiens OX=9606 GN=NFIA PE=1 SV=2	8	2	1	55.9	4	509	8.44	1		1	1	1	1	1		1	1	1	1
High	Q9UI10	Translation initiation factor eIF-2B subunit delta OS=Homo sapiens OX=9606 GN=EIF2B4 PE=1 SV=2	8	3	3	57.5	12	523	9.38	1	2	2				1	2	2			
High	Q9H3M7	Thioredoxin-interacting protein OS=Homo sapiens OX=9606 GN=TXNIP PE=1 SV=1	8	5	5	43.6	22	391	7.5		2	3					2	2			
High	Q9BRD0	BUD13 homolog OS=Homo sapiens OX=9606 GN=BUD13 PE=1 SV=1	8	2	2	70.5	3	619	9.86	1	1			1	1	1	1			1	1
High	Q9NZL4	Hsp70-binding protein 1 OS=Homo sapiens OX=9606 GN=HSPBP1 PE=1 SV=2	8	2	2	39.3	9	359	5.21				1		1				1		1
High	Q9NV88	Integrator complex subunit 9 OS=Homo sapiens OX=9606 GN=INTS9 PE=1 SV=2	8	5	5	73.8	12	658	6.33	3	2	3				3	2	3			
High	P04258	SWISS-PROT:P04258 (Bos taurus) Similar to Collagen alpha 1(III) chain	8	3	3	138.4	3	1466	6.43				3	2	3				2	2	2
High	Q6IA69	Glutamine-dependent NAD(+) synthetase OS=Homo sapiens OX=9606 GN=NADSYN1 PE=1 SV=3	8	2	2	79.2	4	706	6.44	1	1	1	1	2	1	1	1	1	1	2	1
High	Q9BTE6	Alanyl-tRNA editing protein Aarsd1 OS=Homo sapiens OX=9606 GN=AARSD1 PE=1 SV=2	8	2	2	45.5	8	412	6.42	1	1		1		1	1	1		1		1
High	Q5VTU8	ATP synthase subunit epsilon-like protein, mitochondrial OS=Homo sapiens OX=9606 GN=ATP5F1EP2 PE=1 SV=1	8	1	1	5.8	16	51	10.14	1	1		1	1	1	1	1		1	1	1
High	P13073	Cytochrome c oxidase subunit 4 isoform 1, mitochondrial OS=Homo sapiens OX=9606 GN=COX4I1 PE=1 SV=1	8	3	3	19.6	19	169	9.51	1	1	1				1	1	1			
High	Q9P0J0	NADH dehydrogenase [ubiquinone] 1 alpha subcomplex subunit 13 OS=Homo sapiens OX=9606 GN=NDUFA13 PE=1 SV=3	8	3	3	16.7	21	144	8.43	2	3	2				2	3	2			
High	P32320	Cytidine deaminase OS=Homo sapiens OX=9606 GN=CDA PE=1 SV=2	8	4	4	16.2	55	146	6.92				1						1		
High	P14635	G2/mitotic-specific cyclin-B1 OS=Homo sapiens OX=9606 GN=CCNB1 PE=1 SV=1	8	3	3	48.3	8	433	7.47				2	2	2				2	2	2
High	O75569	Interferon-inducible double-stranded RNA-dependent protein kinase activator A OS=Homo sapiens OX=9606 GN=PRKRA PE=1 SV=	8	3	3	34.4	13	313	8.41			1		1				1		1	
High	Q5JTH9	RRP12-like protein OS=Homo sapiens OX=9606 GN=RRP12 PE=1 SV=2	8	4	4	143.6	4	1297	8.75			1	1		1			1	1		1
High	P20936	Ras GTPase-activating protein 1 OS=Homo sapiens OX=9606 GN=RASA1 PE=1 SV=1	8	3	3	116.3	3	1047	6.54			1	1	1				1	1	1	
High	Q96EY8	Corrinoid adenosyltransferase OS=Homo sapiens OX=9606 GN=MMAB PE=1 SV=1	8	3	3	27.4	18	250	8.6	1	1	1		1		1	1	1		1	
High	O43291	Kunitz-type protease inhibitor 2 OS=Homo sapiens OX=9606 GN=SPINT2 PE=1 SV=2	8	3	3	28.2	13	252	8.29		1	1					1	1			
High	O75419	Cell division control protein 45 homolog OS=Homo sapiens OX=9606 GN=CDC45 PE=1 SV=1	8	2	2	65.5	5	566	5.53	1	2	1		1		1	2	1		1	
High	Q9UDW1	Cytochrome b-c1 complex subunit 9 OS=Homo sapiens OX=9606 GN=UQCR10 PE=1 SV=3	8	2	2	7.3	38	63	9.47	2	2	2		1		2	2	2		1	
High	P54577	Tyrosine--tRNA ligase, cytoplasmic OS=Homo sapiens OX=9606 GN=YARS1 PE=1 SV=4	8	5	5	59.1	9	528	7.05	2	1	3				2	1	3			
High	Q96MF7	E3 SUMO-protein ligase NSE2 OS=Homo sapiens OX=9606 GN=NSMCE2 PE=1 SV=2	8	3	3	27.9	15	247	7.74					1						1	
High	Q6P2E9	Enhancer of mRNA-decapping protein 4 OS=Homo sapiens OX=9606 GN=EDC4 PE=1 SV=1	8	3	3	151.6	2	1401	5.86			2	1		1			2	1		1
High	O94915	Protein furry homolog-like OS=Homo sapiens OX=9606 GN=FRYL PE=1 SV=2	8	2	2	339.4	1	3013	5.58	1				1		1				1	
High	Q96KP4	Cytosolic non-specific dipeptidase OS=Homo sapiens OX=9606 GN=CNDP2 PE=1 SV=2	8	3	3	52.8	8	475	5.97	1						1					
High	Q9NVN8	Guanine nucleotide-binding protein-like 3-like protein OS=Homo sapiens OX=9606 GN=GNL3L PE=1 SV=1	8	2	1	65.5	5	582	8.44		1	1		1			1	1		1	
High	Q9UNL2	Translocon-associated protein subunit gamma OS=Homo sapiens OX=9606 GN=SSR3 PE=1 SV=1	8	1	1	21.1	8	185	9.61	1	1	1		2		1	1	1		1	
High	Q9BTC8	Metastasis-associated protein MTA3 OS=Homo sapiens OX=9606 GN=MTA3 PE=1 SV=2	8	4	3	67.5	7	594	8.57				1		1				1		1
High	P50453	Serpin B9 OS=Homo sapiens OX=9606 GN=SERPINB9 PE=1 SV=1	8	4	3	42.4	13	376	5.86	1	1	2		1	2	1	1	2		1	2
High	Q9UKK6	NTF2-related export protein 1 OS=Homo sapiens OX=9606 GN=NXT1 PE=1 SV=1	8	3	3	15.8	25	140	5.03					1						1	
High	Q68CR1	Protein sel-1 homolog 3 OS=Homo sapiens OX=9606 GN=SEL1L3 PE=1 SV=2	8	3	3	128.5	5	1132	7.28	1	2	2		1		1	2	2		1	
High	Q9BY89	Uncharacterized protein KIAA1671 OS=Homo sapiens OX=9606 GN=KIAA1671 PE=1 SV=2	8	2	2	196.6	2	1806	8.47	2		2				2		2			
High	Q6KC79	Nipped-B-like protein OS=Homo sapiens OX=9606 GN=NIPBL PE=1 SV=2	8	5	5	315.9	2	2804	7.91	2	1	1				2	1	1			
High	Q8NFH4	Nucleoporin Nup37 OS=Homo sapiens OX=9606 GN=NUP37 PE=1 SV=1	8	3	3	36.7	11	326	5.92		1						1				
High	P30876	DNA-directed RNA polymerase II subunit RPB2 OS=Homo sapiens OX=9606 GN=POLR2B PE=1 SV=1	8	3	3	133.8	4	1174	6.87			1		1	1			1		1	1
High	Q9NVX7	Kelch repeat and BTB domain-containing protein 4 OS=Homo sapiens OX=9606 GN=KBTBD4 PE=1 SV=3	8	3	3	58.1	5	518	5.67	1	2	1				1	2	1			
High	Q9NYU2	UDP-glucose:glycoprotein glucosyltransferase 1 OS=Homo sapiens OX=9606 GN=UGGT1 PE=1 SV=3	8	4	4	177.1	3	1555	5.63			1		1				1		1	
High	Q15003	Condensin complex subunit 2 OS=Homo sapiens OX=9606 GN=NCAPH PE=1 SV=3	8	3	3	82.5	5	741	5.06				1	2	2				1	2	2
High	O00442	RNA 3'-terminal phosphate cyclase OS=Homo sapiens OX=9606 GN=RTCA PE=1 SV=1	8	2	2	39.3	5	366	7.85				1	1	1				1	1	1
High	Q7L1Q6	eIF5-mimic protein 2 OS=Homo sapiens OX=9606 GN=BZW1 PE=1 SV=1	8	2	1	48	4	419	5.92	2	2	2			2	2	2	2			2
High	Q9UD71	Protein phosphatase 1 regulatory subunit 1B OS=Homo sapiens OX=9606 GN=PPP1R1B PE=1 SV=2	8	2	2	22.9	23	204	4.53	1	1	1				1	1	1			
High	Q9BST9	Rhotekin OS=Homo sapiens OX=9606 GN=RTKN PE=1 SV=2	8	2	2	62.6	4	563	7.44	2	1	1	1		1	2	1	1	1		1
High	P14324	Farnesyl pyrophosphate synthase OS=Homo sapiens OX=9606 GN=FDPS PE=1 SV=4	8	3	3	48.2	9	419	6.15			1	1					1	1		
High	Q7Z5L9	Interferon regulatory factor 2-binding protein 2 OS=Homo sapiens OX=9606 GN=IRF2BP2 PE=1 SV=2	8	3	3	61	8	587	8.69		1	2		1			1	2		1	
High	P30049	ATP synthase subunit delta, mitochondrial OS=Homo sapiens OX=9606 GN=ATP5F1D PE=1 SV=2	8	1	1	17.5	5	168	5.49		1	1	1	1	1		1	1	1	1	1
High	Q9UBR2	Cathepsin Z OS=Homo sapiens OX=9606 GN=CTSZ PE=1 SV=1	8	3	3	33.8	13	303	7.11	1	1			1	1	1	1			1	1
High	Q9BQ75	Protein CMSS1 OS=Homo sapiens OX=9606 GN=CMSS1 PE=1 SV=2	8	6	6	31.9	21	279	9.19												
High	P50213	Isocitrate dehydrogenase [NAD] subunit alpha, mitochondrial OS=Homo sapiens OX=9606 GN=IDH3A PE=1 SV=1	8	3	3	39.6	13	366	6.92	1	1	3	1		2	1	1	3	1		2
High	Q15738	Sterol-4-alpha-carboxylate 3-dehydrogenase, decarboxylating OS=Homo sapiens OX=9606 GN=NSDHL PE=1 SV=2	8	5	5	41.9	19	373	8.06	3	3	1				3	3	1			
High	P42226	Signal transducer and activator of transcription 6 OS=Homo sapiens OX=9606 GN=STAT6 PE=1 SV=1	8	2	2	94.1	3	847	6.23	1	1	1			1	1	1	1			1
High	P60983	Glia maturation factor beta OS=Homo sapiens OX=9606 GN=GMFB PE=1 SV=2	8	2	2	16.7	11	142	5.29			1	2	2	2			1	2	2	2
High	Q9Y5Q9	General transcription factor 3C polypeptide 3 OS=Homo sapiens OX=9606 GN=GTF3C3 PE=1 SV=1	8	4	4	101.2	8	886	5.07						1						1
High	Q9NSK0	Kinesin light chain 4 OS=Homo sapiens OX=9606 GN=KLC4 PE=1 SV=3	7	3	1	68.6	4	619	6.18	1	1	1	1	1		1	1	1	1	1	
High	Q9UID3	Vacuolar protein sorting-associated protein 51 homolog OS=Homo sapiens OX=9606 GN=VPS51 PE=1 SV=2	7	2	2	86	3	782	6.47	1	1	2				1	1	2			
High	P35270	Sepiapterin reductase OS=Homo sapiens OX=9606 GN=SPR PE=1 SV=1	7	4	4	28	21	261	8.05		1		1		1		1		1		1
High	O15260	Surfeit locus protein 4 OS=Homo sapiens OX=9606 GN=SURF4 PE=1 SV=3	7	2	2	30.4	9	269	7.78	1		2		1		1		1		1	
High	O75348	V-type proton ATPase subunit G 1 OS=Homo sapiens OX=9606 GN=ATP6V1G1 PE=1 SV=3	7	2	2	13.7	21	118	8.79	1		1		2		1		1		1	
High	Q9UBP9	PTB domain-containing engulfment adapter protein 1 OS=Homo sapiens OX=9606 GN=GULP1 PE=1 SV=1	7	5	5	34.5	17	304	7.9	1	3	1				1	3	1			
High	P18206	Vinculin OS=Homo sapiens OX=9606 GN=VCL PE=1 SV=4	7	2	2	123.7	3	1134	5.66		1	1		2	2		1	1		2	2
High	Q8N465	D-2-hydroxyglutarate dehydrogenase, mitochondrial OS=Homo sapiens OX=9606 GN=D2HGDH PE=1 SV=3	7	3	3	56.4	8	521	7.99												
High	Q8NFC6	Biorientation of chromosomes in cell division protein 1-like 1 OS=Homo sapiens OX=9606 GN=BOD1L1 PE=1 SV=2	7	2	2	330.3	1	3051	5.08				1	2	1				1	2	1
High	Q92576	PHD finger protein 3 OS=Homo sapiens OX=9606 GN=PHF3 PE=1 SV=3	7	2	1	229.3	1	2039	6.96				1	1	1				1	1	1
High	Q9Y2H1	Serine/threonine-protein kinase 38-like OS=Homo sapiens OX=9606 GN=STK38L PE=1 SV=3	7	3	1	54	8	464	6.81		1	1	1				1	1	1		
High	P25815	Protein S100-P OS=Homo sapiens OX=9606 GN=S100P PE=1 SV=2	7	1	1	10.4	14	95	4.88			1	1	1	1			1	1	1	1
High	Q9P107	GEM-interacting protein OS=Homo sapiens OX=9606 GN=GMIP PE=1 SV=2	7	4	4	106.6	6	970	5.72												
High	Q93034	Cullin-5 OS=Homo sapiens OX=9606 GN=CUL5 PE=1 SV=4	7	3	3	90.9	4	780	7.94	1			1	1	1	1			1	1	1
High	Q9NQX3	Gephyrin OS=Homo sapiens OX=9606 GN=GPHN PE=1 SV=1	7	3	3	79.7	4	736	5.43						1						1
High	Q12824	SWI/SNF-related matrix-associated actin-dependent regulator of chromatin subfamily B member 1 OS=Homo sapiens OX=9606 GI	7	4	4	44.1	18	385	6.23					1						1	
High	Q9NXA8	NAD-dependent protein deacylase sirtuin-5, mitochondrial OS=Homo sapiens OX=9606 GN=SIRT5 PE=1 SV=2	7	2	2	33.9	9	310	8.47	1	1	1	1			1	1	1	1		
High	O43399	Tumor protein D54 OS=Homo sapiens OX=9606 GN=TPD52L2 PE=1 SV=2	7	1	1	22.2	4	206	5.36	1	1	1	1	1		1	1	1	1	1	
High	Q92947	Glutaryl-CoA dehydrogenase, mitochondrial OS=Homo sapiens OX=9606 GN=GCDH PE=1 SV=1	7	3	3	48.1	13	438	8.06		2	3					2	3			
High	Q9BQ70	Transcription factor 25 OS=Homo sapiens OX=9606 GN=TCF25 PE=1 SV=1	7	2	2	76.6	4	676	6.35						1						1
High	Q9Y6I3	Epsin-1 OS=Homo sapiens OX=9606 GN=EPN1 PE=1 SV=2	7	5	5	60.3	17	576	4.83												
High	Q9BQ69	ADP-ribose glycohydrolase MACROD1 OS=Homo sapiens OX=9606 GN=MACROD1 PE=1 SV=2	7	2	2	35.5	10	325	9.51			1		1				1		1	
High	P57088	Transmembrane protein 33 OS=Homo sapiens OX=9606 GN=TMEM33 PE=1 SV=2	7	2	2	28	9	247	9.7	2	2	1		1		2	2	1		1	
High	Q8TBC4	NEDD8-activating enzyme E1 catalytic subunit OS=Homo sapiens OX=9606 GN=UBA3 PE=1 SV=2	7	4	4	51.8	14	463	5.45		1	1	1		2		1	1	1		2

High	Q9H857	5'-nucleotidase domain-containing protein 2 OS=Homo sapiens OX=9606 GN=NT5DC2 PE=1 SV=1	7	4	4	60.7	8	520	6.77	1	1	1				1	1	1			
High	Q9Y6M9	NADH dehydrogenase [ubiquinone] 1 beta subcomplex subunit 9 OS=Homo sapiens OX=9606 GN=NDUFB9 PE=1 SV=3	7	2	2	21.8	18	179	8.38	2	2	2				2	2	2			
High	Q9Y4F5	Centrosomal protein of 170 kDa protein B OS=Homo sapiens OX=9606 GN=CEP170B PE=1 SV=4	7	3	3	171.6	3	1589	6.84												
High	Q14145	Kelch-like ECH-associated protein 1 OS=Homo sapiens OX=9606 GN=KEAP1 PE=1 SV=2	7	3	3	69.6	5	624	6.44				2	3	2				2	3	2
High	P49419	Alpha-aminoadipic semialdehyde dehydrogenase OS=Homo sapiens OX=9606 GN=ALDH7A1 PE=1 SV=5	7	2	2	58.5	7	539	7.99		1			1	1		1			1	1
High	Q9H788	SH2 domain-containing protein 4A OS=Homo sapiens OX=9606 GN=SH2D4A PE=1 SV=1	7	1	1	52.7	3	454	8.06			1	1	1	1			1	1	1	1
High	Q96D46	60S ribosomal export protein NMD3 OS=Homo sapiens OX=9606 GN=NMD3 PE=1 SV=1	7	3	3	57.6	8	503	7.14	1			1	1	1	1			1	1	1
High	Q9NV56	MRG/MORF4L-binding protein OS=Homo sapiens OX=9606 GN=MRGBP PE=1 SV=1	7	1	1	22.4	7	204	5.83		1	1	1		1		1	1	1		1
High	Q8N1G0	Zinc finger protein 687 OS=Homo sapiens OX=9606 GN=ZNF687 PE=1 SV=1	7	3	3	129.4	5	1237	8.19				1		1				1		1
High	Q9UKU7	Isobutyryl-CoA dehydrogenase, mitochondrial OS=Homo sapiens OX=9606 GN=ACAD8 PE=1 SV=1	7	2	2	45	4	415	7.85	1	1	1				1	1	1			
High	O43414	ERI1 exoribonuclease 3 OS=Homo sapiens OX=9606 GN=ERI3 PE=1 SV=2	7	4	4	37.2	14	337	8.07	1	1		1	2		1	1		1	1	
High	P62308	Small nuclear ribonucleoprotein G OS=Homo sapiens OX=9606 GN=SNRPG PE=1 SV=1	7	2	2	8.5	25	76	8.88		2	1	1	1			2	1	1	1	
High	P26440	Isovaleryl-CoA dehydrogenase, mitochondrial OS=Homo sapiens OX=9606 GN=IVD PE=1 SV=2	7	1	1	46.6	2	426	8.05	1	1	1		1		1	1	1		1	
High	P11234	Ras-related protein Ral-B OS=Homo sapiens OX=9606 GN=RALB PE=1 SV=1	7	3	3	23.4	16	206	6.62		1			1			1			1	
High	P62841	40S ribosomal protein S15 OS=Homo sapiens OX=9606 GN=RPS15 PE=1 SV=2	7	1	1	17	9	145	10.39	1	1	1		1		1	1	1		1	
High	O75190	DnaJ homolog subfamily B member 6 OS=Homo sapiens OX=9606 GN=DNAJB6 PE=1 SV=2	7	4	3	36.1	17	326	9.16	2	1	2				2	1	2			
High	P47736	Rap1 GTPase-activating protein 1 OS=Homo sapiens OX=9606 GN=RAP1GAP PE=1 SV=2	7	3	3	73.3	6	663	5.82	2	2	1				2	2	1			
High	Q9Y3B8	Oligoribonuclease, mitochondrial OS=Homo sapiens OX=9606 GN=REXO2 PE=1 SV=3	7	1	1	26.8	3	237	6.87	1	1		1	1	1	1	1		1	1	1
High	Q9Y5J1	U3 small nucleolar RNA-associated protein 18 homolog OS=Homo sapiens OX=9606 GN=UTP18 PE=1 SV=3	7	2	2	62	6	556	8.76	1	1	1				1	1	1			
High	Q06587	E3 ubiquitin-protein ligase RING1 OS=Homo sapiens OX=9606 GN=RING1 PE=1 SV=2	7	3	2	42.4	11	406	5.62	1		1		1	2	1		1		1	2
High	O00507	Probable ubiquitin carboxyl-terminal hydrolase FAF-Y OS=Homo sapiens OX=9606 GN=USP9Y PE=2 SV=2	7	4	1	290.9	2	2555	5.86	1		1				1		1			
High	Q02413	Desmoglein-1 OS=Homo sapiens OX=9606 GN=DSG1 PE=1 SV=2	7	6	6	113.7	8	1049	5.03	1		5		1		1		5		1	
High	Q92973	Transportin-1 OS=Homo sapiens OX=9606 GN=TNPO1 PE=1 SV=2	7	2	2	102.3	2	898	4.98			1						1			
High	P57678	Gem-associated protein 4 OS=Homo sapiens OX=9606 GN=GEMIN4 PE=1 SV=2	7	4	4	120	5	1058	6.04	2	1					2	1				
High	Q5T653	39S ribosomal protein L2, mitochondrial OS=Homo sapiens OX=9606 GN=MRPL2 PE=1 SV=2	7	3	3	33.3	14	305	11.3				1	1	1				1	1	1
High	Q5T6V5	Queuosine salvage protein OS=Homo sapiens OX=9606 GN=C9orf64 PE=1 SV=1	7	1	1	39	2	341	5.88	1	1	1		1		1	1	1		1	
High	P02666	SWISS-PROT:P02666 Beta-casein - Bos taurus (Bovine).	7	2	2	23.6	12	209	5.19		1				1		1				1
High	Q9BRP8	Partner of Y14 and mago OS=Homo sapiens OX=9606 GN=PYM1 PE=1 SV=1	7	2	2	22.6	14	204	9.45			1						1			
High	Q92817	Envoplakin OS=Homo sapiens OX=9606 GN=EVPL PE=1 SV=3	7	4	4	231.5	3	2033	6.96												
High	P05114	Non-histone chromosomal protein HMG-14 OS=Homo sapiens OX=9606 GN=HMGN1 PE=1 SV=3	7	2	2	10.7	16	100	9.6	1	2		1	1	2	1	2		1	1	2
High	P25311	Zinc-alpha-2-glycoprotein OS=Homo sapiens OX=9606 GN=AZGP1 PE=1 SV=2	7	3	3	34.2	12	298	6.05				3	2	2				3	2	2
High	Q9BYD2	39S ribosomal protein L9, mitochondrial OS=Homo sapiens OX=9606 GN=MRPL9 PE=1 SV=2	7	2	2	30.2	7	267	10.08				1	1	1				1	1	1
High	P20810	Calpastatin OS=Homo sapiens OX=9606 GN=CAST PE=1 SV=4	7	3	3	76.5	9	708	5.07	1	1	1		1		1	1	1		1	
High	Q13188	Serine/threonine-protein kinase 3 OS=Homo sapiens OX=9606 GN=STK3 PE=1 SV=2	7	4	3	56.3	12	491	5.24	2	1	2		1		2	1	2		1	
High	P55064	Aquaporin-5 OS=Homo sapiens OX=9606 GN=AQP5 PE=1 SV=1	7	2	2	28.3	10	265	8.62	1	1	1				1	1	1			
High	Q9NVV4	Poly(A) RNA polymerase, mitochondrial OS=Homo sapiens OX=9606 GN=MTPAP PE=1 SV=1	7	2	2	66.1	4	582	9.04	1	1	1		2		1	1	1		2	
High	Q9BXB5	Oxysterol-binding protein-related protein 10 OS=Homo sapiens OX=9606 GN=OSBPL10 PE=1 SV=2	7	1	1	83.9	2	764	8.31		1	1	1	1			1	1	1	1	
High	Q8TB61	Adenosine 3'-phospho 5'-phosphosulfate transporter 1 OS=Homo sapiens OX=9606 GN=SLC35B2 PE=1 SV=1	7	3	3	47.5	7	432	9.16	1	1	2			1	1	1	2			1
High	O00273	DNA fragmentation factor subunit alpha OS=Homo sapiens OX=9606 GN=DFFA PE=1 SV=1	7	2	2	36.5	9	331	4.79	1	1	1				1	1	1			
High	Q9Y5V3	Melanoma-associated antigen D1 OS=Homo sapiens OX=9606 GN=MAGED1 PE=1 SV=3	7	3	3	86.1	5	778	5.83	1	3	2	1			1	3	2	1		
High	Q9HC52	Chromobox protein homolog 8 OS=Homo sapiens OX=9606 GN=CBX8 PE=1 SV=3	7	2	2	43.4	8	389	9.91				1	2					1	2	
High	Q15691	Microtubule-associated protein RP/EB family member 1 OS=Homo sapiens OX=9606 GN=MAPRE1 PE=1 SV=3	7	2	2	30	9	268	5.14	2	1		1	1	1	2	1		1	1	1
High	Q9BRJ7	Tudor-interacting repair regulator protein OS=Homo sapiens OX=9606 GN=NUDT16L1 PE=1 SV=1	7	2	2	23.3	13	211	8.91	1	1	1			1	1	1	1			1
High	P62877	E3 ubiquitin-protein ligase RBX1 OS=Homo sapiens OX=9606 GN=RBX1 PE=1 SV=1	7	1	1	12.3	7	108	6.96	1			1	1	1	1			1	1	1
High	Q96LD4	E3 ubiquitin-protein ligase TRIM47 OS=Homo sapiens OX=9606 GN=TRIM47 PE=1 SV=2	7	4	4	69.5	8	638	6.44												
High	Q68CP9	AT-rich interactive domain-containing protein 2 OS=Homo sapiens OX=9606 GN=ARID2 PE=1 SV=2	7	2	2	197.3	2	1835	7.42	1		1				1		1			
High	P54619	5'-AMP-activated protein kinase subunit gamma-1 OS=Homo sapiens OX=9606 GN=PRKAG1 PE=1 SV=1	7	2	2	37.6	6	331	6.92			1	1	1	1			1	1	1	1
High	Q9HAS0	Protein Njmu-R1 OS=Homo sapiens OX=9606 GN=C17orf75 PE=1 SV=2	7	1	1	44.6	3	396	5.03			1	1	1	1			1	1	1	1
High	Q07507	Dermatopontin OS=Homo sapiens OX=9606 GN=DPT PE=1 SV=2	7	3	3	24	13	201	4.82				3	2	2				3	2	2
High	Q69YN2	CWF19-like protein 1 OS=Homo sapiens OX=9606 GN=CWF19L1 PE=1 SV=2	7	2	2	60.6	4	538	7.24	1			1			1			1		
High	O00743	Serine/threonine-protein phosphatase 6 catalytic subunit OS=Homo sapiens OX=9606 GN=PPP6C PE=1 SV=1	7	3	3	35.1	12	305	5.69				1	1					1	1	
High	Q5VZF2	Muscleblind-like protein 2 OS=Homo sapiens OX=9606 GN=MBNL2 PE=1 SV=2	7	2	2	40.5	5	373	8.38				1	1	1				1	1	1
High	O95363	Phenylalanine--tRNA ligase, mitochondrial OS=Homo sapiens OX=9606 GN=FARS2 PE=1 SV=1	7	2	2	52.3	4	451	7.46				1		2				1		2
High	Q9H9J2	39S ribosomal protein L44, mitochondrial OS=Homo sapiens OX=9606 GN=MRPL44 PE=1 SV=1	7	3	3	37.5	7	332	8.4				1	1					1	1	
High	Q96EK6	Glucosamine 6-phosphate N-acetyltransferase OS=Homo sapiens OX=9606 GN=GNPNAT1 PE=1 SV=1	7	4	4	20.7	23	184	7.99	1			1		1	1			1		1
High	P30536	Translocator protein OS=Homo sapiens OX=9606 GN=TSPO PE=1 SV=3	7	1	1	18.8	5	169	9.36	1	1	1	1			1	1	1	1		
High	O94842	TOX high mobility group box family member 4 OS=Homo sapiens OX=9606 GN=TOX4 PE=1 SV=1	7	2	2	66.2	4	621	5.06				2	2	1				2	2	1
High	Q9C098	Serine/threonine-protein kinase DCLK3 OS=Homo sapiens OX=9606 GN=DCLK3 PE=2 SV=2	7	2	1	73.8	2	648	8.84	1	1	1			1	1	1	1			1
High	Q9GZS1	DNA-directed RNA polymerase I subunit RPA49 OS=Homo sapiens OX=9606 GN=POLR1E PE=1 SV=3	7	4	4	47.2	12	419	8.94					4						3	
High	P16152	Carbonyl reductase [NADPH] 1 OS=Homo sapiens OX=9606 GN=CBR1 PE=1 SV=3	7	2	2	30.4	8	277	8.32	2	2	1				2	2	1			
High	Q9UPN9	E3 ubiquitin-protein ligase TRIM33 OS=Homo sapiens OX=9606 GN=TRIM33 PE=1 SV=3	7	3	3	122.5	3	1127	6.67						1						1
High	P16422	Epithelial cell adhesion molecule OS=Homo sapiens OX=9606 GN=EPCAM PE=1 SV=2	7	2	2	34.9	12	314	7.46	1	1	1				1	1	1			
High	Q9Y3D0	Cytosolic iron-sulfur assembly component 2B OS=Homo sapiens OX=9606 GN=CIAO2B PE=1 SV=1	7	1	1	17.7	13	163	5.19			1	1	1	1			1	1	1	1
High	P82921	28S ribosomal protein S21, mitochondrial OS=Homo sapiens OX=9606 GN=MRPS21 PE=1 SV=3	7	3	3	10.7	39	87	9.92		1		1		1		1		1		1
High	Q9UHF7	Zinc finger transcription factor Trps1 OS=Homo sapiens OX=9606 GN=TRPS1 PE=1 SV=2	7	3	3	141.4	4	1281	7.58			1						1			
High	Q9P2R3	Rabankyrin-5 OS=Homo sapiens OX=9606 GN=ANKFY1 PE=1 SV=2	7	2	2	128.3	1	1169	6.1	1	1	2				1	1	2			
High	Q15773	Myeloid leukemia factor 2 OS=Homo sapiens OX=9606 GN=MLF2 PE=1 SV=1	7	1	1	28.1	4	248	6.9	1	1	1		1	1	1	1	1		1	1
High	Q9Y2Q5	Ragulator complex protein LAMTOR2 OS=Homo sapiens OX=9606 GN=LAMTOR2 PE=1 SV=1	7	2	2	13.5	17	125	5.4	1	1	1				1	1	1			
High	P49406	39S ribosomal protein L19, mitochondrial OS=Homo sapiens OX=9606 GN=MRPL19 PE=1 SV=2	7	3	3	33.5	14	292	9.5	1				1		1				1	
High	Q12802	A-kinase anchor protein 13 OS=Homo sapiens OX=9606 GN=AKAP13 PE=1 SV=2	7	4	4	307.4	3	2813	5.24		1	2	1				1	2	1		
High	Q13325	Interferon-induced protein with tetratricopeptide repeats 5 OS=Homo sapiens OX=9606 GN=IFIT5 PE=1 SV=1	7	3	3	55.8	10	482	7.4	2			1	1		2			1	1	
High	P20290	Transcription factor BTF3 OS=Homo sapiens OX=9606 GN=BTF3 PE=1 SV=1	7	2	2	22.2	20	206	9.38	1	1	1		2		1	1	1		2	
High	O43396	Thioredoxin-like protein 1 OS=Homo sapiens OX=9606 GN=TXNL1 PE=1 SV=3	7	1	1	32.2	6	289	4.96	1	1	1		1		1	1	1		1	
High	P00387	NADH-cytochrome b5 reductase 3 OS=Homo sapiens OX=9606 GN=CYB5R3 PE=1 SV=3	7	3	3	34.2	14	301	7.59	1	2	2				1	2	2			
High	P10398	Serine/threonine-protein kinase A-Raf OS=Homo sapiens OX=9606 GN=ARAF PE=1 SV=2	7	3	3	67.5	6	606	9.01	1	1			1		1	1			1	
High	Q9UPY8	Microtubule-associated protein RP/EB family member 3 OS=Homo sapiens OX=9606 GN=MAPRE3 PE=1 SV=1	7	2	2	32	5	281	5.54				1	1	1				1	1	1
High	Q7Z6E9	E3 ubiquitin-protein ligase RBBP6 OS=Homo sapiens OX=9606 GN=RBBP6 PE=1 SV=1	7	4	4	201.4	3	1792	9.64					2						2	
High	Q9BTE3	Mini-chromosome maintenance complex-binding protein OS=Homo sapiens OX=9606 GN=MCMBP PE=1 SV=2	7	3	3	72.9	5	642	5.87				1	2	2				1	2	2
High	Q9UNA1	Rho GTPase-activating protein 26 OS=Homo sapiens OX=9606 GN=ARHGAP26 PE=1 SV=1	7	2	2	92.2	3	814	6.64	2	2	2				2	2	2			
High	Q7Z3K3	Pogo transposable element with ZNF domain OS=Homo sapiens OX=9606 GN=POGZ PE=1 SV=2	7	3	3	155.2	3	1410	7.4		1						1				
High	O60256	Phosphoribosyl pyrophosphate synthase-associated protein 2 OS=Homo sapiens OX=9606 GN=PRPSAP2 PE=1 SV=1	7	2	1	40.9	6	369	7.44	1	1			2	1	1	1			2	1
High	Q9NVS2	39S ribosomal protein S18a, mitochondrial OS=Homo sapiens OX=9606 GN=MRPS18A PE=1 SV=1	7	1	1	22.2	5	196	10.33	1		1	1		1	1		1	1		1
High	Q8NEC7	Glutathione S-transferase C-terminal domain-containing protein OS=Homo sapiens OX=9606 GN=GSTCD PE=1 SV=2	7	1	1	71	2	633	7.78		1	1	1	1			1	1	1	1	
High	O43660	Pleiotropic regulator 1 OS=Homo sapiens OX=9606 GN=PLRG1 PE=1 SV=1	7	2	2	57.2	4	514	9.17			1		1				1		1	
High	Q9Y4E1	WASH complex subunit 2C OS=Homo sapiens OX=9606 GN=WASHC2C PE=1 SV=4	7	3	3	144.8	4	1320	4.79			1		1				1		1	
High	Q8NEU8	DCC-interacting protein 13-beta OS=Homo sapiens OX=9606 GN=APPL2 PE=1 SV=3	7	2	2	74.4	5	664	4.94	1	1	1				1	1	1			
High	P62942	Peptidyl-prolyl cis-trans isomerase FKBP1A OS=Homo sapiens OX=9606 GN=FKBP1A PE=1 SV=2	7	3	3	11.9	30	108	8.16	3	2					3	2				
High	O00303	Eukaryotic translation initiation factor 3 subunit F OS=Homo sapiens OX=9606 GN=EIF3F PE=1 SV=1	7	2	2	37.5	8	357	5.45				1		1				1		1
High	Q969L2	Protein MAL2 OS=Homo sapiens OX=9606 GN=MAL2 PE=1 SV=1	7	1	1	19.1	6	176	6.24	1	1	1		1	1	1	1	1		1	1
High	Q8IUF8	Ribosomal oxygenase 2 OS=Homo sapiens OX=9606 GN=RIOX2 PE=1 SV=1	7	2	2	52.8	5	465	6.7		1		1	1	1		1		1	1	1
High	Q9Y3D9	28S ribosomal protein S23, mitochondrial OS=Homo sapiens OX=9606 GN=MRPS23 PE=1 SV=2	7	2	2	21.8	11	190	8.9		1	1		1			1	1		1	
High	Q66K74	Microtubule-associated protein 1S OS=Homo sapiens OX=9606 GN=MAP1S PE=1 SV=2	7	2	2	112.1	4	1059	7.3	1	1	1	1		1	1	1	1	1		1
High	Q13490	Baculoviral IAP repeat-containing protein 2 OS=Homo sapiens OX=9606 GN=BIRC2 PE=1 SV=2	7	2	2	69.9	4	618	6.7	1	1	1				1	1	1			
High	Q8NC96	Adaptin ear-binding coat-associated protein 1 OS=Homo sapiens OX=9606 GN=NECAP1 PE=1 SV=2	7	2	2	29.7	7	275	6.8			1						1			
High	O15235	28S ribosomal protein S12, mitochondrial OS=Homo sapiens OX=9606 GN=MRPS12 PE=1 SV=1	7	2	2	15.2	14	138	10.29			1	1	1	1			1	1	1	1
High	P30519	Heme oxygenase 2 OS=Homo sapiens OX=9606 GN=HMOX2 PE=1 SV=2	7	2	2	36	7	316	5.41	1	1	1				1	1	1			
High	Q14814	Myocyte-specific enhancer factor 2D OS=Homo sapiens OX=9606 GN=MEF2D PE=1 SV=1	7	2	2	55.9	4	521	7.88	1		1			1	1		1			1
High	P36776	Lon protease homolog, mitochondrial OS=Homo sapiens OX=9606 GN=LONP1 PE=1 SV=2	7	4	4	106.4	5	959	6.39	1		2				1		2			
High	Q8IUQ4	E3 ubiquitin-protein ligase SIAH1 OS=Homo sapiens OX=9606 GN=SIAH1 PE=1 SV=2	7	2	2	31.1	8	282	6.8	1			1	1	1	1			1	1	1
High	O75844	CAAX prenyl protease 1 homolog OS=Homo sapiens OX=9606 GN=ZMPSTE24 PE=1 SV=2	7	2	2	54.8	6	475	7.49	1	1	2				1	1	2			
High	O00764	Pyridoxal kinase OS=Homo sapiens OX=9606 GN=PDXK PE=1 SV=1	7	1	1	35.1	3	312	6.13	1	1	1		1		1	1	1		1	
High	Q16790	Carbonic anhydrase 9 OS=Homo sapiens OX=9606 GN=CA9 PE=1 SV=2	7	2	2	49.7	7	459	4.72	1	1	2				1	1	2			
High	P99999	Cytochrome c OS=Homo sapiens OX=9606 GN=CYCS PE=1 SV=2	7	2	1	11.7	18	105	9.57			2	2	1	2			2	2	1	2
High	Q16644	MAP kinase-activated protein kinase 3 OS=Homo sapiens OX=9606 GN=MAPKAPK3 PE=1 SV=1	7	3	3	43	7	382	7.28			1		2	1			1		2	1
High	Q9BSJ2	Gamma-tubulin complex component 2 OS=Homo sapiens OX=9606 GN=TUBGCP2 PE=1 SV=2	6	2	2	102.5	3	902	6.84	2	1	1				2	1	1			
High	Q92538	Golgi-specific brefeldin A-resistance guanine nucleotide exchange factor 1 OS=Homo sapiens OX=9606 GN=GBF1 PE=1 SV=2	6	4	4	206.3	2	1859	5.73	2	2	2				2	2	2			
High	O14686	Histone-lysine N-methyltransferase 2D OS=Homo sapiens OX=9606 GN=KMT2D PE=1 SV=2	6	3	3	593	1	5537	5.58					2						2	
High	Q14C86	GTPase-activating protein and VPS9 domain-containing protein 1 OS=Homo sapiens OX=9606 GN=GAPVD1 PE=1 SV=2	6	3	3	164.9	3	1478	5.22		2	1					2	1			
High	O14965	Aurora kinase A OS=Homo sapiens OX=9606 GN=AURKA PE=1 SV=3	6	3	3	45.8	10	403	9.39					2	1					2	1
High	Q16718	NADH dehydrogenase [ubiquinone] 1 alpha subcomplex subunit 5 OS=Homo sapiens OX=9606 GN=NDUFA5 PE=1 SV=3	6	1	1	13.5	8	116	5.99			1	1	1	1			1	1	1	1
High	P18583	Protein SON OS=Homo sapiens OX=9606 GN=SON PE=1 SV=4	6	3	3	263.7	3	2426	5.64				1	3	1				1	2	1
High	Q9NP77	RNA polymerase II subunit A C-terminal domain phosphatase SSU72 OS=Homo sapiens OX=9606 GN=SSU72 PE=1 SV=1	6	1	1	22.6	6	194	5.33				1	1	1				1	1	1
High	Q6ZW49	PAX-interacting protein 1 OS=Homo sapiens OX=9606 GN=PAXIP1 PE=1 SV=2	6	2	2	121.3	2	1069	6.7	1	1	1		1	1	1	1	1		1	1
High	O00116	Alkyldihydroxyacetonephosphate synthase, peroxisomal OS=Homo sapiens OX=9606 GN=AGPS PE=1 SV=1	6	4	4	72.9	9	658	7.34			1			1			1			1
High	Q12959	Disks large homolog 1 OS=Homo sapiens OX=9606 GN=DLG1 PE=1 SV=2	6	1	1	100.4	1	904	5.76			1	1		1			1	1		1
High	P0CG13	Chromosome transmission fidelity protein 8 homolog OS=Homo sapiens OX=9606 GN=CHTF8 PE=1 SV=1	6	2	2	13.3	17	121	8.18	1		1				1		1			
High	Q9Y606	tRNA pseudouridine synthase A OS=Homo sapiens OX=9606 GN=PUS1 PE=1 SV=3	6	3	3	47.4	9	427	8.41				1	1	1				1	1	1
High	Q9H4Z3	mRNA (2'-O-methyladenosine-N(6)-)-methyltransferase OS=Homo sapiens OX=9606 GN=PCIF1 PE=1 SV=1	6	1	1	80.6	1	704	7.42	1			1	1	1	1			1	1	1
High	Q01085	Nucleolysin TIAR OS=Homo sapiens OX=9606 GN=TIAL1 PE=1 SV=1	6	2	2	41.6	8	375	7.74	2	1	1		1		2	1	1		1	
High	Q96EL2	28S ribosomal protein S24, mitochondrial OS=Homo sapiens OX=9606 GN=MRPS24 PE=1 SV=1	6	1	1	19	5	167	9.38				1	1	1				1	1	1
High	P28347	Transcriptional enhancer factor TEF-1 OS=Homo sapiens OX=9606 GN=TEAD1 PE=1 SV=2	6	1	1	47.9	2	426	8.15	1	1	1	1	1	1	1	1	1	1	1	1
High	Q8WUF5	RelA-associated inhibitor OS=Homo sapiens OX=9606 GN=PPP1R13L PE=1 SV=4	6	2	2	89	4	828	6.81					1	2					1	2
High	Q14676	Mediator of DNA damage checkpoint protein 1 OS=Homo sapiens OX=9606 GN=MDC1 PE=1 SV=3	6	3	3	226.5	3	2089	5.47	1	1	2				1	1	2			
High	Q8IX01	SURP and G-patch domain-containing protein 2 OS=Homo sapiens OX=9606 GN=SUGP2 PE=1 SV=2	6	2	2	120.1	2	1082	7.28				1	1					1	1	
High	O15156	Zinc finger and BTB domain-containing protein 7B OS=Homo sapiens OX=9606 GN=ZBTB7B PE=1 SV=2	6	1	1	58	2	539	5.86			1	1	1	1			1	1	1	1
High	Q9UNX4	WD repeat-containing protein 3 OS=Homo sapiens OX=9606 GN=WDR3 PE=1 SV=1	6	1	1	106	1	943	6.64	1		1		1		1		1		1	
High	O75676	Ribosomal protein S6 kinase alpha-4 OS=Homo sapiens OX=9606 GN=RPS6KA4 PE=1 SV=1	6	4	4	85.6	5	772	8.28	2	1	2				2	1	2			
High	Q9UMD9	Collagen alpha-1(XVII) chain OS=Homo sapiens OX=9606 GN=COL17A1 PE=1 SV=3	6	2	2	150.3	1	1497	8.79						1						1
High	Q14728	Major facilitator superfamily domain-containing protein 10 OS=Homo sapiens OX=9606 GN=MFSD10 PE=1 SV=1	6	1	1	48.3	3	455	9.6	1	1	1				1	1	1			
High	O14617	AP-3 complex subunit delta-1 OS=Homo sapiens OX=9606 GN=AP3D1 PE=1 SV=1	6	4	4	130.1	5	1153	8.48												
High	O43251	RNA binding protein fox-1 homolog 2 OS=Homo sapiens OX=9606 GN=RBFOX2 PE=1 SV=3	6	2	2	41.3	6	390	7.27		1		1		1		1		1		1
High	O75390	Citrate synthase, mitochondrial OS=Homo sapiens OX=9606 GN=CS PE=1 SV=2	6	2	2	51.7	5	466	8.32				1	2	1				1	2	1
High	O95456	Proteasome assembly chaperone 1 OS=Homo sapiens OX=9606 GN=PSMG1 PE=1 SV=1	6	2	2	32.8	10	288	7.17		2	1			1		2	1			1
High	P31150	Rab GDP dissociation inhibitor alpha OS=Homo sapiens OX=9606 GN=GDI1 PE=1 SV=2	6	2	1	50.6	6	447	5.14	1	1	2				1	1	2			
High	Q99496	E3 ubiquitin-protein ligase RING2 OS=Homo sapiens OX=9606 GN=RNF2 PE=1 SV=1	6	3	2	37.6	14	336	6.84	1		1		1		1		1		1	
High	Q9HCN8	Stromal cell-derived factor 2-like protein 1 OS=Homo sapiens OX=9606 GN=SDF2L1 PE=1 SV=2	6	1	1	23.6	9	221	7.03	1	1	1				1	1	1			
High	Q92686	Neurogranin OS=Homo sapiens OX=9606 GN=NRGN PE=1 SV=1	6	2	2	7.6	60	78	7.87			1	1	2	1			1	1	2	1
High	P62314	Small nuclear ribonucleoprotein Sm D1 OS=Homo sapiens OX=9606 GN=SNRPD1 PE=1 SV=1	6	1	1	13.3	11	119	11.56	1			1	1		1			1	1	
High	Q9NT62	Ubiquitin-like-conjugating enzyme ATG3 OS=Homo sapiens OX=9606 GN=ATG3 PE=1 SV=1	6	2	2	35.8	7	314	4.74					1						1	
High	Q86V21	Acetoacetyl-CoA synthetase OS=Homo sapiens OX=9606 GN=AACS PE=1 SV=1	6	2	2	75.1	3	672	6.24	1		1				1		1			
High	O14545	TRAF-type zinc finger domain-containing protein 1 OS=Homo sapiens OX=9606 GN=TRAFD1 PE=1 SV=1	6	1	1	64.8	2	582	5.29	1	1				1	1	1				1
High	Q14318	Peptidyl-prolyl cis-trans isomerase FKBP8 OS=Homo sapiens OX=9606 GN=FKBP8 PE=1 SV=2	6	1	1	44.5	2	412	4.84				1	1	1				1	1	1
High	Q15386	Ubiquitin-protein ligase E3C OS=Homo sapiens OX=9606 GN=UBE3C PE=1 SV=3	6	4	4	123.8	4	1083	6.71	1	1	3				1	1	3			
High	P32321	Deoxycytidylate deaminase OS=Homo sapiens OX=9606 GN=DCTD PE=1 SV=2	6	2	2	20	15	178	7.56	1				2		1				2	
High	Q5TC63	Growth hormone-regulated TBC protein 1 OS=Homo sapiens OX=9606 GN=GRTP1 PE=1 SV=4	6	3	3	38.5	13	336	8.78	1	1	1				1	1	1			
High	Q9UMR2	ATP-dependent RNA helicase DDX19B OS=Homo sapiens OX=9606 GN=DDX19B PE=1 SV=1	6	4	4	53.9	14	479	6.3		1	1		1			1	1		1	
High	Q9BTV4	Transmembrane protein 43 OS=Homo sapiens OX=9606 GN=TMEM43 PE=1 SV=1	6	4	4	44.8	16	400	8.13	1		1				1		1			
High	P84074	Neuron-specific calcium-binding protein hippocalcin OS=Homo sapiens OX=9606 GN=HPCA PE=1 SV=2	6	3	3	22.4	16	193	4.97		1	1					1	1			
High	Q9UI12	V-type proton ATPase subunit H OS=Homo sapiens OX=9606 GN=ATP6V1H PE=1 SV=1	6	3	3	55.8	10	483	6.48	2	2	2				2	2	2			
High	Q9Y2R5	28S ribosomal protein S17, mitochondrial OS=Homo sapiens OX=9606 GN=MRPS17 PE=1 SV=1	6	2	2	14.5	29	130	9.85					1						1	
High	Q9BSG0	Protease-associated domain-containing protein 1 OS=Homo sapiens OX=9606 GN=PRADC1 PE=1 SV=1	6	2	2	21	11	188	5.47	2	2	2				2	2	2			
High	O43318	Mitogen-activated protein kinase kinase kinase 7 OS=Homo sapiens OX=9606 GN=MAP3K7 PE=1 SV=1	6	2	2	67.2	5	606	7.11	1	1	1		1		1	1	1		1	
High	O43324	Eukaryotic translation elongation factor 1 epsilon-1 OS=Homo sapiens OX=9606 GN=EEF1E1 PE=1 SV=1	6	3	3	19.8	18	174	8.54			1		1				1		1	
High	P12111	Collagen alpha-3(VI) chain OS=Homo sapiens OX=9606 GN=COL6A3 PE=1 SV=5	6	3	3	343.5	1	3177	6.68				1	3	2				1	3	2
High	Q01970	1-phosphatidylinositol 4,5-bisphosphate phosphodiesterase beta-3 OS=Homo sapiens OX=9606 GN=PLCB3 PE=1 SV=2	6	4	4	138.7	4	1234	5.9	1		1				1		1			
High	Q9Y696	Chloride intracellular channel protein 4 OS=Homo sapiens OX=9606 GN=CLIC4 PE=1 SV=4	6	1	1	28.8	5	253	5.59	1	1	3	1			1	1	1	1		
High	Q9BYD6	39S ribosomal protein L1, mitochondrial OS=Homo sapiens OX=9606 GN=MRPL1 PE=1 SV=2	6	3	3	36.9	14	325	8.78			1			1			1			1
High	Q99570	Phosphoinositide 3-kinase regulatory subunit 4 OS=Homo sapiens OX=9606 GN=PIK3R4 PE=1 SV=3	6	5	5	153	5	1358	7.17	1	2	2				1	2	2			
High	Q8N1G2	Cap-specific mRNA (nucleoside-2'-O-)-methyltransferase 1 OS=Homo sapiens OX=9606 GN=CMTR1 PE=1 SV=1	6	4	4	95.3	6	835	7.05		1	1	1	1			1	1	1	1	
High	Q6W2J9	BCL-6 corepressor OS=Homo sapiens OX=9606 GN=BCOR PE=1 SV=1	6	2	1	192.1	1	1755	6.48				2	1	1				2	1	1
High	O95202	Mitochondrial proton/calcium exchanger protein OS=Homo sapiens OX=9606 GN=LETM1 PE=1 SV=1	6	2	2	83.3	3	739	6.7	1		1			1	1		1			1
High	P09001	39S ribosomal protein L3, mitochondrial OS=Homo sapiens OX=9606 GN=MRPL3 PE=1 SV=1	6	1	1	38.6	3	348	9.48			1	1		1			1	1		1
High	Q13042	Cell division cycle protein 16 homolog OS=Homo sapiens OX=9606 GN=CDC16 PE=1 SV=2	6	3	3	71.6	6	620	5.85	3	2	1				3	2	1			
High	Q96QC0	Serine/threonine-protein phosphatase 1 regulatory subunit 10 OS=Homo sapiens OX=9606 GN=PPP1R10 PE=1 SV=1	6	2	2	99	4	940	9.17					1	1					1	1
High	P56182	Ribosomal RNA processing protein 1 homolog A OS=Homo sapiens OX=9606 GN=RRP1 PE=1 SV=1	6	1	1	52.8	2	461	9.33	1	1		1			1	1		1		
High	P52926	High mobility group protein HMGI-C OS=Homo sapiens OX=9606 GN=HMGA2 PE=1 SV=1	6	2	2	11.8	31	109	10.62	2	1	1		1		2	1	1		1	
High	Q9P253	Vacuolar protein sorting-associated protein 18 homolog OS=Homo sapiens OX=9606 GN=VPS18 PE=1 SV=2	6	3	3	110.1	3	973	6.07	1	2	1				1	2	1			
High	Q16851	UTP--glucose-1-phosphate uridylyltransferase OS=Homo sapiens OX=9606 GN=UGP2 PE=1 SV=5	6	1	1	56.9	3	508	8.15	1	1	1				1	1	1			
High	Q04206	Transcription factor p65 OS=Homo sapiens OX=9606 GN=RELA PE=1 SV=2	6	3	3	60.2	7	551	5.68	1	1			1	1	1	1			1	1

High	O60725	Protein-S-isoprenylcysteine O-methyltransferase OS=Homo sapiens OX=9606 GN=ICMT PE=1 SV=1	6	1	1	31.9	6	284	7.96	1	1	1				1	1	1			
High	Q12965	Unconventional myosin-Ie OS=Homo sapiens OX=9606 GN=MYO1E PE=1 SV=2	6	2	2	127	2	1108	8.92	1	1	1			1	1	1	1			1
High	Q8WUY1	Protein THEM6 OS=Homo sapiens OX=9606 GN=THEM6 PE=1 SV=2	6	3	3	23.9	11	208	9.55			1						1			
High	Q96BI1	Solute carrier family 22 member 18 OS=Homo sapiens OX=9606 GN=SLC22A18 PE=1 SV=3	6	2	2	44.8	4	424	9.57	1	1	1				1	1	1			
High	Q13823	Nucleolar GTP-binding protein 2 OS=Homo sapiens OX=9606 GN=GNL2 PE=1 SV=1	6	3	3	83.6	6	731	9.25												
High	O94992	Protein HEXIM1 OS=Homo sapiens OX=9606 GN=HEXIM1 PE=1 SV=1	6	3	3	40.6	11	359	4.89				1	2	1				1	2	1
High	P49247	Ribose-5-phosphate isomerase OS=Homo sapiens OX=9606 GN=RPIA PE=1 SV=3	6	2	2	33.2	8	311	8.54				1		1				1		1
High	O95639	Cleavage and polyadenylation specificity factor subunit 4 OS=Homo sapiens OX=9606 GN=CPSF4 PE=1 SV=1	6	2	2	30.2	15	269	8.31				2	1	1				2	1	1
High	O75683	Surfeit locus protein 6 OS=Homo sapiens OX=9606 GN=SURF6 PE=1 SV=3	6	2	2	41.4	6	361	10.64				1		1				1		1
High	O14683	Tumor protein p53-inducible protein 11 OS=Homo sapiens OX=9606 GN=TP53I11 PE=1 SV=2	6	2	2	21	13	189	9.55					1						1	
High	Q96CS3	FAS-associated factor 2 OS=Homo sapiens OX=9606 GN=FAF2 PE=1 SV=2	6	3	3	52.6	9	445	5.62	2	2	2				2	2	2			
High	Q8WVX9	Fatty acyl-CoA reductase 1 OS=Homo sapiens OX=9606 GN=FAR1 PE=1 SV=1	6	4	4	59.3	10	515	9.17	2	1	3				2	1	3			
High	P45973	Chromobox protein homolog 5 OS=Homo sapiens OX=9606 GN=CBX5 PE=1 SV=1	6	2	2	22.2	13	191	5.86	1				1	1	1				1	1
High	Q9Y6A5	Transforming acidic coiled-coil-containing protein 3 OS=Homo sapiens OX=9606 GN=TACC3 PE=1 SV=1	6	2	2	90.3	2	838	5.05				1	1					1	1	
High	O14772	Fucose-1-phosphate guanylyltransferase OS=Homo sapiens OX=9606 GN=FPGT PE=1 SV=3	6	2	2	68	3	607	6.87		1						1				
High	O00151	PDZ and LIM domain protein 1 OS=Homo sapiens OX=9606 GN=PDLIM1 PE=1 SV=4	6	2	2	36	6	329	7.02		1			1			1			1	
High	Q01664	Transcription factor AP-4 OS=Homo sapiens OX=9606 GN=TFAP4 PE=1 SV=2	6	3	3	38.7	13	338	5.87	2	2	2				2	2	2			
High	Q9UBW7	Zinc finger MYM-type protein 2 OS=Homo sapiens OX=9606 GN=ZMYM2 PE=1 SV=1	6	4	4	154.8	5	1377	6.34					1						1	
High	Q9BTW9	Tubulin-specific chaperone D OS=Homo sapiens OX=9606 GN=TBCD PE=1 SV=2	6	1	1	132.5	1	1192	6.19		1	1			1		1	1			1
High	Q13488	V-type proton ATPase 116 kDa subunit a 3 OS=Homo sapiens OX=9606 GN=TCIRG1 PE=1 SV=3	6	4	4	92.9	5	830	7.12	1						1					
High	P13693	Translationally-controlled tumor protein OS=Homo sapiens OX=9606 GN=TPT1 PE=1 SV=1	6	1	1	19.6	8	172	4.93	1	1	1				1	1	1			
High	O75787	Renin receptor OS=Homo sapiens OX=9606 GN=ATP6AP2 PE=1 SV=2	6	2	2	39	5	350	6.1	1	1	1				1	1	1			
High	Q9UBU8	Mortality factor 4-like protein 1 OS=Homo sapiens OX=9606 GN=MORF4L1 PE=1 SV=2	6	2	1	41.4	6	362	9.28				1	1	2				1	1	2
High	Q8NEV8	Exophilin-5 OS=Homo sapiens OX=9606 GN=EXPH5 PE=1 SV=4	6	1	1	222.4	1	1989	7.81	1	1	1				1	1	1			
High	P35556	Fibrillin-2 OS=Homo sapiens OX=9606 GN=FBN2 PE=1 SV=3	6	2	1	314.6	1	2912	4.86				1	3	2				1	2	2
High	Q8WXE1	ATR-interacting protein OS=Homo sapiens OX=9606 GN=ATRIP PE=1 SV=1	6	1	1	85.8	1	791	6.32				1	1	1				1	1	1
High	P19838	Nuclear factor NF-kappa-B p105 subunit OS=Homo sapiens OX=9606 GN=NFKB1 PE=1 SV=2	6	4	4	105.3	5	968	5.4												
High	Q9P2D0	Inhibitor of Bruton tyrosine kinase OS=Homo sapiens OX=9606 GN=IBTK PE=1 SV=3	6	4	4	150.4	5	1353	7.71												
High	Q8WXH0	Nesprin-2 OS=Homo sapiens OX=9606 GN=SYNE2 PE=1 SV=3	6	4	4	795.9	1	6885	5.36		1	2					1	2			
High	Q04656	Copper-transporting ATPase 1 OS=Homo sapiens OX=9606 GN=ATP7A PE=1 SV=4	6	3	3	163.3	3	1500	6.33	1	1	1				1	1	1			
High	Q9Y6K0	Choline/ethanolaminephosphotransferase 1 OS=Homo sapiens OX=9606 GN=CEPT1 PE=1 SV=1	6	1	1	46.5	2	416	8.21	1	1	1			1	1	1	1	1		
High	P13051	Uracil-DNA glycosylase OS=Homo sapiens OX=9606 GN=UNG PE=1 SV=2	6	3	3	34.6	14	313	9.32					2						2	
High	P51570	Galactokinase OS=Homo sapiens OX=9606 GN=GALK1 PE=1 SV=1	6	3	3	42.2	8	392	6.46	1	1	1				1	1	1			
High	P00167	Cytochrome b5 OS=Homo sapiens OX=9606 GN=CYB5A PE=1 SV=2	6	2	2	15.3	25	134	4.96				1	1	2				1	1	2
High	Q08378	Golgin subfamily A member 3 OS=Homo sapiens OX=9606 GN=GOLGA3 PE=1 SV=2	6	3	3	167.3	3	1498	5.44		1						1				
High	Q9NQ55	Suppressor of SWI4 1 homolog OS=Homo sapiens OX=9606 GN=PPAN PE=2 SV=1	6	2	2	53.2	4	473	10.13				1		1				1		1
High	O00487	26S proteasome non-ATPase regulatory subunit 14 OS=Homo sapiens OX=9606 GN=PSMD14 PE=1 SV=1	6	1	1	34.6	4	310	6.52				1	1	1				1	1	1
High	Q15427	Splicing factor 3B subunit 4 OS=Homo sapiens OX=9606 GN=SF3B4 PE=1 SV=1	6	1	1	44.4	3	424	8.56				1	1	1				1	1	1
High	Q8TBX8	Phosphatidylinositol 5-phosphate 4-kinase type-2 gamma OS=Homo sapiens OX=9606 GN=PIP4K2C PE=1 SV=3	6	2	2	47.3	6	421	6.84	2	1	1				2	1	1			
High	Q7Z4G4	tRNA (guanine(10)-N2)-methyltransferase homolog OS=Homo sapiens OX=9606 GN=TRMT11 PE=1 SV=1	6	2	2	53.4	3	463	7.78			1			1			1			1
High	P46439	Glutathione S-transferase Mu 5 OS=Homo sapiens OX=9606 GN=GSTM5 PE=1 SV=3	6	2	2	25.7	8	218	7.39				2	2	2				2	2	2
High	P29590	Protein PML OS=Homo sapiens OX=9606 GN=PML PE=1 SV=3	6	1	1	97.5	1	882	6.21			1	1		1			1	1		1
High	Q9BSH4	Translational activator of cytochrome c oxidase 1 OS=Homo sapiens OX=9606 GN=TACO1 PE=1 SV=1	6	2	2	32.5	9	297	8.13		1						1				
High	Q9ULX3	RNA-binding protein NOB1 OS=Homo sapiens OX=9606 GN=NOB1 PE=1 SV=1	6	2	2	46.6	3	412	7.18		1		1		1		1		1		1
High	P02662	SWISS-PROT:P02662 Alpha-S1-casein - Bos taurus (Bovine).	6	2	2	23	14	199	4.94		1						1				
High	Q96IX5	ATP synthase membrane subunit K, mitochondrial OS=Homo sapiens OX=9606 GN=ATP5MK PE=1 SV=1	6	1	1	6.5	17	58	9.76	1		1	1	1	1	1		1	1	1	1
High	Q8IXK0	Polyhomeotic-like protein 2 OS=Homo sapiens OX=9606 GN=PHC2 PE=1 SV=1	6	3	3	90.7	3	858	8.69												
High	Q96EV2	RNA-binding protein 33 OS=Homo sapiens OX=9606 GN=RBM33 PE=1 SV=3	6	4	4	129.9	6	1170	6.93					1						1	
High	O95071	E3 ubiquitin-protein ligase UBR5 OS=Homo sapiens OX=9606 GN=UBR5 PE=1 SV=2	6	4	4	309.2	2	2799	5.85												
High	O94888	UBX domain-containing protein 7 OS=Homo sapiens OX=9606 GN=UBXN7 PE=1 SV=2	6	1	1	54.8	3	489	5.16					2						1	
High	Q9NWT1	p21-activated protein kinase-interacting protein 1 OS=Homo sapiens OX=9606 GN=PAK1IP1 PE=1 SV=2	6	2	2	43.9	6	392	8.91				1	1	1				1	1	1
High	Q9H583	HEAT repeat-containing protein 1 OS=Homo sapiens OX=9606 GN=HEATR1 PE=1 SV=3	6	4	4	242.2	2	2144	6.54	2	3	1				2	3	1			
High	P40692	DNA mismatch repair protein Mlh1 OS=Homo sapiens OX=9606 GN=MLH1 PE=1 SV=1	6	2	2	84.5	3	756	5.72	1		1			1	1		1			1
High	Q7Z3U7	Protein MON2 homolog OS=Homo sapiens OX=9606 GN=MON2 PE=1 SV=3	6	3	3	190.2	2	1717	6.06	2	3	1				2	3	1			
High	Q05048	Cleavage stimulation factor subunit 1 OS=Homo sapiens OX=9606 GN=CSTF1 PE=1 SV=1	6	2	2	48.3	6	431	6.58				1	1	1				1	1	1
High	P40925	Malate dehydrogenase, cytoplasmic OS=Homo sapiens OX=9606 GN=MDH1 PE=1 SV=4	6	2	2	36.4	9	334	7.36				2		2				2		2
High	P53602	Diphosphomevalonate decarboxylase OS=Homo sapiens OX=9606 GN=MVD PE=1 SV=1	6	3	3	43.4	8	400	7.23	1	1		1	1		1	1		1	1	
High	Q9HCM4	Band 4.1-like protein 5 OS=Homo sapiens OX=9606 GN=EPB41L5 PE=1 SV=3	6	2	2	81.8	4	733	6.58	1	1	1				1	1	1			
High	Q15287	RNA-binding protein with serine-rich domain 1 OS=Homo sapiens OX=9606 GN=RNPS1 PE=1 SV=1	6	1	1	34.2	3	305	11.84				1	1	1				1	1	1
High	Q9NRX2	39S ribosomal protein L17, mitochondrial OS=Homo sapiens OX=9606 GN=MRPL17 PE=1 SV=1	6	1	1	20	4	175	10.11			1	1		1			1	1		1
High	Q6FI81	Anamorsin OS=Homo sapiens OX=9606 GN=CIAPIN1 PE=1 SV=2	6	3	3	33.6	12	312	5.62	1				1		1				1	
High	Q92610	Zinc finger protein 592 OS=Homo sapiens OX=9606 GN=ZNF592 PE=1 SV=2	6	3	3	137.4	5	1267	7.84												
High	Q9UEG4	Zinc finger protein 629 OS=Homo sapiens OX=9606 GN=ZNF629 PE=1 SV=2	6	1	1	96.6	2	869	7.93	1	1	1	1	1	1	1	1	1	1	1	1
High	Q9NRP0	Oligosaccharyltransferase complex subunit OSTC OS=Homo sapiens OX=9606 GN=OSTC PE=1 SV=1	6	1	1	16.8	8	149	9.13	1	1	1		1		1	1	1		1	
High	Q13330	Metastasis-associated protein MTA1 OS=Homo sapiens OX=9606 GN=MTA1 PE=1 SV=2	6	3	2	80.7	6	715	9.26				1						1		
High	Q5XKP0	MICOS complex subunit MIC13 OS=Homo sapiens OX=9606 GN=MICOS13 PE=1 SV=1	6	2	2	13.1	23	118	9.42		1	1					1	1			
High	Q9BUT9	MAPK regulated corepressor interacting protein 2 OS=Homo sapiens OX=9606 GN=MCRIP2 PE=1 SV=2	6	1	1	17.8	7	160	9.41			1	1	1				1	1	1	
High	O00767	Stearoyl-CoA desaturase OS=Homo sapiens OX=9606 GN=SCD PE=1 SV=2	6	2	2	41.5	11	359	9	2	2	2				2	2	2			
High	Q9GZT9	Egl nine homolog 1 OS=Homo sapiens OX=9606 GN=EGLN1 PE=1 SV=1	6	4	4	46	13	426	8.53				1		1				1		1
High	Q7L8W6	Diphthine--ammonia ligase OS=Homo sapiens OX=9606 GN=DPH6 PE=1 SV=3	6	3	3	30.3	12	267	5.4		1		1	1	1		1		1	1	1
High	Q96GW9	Methionine--tRNA ligase, mitochondrial OS=Homo sapiens OX=9606 GN=MARS2 PE=1 SV=2	6	2	2	66.5	3	593	8.09	1	1		1		1	1	1		1		1
High	P05556	Integrin beta-1 OS=Homo sapiens OX=9606 GN=ITGB1 PE=1 SV=2	6	2	2	88.4	3	798	5.39	1	1	2				1	1	2			
High	O94880	PHD finger protein 14 OS=Homo sapiens OX=9606 GN=PHF14 PE=1 SV=2	6	3	3	100	5	888	5.33	1	1	3				1	1	2			
High	Q29RF7	Sister chromatid cohesion protein PDS5 homolog A OS=Homo sapiens OX=9606 GN=PDS5A PE=1 SV=1	6	2	2	150.7	2	1337	7.91			1		1				1		1	
High	Q6PCB5	Lysine-specific demethylase RSBN1L OS=Homo sapiens OX=9606 GN=RSBN1L PE=1 SV=2	6	3	3	94.8	3	846	8.78						1						1
High	O14776	Transcription elongation regulator 1 OS=Homo sapiens OX=9606 GN=TCERG1 PE=1 SV=2	6	2	2	123.8	2	1098	8.65	1	1	1				1	1	1			
High	Q9BYC9	39S ribosomal protein L20, mitochondrial OS=Homo sapiens OX=9606 GN=MRPL20 PE=1 SV=1	6	1	1	17.4	5	149	10.86				1	1	1				1	1	1
High	Q96RE7	Nucleus accumbens-associated protein 1 OS=Homo sapiens OX=9606 GN=NACC1 PE=1 SV=1	6	2	2	57.2	4	527	5.74												
High	Q9NZN8	CCR4-NOT transcription complex subunit 2 OS=Homo sapiens OX=9606 GN=CNOT2 PE=1 SV=1	6	4	4	59.7	11	540	7.66												
High	Q9HD33	39S ribosomal protein L47, mitochondrial OS=Homo sapiens OX=9606 GN=MRPL47 PE=1 SV=2	6	2	2	29.4	8	250	10.37					1						1	
High	Q96BA8	Cyclic AMP-responsive element-binding protein 3-like protein 1 OS=Homo sapiens OX=9606 GN=CREB3L1 PE=1 SV=1	6	3	3	57	8	519	5.17				1	2					1	2	
High	Q00577	Transcriptional activator protein Pur-alpha OS=Homo sapiens OX=9606 GN=PURA PE=1 SV=2	5	3	3	34.9	17	322	6.44					1						1	
High	O43617	Trafficking protein particle complex subunit 3 OS=Homo sapiens OX=9606 GN=TRAPPC3 PE=1 SV=1	5	2	2	20.3	10	180	4.96					1						1	
High	O14879	Interferon-induced protein with tetratricopeptide repeats 3 OS=Homo sapiens OX=9606 GN=IFIT3 PE=1 SV=1	5	1	1	56	2	490	5.2	1		1	1	1	1	1		1	1	1	1
High	Q86XN8	RNA-binding protein MEX3D OS=Homo sapiens OX=9606 GN=MEX3D PE=1 SV=3	5	1	1	64.8	1	651	5.35	1	1		1	1	1	1	1		1	1	1
High	Q8TED0	U3 small nucleolar RNA-associated protein 15 homolog OS=Homo sapiens OX=9606 GN=UTP15 PE=1 SV=3	5	4	4	58.4	8	518	9.11					2						2	
High	Q96B21	Transmembrane protein 45B OS=Homo sapiens OX=9606 GN=TMEM45B PE=1 SV=1	5	1	1	31.8	3	275	6.95				1	1					1	1	
High	P08133	Annexin A6 OS=Homo sapiens OX=9606 GN=ANXA6 PE=1 SV=3	5	4	4	75.8	5	673	5.6				3	1	1				3	1	1
High	O43688	Phospholipid phosphatase 2 OS=Homo sapiens OX=9606 GN=PLPP2 PE=1 SV=1	5	2	2	32.6	8	288	8.35			1		1				1		1	
High	Q8WZ82	Esterase OVCA2 OS=Homo sapiens OX=9606 GN=OVCA2 PE=1 SV=1	5	2	2	24.4	10	227	6.89		1	1	1				1	1	1		
High	Q13882	Protein-tyrosine kinase 6 OS=Homo sapiens OX=9606 GN=PTK6 PE=1 SV=1	5	2	1	51.8	4	451	7.03					1						1	
High	O43570	Carbonic anhydrase 12 OS=Homo sapiens OX=9606 GN=CA12 PE=1 SV=1	5	1	1	39.4	4	354	7.23		1	1					1	1			
High	Q86T03	Type 1 phosphatidylinositol 4,5-bisphosphate 4-phosphatase OS=Homo sapiens OX=9606 GN=PIP4P1 PE=1 SV=1	5	1	1	29.5	8	277	8.91	1	1	1				1	1	1			
High	Q03519	Antigen peptide transporter 2 OS=Homo sapiens OX=9606 GN=TAP2 PE=1 SV=1	5	1	1	75.6	1	686	8.02	1	1	1			1	1	1	1			1
High	Q7L8L6	FAST kinase domain-containing protein 5, mitochondrial OS=Homo sapiens OX=9606 GN=FASTKD5 PE=1 SV=1	5	2	2	86.5	2	764	8.13	2	2	1				2	2	1			
High	O14682	Ectoderm-neural cortex protein 1 OS=Homo sapiens OX=9606 GN=ENC1 PE=1 SV=2	5	2	2	66.1	3	589	6.84	1		1		2		1		1		2	
High	Q9H147	Deoxynucleotidyltransferase terminal-interacting protein 1 OS=Homo sapiens OX=9606 GN=DNTTIP1 PE=1 SV=2	5	3	3	37	14	329	8.97												
High	Q8IWR0	Zinc finger CCCH domain-containing protein 7A OS=Homo sapiens OX=9606 GN=ZC3H7A PE=1 SV=1	5	1	1	110.5	2	971	7.3	1	1	1				1	1	1			
High	Q6UX53	Thiol S-methyltransferase METTL7B OS=Homo sapiens OX=9606 GN=METTL7B PE=1 SV=2	5	2	2	27.8	9	244	8.38			1		1				1		1	
High	Q16514	Transcription initiation factor TFIID subunit 12 OS=Homo sapiens OX=9606 GN=TAF12 PE=1 SV=1	5	1	1	17.9	6	161	7.99				1	1					1	1	
High	Q15542	Transcription initiation factor TFIID subunit 5 OS=Homo sapiens OX=9606 GN=TAF5 PE=1 SV=3	5	2	2	86.8	4	800	5.64				1						1		
High	Q8NB15	Zinc finger protein 511 OS=Homo sapiens OX=9606 GN=ZNF511 PE=1 SV=2	5	1	1	28.2	6	252	7.66		1	1			1		1	1			1
High	Q9BYD3	39S ribosomal protein L4, mitochondrial OS=Homo sapiens OX=9606 GN=MRPL4 PE=1 SV=1	5	2	2	34.9	9	311	9.72	1	1	1				1	1	1			
High	O43278	Kunitz-type protease inhibitor 1 OS=Homo sapiens OX=9606 GN=SPINT1 PE=1 SV=2	5	3	3	58.4	6	529	6.29												
High	Q14494	Endoplasmic reticulum membrane sensor NFE2L1 OS=Homo sapiens OX=9606 GN=NFE2L1 PE=1 SV=1	5	2	2	84.7	2	772	4.64				1	1	2				1	1	2
High	Q96T23	Remodeling and spacing factor 1 OS=Homo sapiens OX=9606 GN=RSF1 PE=1 SV=2	5	3	3	163.7	4	1441	5.01	1		2				1		2			
High	P29508	Serpin B3 OS=Homo sapiens OX=9606 GN=SERPINB3 PE=1 SV=2	5	3	3	44.5	6	390	6.81				2	2	1				2	2	1
High	Q96IR7	4-hydroxyphenylpyruvate dioxygenase-like protein OS=Homo sapiens OX=9606 GN=HPDL PE=1 SV=1	5	2	2	39.4	11	371	7.03	1	1	1				1	1	1			
High	O00429	Dynamin-1-like protein OS=Homo sapiens OX=9606 GN=DNM1L PE=1 SV=2	5	4	4	81.8	10	736	6.81	1		2		1		1		2		1	
High	Q8ND56	Protein LSM14 homolog A OS=Homo sapiens OX=9606 GN=LSM14A PE=1 SV=3	5	2	2	50.5	4	463	9.52			1	1	1				1	1	1	
High	O14734	Acyl-coenzyme A thioesterase 8 OS=Homo sapiens OX=9606 GN=ACOT8 PE=1 SV=1	5	2	2	35.9	13	319	7.56	1	1	1				1	1	1			
High	Q15375	Ephrin type-A receptor 7 OS=Homo sapiens OX=9606 GN=EPHA7 PE=1 SV=3	5	3	1	112	3	998	5.8				2		1				2		1
High	P11279	Lysosome-associated membrane glycoprotein 1 OS=Homo sapiens OX=9606 GN=LAMP1 PE=1 SV=3	5	2	2	44.9	4	417	8.75	2		2			1	2		2			1
High	Q9Y6G3	39S ribosomal protein L42, mitochondrial OS=Homo sapiens OX=9606 GN=MRPL42 PE=1 SV=1	5	1	1	16.7	11	142	8.35					1	1					1	1
High	Q9H0S4	Probable ATP-dependent RNA helicase DDX47 OS=Homo sapiens OX=9606 GN=DDX47 PE=1 SV=1	5	3	3	50.6	10	455	9.1			1		1				1		1	
High	Q9Y4A5	Transformation/transcription domain-associated protein OS=Homo sapiens OX=9606 GN=TRRAP PE=1 SV=3	5	3	3	437.3	1	3859	8.19	1		1		1		1		1		1	
High	Q15785	Mitochondrial import receptor subunit TOM34 OS=Homo sapiens OX=9606 GN=TOMM34 PE=1 SV=2	5	3	3	34.5	9	309	8.98		1		1		2		1		1		2
High	Q5T200	Zinc finger CCCH domain-containing protein 13 OS=Homo sapiens OX=9606 GN=ZC3H13 PE=1 SV=1	5	3	3	196.5	3	1668	9.42					1						1	
High	Q13884	Beta-1-syntrophin OS=Homo sapiens OX=9606 GN=SNTB1 PE=1 SV=3	5	3	3	58	9	538	8.63			1						1			
High	Q9H4L5	Oxysterol-binding protein-related protein 3 OS=Homo sapiens OX=9606 GN=OSBPL3 PE=1 SV=1	5	1	1	101.2	2	887	6.87		1	1					1	1			
High	O95149	Snurportin-1 OS=Homo sapiens OX=9606 GN=SNUPN PE=1 SV=1	5	2	2	41.1	5	360	6.64			1	1	1	2			1	1	1	2
High	Q13443	Disintegrin and metalloproteinase domain-containing protein 9 OS=Homo sapiens OX=9606 GN=ADAM9 PE=1 SV=1	5	2	2	90.5	3	819	7.52												
High	Q13618	Cullin-3 OS=Homo sapiens OX=9606 GN=CUL3 PE=1 SV=2	5	2	2	88.9	3	768	8.48			1						1			
High	Q9Y2U8	Inner nuclear membrane protein Man1 OS=Homo sapiens OX=9606 GN=LEMD3 PE=1 SV=2	5	2	2	99.9	5	911	7.55					1						1	
High	P16435	NADPH--cytochrome P450 reductase OS=Homo sapiens OX=9606 GN=POR PE=1 SV=2	5	1	1	76.6	1	677	5.58	1	1	1				1	1	1			
High	Q9BXV9	EKC/KEOPS complex subunit GON7 OS=Homo sapiens OX=9606 GN=GON7 PE=1 SV=2	5	2	2	10.9	41	100	4.27	1	1	1				1	1	1			
High	Q5T0W9	Protein FAM83B OS=Homo sapiens OX=9606 GN=FAM83B PE=1 SV=1	5	3	3	114.7	4	1011	8.97	3	2					3	2				
High	P07196	Neurofilament light polypeptide OS=Homo sapiens OX=9606 GN=NEFL PE=1 SV=3	5	2	1	61.5	3	543	4.65				2	2	1				2	2	1
High	Q9UHA4	Ragulator complex protein LAMTOR3 OS=Homo sapiens OX=9606 GN=LAMTOR3 PE=1 SV=1	5	2	2	13.6	27	124	7.34		2	1					2	1			
High	P14735	Insulin-degrading enzyme OS=Homo sapiens OX=9606 GN=IDE PE=1 SV=4	5	2	2	117.9	2	1019	6.61			1			1			1			1
High	O00750	Phosphatidylinositol 4-phosphate 3-kinase C2 domain-containing subunit beta OS=Homo sapiens OX=9606 GN=PIK3C2B PE=1 SV=	5	2	2	184.7	1	1634	7.3			1		1				1		1	
High	O15357	Phosphatidylinositol 3,4,5-trisphosphate 5-phosphatase 2 OS=Homo sapiens OX=9606 GN=INPPL1 PE=1 SV=2	5	3	3	138.5	3	1258	6.54		1	1	1				1	1	1		
High	Q9Y3S1	Serine/threonine-protein kinase WNK2 OS=Homo sapiens OX=9606 GN=WNK2 PE=1 SV=4	5	2	2	242.5	1	2297	6.13				1						1		
High	P08651	Nuclear factor 1 C-type OS=Homo sapiens OX=9606 GN=NFIC PE=1 SV=2	5	2	1	55.6	6	508	8.38				1	1	1				1	1	1
High	Q15311	RalA-binding protein 1 OS=Homo sapiens OX=9606 GN=RALBP1 PE=1 SV=3	5	4	4	76	8	655	5.88	1		1				1		1			
High	Q15024	Exosome complex component RRP42 OS=Homo sapiens OX=9606 GN=EXOSC7 PE=1 SV=3	5	2	2	31.8	11	291	5.19					2						1	
High	Q9Y3C1	Nucleolar protein 16 OS=Homo sapiens OX=9606 GN=NOP16 PE=1 SV=2	5	2	2	21.2	11	178	9.94					1	1					1	1
High	P54760	Ephrin type-B receptor 4 OS=Homo sapiens OX=9606 GN=EPHB4 PE=1 SV=2	5	3	1	108.2	3	987	6.9		1		1		1		1		1		1
High	P56134	ATP synthase subunit f, mitochondrial OS=Homo sapiens OX=9606 GN=ATP5MF PE=1 SV=3	5	2	2	10.9	26	94	9.67	2	2	1				2	2	1			
High	Q9Y312	Protein AAR2 homolog OS=Homo sapiens OX=9606 GN=AAR2 PE=1 SV=2	5	2	2	43.4	9	384	5.96	1	2	1				1	2	1			
High	P12273	Prolactin-inducible protein OS=Homo sapiens OX=9606 GN=PIP PE=1 SV=1	5	2	2	16.6	13	146	8.05				2	2	1				2	2	1
High	Q5D862	SWISS-PROT:Q5D862 Tax_Id=9606 Gene_Symbol=FLG2 Filaggrin-2	5	4	4	247.9	2	2391	8.31		1	3		1			1	3		1	
High	Q9NX14	NADH dehydrogenase [ubiquinone] 1 beta subcomplex subunit 11, mitochondrial OS=Homo sapiens OX=9606 GN=NDUFB11 PE=1	5	1	1	17.3	7	153	5.22	1	1					1	1				
High	Q15404	Ras suppressor protein 1 OS=Homo sapiens OX=9606 GN=RSU1 PE=1 SV=3	5	2	2	31.5	6	277	8.65			1						1			
High	O60825	6-phosphofructo-2-kinase/fructose-2,6-bisphosphatase 2 OS=Homo sapiens OX=9606 GN=PFKFB2 PE=1 SV=2	5	3	1	58.4	6	505	8.38					1						1	
High	P50502	Hsc70-interacting protein OS=Homo sapiens OX=9606 GN=ST13 PE=1 SV=2	5	2	2	41.3	4	369	5.27			2	1		1			2	1		1
High	Q9P2D1	Chromodomain-helicase-DNA-binding protein 7 OS=Homo sapiens OX=9606 GN=CHD7 PE=1 SV=3	5	3	2	335.7	1	2997	6.34	2	1	2				2	1	2			
High	O96005	Cleft lip and palate transmembrane protein 1 OS=Homo sapiens OX=9606 GN=CLPTM1 PE=1 SV=1	5	3	3	76	3	669	6.3	2	1	1				2	1	1			
High	Q9H9T3	Elongator complex protein 3 OS=Homo sapiens OX=9606 GN=ELP3 PE=1 SV=2	5	3	3	62.2	8	547	8.88	1	1				1	1	1				1
High	Q8IWA0	WD repeat-containing protein 75 OS=Homo sapiens OX=9606 GN=WDR75 PE=1 SV=1	5	1	1	94.4	1	830	5.96		1	1					1	1			
High	Q8TAA5	GrpE protein homolog 2, mitochondrial OS=Homo sapiens OX=9606 GN=GRPEL2 PE=1 SV=1	5	3	3	25.4	12	225	7.72	1	1					1	1				
High	Q9BW91	ADP-ribose pyrophosphatase, mitochondrial OS=Homo sapiens OX=9606 GN=NUDT9 PE=1 SV=1	5	1	1	39.1	2	350	8.22			1		1				1		1	
High	Q99442	Translocation protein SEC62 OS=Homo sapiens OX=9606 GN=SEC62 PE=1 SV=1	5	3	3	45.8	8	399	7.12												
High	Q9BVT8	Transmembrane and ubiquitin-like domain-containing protein 1 OS=Homo sapiens OX=9606 GN=TMUB1 PE=1 SV=1	5	2	2	26.2	9	246	5.72			1						1			
High	O75616	GTPase Era, mitochondrial OS=Homo sapiens OX=9606 GN=ERAL1 PE=1 SV=2	5	1	1	48.3	3	437	8.84			1		1				1		1	
High	O60229	Kalirin OS=Homo sapiens OX=9606 GN=KALRN PE=1 SV=3	5	3	3	340	1	2986	6.07			1						1			
High	O00411	DNA-directed RNA polymerase, mitochondrial OS=Homo sapiens OX=9606 GN=POLRMT PE=1 SV=2	5	2	2	138.5	1	1230	8.98				1						1		
High	Q8WUW1	Protein BRICK1 OS=Homo sapiens OX=9606 GN=BRK1 PE=1 SV=1	5	1	1	8.7	17	75	5.45			2						1			
High	Q9NX57	Ras-related protein Rab-20 OS=Homo sapiens OX=9606 GN=RAB20 PE=1 SV=1	5	3	3	26.3	14	234	6.55	1						1					
High	Q9C0B9	Zinc finger CCHC domain-containing protein 2 OS=Homo sapiens OX=9606 GN=ZCCHC2 PE=1 SV=6	5	4	4	125.9	5	1178	7.01				2	1	2				2	1	2
High	Q9H2M9	Rab3 GTPase-activating protein non-catalytic subunit OS=Homo sapiens OX=9606 GN=RAB3GAP2 PE=1 SV=1	5	2	2	155.9	2	1393	5.62			1						1			
High	P35754	Glutaredoxin-1 OS=Homo sapiens OX=9606 GN=GLRX PE=1 SV=2	5	2	2	11.8	30	106	8.09			1		1				1		1	
High	Q9GZZ9	Ubiquitin-like modifier-activating enzyme 5 OS=Homo sapiens OX=9606 GN=UBA5 PE=1 SV=1	5	2	2	44.8	8	404	4.84	1	1					1	1				
High	Q86WB0	Nuclear-interacting partner of ALK OS=Homo sapiens OX=9606 GN=ZC3HC1 PE=1 SV=1	5	2	2	55.2	4	502	5.62			1	1					1	1		
High	Q9Y4C1	Lysine-specific demethylase 3A OS=Homo sapiens OX=9606 GN=KDM3A PE=1 SV=4	5	4	4	147.2	5	1321	8.07			1						1			
High	Q9NUQ3	Gamma-taxilin OS=Homo sapiens OX=9606 GN=TXLNG PE=1 SV=2	5	2	2	60.5	3	528	7.52			1	1	1	1			1	1	1	1
High	P11274	Breakpoint cluster region protein OS=Homo sapiens OX=9606 GN=BCR PE=1 SV=2	5	4	3	142.7	4	1271	7.03					1						1	
High	Q9BUL9	Ribonuclease P protein subunit p25 OS=Homo sapiens OX=9606 GN=RPP25 PE=1 SV=1	5	2	2	20.6	11	199	9.61				1	1					1	1	
High	Q86WR7	Proline and serine-rich protein 2 OS=Homo sapiens OX=9606 GN=PROSER2 PE=1 SV=2	5	2	2	45.8	6	435	7.2	1	2	1				1	2	1			

High	Q01415	N-acetylgalactosamine kinase OS=Homo sapiens OX=9606 GN=GALK2 PE=1 SV=1	5	1	1	50.3	2	458	6.61		1	1			1		1	1			1
High	Q9Y365	START domain-containing protein 10 OS=Homo sapiens OX=9606 GN=STARD10 PE=1 SV=2	5	2	2	33	10	291	7.12		1						1				
High	O00203	AP-3 complex subunit beta-1 OS=Homo sapiens OX=9606 GN=AP3B1 PE=1 SV=3	5	2	2	121.2	2	1094	6.04	1	2	1				1	2	1			
High	O94826	Mitochondrial import receptor subunit TOM70 OS=Homo sapiens OX=9606 GN=TOMM70 PE=1 SV=1	5	2	2	67.4	5	608	7.12		1						1				
High	P52735	Guanine nucleotide exchange factor VAV2 OS=Homo sapiens OX=9606 GN=VAV2 PE=1 SV=2	5	2	2	101.2	3	878	7.08	1	1	1				1	1	1			
High	Q96I99	Succinate--CoA ligase [GDP-forming] subunit beta, mitochondrial OS=Homo sapiens OX=9606 GN=SUCLG2 PE=1 SV=2	5	2	2	46.5	5	432	6.39		1	1	1				1	1	1		
High	Q8WW01	tRNA-splicing endonuclease subunit Sen15 OS=Homo sapiens OX=9606 GN=TSEN15 PE=1 SV=1	5	3	3	18.6	33	171	4.58						1						1
High	A4D161	Protein FAM221A OS=Homo sapiens OX=9606 GN=FAM221A PE=1 SV=1	5	2	2	33.1	8	298	6.74			1						1			
High	Q96L92	Sorting nexin-27 OS=Homo sapiens OX=9606 GN=SNX27 PE=1 SV=2	5	1	1	61.2	2	541	6.49			1	1					1	1		
High	Q9NP92	39S ribosomal protein S30, mitochondrial OS=Homo sapiens OX=9606 GN=MRPS30 PE=1 SV=2	5	3	3	50.3	15	439	7.97	1	1			1		1	1			1	
High	Q9H4A6	Golgi phosphoprotein 3 OS=Homo sapiens OX=9606 GN=GOLPH3 PE=1 SV=1	5	3	3	33.8	17	298	6.44	1		1	1			1		1	1		
High	Q9C0C9	(E3-independent) E2 ubiquitin-conjugating enzyme OS=Homo sapiens OX=9606 GN=UBE2O PE=1 SV=3	5	2	2	141.2	1	1292	5.12	1		1				1		1			
High	Q6GMV2	Histone-lysine N-trimethyltransferase SMYD5 OS=Homo sapiens OX=9606 GN=SMYD5 PE=1 SV=2	5	1	1	47.3	3	418	5.05			1		1				1		1	
High	Q6P1N0	Coiled-coil and C2 domain-containing protein 1A OS=Homo sapiens OX=9606 GN=CC2D1A PE=1 SV=1	5	2	2	104	3	951	8.09												
High	P02751	Fibronectin OS=Homo sapiens OX=9606 GN=FN1 PE=1 SV=5	5	2	2	272.2	1	2477	5.5				1		1				1		1
High	Q15392	Delta(24)-sterol reductase OS=Homo sapiens OX=9606 GN=DHCR24 PE=1 SV=2	5	2	2	60.1	5	516	8.16	1	1	1				1	1	1			
High	O75821	Eukaryotic translation initiation factor 3 subunit G OS=Homo sapiens OX=9606 GN=EIF3G PE=1 SV=2	5	2	2	35.6	10	320	6.13	2	2	1				2	2	1			
High	Q9NP97	Dynein light chain roadblock-type 1 OS=Homo sapiens OX=9606 GN=DYNLRB1 PE=1 SV=3	5	2	2	10.9	34	96	7.25	1	1	1				1	1	1			
High	Q9HAU5	Regulator of nonsense transcripts 2 OS=Homo sapiens OX=9606 GN=UPF2 PE=1 SV=1	5	4	4	147.7	4	1272	5.69			1	2					1	2		
High	Q7L3T8	Probable proline--tRNA ligase, mitochondrial OS=Homo sapiens OX=9606 GN=PARS2 PE=1 SV=1	5	3	3	53.2	8	475	8.1												
High	Q7Z2Z2	Elongation factor-like GTPase 1 OS=Homo sapiens OX=9606 GN=EFL1 PE=1 SV=2	5	2	2	125.4	3	1120	5.91			1		1				1		1	
High	O15162	Phospholipid scramblase 1 OS=Homo sapiens OX=9606 GN=PLSCR1 PE=1 SV=1	5	2	2	35	7	318	4.94		1						1				
High	Q969X5	Endoplasmic reticulum-Golgi intermediate compartment protein 1 OS=Homo sapiens OX=9606 GN=ERGIC1 PE=1 SV=1	5	2	2	32.6	6	290	7.06					1						1	
High	P21953	2-oxoisovalerate dehydrogenase subunit beta, mitochondrial OS=Homo sapiens OX=9606 GN=BCKDHB PE=1 SV=2	5	4	4	43.1	14	392	6.29			1						1			
High	Q9BV86	N-terminal Xaa-Pro-Lys N-methyltransferase 1 OS=Homo sapiens OX=9606 GN=NTMT1 PE=1 SV=3	5	1	1	25.4	7	223	5.52					1	1					1	1
High	O60934	Nibrin OS=Homo sapiens OX=9606 GN=NBN PE=1 SV=1	5	4	4	84.9	7	754	6.9			1						1			
High	Q9UGP4	LIM domain-containing protein 1 OS=Homo sapiens OX=9606 GN=LIMD1 PE=1 SV=1	5	2	2	72.1	5	676	6.65						1						1
High	Q8WXX5	DnaJ homolog subfamily C member 9 OS=Homo sapiens OX=9606 GN=DNAJC9 PE=1 SV=1	5	3	3	29.9	13	260	5.73					1	1					1	1
High	Q96H55	Unconventional myosin-XIX OS=Homo sapiens OX=9606 GN=MYO19 PE=1 SV=2	5	2	2	109.1	2	970	7.71			2	1					2	1		
High	P83111	Serine beta-lactamase-like protein LACTB, mitochondrial OS=Homo sapiens OX=9606 GN=LACTB PE=1 SV=2	5	2	2	60.7	3	547	8.53			1						1			
High	Q9Y287	Integral membrane protein 2B OS=Homo sapiens OX=9606 GN=ITM2B PE=1 SV=1	5	2	2	30.3	14	266	5.14	1	1	1				1	1	1			
High	Q9Y3E5	Peptidyl-tRNA hydrolase 2, mitochondrial OS=Homo sapiens OX=9606 GN=PTRH2 PE=1 SV=1	5	2	2	19.2	21	179	8.73	1					1	1					1
High	Q9BVG4	Protein PBDC1 OS=Homo sapiens OX=9606 GN=PBDC1 PE=1 SV=1	5	1	1	26	4	233	4.79			1		1				1		1	
High	Q16625	Occludin OS=Homo sapiens OX=9606 GN=OCLN PE=1 SV=1	5	1	1	59.1	4	522	6.09	1		1				1		1			
High	Q15459	Splicing factor 3A subunit 1 OS=Homo sapiens OX=9606 GN=SF3A1 PE=1 SV=1	5	3	3	88.8	6	793	5.22	1					2	1					2
High	Q9GZT3	SRA stem-loop-interacting RNA-binding protein, mitochondrial OS=Homo sapiens OX=9606 GN=SLIRP PE=1 SV=1	5	1	1	12.3	13	109	10.24			1		1				1		1	
High	C9J798	Ras GTPase-activating protein 4B OS=Homo sapiens OX=9606 GN=RASA4B PE=3 SV=2	5	4	4	90.4	7	803	7.74		1						1				
High	Q5T2T1	MAGUK p55 subfamily member 7 OS=Homo sapiens OX=9606 GN=MPP7 PE=1 SV=1	5	2	2	65.5	5	576	7.14	1	1	1				1	1	1			
High	Q96PZ0	Pseudouridylate synthase 7 homolog OS=Homo sapiens OX=9606 GN=PUS7 PE=1 SV=2	5	4	4	75	12	661	6.37			1						1			
High	P22307	Sterol carrier protein 2 OS=Homo sapiens OX=9606 GN=SCP2 PE=1 SV=2	5	1	1	59	1	547	6.89			1		1				1		1	
High	P08574	Cytochrome c1, heme protein, mitochondrial OS=Homo sapiens OX=9606 GN=CYC1 PE=1 SV=3	5	2	2	35.4	10	325	9	2	1	1				2	1	1			
High	Q9UK45	U6 snRNA-associated Sm-like protein LSm7 OS=Homo sapiens OX=9606 GN=LSM7 PE=1 SV=1	5	2	2	11.6	33	103	5.27					1						1	
High	O95340	Bifunctional 3'-phosphoadenosine 5'-phosphosulfate synthase 2 OS=Homo sapiens OX=9606 GN=PAPSS2 PE=1 SV=2	5	2	2	69.5	5	614	8.03			1		1				1		1	
High	O95365	Zinc finger and BTB domain-containing protein 7A OS=Homo sapiens OX=9606 GN=ZBTB7A PE=1 SV=1	5	2	2	61.4	6	584	5.19	1	1					1	1				
High	O15270	Serine palmitoyltransferase 2 OS=Homo sapiens OX=9606 GN=SPTLC2 PE=1 SV=1	5	2	2	62.9	4	562	7.78					2						2	
High	Q8TEW0	Partitioning defective 3 homolog OS=Homo sapiens OX=9606 GN=PARD3 PE=1 SV=2	5	4	4	151.3	3	1356	7.68	3						3					
High	P40937	Replication factor C subunit 5 OS=Homo sapiens OX=9606 GN=RFC5 PE=1 SV=1	5	2	2	38.5	10	340	7.2			1						1			
High	Q9Y294	Histone chaperone ASF1A OS=Homo sapiens OX=9606 GN=ASF1A PE=1 SV=1	5	2	2	23	9	204	4.41		1			2	1		1			2	1
High	Q13601	KRR1 small subunit processome component homolog OS=Homo sapiens OX=9606 GN=KRR1 PE=1 SV=4	5	3	3	43.6	7	381	9.77					2						2	
High	P49407	Beta-arrestin-1 OS=Homo sapiens OX=9606 GN=ARRB1 PE=1 SV=2	5	3	3	47	9	418	6.2			1						1			
High	Q8WUP2	Filamin-binding LIM protein 1 OS=Homo sapiens OX=9606 GN=FBLIM1 PE=1 SV=2	5	2	2	40.6	9	373	6.02				1		1				1		1
High	Q9UJY4	ADP-ribosylation factor-binding protein GGA2 OS=Homo sapiens OX=9606 GN=GGA2 PE=1 SV=3	5	3	3	67.1	8	613	6.55		1						1				
High	P15090	Fatty acid-binding protein, adipocyte OS=Homo sapiens OX=9606 GN=FABP4 PE=1 SV=3	5	2	2	14.7	16	132	7.14				2	1	2				2	1	2
High	O43847	Nardilysin OS=Homo sapiens OX=9606 GN=NRDC PE=1 SV=3	5	3	3	131.6	3	1151	4.98			1		2				1		2	
High	O95453	Poly(A)-specific ribonuclease PARN OS=Homo sapiens OX=9606 GN=PARN PE=1 SV=1	5	2	2	73.4	3	639	6.2				1		1				1		1
High	O14733	Dual specificity mitogen-activated protein kinase kinase 7 OS=Homo sapiens OX=9606 GN=MAP2K7 PE=1 SV=2	5	2	2	47.5	6	419	9.16	2	1			1		2	1			1	
High	Q6UWE0	E3 ubiquitin-protein ligase LRSAM1 OS=Homo sapiens OX=9606 GN=LRSAM1 PE=1 SV=1	5	2	2	83.5	4	723	5.94			1						1			
High	O95858	Tetraspanin-15 OS=Homo sapiens OX=9606 GN=TSPAN15 PE=1 SV=1	5	1	1	33.1	3	294	5.48		1	1					1	1			
High	Q9H299	SH3 domain-binding glutamic acid-rich-like protein 3 OS=Homo sapiens OX=9606 GN=SH3BGRL3 PE=1 SV=1	5	1	1	10.4	11	93	4.93	1	1	1	1		1	1	1	1	1		1
High	P17026	Zinc finger protein 22 OS=Homo sapiens OX=9606 GN=ZNF22 PE=1 SV=3	5	2	2	25.9	8	224	10.04												
High	P04181	Ornithine aminotransferase, mitochondrial OS=Homo sapiens OX=9606 GN=OAT PE=1 SV=1	5	1	1	48.5	2	439	7.03		1	1			1		1	1			1
High	Q86XZ4	Spermatogenesis-associated serine-rich protein 2 OS=Homo sapiens OX=9606 GN=SPATS2 PE=1 SV=1	5	3	3	59.5	10	545	8.9						1						1
High	Q15555	Microtubule-associated protein RP/EB family member 2 OS=Homo sapiens OX=9606 GN=MAPRE2 PE=1 SV=1	5	2	2	37	7	327	5.57	1	1	1		1		1	1	1		1	
High	P61960	Ubiquitin-fold modifier 1 OS=Homo sapiens OX=9606 GN=UFM1 PE=1 SV=1	5	2	2	9.1	27	85	9.31				1	2					1	2	
High	Q9H6S0	3'-5' RNA helicase YTHDC2 OS=Homo sapiens OX=9606 GN=YTHDC2 PE=1 SV=2	5	3	3	160.1	2	1430	8.4												
High	Q93099	Homogentisate 1,2-dioxygenase OS=Homo sapiens OX=9606 GN=HGD PE=1 SV=2	5	3	3	49.9	13	445	6.96		1						1				
High	Q9NPJ3	Acyl-coenzyme A thioesterase 13 OS=Homo sapiens OX=9606 GN=ACOT13 PE=1 SV=1	5	1	1	15	7	140	9.14					1	1					1	1
High	Q9H4A5	Golgi phosphoprotein 3-like OS=Homo sapiens OX=9606 GN=GOLPH3L PE=1 SV=1	5	1	1	32.7	6	285	5.83	1	1	1				1	1	1			
High	Q9Y597	BTB/POZ domain-containing protein KCTD3 OS=Homo sapiens OX=9606 GN=KCTD3 PE=1 SV=2	5	2	1	88.9	3	815	7.03					1						1	
High	Q15653	NF-kappa-B inhibitor beta OS=Homo sapiens OX=9606 GN=NFKBIB PE=1 SV=2	5	1	1	37.7	4	356	4.84	1		1				1		1			
High	O75832	26S proteasome non-ATPase regulatory subunit 10 OS=Homo sapiens OX=9606 GN=PSMD10 PE=1 SV=1	5	3	3	24.4	16	226	6.1				1	1	1				1	1	1
High	Q9Y5Q8	General transcription factor 3C polypeptide 5 OS=Homo sapiens OX=9606 GN=GTF3C5 PE=1 SV=2	5	1	1	59.5	2	519	6.9				1	1	1				1	1	1
High	Q96A35	39S ribosomal protein L24, mitochondrial OS=Homo sapiens OX=9606 GN=MRPL24 PE=1 SV=1	5	4	4	24.9	23	216	9.29												
High	P25685	DnaJ homolog subfamily B member 1 OS=Homo sapiens OX=9606 GN=DNAJB1 PE=1 SV=4	5	5	4	38	17	340	8.63		1	1					1	1			
High	Q96GX9	Methylthioribulose-1-phosphate dehydratase OS=Homo sapiens OX=9606 GN=APIP PE=1 SV=1	4	1	1	27.1	7	242	7.12	1	1	1				1	1	1			
High	Q7L5A8	Fatty acid 2-hydroxylase OS=Homo sapiens OX=9606 GN=FA2H PE=1 SV=1	4	2	2	42.8	6	372	8.66	1	1	1				1	1	1			
High	P31751	RAC-beta serine/threonine-protein kinase OS=Homo sapiens OX=9606 GN=AKT2 PE=1 SV=2	4	2	2	55.7	6	481	6.37			1						1			
High	Q12979	Active breakpoint cluster region-related protein OS=Homo sapiens OX=9606 GN=ABR PE=1 SV=2	4	2	1	97.5	3	859	6.55			1		1				1		1	
High	Q4G0F5	Vacuolar protein sorting-associated protein 26B OS=Homo sapiens OX=9606 GN=VPS26B PE=1 SV=2	4	2	1	39.1	8	336	7.36		1			1			1			1	
High	Q9BU68	Proline-rich protein 15-like protein OS=Homo sapiens OX=9606 GN=PRR15L PE=3 SV=1	4	1	1	11.7	17	103	9.5	1	1	1				1	1	1			
High	Q9BVS4	Serine/threonine-protein kinase RIO2 OS=Homo sapiens OX=9606 GN=RIOK2 PE=1 SV=2	4	1	1	63.2	4	552	5.94			1						1			
High	Q9BVC4	Target of rapamycin complex subunit LST8 OS=Homo sapiens OX=9606 GN=MLST8 PE=1 SV=1	4	2	2	35.9	12	326	5.86		1						1				
High	O15530	3-phosphoinositide-dependent protein kinase 1 OS=Homo sapiens OX=9606 GN=PDPK1 PE=1 SV=1	4	2	2	63.1	5	556	7.36	1	1	1				1	1	1			
High	P48739	Phosphatidylinositol transfer protein beta isoform OS=Homo sapiens OX=9606 GN=PITPNB PE=1 SV=2	4	3	3	31.5	13	271	6.87				1		1				1		1
High	Q96IW7	Vesicle-trafficking protein SEC22a OS=Homo sapiens OX=9606 GN=SEC22A PE=1 SV=1	4	1	1	34.9	2	307	8.24	1		1				1		1			
High	P49441	Inositol polyphosphate 1-phosphatase OS=Homo sapiens OX=9606 GN=INPP1 PE=1 SV=1	4	2	2	44	9	399	5.26	1	1	1				1	1	1			
High	O00267	Transcription elongation factor SPT5 OS=Homo sapiens OX=9606 GN=SUPT5H PE=1 SV=1	4	2	2	120.9	3	1087	5.06	1				1		1				1	
High	P48059	LIM and senescent cell antigen-like-containing domain protein 1 OS=Homo sapiens OX=9606 GN=LIMS1 PE=1 SV=4	4	3	3	37.2	9	325	8.05												
High	Q9NQT5	Exosome complex component RRP40 OS=Homo sapiens OX=9606 GN=EXOSC3 PE=1 SV=3	4	3	3	29.6	12	275	8.1												
High	Q9NV35	Nucleotide triphosphate diphosphatase NUDT15 OS=Homo sapiens OX=9606 GN=NUDT15 PE=1 SV=1	4	1	1	18.6	7	164	6.14			1	1		1			1	1		1
High	Q13445	Transmembrane emp24 domain-containing protein 1 OS=Homo sapiens OX=9606 GN=TMED1 PE=1 SV=1	4	1	1	25.2	7	227	4.48	1	1	1				1	1	1			
High	O75964	ATP synthase subunit g, mitochondrial OS=Homo sapiens OX=9606 GN=ATP5MG PE=1 SV=3	4	1	1	11.4	11	103	9.64		1	1					1	1			
High	P16220	Cyclic AMP-responsive element-binding protein 1 OS=Homo sapiens OX=9606 GN=CREB1 PE=1 SV=3	4	2	2	35.1	12	327	5.27				2	1	1				2	1	1
High	P01857	Immunoglobulin heavy constant gamma 1 OS=Homo sapiens OX=9606 GN=IGHG1 PE=1 SV=1	4	2	1	36.1	7	330	8.19				1	1	2				1	1	2
High	Q96Q11	CCA tRNA nucleotidyltransferase 1, mitochondrial OS=Homo sapiens OX=9606 GN=TRNT1 PE=1 SV=2	4	2	2	50.1	6	434	8.1			1			1			1			1
High	Q08188	Protein-glutamine gamma-glutamyltransferase E OS=Homo sapiens OX=9606 GN=TGM3 PE=1 SV=4	4	3	3	76.6	3	693	5.86				2	1	1				2	1	1
High	Q5JPH6	Probable glutamate--tRNA ligase, mitochondrial OS=Homo sapiens OX=9606 GN=EARS2 PE=1 SV=2	4	2	2	58.7	5	523	8.76	1	1	1				1	1	1			
High	P61201	COP9 signalosome complex subunit 2 OS=Homo sapiens OX=9606 GN=COPS2 PE=1 SV=1	4	1	1	51.6	6	443	5.53			1						1			
High	O75689	Arf-GAP with dual PH domain-containing protein 1 OS=Homo sapiens OX=9606 GN=ADAP1 PE=1 SV=2	4	2	2	43.4	7	374	8.97			1	1		1			1	1		1
High	Q16775	Hydroxyacylglutathione hydrolase, mitochondrial OS=Homo sapiens OX=9606 GN=HAGH PE=1 SV=2	4	1	1	33.8	5	308	8.12			1						1			
High	O94819	Kelch repeat and BTB domain-containing protein 11 OS=Homo sapiens OX=9606 GN=KBTBD11 PE=1 SV=1	4	2	2	65.7	4	623	6.07												
High	P13798	Acylamino-acid-releasing enzyme OS=Homo sapiens OX=9606 GN=APEH PE=1 SV=4	4	2	2	81.2	4	732	5.48	1	1	1		1		1	1	1		1	
High	Q7L5N1	COP9 signalosome complex subunit 6 OS=Homo sapiens OX=9606 GN=COPS6 PE=1 SV=1	4	3	3	36.1	12	327	5.73					1						1	
High	Q68DH5	G-protein coupled receptor-associated protein LMBRD2 OS=Homo sapiens OX=9606 GN=LMBRD2 PE=1 SV=1	4	3	3	81.1	6	695	7.5	2	1	1				2	1	1			
High	Q5W0U4	B box and SPRY domain-containing protein OS=Homo sapiens OX=9606 GN=BSPRY PE=2 SV=1	4	2	2	44.4	5	402	6.42				1		1				1		1
High	Q9Y371	Endophilin-B1 OS=Homo sapiens OX=9606 GN=SH3GLB1 PE=1 SV=1	4	2	2	40.8	6	365	6.04		1						1				
High	Q15629	Translocating chain-associated membrane protein 1 OS=Homo sapiens OX=9606 GN=TRAM1 PE=1 SV=3	4	1	1	43	3	374	9.63	1	1	1				1	1	1			
High	O15243	Leptin receptor gene-related protein OS=Homo sapiens OX=9606 GN=LEPROT PE=1 SV=1	4	1	1	14.2	10	131	6.51	1	1					1	1				
High	Q8N884	Cyclic GMP-AMP synthase OS=Homo sapiens OX=9606 GN=CGAS PE=1 SV=2	4	2	2	58.8	4	522	9.48	1	1	2				1	1	2			
High	Q9UNQ0	Broad substrate specificity ATP-binding cassette transporter ABCG2 OS=Homo sapiens OX=9606 GN=ABCG2 PE=1 SV=3	4	3	3	72.3	4	655	8.69			1						1			
High	Q9BSC4	Nucleolar protein 10 OS=Homo sapiens OX=9606 GN=NOL10 PE=1 SV=1	4	1	1	80.3	1	688	8.46					1						1	
High	Q9UL15	BAG family molecular chaperone regulator 5 OS=Homo sapiens OX=9606 GN=BAG5 PE=1 SV=1	4	2	2	51.2	5	447	6.05	1	2				1	1	2				1
High	P05413	Fatty acid-binding protein, heart OS=Homo sapiens OX=9606 GN=FABP3 PE=1 SV=4	4	2	2	14.8	12	133	6.8				1	1	2				1	1	2
High	Q13217	DnaJ homolog subfamily C member 3 OS=Homo sapiens OX=9606 GN=DNAJC3 PE=1 SV=1	4	1	1	57.5	3	504	6.15			1						1			
High	Q96DH6	RNA-binding protein Musashi homolog 2 OS=Homo sapiens OX=9606 GN=MSI2 PE=1 SV=1	4	3	3	35.2	12	328	8.48												
High	Q05443	SWISS-PROT:Q05443 (Bos taurus) Lumican precursor	4	3	3	38.7	8	342	6.35				1	1	2				1	1	2
High	P30043	Flavin reductase (NADPH) OS=Homo sapiens OX=9606 GN=BLVRB PE=1 SV=3	4	1	1	22.1	5	206	7.65	1						1					
High	P63272	Transcription elongation factor SPT4 OS=Homo sapiens OX=9606 GN=SUPT4H1 PE=1 SV=1	4	1	1	13.2	6	117	8.06					1	1					1	1
High	P29375	Lysine-specific demethylase 5A OS=Homo sapiens OX=9606 GN=KDM5A PE=1 SV=3	4	2	1	192	2	1690	6.49												
High	O75528	Transcriptional adapter 3 OS=Homo sapiens OX=9606 GN=TADA3 PE=1 SV=1	4	1	1	48.9	5	432	6.27					1	1					1	1
High	O15460	Prolyl 4-hydroxylase subunit alpha-2 OS=Homo sapiens OX=9606 GN=P4HA2 PE=1 SV=1	4	4	4	60.9	10	535	5.71			2						2			
High	Q8IUD2	ELKS/Rab6-interacting/CAST family member 1 OS=Homo sapiens OX=9606 GN=ERC1 PE=1 SV=1	4	3	3	128	3	1116	5.97			3						3			
High	Q8TC07	TBC1 domain family member 15 OS=Homo sapiens OX=9606 GN=TBC1D15 PE=1 SV=2	4	3	3	79.4	4	691	5.67		2						2				
High	P55198	Protein AF-17 OS=Homo sapiens OX=9606 GN=MLLT6 PE=1 SV=3	4	1	1	112	1	1093	8.62				1		1				1		1
High	Q8IZX4	Transcription initiation factor TFIID subunit 1-like OS=Homo sapiens OX=9606 GN=TAF1L PE=1 SV=1	4	2	2	207.2	1	1826	5.4												
High	P48147	Prolyl endopeptidase OS=Homo sapiens OX=9606 GN=PREP PE=1 SV=2	4	3	3	80.6	6	710	5.86	1	1					1	1				
High	Q9Y4Z0	U6 snRNA-associated Sm-like protein LSm4 OS=Homo sapiens OX=9606 GN=LSM4 PE=1 SV=1	4	1	1	15.3	7	139	9.99					1						1	
High	Q5VYS8	Terminal uridylyltransferase 7 OS=Homo sapiens OX=9606 GN=TUT7 PE=1 SV=1	4	2	2	171.1	2	1495	6.83					1						1	
High	Q9H8N7	Zinc finger protein 395 OS=Homo sapiens OX=9606 GN=ZNF395 PE=1 SV=2	4	1	1	54.9	4	513	7.43			1						1			
High	P20591	Interferon-induced GTP-binding protein Mx1 OS=Homo sapiens OX=9606 GN=MX1 PE=1 SV=4	4	2	2	75.5	5	662	5.83				1	1	2				1	1	2
High	Q96HP4	Oxidoreductase NAD-binding domain-containing protein 1 OS=Homo sapiens OX=9606 GN=OXNAD1 PE=1 SV=1	4	2	2	34.8	6	312	8.37		1						1				
High	P49755	Transmembrane emp24 domain-containing protein 10 OS=Homo sapiens OX=9606 GN=TMED10 PE=1 SV=2	4	3	3	25	19	219	7.44	1	1	1		1		1	1	1		1	
High	Q9BXM0	Periaxin OS=Homo sapiens OX=9606 GN=PRX PE=1 SV=2	4	2	2	154.8	1	1461	7.5				1	2	1				1	2	1
High	Q9H019	Mitochondrial fission regulator 1-like OS=Homo sapiens OX=9606 GN=MTFR1L PE=1 SV=2	4	2	2	31.9	11	292	6.1	1	1	1				1	1	1			
High	Q92766	Ras-responsive element-binding protein 1 OS=Homo sapiens OX=9606 GN=RREB1 PE=1 SV=3	4	2	2	181.3	2	1687	6.98			1						1			
High	Q9NVZ3	Adaptin ear-binding coat-associated protein 2 OS=Homo sapiens OX=9606 GN=NECAP2 PE=1 SV=1	4	2	2	28.3	13	263	8.38	1		1		1		1		1		1	
High	Q96BN8	Ubiquitin thioesterase otulin OS=Homo sapiens OX=9606 GN=OTULIN PE=1 SV=3	4	2	2	40.2	5	352	5.47			1						1			
High	Q9BRR6	ADP-dependent glucokinase OS=Homo sapiens OX=9606 GN=ADPGK PE=1 SV=1	4	1	1	54.1	5	497	6.2	1	1					1	1				
High	P68402	Platelet-activating factor acetylhydrolase IB subunit alpha2 OS=Homo sapiens OX=9606 GN=PAFAH1B2 PE=1 SV=1	4	1	1	25.6	8	229	5.92	1	2	1				1	1	1			
High	Q92688	Acidic leucine-rich nuclear phosphoprotein 32 family member B OS=Homo sapiens OX=9606 GN=ANP32B PE=1 SV=1	4	3	3	28.8	14	251	4.06		1			1			1			1	
High	Q16637	Survival motor neuron protein OS=Homo sapiens OX=9606 GN=SMN1 PE=1 SV=1	4	2	2	31.8	10	294	6.55				1	1	1				1	1	1
High	Q9Y2P8	RNA 3'-terminal phosphate cyclase-like protein OS=Homo sapiens OX=9606 GN=RCL1 PE=1 SV=3	4	2	2	40.8	7	373	9.26												
High	Q9NXJ5	Pyroglutamyl-peptidase 1 OS=Homo sapiens OX=9606 GN=PGPEP1 PE=1 SV=1	4	2	2	23.1	13	209	5.9	1	1	1				1	1	1			
High	Q8TER5	Rho guanine nucleotide exchange factor 40 OS=Homo sapiens OX=9606 GN=ARHGEF40 PE=1 SV=3	4	3	3	164.6	3	1519	6.15												
High	Q8NHH9	Atlastin-2 OS=Homo sapiens OX=9606 GN=ATL2 PE=1 SV=2	4	2	2	66.2	7	583	5.48		1	1					1	1			
High	Q96NL6	Sodium channel and clathrin linker 1 OS=Homo sapiens OX=9606 GN=SCLT1 PE=1 SV=2	4	2	2	80.9	2	688	6.07	1	1	2				1	1	2			
High	Q8IYT2	Cap-specific mRNA (nucleoside-2'-O-)-methyltransferase 2 OS=Homo sapiens OX=9606 GN=CMTR2 PE=1 SV=2	4	1	1	88.1	1	770	7.01	1	1					1	1				
High	P61769	Beta-2-microglobulin OS=Homo sapiens OX=9606 GN=B2M PE=1 SV=1	4	2	2	13.7	27	119	6.52	1	2	1				1	2	1			
High	Q6P1J9	Parafibromin OS=Homo sapiens OX=9606 GN=CDC73 PE=1 SV=1	4	3	3	60.5	6	531	9.61					1						1	
High	Q96RN5	Mediator of RNA polymerase II transcription subunit 15 OS=Homo sapiens OX=9606 GN=MED15 PE=1 SV=2	4	1	1	86.7	3	788	9.42			1						1			
High	P25686	DnaJ homolog subfamily B member 2 OS=Homo sapiens OX=9606 GN=DNAJB2 PE=1 SV=3	4	2	1	35.6	8	324	5.95	1	1	2				1	1	2			
High	Q7Z589	BRCA2-interacting transcriptional repressor EMSY OS=Homo sapiens OX=9606 GN=EMSY PE=1 SV=2	4	2	2	141.4	2	1322	9.33					1						1	
High	Q5JSZ5	Protein PRRC2B OS=Homo sapiens OX=9606 GN=PRRC2B PE=1 SV=2	4	1	1	242.8	1	2229	8.34	1	1	1				1	1	1			
High	Q96JC1	Vam6/Vps39-like protein OS=Homo sapiens OX=9606 GN=VPS39 PE=1 SV=2	4	1	1	101.7	2	886	6.99	1		1				1		1			
High	P12830	Cadherin-1 OS=Homo sapiens OX=9606 GN=CDH1 PE=1 SV=3	4	1	1	97.4	1	882	4.73			1		1				1		1	
High	P49005	DNA polymerase delta subunit 2 OS=Homo sapiens OX=9606 GN=POLD2 PE=1 SV=1	4	2	2	51.3	5	469	5.58												
High	P12955	Xaa-Pro dipeptidase OS=Homo sapiens OX=9606 GN=PEPD PE=1 SV=3	4	2	2	54.5	3	493	6				1	2	1				1	2	1
High	Q9NWZ8	Gem-associated protein 8 OS=Homo sapiens OX=9606 GN=GEMIN8 PE=1 SV=1	4	2	2	28.6	12	242	6.8					2						2	
High	Q8N7H5	RNA polymerase II-associated factor 1 homolog OS=Homo sapiens OX=9606 GN=PAF1 PE=1 SV=2	4	2	2	59.9	5	531	4.63												
High	O60293	Zinc finger C3H1 domain-containing protein OS=Homo sapiens OX=9606 GN=ZFC3H1 PE=1 SV=3	4	3	3	226.2	2	1989	8.13												
High	Q15269	Periodic tryptophan protein 2 homolog OS=Homo sapiens OX=9606 GN=PWP2 PE=1 SV=2	4	1	1	102.4	1	919	6.15					1						1	
High	Q8N5N7	39S ribosomal protein L50, mitochondrial OS=Homo sapiens OX=9606 GN=MRPL50 PE=1 SV=2	4	2	2	18.3	22	158	7.88	1						1					
High	Q9NQW7	Xaa-Pro aminopeptidase 1 OS=Homo sapiens OX=9606 GN=XPNPEP1 PE=1 SV=3	4	1	1	69.9	1	623	5.67	1	1	1				1	1	1			
High	Q5T5U3	Rho GTPase-activating protein 21 OS=Homo sapiens OX=9606 GN=ARHGAP21 PE=1 SV=2	4	2	2	217.3	1	1958	7.8			1		1				1		1	
High	Q9BXK1	Krueppel-like factor 16 OS=Homo sapiens OX=9606 GN=KLF16 PE=1 SV=1	4	2	2	25.4	17	252	9.88				1	2	1				1	2	1
High	Q96B26	Exosome complex component RRP43 OS=Homo sapiens OX=9606 GN=EXOSC8 PE=1 SV=1	4	2	2	30	7	276	5.3						1						1
High	O43172	U4/U6 small nuclear ribonucleoprotein Prp4 OS=Homo sapiens OX=9606 GN=PRPF4 PE=1 SV=2	4	2	2	58.4	8	522	7.42												
High	P61923	Coatomer subunit zeta-1 OS=Homo sapiens OX=9606 GN=COPZ1 PE=1 SV=1	4	2	2	20.2	12	177	4.81	2	1					2	1				
High	O43639	Cytoplasmic protein NCK2 OS=Homo sapiens OX=9606 GN=NCK2 PE=1 SV=2	4	1	1	42.9	5	380	6.95	1	1	1				1	1	1			

High	Q15042	Rab3 GTPase-activating protein catalytic subunit OS=Homo sapiens OX=9606 GN=RAB3GAP1 PE=1 SV=3	4	2	2	110.5	2	981	5.55	2	1	1				2	1	1			
High	Q16204	Coiled-coil domain-containing protein 6 OS=Homo sapiens OX=9606 GN=CCDC6 PE=1 SV=2	4	4	4	53.3	9	474	7.34				1						1		
High	O15111	Inhibitor of nuclear factor kappa-B kinase subunit alpha OS=Homo sapiens OX=9606 GN=CHUK PE=1 SV=2	4	1	1	84.6	2	745	6.73	1	1	1				1	1	1			
High	P24928	DNA-directed RNA polymerase II subunit RPB1 OS=Homo sapiens OX=9606 GN=POLR2A PE=1 SV=2	4	2	2	217	2	1970	7.37												
High	Q9NRR3	CDC42 small effector protein 2 OS=Homo sapiens OX=9606 GN=CDC42SE2 PE=1 SV=1	4	1	1	9.2	23	84	8.35												
High	Q8NE86	Calcium uniporter protein, mitochondrial OS=Homo sapiens OX=9606 GN=MCU PE=1 SV=1	4	2	2	39.8	9	351	8.65	1	2	1				1	2	1			
High	Q13409	Cytoplasmic dynein 1 intermediate chain 2 OS=Homo sapiens OX=9606 GN=DYNC1I2 PE=1 SV=3	4	1	1	71.4	2	638	5.2					1	1					1	1
High	Q8NFH5	Nucleoporin NUP35 OS=Homo sapiens OX=9606 GN=NUP35 PE=1 SV=1	4	1	1	34.8	6	326	9.09			1						1			
High	Q01804	OTU domain-containing protein 4 OS=Homo sapiens OX=9606 GN=OTUD4 PE=1 SV=4	4	1	1	124	1	1114	6.71	1	1	1				1	1	1			
High	P11717	Cation-independent mannose-6-phosphate receptor OS=Homo sapiens OX=9606 GN=IGF2R PE=1 SV=3	4	2	2	274.2	1	2491	5.94			1						1			
High	O95674	Phosphatidate cytidylyltransferase 2 OS=Homo sapiens OX=9606 GN=CDS2 PE=1 SV=1	4	1	1	51.4	4	445	7.09	1	1	2				1	1	1			
High	O94818	Nucleolar protein 4 OS=Homo sapiens OX=9606 GN=NOL4 PE=1 SV=2	4	1	1	71.3	1	638	5.62				1	1					1	1	
High	P27986	Phosphatidylinositol 3-kinase regulatory subunit alpha OS=Homo sapiens OX=9606 GN=PIK3R1 PE=1 SV=2	4	3	3	83.5	7	724	6.16			1		1				1		1	
High	P42356	Phosphatidylinositol 4-kinase alpha OS=Homo sapiens OX=9606 GN=PI4KA PE=1 SV=4	4	2	2	236.7	1	2102	7.06	1	2	1				1	2	1			
High	Q93062	RNA-binding protein with multiple splicing OS=Homo sapiens OX=9606 GN=RBPMS PE=1 SV=1	4	2	2	21.8	20	196	8.07	1		1				1		1			
High	Q9NP61	ADP-ribosylation factor GTPase-activating protein 3 OS=Homo sapiens OX=9606 GN=ARFGAP3 PE=1 SV=1	4	2	2	56.9	5	516	7.36		1						1				
High	Q6ZN55	Zinc finger protein 574 OS=Homo sapiens OX=9606 GN=ZNF574 PE=1 SV=2	4	2	2	98.8	3	896	8.07					1						1	
High	Q8IWB7	WD repeat and FYVE domain-containing protein 1 OS=Homo sapiens OX=9606 GN=WDFY1 PE=1 SV=1	4	2	2	46.3	8	410	7.33	1	1	2				1	1	2			
High	Q9H9A5	CCR4-NOT transcription complex subunit 10 OS=Homo sapiens OX=9606 GN=CNOT10 PE=1 SV=1	4	2	2	82.3	3	744	7.78												
High	P42696	RNA-binding protein 34 OS=Homo sapiens OX=9606 GN=RBM34 PE=1 SV=2	4	3	3	48.5	7	430	10.11					1	1					1	1
High	P12429	Annexin A3 OS=Homo sapiens OX=9606 GN=ANXA3 PE=1 SV=3	4	2	2	36.4	5	323	5.92				2	1	1				2	1	1
High	Q9BUK6	Protein misato homolog 1 OS=Homo sapiens OX=9606 GN=MSTO1 PE=1 SV=1	4	3	3	61.8	8	570	6.11			3	1					3	1		
High	P19784	Casein kinase II subunit alpha' OS=Homo sapiens OX=9606 GN=CSNK2A2 PE=1 SV=1	4	3	3	41.2	13	350	8.56			1						1			
High	Q9BRJ2	39S ribosomal protein L45, mitochondrial OS=Homo sapiens OX=9606 GN=MRPL45 PE=1 SV=2	4	1	1	35.3	3	306	9.03					1						1	
High	P01040	Cystatin-A OS=Homo sapiens OX=9606 GN=CSTA PE=1 SV=1	4	3	3	11	38	98	5.5				1	3					1	3	
High	P51572	B-cell receptor-associated protein 31 OS=Homo sapiens OX=9606 GN=BCAP31 PE=1 SV=3	4	2	2	28	7	246	8.44			1						1			
High	P20226	TATA-box-binding protein OS=Homo sapiens OX=9606 GN=TBP PE=1 SV=2	4	2	2	37.7	7	339	9.79	1	2	1				1	2	1			
High	Q9H814	Phosphorylated adapter RNA export protein OS=Homo sapiens OX=9606 GN=PHAX PE=1 SV=1	4	2	2	44.4	8	394	5.4				1	1	1				1	1	1
High	P50548	ETS domain-containing transcription factor ERF OS=Homo sapiens OX=9606 GN=ERF PE=1 SV=2	4	1	1	58.7	4	548	7.3	1	1	1				1	1	1			
High	Q9HA92	Radical S-adenosyl methionine domain-containing protein 1, mitochondrial OS=Homo sapiens OX=9606 GN=RSAD1 PE=1 SV=2	4	2	2	48.7	4	442	7.78												
High	Q86WJ1	Chromodomain-helicase-DNA-binding protein 1-like OS=Homo sapiens OX=9606 GN=CHD1L PE=1 SV=3	4	3	3	100.9	4	897	6.9		1	1					1	1			
High	Q96MU7	YTH domain-containing protein 1 OS=Homo sapiens OX=9606 GN=YTHDC1 PE=1 SV=3	4	1	1	84.6	2	727	6.23			1						1			
High	Q9GZP9	Derlin-2 OS=Homo sapiens OX=9606 GN=DERL2 PE=1 SV=1	4	1	1	27.5	3	239	7.28		1	1	1		1		1	1	1		1
High	Q8WUA7	TBC1 domain family member 22A OS=Homo sapiens OX=9606 GN=TBC1D22A PE=1 SV=2	4	3	3	59.1	7	517	5.97	2	1	1				2	1	1			
High	Q8NHM5	Lysine-specific demethylase 2B OS=Homo sapiens OX=9606 GN=KDM2B PE=1 SV=1	4	2	2	152.5	2	1336	8.56				1	2	1				1	2	1
High	Q16594	Transcription initiation factor TFIID subunit 9 OS=Homo sapiens OX=9606 GN=TAF9 PE=1 SV=1	4	3	3	29	16	264	8.66					1						1	
High	Q8N4J0	Carnosine N-methyltransferase OS=Homo sapiens OX=9606 GN=CARNMT1 PE=1 SV=1	4	1	1	47.2	3	409	6.32					1						1	
High	P82664	28S ribosomal protein S10, mitochondrial OS=Homo sapiens OX=9606 GN=MRPS10 PE=1 SV=2	4	2	2	23	15	201	8												
High	P61225	Ras-related protein Rap-2b OS=Homo sapiens OX=9606 GN=RAP2B PE=1 SV=1	4	2	2	20.5	14	183	4.81												
High	O43395	U4/U6 small nuclear ribonucleoprotein Prp3 OS=Homo sapiens OX=9606 GN=PRPF3 PE=1 SV=2	4	2	2	77.5	6	683	9.5				1						1		
High	Q9NVA2	Septin-11 OS=Homo sapiens OX=9606 GN=SEPTIN11 PE=1 SV=3	4	2	1	49.4	7	429	6.81	1		1	1			1		1	1		
High	O43747	AP-1 complex subunit gamma-1 OS=Homo sapiens OX=9606 GN=AP1G1 PE=1 SV=5	4	2	2	91.3	2	822	6.8		1	1		1			1	1		1	
High	Q9H3P2	Negative elongation factor A OS=Homo sapiens OX=9606 GN=NELFA PE=1 SV=3	4	1	1	57.2	4	528	9.03				1		1				1		1
High	Q9HCK0	Zinc finger and BTB domain-containing protein 26 OS=Homo sapiens OX=9606 GN=ZBTB26 PE=1 SV=2	4	1	1	49.9	4	441	7.08			1						1			
High	P49593	Protein phosphatase 1F OS=Homo sapiens OX=9606 GN=PPM1F PE=1 SV=3	4	1	1	49.8	2	454	5.1		1						1				
High	Q8IWW6	Rho GTPase-activating protein 12 OS=Homo sapiens OX=9606 GN=ARHGAP12 PE=1 SV=1	4	2	2	96.2	4	846	7.61		1	1			1		1	1			1
High	A1X283	SH3 and PX domain-containing protein 2B OS=Homo sapiens OX=9606 GN=SH3PXD2B PE=1 SV=3	4	2	2	101.5	4	911	8.69				1	1	2				1	1	2
High	Q8IYU8	Calcium uptake protein 2, mitochondrial OS=Homo sapiens OX=9606 GN=MICU2 PE=1 SV=2	4	2	2	49.6	9	434	9.09	1		2				1		2			
High	Q14142	Tripartite motif-containing protein 14 OS=Homo sapiens OX=9606 GN=TRIM14 PE=1 SV=2	4	2	2	49.7	6	442	7.87												
High	Q99627	COP9 signalosome complex subunit 8 OS=Homo sapiens OX=9606 GN=COPS8 PE=1 SV=1	4	1	1	23.2	10	209	5.38					1	1					1	1
High	Q9NVU0	DNA-directed RNA polymerase III subunit RPC5 OS=Homo sapiens OX=9606 GN=POLR3E PE=1 SV=1	4	2	2	79.8	4	708	6.47												
High	Q9NU22	Midasin OS=Homo sapiens OX=9606 GN=MDN1 PE=1 SV=2	4	3	3	632.4	1	5596	5.68		2						1				
High	Q12768	WASH complex subunit 5 OS=Homo sapiens OX=9606 GN=WASHC5 PE=1 SV=1	4	1	1	134.2	1	1159	6.98	1	1					1	1				
High	Q99549	M-phase phosphoprotein 8 OS=Homo sapiens OX=9606 GN=MPHOSPH8 PE=1 SV=2	4	2	2	97.1	4	860	6.06												
High	P82912	28S ribosomal protein S11, mitochondrial OS=Homo sapiens OX=9606 GN=MRPS11 PE=1 SV=2	4	1	1	20.6	8	194	10.81				1						1		
High	Q8N5P1	Zinc finger CCCH domain-containing protein 8 OS=Homo sapiens OX=9606 GN=ZC3H8 PE=1 SV=2	4	1	1	33.6	4	291	8.28				1						1		
High	P37837	Transaldolase OS=Homo sapiens OX=9606 GN=TALDO1 PE=1 SV=2	4	2	2	37.5	6	337	6.81	1	1	2				1	1	2			
High	Q86X10	Ral GTPase-activating protein subunit beta OS=Homo sapiens OX=9606 GN=RALGAPB PE=1 SV=1	4	2	2	166.7	2	1494	6.79	1	1					1	1				
High	Q9UKM7	Endoplasmic reticulum mannosyl-oligosaccharide 1,2-alpha-mannosidase OS=Homo sapiens OX=9606 GN=MAN1B1 PE=1 SV=2	4	2	2	79.5	5	699	7.72	1	1					1	1				
High	O14646	Chromodomain-helicase-DNA-binding protein 1 OS=Homo sapiens OX=9606 GN=CHD1 PE=1 SV=2	4	2	1	196.6	1	1710	7.14	1	1	1				1	1	1			
High	P25325	3-mercaptopyruvate sulfurtransferase OS=Homo sapiens OX=9606 GN=MPST PE=1 SV=3	4	2	2	33.2	13	297	6.6			1						1			
High	Q9H4L4	Sentrin-specific protease 3 OS=Homo sapiens OX=9606 GN=SENP3 PE=1 SV=2	4	2	2	65	4	574	8.56	1	1					1	1				
High	Q14002	Carcinoembryonic antigen-related cell adhesion molecule 7 OS=Homo sapiens OX=9606 GN=CEACAM7 PE=1 SV=2	4	3	3	29.3	14	265	5.54												
High	Q96IJ6	Mannose-1-phosphate guanyltransferase alpha OS=Homo sapiens OX=9606 GN=GMPPA PE=1 SV=1	4	2	2	46.3	5	420	7.21	1	1					1	1				
High	P00505	Aspartate aminotransferase, mitochondrial OS=Homo sapiens OX=9606 GN=GOT2 PE=1 SV=3	4	1	1	47.5	2	430	9.01			1	1	1	1			1	1	1	1
High	O43933	Peroxisome biogenesis factor 1 OS=Homo sapiens OX=9606 GN=PEX1 PE=1 SV=1	4	2	2	142.8	1	1283	6.33			1						1			
High	Q8WTW3	Conserved oligomeric Golgi complex subunit 1 OS=Homo sapiens OX=9606 GN=COG1 PE=1 SV=1	4	2	2	108.9	2	980	7.31	1	2	1				1	2	1			
High	Q9UBB6	Neurochondrin OS=Homo sapiens OX=9606 GN=NCDN PE=1 SV=1	4	2	2	78.8	4	729	5.48	1	1	1				1	1	1			
High	Q9Y666	Solute carrier family 12 member 7 OS=Homo sapiens OX=9606 GN=SLC12A7 PE=1 SV=3	4	1	1	119	2	1083	6.71			1						1			
High	O15228	Dihydroxyacetone phosphate acyltransferase OS=Homo sapiens OX=9606 GN=GNPAT PE=1 SV=1	4	3	3	77.1	4	680	6.57					1						1	
High	P50395	Rab GDP dissociation inhibitor beta OS=Homo sapiens OX=9606 GN=GDI2 PE=1 SV=2	4	2	1	50.6	6	445	6.47	1	1	1				1	1	1			
High	O00204	Sulfotransferase 2B1 OS=Homo sapiens OX=9606 GN=SULT2B1 PE=1 SV=2	4	2	2	41.3	11	365	5.4	1		1				1		1			
High	P07108	Acyl-CoA-binding protein OS=Homo sapiens OX=9606 GN=DBI PE=1 SV=2	4	1	1	10	23	87	6.57	1	1	1				1	1	1			
High	Q9H5V8	CUB domain-containing protein 1 OS=Homo sapiens OX=9606 GN=CDCP1 PE=1 SV=3	4	1	1	92.9	2	836	7.96	1	1					1	1				
High	P35226	Polycomb complex protein BMI-1 OS=Homo sapiens OX=9606 GN=BMI1 PE=1 SV=2	4	2	2	36.9	6	326	8.63				1	1					1	1	
High	Q9Y3C8	Ubiquitin-fold modifier-conjugating enzyme 1 OS=Homo sapiens OX=9606 GN=UFC1 PE=1 SV=3	4	2	2	19.4	11	167	7.4						1						1
High	Q13613	Myotubularin-related protein 1 OS=Homo sapiens OX=9606 GN=MTMR1 PE=1 SV=4	4	3	3	74.6	9	665	7.14	1	2					1	2				
High	Q9UNE7	E3 ubiquitin-protein ligase CHIP OS=Homo sapiens OX=9606 GN=STUB1 PE=1 SV=2	4	1	1	34.8	4	303	5.87					1	1					1	1
High	O95801	Tetratricopeptide repeat protein 4 OS=Homo sapiens OX=9606 GN=TTC4 PE=1 SV=3	4	1	1	44.7	3	387	5.6	1			1		1	1			1		1
High	Q5JVS0	Intracellular hyaluronan-binding protein 4 OS=Homo sapiens OX=9606 GN=HABP4 PE=1 SV=1	4	1	1	45.8	6	413	7.46	1	1			1		1	1			1	
High	P14314	Glucosidase 2 subunit beta OS=Homo sapiens OX=9606 GN=PRKCSH PE=1 SV=2	4	2	2	59.4	4	528	4.41		2	1					2	1			
High	Q96A49	Synapse-associated protein 1 OS=Homo sapiens OX=9606 GN=SYAP1 PE=1 SV=1	4	2	2	39.9	6	352	4.53												
High	Q96J02	E3 ubiquitin-protein ligase Itchy homolog OS=Homo sapiens OX=9606 GN=ITCH PE=1 SV=2	4	2	1	102.7	3	903	6.3	1	1					1	1				
High	Q5SNT2	Transmembrane protein 201 OS=Homo sapiens OX=9606 GN=TMEM201 PE=1 SV=1	4	2	2	72.2	3	666	9.22	1	1	1				1	1	1			
High	Q9H3S7	Tyrosine-protein phosphatase non-receptor type 23 OS=Homo sapiens OX=9606 GN=PTPN23 PE=1 SV=1	4	2	2	178.9	2	1636	6.92					1						1	
High	Q9H400	Lck-interacting transmembrane adapter 1 OS=Homo sapiens OX=9606 GN=LIME1 PE=1 SV=1	4	2	2	31.3	11	295	9.58												
High	Q08554	Desmocollin-1 OS=Homo sapiens OX=9606 GN=DSC1 PE=1 SV=2	4	1	1	99.9	1	894	5.43			1	1	1	1			1	1	1	1
High	Q96HW7	Integrator complex subunit 4 OS=Homo sapiens OX=9606 GN=INTS4 PE=1 SV=2	4	2	2	108.1	3	963	6.44	1	1	1				1	1	1			
High	Q9NR12	PDZ and LIM domain protein 7 OS=Homo sapiens OX=9606 GN=PDLIM7 PE=1 SV=1	4	3	3	49.8	7	457	8.41	1		2				1		1			
High	P02668	SWISS-PROT:P02668 Kappa-casein [Contains: Casoxin C; Casoxin 6; Casoxin A; Casoxin B; Casoplatelin] - Bos taurus (Bovine).	4	2	2	19	12	169	6.3					1						1	
High	O75884	Serine hydrolase RBBP9 OS=Homo sapiens OX=9606 GN=RBBP9 PE=1 SV=2	4	2	2	21	11	186	6.2			1						1			
High	P62894	SWISS-PROT:P62894 Cytochrome c - Bos taurus (Bovine).	4	2	1	11.7	17	105	9.5			1	1	1	1			1	1	1	1
High	Q16527	Cysteine and glycine-rich protein 2 OS=Homo sapiens OX=9606 GN=CSRP2 PE=1 SV=3	4	3	3	20.9	18	193	8.62				2	1	1				2	1	1
High	Q9H9Q4	Non-homologous end-joining factor 1 OS=Homo sapiens OX=9606 GN=NHEJ1 PE=1 SV=1	3	1	1	33.3	7	299	5.97			1						1			
High	Q96D71	RalBP1-associated Eps domain-containing protein 1 OS=Homo sapiens OX=9606 GN=REPS1 PE=1 SV=3	3	1	1	86.6	1	796	5.69												
High	Q96S66	Chloride channel CLIC-like protein 1 OS=Homo sapiens OX=9606 GN=CLCC1 PE=1 SV=1	3	1	1	62	5	551	5.55					1						1	
High	P53701	Holocytochrome c-type synthase OS=Homo sapiens OX=9606 GN=HCCS PE=1 SV=1	3	3	3	30.6	16	268	6.68												
High	Q8N9N2	Activating signal cointegrator 1 complex subunit 1 OS=Homo sapiens OX=9606 GN=ASCC1 PE=1 SV=1	3	1	1	45.5	2	400	5.54				1	1	1				1	1	1
High	Q5F1R6	DnaJ homolog subfamily C member 21 OS=Homo sapiens OX=9606 GN=DNAJC21 PE=1 SV=2	3	1	1	62	3	531	5.47												
High	Q9GZM7	Tubulointerstitial nephritis antigen-like OS=Homo sapiens OX=9606 GN=TINAGL1 PE=1 SV=1	3	1	1	52.4	2	467	6.99												
High	P49848	Transcription initiation factor TFIID subunit 6 OS=Homo sapiens OX=9606 GN=TAF6 PE=1 SV=1	3	2	2	72.6	3	677	8.6						1						1
High	Q96C36	Pyrroline-5-carboxylate reductase 2 OS=Homo sapiens OX=9606 GN=PYCR2 PE=1 SV=1	3	1	1	33.6	2	320	7.77												
High	O94988	Protein FAM13A OS=Homo sapiens OX=9606 GN=FAM13A PE=1 SV=2	3	1	1	116.9	2	1023	6.48			1						1			
High	P09914	Interferon-induced protein with tetratricopeptide repeats 1 OS=Homo sapiens OX=9606 GN=IFIT1 PE=1 SV=2	3	1	1	55.3	4	478	7.2	1				1	1	1				1	1
High	Q9P2R6	Arginine-glutamic acid dipeptide repeats protein OS=Homo sapiens OX=9606 GN=RERE PE=1 SV=2	3	1	1	172.3	1	1566	7.69			1		1				1		1	
High	Q13043	Serine/threonine-protein kinase 4 OS=Homo sapiens OX=9606 GN=STK4 PE=1 SV=2	3	2	1	55.6	5	487	5.07	1				1		1				1	
High	Q9Y4H2	Insulin receptor substrate 2 OS=Homo sapiens OX=9606 GN=IRS2 PE=1 SV=2	3	3	3	137.2	6	1338	8.65					1						1	
High	Q7Z333	Probable helicase senataxin OS=Homo sapiens OX=9606 GN=SETX PE=1 SV=4	3	2	2	302.7	1	2677	7.17					1						1	
High	P63146	Ubiquitin-conjugating enzyme E2 B OS=Homo sapiens OX=9606 GN=UBE2B PE=1 SV=1	3	1	1	17.3	11	152	5.01	1	1	1				1	1	1			
High	Q96TC7	Regulator of microtubule dynamics protein 3 OS=Homo sapiens OX=9606 GN=RMDN3 PE=1 SV=2	3	1	1	52.1	4	470	5.1	1	1	1				1	1	1			
High	Q6UW68	Transmembrane protein 205 OS=Homo sapiens OX=9606 GN=TMEM205 PE=1 SV=1	3	1	1	21.2	9	189	8.62			1						1			
High	P13674	Prolyl 4-hydroxylase subunit alpha-1 OS=Homo sapiens OX=9606 GN=P4HA1 PE=1 SV=2	3	2	2	61	5	534	6.01			1						1			
High	O14562	Ubiquitin domain-containing protein UBFD1 OS=Homo sapiens OX=9606 GN=UBFD1 PE=1 SV=2	3	2	2	33.4	7	309	5.77												
High	P05496	ATP synthase F(0) complex subunit C1, mitochondrial OS=Homo sapiens OX=9606 GN=ATP5MC1 PE=1 SV=2	3	1	1	14.3	23	136	9.74				1		1				1		1
High	Q9BUZ4	TNF receptor-associated factor 4 OS=Homo sapiens OX=9606 GN=TRAF4 PE=1 SV=1	3	1	1	53.5	3	470	8.15												
High	P26583	High mobility group protein B2 OS=Homo sapiens OX=9606 GN=HMGB2 PE=1 SV=2	3	1	1	24	6	209	7.81	1	1	1				1	1	1			
High	P33240	Cleavage stimulation factor subunit 2 OS=Homo sapiens OX=9606 GN=CSTF2 PE=1 SV=1	3	1	1	60.9	2	577	6.83					1	1					1	1
High	Q969T9	WW domain-binding protein 2 OS=Homo sapiens OX=9606 GN=WBP2 PE=1 SV=1	3	1	1	28.1	4	261	5.91	1		1				1		1			
High	Q9HBY8	Serine/threonine-protein kinase Sgk2 OS=Homo sapiens OX=9606 GN=SGK2 PE=1 SV=2	3	1	1	41.1	2	367	6.74	1	1					1	1				
High	Q8N3D4	EH domain-binding protein 1-like protein 1 OS=Homo sapiens OX=9606 GN=EHBP1L1 PE=1 SV=2	3	1	1	161.8	1	1523	4.83			1						1			
High	Q8N163	Cell cycle and apoptosis regulator protein 2 OS=Homo sapiens OX=9606 GN=CCAR2 PE=1 SV=2	3	1	1	102.8	1	923	5.22	1						1					
High	Q96GX2	Ataxin-7-like protein 3B OS=Homo sapiens OX=9606 GN=ATXN7L3B PE=1 SV=2	3	2	2	10.8	31	97	4.36			1		1				1		1	
High	Q8N5Z5	BTB/POZ domain-containing protein KCTD17 OS=Homo sapiens OX=9606 GN=KCTD17 PE=1 SV=3	3	2	2	35.6	8	321	4.86					2	1					2	1
High	Q5TCZ1	SH3 and PX domain-containing protein 2A OS=Homo sapiens OX=9606 GN=SH3PXD2A PE=1 SV=1	3	1	1	125.2	1	1133	8.9												
High	P51648	Aldehyde dehydrogenase family 3 member A2 OS=Homo sapiens OX=9606 GN=ALDH3A2 PE=1 SV=1	3	1	1	54.8	3	485	7.88					1						1	
High	O60573	Eukaryotic translation initiation factor 4E type 2 OS=Homo sapiens OX=9606 GN=EIF4E2 PE=1 SV=1	3	2	2	28.3	16	245	8.88			1						1			
High	Q13405	39S ribosomal protein L49, mitochondrial OS=Homo sapiens OX=9606 GN=MRPL49 PE=1 SV=1	3	2	2	19.2	16	166	9.45												
High	Q8IZQ5	Selenoprotein H OS=Homo sapiens OX=9606 GN=SELENOH PE=1 SV=2	3	1	1	13.4	9	122	9.74				1						1		
High	Q9UGU0	Transcription factor 20 OS=Homo sapiens OX=9606 GN=TCF20 PE=1 SV=3	3	3	3	211.6	2	1960	9.04		1	2					1	2			
High	Q9UBP0	Spastin OS=Homo sapiens OX=9606 GN=SPAST PE=1 SV=1	3	1	1	67.2	3	616	9.64	1		1				1		1			
High	Q7L2H7	Eukaryotic translation initiation factor 3 subunit M OS=Homo sapiens OX=9606 GN=EIF3M PE=1 SV=1	3	2	2	42.5	9	374	5.63	1	1					1	1				
High	Q9H078	Caseinolytic peptidase B protein homolog OS=Homo sapiens OX=9606 GN=CLPB PE=1 SV=1	3	1	1	78.7	3	707	9.01					1						1	
High	Q92844	TRAF family member-associated NF-kappa-B activator OS=Homo sapiens OX=9606 GN=TANK PE=1 SV=2	3	2	2	47.8	9	425	5.68												
High	P49903	Selenide, water dikinase 1 OS=Homo sapiens OX=9606 GN=SEPHS1 PE=1 SV=2	3	1	1	42.9	4	392	5.97			1						1			
High	Q9UBT2	SUMO-activating enzyme subunit 2 OS=Homo sapiens OX=9606 GN=UBA2 PE=1 SV=2	3	2	2	71.2	3	640	5.29		1	1					1	1			
High	O95352	Ubiquitin-like modifier-activating enzyme ATG7 OS=Homo sapiens OX=9606 GN=ATG7 PE=1 SV=1	3	2	2	77.9	3	703	6.24												
High	Q9BX46	RNA-binding protein 24 OS=Homo sapiens OX=9606 GN=RBM24 PE=1 SV=1	3	1	1	24.8	7	236	8.51			1						1			
High	O00178	GTP-binding protein 1 OS=Homo sapiens OX=9606 GN=GTPBP1 PE=1 SV=3	3	1	1	72.4	2	669	8.34			1						1			
High	Q5VT52	Regulation of nuclear pre-mRNA domain-containing protein 2 OS=Homo sapiens OX=9606 GN=RPRD2 PE=1 SV=1	3	2	2	155.9	3	1461	7.42												
High	O76062	Delta(14)-sterol reductase TM7SF2 OS=Homo sapiens OX=9606 GN=TM7SF2 PE=1 SV=3	3	1	1	46.4	3	418	8.87	1	1	1				1	1	1			
High	Q9UJ83	2-hydroxyacyl-CoA lyase 1 OS=Homo sapiens OX=9606 GN=HACL1 PE=1 SV=2	3	2	2	63.7	6	578	7.36												
High	Q14802	FXYD domain-containing ion transport regulator 3 OS=Homo sapiens OX=9606 GN=FXYD3 PE=1 SV=1	3	1	1	9.3	22	87	7.3												
High	Q92692	Nectin-2 OS=Homo sapiens OX=9606 GN=NECTIN2 PE=1 SV=1	3	2	2	57.7	3	538	4.82												
High	P46937	Transcriptional coactivator YAP1 OS=Homo sapiens OX=9606 GN=YAP1 PE=1 SV=2	3	2	2	54.4	7	504	5.17					1						1	
High	Q9H9B1	Histone-lysine N-methyltransferase EHMT1 OS=Homo sapiens OX=9606 GN=EHMT1 PE=1 SV=4	3	1	1	141.4	1	1298	5.76												
High	Q9BTE1	Dynactin subunit 5 OS=Homo sapiens OX=9606 GN=DCTN5 PE=1 SV=1	3	2	2	20.1	12	182	8.02				1		1				1		1
High	Q6ZU15	Septin-14 OS=Homo sapiens OX=9606 GN=SEPTIN14 PE=1 SV=2	3	2	1	50	6	432	6.24				1						1		
High	Q9Y5S9	RNA-binding protein 8A OS=Homo sapiens OX=9606 GN=RBM8A PE=1 SV=1	3	2	2	19.9	16	174	5.72			1		1				1		1	
High	Q9BRK4	Leucine zipper putative tumor suppressor 2 OS=Homo sapiens OX=9606 GN=LZTS2 PE=1 SV=2	3	1	1	72.7	2	669	6.51	1	1					1	1				
High	P06732	Creatine kinase M-type OS=Homo sapiens OX=9606 GN=CKM PE=1 SV=2	3	2	2	43.1	6	381	7.25				2		1				2		1
High	O95398	Rap guanine nucleotide exchange factor 3 OS=Homo sapiens OX=9606 GN=RAPGEF3 PE=1 SV=6	3	2	2	103.7	3	923	7.56												
High	Q9NXE8	Pre-mRNA-splicing factor CWC25 homolog OS=Homo sapiens OX=9606 GN=CWC25 PE=1 SV=1	3	2	2	49.6	5	425	10.18					2	1					2	1
High	P60059	Protein transport protein Sec61 subunit gamma OS=Homo sapiens OX=9606 GN=SEC61G PE=1 SV=1	3	1	1	7.7	18	68	9.99												
High	Q99538	Legumain OS=Homo sapiens OX=9606 GN=LGMN PE=1 SV=1	3	2	2	49.4	7	433	6.55	1		2				1		1			
High	Q96HI0	Sentrin-specific protease 5 OS=Homo sapiens OX=9606 GN=SENP5 PE=1 SV=3	3	3	3	86.6	5	755	9.17												
High	Q5SWX8	Protein odr-4 homolog OS=Homo sapiens OX=9606 GN=ODR4 PE=1 SV=1	3	2	2	51.1	6	454	5.92	1	1					1	1				
High	Q12955	Ankyrin-3 OS=Homo sapiens OX=9606 GN=ANK3 PE=1 SV=3	3	1	1	480.1	0	4377	6.49					1						1	
High	P30989	Neurotensin receptor type 1 OS=Homo sapiens OX=9606 GN=NTSR1 PE=1 SV=2	3	1	1	46.2	4	418	9.35	1	1					1	1				
High	P09884	DNA polymerase alpha catalytic subunit OS=Homo sapiens OX=9606 GN=POLA1 PE=1 SV=2	3	2	2	165.8	2	1462	5.85	1	1					1	1				
High	P61009	Signal peptidase complex subunit 3 OS=Homo sapiens OX=9606 GN=SPCS3 PE=1 SV=1	3	1	1	20.3	5	180	8.62	1						1					
High	Q8IUR0	Trafficking protein particle complex subunit 5 OS=Homo sapiens OX=9606 GN=TRAPPC5 PE=1 SV=1	3	1	1	20.8	4	188	9.66												
High	O60563	Cyclin-T1 OS=Homo sapiens OX=9606 GN=CCNT1 PE=1 SV=1	3	1	1	80.6	2	726	8.78												
High	Q92917	G-patch domain and KOW motifs-containing protein OS=Homo sapiens OX=9606 GN=GPKOW PE=1 SV=2	3	2	2	52.2	6	476	6.15												
High	Q8NF37	Lysophosphatidylcholine acyltransferase 1 OS=Homo sapiens OX=9606 GN=LPCAT1 PE=1 SV=2	3	2	2	59.1	4	534	6.02					1						1	
High	Q9NPF5	DNA methyltransferase 1-associated protein 1 OS=Homo sapiens OX=9606 GN=DMAP1 PE=1 SV=1	3	1	1	53	3	467	9.5					1						1	
High	Q5EBM0	UMP-CMP kinase 2, mitochondrial OS=Homo sapiens OX=9606 GN=CMPK2 PE=1 SV=3	3	1	1	49.4	3	449	7.01					1	1					1	1
High	Q9Y450	HBS1-like protein OS=Homo sapiens OX=9606 GN=HBS1L PE=1 SV=1	3	2	2	75.4	4	684	6.61												
High	O15037	Protein KHNYN OS=Homo sapiens OX=9606 GN=KHNYN PE=1 SV=3	3	1	1	74.5	3	678	7.02	1	1	1				1	1	1			
High	Q15800	Methylsterol monooxygenase 1 OS=Homo sapiens OX=9606 GN=MSMO1 PE=1 SV=1	3	1	1	35.2	5	293	7.23	1	1	1				1	1	1			

High	Q9P0M9	39S ribosomal protein L27, mitochondrial OS=Homo sapiens OX=9606 GN=MRPL27 PE=1 SV=1	3	1	1	16.1	7	148	10.42				1		1				1		1
High	O43237	Cytoplasmic dynein 1 light intermediate chain 2 OS=Homo sapiens OX=9606 GN=DYNC1LI2 PE=1 SV=1	3	2	2	54.1	4	492	6.38												
High	P51948	CDK-activating kinase assembly factor MAT1 OS=Homo sapiens OX=9606 GN=MNAT1 PE=1 SV=1	3	1	1	35.8	5	309	6.09			1						1			
High	P54709	Sodium/potassium-transporting ATPase subunit beta-3 OS=Homo sapiens OX=9606 GN=ATP1B3 PE=1 SV=1	3	1	1	31.5	5	279	8.35					1						1	
High	Q9BVQ7	Spermatogenesis-associated protein 5-like protein 1 OS=Homo sapiens OX=9606 GN=SPATA5L1 PE=1 SV=2	3	1	1	80.7	3	753	8.09		1						1				
High	Q7L7V1	Putative pre-mRNA-splicing factor ATP-dependent RNA helicase DHX32 OS=Homo sapiens OX=9606 GN=DHX32 PE=1 SV=1	3	1	1	84.4	2	743	4.97			1						1			
High	O75027	Iron-sulfur clusters transporter ABCB7, mitochondrial OS=Homo sapiens OX=9606 GN=ABCB7 PE=1 SV=2	3	1	1	82.6	2	752	9.33	1	1	1				1	1	1			
High	Q6ZN54	Differentially expressed in FDCP 8 homolog OS=Homo sapiens OX=9606 GN=DEF8 PE=1 SV=2	3	1	1	58.7	3	512	6.52	1	1	1				1	1	1			
High	O60488	Long-chain-fatty-acid--CoA ligase 4 OS=Homo sapiens OX=9606 GN=ACSL4 PE=1 SV=2	3	2	1	79.1	5	711	8.38												
High	Q00653	Nuclear factor NF-kappa-B p100 subunit OS=Homo sapiens OX=9606 GN=NFKB2 PE=1 SV=4	3	2	2	96.7	3	900	6.25		1			1			1			1	
High	P49585	Choline-phosphate cytidylyltransferase A OS=Homo sapiens OX=9606 GN=PCYT1A PE=1 SV=2	3	2	2	41.7	5	367	7.25				1		1				1		1
High	Q14210	Lymphocyte antigen 6D OS=Homo sapiens OX=9606 GN=LY6D PE=1 SV=1	3	1	1	13.3	7	128	8.21				1	1	1				1	1	1
High	P00568	Adenylate kinase isoenzyme 1 OS=Homo sapiens OX=9606 GN=AK1 PE=1 SV=3	3	1	1	21.6	7	194	8.63			1		1				1		1	
High	Q53HC5	Kelch-like protein 26 OS=Homo sapiens OX=9606 GN=KLHL26 PE=1 SV=2	3	1	1	68.1	1	615	6.48				1	1	1				1	1	1
High	Q9Y673	Dolichyl-phosphate beta-glucosyltransferase OS=Homo sapiens OX=9606 GN=ALG5 PE=1 SV=1	3	2	2	36.9	10	324	9.28			1						1			
High	Q8N9N8	Probable RNA-binding protein EIF1AD OS=Homo sapiens OX=9606 GN=EIF1AD PE=1 SV=1	3	1	1	19	10	165	5.21					1						1	
High	Q8NHQ9	ATP-dependent RNA helicase DDX55 OS=Homo sapiens OX=9606 GN=DDX55 PE=1 SV=3	3	2	2	68.5	4	600	9.25					1						1	
High	Q14232	Translation initiation factor eIF-2B subunit alpha OS=Homo sapiens OX=9606 GN=EIF2B1 PE=1 SV=1	3	1	1	33.7	3	305	7.33			1						1			
High	P78314	SH3 domain-binding protein 2 OS=Homo sapiens OX=9606 GN=SH3BP2 PE=1 SV=2	3	2	2	62.2	4	561	7.78		1						1				
High	Q9HD20	Endoplasmic reticulum transmembrane helix translocase OS=Homo sapiens OX=9606 GN=ATP13A1 PE=1 SV=2	3	1	1	132.9	1	1204	8.13			1						1			
High	Q92599	Septin-8 OS=Homo sapiens OX=9606 GN=SEPTIN8 PE=1 SV=4	3	2	1	55.7	5	483	6.28		1		1				1		1		
High	Q96AQ6	Pre-B-cell leukemia transcription factor-interacting protein 1 OS=Homo sapiens OX=9606 GN=PBXIP1 PE=1 SV=1	3	2	2	80.6	5	731	5.33												
High	Q8IZ69	tRNA (uracil-5-)-methyltransferase homolog A OS=Homo sapiens OX=9606 GN=TRMT2A PE=1 SV=2	3	2	2	68.7	6	625	7.94	1	1					1	1				
High	Q8IY81	pre-rRNA 2'-O-ribose RNA methyltransferase FTSJ3 OS=Homo sapiens OX=9606 GN=FTSJ3 PE=1 SV=2	3	2	2	96.5	4	847	8.4				1						1		
High	Q7Z4H7	HAUS augmin-like complex subunit 6 OS=Homo sapiens OX=9606 GN=HAUS6 PE=1 SV=2	3	1	1	108.6	2	955	6.47		1	1					1	1			
High	Q9Y2H6	Fibronectin type-III domain-containing protein 3A OS=Homo sapiens OX=9606 GN=FNDC3A PE=1 SV=4	3	2	2	131.8	3	1198	6.71												
High	P55957	BH3-interacting domain death agonist OS=Homo sapiens OX=9606 GN=BID PE=1 SV=1	3	1	1	22	8	195	5.44	1	1	1				1	1	1			
High	Q13227	G protein pathway suppressor 2 OS=Homo sapiens OX=9606 GN=GPS2 PE=1 SV=3	3	1	1	36.7	2	327	9.52												
High	Q9NY59	Sphingomyelin phosphodiesterase 3 OS=Homo sapiens OX=9606 GN=SMPD3 PE=1 SV=1	3	1	1	71	2	655	5.78												
High	Q03169	Tumor necrosis factor alpha-induced protein 2 OS=Homo sapiens OX=9606 GN=TNFAIP2 PE=1 SV=2	3	2	2	72.6	4	654	6.46		1	2					1	2			
High	O75306	NADH dehydrogenase [ubiquinone] iron-sulfur protein 2, mitochondrial OS=Homo sapiens OX=9606 GN=NDUFS2 PE=1 SV=2	3	2	2	52.5	3	463	7.55	1		1				1		1			
High	Q6DCA0	AMMECR1-like protein OS=Homo sapiens OX=9606 GN=AMMECR1L PE=1 SV=1	3	1	1	34.5	2	310	8.98				1	1	1				1	1	1
High	P15497	SWISS-PROT:P15497 (Bos taurus) Apolipoprotein A-I precursor	3	1	1	30.3	4	265	5.97				1	1	1				1	1	1
High	O00566	U3 small nucleolar ribonucleoprotein protein MPP10 OS=Homo sapiens OX=9606 GN=MPHOSPH10 PE=1 SV=2	3	1	1	78.8	1	681	4.86												
High	Q9BXS5	AP-1 complex subunit mu-1 OS=Homo sapiens OX=9606 GN=AP1M1 PE=1 SV=3	3	1	1	48.6	4	423	7.3			1						1			
High	Q8NCW5	NAD(P)H-hydrate epimerase OS=Homo sapiens OX=9606 GN=NAXE PE=1 SV=2	3	2	2	31.7	10	288	7.66												
High	O43402	ER membrane protein complex subunit 8 OS=Homo sapiens OX=9606 GN=EMC8 PE=1 SV=1	3	1	1	23.8	4	210	6.4												
High	Q9C0C7	Activating molecule in BECN1-regulated autophagy protein 1 OS=Homo sapiens OX=9606 GN=AMBRA1 PE=1 SV=2	3	2	2	142.4	3	1298	7.18				1	1					1	1	
High	Q5UIP0	Telomere-associated protein RIF1 OS=Homo sapiens OX=9606 GN=RIF1 PE=1 SV=2	3	2	2	274.3	1	2472	5.52												
High	O00244	Copper transport protein ATOX1 OS=Homo sapiens OX=9606 GN=ATOX1 PE=1 SV=1	3	1	1	7.4	12	68	7.24			1		1				1		1	
High	Q9UBB4	Ataxin-10 OS=Homo sapiens OX=9606 GN=ATXN10 PE=1 SV=1	3	1	1	53.5	2	475	5.25		1	1			1		1	1			1
High	Q15004	PCNA-associated factor OS=Homo sapiens OX=9606 GN=PCLAF PE=1 SV=1	3	2	2	12	28	111	9.83				1	1	1				1	1	1
High	P61803	Dolichyl-diphosphooligosaccharide--protein glycosyltransferase subunit DAD1 OS=Homo sapiens OX=9606 GN=DAD1 PE=1 SV=3	3	1	1	12.5	11	113	7.08	1	1	1				1	1	1			
High	P00519	Tyrosine-protein kinase ABL1 OS=Homo sapiens OX=9606 GN=ABL1 PE=1 SV=4	3	1	1	122.8	1	1130	8.66			1						1			
High	Q92504	Zinc transporter SLC39A7 OS=Homo sapiens OX=9606 GN=SLC39A7 PE=1 SV=2	3	2	2	50.1	3	469	6.87		1						1				
High	Q03426	Mevalonate kinase OS=Homo sapiens OX=9606 GN=MVK PE=1 SV=1	3	2	2	42.4	12	396	6.46	1		1				1		1			
High	P28065	Proteasome subunit beta type-9 OS=Homo sapiens OX=9606 GN=PSMB9 PE=1 SV=2	3	1	1	23.3	5	219	5.03				1	1	1				1	1	1
High	O15120	1-acyl-sn-glycerol-3-phosphate acyltransferase beta OS=Homo sapiens OX=9606 GN=AGPAT2 PE=1 SV=1	3	1	1	30.9	3	278	9.01	1	1					1	1				
High	Q04864	Proto-oncogene c-Rel OS=Homo sapiens OX=9606 GN=REL PE=1 SV=1	3	1	1	68.5	2	619	5.86												
High	Q68D10	Protein SPT2 homolog OS=Homo sapiens OX=9606 GN=SPTY2D1 PE=1 SV=3	3	2	2	75.6	3	685	9.79	1						1					
High	P49773	Adenosine 5'-monophosphoramidase HINT1 OS=Homo sapiens OX=9606 GN=HINT1 PE=1 SV=2	3	1	1	13.8	7	126	6.95	1				1		1				1	
High	Q96GQ5	RUS family member 1 OS=Homo sapiens OX=9606 GN=RUSF1 PE=1 SV=2	3	2	2	51	8	468	6.93	1	1	1				1	1	1			
High	Q9UIV1	CCR4-NOT transcription complex subunit 7 OS=Homo sapiens OX=9606 GN=CNOT7 PE=1 SV=3	3	2	2	32.7	9	285	4.84			1						1			
High	Q8NDH3	Probable aminopeptidase NPEPL1 OS=Homo sapiens OX=9606 GN=NPEPL1 PE=1 SV=3	3	2	2	55.8	5	523	6.87		1	1					1	1			
High	P13716	Delta-aminolevulinic acid dehydratase OS=Homo sapiens OX=9606 GN=ALAD PE=1 SV=1	3	1	1	36.3	3	330	6.79	1				1		1				1	
High	P55854	Small ubiquitin-related modifier 3 OS=Homo sapiens OX=9606 GN=SUMO3 PE=1 SV=2	3	1	1	11.6	12	103	5.49												
High	Q86Y37	CDK2-associated and cullin domain-containing protein 1 OS=Homo sapiens OX=9606 GN=CACUL1 PE=1 SV=1	3	2	2	41	9	369	5.19	1	1					1	1				
High	Q9BV38	WD repeat-containing protein 18 OS=Homo sapiens OX=9606 GN=WDR18 PE=1 SV=2	3	2	2	47.4	6	432	6.7	1		1	1			1		1	1		
High	Q9UIA9	Exportin-7 OS=Homo sapiens OX=9606 GN=XPO7 PE=1 SV=3	3	3	3	123.8	4	1087	6.32		1	1					1	1			
High	Q9NQ84	G-protein coupled receptor family C group 5 member C OS=Homo sapiens OX=9606 GN=GPRC5C PE=1 SV=2	3	2	2	48.2	5	441	8.43												
High	Q9BZ29	Dedicator of cytokinesis protein 9 OS=Homo sapiens OX=9606 GN=DOCK9 PE=1 SV=2	3	2	2	236.3	1	2069	7.49	1	1					1	1				
High	P07741	Adenine phosphoribosyltransferase OS=Homo sapiens OX=9606 GN=APRT PE=1 SV=2	3	2	2	19.6	10	180	6.02	1		1				1		1			
High	Q9Y6I9	Testis-expressed protein 264 OS=Homo sapiens OX=9606 GN=TEX264 PE=1 SV=1	3	1	1	34.2	4	313	4.86												
High	Q14534	Squalene monooxygenase OS=Homo sapiens OX=9606 GN=SQLE PE=1 SV=3	3	2	2	63.9	5	574	8.63			2						2			
High	Q9NYI0	PH and SEC7 domain-containing protein 3 OS=Homo sapiens OX=9606 GN=PSD3 PE=1 SV=2	3	1	1	116	2	1048	5.99			1						1			
High	Q9NZT1	Calmodulin-like protein 5 OS=Homo sapiens OX=9606 GN=CALML5 PE=1 SV=2	3	1	1	15.9	5	146	4.44				1	1	1				1	1	1
High	Q05469	Hormone-sensitive lipase OS=Homo sapiens OX=9606 GN=LIPE PE=1 SV=4	3	2	2	116.5	1	1076	6.7												
High	Q9Y262	Eukaryotic translation initiation factor 3 subunit L OS=Homo sapiens OX=9606 GN=EIF3L PE=1 SV=1	3	3	3	66.7	6	564	6.34					1						1	
High	Q96P48	Arf-GAP with Rho-GAP domain, ANK repeat and PH domain-containing protein 1 OS=Homo sapiens OX=9606 GN=ARAP1 PE=1 SV=	3	2	2	162.1	2	1450	6.23												
High	Q9UKK9	ADP-sugar pyrophosphatase OS=Homo sapiens OX=9606 GN=NUDT5 PE=1 SV=1	3	1	1	24.3	4	219	4.94			1		1				1		1	
High	P53384	Cytosolic Fe-S cluster assembly factor NUBP1 OS=Homo sapiens OX=9606 GN=NUBP1 PE=1 SV=2	3	1	1	34.5	6	320	5.33												
High	Q9H7B2	Ribosome production factor 2 homolog OS=Homo sapiens OX=9606 GN=RPF2 PE=1 SV=2	3	1	1	35.6	6	306	9.99	1		1				1		1			
High	Q9UL33	Trafficking protein particle complex subunit 2-like protein OS=Homo sapiens OX=9606 GN=TRAPPC2L PE=1 SV=1	3	1	1	16.1	14	140	6.77			1						1			
High	Q9BUN8	Derlin-1 OS=Homo sapiens OX=9606 GN=DERL1 PE=1 SV=1	3	1	1	28.8	4	251	9.51	1	1	1				1	1	1			
High	Q15032	R3H domain-containing protein 1 OS=Homo sapiens OX=9606 GN=R3HDM1 PE=1 SV=3	3	2	2	120.6	3	1099	8.75		1	2					1	2			
High	Q16540	39S ribosomal protein L23, mitochondrial OS=Homo sapiens OX=9606 GN=MRPL23 PE=1 SV=1	3	1	1	17.8	10	153	9.69				1						1		
High	O14497	AT-rich interactive domain-containing protein 1A OS=Homo sapiens OX=9606 GN=ARID1A PE=1 SV=3	3	2	2	241.9	2	2285	6.7												
High	Q8N357	Solute carrier family 35 member F6 OS=Homo sapiens OX=9606 GN=SLC35F6 PE=1 SV=1	3	1	1	40.2	3	371	6.93	1	1	1				1	1	1			
High	O43292	Glycosylphosphatidylinositol anchor attachment 1 protein OS=Homo sapiens OX=9606 GN=GPAA1 PE=1 SV=3	3	1	1	67.6	1	621	8.06	1	1	1				1	1	1			
High	Q8IWI9	MAX gene-associated protein OS=Homo sapiens OX=9606 GN=MGA PE=1 SV=4	3	1	1	336	0	3065	6.9												
High	Q9H974	Queuine tRNA-ribosyltransferase accessory subunit 2 OS=Homo sapiens OX=9606 GN=QTRT2 PE=1 SV=1	3	1	1	46.7	5	415	6.81	1	1	1				1	1	1			
High	Q9UPP1	Histone lysine demethylase PHF8 OS=Homo sapiens OX=9606 GN=PHF8 PE=1 SV=3	3	3	3	117.8	4	1060	8.72												
High	Q96JN0	Ligand-dependent corepressor OS=Homo sapiens OX=9606 GN=LCOR PE=1 SV=2	3	1	1	47	3	433	9.38	1	1	1				1	1	1			
High	O43709	Probable 18S rRNA (guanine-N(7))-methyltransferase OS=Homo sapiens OX=9606 GN=BUD23 PE=1 SV=2	3	2	2	31.9	7	281	8.73					1	1					1	1
High	O14925	Mitochondrial import inner membrane translocase subunit Tim23 OS=Homo sapiens OX=9606 GN=TIMM23 PE=1 SV=1	3	1	1	21.9	5	209	8.6	1	1	1				1	1	1			
High	Q8N9E0	Protein FAM133A OS=Homo sapiens OX=9606 GN=FAM133A PE=1 SV=1	3	1	1	28.9	3	248	10.1				1	1	1				1	1	1
High	Q96M96	FYVE, RhoGEF and PH domain-containing protein 4 OS=Homo sapiens OX=9606 GN=FGD4 PE=1 SV=2	3	1	1	86.6	1	766	6.13												
High	Q9NRR4	Ribonuclease 3 OS=Homo sapiens OX=9606 GN=DROSHA PE=1 SV=2	3	1	1	159.2	1	1374	7.87		1	1					1	1			
High	P62745	Rho-related GTP-binding protein RhoB OS=Homo sapiens OX=9606 GN=RHOB PE=1 SV=1	3	2	1	22.1	17	196	5.24	1	1	1				1	1	1			
High	P82909	Alpha-ketoglutarate dehydrogenase component 4 OS=Homo sapiens OX=9606 GN=MRPS36 PE=1 SV=2	3	2	2	11.5	27	103	9.99	1	1					1	1				
High	Q9NPR2	Semaphorin-4B OS=Homo sapiens OX=9606 GN=SEMA4B PE=1 SV=4	3	2	2	92.7	3	837	7.05	1	1					1	1				
High	Q66K14	TBC1 domain family member 9B OS=Homo sapiens OX=9606 GN=TBC1D9B PE=1 SV=3	3	1	1	140.4	2	1250	5.25			1						1			
High	Q15054	DNA polymerase delta subunit 3 OS=Homo sapiens OX=9606 GN=POLD3 PE=1 SV=2	3	2	2	51.4	5	466	9.35			1						1			
High	O60504	Vinexin OS=Homo sapiens OX=9606 GN=SORBS3 PE=1 SV=2	3	1	1	75.3	2	671	9.45												
High	Q8NEZ4	Histone-lysine N-methyltransferase 2C OS=Homo sapiens OX=9606 GN=KMT2C PE=1 SV=3	3	2	2	541	0	4911	6.49				1	1	1				1	1	1
High	Q9NR09	Baculoviral IAP repeat-containing protein 6 OS=Homo sapiens OX=9606 GN=BIRC6 PE=1 SV=3	3	2	2	529.9	1	4857	6.05												
High	O95154	Aflatoxin B1 aldehyde reductase member 3 OS=Homo sapiens OX=9606 GN=AKR7A3 PE=1 SV=2	3	3	2	37.2	13	331	7.15		1						1				
High	Q00403	Transcription initiation factor IIB OS=Homo sapiens OX=9606 GN=GTF2B PE=1 SV=1	3	1	1	34.8	5	316	8.35		1			1			1			1	
High	Q9C0D5	Protein TANC1 OS=Homo sapiens OX=9606 GN=TANC1 PE=1 SV=3	3	2	2	202.1	1	1861	8.32												
High	Q4G0X4	BTB/POZ domain-containing protein KCTD21 OS=Homo sapiens OX=9606 GN=KCTD21 PE=1 SV=1	3	1	1	29.6	7	260	6.54	1	1	1				1	1	1			
High	Q969S9	Ribosome-releasing factor 2, mitochondrial OS=Homo sapiens OX=9606 GN=GFM2 PE=1 SV=1	3	2	2	86.5	2	779	6.51	1	1					1	1				
High	Q8IXM3	39S ribosomal protein L41, mitochondrial OS=Homo sapiens OX=9606 GN=MRPL41 PE=1 SV=1	3	2	2	15.4	18	137	9.57						1						1
High	Q9H300	Presenilins-associated rhomboid-like protein, mitochondrial OS=Homo sapiens OX=9606 GN=PARL PE=1 SV=2	3	1	1	42.2	2	379	9.79	1	1	1				1	1	1			
High	P02663	SWISS-PROT:P02663 Alpha-S2-casein [Contains: Casocidin-1 - Bos taurus (Bovine).	3	2	2	24.3	9	207	8.27												
High	P49840	Glycogen synthase kinase-3 alpha OS=Homo sapiens OX=9606 GN=GSK3A PE=1 SV=2	3	1	1	50.9	3	483	8.75	1	1	1				1	1	1			
High	Q6ZU65	Ubinuclein-2 OS=Homo sapiens OX=9606 GN=UBN2 PE=1 SV=2	3	2	2	146	3	1347	9.19			1						1			
High	Q7Z4V5	Hepatoma-derived growth factor-related protein 2 OS=Homo sapiens OX=9606 GN=HDGFL2 PE=1 SV=1	3	2	2	74.3	4	671	7.49	1						1					
High	Q5T750	Skin-specific protein 32 OS=Homo sapiens OX=9606 GN=XP32 PE=1 SV=1	3	1	1	26.2	3	250	7.97			1		1				1		1	
High	P28062	Proteasome subunit beta type-8 OS=Homo sapiens OX=9606 GN=PSMB8 PE=1 SV=3	3	2	2	30.3	9	276	7.43				1	1	1				1	1	1
High	O43159	Ribosomal RNA-processing protein 8 OS=Homo sapiens OX=9606 GN=RRP8 PE=1 SV=2	3	2	2	50.7	6	456	9.42												
High	O95619	YEATS domain-containing protein 4 OS=Homo sapiens OX=9606 GN=YEATS4 PE=1 SV=1	3	1	1	26.5	4	227	8.41	1	1	1				1	1	1			
High	O60936	Nucleolar protein 3 OS=Homo sapiens OX=9606 GN=NOL3 PE=1 SV=2	3	1	1	22.6	9	208	4.18												
High	Q13610	Periodic tryptophan protein 1 homolog OS=Homo sapiens OX=9606 GN=PWP1 PE=1 SV=1	3	2	2	55.8	5	501	4.77												
High	P46100	Transcriptional regulator ATRX OS=Homo sapiens OX=9606 GN=ATRX PE=1 SV=6	3	2	2	282.4	1	2492	6.55			2						2			
High	Q13572	Inositol-tetrakisphosphate 1-kinase OS=Homo sapiens OX=9606 GN=ITPK1 PE=1 SV=2	3	2	2	45.6	7	414	6.16	1	1					1	1				
High	Q9NXC5	GATOR complex protein MIOS OS=Homo sapiens OX=9606 GN=MIOS PE=1 SV=2	3	1	1	98.5	1	875	6.73			1						1			
High	P00846	ATP synthase subunit a OS=Homo sapiens OX=9606 GN=MT-ATP6 PE=1 SV=1	3	1	1	24.8	4	226	10.1	1	1	1				1	1	1			
High	Q9NVM9	Integrator complex subunit 13 OS=Homo sapiens OX=9606 GN=INTS13 PE=1 SV=2	3	2	2	80.2	3	706	6.7					1						1	
High	Q13459	Unconventional myosin-IXb OS=Homo sapiens OX=9606 GN=MYO9B PE=1 SV=3	3	2	2	243.2	1	2157	8.75		1	1					1	1			
High	O60870	DNA/RNA-binding protein KIN17 OS=Homo sapiens OX=9606 GN=KIN PE=1 SV=2	3	1	1	45.3	2	393	8.95			1		1	1			1		1	1
High	Q969E8	Pre-rRNA-processing protein TSR2 homolog OS=Homo sapiens OX=9606 GN=TSR2 PE=1 SV=1	3	1	1	20.9	5	191	4.39				1						1		
High	P27707	Deoxycytidine kinase OS=Homo sapiens OX=9606 GN=DCK PE=1 SV=1	3	2	2	30.5	7	260	5.21				1	1					1	1	
High	Q13564	NEDD8-activating enzyme E1 regulatory subunit OS=Homo sapiens OX=9606 GN=NAE1 PE=1 SV=1	3	3	3	60.2	9	534	5.4				1						1		
High	Q15649	Zinc finger HIT domain-containing protein 3 OS=Homo sapiens OX=9606 GN=ZNHIT3 PE=1 SV=2	3	2	2	17.6	12	155	5.67						1						1
High	Q96JY6	PDZ and LIM domain protein 2 OS=Homo sapiens OX=9606 GN=PDLIM2 PE=1 SV=1	3	1	1	37.4	6	352	8.72												
High	Q13868	Exosome complex component RRP4 OS=Homo sapiens OX=9606 GN=EXOSC2 PE=1 SV=2	3	3	3	32.8	12	293	7.5												
High	Q92796	Disks large homolog 3 OS=Homo sapiens OX=9606 GN=DLG3 PE=1 SV=2	3	2	2	90.3	4	817	7.03												
High	O94979	Protein transport protein Sec31A OS=Homo sapiens OX=9606 GN=SEC31A PE=1 SV=3	3	1	1	132.9	1	1220	6.89												
High	P20585	DNA mismatch repair protein Msh3 OS=Homo sapiens OX=9606 GN=MSH3 PE=1 SV=4	3	1	1	127.3	1	1137	8.02				1		1				1		1
High	P29218	Inositol monophosphatase 1 OS=Homo sapiens OX=9606 GN=IMPA1 PE=1 SV=1	3	2	2	30.2	5	277	5.26												
High	Q8NB37	Glutamine amidotransferase-like class 1 domain-containing protein 1 OS=Homo sapiens OX=9606 GN=GATD1 PE=1 SV=1	3	2	2	23.3	14	220	6.61												
High	P20645	Cation-dependent mannose-6-phosphate receptor OS=Homo sapiens OX=9606 GN=M6PR PE=1 SV=1	3	1	1	31	5	277	5.83		1	1					1	1			
High	Q9H2W6	39S ribosomal protein L46, mitochondrial OS=Homo sapiens OX=9606 GN=MRPL46 PE=1 SV=1	3	2	2	31.7	12	279	7.05	1	1					1	1				
High	P09132	Signal recognition particle 19 kDa protein OS=Homo sapiens OX=9606 GN=SRP19 PE=1 SV=3	3	1	1	16.1	10	144	9.85			1	1		1			1	1		1
High	Q8NDA8	Maestro heat-like repeat-containing protein family member 1 OS=Homo sapiens OX=9606 GN=MROH1 PE=2 SV=3	3	2	2	181.1	1	1641	6.89	1		1				1		1			
High	Q5VYX0	Renalase OS=Homo sapiens OX=9606 GN=RNLS PE=1 SV=1	3	2	2	37.8	5	342	6.52					1						1	
High	Q2NKX8	DNA excision repair protein ERCC-6-like OS=Homo sapiens OX=9606 GN=ERCC6L PE=1 SV=1	3	1	1	141	1	1250	5.31	1						1					
High	P55001	Microfibrillar-associated protein 2 OS=Homo sapiens OX=9606 GN=MFAP2 PE=1 SV=1	3	1	1	20.8	5	183	4.97				1	1	1				1	1	1
High	O60879	Protein diaphanous homolog 2 OS=Homo sapiens OX=9606 GN=DIAPH2 PE=1 SV=1	3	1	1	125.5	1	1101	6.58												
High	Q8NCA5	Protein FAM98A OS=Homo sapiens OX=9606 GN=FAM98A PE=1 SV=2	3	3	3	55.2	10	518	8.95			1		1				1		1	
High	P51797	H(+)/Cl(-) exchange transporter 6 OS=Homo sapiens OX=9606 GN=CLCN6 PE=1 SV=2	3	1	1	97.2	1	869	6.81			1						1			
High	P15036	Protein C-ets-2 OS=Homo sapiens OX=9606 GN=ETS2 PE=1 SV=1	3	1	1	53	2	469	5.03												
High	P04156	Major prion protein OS=Homo sapiens OX=9606 GN=PRNP PE=1 SV=1	3	1	1	27.6	5	253	9												
High	Q5XG87	Terminal nucleotidyltransferase 4A OS=Homo sapiens OX=9606 GN=TENT4A PE=1 SV=3	2	1	1	82.3	1	772	9.52				1		1				1		1
High	P0DPB6	DNA-directed RNA polymerases I and III subunit RPAC2 OS=Homo sapiens OX=9606 GN=POLR1D PE=1 SV=1	2	2	2	15.2	8	133	5.8					1						1	
High	Q71RC2	La-related protein 4 OS=Homo sapiens OX=9606 GN=LARP4 PE=1 SV=3	2	1	1	80.5	3	724	6.61												
High	Q5T280	Putative methyltransferase C9orf114 OS=Homo sapiens OX=9606 GN=SPOUT1 PE=1 SV=3	2	1	1	42	4	376	7.43			1		1				1		1	
High	P10644	cAMP-dependent protein kinase type I-alpha regulatory subunit OS=Homo sapiens OX=9606 GN=PRKAR1A PE=1 SV=1	2	1	1	43	3	381	5.35	1	1					1	1				
High	Q9P1F3	Costars family protein ABRACL OS=Homo sapiens OX=9606 GN=ABRACL PE=1 SV=1	2	1	1	9.1	20	81	6.29	1	1					1	1				
High	Q9NR28	Diablo IAP-binding mitochondrial protein OS=Homo sapiens OX=9606 GN=DIABLO PE=1 SV=1	2	1	1	27.1	4	239	5.9												
High	Q99590	Protein SCAF11 OS=Homo sapiens OX=9606 GN=SCAF11 PE=1 SV=2	2	1	1	164.6	1	1463	8.41												
High	Q9NZC9	SWI/SNF-related matrix-associated actin-dependent regulator of chromatin subfamily A-like protein 1 OS=Homo sapiens OX=9606	2	2	2	105.9	2	954	9.06	1		1				1		1			
High	Q7Z2W9	39S ribosomal protein L21, mitochondrial OS=Homo sapiens OX=9606 GN=MRPL21 PE=1 SV=2	2	1	1	22.8	5	205	9.89												
High	Q8WW12	PEST proteolytic signal-containing nuclear protein OS=Homo sapiens OX=9606 GN=PCNP PE=1 SV=2	2	1	1	18.9	9	178	7.49			1						1			
High	Q9UHW5	GPN-loop GTPase 3 OS=Homo sapiens OX=9606 GN=GPN3 PE=1 SV=2	2	1	1	32.7	6	284	4.5		1	1					1	1			
High	Q8IZV5	Retinol dehydrogenase 10 OS=Homo sapiens OX=9606 GN=RDH10 PE=1 SV=1	2	1	1	38.1	5	341	7.4												
High	O15550	Lysine-specific demethylase 6A OS=Homo sapiens OX=9606 GN=KDM6A PE=1 SV=2	2	2	2	154.1	2	1401	7.44					1						1	
High	Q8N1P7	Beta/gamma crystallin domain-containing protein 2 OS=Homo sapiens OX=9606 GN=CRYBG2 PE=2 SV=2	2	1	1	177.8	1	1661	6.05		1						1				
High	Q8NF64	Zinc finger MIZ domain-containing protein 2 OS=Homo sapiens OX=9606 GN=ZMIZ2 PE=1 SV=2	2	2	2	96.5	2	920	7.12												
High	Q96EL3	39S ribosomal protein L53, mitochondrial OS=Homo sapiens OX=9606 GN=MRPL53 PE=1 SV=1	2	1	1	12.1	7	112	8.76												
High	O95707	Ribonuclease P protein subunit p29 OS=Homo sapiens OX=9606 GN=POP4 PE=1 SV=2	2	1	1	25.4	8	220	10.07												
High	Q13347	Eukaryotic translation initiation factor 3 subunit I OS=Homo sapiens OX=9606 GN=EIF3I PE=1 SV=1	2	2	2	36.5	6	325	5.64												
High	Q2M389	WASH complex subunit 4 OS=Homo sapiens OX=9606 GN=WASHC4 PE=1 SV=2	2	1	1	136.3	1	1173	7.44	1	1					1	1				
High	Q96DI7	U5 small nuclear ribonucleoprotein 40 kDa protein OS=Homo sapiens OX=9606 GN=SNRNP40 PE=1 SV=1	2	1	1	39.3	4	357	8.1												
High	O60658	High affinity cAMP-specific and IBMX-insensitive 3',5'-cyclic phosphodiesterase 8A OS=Homo sapiens OX=9606 GN=PDE8A PE=1 S\	2	1	1	93.2	2	829	6.11			1						1			
High	P05166	Propionyl-CoA carboxylase beta chain, mitochondrial OS=Homo sapiens OX=9606 GN=PCCB PE=1 SV=3	2	2	2	58.2	5	539	7.64	1						1					
High	Q96Q05	Trafficking protein particle complex subunit 9 OS=Homo sapiens OX=9606 GN=TRAPPC9 PE=1 SV=2	2	2	2	128.4	2	1148	6.62												
High	Q9UJW0	Dynactin subunit 4 OS=Homo sapiens OX=9606 GN=DCTN4 PE=1 SV=1	2	1	1	52.3	5	460	7.34			1						1			
High	O75110	Probable phospholipid-transporting ATPase IIA OS=Homo sapiens OX=9606 GN=ATP9A PE=1 SV=3	2	2	2	118.5	3	1047	7.77			1						1			

High	Q9Y4P8	WD repeat domain phosphoinositide-interacting protein 2 OS=Homo sapiens OX=9606 GN=WIPI2 PE=1 SV=1	2	1	1	49.4	4	454	6.46			1						1			
High	P36402	Transcription factor 7 OS=Homo sapiens OX=9606 GN=TCF7 PE=1 SV=3	2	1	1	41.6	3	384	6.81	1	1					1	1				
High	Q5VTL8	Pre-mRNA-splicing factor 38B OS=Homo sapiens OX=9606 GN=PRPF38B PE=1 SV=1	2	1	1	64.4	2	546	10.54				1		1				1		1
High	P07585	Decorin OS=Homo sapiens OX=9606 GN=DCN PE=1 SV=1	2	1	1	39.7	3	359	8.54				1		1				1		1
High	Q96SB3	Neurabin-2 OS=Homo sapiens OX=9606 GN=PPP1R9B PE=1 SV=3	2	2	2	89.3	3	817	4.97												
High	Q13617	Cullin-2 OS=Homo sapiens OX=9606 GN=CUL2 PE=1 SV=2	2	1	1	86.9	1	745	6.92												
High	P10253	Lysosomal alpha-glucosidase OS=Homo sapiens OX=9606 GN=GAA PE=1 SV=4	2	1	1	105.3	2	952	6	1	1					1	1				
High	Q53SF7	Cordon-bleu protein-like 1 OS=Homo sapiens OX=9606 GN=COBLL1 PE=1 SV=3	2	1	1	123.8	2	1128	6.58			1						1			
High	Q9NV96	Cell cycle control protein 50A OS=Homo sapiens OX=9606 GN=TMEM30A PE=1 SV=1	2	1	1	40.7	2	361	8.59												
High	Q9P2S5	WD repeat-containing protein WRAP73 OS=Homo sapiens OX=9606 GN=WRAP73 PE=1 SV=1	2	1	1	51.6	4	460	6.81												
High	Q15118	[Pyruvate dehydrogenase (acetyl-transferring)] kinase isozyme 1, mitochondrial OS=Homo sapiens OX=9606 GN=PDK1 PE=1 SV=1	2	2	2	49.2	6	436	8.81			1						1			
High	Q2NL82	Pre-rRNA-processing protein TSR1 homolog OS=Homo sapiens OX=9606 GN=TSR1 PE=1 SV=1	2	2	2	91.8	3	804	7.42												
High	P07225	Vitamin K-dependent protein S OS=Homo sapiens OX=9606 GN=PROS1 PE=1 SV=1	2	1	1	75.1	3	676	5.67		1						1				
High	Q14137	Ribosome biogenesis protein BOP1 OS=Homo sapiens OX=9606 GN=BOP1 PE=1 SV=2	2	2	2	83.6	6	746	6.19												
High	Q92989	Polyribonucleotide 5'-hydroxyl-kinase Clp1 OS=Homo sapiens OX=9606 GN=CLP1 PE=1 SV=1	2	2	2	47.6	4	425	6.62												
High	Q9P246	Stromal interaction molecule 2 OS=Homo sapiens OX=9606 GN=STIM2 PE=1 SV=2	2	2	2	83.9	4	746	6.76												
High	Q8NI60	Atypical kinase COQ8A, mitochondrial OS=Homo sapiens OX=9606 GN=COQ8A PE=1 SV=1	2	2	2	71.9	4	647	6.99			2						2			
High	Q86YZ3	SWISS-PROT:Q86YZ3 Tax_Id=9606 Gene_Symbol=HRNR Hornerin	2	1	1	282.2	2	2850	10.04					1						1	
High	Q9NPD8	Ubiquitin-conjugating enzyme E2 T OS=Homo sapiens OX=9606 GN=UBE2T PE=1 SV=1	2	2	2	22.5	13	197	7.99					1	1					1	1
High	Q9UHI6	Probable ATP-dependent RNA helicase DDX20 OS=Homo sapiens OX=9606 GN=DDX20 PE=1 SV=2	2	2	2	92.2	4	824	6.95	1						1					
High	P46783	40S ribosomal protein S10 OS=Homo sapiens OX=9606 GN=RPS10 PE=1 SV=1	2	1	1	18.9	5	165	10.15		1						1				
High	Q6IQ49	Replication stress response regulator SDE2 OS=Homo sapiens OX=9606 GN=SDE2 PE=1 SV=1	2	1	1	49.7	5	451	6.05												
High	Q5T3J3	Ligand-dependent nuclear receptor-interacting factor 1 OS=Homo sapiens OX=9606 GN=LRIF1 PE=1 SV=1	2	2	2	84.5	5	769	9.72												
High	Q9BZZ5	Apoptosis inhibitor 5 OS=Homo sapiens OX=9606 GN=API5 PE=1 SV=3	2	1	1	59	2	524	7.34						1						1
High	Q7Z6L1	Tectonin beta-propeller repeat-containing protein 1 OS=Homo sapiens OX=9606 GN=TECPR1 PE=1 SV=1	2	1	1	129.6	1	1165	6.24												
High	Q05932	Folylpolyglutamate synthase, mitochondrial OS=Homo sapiens OX=9606 GN=FPGS PE=1 SV=3	2	1	1	64.6	3	587	7.94			1						1			
High	Q5EBL8	PDZ domain-containing protein 11 OS=Homo sapiens OX=9606 GN=PDZD11 PE=1 SV=2	2	1	1	16.1	19	140	7.21	1	1					1	1				
High	Q9UHP3	Ubiquitin carboxyl-terminal hydrolase 25 OS=Homo sapiens OX=9606 GN=USP25 PE=1 SV=4	2	1	1	122.1	1	1055	5.34												
High	Q86U44	N6-adenosine-methyltransferase catalytic subunit OS=Homo sapiens OX=9606 GN=METTL3 PE=1 SV=2	2	1	1	64.4	2	580	6.42	1	1					1	1				
High	Q9H981	Actin-related protein 8 OS=Homo sapiens OX=9606 GN=ACTR8 PE=1 SV=2	2	1	1	70.4	1	624	7.78					1						1	
High	Q12765	Secernin-1 OS=Homo sapiens OX=9606 GN=SCRN1 PE=1 SV=2	2	1	1	46.4	4	414	4.75												
High	O75822	Eukaryotic translation initiation factor 3 subunit J OS=Homo sapiens OX=9606 GN=EIF3J PE=1 SV=2	2	2	2	29	9	258	4.83			1						1			
High	Q9NX00	Transmembrane protein 160 OS=Homo sapiens OX=9606 GN=TMEM160 PE=1 SV=1	2	1	1	19.6	4	188	8.03					1	1					1	1
High	Q7Z7H5	Transmembrane emp24 domain-containing protein 4 OS=Homo sapiens OX=9606 GN=TMED4 PE=1 SV=1	2	1	1	25.9	4	227	8.28	1	1					1	1				
High	Q96RF0	Sorting nexin-18 OS=Homo sapiens OX=9606 GN=SNX18 PE=1 SV=2	2	1	1	68.9	3	628	5.68												
High	P11766	Alcohol dehydrogenase class-3 OS=Homo sapiens OX=9606 GN=ADH5 PE=1 SV=4	2	1	1	39.7	4	374	7.49			1						1			
High	Q969F9	Hermansky-Pudlak syndrome 3 protein OS=Homo sapiens OX=9606 GN=HPS3 PE=1 SV=1	2	1	1	113.7	2	1004	6.43	1	1					1	1				
High	P51452	Dual specificity protein phosphatase 3 OS=Homo sapiens OX=9606 GN=DUSP3 PE=1 SV=1	2	2	2	20.5	12	185	7.8			1			1			1			1
High	Q15904	V-type proton ATPase subunit S1 OS=Homo sapiens OX=9606 GN=ATP6AP1 PE=1 SV=2	2	1	1	52	2	470	6.14												
High	P05109	Protein S100-A8 OS=Homo sapiens OX=9606 GN=S100A8 PE=1 SV=1	2	1	1	10.8	12	93	7.03				1	1					1	1	
High	O75044	SLIT-ROBO Rho GTPase-activating protein 2 OS=Homo sapiens OX=9606 GN=SRGAP2 PE=1 SV=3	2	2	2	120.8	3	1071	6.7		1						1				
High	P31271	Homeobox protein Hox-A13 OS=Homo sapiens OX=9606 GN=HOXA13 PE=1 SV=3	2	2	2	39.7	7	388	9.13	1						1					
High	P03886	NADH-ubiquinone oxidoreductase chain 1 OS=Homo sapiens OX=9606 GN=MT-ND1 PE=1 SV=1	2	1	1	35.6	3	318	6.55	1	1					1	1				
High	Q9HB40	Retinoid-inducible serine carboxypeptidase OS=Homo sapiens OX=9606 GN=SCPEP1 PE=1 SV=1	2	1	1	50.8	2	452	5.81	1		1				1		1			
High	Q66PJ3	ADP-ribosylation factor-like protein 6-interacting protein 4 OS=Homo sapiens OX=9606 GN=ARL6IP4 PE=1 SV=3	2	1	1	26.4	8	237	10.86												
High	Q9C002	Normal mucosa of esophagus-specific gene 1 protein OS=Homo sapiens OX=9606 GN=NMES1 PE=1 SV=1	2	2	2	9.6	28	83	9.47												
High	Q9NW82	WD repeat-containing protein 70 OS=Homo sapiens OX=9606 GN=WDR70 PE=1 SV=1	2	2	2	73.2	4	654	6.33												
High	Q99418	Cytohesin-2 OS=Homo sapiens OX=9606 GN=CYTH2 PE=1 SV=2	2	2	2	46.5	7	400	5.54			1			1			1			1
High	Q9BZL1	Ubiquitin-like protein 5 OS=Homo sapiens OX=9606 GN=UBL5 PE=1 SV=1	2	1	1	8.5	12	73	8.44	1	1					1	1				
High	Q13608	Peroxisome assembly factor 2 OS=Homo sapiens OX=9606 GN=PEX6 PE=1 SV=2	2	2	2	104	1	980	6.34												
High	Q9P031	Thyroid transcription factor 1-associated protein 26 OS=Homo sapiens OX=9606 GN=CCDC59 PE=1 SV=2	2	1	1	28.7	5	241	9.88												
High	Q9UM00	Calcium load-activated calcium channel OS=Homo sapiens OX=9606 GN=TMCO1 PE=1 SV=2	2	1	1	27.1	6	239	10.26	1	1					1	1				
High	P49459	Ubiquitin-conjugating enzyme E2 A OS=Homo sapiens OX=9606 GN=UBE2A PE=1 SV=2	2	1	1	17.3	7	152	5.15												
High	Q96QZ7	Membrane-associated guanylate kinase, WW and PDZ domain-containing protein 1 OS=Homo sapiens OX=9606 GN=MAGI1 PE=1 S	2	1	1	164.5	1	1491	7.58												
High	Q9P2W9	Syntaxin-18 OS=Homo sapiens OX=9606 GN=STX18 PE=1 SV=1	2	1	1	38.7	5	335	5.49		1	1					1	1			
High	O15254	Peroxisomal acyl-coenzyme A oxidase 3 OS=Homo sapiens OX=9606 GN=ACOX3 PE=1 SV=2	2	1	1	77.6	3	700	7.25												
High	P42765	3-ketoacyl-CoA thiolase, mitochondrial OS=Homo sapiens OX=9606 GN=ACAA2 PE=1 SV=2	2	1	1	41.9	2	397	8.09		1	1					1	1			
High	Q9UQR1	Zinc finger protein 148 OS=Homo sapiens OX=9606 GN=ZNF148 PE=1 SV=2	2	2	2	88.9	2	794	6.48	1		1				1		1			
High	Q9Y6W3	Calpain-7 OS=Homo sapiens OX=9606 GN=CAPN7 PE=1 SV=1	2	1	1	92.6	1	813	7.65	1	1					1	1				
High	P10606	Cytochrome c oxidase subunit 5B, mitochondrial OS=Homo sapiens OX=9606 GN=COX5B PE=1 SV=2	2	1	1	13.7	6	129	8.81												
High	Q13751	Laminin subunit beta-3 OS=Homo sapiens OX=9606 GN=LAMB3 PE=1 SV=1	2	2	2	129.5	2	1172	7.21												
High	O60306	RNA helicase aquarius OS=Homo sapiens OX=9606 GN=AQR PE=1 SV=4	2	2	2	171.2	2	1485	6.37					1						1	
High	Q8N0Z8	tRNA pseudouridine synthase-like 1 OS=Homo sapiens OX=9606 GN=PUSL1 PE=1 SV=1	2	1	1	33.2	3	303	9.85		1						1				
High	O15321	Transmembrane 9 superfamily member 1 OS=Homo sapiens OX=9606 GN=TM9SF1 PE=2 SV=2	2	1	1	68.8	1	606	7.17	1	1					1	1				
High	P19532	Transcription factor E3 OS=Homo sapiens OX=9606 GN=TFE3 PE=1 SV=4	2	2	2	61.5	3	575	5.58		1	1					1	1			
High	Q13642	Four and a half LIM domains protein 1 OS=Homo sapiens OX=9606 GN=FHL1 PE=1 SV=4	2	1	1	36.2	4	323	8.97				1		1				1		1
High	Q9BV68	E3 ubiquitin-protein ligase RNF126 OS=Homo sapiens OX=9606 GN=RNF126 PE=1 SV=2	2	1	1	33.8	8	311	5.47												
High	Q969X1	Protein lifeguard 3 OS=Homo sapiens OX=9606 GN=TMBIM1 PE=1 SV=2	2	1	1	34.6	5	311	7.72	1		1				1		1			
High	Q9H2H8	Peptidyl-prolyl cis-trans isomerase-like 3 OS=Homo sapiens OX=9606 GN=PPIL3 PE=1 SV=1	2	1	1	18.1	12	161	6.79	1						1					
High	O75691	Small subunit processome component 20 homolog OS=Homo sapiens OX=9606 GN=UTP20 PE=1 SV=3	2	2	2	318.2	1	2785	7.39												
High	O43504	Ragulator complex protein LAMTOR5 OS=Homo sapiens OX=9606 GN=LAMTOR5 PE=1 SV=1	2	1	1	9.6	26	91	4.87												
High	Q9P227	Rho GTPase-activating protein 23 OS=Homo sapiens OX=9606 GN=ARHGAP23 PE=1 SV=2	2	1	1	162.1	1	1491	9.19												
High	P11047	Laminin subunit gamma-1 OS=Homo sapiens OX=9606 GN=LAMC1 PE=1 SV=3	2	1	1	177.5	1	1609	5.12					1	1					1	1
High	P54098	DNA polymerase subunit gamma-1 OS=Homo sapiens OX=9606 GN=POLG PE=1 SV=1	2	1	1	139.5	1	1239	6.9			1						1			
High	P46976	Glycogenin-1 OS=Homo sapiens OX=9606 GN=GYG1 PE=1 SV=4	2	2	2	39.4	7	350	5.53												
High	P61457	Pterin-4-alpha-carbinolamine dehydratase OS=Homo sapiens OX=9606 GN=PCBD1 PE=1 SV=2	2	1	1	12	10	104	6.8	1						1					
High	P07477	SWISS-PROT:P07477 Tax_Id=9606 Gene_Symbol=PRSS1 Trypsin-1 precursor	2	1	1	26.5	8	247	6.51	1						1					
High	Q8N594	MPN domain-containing protein OS=Homo sapiens OX=9606 GN=MPND PE=1 SV=1	2	2	2	50.6	6	471	5.72		1	1					1	1			
High	P51159	Ras-related protein Rab-27A OS=Homo sapiens OX=9606 GN=RAB27A PE=1 SV=3	2	2	2	24.9	8	221	5.22												
High	Q8WU90	Zinc finger CCCH domain-containing protein 15 OS=Homo sapiens OX=9606 GN=ZC3H15 PE=1 SV=1	2	2	2	48.6	6	426	5.31					1						1	
High	O75717	WD repeat and HMG-box DNA-binding protein 1 OS=Homo sapiens OX=9606 GN=WDHD1 PE=1 SV=1	2	1	1	125.9	1	1129	5.62												
High	O43929	Origin recognition complex subunit 4 OS=Homo sapiens OX=9606 GN=ORC4 PE=1 SV=2	2	2	2	50.3	6	436	8	1	1					1	1				
High	O60664	Perilipin-3 OS=Homo sapiens OX=9606 GN=PLIN3 PE=1 SV=3	2	1	1	47	4	434	5.44	1	1					1	1				
High	Q15828	Cystatin-M OS=Homo sapiens OX=9606 GN=CST6 PE=1 SV=1	2	1	1	16.5	7	149	8.09				1		1				1		1
High	Q9Y572	Receptor-interacting serine/threonine-protein kinase 3 OS=Homo sapiens OX=9606 GN=RIPK3 PE=1 SV=2	2	1	1	56.9	4	518	6.47			1						1			
High	Q04446	1,4-alpha-glucan-branching enzyme OS=Homo sapiens OX=9606 GN=GBE1 PE=1 SV=3	2	1	1	80.4	1	702	6.32												
High	Q9BVG9	Phosphatidylserine synthase 2 OS=Homo sapiens OX=9606 GN=PTDSS2 PE=1 SV=1	2	1	1	56.2	5	487	6.25	1		1				1		1			
High	Q99797	Mitochondrial intermediate peptidase OS=Homo sapiens OX=9606 GN=MIPEP PE=1 SV=2	2	1	1	80.6	1	713	7.05												
High	Q99943	1-acyl-sn-glycerol-3-phosphate acyltransferase alpha OS=Homo sapiens OX=9606 GN=AGPAT1 PE=1 SV=2	2	1	1	31.7	7	283	9.38												
High	P54792	Putative segment polarity protein dishevelled homolog DVL1P1 OS=Homo sapiens OX=9606 GN=DVL1P1 PE=5 SV=1	2	1	1	73.2	1	670	6.46					1	1					1	1
High	P03928	ATP synthase protein 8 OS=Homo sapiens OX=9606 GN=MT-ATP8 PE=1 SV=1	2	1	1	8	13	68	9.91			2						1			
High	Q9HB58	Sp110 nuclear body protein OS=Homo sapiens OX=9606 GN=SP110 PE=1 SV=5	2	2	2	78.3	3	689	8.87						1						1
High	Q8IY18	Structural maintenance of chromosomes protein 5 OS=Homo sapiens OX=9606 GN=SMC5 PE=1 SV=2	2	1	1	128.7	1	1101	8.38												
High	P17174	Aspartate aminotransferase, cytoplasmic OS=Homo sapiens OX=9606 GN=GOT1 PE=1 SV=3	2	1	1	46.2	2	413	7.01			2						1			
High	P53990	IST1 homolog OS=Homo sapiens OX=9606 GN=IST1 PE=1 SV=1	2	2	2	39.7	8	364	5.35												
High	P61073	C-X-C chemokine receptor type 4 OS=Homo sapiens OX=9606 GN=CXCR4 PE=1 SV=1	2	1	1	39.7	3	352	8.21	1		1				1		1			
High	P51809	Vesicle-associated membrane protein 7 OS=Homo sapiens OX=9606 GN=VAMP7 PE=1 SV=3	2	1	1	24.9	5	220	8.6	1	1					1	1				
High	Q9Y3X0	Coiled-coil domain-containing protein 9 OS=Homo sapiens OX=9606 GN=CCDC9 PE=1 SV=1	2	2	2	59.7	4	531	5.4		1		1				1		1		
High	Q8NEW0	Zinc transporter 7 OS=Homo sapiens OX=9606 GN=SLC30A7 PE=2 SV=1	2	1	1	41.6	4	376	6.95	1	1					1	1				
High	Q92908	Transcription factor GATA-6 OS=Homo sapiens OX=9606 GN=GATA6 PE=1 SV=2	2	1	1	60	2	595	8.35												
High	Q96GC9	Vacuole membrane protein 1 OS=Homo sapiens OX=9606 GN=VMP1 PE=1 SV=1	2	1	1	46.2	2	406	6.95												
High	Q9Y2Y1	DNA-directed RNA polymerase III subunit RPC10 OS=Homo sapiens OX=9606 GN=POLR3K PE=1 SV=3	2	1	1	12.3	13	108	7.83												
High	Q14644	Ras GTPase-activating protein 3 OS=Homo sapiens OX=9606 GN=RASA3 PE=1 SV=3	2	2	2	95.6	2	834	7.15					1						1	
High	Q9BQE5	Apolipoprotein L2 OS=Homo sapiens OX=9606 GN=APOL2 PE=1 SV=1	2	1	1	37.1	3	337	6.74												
High	Q9Y679	Lipid droplet-regulating VLDL assembly factor AUP1 OS=Homo sapiens OX=9606 GN=AUP1 PE=1 SV=2	2	2	2	45.8	6	410	8.65	1						1					
High	Q7Z404	Transmembrane channel-like protein 4 OS=Homo sapiens OX=9606 GN=TMC4 PE=1 SV=3	2	1	1	79.2	2	712	8.94			1						1			
High	Q8NBT2	Kinetochore protein Spc24 OS=Homo sapiens OX=9606 GN=SPC24 PE=1 SV=3	2	1	1	22.4	7	197	4.7	1	1					1	1				
High	Q9Y4P1	Cysteine protease ATG4B OS=Homo sapiens OX=9606 GN=ATG4B PE=1 SV=2	2	2	2	44.3	8	393	5.07												
High	Q5T0N5	Formin-binding protein 1-like OS=Homo sapiens OX=9606 GN=FNBP1L PE=1 SV=3	2	1	1	70	1	605	6.64			1						1			
High	O95786	Antiviral innate immune response receptor RIG-I OS=Homo sapiens OX=9606 GN=DDX58 PE=1 SV=2	2	1	1	106.5	1	925	6.4					1						1	
High	Q07654	Trefoil factor 3 OS=Homo sapiens OX=9606 GN=TFF3 PE=1 SV=3	2	1	1	8.6	14	80	5.92												
High	Q9Y2Q9	28S ribosomal protein S28, mitochondrial OS=Homo sapiens OX=9606 GN=MRPS28 PE=1 SV=1	2	2	2	20.8	11	187	9.1						1						1
High	P53611	Geranylgeranyl transferase type-2 subunit beta OS=Homo sapiens OX=9606 GN=RABGGTB PE=1 SV=2	2	1	1	36.9	3	331	5.03				1		1				1		1
High	Q4KMP7	TBC1 domain family member 10B OS=Homo sapiens OX=9606 GN=TBC1D10B PE=1 SV=3	2	1	1	87.1	3	808	9.19												
High	P55210	Caspase-7 OS=Homo sapiens OX=9606 GN=CASP7 PE=1 SV=1	2	1	1	34.3	8	303	6.07				1						1		
High	P06746	DNA polymerase beta OS=Homo sapiens OX=9606 GN=POLB PE=1 SV=3	2	1	1	38.2	3	335	8.95				1		1				1		1
High	O14732	Inositol monophosphatase 2 OS=Homo sapiens OX=9606 GN=IMPA2 PE=1 SV=1	2	2	2	31.3	8	288	6.61				1						1		
High	Q9NPI1	Bromodomain-containing protein 7 OS=Homo sapiens OX=9606 GN=BRD7 PE=1 SV=1	2	2	2	74.1	6	651	6.39												
High	Q13469	Nuclear factor of activated T-cells, cytoplasmic 2 OS=Homo sapiens OX=9606 GN=NFATC2 PE=1 SV=2	2	1	1	100.1	2	925	7.28	1	1					1	1				
High	Q9NZM3	Intersectin-2 OS=Homo sapiens OX=9606 GN=ITSN2 PE=1 SV=3	2	1	1	193.3	1	1697	8.12												
High	Q8WWY3	U4/U6 small nuclear ribonucleoprotein Prp31 OS=Homo sapiens OX=9606 GN=PRPF31 PE=1 SV=2	2	2	2	55.4	7	499	5.78												
High	Q6Q0C0	E3 ubiquitin-protein ligase TRAF7 OS=Homo sapiens OX=9606 GN=TRAF7 PE=1 SV=1	2	1	1	74.6	1	670	7.15				1		1				1		1
High	P19075	Tetraspanin-8 OS=Homo sapiens OX=9606 GN=TSPAN8 PE=1 SV=1	2	1	1	26	4	237	5.6												
High	Q14978	Nucleolar and coiled-body phosphoprotein 1 OS=Homo sapiens OX=9606 GN=NOLC1 PE=1 SV=2	2	1	1	73.6	1	699	9.47					1	1					1	1
High	Q2PZI1	Probable C-mannosyltransferase DPY19L1 OS=Homo sapiens OX=9606 GN=DPY19L1 PE=2 SV=1	2	1	1	77.3	3	675	8.95			1						1			
High	Q9BPX6	Calcium uptake protein 1, mitochondrial OS=Homo sapiens OX=9606 GN=MICU1 PE=1 SV=1	2	1	1	54.3	3	476	8.41			1						1			
High	O14966	Ras-related protein Rab-7L1 OS=Homo sapiens OX=9606 GN=RAB29 PE=1 SV=1	2	1	1	23.1	5	203	7.18												
High	P51790	H(+)/Cl(-) exchange transporter 3 OS=Homo sapiens OX=9606 GN=CLCN3 PE=1 SV=2	2	2	2	90.9	2	818	6.28		1	1					1	1			
High	Q9BZK3	Putative nascent polypeptide-associated complex subunit alpha-like protein OS=Homo sapiens OX=9606 GN=NACA4P PE=5 SV=1	2	1	1	23.3	7	213	4.59												
High	Q9BVM2	Protein DPCD OS=Homo sapiens OX=9606 GN=DPCD PE=1 SV=2	2	1	1	23.2	4	203	9.03												
High	Q5C9Z4	Nucleolar MIF4G domain-containing protein 1 OS=Homo sapiens OX=9606 GN=NOM1 PE=1 SV=1	2	1	1	96.2	1	860	8.1												
High	P53567	CCAAT/enhancer-binding protein gamma OS=Homo sapiens OX=9606 GN=CEBPG PE=1 SV=1	2	1	1	16.4	21	150	9.77	1	1					1	1				
High	O15169	Axin-1 OS=Homo sapiens OX=9606 GN=AXIN1 PE=1 SV=2	2	1	1	95.6	2	862	6.96												
High	O94913	Pre-mRNA cleavage complex 2 protein Pcf11 OS=Homo sapiens OX=9606 GN=PCF11 PE=1 SV=3	2	1	1	172.9	1	1555	8.48												
High	Q8N3U4	Cohesin subunit SA-2 OS=Homo sapiens OX=9606 GN=STAG2 PE=1 SV=3	2	2	2	141.2	2	1231	5.43	1	1					1	1				
High	Q15005	Signal peptidase complex subunit 2 OS=Homo sapiens OX=9606 GN=SPCS2 PE=1 SV=3	2	2	2	25	8	226	8.47	1						1					
High	Q6IQ23	Pleckstrin homology domain-containing family A member 7 OS=Homo sapiens OX=9606 GN=PLEKHA7 PE=1 SV=2	2	2	2	127.1	2	1121	9.35			1						1			
High	Q02809	Procollagen-lysine,2-oxoglutarate 5-dioxygenase 1 OS=Homo sapiens OX=9606 GN=PLOD1 PE=1 SV=2	2	1	1	83.5	2	727	6.95			1						1			
High	Q9BQP7	Mitochondrial genome maintenance exonuclease 1 OS=Homo sapiens OX=9606 GN=MGME1 PE=1 SV=1	2	1	1	39.4	6	344	7.68					1						1	
High	O95155	Ubiquitin conjugation factor E4 B OS=Homo sapiens OX=9606 GN=UBE4B PE=1 SV=1	2	1	1	146.1	1	1302	6.55	1		1				1		1			
High	Q9BQC6	Ribosomal protein 63, mitochondrial OS=Homo sapiens OX=9606 GN=MRPL57 PE=1 SV=1	2	1	1	12.3	13	102	11.44			1						1			
High	Q9NZM5	Ribosome biogenesis protein NOP53 OS=Homo sapiens OX=9606 GN=NOP53 PE=1 SV=2	2	1	1	54.4	4	478	10.32												
High	Q92541	RNA polymerase-associated protein RTF1 homolog OS=Homo sapiens OX=9606 GN=RTF1 PE=1 SV=4	2	2	2	80.3	4	710	8.15												
High	P55809	Succinyl-CoA:3-ketoacid coenzyme A transferase 1, mitochondrial OS=Homo sapiens OX=9606 GN=OXCT1 PE=1 SV=1	2	1	1	56.1	6	520	7.46					2						1	
High	Q12788	Transducin beta-like protein 3 OS=Homo sapiens OX=9606 GN=TBL3 PE=1 SV=2	2	1	1	89	2	808	6.9					1						1	
High	Q96CM8	Medium-chain acyl-CoA ligase ACSF2, mitochondrial OS=Homo sapiens OX=9606 GN=ACSF2 PE=1 SV=2	2	1	1	68.1	3	615	7.55			1						1			
High	Q5T0D9	Tumor protein p63-regulated gene 1-like protein OS=Homo sapiens OX=9606 GN=TPRG1L PE=1 SV=1	2	1	1	30.2	7	272	7.34	1	1					1	1				
High	Q6ZNJ1	Neurobeachin-like protein 2 OS=Homo sapiens OX=9606 GN=NBEAL2 PE=1 SV=2	2	1	1	302.3	1	2754	6.38			1						1			
High	P32121	Beta-arrestin-2 OS=Homo sapiens OX=9606 GN=ARRB2 PE=1 SV=2	2	1	1	46.1	4	409	7.69					1						1	
High	Q02040	A-kinase anchor protein 17A OS=Homo sapiens OX=9606 GN=AKAP17A PE=1 SV=2	2	2	2	80.7	3	695	9.73		1						1				
High	Q9BQ13	BTB/POZ domain-containing protein KCTD14 OS=Homo sapiens OX=9606 GN=KCTD14 PE=1 SV=2	2	1	1	29.6	5	255	8.59												
High	P13569	Cystic fibrosis transmembrane conductance regulator OS=Homo sapiens OX=9606 GN=CFTR PE=1 SV=3	2	1	1	168	1	1480	8.73		1	1					1	1			
High	Q13144	Translation initiation factor eIF-2B subunit epsilon OS=Homo sapiens OX=9606 GN=EIF2B5 PE=1 SV=3	2	2	2	80.3	4	721	5.08	1						1					
High	P08582	Melanotransferrin OS=Homo sapiens OX=9606 GN=MELTF PE=1 SV=2	2	1	1	80.2	3	738	5.94												
High	P07451	Carbonic anhydrase 3 OS=Homo sapiens OX=9606 GN=CA3 PE=1 SV=3	2	1	1	29.5	3	260	7.34				1		1				1		1
High	Q9NWW5	Ceroid-lipofuscinosis neuronal protein 6 OS=Homo sapiens OX=9606 GN=CLN6 PE=1 SV=1	2	1	1	35.9	7	311	8.87												
High	Q96FS4	Signal-induced proliferation-associated protein 1 OS=Homo sapiens OX=9606 GN=SIPA1 PE=1 SV=1	2	1	1	112.1	1	1042	6.6			1	1					1	1		
High	Q92625	Ankyrin repeat and SAM domain-containing protein 1A OS=Homo sapiens OX=9606 GN=ANKS1A PE=1 SV=4	2	1	1	123	2	1134	6.38	1	1					1	1				
High	Q9BW71	HIRA-interacting protein 3 OS=Homo sapiens OX=9606 GN=HIRIP3 PE=1 SV=3	2	1	1	61.9	2	556	8.54												
High	Q9H1I8	Activating signal cointegrator 1 complex subunit 2 OS=Homo sapiens OX=9606 GN=ASCC2 PE=1 SV=3	2	1	1	86.3	2	757	5.16	1						1					
High	O60281	Zinc finger protein 292 OS=Homo sapiens OX=9606 GN=ZNF292 PE=1 SV=3	2	2	2	304.6	1	2723	7.39												
High	P63098	Calcineurin subunit B type 1 OS=Homo sapiens OX=9606 GN=PPP3R1 PE=1 SV=2	2	1	1	19.3	5	170	4.81			1						1			
High	Q9NTZ6	RNA-binding protein 12 OS=Homo sapiens OX=9606 GN=RBM12 PE=1 SV=1	2	1	1	97.3	1	932	8.63					1						1	
High	Q6UVK1	Chondroitin sulfate proteoglycan 4 OS=Homo sapiens OX=9606 GN=CSPG4 PE=1 SV=2	2	1	1	250.4	1	2322	5.47	1		1				1		1			
High	Q6ZVM7	TOM1-like protein 2 OS=Homo sapiens OX=9606 GN=TOM1L2 PE=1 SV=1	2	2	2	55.5	6	507	4.79			1						1			
High	P02794	Ferritin heavy chain OS=Homo sapiens OX=9606 GN=FTH1 PE=1 SV=2	2	1	1	21.2	4	183	5.55				1		1				1		1
High	Q13867	Bleomycin hydrolase OS=Homo sapiens OX=9606 GN=BLMH PE=1 SV=1	2	2	2	52.5	5	455	6.27			2						2			
High	Q8TEX9	Importin-4 OS=Homo sapiens OX=9606 GN=IPO4 PE=1 SV=2	2	1	1	118.6	2	1081	4.96												
High	Q9BTD8	RNA-binding protein 42 OS=Homo sapiens OX=9606 GN=RBM42 PE=1 SV=1	2	1	1	50.4	4	480	9.63												

High	Q5VZ89	DENN domain-containing protein 4C OS=Homo sapiens OX=9606 GN=DENND4C PE=1 SV=3	2	1	1	212.6	0	1909	6.83												
High	P55081	Microfibrillar-associated protein 1 OS=Homo sapiens OX=9606 GN=MFAP1 PE=1 SV=2	2	1	1	51.9	2	439	4.98				1		1				1		1
High	Q9UDY4	DnaJ homolog subfamily B member 4 OS=Homo sapiens OX=9606 GN=DNAJB4 PE=1 SV=1	2	2	1	37.8	8	337	8.5												
High	P51617	Interleukin-1 receptor-associated kinase 1 OS=Homo sapiens OX=9606 GN=IRAK1 PE=1 SV=2	2	1	1	76.5	2	712	6.62		1	1					1	1			
High	Q969Z0	FAST kinase domain-containing protein 4 OS=Homo sapiens OX=9606 GN=TBRG4 PE=1 SV=1	2	2	2	70.7	4	631	7.42	1	1					1	1				
High	O75962	Triple functional domain protein OS=Homo sapiens OX=9606 GN=TRIO PE=1 SV=2	2	1	1	346.7	0	3097	6.37					1						1	
High	O95602	DNA-directed RNA polymerase I subunit RPA1 OS=Homo sapiens OX=9606 GN=POLR1A PE=1 SV=2	2	1	1	194.7	1	1720	7.03												
High	Q2TAY7	WD40 repeat-containing protein SMU1 OS=Homo sapiens OX=9606 GN=SMU1 PE=1 SV=2	2	2	2	57.5	4	513	7.18					1						1	
High	P21817	Ryanodine receptor 1 OS=Homo sapiens OX=9606 GN=RYR1 PE=1 SV=3	2	2	2	564.8	1	5038	5.3		1	1					1	1			
High	Q9BRG2	SH2 domain-containing protein 3A OS=Homo sapiens OX=9606 GN=SH2D3A PE=1 SV=1	2	2	2	63.1	6	576	7.42			1						1			
High	Q03405	Urokinase plasminogen activator surface receptor OS=Homo sapiens OX=9606 GN=PLAUR PE=1 SV=1	2	2	2	37	10	335	6.65		1						1				
High	Q9P0U3	Sentrin-specific protease 1 OS=Homo sapiens OX=9606 GN=SENP1 PE=1 SV=2	2	1	1	73.4	1	644	8.47												
High	Q6UX65	DNA damage-regulated autophagy modulator protein 2 OS=Homo sapiens OX=9606 GN=DRAM2 PE=1 SV=1	2	1	1	29.7	8	266	8.03	1	1					1	1				
High	Q16651	Prostasin OS=Homo sapiens OX=9606 GN=PRSS8 PE=1 SV=1	2	1	1	36.4	4	343	5.85												
High	Q5BJD5	Transmembrane protein 41B OS=Homo sapiens OX=9606 GN=TMEM41B PE=1 SV=1	2	1	1	32.5	7	291	9.58	2						1					
High	Q9Y291	28S ribosomal protein S33, mitochondrial OS=Homo sapiens OX=9606 GN=MRPS33 PE=1 SV=1	2	1	1	12.6	8	106	10.11												
High	C9JLW8	Mapk-regulated corepressor-interacting protein 1 OS=Homo sapiens OX=9606 GN=MCRIP1 PE=1 SV=1	2	1	1	10.9	9	97	9.41				1		1				1		1
High	Q9UH99	SUN domain-containing protein 2 OS=Homo sapiens OX=9606 GN=SUN2 PE=1 SV=3	2	1	1	80.3	1	717	6.73			1						1			
High	Q15633	RISC-loading complex subunit TARBP2 OS=Homo sapiens OX=9606 GN=TARBP2 PE=1 SV=3	2	1	1	39	4	366	6.54												
High	Q69YN4	Protein virilizer homolog OS=Homo sapiens OX=9606 GN=VIRMA PE=1 SV=2	2	1	1	201.9	0	1812	5.01												
High	Q9UKJ3	G patch domain-containing protein 8 OS=Homo sapiens OX=9606 GN=GPATCH8 PE=1 SV=2	2	1	1	164.1	1	1502	8.66												
High	P37287	Phosphatidylinositol N-acetylglucosaminyltransferase subunit A OS=Homo sapiens OX=9606 GN=PIGA PE=1 SV=1	2	1	1	54.1	2	484	8.34												
High	Q99504	Eyes absent homolog 3 OS=Homo sapiens OX=9606 GN=EYA3 PE=1 SV=3	2	1	1	62.6	2	573	5.21			1						1			
High	Q96JB5	CDK5 regulatory subunit-associated protein 3 OS=Homo sapiens OX=9606 GN=CDK5RAP3 PE=1 SV=2	2	1	1	56.9	3	506	4.75												
High	Q14CX7	N-alpha-acetyltransferase 25, NatB auxiliary subunit OS=Homo sapiens OX=9606 GN=NAA25 PE=1 SV=1	2	1	1	112.2	1	972	6.64			1						1			
High	Q15596	Nuclear receptor coactivator 2 OS=Homo sapiens OX=9606 GN=NCOA2 PE=1 SV=2	2	2	2	159.1	3	1464	6.64												
High	Q9BUB7	Transmembrane protein 70, mitochondrial OS=Homo sapiens OX=9606 GN=TMEM70 PE=1 SV=2	2	1	1	29	4	260	8.91	1	1					1	1				
High	Streptavid	(S.avidinii)	2	1	1	16.6	12	160	6.55	1				1		1				1	
High	O94763	Unconventional prefoldin RPB5 interactor 1 OS=Homo sapiens OX=9606 GN=URI1 PE=1 SV=3	2	2	2	59.8	8	535	5.05												
High	Q01469	Fatty acid-binding protein 5 OS=Homo sapiens OX=9606 GN=FABP5 PE=1 SV=3	2	1	1	15.2	7	135	7.01				1		1				1		1
High	Q86TB9	Protein PAT1 homolog 1 OS=Homo sapiens OX=9606 GN=PATL1 PE=1 SV=2	2	2	2	86.8	2	770	6.67					1						1	
High	P09960	Leukotriene A-4 hydrolase OS=Homo sapiens OX=9606 GN=LTA4H PE=1 SV=2	2	1	1	69.2	2	611	6.18												
High	Q9GZL7	Ribosome biogenesis protein WDR12 OS=Homo sapiens OX=9606 GN=WDR12 PE=1 SV=2	2	1	1	47.7	2	423	5.9												
High	Q9H9P8	L-2-hydroxyglutarate dehydrogenase, mitochondrial OS=Homo sapiens OX=9606 GN=L2HGDH PE=1 SV=3	2	1	1	50.3	2	463	8.15												
High	Q96C57	Protein CUSTOS OS=Homo sapiens OX=9606 GN=CUSTOS PE=1 SV=2	2	1	1	28.2	7	262	9.42	1	1					1	1				
High	Q9H840	Gem-associated protein 7 OS=Homo sapiens OX=9606 GN=GEMIN7 PE=1 SV=1	2	1	1	14.5	10	131	7.4												
High	O60232	Protein ZNRD2 OS=Homo sapiens OX=9606 GN=ZNRD2 PE=1 SV=1	2	1	1	21.5	4	199	5.24												
High	Q53EZ4	Centrosomal protein of 55 kDa OS=Homo sapiens OX=9606 GN=CEP55 PE=1 SV=3	2	2	2	54.1	5	464	7.01			1						1			
High	Q9HD47	Ran guanine nucleotide release factor OS=Homo sapiens OX=9606 GN=RANGRF PE=1 SV=1	2	1	1	20.4	7	186	4.94												
High	Q9NVD7	Alpha-parvin OS=Homo sapiens OX=9606 GN=PARVA PE=1 SV=1	2	1	1	42.2	4	372	5.95												
High	Q07065	Cytoskeleton-associated protein 4 OS=Homo sapiens OX=9606 GN=CKAP4 PE=1 SV=2	2	1	1	66	3	602	5.92												
High	Q96NT0	Coiled-coil domain-containing protein 115 OS=Homo sapiens OX=9606 GN=CCDC115 PE=1 SV=1	2	1	1	19.7	8	180	6.95		1						1				
High	Q8NFA0	Ubiquitin carboxyl-terminal hydrolase 32 OS=Homo sapiens OX=9606 GN=USP32 PE=1 SV=1	2	1	1	181.5	1	1604	6.44			1						1			
High	A1KXE4	Myelin-associated neurite-outgrowth inhibitor OS=Homo sapiens OX=9606 GN=FAM168B PE=1 SV=1	2	1	1	20.3	9	195	9.23				1		1				1		1
High	P17275	Transcription factor JunB OS=Homo sapiens OX=9606 GN=JUNB PE=1 SV=1	2	1	1	35.9	2	347	9.22						1						1
High	P49354	Protein farnesyltransferase/geranylgeranyltransferase type-1 subunit alpha OS=Homo sapiens OX=9606 GN=FNTA PE=1 SV=1	2	1	1	44.4	3	379	5.08		1						1				
High	P67812	Signal peptidase complex catalytic subunit SEC11A OS=Homo sapiens OX=9606 GN=SEC11A PE=1 SV=1	2	1	1	20.6	7	179	9.48	1	1					1	1				
High	Q9H490	Phosphatidylinositol glycan anchor biosynthesis class U protein OS=Homo sapiens OX=9606 GN=PIGU PE=1 SV=3	2	1	1	50	3	435	7.72												
High	P00711	SWISS-PROT:P00711 Alpha-lactalbumin - Bos taurus (Bovine).	2	1	1	14.2	14	123	5						1						1
High	Q9BRQ6	MICOS complex subunit MIC25 OS=Homo sapiens OX=9606 GN=CHCHD6 PE=1 SV=1	2	1	1	26.4	5	235	8.85	1		1				1		1			
High	Q9H8Y5	Ankyrin repeat and zinc finger domain-containing protein 1 OS=Homo sapiens OX=9606 GN=ANKZF1 PE=1 SV=1	2	2	2	80.9	3	726	8.41												
High	Q9Y388	RNA-binding motif protein, X-linked 2 OS=Homo sapiens OX=9606 GN=RBMX2 PE=1 SV=2	2	2	2	37.3	7	322	9.83					2						2	
High	P60604	Ubiquitin-conjugating enzyme E2 G2 OS=Homo sapiens OX=9606 GN=UBE2G2 PE=1 SV=1	2	1	1	18.6	13	165	4.7	1	1					1	1				
High	Q7Z624	Calmodulin-lysine N-methyltransferase OS=Homo sapiens OX=9606 GN=CAMKMT PE=1 SV=2	1	1	1	36.1	4	323	6.83												
High	P12757	Ski-like protein OS=Homo sapiens OX=9606 GN=SKIL PE=1 SV=2	1	1	1	76.9	2	684	7.11												
High	Q86U28	Iron-sulfur cluster assembly 2 homolog, mitochondrial OS=Homo sapiens OX=9606 GN=ISCA2 PE=1 SV=2	1	1	1	16.5	8	154	5.25												
High	Q9ULM3	YEATS domain-containing protein 2 OS=Homo sapiens OX=9606 GN=YEATS2 PE=1 SV=2	1	1	1	150.7	1	1422	8.98												
High	O43166	Signal-induced proliferation-associated 1-like protein 1 OS=Homo sapiens OX=9606 GN=SIPA1L1 PE=1 SV=4	1	1	1	199.9	0	1804	8.19												
High	Q86YV5	Inactive tyrosine-protein kinase PRAG1 OS=Homo sapiens OX=9606 GN=PRAG1 PE=1 SV=4	1	1	1	149.5	1	1406	7.2												
High	P27695	DNA-(apurinic or apyrimidinic site) endonuclease OS=Homo sapiens OX=9606 GN=APEX1 PE=1 SV=2	1	1	1	35.5	7	318	8.12					1						1	
High	Q7L5Y9	E3 ubiquitin-protein transferase MAEA OS=Homo sapiens OX=9606 GN=MAEA PE=1 SV=1	1	1	1	45.3	2	396	8.69					1						1	
High	P15428	15-hydroxyprostaglandin dehydrogenase [NAD(+)] OS=Homo sapiens OX=9606 GN=HPGD PE=1 SV=1	1	1	1	29	3	266	5.86												
High	Q13561	Dynactin subunit 2 OS=Homo sapiens OX=9606 GN=DCTN2 PE=1 SV=4	1	1	1	44.2	2	401	5.21												
High	Q9NZM1	Myoferlin OS=Homo sapiens OX=9606 GN=MYOF PE=1 SV=1	1	1	1	234.6	1	2061	6.18												
High	Q9Y6R9	Centrosomal protein CCDC61 OS=Homo sapiens OX=9606 GN=CCDC61 PE=1 SV=3	1	1	1	57.3	2	512	10.27												
High	Q9ULH1	Arf-GAP with SH3 domain, ANK repeat and PH domain-containing protein 1 OS=Homo sapiens OX=9606 GN=ASAP1 PE=1 SV=4	1	1	1	125.4	2	1129	7.31												
High	Q9P0J1	[Pyruvate dehydrogenase [acetyl-transferring]]-phosphatase 1, mitochondrial OS=Homo sapiens OX=9606 GN=PDP1 PE=1 SV=3	1	1	1	61	3	537	6.67			1						1			
High	Q9UKG1	DCC-interacting protein 13-alpha OS=Homo sapiens OX=9606 GN=APPL1 PE=1 SV=1	1	1	1	79.6	2	709	5.41		1						1				
High	Q9NWV4	CXXC motif containing zinc binding protein OS=Homo sapiens OX=9606 GN=CZIB PE=1 SV=1	1	1	1	18	11	160	5.01			1						1			
High	Q01196	Runt-related transcription factor 1 OS=Homo sapiens OX=9606 GN=RUNX1 PE=1 SV=3	1	1	1	48.7	2	453	9.35			1						1			
High	P19387	DNA-directed RNA polymerase II subunit RPB3 OS=Homo sapiens OX=9606 GN=POLR2C PE=1 SV=2	1	1	1	31.4	4	275	4.92												
High	Q5VWZ2	Lysophospholipase-like protein 1 OS=Homo sapiens OX=9606 GN=LYPLAL1 PE=1 SV=3	1	1	1	26.3	4	237	7.84												
High	Q6WKZ4	Rab11 family-interacting protein 1 OS=Homo sapiens OX=9606 GN=RAB11FIP1 PE=1 SV=3	1	1	1	137.1	2	1283	5.43												
High	Q3LXA3	Triokinase/FMN cyclase OS=Homo sapiens OX=9606 GN=TKFC PE=1 SV=2	1	1	1	58.9	2	575	7.49												
High	Q5TH69	Brefeldin A-inhibited guanine nucleotide-exchange protein 3 OS=Homo sapiens OX=9606 GN=ARFGEF3 PE=1 SV=3	1	1	1	240.5	0	2177	5.82			1						1			
High	Q03701	CCAAT/enhancer-binding protein zeta OS=Homo sapiens OX=9606 GN=CEBPZ PE=1 SV=3	1	1	1	120.9	1	1054	5.94												
High	Q96L73	Histone-lysine N-methyltransferase, H3 lysine-36 specific OS=Homo sapiens OX=9606 GN=NSD1 PE=1 SV=1	1	1	1	296.5	1	2696	8.03	1						1					
High	Q9H3N1	Thioredoxin-related transmembrane protein 1 OS=Homo sapiens OX=9606 GN=TMX1 PE=1 SV=1	1	1	1	31.8	4	280	4.98												
High	Q460N5	Protein mono-ADP-ribosyltransferase PARP14 OS=Homo sapiens OX=9606 GN=PARP14 PE=1 SV=3	1	1	1	202.7	1	1801	7.18	1						1					
High	P52565	Rho GDP-dissociation inhibitor 1 OS=Homo sapiens OX=9606 GN=ARHGDIA PE=1 SV=3	1	1	1	23.2	8	204	5.11			1						1			
High	Q9H4L7	SWI/SNF-related matrix-associated actin-dependent regulator of chromatin subfamily A containing DEAD/H box 1 OS=Homo sapie	1	1	1	117.3	1	1026	5.55			1						1			
High	Q9H7X7	Intraflagellar transport protein 22 homolog OS=Homo sapiens OX=9606 GN=IFT22 PE=1 SV=1	1	1	1	20.8	12	185	5.15												
High	P39019	40S ribosomal protein S19 OS=Homo sapiens OX=9606 GN=RPS19 PE=1 SV=2	1	1	1	16.1	9	145	10.32	1						1					
High	Q9H329	Band 4.1-like protein 4B OS=Homo sapiens OX=9606 GN=EPB41L4B PE=2 SV=2	1	1	1	99.7	1	900	9.04												
High	Q86Y39	NADH dehydrogenase [ubiquinone] 1 alpha subcomplex subunit 11 OS=Homo sapiens OX=9606 GN=NDUFA11 PE=1 SV=3	1	1	1	14.8	16	141	8.72			1						1			
High	Q9NR31	GTP-binding protein SAR1a OS=Homo sapiens OX=9606 GN=SAR1A PE=1 SV=1	1	1	1	22.4	6	198	6.68												
High	Q8NCE0	tRNA-splicing endonuclease subunit Sen2 OS=Homo sapiens OX=9606 GN=TSEN2 PE=1 SV=2	1	1	1	53.2	5	465	7.85												
High	Q96HU1	Small G protein signaling modulator 3 OS=Homo sapiens OX=9606 GN=SGSM3 PE=1 SV=1	1	1	1	85.3	1	749	6												
High	P35030	Trypsin-3 OS=Homo sapiens OX=9606 GN=PRSS3 PE=1 SV=2	1	1	1	32.5	4	304	7.49					1						1	
High	Q9H269	Vacuolar protein sorting-associated protein 16 homolog OS=Homo sapiens OX=9606 GN=VPS16 PE=1 SV=2	1	1	1	94.6	1	839	6.77												
High	Q99973	Telomerase protein component 1 OS=Homo sapiens OX=9606 GN=TEP1 PE=1 SV=2	1	1	1	290.3	1	2627	7.97												
High	Q15363	Transmembrane emp24 domain-containing protein 2 OS=Homo sapiens OX=9606 GN=TMED2 PE=1 SV=1	1	1	1	22.7	8	201	5.17			1						1			
High	P48443	Retinoic acid receptor RXR-gamma OS=Homo sapiens OX=9606 GN=RXRG PE=1 SV=1	1	1	1	50.8	2	463	7.62			1						1			
High	P18615	Negative elongation factor E OS=Homo sapiens OX=9606 GN=NELFE PE=1 SV=3	1	1	1	43.2	6	380	9.33			1						1			
High	Q9BSF4	Mitochondrial import inner membrane translocase subunit Tim29 OS=Homo sapiens OX=9606 GN=TIMM29 PE=1 SV=2	1	1	1	29.2	5	260	8.09			1						1			
High	Q14790	Caspase-8 OS=Homo sapiens OX=9606 GN=CASP8 PE=1 SV=1	1	1	1	55.4	2	479	5.1	1						1					
High	O75052	Carboxyl-terminal PDZ ligand of neuronal nitric oxide synthase protein OS=Homo sapiens OX=9606 GN=NOS1AP PE=1 SV=3	1	1	1	56.1	2	506	6.3												
High	Q13111	Chromatin assembly factor 1 subunit A OS=Homo sapiens OX=9606 GN=CHAF1A PE=1 SV=3	1	1	1	106.8	2	956	5.94												
High	E9PRG8	Uncharacterized protein C11orf98 OS=Homo sapiens OX=9606 GN=C11orf98 PE=4 SV=2	1	1	1	14.2	11	123	11.53												
High	P61086	Ubiquitin-conjugating enzyme E2 K OS=Homo sapiens OX=9606 GN=UBE2K PE=1 SV=3	1	1	1	22.4	5	200	5.44					1						1	
High	Q15582	Transforming growth factor-beta-induced protein ig-h3 OS=Homo sapiens OX=9606 GN=TGFBI PE=1 SV=1	1	1	1	74.6	3	683	7.71					1						1	
High	A0AVI4	E3 ubiquitin-protein ligase TM129 OS=Homo sapiens OX=9606 GN=TMEM129 PE=1 SV=1	1	1	1	40.4	5	362	7.66			1						1			
High	Q9H270	Vacuolar protein sorting-associated protein 11 homolog OS=Homo sapiens OX=9606 GN=VPS11 PE=1 SV=1	1	1	1	107.8	3	941	7.05	1						1					
High	P34955	SWISS-PROT:P34955 (Bos taurus) Alpha-1-antiproteinase precursor	1	1	1	46.1	2	416	6.52					1						1	
High	Q86W56	Poly(ADP-ribose) glycohydrolase OS=Homo sapiens OX=9606 GN=PARG PE=1 SV=1	1	1	1	111	1	976	6.43												
High	P57772	Selenocysteine-specific elongation factor OS=Homo sapiens OX=9606 GN=EEFSEC PE=1 SV=4	1	1	1	65.3	2	596	8.35												
High	Q9NZD8	Maspardin OS=Homo sapiens OX=9606 GN=SPG21 PE=1 SV=1	1	1	1	34.9	6	308	6.28												
High	Q96JC9	ELL-associated factor 1 OS=Homo sapiens OX=9606 GN=EAF1 PE=1 SV=1	1	1	1	29	7	268	5.4												
High	Q5TA45	Integrator complex subunit 11 OS=Homo sapiens OX=9606 GN=INTS11 PE=1 SV=2	1	1	1	67.6	2	600	8.06			1						1			
High	Q5TCQ9	Membrane-associated guanylate kinase, WW and PDZ domain-containing protein 3 OS=Homo sapiens OX=9606 GN=MAGI3 PE=1 S	1	1	1	162.8	1	1481	8.09												
High	Q9NVR5	Protein kintoun OS=Homo sapiens OX=9606 GN=DNAAF2 PE=1 SV=2	1	1	1	91.1	2	837	5.2												
High	P17813	Endoglin OS=Homo sapiens OX=9606 GN=ENG PE=1 SV=2	1	1	1	70.5	3	658	6.61												
High	O75330	Hyaluronan mediated motility receptor OS=Homo sapiens OX=9606 GN=HMMR PE=1 SV=2	1	1	1	84	2	724	5.83												
High	P07199	Major centromere autoantigen B OS=Homo sapiens OX=9606 GN=CENPB PE=1 SV=2	1	1	1	65.1	2	599	4.55			1						1			
High	Q9BZI7	Regulator of nonsense transcripts 3B OS=Homo sapiens OX=9606 GN=UPF3B PE=1 SV=1	1	1	1	57.7	2	483	9.48			1						1			
High	A7KAX9	Rho GTPase-activating protein 32 OS=Homo sapiens OX=9606 GN=ARHGAP32 PE=1 SV=1	1	1	1	230.4	0	2087	6.74												
High	Q9BVI4	Nucleolar complex protein 4 homolog OS=Homo sapiens OX=9606 GN=NOC4L PE=1 SV=1	1	1	1	58.4	2	516	7.49												
High	Q86SK9	Stearoyl-CoA desaturase 5 OS=Homo sapiens OX=9606 GN=SCD5 PE=1 SV=2	1	1	1	37.6	5	330	9.61	1						1					
High	P08047	Transcription factor Sp1 OS=Homo sapiens OX=9606 GN=SP1 PE=1 SV=3	1	1	1	80.6	4	785	7.34												
High	O15541	E3 ubiquitin-protein ligase RNF113A OS=Homo sapiens OX=9606 GN=RNF113A PE=1 SV=1	1	1	1	38.8	3	343	5.69												
High	Q8N511	Transmembrane protein 199 OS=Homo sapiens OX=9606 GN=TMEM199 PE=1 SV=1	1	1	1	23.1	4	208	9.01	1						1					
High	O95834	Echinoderm microtubule-associated protein-like 2 OS=Homo sapiens OX=9606 GN=EML2 PE=1 SV=1	1	1	1	70.6	2	649	6.32												
High	P63316	Troponin C, slow skeletal and cardiac muscles OS=Homo sapiens OX=9606 GN=TNNC1 PE=1 SV=1	1	1	1	18.4	11	161	4.18					1						1	
High	Q9ULR3	Protein phosphatase 1H OS=Homo sapiens OX=9606 GN=PPM1H PE=1 SV=2	1	1	1	56.4	3	514	6.6												
High	P40855	Peroxisomal biogenesis factor 19 OS=Homo sapiens OX=9606 GN=PEX19 PE=1 SV=1	1	1	1	32.8	5	299	4.34												
High	Q9Y4B6	DDB1- and CUL4-associated factor 1 OS=Homo sapiens OX=9606 GN=DCAF1 PE=1 SV=3	1	1	1	168.9	1	1507	5.06												
High	Q9BZA7	Protocadherin-11 X-linked OS=Homo sapiens OX=9606 GN=PCDH11X PE=2 SV=1	1	1	1	147.5	1	1347	5.21												
High	P15104	Glutamine synthetase OS=Homo sapiens OX=9606 GN=GLUL PE=1 SV=4	1	1	1	42	5	373	6.89			1						1			
High	Q9H7E2	Tudor domain-containing protein 3 OS=Homo sapiens OX=9606 GN=TDRD3 PE=1 SV=1	1	1	1	73.1	2	651	9.23												
High	P49821	NADH dehydrogenase [ubiquinone] flavoprotein 1, mitochondrial OS=Homo sapiens OX=9606 GN=NDUFV1 PE=1 SV=4	1	1	1	50.8	2	464	8.21												
High	P29372	DNA-3-methyladenine glycosylase OS=Homo sapiens OX=9606 GN=MPG PE=1 SV=3	1	1	1	32.8	4	298	9.57	1						1					
High	Q12962	Transcription initiation factor TFIID subunit 10 OS=Homo sapiens OX=9606 GN=TAF10 PE=1 SV=1	1	1	1	21.7	4	218	6.57												
High	Q14197	Peptidyl-tRNA hydrolase ICT1, mitochondrial OS=Homo sapiens OX=9606 GN=MRPL58 PE=1 SV=1	1	1	1	23.6	5	206	10.07												
High	O43572	A-kinase anchor protein 10, mitochondrial OS=Homo sapiens OX=9606 GN=AKAP10 PE=1 SV=2	1	1	1	73.8	3	662	6.39			1						1			
High	Q6AI12	Ankyrin repeat domain-containing protein 40 OS=Homo sapiens OX=9606 GN=ANKRD40 PE=1 SV=2	1	1	1	41.1	5	368	4.97												
High	Q9UGK8	Secretion-regulating guanine nucleotide exchange factor OS=Homo sapiens OX=9606 GN=SERGEF PE=1 SV=2	1	1	1	49	2	458	6.46												
High	Q9H8H0	Nucleolar protein 11 OS=Homo sapiens OX=9606 GN=NOL11 PE=1 SV=1	1	1	1	81.1	1	719	6.07												
High	P42336	Phosphatidylinositol 4,5-bisphosphate 3-kinase catalytic subunit alpha isoform OS=Homo sapiens OX=9606 GN=PIK3CA PE=1 SV=2	1	1	1	124.2	2	1068	7.23												
High	Q8TAD8	Smad nuclear-interacting protein 1 OS=Homo sapiens OX=9606 GN=SNIP1 PE=1 SV=1	1	1	1	45.8	4	396	9.99												
High	Q15061	WD repeat-containing protein 43 OS=Homo sapiens OX=9606 GN=WDR43 PE=1 SV=3	1	1	1	74.8	4	677	5.57												
High	Q684P5	Rap1 GTPase-activating protein 2 OS=Homo sapiens OX=9606 GN=RAP1GAP2 PE=1 SV=2	1	1	1	80	1	730	6.67			1						1			
High	O95359	Transforming acidic coiled-coil-containing protein 2 OS=Homo sapiens OX=9606 GN=TACC2 PE=1 SV=3	1	1	1	309.2	0	2948	4.79												
High	Q5QJE6	Deoxynucleotidyltransferase terminal-interacting protein 2 OS=Homo sapiens OX=9606 GN=DNTTIP2 PE=1 SV=2	1	1	1	84.4	2	756	6.16			1						1			
High	O00189	AP-4 complex subunit mu-1 OS=Homo sapiens OX=9606 GN=AP4M1 PE=1 SV=2	1	1	1	49.9	3	453	7.2			1						1			
High	Q8TBE9	N-acylneuraminate-9-phosphatase OS=Homo sapiens OX=9606 GN=NANP PE=1 SV=1	1	1	1	27.8	5	248	6.4												
High	Q9H0N5	Pterin-4-alpha-carbinolamine dehydratase 2 OS=Homo sapiens OX=9606 GN=PCBD2 PE=1 SV=4	1	1	1	14.4	12	130	9.11	1						1					
High	Q9NWU1	3-oxoacyl-[acyl-carrier-protein] synthase, mitochondrial OS=Homo sapiens OX=9606 GN=OXSM PE=1 SV=1	1	1	1	48.8	3	459	7.66												
High	O43678	NADH dehydrogenase [ubiquinone] 1 alpha subcomplex subunit 2 OS=Homo sapiens OX=9606 GN=NDUFA2 PE=1 SV=3	1	1	1	10.9	21	99	9.57												
High	O60783	28S ribosomal protein S14, mitochondrial OS=Homo sapiens OX=9606 GN=MRPS14 PE=1 SV=1	1	1	1	15.1	12	128	11.41												
High	Q9BW30	Tubulin polymerization-promoting protein family member 3 OS=Homo sapiens OX=9606 GN=TPPP3 PE=1 SV=1	1	1	1	19	5	176	9.13					1						1	
High	Q8TD19	Serine/threonine-protein kinase Nek9 OS=Homo sapiens OX=9606 GN=NEK9 PE=1 SV=2	1	1	1	107.1	1	979	5.74												
High	Q5VV41	Rho guanine nucleotide exchange factor 16 OS=Homo sapiens OX=9606 GN=ARHGEF16 PE=1 SV=1	1	1	1	80.1	1	709	7.36			1						1			
High	P10619	Lysosomal protective protein OS=Homo sapiens OX=9606 GN=CTSA PE=1 SV=2	1	1	1	54.4	3	480	6.61					1						1	
High	Q9BT25	HAUS augmin-like complex subunit 8 OS=Homo sapiens OX=9606 GN=HAUS8 PE=1 SV=3	1	1	1	44.8	2	410	7.06			1						1			
High	Q969K7	Transmembrane protein 54 OS=Homo sapiens OX=9606 GN=TMEM54 PE=1 SV=1	1	1	1	23.8	4	222	7.83												
High	Q9ULQ0	Striatin-interacting protein 2 OS=Homo sapiens OX=9606 GN=STRIP2 PE=1 SV=2	1	1	1	95.3	1	834	5.91												
High	Q8IY22	C-Maf-inducing protein OS=Homo sapiens OX=9606 GN=CMIP PE=1 SV=3	1	1	1	86.3	3	773	6.7												
High	P35869	Aryl hydrocarbon receptor OS=Homo sapiens OX=9606 GN=AHR PE=1 SV=2	1	1	1	96.1	1	848	6.38												
High	Q8IXH7	Negative elongation factor C/D OS=Homo sapiens OX=9606 GN=NELFCD PE=1 SV=2	1	1	1	66.2	2	590	5.1												
High	Q8IYL3	UPF0688 protein C1orf174 OS=Homo sapiens OX=9606 GN=C1orf174 PE=1 SV=2	1	1	1	26	6	243	6.9												
High	O95396	Adenylyltransferase and sulfurtransferase MOCS3 OS=Homo sapiens OX=9606 GN=MOCS3 PE=1 SV=1	1	1	1	49.6	4	460	6.21		1						1				
High	Q9UPN4	Centrosomal protein of 131 kDa OS=Homo sapiens OX=9606 GN=CEP131 PE=1 SV=3	1	1	1	122.1	1	1083	8.69												
High	P09913	Interferon-induced protein with tetratricopeptide repeats 2 OS=Homo sapiens OX=9606 GN=IFIT2 PE=1 SV=1	1	1	1	54.6	2	472	6.76					1						1	
High	Q9Y2H0	Disks large-associated protein 4 OS=Homo sapiens OX=9606 GN=DLGAP4 PE=1 SV=3	1	1	1	107.9	1	992	7.08												
High	Q8NFU3	Thiosulfate:glutathione sulfurtransferase OS=Homo sapiens OX=9606 GN=TSTD1 PE=1 SV=3	1	1	1	12.5	12	115	6.07												
High	Q96LB3	Intraflagellar transport protein 74 homolog OS=Homo sapiens OX=9606 GN=IFT74 PE=1 SV=1	1	1	1	69.2	3	600	6		1						1				
High	Q9NW08	DNA-directed RNA polymerase III subunit RPC2 OS=Homo sapiens OX=9606 GN=POLR3B PE=1 SV=2	1	1	1	127.7	1	1133	8.5					1						1	
High	O95478	Ribosome biogenesis protein NSA2 homolog OS=Homo sapiens OX=9606 GN=NSA2 PE=1 SV=1	1	1	1	30	4	260	10.27												
High	Q6PJT7	Zinc finger CCCH domain-containing protein 14 OS=Homo sapiens OX=9606 GN=ZC3H14 PE=1 SV=1	1	1	1	82.8	1	736	7.31												

High	O00165	HCLS1-associated protein X-1 OS=Homo sapiens OX=9606 GN=HAX1 PE=1 SV=2	1	1	1	31.6	4	279	4.92			1						1			
High	Q9HCN4	GPN-loop GTPase 1 OS=Homo sapiens OX=9606 GN=GPN1 PE=1 SV=1	1	1	1	41.7	4	374	4.92			1						1			
High	Q7Z2K6	Endoplasmic reticulum metallopeptidase 1 OS=Homo sapiens OX=9606 GN=ERMP1 PE=1 SV=2	1	1	1	100.2	1	904	7.52			1						1			
High	Q9H3R2	Mucin-13 OS=Homo sapiens OX=9606 GN=MUC13 PE=1 SV=3	1	1	1	54.6	4	512	5.07												
High	Q12874	Splicing factor 3A subunit 3 OS=Homo sapiens OX=9606 GN=SF3A3 PE=1 SV=1	1	1	1	58.8	2	501	5.38												
High	O14880	Microsomal glutathione S-transferase 3 OS=Homo sapiens OX=9606 GN=MGST3 PE=1 SV=1	1	1	1	16.5	14	152	9.38												
High	Q92835	Phosphatidylinositol 3,4,5-trisphosphate 5-phosphatase 1 OS=Homo sapiens OX=9606 GN=INPP5D PE=1 SV=2	1	1	1	133.2	1	1189	7.59												
High	Q96MC6	Hippocampus abundant transcript 1 protein OS=Homo sapiens OX=9606 GN=MFSD14A PE=1 SV=2	1	1	1	53	4	490	8.4	1						1					
High	O00400	Acetyl-coenzyme A transporter 1 OS=Homo sapiens OX=9606 GN=SLC33A1 PE=1 SV=1	1	1	1	60.9	2	549	7.33												
High	Q96AZ6	Interferon-stimulated gene 20 kDa protein OS=Homo sapiens OX=9606 GN=ISG20 PE=1 SV=2	1	1	1	20.4	9	181	8.92												
High	Q9NUQ2	1-acyl-sn-glycerol-3-phosphate acyltransferase epsilon OS=Homo sapiens OX=9606 GN=AGPAT5 PE=1 SV=3	1	1	1	42	6	364	9.1												
High	O60238	BCL2/adenovirus E1B 19 kDa protein-interacting protein 3-like OS=Homo sapiens OX=9606 GN=BNIP3L PE=1 SV=1	1	1	1	23.9	7	219	5.85			1						1			
High	O75431	Metaxin-2 OS=Homo sapiens OX=9606 GN=MTX2 PE=1 SV=1	1	1	1	29.7	4	263	6.29						1						1
High	Q8NFV4	Protein ABHD11 OS=Homo sapiens OX=9606 GN=ABHD11 PE=1 SV=1	1	1	1	34.7	5	315	9.48			1						1			
High	P31749	RAC-alpha serine/threonine-protein kinase OS=Homo sapiens OX=9606 GN=AKT1 PE=1 SV=2	1	1	1	55.7	3	480	6.07												
High	O14949	Cytochrome b-c1 complex subunit 8 OS=Homo sapiens OX=9606 GN=UQCRQ PE=1 SV=4	1	1	1	9.9	16	82	10.08		1						1				
High	Q6PJ69	Tripartite motif-containing protein 65 OS=Homo sapiens OX=9606 GN=TRIM65 PE=1 SV=3	1	1	1	57.3	3	517	6.7			1						1			
High	O75817	Ribonuclease P protein subunit p20 OS=Homo sapiens OX=9606 GN=POP7 PE=1 SV=2	1	1	1	15.6	11	140	8.94												
High	Q9NUD5	Zinc finger CCHC domain-containing protein 3 OS=Homo sapiens OX=9606 GN=ZCCHC3 PE=1 SV=2	1	1	1	43.5	4	403	8.53												
High	Q96EH3	Mitochondrial assembly of ribosomal large subunit protein 1 OS=Homo sapiens OX=9606 GN=MALSU1 PE=1 SV=1	1	1	1	26.2	9	234	5.49												
High	Q9BTL3	RNA guanine-N7 methyltransferase activating subunit OS=Homo sapiens OX=9606 GN=RAMAC PE=1 SV=1	1	1	1	14.4	11	118	8.94			1						1			
High	Q9BSR8	Protein YIPF4 OS=Homo sapiens OX=9606 GN=YIPF4 PE=1 SV=1	1	1	1	27.1	4	244	4.65			1						1			
High	Q14139	Ubiquitin conjugation factor E4 A OS=Homo sapiens OX=9606 GN=UBE4A PE=1 SV=2	1	1	1	122.5	1	1066	5.24												
High	Q8TBF4	Zinc finger CCHC-type and RNA-binding motif-containing protein 1 OS=Homo sapiens OX=9606 GN=ZCRB1 PE=1 SV=2	1	1	1	24.6	7	217	8.53												
High	Q96QT4	Transient receptor potential cation channel subfamily M member 7 OS=Homo sapiens OX=9606 GN=TRPM7 PE=1 SV=1	1	1	1	212.6	1	1865	7.88												
High	Q9NRY4	Rho GTPase-activating protein 35 OS=Homo sapiens OX=9606 GN=ARHGAP35 PE=1 SV=3	1	1	1	170.4	1	1499	6.64												
High	P08243	Asparagine synthetase [glutamine-hydrolyzing] OS=Homo sapiens OX=9606 GN=ASNS PE=1 SV=4	1	1	1	64.3	2	561	6.86			1						1			
High	Q9NV70	Exocyst complex component 1 OS=Homo sapiens OX=9606 GN=EXOC1 PE=1 SV=4	1	1	1	101.9	1	894	6.61												
High	Q96EK5	KIF-binding protein OS=Homo sapiens OX=9606 GN=KIFBP PE=1 SV=1	1	1	1	71.8	2	621	5.49	1						1					
High	P31937	3-hydroxyisobutyrate dehydrogenase, mitochondrial OS=Homo sapiens OX=9606 GN=HIBADH PE=1 SV=2	1	1	1	35.3	4	336	8.13			1						1			
High	Q99102	Mucin-4 OS=Homo sapiens OX=9606 GN=MUC4 PE=1 SV=5	1	1	1	542	1	5412	5.3												
High	Q99470	Stromal cell-derived factor 2 OS=Homo sapiens OX=9606 GN=SDF2 PE=1 SV=2	1	1	1	23	7	211	7.33		1						1				
High	Q9H0H0	Integrator complex subunit 2 OS=Homo sapiens OX=9606 GN=INTS2 PE=1 SV=2	1	1	1	134.2	1	1204	6.05			1						1			
High	Q9BXS4	Transmembrane protein 59 OS=Homo sapiens OX=9606 GN=TMEM59 PE=1 SV=1	1	1	1	36.2	4	323	5.1												
High	Q5T8D3	Acyl-CoA-binding domain-containing protein 5 OS=Homo sapiens OX=9606 GN=ACBD5 PE=1 SV=1	1	1	1	60.1	3	534	5.33												
High	Q9BSM1	Polycomb group RING finger protein 1 OS=Homo sapiens OX=9606 GN=PCGF1 PE=1 SV=2	1	1	1	30.3	4	259	8.91					1						1	
High	Q12974	Protein tyrosine phosphatase type IVA 2 OS=Homo sapiens OX=9606 GN=PTP4A2 PE=1 SV=1	1	1	1	19.1	8	167	8.37		1						1				
High	Q9NTJ5	Phosphatidylinositol-3-phosphatase SAC1 OS=Homo sapiens OX=9606 GN=SACM1L PE=1 SV=2	1	1	1	66.9	2	587	7.12			1						1			
High	Q6UWP8	Suprabasin OS=Homo sapiens OX=9606 GN=SBSN PE=1 SV=2	1	1	1	60.5	3	590	7.01					1						1	
High	Q6ZRP7	Sulfhydryl oxidase 2 OS=Homo sapiens OX=9606 GN=QSOX2 PE=1 SV=3	1	1	1	77.5	2	698	7.72												
High	Q15833	Syntaxin-binding protein 2 OS=Homo sapiens OX=9606 GN=STXBP2 PE=1 SV=2	1	1	1	66.4	3	593	6.55		1						1				
High	Q9NSP4	Centromere protein M OS=Homo sapiens OX=9606 GN=CENPM PE=1 SV=1	1	1	1	19.7	6	180	7.2						1						1
High	Q9Y3T9	Nucleolar complex protein 2 homolog OS=Homo sapiens OX=9606 GN=NOC2L PE=1 SV=4	1	1	1	84.9	1	749	5.62												
High	Q8TAP8	Protein phosphatase 1 regulatory subunit 35 OS=Homo sapiens OX=9606 GN=PPP1R35 PE=1 SV=1	1	1	1	27.9	9	253	8.68												
High	P17612	cAMP-dependent protein kinase catalytic subunit alpha OS=Homo sapiens OX=9606 GN=PRKACA PE=1 SV=2	1	1	1	40.6	5	351	8.79												
High	P06756	Integrin alpha-V OS=Homo sapiens OX=9606 GN=ITGAV PE=1 SV=2	1	1	1	116	2	1048	5.68												
High	Q9H1K1	Iron-sulfur cluster assembly enzyme ISCU, mitochondrial OS=Homo sapiens OX=9606 GN=ISCU PE=1 SV=2	1	1	1	18	10	167	9.48												
High	P13498	Cytochrome b-245 light chain OS=Homo sapiens OX=9606 GN=CYBA PE=1 SV=3	1	1	1	21	4	195	9.54												
High	Q9Y6A4	Cilia- and flagella-associated protein 20 OS=Homo sapiens OX=9606 GN=CFAP20 PE=1 SV=1	1	1	1	22.8	4	193	9.76												
High	Q6I9Y2	THO complex subunit 7 homolog OS=Homo sapiens OX=9606 GN=THOC7 PE=1 SV=3	1	1	1	23.7	7	204	5.67												
High	Q9HC07	Transmembrane protein 165 OS=Homo sapiens OX=9606 GN=TMEM165 PE=1 SV=1	1	1	1	34.9	8	324	7.02			1						1			
High	Q14689	Disco-interacting protein 2 homolog A OS=Homo sapiens OX=9606 GN=DIP2A PE=1 SV=2	1	1	1	170.3	1	1571	8.03												
High	O60921	Checkpoint protein HUS1 OS=Homo sapiens OX=9606 GN=HUS1 PE=1 SV=1	1	1	1	31.7	4	280	6.84			1						1			
High	Q9Y6H1	Coiled-coil-helix-coiled-coil-helix domain-containing protein 2 OS=Homo sapiens OX=9606 GN=CHCHD2 PE=1 SV=1	1	1	1	15.5	7	151	9.22		1						1				
High	Q8N6N3	UPF0690 protein C1orf52 OS=Homo sapiens OX=9606 GN=C1orf52 PE=1 SV=1	1	1	1	20.6	8	182	5.21												
High	Q16719	Kynureninase OS=Homo sapiens OX=9606 GN=KYNU PE=1 SV=1	1	1	1	52.3	6	465	7.03					1						1	
High	Q9BYD1	39S ribosomal protein L13, mitochondrial OS=Homo sapiens OX=9606 GN=MRPL13 PE=1 SV=1	1	1	1	20.7	4	178	9.16					1						1	
High	Q5K651	Sterile alpha motif domain-containing protein 9 OS=Homo sapiens OX=9606 GN=SAMD9 PE=1 SV=1	1	1	1	184.2	0	1589	7.83					1						1	
High	O75907	Diacylglycerol O-acyltransferase 1 OS=Homo sapiens OX=9606 GN=DGAT1 PE=1 SV=2	1	1	1	55.2	4	488	9.32												
High	P07357	Complement component C8 alpha chain OS=Homo sapiens OX=9606 GN=C8A PE=1 SV=2	1	1	1	65.1	5	584	6.47												
High	Q86UV5	Ubiquitin carboxyl-terminal hydrolase 48 OS=Homo sapiens OX=9606 GN=USP48 PE=1 SV=1	1	1	1	119	1	1035	6.05												
High	Q9BWH6	RNA polymerase II-associated protein 1 OS=Homo sapiens OX=9606 GN=RPAP1 PE=1 SV=3	1	1	1	152.7	1	1393	6.38			1						1			
High	Q8IY17	Patatin-like phospholipase domain-containing protein 6 OS=Homo sapiens OX=9606 GN=PNPLA6 PE=1 SV=3	1	1	1	150.9	1	1375	7.74	1						1					
High	Q8N5V2	Ephexin-1 OS=Homo sapiens OX=9606 GN=NGEF PE=1 SV=2	1	1	1	82.4	2	710	5.57		1						1				
High	Q96EY9	Probable inactive tRNA-specific adenosine deaminase-like protein 3 OS=Homo sapiens OX=9606 GN=ADAT3 PE=1 SV=1	1	1	1	38	7	351	7.88												
High	Q15654	Thyroid receptor-interacting protein 6 OS=Homo sapiens OX=9606 GN=TRIP6 PE=1 SV=3	1	1	1	50.3	4	476	7.37												
High	O95985	DNA topoisomerase 3-beta-1 OS=Homo sapiens OX=9606 GN=TOP3B PE=1 SV=1	1	1	1	96.6	2	862	8.12		1						1				
High	Q15075	Early endosome antigen 1 OS=Homo sapiens OX=9606 GN=EEA1 PE=1 SV=2	1	1	1	162.4	1	1411	5.68			1						1			
High	Q8IYB7	DIS3-like exonuclease 2 OS=Homo sapiens OX=9606 GN=DIS3L2 PE=1 SV=4	1	1	1	99.2	2	885	6.1												
High	O75251	NADH dehydrogenase [ubiquinone] iron-sulfur protein 7, mitochondrial OS=Homo sapiens OX=9606 GN=NDUFS7 PE=1 SV=3	1	1	1	23.5	4	213	9.99		1						1				
High	Q9BT40	Inositol polyphosphate 5-phosphatase K OS=Homo sapiens OX=9606 GN=INPP5K PE=1 SV=3	1	1	1	51.1	2	448	6.54		1						1				
High	Q9H7Z7	Prostaglandin E synthase 2 OS=Homo sapiens OX=9606 GN=PTGES2 PE=1 SV=1	1	1	1	41.9	3	377	9.16	1						1					
High	Q8WX93	Palladin OS=Homo sapiens OX=9606 GN=PALLD PE=1 SV=3	1	1	1	150.5	1	1383	7.09				1						1		
High	O15525	Transcription factor MafG OS=Homo sapiens OX=9606 GN=MAFG PE=1 SV=1	1	1	1	17.8	6	162	10.04						1						1
High	Q96A32	Myosin regulatory light chain 2, skeletal muscle isoform OS=Homo sapiens OX=9606 GN=MYLPF PE=1 SV=1	1	1	1	19	8	169	5.01				1						1		
High	Q15125	3-beta-hydroxysteroid-Delta(8),Delta(7)-isomerase OS=Homo sapiens OX=9606 GN=EBP PE=1 SV=3	1	1	1	26.3	4	230	7.9			1						1			
High	Q9BX40	Protein LSM14 homolog B OS=Homo sapiens OX=9606 GN=LSM14B PE=1 SV=1	1	1	1	42	3	385	9.69												
High	Q92544	Transmembrane 9 superfamily member 4 OS=Homo sapiens OX=9606 GN=TM9SF4 PE=1 SV=2	1	1	1	74.5	3	642	6.54		1						1				
High	Q6ZRI6	Uncharacterized protein C15orf39 OS=Homo sapiens OX=9606 GN=C15orf39 PE=1 SV=3	1	1	1	110.6	2	1047	7.64												
High	Q9UM13	Anaphase-promoting complex subunit 10 OS=Homo sapiens OX=9606 GN=ANAPC10 PE=1 SV=1	1	1	1	21.2	6	185	9.06	1						1					
High	Q9Y2B0	Protein canopy homolog 2 OS=Homo sapiens OX=9606 GN=CNPY2 PE=1 SV=1	1	1	1	20.6	8	182	4.92	1						1					
High	Q96PE2	Rho guanine nucleotide exchange factor 17 OS=Homo sapiens OX=9606 GN=ARHGEF17 PE=1 SV=1	1	1	1	221.5	0	2063	6.29												
High	Q8NC56	LEM domain-containing protein 2 OS=Homo sapiens OX=9606 GN=LEMD2 PE=1 SV=1	1	1	1	56.9	3	503	9		1						1				
High	Q5XXA6	Anoctamin-1 OS=Homo sapiens OX=9606 GN=ANO1 PE=1 SV=1	1	1	1	114	2	986	8.54												
High	P35568	Insulin receptor substrate 1 OS=Homo sapiens OX=9606 GN=IRS1 PE=1 SV=1	1	1	1	131.5	1	1242	8.54												
High	Q5JVF3	PCI domain-containing protein 2 OS=Homo sapiens OX=9606 GN=PCID2 PE=1 SV=2	1	1	1	46	3	399	8.53												
High	P50135	Histamine N-methyltransferase OS=Homo sapiens OX=9606 GN=HNMT PE=1 SV=1	1	1	1	33.3	8	292	5.34												
High	O43681	ATPase GET3 OS=Homo sapiens OX=9606 GN=GET3 PE=1 SV=2	1	1	1	38.8	3	348	4.91												
High	Q9BW85	Splicing factor YJU2 OS=Homo sapiens OX=9606 GN=YJU2 PE=1 SV=1	1	1	1	37.1	5	323	5.92												
High	Q96QA5	Gasdermin-A OS=Homo sapiens OX=9606 GN=GSDMA PE=1 SV=4	1	1	1	49.3	6	445	5.29			1						1			
High	Q8N392	Rho GTPase-activating protein 18 OS=Homo sapiens OX=9606 GN=ARHGAP18 PE=1 SV=3	1	1	1	74.9	2	663	6.44			1						1			
High	Q8TB03	Uncharacterized protein CXorf38 OS=Homo sapiens OX=9606 GN=CXorf38 PE=1 SV=1	1	1	1	36.6	6	319	6.3			1						1			
High	Q96FX7	tRNA (adenine(58)-N(1))-methyltransferase catalytic subunit TRMT61A OS=Homo sapiens OX=9606 GN=TRMT61A PE=1 SV=1	1	1	1	31.4	9	289	7.36												
High	Q6NZI2	Caveolae-associated protein 1 OS=Homo sapiens OX=9606 GN=CAVIN1 PE=1 SV=1	1	1	1	43.5	5	390	5.6				1						1		
High	O95376	E3 ubiquitin-protein ligase ARIH2 OS=Homo sapiens OX=9606 GN=ARIH2 PE=1 SV=1	1	1	1	57.8	3	493	5.63												
High	A5D8V6	Vacuolar protein sorting-associated protein 37C OS=Homo sapiens OX=9606 GN=VPS37C PE=1 SV=2	1	1	1	38.6	3	355	5.21												
High	Q96SZ6	Mitochondrial tRNA methylthiotransferase CDK5RAP1 OS=Homo sapiens OX=9606 GN=CDK5RAP1 PE=1 SV=2	1	1	1	67.6	3	601	8.21												
High	O94804	Serine/threonine-protein kinase 10 OS=Homo sapiens OX=9606 GN=STK10 PE=1 SV=1	1	1	1	112.1	2	968	6.95												
High	P19404	NADH dehydrogenase [ubiquinone] flavoprotein 2, mitochondrial OS=Homo sapiens OX=9606 GN=NDUFV2 PE=1 SV=2	1	1	1	27.4	4	249	8.06												
High	Q9Y4W2	Ribosomal biogenesis protein LAS1L OS=Homo sapiens OX=9606 GN=LAS1L PE=1 SV=2	1	1	1	83	2	734	4.73			1						1			
High	Q9NVH2	Integrator complex subunit 7 OS=Homo sapiens OX=9606 GN=INTS7 PE=1 SV=1	1	1	1	106.8	1	962	8.02			1						1			
High	Q8IUH5	Palmitoyltransferase ZDHHC17 OS=Homo sapiens OX=9606 GN=ZDHHC17 PE=1 SV=2	1	1	1	72.6	3	632	7.5			1						1			
High	Q13503	Mediator of RNA polymerase II transcription subunit 21 OS=Homo sapiens OX=9606 GN=MED21 PE=1 SV=1	1	1	1	15.6	13	144	4.45												
High	Q13586	Stromal interaction molecule 1 OS=Homo sapiens OX=9606 GN=STIM1 PE=1 SV=3	1	1	1	77.4	2	685	6.67												
High	Q9BTE7	DCN1-like protein 5 OS=Homo sapiens OX=9606 GN=DCUN1D5 PE=1 SV=1	1	1	1	27.5	5	237	5.58												
High	Q9UPT8	Zinc finger CCCH domain-containing protein 4 OS=Homo sapiens OX=9606 GN=ZC3H4 PE=1 SV=3	1	1	1	140.2	2	1303	6.27												
High	Q8N0Z3	Spindle and centriole-associated protein 1 OS=Homo sapiens OX=9606 GN=SPICE1 PE=1 SV=1	1	1	1	96.2	2	855	7.44												
High	Q15056	Eukaryotic translation initiation factor 4H OS=Homo sapiens OX=9606 GN=EIF4H PE=1 SV=5	1	1	1	27.4	5	248	7.23			1						1			
High	Q13277	Syntaxin-3 OS=Homo sapiens OX=9606 GN=STX3 PE=1 SV=3	1	1	1	33.1	3	289	5.44												
High	O96008	Mitochondrial import receptor subunit TOM40 homolog OS=Homo sapiens OX=9606 GN=TOMM40 PE=1 SV=1	1	1	1	37.9	3	361	7.25												
High	Q14680	Maternal embryonic leucine zipper kinase OS=Homo sapiens OX=9606 GN=MELK PE=1 SV=3	1	1	1	74.6	1	651	8.72					1						1	
High	Q13952	Nuclear transcription factor Y subunit gamma OS=Homo sapiens OX=9606 GN=NFYC PE=1 SV=3	1	1	1	50.3	2	458	6.1			1						1			
