# Supplementary material for: Inhibition of O-GlcNAc transferase activates type I interferon-dependent antitumor immunity by bridging cGAS-STING pathway
Source: bioRxiv. 2024 Jun 11:2023.12.14.571787. Originally published 2023 Dec 15. Preprint. [Version 3] doi: 10.1101/2023.12.14.571787 (PMC10760207; doi:10.1101/2023.12.14.571787)
Supplement: 1 [file NIHPP2023.12.14.571787V3-supplement-1.pdf]

bioRxiv preprint doi: https://doi.org/10.1101/2023.12.14.571787; this version posted June 11, 2024. The copyright holder for this preprint (which was not certified by peer review) is the author/funder. This article is a US Government work. It is not subject to copyright under 17 USC 105 and is also made available for use under a CC0 license.

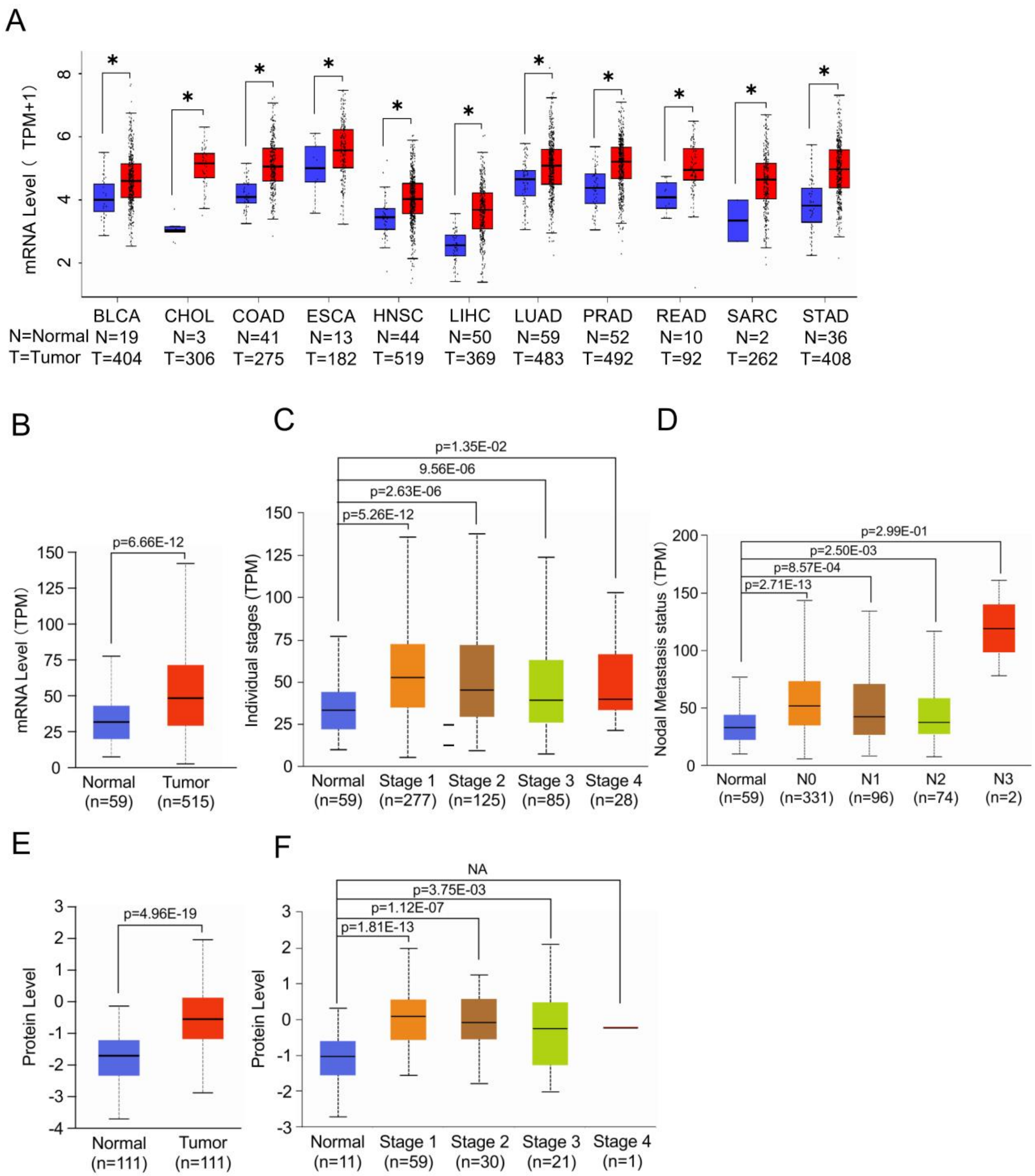

Supplemental Fig. 1 The expression pattern of OGT in TCGA and CTPAC databases.

A) Boxplot showing mRNA expression level of Ogt in multiple types of cancers. The plot was generated using the GEPIA2 online server. *p < 0.05. **B-D)** Boxplot showing mRNA expression level of *Ogt* in LUAD, Normal and tumor samples (**B**), Individual stages (**C**), Nodal metastasis

bioRxiv preprint doi: https://doi.org/10.1101/2023.12.14.571787; this version posted June 11, 2024. The copyright holder for this preprint (which was not certified by peer review) is the author/funder. This article is a US Government work. It is not subject to copyright under 17 USC 105 and is also made available for use under a CC0 license.

status (**D**). The plot was generated using the UALCAN online server. **E-F)** Boxplot showing protein expression level of OGT in LUAD, Normal and tumor samples (**E**), Individual stages (**F**). The plot was generated using the UALCAN online server (https://ualcan.path.uab.edu/analysis.html). Statistical significance was determined by Pearson test, unpaired Student's t-test, $*p < 0.05$, $**p < 0.01$, $***p < 0.001$, ns, no significant difference. Data represent the mean of ± SD.

bioRxiv preprint doi: https://doi.org/10.1101/2023.12.14.571787; this version posted June 11, 2024. The copyright holder for this preprint (which was not certified by peer review) is the author/funder. This article is a US Government work. It is not subject to copyright under 17 USC 105 and is also made available for use under a CC0 license.

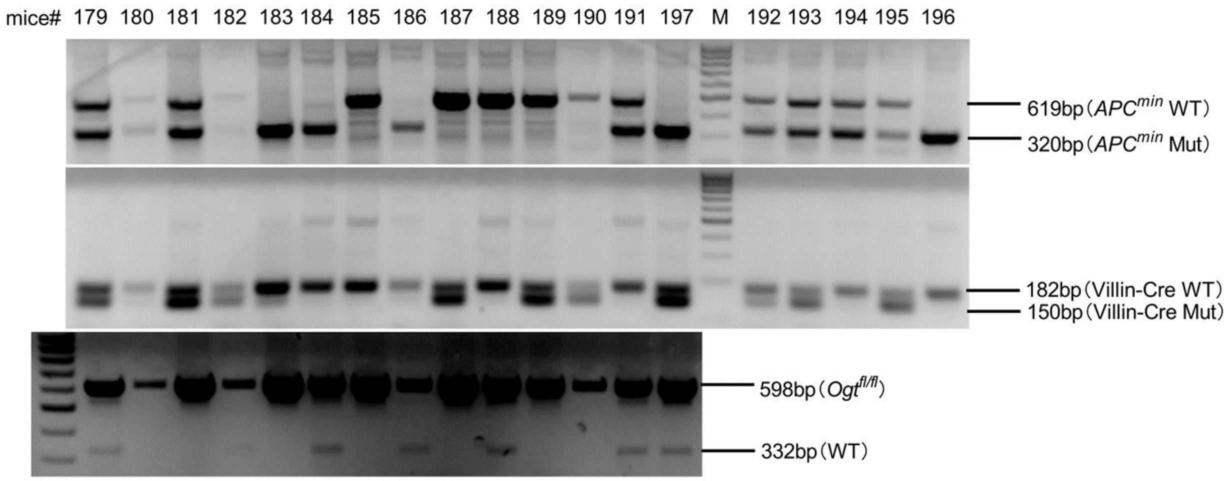

Supplemental Fig. 2. The genotype of *APC^{min}* ,*Villin-Cre* and *Ogt$^{fl/fl}$* mice.

Genomic DNA was extracted from the tails of *APC^{min}*, *Villin-Cre, and Ogt$^{fl/fl}$* mice and used for PCR with various primers. The resulting products were separated by agarose gel electrophoresis to determine the genotype.

bioRxiv preprint doi: https://doi.org/10.1101/2023.12.14.571787; this version posted June 11, 2024. The copyright holder for this preprint (which was not certified by peer review) is the author/funder. This article is a US Government work. It is not subject to copyright under 17 USC 105 and is also made available for use under a CC0 license.

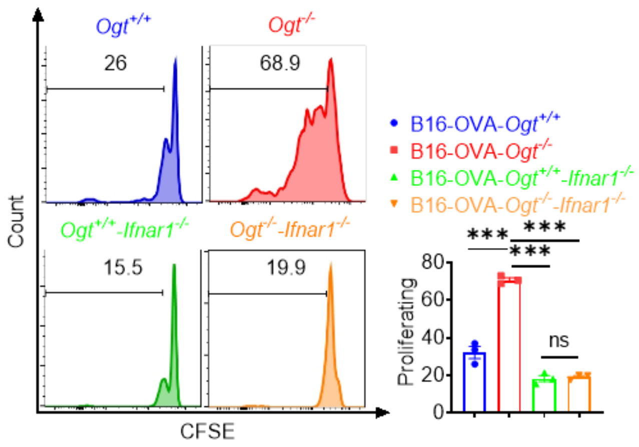

Supplemental Fig. 3 *In vitro* Cross-Priming of T Cells by *Ifnar*$^{-/-}$ BMDCs.

Ifnar1$^{-/-}$ BMDCs was pre-treated with B16-OVA-*Ogt*$^{+/+}$ or B16-OVA-*Ogt*$^{-/-}$ supernatant, and co-cultured with OT-1 T cell, then T cell proliferation was evaluated by flow cytometry. Representative fluorescence-activated cell sorting histograms and statistical data are shown. Data are representative of three independent experiments. Statistical significance was determined by one-way ANOVA, *$p < 0.05$, **$p < 0.01$, ***$p < 0.001$, ns, no significant difference. Data represent the mean of ± SD.

bioRxiv preprint doi: https://doi.org/10.1101/2023.12.14.571787; this version posted June 11, 2024. The copyright holder for this preprint (which was not certified by peer review) is the author/funder. This article is a US Government work. It is not subject to copyright under 17 USC 105 and is also made available for use under a CC0 license.

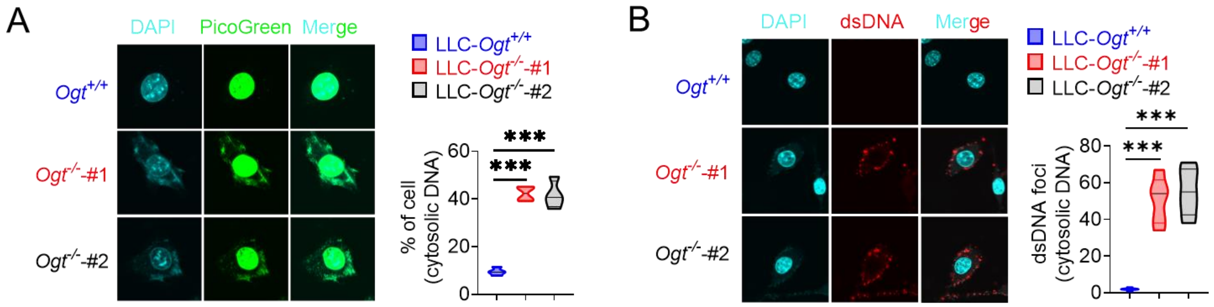

Supplemental Fig. 4 OGT deficiency causes DNA damage and accumulates cytosolic DNA. **A)** The extranuclear dsDNA in different *Ogt*$^{-/-}$ LLC clones were determined by PicoGreen staining assay and was quantified by image J. **B)** The extranuclear dsDNA in different *Ogt*$^{-/-}$ LLC clones were determined by anti-dsDNA fluorescence staining assay and was quantified by image J. Data are representative of three independent experiments. Statistical significance was determined by unpaired Student's t-test, two-way ANOVA, $*p < 0.05$, $**p < 0.01$, $***p < 0.001$, ns, no significant difference. Data represent the mean of ± SD.

bioRxiv preprint doi: https://doi.org/10.1101/2023.12.14.571787; this version posted June 11, 2024. The copyright holder for this preprint (which was not certified by peer review) is the author/funder. This article is a US Government work. It is not subject to copyright under 17 USC 105 and is also made available for use under a CC0 license.

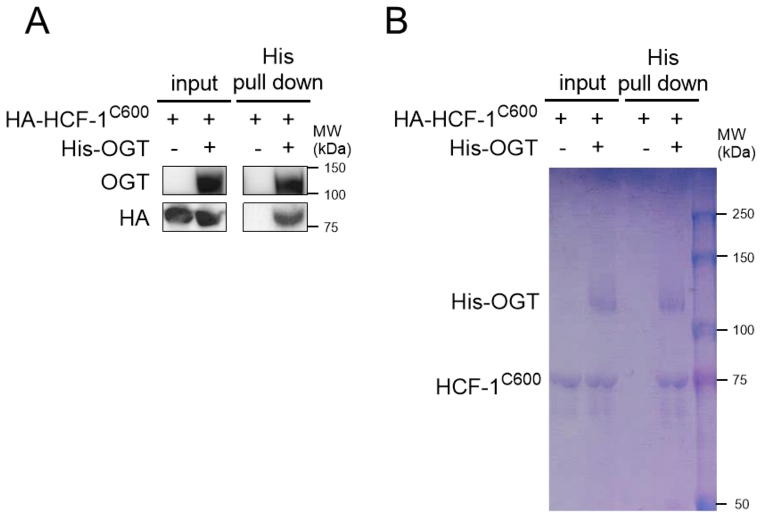

Supplemental Fig. 5 *In vitro* pull-down assay analysis of the interaction of HCF-1^{C600} and OGT.

A) His pull-down assays were used to analyze the interaction between HCF-1C600 and OGT. **B)** His-tagged OGT and HCF1 were expressed and purified, followed by SDS-PAGE separation and staining with Coomassie blue.

bioRxiv preprint doi: https://doi.org/10.1101/2023.12.14.571787; this version posted June 11, 2024. The copyright holder for this preprint (which was not certified by peer review) is the author/funder. This article is a US Government work. It is not subject to copyright under 17 USC 105 and is also made available for use under a CC0 license.

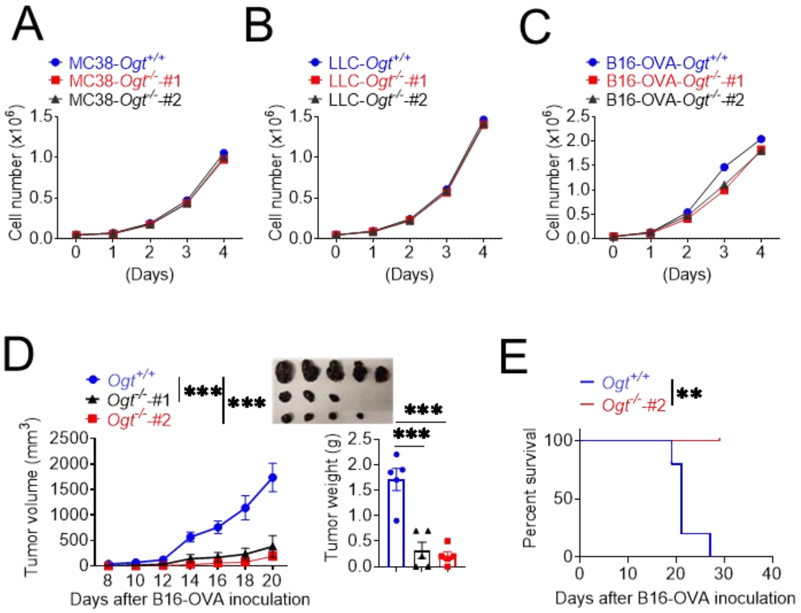

Supplemental Fig. 6 The cell proliferation of different tumor model *in vitro* and B16-OVA tumor growth analysis *in vivo*.

A-C), The cell proliferation of different *Ogt*$^{-/-}$ tumor model in vitro. **A)** MC38, **B)** LLC, **C)** B16-OVA *Ogt*$^{-/-}$ cells proliferation in *vitro*. **D-E)** Tumor volume, weight of *Ogt*$^{+/+}$ or *Ogt*$^{-/-}$ B16-OVA tumors in C57BL/6J mice, and mice survival, n=5 respectively. Data are representative of two or three independent experiments. Statistical significance was determined by unpaired Student's t-test, two-way ANOVA, *$p < 0.05$, **$p < 0.01$, ***$p < 0.001$, ns, no significant difference. Data represent the mean of ± SD.

bioRxiv preprint doi: https://doi.org/10.1101/2023.12.14.571787; this version posted June 11, 2024. The copyright holder for this preprint (which was not certified by peer review) is the author/funder. This article is a US Government work. It is not subject to copyright under 17 USC 105 and is also made available for use under a CC0 license.

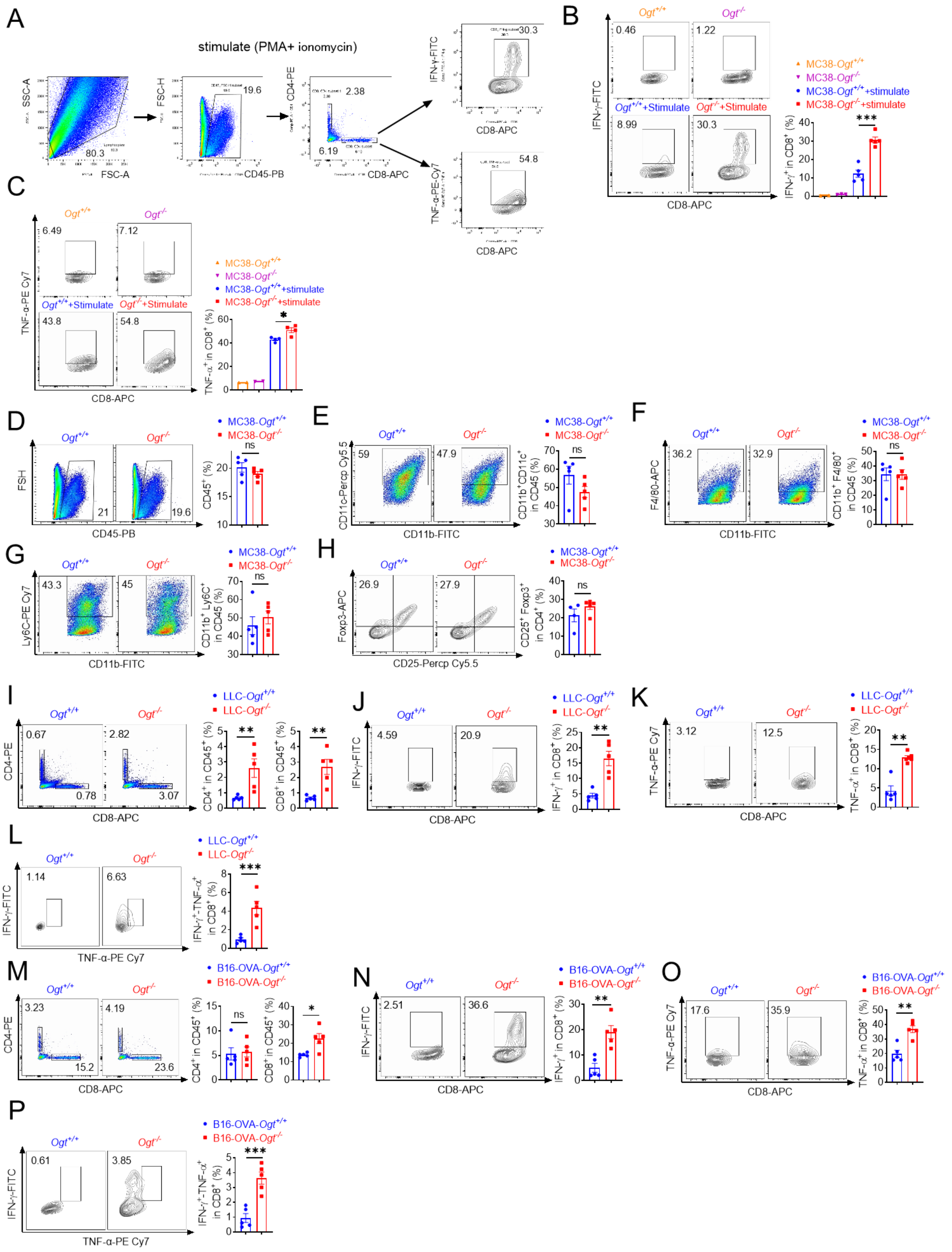

bioRxiv preprint doi: https://doi.org/10.1101/2023.12.14.571787; this version posted June 11, 2024. The copyright holder for this preprint (which was not certified by peer review) is the author/funder. This article is a US Government work. It is not subject to copyright under 17 USC 105 and is also made available for use under a CC0 license.

Supplemental Fig. 7 *Ogt* deficiency inhibits tumor progression through enhancing infiltration of CD8$^+$ T cells.

A) A total schematic of flow cytometry analysis. **B-C)** Flow cytometry analysis showing percentage of IFN-γ and TNF-α-expressing intratumoral CD8$^+$ T cells in MC38 tumors with or without PMA and ionomycin stimulation. **D-H)** Flow cytometry analysis showing percentage of CD45$^+$ (**D**), CD11b$^+$ CD11c$^+$ (**E**), CD11b$^+$ F4/80$^+$ (**F**), CD11b$^+$ Ly6C$^+$ (**G**) and Treg cells (**H**) in MC38 tumor model, n=5 respectively. **I-P)** Flow cytometry analysis showing percentage of CD4$^+$ and CD8$^+$ T cells in LLC (**I**), B16-OVA cells (**M**). IFN-γ$^+$, TNF-α$^+$ and IFN-γ$^+$ TNF-α$^+$ double positive expressing intratumoral CD8$^+$ T cells in LLC (**J-L**), or B16-OVA (**N-P**) tumors isolated at day 18 post-tumor inoculation, n=5 respectively. Data are representative of two or three independent experiments. Statistical significance was determined by unpaired Student's t-test, one-way ANOVA, *$p < 0.05$, **$p < 0.01$, ***$p < 0.001$, ns, no significant difference. Data represent the mean of ± SD.

bioRxiv preprint doi: https://doi.org/10.1101/2023.12.14.571787; this version posted June 11, 2024. The copyright holder for this preprint (which was not certified by peer review) is the author/funder. This article is a US Government work. It is not subject to copyright under 17 USC 105 and is also made available for use under a CC0 license.

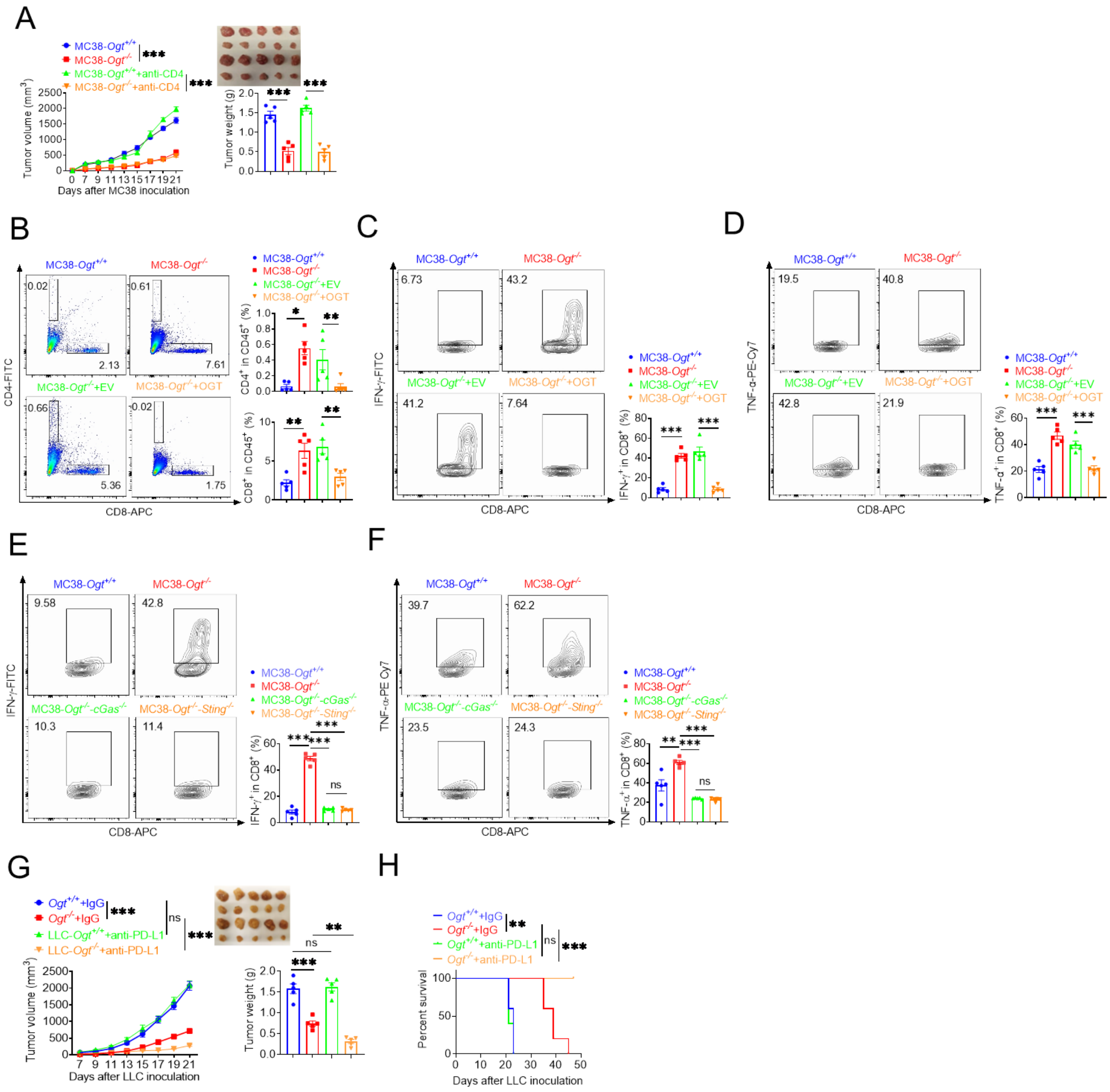

Supplemental Fig. 8. *Ogt* deficiency inhibits tumor progression through enhancing infiltration of CD8^{+} T cells.

A) Tumor volume and weight of *Ogt*$^{+/+}$ or *Ogt*$^{-/-}$ MC38 tumors injected with control IgG or anti-CD4 antibody at day 0, 7 and 14 post tumor inoculation in C57BL/6J mice, n=5 respectively. **B-D)** The percentage of CD4^{+} and CD8^{+} T cells (**B**), CD8^{+} IFN-γ^{+} (**C**) and CD8^{+} TNF-α^{+} (**D**) in *Ogt*$^{-/-}$ rescued MC38 tumors isolated at day 18 post-tumor inoculation. **E-F**) Flow cytometry analysis

bioRxiv preprint doi: https://doi.org/10.1101/2023.12.14.571787; this version posted June 11, 2024. The copyright holder for this preprint (which was not certified by peer review) is the author/funder. This article is a US Government work. It is not subject to copyright under 17 USC 105 and is also made available for use under a CC0 license.

showing percentage of CD8^{+} IFN-γ (**E**), CD8^{+} TNF-α^{+} (**F**) in *Ogt*$^{-/-}$*cGAS*$^{-/-}$ or *Ogt*$^{-/-}$*Sting*$^{-/-}$ double knockout MC38 tumors in C57BL/6J mice, subcutaneous tumor isolated at day 18 post-tumor inoculation. **G-H)** Tumor volume, weight of *Ogt*$^{+/+}$ or *Ogt*$^{-/-}$ LLC tumors injected with control IgG or anti-PD-L1 antibody at day 7, 10 and 13 post tumor inoculation in C57BL/6J mice, and mice survival, n=5 respectively. Data are representative of two or three experiments. Statistical significance was determined by unpaired Student's t-test, one-way ANOVA, two-way ANOVA, *$p < 0.05$, **$p < 0.01$, ***$p < 0.001$, ns, no significant difference. Data represent the mean of ± SD.

bioRxiv preprint doi: https://doi.org/10.1101/2023.12.14.571787; this version posted June 11, 2024. The copyright holder for this preprint (which was not certified by peer review) is the author/funder. This article is a US Government work. It is not subject to copyright under 17 USC 105 and is also made available for use under a CC0 license.

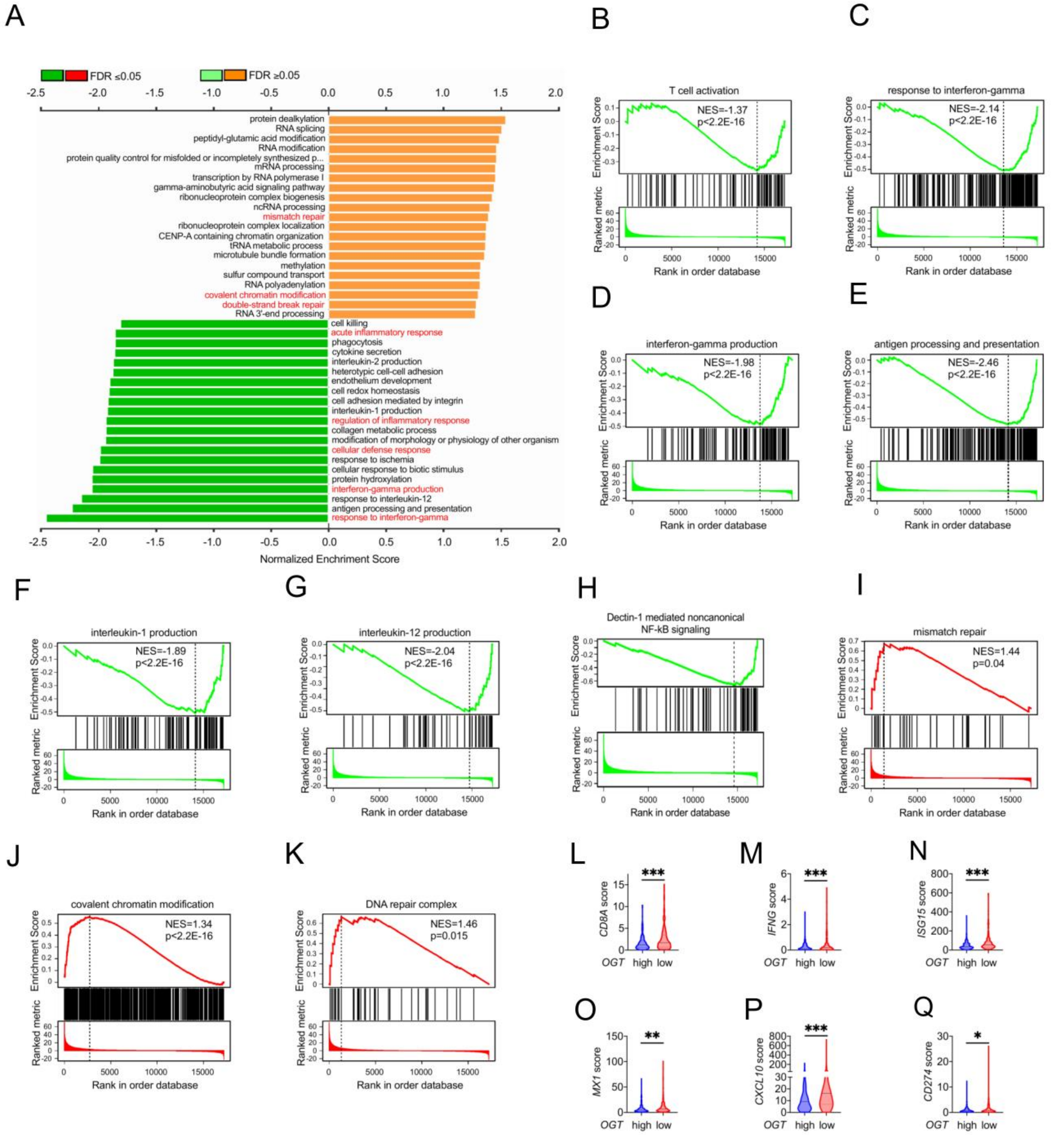

Supplemental Fig. 9 *OGT* expression is negatively related to CD8^{+} T cells infiltration in human colorectal cancer.

A) Gene Ontology (GO) enrichment and pathway analysis in *OGT* high and *OGT* low patients. **B-K)** GSEA analysis in *OGT* high and *OGT* low patients. T cells activation (**B**), response to interferon-gamma (**C**), interferon-gamma production (**D**), antigen processing and presentation (**E**),

bioRxiv preprint doi: https://doi.org/10.1101/2023.12.14.571787; this version posted June 11, 2024. The copyright holder for this preprint (which was not certified by peer review) is the author/funder. This article is a US Government work. It is not subject to copyright under 17 USC 105 and is also made available for use under a CC0 license.

interleukin-1 production (**F**), interleukin-12 production (**G**), dectin-1 mediated noncanonical NF-κB signaling (**H**), mismatch repair (**I**), covalent chromatin modification (**J**) and DNA repair complex (**K**) in *OGT* high and *OGT* low patients. **L-Q)** RNAseq analysis of mRNA expression pattern in *OGT* high and *OGT* low patients, CD8A (**L**), *IFNG* (**M**), *ISG15* (**N**), *MX1* (**O**), *CD274* (**P**) and *CXCL10* (**Q**) mRNA expression patterns in *OGT* high and *OGT* low patients. Statistical significance was determined by Pearson test, unpaired Student's t-test, *$p < 0.05$, **$p < 0.01$, ***$p < 0.001$, ns, no significant difference. Data represent the mean of ± SD.

bioRxiv preprint doi: https://doi.org/10.1101/2023.12.14.571787; this version posted June 11, 2024. The copyright holder for this preprint (which was not certified by peer review) is the author/funder. This article is a US Government work. It is not subject to copyright under 17 USC 105 and is also made available for use under a CC0 license.

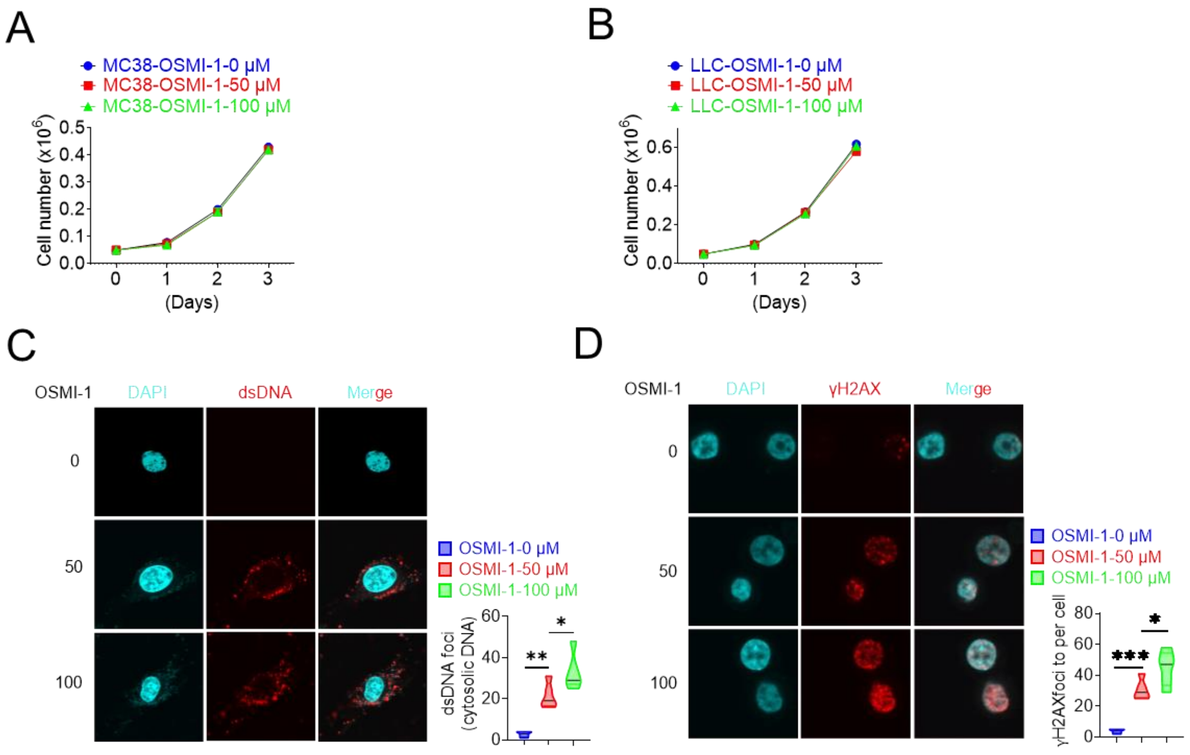

Supplemental Fig. 10 OSMI-1 could significantly induce a high percentage of DNA damage. A-B) The cell proliferation in different treatments in *vitro*. **A)** MC38 cell proliferation, **B)** LLC cell proliferation. **C)** The extranuclear dsDNA was measured by anti-dsDNA fluorescence staining treated with 50 μM and 100 μM in LLC cells, respectively. **D)** The γH2AX expression was measured anti-γH2AX fluorescence staining treated with 50 μM and 100 μM in LLC cells, respectively. Data are representative of three experiments. Statistical significance was determined by one-way ANOVA, **p* < 0.05, ***p* < 0.01, ****p* < 0.001, ns, no significant difference. Data represent the mean of ± SD.

bioRxiv preprint doi: https://doi.org/10.1101/2023.12.14.571787; this version posted June 11, 2024. The copyright holder for this preprint (which was not certified by peer review) is the author/funder. This article is a US Government work. It is not subject to copyright under 17 USC 105 and is also made available for use under a CC0 license.

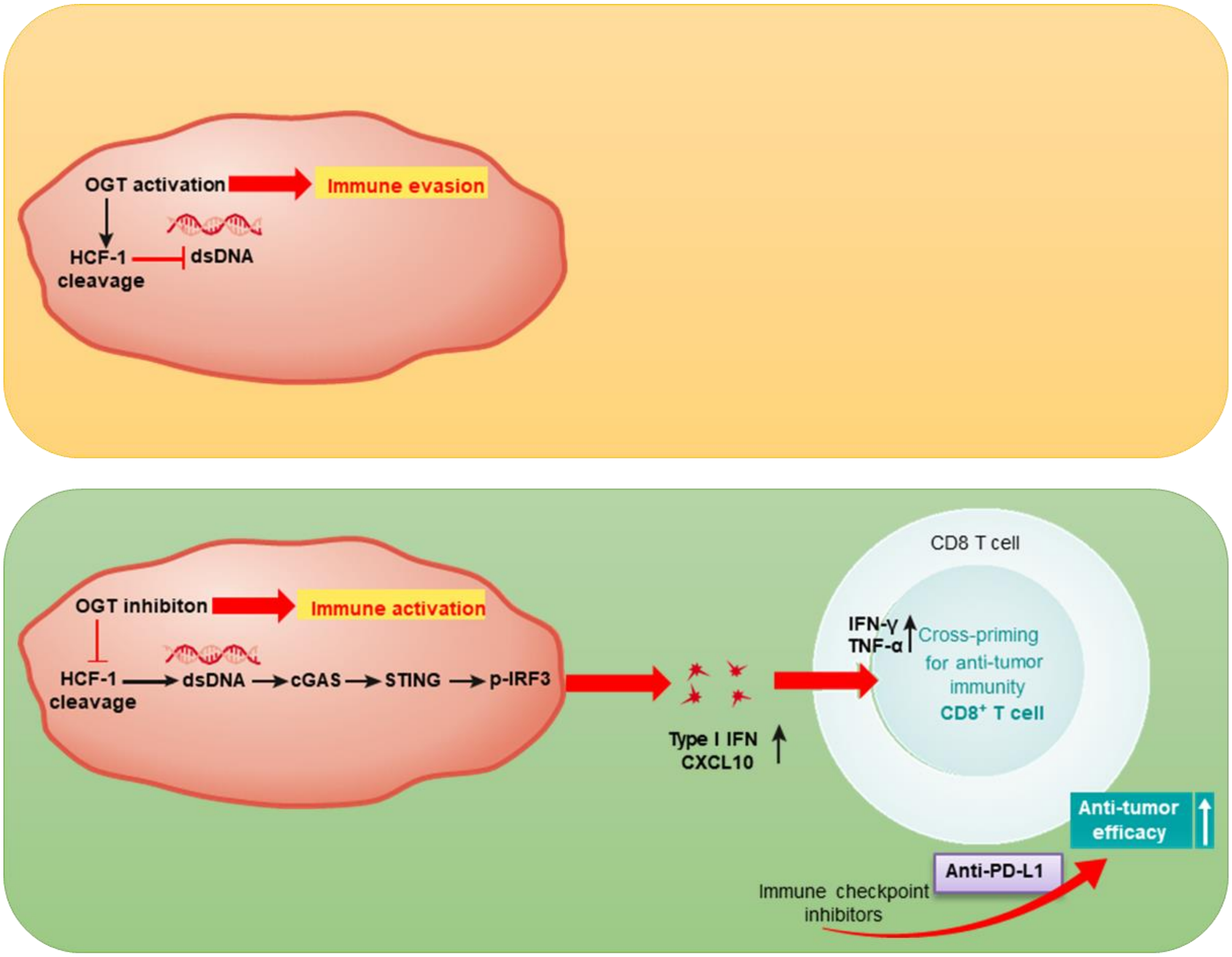

Supplementary Fig. 11 A schematic of critical role of OGT-mediated antitumor immunity.

Inhibition of O-GlcNAc transferase promotes the activation of cGAS-STING pathway and the production of type I interferon, which enhances CD8 T cells dependent antitumor immunity.

bioRxiv preprint doi: https://doi.org/10.1101/2023.12.14.571787; this version posted June 11, 2024. The copyright holder for this preprint (which was not certified by peer review) is the author/funder. This article is a US Government work. It is not subject to copyright under 17 USC 105 and is also made available for use under a CC0 license.

Table S1. Primer sequences for genotype

Genes	Sequence	Size
Ogt$^{fl/fl}$	Forward CATCTCTCCAGCCCCACAAACTG	WT 332 bp,
	Reverse GACGAAGCAGGAGGGGAGAGCAC	Mutant 487 bp
Villin-Cre	WT Forward TATAGGGCAGAGCTGGAGGA	WT 182 bp,
(△*IEC*)	Mut Forward AGGCAAATTTTGGTGTACGG	Mutant 150 bp
	Common Rev GCCTTCTCCTCTAGGCTCGT	
Apc^{min}	WT Forward GCCATCCCTTCACGTTAG	WT 619 bp,
	Mut Forward TTCTGAGAAAGACAGAAGTTA	Mutant 320 bp
	Common Rev TTCCACTTTGGCATAAGGC	

Table S2. Related to Experimental Procedures. Primer sequences for RT-PCR

Genes	Forward	Reverse
Mouse *Ifna4*	CCTGTGTGATGCAGGAACC	TCACCTCCCAGGCACAGA
Mouse *Ifnb1*	ATGAGTGGTGGTTGCAGGC	TGACCTTTCAAATGCAGTAGAGTCA
Mouse *Ifng*	TCAAGTGGCATAGATGTGGAAGAA	TGGCTCTGCAGGATTTTCATG
Mouse *Il1a*	GCACCTTACACCTACCAGAGT	AAACTTCTGCCTGACGAGCTT
Mouse *Il1b*	CTCATTGTGGCTGTGGAGAAG	ACCAGCAGGTTATCATCATCAT
Mouse *Il6*	AGCTGGAGTCACAGAAGGAG	AGGCATAACGCACTAGGTTT
Mouse *Il10*	CCCTTTGCTATGGTGTCCTT	TGGTTTCTCTTCCCAAGACC
Mouse *Il12a*	GAGGACTTGAAGATGTACCAG	TCCTATCTGTGTGAGGAGGGC
Mouse *Tnfa*	GTCAGGTTGCCTCTGTCTCA	TCAGGGAAGAGTCTGGAAAG
Mouse *Cxcl10*	CCTGCCCACGTGTTGAGAT	TGATGGTCTTAGATTCCGGATTC
Mouse *Isg15*	TGGAAAGGGTAAGACCGTCCT	GGTGTCCGTGACTAACTCCAT
Mouse *Mx1*	GGGGAGGAAATAGAGAAAATGAT	GTTTACAAAGGGCTTGCTTGCT
Mouse *Actb*	AGGGCTATGCTCTCCCTCAC	CTCTCAGCTGTGGTGGTGAA
Human *IFNB1*	CATTACCTGAAGGCCAAGGA	CAATTGTCCAGTCCCAGAGG
Human *ISG15*	CTGAGAGGCAGCGAACTCAT	AGCATCTTCACCGTCAGGTC
Human *MX1*	AGAGAAGGTGAGAAGCTGATCC	TTCTTCCAGCTCCTTCTCCTG
Human *CXCL10*	CTCCAGTCTCAGCACCATGA	GCTCCCCTCTGGTTTTAAGG
Human *GADPH*	ATGACATCAAGAAGGTGGTG	CATACCAGGAAATGAGCTTG

bioRxiv preprint doi: https://doi.org/10.1101/2023.12.14.571787; this version posted June 11, 2024. The copyright holder for this preprint (which was not certified by peer review) is the author/funder. This article is a US Government work. It is not subject to copyright under 17 USC 105 and is also made available for use under a CC0 license.

Table S3. Related to CRISPR/Cas9. Primer sequences for molecular cloning

Genes	Forward	Reverse
Ogt gRNA#1	CACCGTGCCCACGGAAGACGCCATC	AAACGATGGCGTCTTCCGTGGGCAC
Ogt gRNA#2	CACCGGCTCCAGATGGCGTCTTCCG	AAACCGGAAGACGCCATCTGGAGCC
mMavs gRNA#1	ACCGGCCGTCGCGAGGATGTCTGG	AACCCAGACATCCTCGCGACGGCC
mMavs gRNA#2	CACCGGATACCCTCTCCTAACCAGC	AACGCTGGTTAGGAGAGGGTATCC
mCgas gRNA	CACCGATATGGAAGATCCGCGTAGA	AAACTCTACGCGGATCTTCCATATC
mSting gRNA	CACCGGCTGGATGCAGGTTGGAGTA	AAACTACTCCAACCTGCATCCAGCC
hcGAS gRNA	CACCGAAGTGCGACTCCGCGTTCAG	AAACCTGAACGCGGAGTCGCACTT
hSTING gRNA	CACCGGGATGTTCAGTGCCTGCGAG	AAACCTCGCAGGCACTGAACATCC

Table S4. Mass spectrometry assay of OGT interactome
